# Rapid Access to Photoswitchable RNA Binders: Fluorination Enhances Protein Rescue by Exon Inclusion

**DOI:** 10.1002/chem.71371

**Published:** 2026-07-12

**Authors:** Lei Zhang, Nemanja Djokovic, Alexander Herr, Anna Wacker, Emily Swanson Hay, Daniel Santana, Amanda E. Hargrove, Harald Schwalbe, Katarina Nikolic, Frank Abendroth, Olalla Vázquez

**Affiliations:** ^1^ Chemistry Department Marburg University Marburg Germany; ^2^ Department of Pharmaceutical Chemistry Faculty of Pharmacy University of Belgrade Belgrade Serbia; ^3^ Institute For Organic Chemistry and Chemical Biology Center for Biomolecular Magnetic Resonance (BMRZ) Goethe University Frankfurt Frankfurt Germany; ^4^ Department of Chemical and Physical Science University of Toronto Toronto Ontario Canada; ^5^ Center For Synthetic Microbiology (SYNMIKRO) Marburg Germany; ^6^ Max Planck Institute for Terrestrial Microbiology Marburg Germany

**Keywords:** fluorination, photoswitches, RNA binders, SMA, splicing

## Abstract

Targeting RNA is a rich, yet largely untackled opportunity for controlling biological functions, with high potential for therapeutic intervention. However, it remains inherently challenging. Beyond RNA structural diversity, functional RNA motifs are frequently context‐dependent and transient, complicating the rational design of selective small‐molecule binders. We here develop novel photoswitchable ligands to target RNA. They offer highly desirable, precise intervention by enabling light‐controlled regulation of both direct RNA interactions and downstream events. Unfortunately, access to such photoswitchable RNA molecular tools is scarce, requiring complex and lengthy synthesis routes. We present a readily adaptable platform for the straightforward synthesis of photoswitchable RNA binders capable of targeting pre‐mRNA and restoring functional survival motor neuron (SMN) protein levels by rescuing exon inclusion. Evaluation of our compounds demonstrated that both fluorination and heteroaryl groups (e.g., benzo‐ and thioxozaole) enhance binding affinity to the targeted dsRNA with *in‐cellulo* activity. Importantly, molecular recognition and structure–activity relationships were rationalized through a combination of computational studies and NMR spectroscopy.

## Graphical abstract

An adaptable approach, many light‐switchable RNA binders. We report a versatile strategy for rapidly synthesizing photoswitchable RNA binders. The ligands can target SMN2 pre‐mRNA, restoring the levels of the corresponding protein by exon‐inclusion rescue. We demonstrated that both fluorination and heteroaryl groups enhance binding and cellular activity.

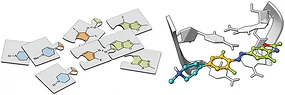

## 1 Introduction

Proteins play crucial roles in biological function. Indeed, the functional roles of proteins are the main factors responsible for the optimal orchestration that determines human health [1, 2]. Thus, proteins have been—and remain—the main workhorse of the pharmaceutical industry to overcome disease states. Traditionally, activity inhibition of the protein of interest was the most common strategy for developing therapeutics. However, we now know that this approach is far too simplistic, despite its unquestionable success.

Given the key functional role of proteins in cellular homeostasis, the number of transcribed genes is surprisingly low. It is RNA splicing that contributes to proteome diversity and proteostasis [3]. Splice versions of a gene result in protein isoforms with different properties, which often affect their function. Consequently, targeting splicing provides an early intervention point to combat disease‐induced disorders. Thus, alteration of the output of RNA splicing has been successfully applied to cancer and neuronal diseases [4, 5, 6].

Beyond the therapeutic potential, splicing modifiers are immediately valuable chemical biology tools for regulating protein production. Thus, the X^on^ system, which combines the splicing modulator branaplam with engineered SF3B alternative splice sites, achieved a precise externally controlled protein production in vivo [7]. We have also recently contributed to this field by adding an extra layer of control, that is, photocontrol. We reported the first photoswitchable splicing modifier [8]. Our compound **1** can restore the levels of the molecular target of spinal muscular atrophy (SMA), that is, the survival motor neuron (SMN) protein, in human primary patient cells in a light‐dependent fashion. Together, these studies lay the foundation that splice‐switching molecules can provide programmable control over gene expression with unprecedented spatiotemporal resolution, enabling powerful opportunities for biotechnology [9] and chemical biology [10].

**FIGURE 1:**
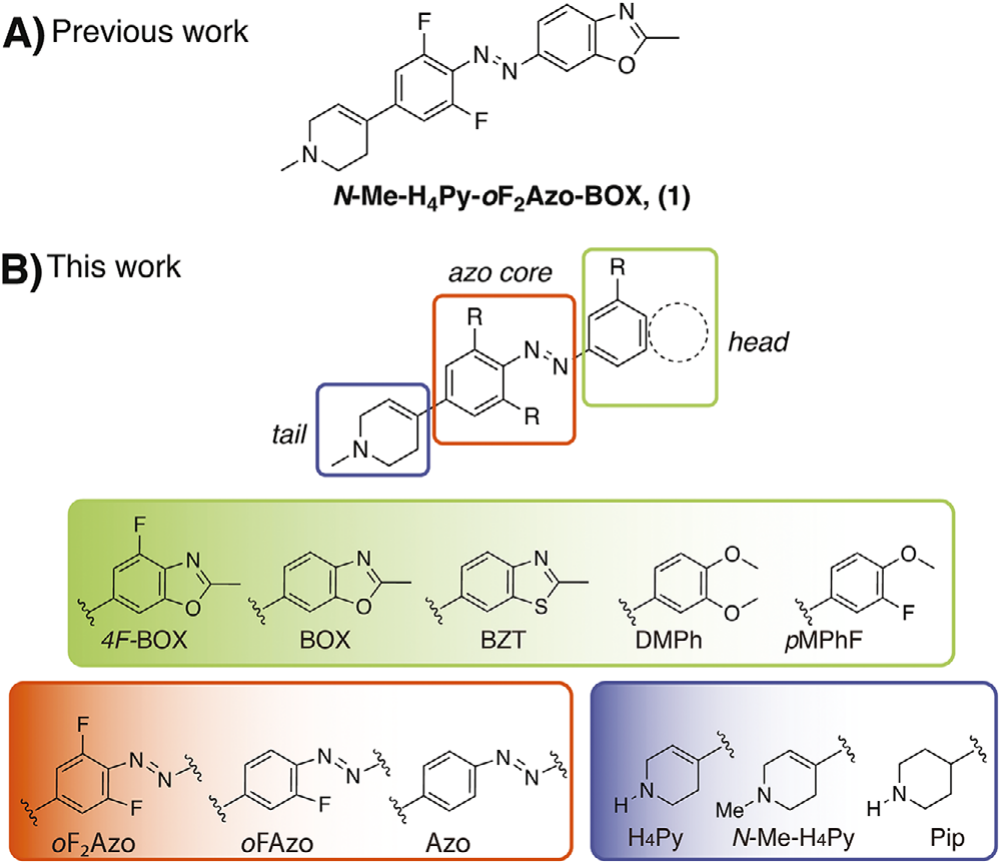
(A) Structure of our reported photoswitchable splicing modulator **1** capable of increasing the levels of survival motor neuron (SMN) protein by exon inclusion. (B) Library of potential light‐activated SMN inducers. BOX: benzoxazole; BZT: benzothiazole; DMPh: dimethoxyphenyl; MPhF: methoxy‐fluorophenyl; Py: pyridyl; Pip: piperidyl.

Here, we describe a versatile synthetic strategy for the easy and efficient access to photoswitchable RNA binders. From commercial material, this synthetic strategy allows us to explore novel functionalizations, retaining the azoarene core while affecting both steric and electronic properties to ultimately influence RNA:ligand interactions. Compared to the parental **1**, our library (Figure 1) provides azoarenes with improved bioactivity on splicing output, surpassing **1** in protein synthesis capacity. We also used molecular simulations and NMR to gain insight into molecular mechanisms and to identify the major interactions involved in molecular recognition. We reveal that ligand fluorination is a key feature for biological activity.

## 2 Results and Discussion

### 2.1 A Modular Synthetic Strategy for Photoswitchable RNA Binders

The selection of the chemotype was inspired by the reported fluorobenzamide analogues, in which the incorporation of this moiety not only reduced toxic side effects but also stabilized a quasi‐planar bioactive conformation via an intramolecular hydrogen‐bonding interaction [11]. The pronounced dependence of the splicing‐modulatory activity on planarity suggested that this system would be particularly suitable for the introduction of a diazine‐containing photoswitch. We reasoned that light‐induced control over the conformation could provide novel photoswitchable RNA binders. Thus, in the lead compound series, a tail and a head functional group are connected by an azo core (Figure 1B). Based on this design, a chemical series is built in two robust chemical steps: an azo coupling and a Suzuki–Miyaura coupling (Scheme 1). This stepwise approach enables the design of a library that modifies both sides of the molecule using commercially available anilines and boronic acid pinacol esters. The library was designed to provide rational insight into the effects of steric and electronic features of substituents on binding. For instance, previous studies of SMA splicing modifiers demonstrated that *N*‐methylated compounds undergo demethylation, yielding more potent metabolites in fibroblasts [12]. Considering the accessible building blocks on the tail, we planned novel derivatives in which the tetrahydropyridyl (*N*‐Me‐H_4_Py) group is replaced by a piperidyl (Pip) group and *N*‐dealkylated (H_4_Py) modifications. According to our previous results [8], **1** appeared to be involved in both electrostatic (e.g., between its *N*‐Me‐H_4_Py group and the phosphodiesters of the pseudouridine‐containing dsRNA) and π–π stacking interactions. Therefore, we designed a library to tackle these interactions by affecting not only the basicity of the ligands through methylation but also the electronic properties of the aromatic moieties. Thus, fragments such as benzothiazole and methoxybenzol, which can participate in donor‐acceptor π–π stacking interactions, were considered in the head part. Finally, the effect of fluorine was evaluated by preparing both monosubstituted and unsubstituted azo cores as well as the fluorinated benzoxazole compound.

**SCHEME 1:**
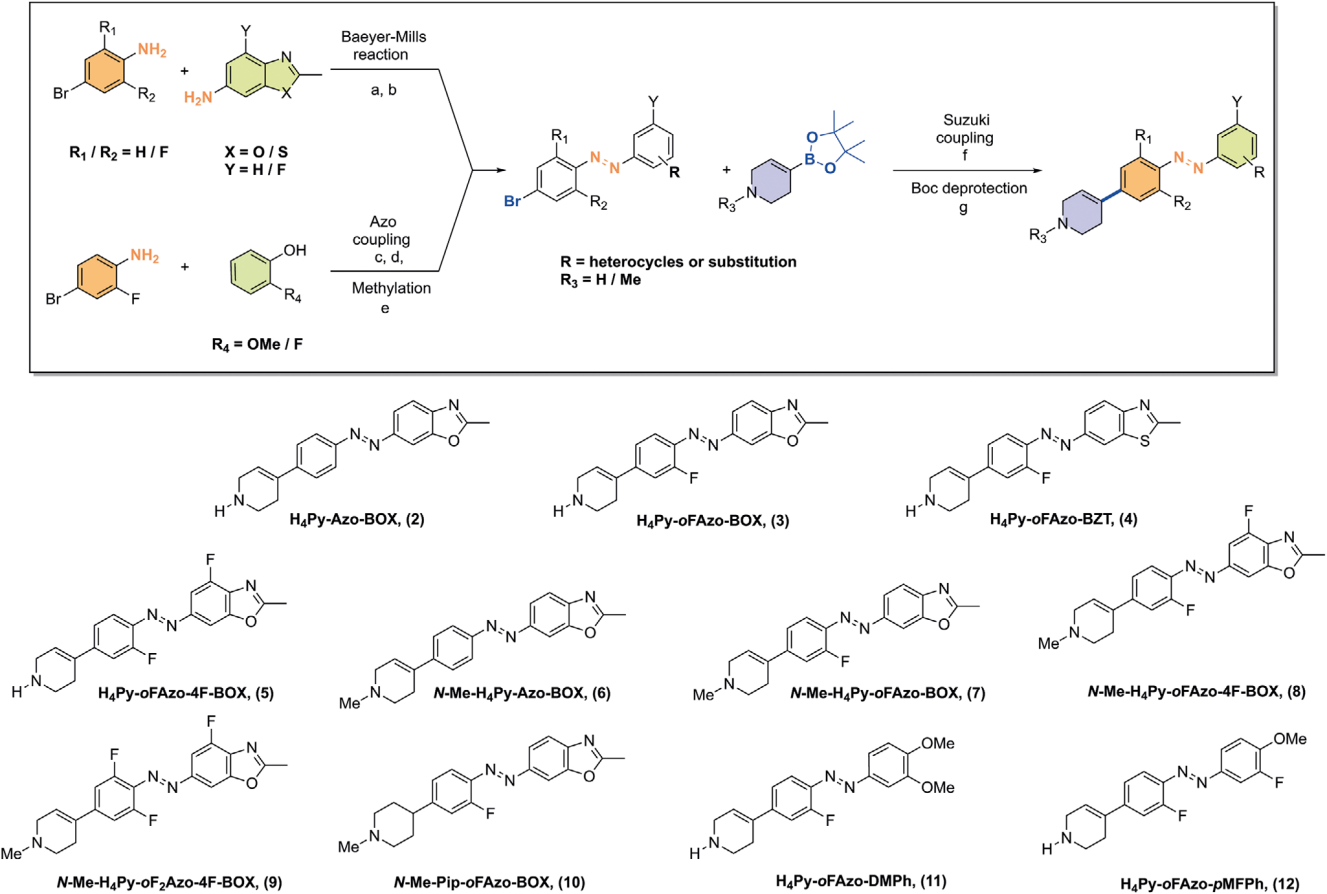
General route; conditions and reagents: Baeyer‐Mills reaction: (a) Oxone, DCM/H_2_O 4:1, rt, 16 h; (b) AcOH, rt, 48 h. Azo coupling: (c) conc. HCl/H_2_O 1:3, NaNO_2_, 0°C, 30 min; (d) corresponding phenol, NaOH, 0°C, 30 min. Methylation: (e) Cs_2_CO_3_, acetone, rt, 2 h; MeI, 60°C, 2 h. Suzuki–Miyaura coupling: (f) Pd(dppf)Cl_2_, K_3_PO_4_·3H_2_O, dioxane/H_2_O 4:1, 85°C, 16 h; Boc deprotection: (g) TFA/DCM 1:1, rt, 2 h.

First, the corresponding azo compounds were obtained by either a Baeyer–Mills reaction (compounds **1–10**) or a diazonium coupling (**11** and **12**) in acceptable/good yields (43%–87%) (Scheme 1). Afterwards, a Suzuki–Miyaura reaction enabled the functionalization of the tail of the molecules. We used Boc‐protected amines for the dealkylated molecules (compounds **2**–**5**), whose final hydrolysis was performed under standard TFA conditions, yielding compounds in good yields (70%–90%). This general route worked for all cases except **10** due to the incompatibility of cross‐coupling conditions for carbons with different hybridisation [13]. This issue was easily solved by exchanging the reaction order. In addition, we used phenol precursors for the methoxy azobenzene molecules **11** and **12** because this allows an effective reaction of diazotization and aromatic nucleophilic substitution in one pot. The subsequent methylation using methyl iodide was successful (∼90% yield) and compatible with the fluorine substitution. Unfortunately, our attempts to obtain an *ortho*‐difluoroazoarene bearing a secondary amine resulted in diazinyl bond oxidation, even under mild conditions such as low reaction temperature, low TFA concentration, anhydrous HCl in dioxane, and glacial acid.

### 2.2 Photochemical Characterisation

Once the compounds were synthesized, we evaluated their photochemical properties under physiological conditions (Table 1). UV–vis spectra showed that all compounds undergo reversible photoisomerization under irradiation (Tables S2–S4), with the *trans* photostationary state (PSS*_trans_)* being the thermodynamically most stable. HPLC measurements showed satisfactory isomerization ratios in all cases (Table S4). Interestingly, the weak band assigned to the n→π* transition at *λ*_max_ ∼ 430 nm could hardly be detected in the *trans* isomers. On the contrary, the characteristically intense band at *λ*_max_ ∼ 350 nm due to the π→π* transition was always clearly observable. Furthermore, all compounds undergo fast (1 min irradiation at 365 nm) *cis*‐to‐*trans* isomerization. Reversible photoisomerization cycles up to at least 20 times were conducted without any degradation (Table S3). In comparison with **1**, as expected, the methoxyphenyl photoswitches (**11** and **12**) are the ones with the most red‐shifted π → π* absorption band [14]. *Ortho*‐fluorination (**1**, **9**) highly increases both the extinction coefficients and the stability to the spontaneous thermal back *cis*‐to‐*trans* relaxation (Table 1) [15]. This latter tendency was also observed for the left‐hand piperidinyl compound (**10**). Regarding the quantum yields (*Φ*), although there is no clear general tendency, we observed that the values are overall higher than those of unmodified azobenzenes, especially for irradiation in the π→π* band [16]. The saturated piperidine derivative **10** exhibited the highest quantum yields for both forward (*trans*→*cis*) and reverse (*cis*→*trans*) photoisomerization. In contrast, the tri‐fluorinated compound **9** has the lowest *Φ_trans_*_→_ *_cis_*. Fluorination is known to reduce fluorescence and affect photoisomerization by altering the conical intersection [17]. Nonetheless, determined quantum yields *(Φ*) remain within the typical range. Further investigations would be required to rationalize these findings. Importantly, measured lifetimes (*t*_1/2_) are compatible with the temporal resolution needed for dynamic control of protein expression by RNA splicing (*t*_1/2_> 6 h).

**TABLE 1:** Photochemical properties of the library.

Compound	X	Y	R_1_	R_2_	R_3_	*ε* [M^−1^·cm^−1^]^a^	*λ*_max_ (π → π*) *trans* [nm]^b^	Photoisomerization quantum yield^b^		PSD (% *cis*)^c^		*cis t*_1/2_ (h)^d^
								*Φ_trans→cis_*	*Φ_cis→trans_*	365 (nm)	525 (nm)	
**1**	O	H	F	F	Me	12678 ± 123	349	0.42 ± 0.02	0.32 ± 0.02	90	45	111.9
**2**	O	H	H	H	H	6335 ± 106	349	0.32 ± 0.01	0.45 ± 0.02	85	23	20.7
**3**	O	H	H	F	H	6020 ± 39	361	0.32 ± 0.01	0.28 ± 0.01	91	38	28.5
**4**	S	H	H	F	H	5822 ± 65	353	0.34 ± 0.01	0.24 ± 0.01	88	30	29.0
**5**	O	F	H	F	H	5507 ± 27	359	0.48 ± 0.02	0.37 ± 0.02	84	26	25.9
**6**	O	H	H	H	Me	7781 ± 77	357	0.27 ± 0.01	0.53 ± 0.03	77	22	17.6
**7**	O	H	H	F	Me	7876 ± 39	361	0.33 ± 0.01	0.15 ± 0.01	88	45	20.4
**8**	O	F	H	F	Me	7914 ± 60	361	0.38 ± 0.01	0.49 ± 0.02	92	24	18.0
**9**	O	F	F	F	Me	12132 ± 123	353	0.083 ± 0.01	0.23 ± 0.01	78	34	70.1
**10**	O	H	H	F	Me	6508 ± 40	349	0.47 ± 0.02	0.58 ± 0.03	92	37	93.6
		**R_42._**	**R_1_**	**R_2_**	**R_3_**							
**11**		OMe	H	F	H	5439 ± 35	377	0.54 ± 0.02	0.44 ± 0.02	78	23	8.9
**12**		F	H	F	H	5722 ± 53	366	0.38 ± 0.01	0.49 ± 0.02	82	24	14.5

Maximum absorption wavelength (***λ*_max_**); molar attenuation coefficient (***ε***), photostationary state distribution upon irradiation (PSD).

^a^ In DMSO.

^b^ In phosphate buffer (10 mM NaH_2_PO_4_, 150 mM NaCl, pH 7.4).

^c^ Determined by reverse‐phase (RP) HPLC.

^d^ Derived by nonlinear curve fitting with software Graphpad Prism 8.

### 2.3 Functional Validation

Next, the RNA‐binding capabilities at the PSD, with a predominance of the thermodynamically stable *trans* isomer, were initially evaluated using the indicator displacement assay (IDA) with RiboGreen [18]. After determining the indicator's binding constant to the SMN2 RNA target (1.1 ± 0.2 µM), the binding affinity of the *trans* isomers was quantified by the concentration where 50% (CD_50_) of RiboGreen was displaced (Table S9). The obtained value for the parental compound **1** was consistent with our previous ^19^F‐NMR titrations [8] (53 ± 20 µM vs. 65 ± 8 µM). Overall, differences in binding across the library were modest, ranging from 20 to 150 µM. Additionally, three molecules with moderate CD_50_ values, including **1**, were evaluated in their *cis* form as predominant PSD. For these three molecules, the *trans* isomers displayed stronger binding, as expected, due to a more rod‐like conformation, which has been observed previously in small‐molecule:RNA interactions [19, 20, 21]. The slight differences, together with the intrinsic complexity of the spliceosome machinery, suggested that biophysical measurements should be considered alongside activity (Figure 2, and Tables 2 and S10). Importantly, the biological consequences of splicing modulators cannot be understood solely in terms of RNA targeting [9, 22]. Mechanistic studies of risdiplam [23], branaplam [12, 24], and related SMN‐C derivatives have revealed that their activity arises from a complex interplay of effects, including stabilization of the transient RNA duplex formed between the 5′ splice site and U1 snRNA, modulation of local RNA secondary structure, and altered recruitment of splicing regulatory proteins such as FUBP1 and KHSRP [25]. Furthermore, distinct chemotypes can act through overlapping yet non‐identical mechanisms, targeting different structural elements within the SMN2 pre‐mRNA while ultimately converging on enhanced exon 7 inclusion. Splice correction results from the perturbation of a dynamic RNA–protein network rather than from simple ligand–RNA recognition. Consequently, we used a bioluminescent reporter assay to evaluate the ability of the entire library to restore protein expression in HEK293T cells by correcting splicing [26]. In this system, increased luminescence directly reflects enhanced enzymatic activity, driven by increased protein translation from SMN2 exon 7 inclusion. Structural differences revealed clear structure–activity relationships. Azobenzene derivatives **11** and **12** showed reduced turn‐on, hindering EC_50_ determinations. In contrast, most benzoxazole (or benzothiazole) derivatives displayed an increment of activity, likely due to their extended conjugated π‐system that favors π–π stacking and the presence of electronegative heteroatoms that increase hydrogen‐bond acceptor capacity and potential binding affinity. These results reinforce heteroaryl scaffolds as effective chemotypes for modulating SMA splicing. Reduction of the double bond in piperidine derivative **10**, relative to **7**, had a drastic effect, which may be due to a decreased planarity while increasing rotational freedom of the piperidyl group, weakening interactions with SMN2 mRNA. As expected, the photostationary state (PSS) where the planar *trans* isomer is the majority, that is, non‐irradiated, is the most potent. Interestingly, in this context, fluorination emerges as another key factor of activity: non‐fluorinated compounds **2** and **6** showed little increase in protein expression, whereas this increased directly in proportion to F content, surpassing our previously reported compound **1**. Regarding the distinction between PSS, the results were modest. However, as we observed in our previous work, the inherently nonlinear nature of biological interactions demonstrated that small differences (∽ 2‐fold) in target engagement can be amplified and ultimately result in pronounced effects such as restoration of SMN protein levels in primary SMA patient cells [8]. Importantly, although **9** substantially increased overall protein production compared to the parental compound **1**, it had comparable photoswitchable control. These results indicate that other photoswitchable scaffolds may be further considered in future studies.

**FIGURE 2:**
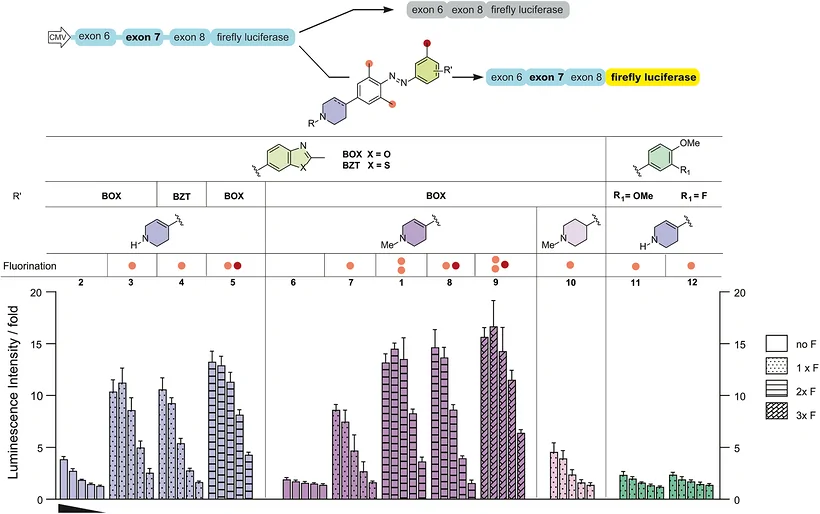
Top: Illustration of the splicing SMN2‐reporter gene assay. Bottom: Dose‐dependent induction of luciferase activity from the SMN2‐Luc reporter gene in HEK293T cells treated with the expected most active PSD (*trans* isomers) for 6 h. Luciferase activity is expressed as fold change relative to DMSO‐treated controls. The concentration range was identical for all compounds (6, 4, 2, 1, and 0.5 µM). Mean data points and standard deviations are derived from three independent experiments.

**TABLE 2:** Effect of the library on SMN2‐Luc protein expression.

Compound	EC_50_ (µM)^a^		Max. fold induction of protein expression
	Non‐irradiated	PSD_365nm_	
**2**	n.c.	n.c.	5.7
**6**	n.c.	n.c.	2.4
**11**	n.c.	n.c.	3.8
**12**	n.c.	n.c.	3.5
**10**	n.c.	n.c.	8.9
**3**	1.01 ± 0.18	1.32 ± 0.19	11.2
**4**	2.39 ± 0.27	2.92 ± 0.41	11.8
**5**	0.77 ± 0.05	0.85 ± 0.06	12.9
**7**	2.54 ± 0.27	3.23 ± 0.36	8.8
**1**	0.76 ± 0.14	1.93 ± 0.14	14.5
**8**	1.71 ± 0.17	2.19 ± 0.19	14.7
**9**	0.64 ± 0.10	1.07 ± 0.16	16.0

^Note:^ Luciferase activity is expressed as half maximal effective concentration (EC_50_, Table S8), including maximum protein induction fold for 6 h at 14 µM after solvent‐control normalization. Mean data points and standard deviations are derived from three independent experiments.

^a^ Calculated by exponential one‐phase decay test with software Graphpad Prism 8; n.c. not calculable.

### 2.4 Computational Insights into RNA‐Ligand Recognition

Next, molecular docking followed by molecular dynamics (MD) simulations were performed to elucidate the molecular basis of dsRNA‐ligand recognition of the library. MD analyses revealed a conserved dsRNA‐binding mode shared with our previously reported ligand **1** [8], in which ligands bind at the exon–intron junction and stabilize the bulged adenine (A_‐1_). This interaction increases hydrogen bonds within the G_‐3_/G_‐2_/A_‐1_–G_11_/U_10_/C_9_ region, thereby enhancing dsRNA stability (Figures 3 and S95–S96). The predicted mechanism is consistent with structural studies of the SMN2 splicing modifier SMN‐C5 [27]. Principal component analysis of the MD trajectories further confirmed distinct dsRNA conformational behavior upon binding of the most potent ligands compared with the *apo* state (Figure S97). Visual inspection of intermolecular interactions indicated that the protonated tetrahydropyridyl moiety engages in electrostatic interactions with the phosphate of C_8_, while the remainder of the ligand predominantly participates in π–π stacking with adjacent nucleobases (Figures 3 and S96). Consistent with this model, secondary amines were more potent than their tertiary analogues (**5** vs. **8**; **3** vs. **7**), likely reflecting the increased basicity (Table S9) and stronger electrostatic interactions, as the *N*‐methyl group was not predicted to contribute additional contacts with dsRNA (Figures 3 and S96). Fluorination of the azo core further enhanced activity, in agreement with literature reports that fluorination strengthens aromatic stacking with nucleic acid bases [28]; this effect is attributed to reinforced π–π interactions between the fluorinated azo moiety and the C_9_–C_8_ region. In line with our experiments, MD simulations highlighted the importance of the benzoxazole tail. Closer inspection of intermolecular interactions between benzoxazole head and dsRNA indicated π–π stacking of benzoxazole with bulged adenine A_‐1_ spanning across the whole heterocycle (Figures 3 and S96). According to the MD simulations, ligands lacking benzoxazole (**11** and **12**) partially or completely unbound from the putative binding site (Figures S96F and S98), which could be attributed to the lack of oxazole and extended π–π stacking interactions. Interestingly, oxazole derivatives lacking the fluorine in both—azo core and benzoxazole head (**2** and **6**) were predicted to have slightly different binding modes with the stacking occurring primarily with G_‐2_, instead of bulged adenine A_‐1_ (Figure S96E). It is also interesting to note that, within the tertiary amine series of ligands (**1** and **6–10**), fluorination of benzoxazole led to increased stacking with A_‐1_. Analogues lacking fluorine in the benzoxazole were more flexible during the course of MD simulation and were able to simultaneously interact with A_‐1_ and G_‐2_ (Figures S96 and S99). Considering the importance of stabilization of bulged adenine A_‐1_ in dsRNA base stack for the mechanism of action of SMN2 splicing modifiers, the abovementioned importance of π–π stacking interactions revealed by the molecular modeling study could provide a rationale for the future design of novel small‐molecule SMN2 splicing modifiers.

**FIGURE 3:**
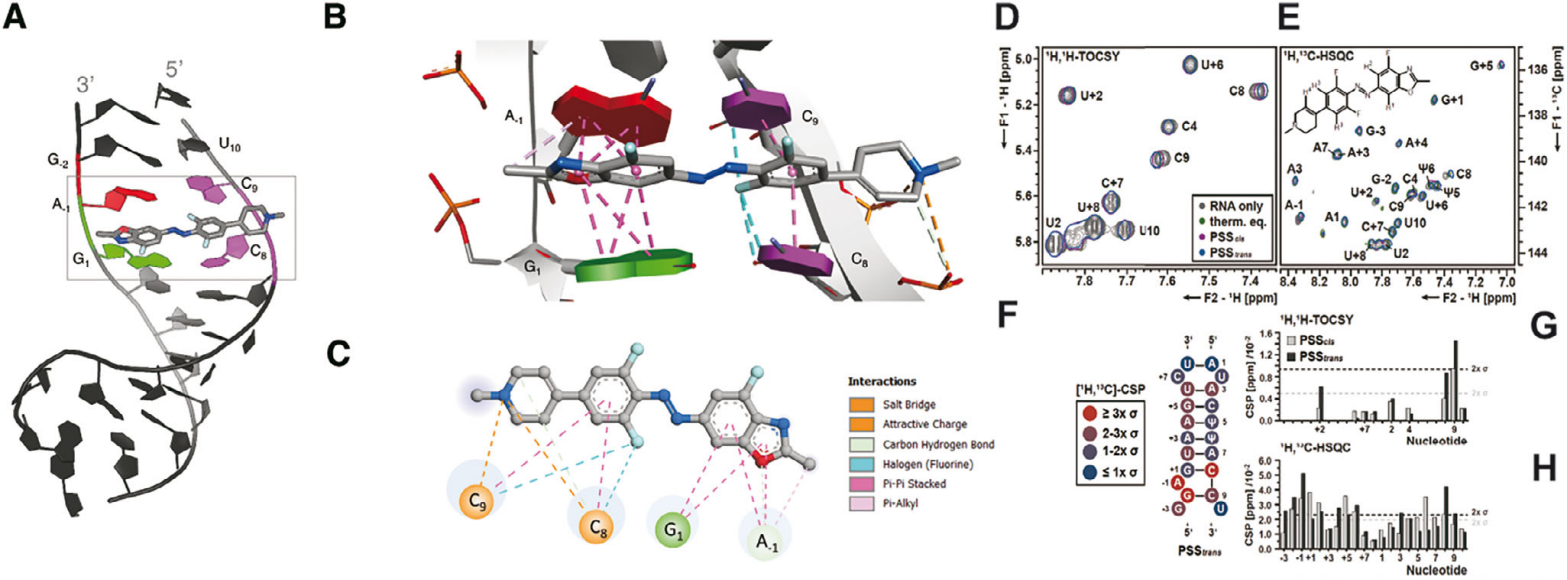
Representation of predicted binding mode and intermolecular interactions and NMR‐based binding site mapping of E7_5'ss:U1 RNA with the most potent ligand **9** (A) Overall structure of dsRNA:**9** complex; (B) Close‐up view of the most significant interactions between ligand 9 and dsRNA; (C) 2D representation of achieved interactions; (D, E) ^1^H,^1^H‐TOCSY spectra (D) and C6‐H6/ C8‐H8 region of ^1^H,^13^C‐HSQC spectra (E) of E7_5'ss:U1 RNA only (black) or in presence of 2 eq compound **9** in its thermal equilibrium (green), in PSS*_cis_* (purple) and PSS*_trans_* (blue). The structural formula of the *trans*‐stereoisomer of compound **9** is shown in the top left corner of (E); (F) Secondary structure of E7_5'ss:U1 RNA, colored with an HSQC‐based chemical shift perturbation (CSP) heatmap with red corresponding to signals with a CSP strength of over threefold the standard deviation. The CSPs were calculated from shifts of C6‐H6/ C8‐H8 resonances induced by compound **9** in its PSS*_trans_*; (G, H) Histogram of ^1^H,^1^H‐TOCSY‐ (G) or ^1^H,^13^C‐HSQC‐based (H) Euclidean distances of RNA resonances with 0 eq of **9** and with 2 eq of **9** in its PSS*_cis_* (light grey) and PSS*_trans_* (dark grey). A threshold of twofold the standard deviation was used and is highlighted in the respective color. All experiments were recorded at 298 K and 800 MHz with samples of 500 µM RNA (without ligand) and 200 µM RNA with 400 µM of **9** in 25 mM KPi, 50 mM KCl, pH 7.4 with 5% DMSO‐*d_6_*. Photoisomerization of **9** was achieved by in situ irradiation with a 488 nm or 355 nm laser for 400 ms or 2,000 ms total irradiation time over multiple irradiation intervals (200 ms).

### 2.5 NMR Mapping of RNA‐Ligand Interactions

Finally, we conducted NMR measurements with the most promising compound **9** to map the binding site to E7_5'ss:U1 RNA [8] (Figure 3D–H). Our experiments clearly showed its specific binding to the A‐bulge (A_‐1_), as evidenced by pronounced chemical shift perturbations (CSPs) that are substantially larger than those observed for other signals. In addition, significant CSPs above the threshold were detected for the opposing cytosines in both ^1^H, ^1^H‐TOCSY and ^1^H, ^13^C‐HSQC spectra, further supporting a well‐defined binding interface. Beyond these localized effects, the observed perturbation pattern indicates that ligand binding induces not only strong local stabilization of the A‐bulge but also more global conformational changes across the RNA scaffold. While the NMR data alone do not unequivocally discriminate between binding of the stereoisomers, the in situ photoirradiation experiments confirmed an isomer‐dependence of **9** on binding of the E7_5'ss:U1 RNA. The *cis*‐stereoisomer induces CSPs across the entire RNA stem, while *trans*‐**9**‐induced CSPs are clustered around A_‐1_ and are significantly higher in intensity (Figures 3 and S100). Our comparative NMR data show that the thermodynamically favored *trans*‐isomer of **9** is bound both preferentially and more selectively, two important preconditions for targeted modulation of RNA function, and provide complementary evidence of isomer‐dependent modulation.

## 3 Conclusion

In summary, we developed a versatile yet simple synthetic platform for photoswitchable RNA‐binding ligands based on commercial scaffolds. This approach relies on only two steps; that is, a Baeyer–Mills coupling and a Suzuki–Miyaura cross‐coupling, which ensure broad scope and applicability. Our results demonstrate that heteroaryls such as benzoxazole and benzothiazole are effective for both SMN2 pre‐mRNA targeting and restoring deficient protein levels by splice correction. Interestingly, strategic fluorination fine‐tunes the photochemical properties as well as it strengthens binding to the A‐bulge motif in the target dsRNA—key interaction for risdiplam and SMN‐C ligands related to the stabilization of the transient E7_5'ss:U1 RNA splice‐site duplex. Thus, the incorporation of fluorine into such a scaffold offers unique opportunities with negligible steric effects [29], facilitating the study of RNA interactions [30, 31]. Of note, splicing correction is a multidimensional phenomenon arising from cooperative effects on RNA conformation, U1 snRNP engagement, splicing‐factor recruitment, and related processes. Therefore, it is important to combine rational chemical design with straightforward synthesis to generate RNA‐targeting libraries. Moving forward in this direction holds the promise for future applications in other splicing systems and premature termination codon (PTC)‐directed transcript engineering, which highlights the broader potential of RNA‐splicing modulation [32, 33]. Altogether, a foundation will be established for the development of next‐generation small‐molecule tools to control RNA splicing with spatial and temporal precision.

## Conflicts of Interest

The authors declare no conflicts of interest.

## Supporting information




**Supporting File 1**: chem71371‐sup‐0001‐SuppMat.pdf

## Data Availability

The data that support the findings of this study are openly available in ChemRxiv at https://doi.org/10.26434/chemrxiv.10001843/.
