# Supplementary material for: Rapid Access to Photoswitchable RNA Binders: Fluorination Enhances Protein Rescue by Exon Inclusion
Source: Chemistry. 2026 Jul 12;32(39):e71371. doi: 10.1002/chem.71371 (PMC13715466; doi:10.1002/chem.71371)
Supplement: Supplementary file 1 [file CHEM-32-e71371-s001.pdf]

Supporting Information
©Wiley-VCH 2021
69451 Weinheim, Germany

# Rapid Access to Photoswitchable RNA Binders: Fluorination Enhances Protein Rescue by Exon Inclusion

Lei Zhang,[a] Nemanja Djokovic,[b] Alexander Herr,[c] Anna Wacker,[c] Emily Swanson Hay,[d] Daniel Santana,[d] Amanda E. Hargrove,[d] Harald Schwalbe,[c] Katarina Nikolic,[b] Frank Abendroth,[a]* Olalla Vázquez [a,e]*

**Abstract:** Targeting RNA is a rich, yet largely untackled opportunity for controlling biological functions, with high potential for therapeutic intervention. However, it remains inherently challenging. Beyond RNA structural diversity, functional RNA motifs are frequently context-dependent and transient, complicating the rational design of selective small-molecule binders. We here develop novel photoswitchable ligands to target RNA. They offer highly desirable, precise intervention by enabling light-controlled regulation of both direct RNA interactions and downstream events. Unfortunately, access to such photoswitchable RNA molecular tools is scarce, requiring complex and lengthy synthesis routes. We present a readily adaptable platform for the straightforward synthesis of photoswitchable RNA binders capable of targeting pre-mRNA and restoring functional survival motor neuron (SMN) protein levels by rescuing exon inclusion. Evaluation of our compounds demonstrated that both fluorination and heteroaryl groups (e.g., benzo- and thioxozaole) enhance binding affinity to the targeted dsRNA with *in-cellulo* activity. Importantly, molecular recognition and structure–activity relationships were rationalised through a combination of computational studies and NMR spectroscopy.

## Table of Contents

Table of Contents .... 2
Abbreviations .... 3
Experimental Procedures .... 5
Materials .... 5
Mass Spectrometry (MS) .... 5
Nuclear Magnetic Resonance Spectroscopy (NMR) .... 5
High Performance Liquid Chromatography (HPLC) .... 5
Ultraviolet-visible Spectroscopy (UV-vis) .... 5
Photoisomerization .... 6
UV-vis Characterization .... 6
Preparation of Photostationary States (PSS) .... 6
HPLC characterization .... 6
Photoisomerization Quantum Yield .... 6
Cell Culture .... 7
Minigene Constructs .... 7
HEK293T-SMN2-Luc Cell-Based Reporter Gene Assays .... 8
Modelling .... 9
Synthesis .... 10
Results and Discussion .... 133
Characterization .... 133
Molar Extinction Coefficient .... 133
Isomerization .... 135
Reversibility .... 137
Isomer Ratio Quantification .... 139
Thermal Stability .... 141
Photoisomerization Quantum Yield .... 143
Cell-based Assays .... 145
Cell Viability Assays .... 145
Cell-based Luciferase Assay .... 147
Computational Model Generation .... 150
NMR-based Binding Site Mapping .... 159
References .... 160

## Abbreviations

| | |
|---|---|
| °C | degrees Celsius |
| ATP | adenosine triphosphate |
| arom | aromatic |
| AU | absorbance unit |
| *rac*-BINAP | *racemic*-2,2’-bis(diphenylphosphino)-1,1’-binaphthyl |
| br | broad (in NMR spectrometry) |
| calcd | calculated |
| concn | concentration |
| CMV | cytomegalovirus |
| d | doublet (in NMR spectrometry) |
| DCM | dichloromethane |
| DMEM | Dulbecco’s modified Eagle’s medium |
| DMSO | dimethyl sulfoxide |
| DPBS | Dulbecco’s phosphate-buffered saline |
| dppf | 1,1′-bis(diphenylphosphino)ferrocene |
| DTT | 1,4-dithiothreitol |
| EDTA | ethylenediaminetetraacetic acid |
| EtOAc | ethyl acetate |
| EtOH | ethanol |
| ESI | electrospray ionization (mass spectrometry) |
| f | frequency |
| FBS | fetal bovine serum |
| Fluc | firefly luciferase |
| GAFF2 | general AMBER force field 2 |
| HEK | human embryonic kidney |
| HF | high frequency |
| HPLC | high-performance liquid chromatography |
| HRMS | high-resolution mass spectrometry |
| hygro | hygromycin |
| Hz | hertz |
| *J* | coupling constant (in NMR spectrometry) |
| K | Kelvin |
| LED | light-emitting diode |
| LINCS | LINear Constraint Solver |
| Luc | lucifease |
| m | mili ($10^{-3}$)<br>multiplet (spectral) |
| m | molar (moles per liter) |
| M | mega ($10^{6}$) |

| | |
|---|---|
| $M^+/M^-$ | molecular ion |
| MD | molecular dynamics |
| MeCN | acetonitrile |
| MeOH | methanol |
| MS | mass spectrometry |
| NheI-HF | NheI-High-Fidelity restriction enzyme |
| NMP | *N*-methyl-2-pyrrolidon |
| NMR | nuclear magnetic resonance |
| NotI-HF | NotI-High-Fidelity restriction enzyme |
| NPT | constant number of particles, pressure, and temperature |
| NVT | constant number of particles, volume, and temperature |
| n | nano ($10^{-9}$) |
| N/A | not applicable |
| PDB | protein data bank |
| PEM | particle mesh ewald |
| ppm | parts per million |
| PSS | photostationary state |
| q | quartet (in NMR spectrometry) |
| RESP | restrained electrostatic potential |
| RLU | relative light unit |
| $R_f$ | retention factor |
| RP | reversed phase |
| rt | room temperature |
| s | singlet (in NMR spectrometry) |
| SMN | survival motor neuron |
| t | triplet (in NMR spectrometry) |
| TFA | trifluoroacetic acid |
| TIP3P | transferable intermolecular potential with 3 point |
| TLC | thin-layer chromatography |
| TMS | tetramethylsilane |
| $t_R$ | retention time |
| tris | 2-amino-2-(hydroxymethyl)propane-1,3-diol |
| μ | micro ($10^{-6}$) |
| UV-vis | ultraviolet-visible |
| v/v | volume per volume |
| *δ* | NMR chemical shift in parts per million downfield from a standard (tetramethylsilane) |
| *ε* | molar extinction coefficient |
| λ | wavelength |
| ϕ | photoisomerization quantum yield |

## Experimental Procedures

### Materials

All commercially reagents were purchased from the following companies, and used without further purification: *rac*-BINAP, 4-bromoaniline, 4-bromo-2-fluoroanilinem, 4-bromo-2,6-difluoroaniline from Fluorochem (UK); 2-methyl-1,3-benzoxazol-6-amine, 2-methyl-1,3-benzothiazol-6-amine, 1-methyl-1,2,3,6-tetrahydropyridine-4-boronic acid pinacol ester and *N*-*tert*-butoxycarbony-1,2,3,6-tetrahydropyridine-4-boronic acid pinacol ester from BLDPharm (China); 2-methoxyphenol, iodomethane, 2-fluorophenol, palladium on carbon and diphenylmethanimine from TCI (Japan); d-luciferine potassium salt from Cayman Chemicals (USA); ATP sodium salt, magnesium chloride, disodium hydrogen phosphate, sodium dihydrogen phosphate, sodium chloride biograde, ethylenediaminetetraacetic acid (EDTA) and Tris-hydrochloride (Tris HCl) from Carl Roth (Germany); Triton-X and 1,4-dithiothreitol (DTT) from Goldbio (USA); Oxone®, sodium nitrite, sodium hydroxide, palladium acetate, 1,1'-bis-(diphenylphosphino)-ferrocen]-dichloro-palladium(II) (Pd(dppf)$Cl_2$), cesium carbonate, trypsin-EDTA solution, penicillin-streptomycin solution, coenzyme A, bio-grade dimethylsulfoxide (DMSO), trifluoroacetic acid (TFA), resazurin and Zeocin™ from Sigma Aldrich/Merck (Germany); potassium trioxalatoferrate(III) trihydrate, hydrochloric acid 37%, acetic anhydride, aberchrome 670, N-methyl-2-pyrrolidinone (NMP), *N*,*N*-Dimethylformamide (DMF), ethanol absolute, acetonitrile HPLC grade from VWR (France); Dulbecco's modified eagle medium (DMEM), Dulbecco's phosphate-buffered saline (DPBS), Lipofectamine 3000, P3000 reagent and pcDNA4TO-vector from Thermo Fisher Scientific (USA); fetal bovine serum (FBS) from Capricorn (USA); 96-well cell culture plate (655086, 655096) from Greiner Bio-One (Austria); NotI-HF and NheI-HF restriction enzymes from New England BioLabs (USA); ultrapure water was obtained with a MicroPure Water purification system (TKA, Germany). Gene fragments and primers were purchased from IDT (USA).
Safety statement: no unexpected or unusually high safety hazards were encountered.

### Mass Spectrometry (MS)

High-resolution electrospray ionization (ESI) mass spectra were acquired with an LTQ-FT Ultra mass spectrometer (Thermo Fischer Scientific).

### Nuclear Magnetic Resonance Spectroscopy (NMR)

NMR spectra of synthesized compounds were recorded at 300 K on a Bruker AV III HD 300 MHz at a frequency of 300 MHz (for $^{1}$H-NMR spectra), 75 MHz ($^{13}$C-NMR spectra), 282 MHz ($^{19}$F-NMR spectra). The $^{1}$H- and $^{13}$C-NMR spectra were referenced to solvent residue peaks. As internal standards, deuterated chloroform ($CDCl_3$) or deuterated dimethyl sulfoxide (DMSO-$d_6$) with TMS were used. Solvent shifts (ppm): ($CDCl_3$, $\delta$) = 7.26 ppm ($^{1}$H) and 77.16 ppm ($^{13}$C), (DMSO-$d_6$, $\delta$) = 2.50 ppm ($^{1}$H), and 39.52 ppm ($^{13}$C). $^{19}$F chemical shift was referenced to $CFCl_3$ externally.[1] NMR spectra of RNA with and without compound were recorded at 298 K on Bruker AV III HD 800 MHz (Cryo TCI $^{1}$H [$^{13}$C, $^{15}$N]) or AV III 800 MHz (Cryo TXO $^{13}$C [$^{1}$H, $^{15}$N]). DSS was used as internal standard to reference spectra.

### High Performance Liquid Chromatography (HPLC)

For isomer ratio quantification, an Agilent 1260 Infinity II HPLC-System (Agilent Technologies, USA) with an XDB column (1.8 µm XDB-C18, 50 × 4.6 mm, Agilent Technologies) with a flow rate of 1.0 mL/min was used. Signal detection was performed at the isosbestic wavelength (304 nm). The eluents were ultrapure water with addition of 0.05% TFA (A) and MeCN with the addition of 0.03% TFA (B). After column stabilization, the gradient used was 5→95% B in 6.75 min, follow by 95% B in 1 min and 95→5% from 7.75 to 10 min.

### Ultraviolet-visible Spectroscopy (UV-vis)

UV-vis spectra were recorded on a Spark 20M multimode microplate reader (Tecan, Switzerland) using a 1400 µL quartz cuvette (Hellma Analytics (104F-QS)) with a pathlength of 1 cm.

The molar extinction coefficients were determined in bio-grade DMSO at corresponding isosbestic wavelengths of each compound. The corresponding samples were prepared by dissolving weighted amount (3x ~5 mg) in 500 µL DMSO with known concentration and added stepwise 2 µL into 1 mL DMSO in cuvette. For each adding step, the UV-vis spectrum was recorded. The lineal regression of the obtained absorbance measurements at corresponding isosbestic point versus the concentrations of the corresponding measurement allowed obtaining the corresponding molar extinction coefficient of the measured compounds with the Lambert-Beer law:

$$A = \epsilon \cdot c \cdot l$$

where *A* = absorbance; *ε* = molar extinction coefficient; *c* = concentration; *l* = path length.

Concentration determinations were performed by measuring absorbance at corresponding isosbestic point, using the molar extinction coefficient at corresponding isosbestic wavelength, and applying the Beer-Lambert law.

**Photoisomerization**

*UV-vis Characterization*

For analytical isomerization studies, Led-emitters (wavelength of maximum emission: 520 nm, luminous intensity: 16000-27000 mcd, viewing angle: 23°, AVAGO HLMP-CM2B-120DD, Broadcom Limited, United States; 365 nm, UV-A, WEEUV00-CS, viewing angle: 25°, Winger Electronics, Germany) were used for irradiation. The corresponding sample was prepared in phosphate buffer (10 mM $NaH_2PO_4$, 150 mM NaCl, pH 7.4) with a final concentration of 50 µM, then it was placed in a 1400 µL quartz cuvette (Hellma Analytics (104F-QS)) with a pathlength of 1 cm. The cuvette containing compound **2-8**, **10-12** was irradiated first at 365 nm at different time intervals: 10 s / 20 s / 30 s / 40 s / 50 s / 60 s / 90 s; for compound **9**, the sample was irradiated at 365 nm at different time intervals: 10 s / 20 s / 30 s / 40 s / 50 s / 60 s / 90 s / 120 s / 150 s / 180 s / 240 s / 300 s / 360 s; back isomerization of corresponding sample was followed by irradiation at 520 nm at the time intervals: 5 s / 10 s / 20 s / 30 s. After each irradiation the absorbance spectrum was recorded at room temperature. The values were measured in duplicates.

*Preparation of Photostationary States (PSS)*

For stability studies and cell-based assays, the stock solutions of compounds were prepared by dissolving freshly in bio-grade DMSO and the concentration was determined followed the procedure described in section 'Ultraviolet-visible Spectroscopy (UV-vis)'. The corresponding sample of compound **2-12** was then diluted to desired concentrations. Diluted samples were irradiated using UV-emitter 365 nm LED (UV-A, WEEUV00-CS, 4 mW, Winger Electronics, Germany) for 1 min for compound **2-8**, **10-12**, and 6 min for compound **9** to obtain the maximal ratio of the *cis* isomer. Samples without irradiation were protected from light, presented in form of *trans,* were used directly.

*HPLC characterization*

The samples of corresponding compounds were prepared in ultrapure water/acetonitrile 1:1 in microcentrifuge tubes (Eppendorf) with a concentration of 10 µM. The fresh prepared sample was irradiated at 365 nm for 1 min for compound **2-8**, **10-12**, and 6 min for compound **9**; or at 520 nm for 30 s for compound **2-12**; or without irradiation. The corresponding sample was then analyzed via HPLC at the corresponding isosbestic point and the *cis-trans* ratio was calculated using OpenLab ChemStation software (Agilent Technologies) by calculating the area of the integrated peak.

**Photoisomerization Quantum Yield**

Photon fluxes (*I*) of 365 nm UV light LED and 520 nm green light LED were determined by using chemical actinometer trioxalatoferrate and aberchrome 670 respectively and followed the reported procedures.[2,3]
A trioxalatoferrate solution (6 mm in 0.2 m aqueous $H_2SO_4$ solution, 150 µL, $V_1$) was irradiated at 365 nm at different time intervals between 100 and 300 s. 100 µL ($V_2$) of the each irradiated trioxalatoferrate solution was mixed with 200 µL buffer (1.2 M NaOAc, 0.72 m $H_2SO_4$ in distilled water) and 400 µL phenanthroline solution (6 mm in distilled water), and finally diluted to 2.5 mL ($V_3$). The resulted mixture was left to react for 1 h in the dark. Absorbance of each mixture was recorded at 510 nm. The lineal regression of the obtained absorbance measurements versus the irradiation time was plotted. Photon flux *I* was obtained applying following equation: [2]

$$I = slope \cdot \frac{V_1 \cdot V_3}{V_2 \cdot \varepsilon_{510nm} \cdot l \cdot \phi}$$

Where $V$ = volume of corresponding solutions; $\varepsilon_{510nm}$ = molar extinction coefficient of tris-phenanthroline-iron(II) complex (11 100 $m^{-1}cm^{-1}$)[2]; $l$ = path length; $\phi$ = photochemical quantum yield of trioxalatoferrate at 365 nm ($\phi$ = 1.21)[2].
The aberchrome 670 stock solution was prepared in toluene with a concentration of 150 µM and irradiated at 365 nm for 10 min. 4 samples, each containing 200 µL of stock solution, were irradiated at 520 nm green light at different time intervals: 20 s / 40 s / 60 s / 80 s, the corresponding absorbance of each irradiated sample was recorded at 520 nm. The lineal regression of the obtained absorbance measurements versus the irradiation time was plotted. The obtained slope was used to calculate the photon flux.
The photochemical quantum yield $\Phi$ of aberchrome 670 at irradiated wavelength was calculated by applying following equation: [2]

$$\phi = 0.4326 - (3.285 \cdot \lambda - 16.4 \cdot T) \cdot 10^{-4}$$

where $\lambda$ = the irradiation wavelength; $T$ = the measurement temperature (room temperature).
The photon flux $I$ was calculated by applying following equation: [2]

$$I = -\frac{slope \cdot V}{\phi \cdot (1 - 10^{-A_t})}$$

where $A_t$ = absorbance of the sample at irradiation time; $V$ = volume of the sample; $\phi$ = photochemical quantum yield of aberchrome 670.
The photoisomerization quantum yields of compounds **2-12** were calculated by using following equations:[3]

$$f = 1 - 10^{-A_{\lambda_{irr}}}$$

$$f_{ave} = \frac{f_0 + f_1}{2}$$

Where $f$ = fraction of light absorbed by the sample, before ($f_0$) and after irradiation ($f_1$); $A_{\lambda irr}$ = absorbance of the sample at irradiation wavelength; $f_{ave}$ = average of $f_0$ and $f_1$.[3]

$$\Delta[trans] = -\frac{\phi_{trans} \cdot I}{V} \cdot f_{ave} \cdot \Delta t_{irr}$$

$$\Delta[cis] = -\frac{\phi_{cis} \cdot I}{V} \cdot f_{ave} \cdot \Delta t_{irr}$$

Where *Δ[trans/cis]* = concentration difference before and after irradiation of corresponding isomer; $\phi_{trans\,/\,cis}$ = photochemical quantum yield; $I$ = photon flux; $V$ = volume of the sample; $f$ = fraction of light absorbed by the sample; $\Delta t_{irr}$ = time interval of irradiation.

## Cell Culture

All sterile working steps were performed under an MSC-Advantage clean bench (Thermo Scientific). HEK293T-SMN2-Luc, cells were grown in flat-bottomed culture flasks (T75, Sarstedt, Germany) in a humidified cell-culture incubator (Galaxy CO-170 S incubator, New Brunswick Scientific, USA) at 37 °C under $CO_2$ (g) (5% v/v). Growth medium was DMEM nutrient medium supplemented with fetal bovine serum (FBS) (10% v/v), penicillin and streptomycin (10 000 units/mL and 10 mg/mL, 1 % v/v). DMEM and DPBS were stored at 4 °C, additives and trypsin at -20 °C, solutions were tempered to 37 °C prior usage, utilizing a water bath. Cells were grown to 90% confluency and passaged every 2 to 4 days using a trypsin-EDTA solution till passage 10. Cells were counted using a Neubauer improved cell counter (Laboroptik Ltd, Germany) for the determination of seeding densities. Additionally, 180 µg/*mL of Zeocin™ was used as* resistance marker for the plasmid carrying cells selection.

### *Minigene Constructs*

The pCI-SMN2-Luc minigene construct was prepared as previously described.[4] The vector was digested with Nhe1-HF and Not1-HF restriction enzymes (New England Biolabs) and the purified SMN2-Luc fragment was subcloned into the NHE1/NOT1 restriction site of a multiple cloning site modified derivative of the original pcDNA4TO-vector (Thermo fisher).
500 000 HEK293T cells were transfected with 5 µg pcDNA4TO SMN2-Luc or CMV-Hygro SMN2-NlucP vector with Lipofectamine 3000 (Thermo fisher) according to manufacturer instructions (7.5 µL Lipofectamine 3000 + 10 µL P3000 reagent). After 48 h hours cells were selected over a course of several weeks using resistance marker Zeocin$^{TM}$ in a concentration of 400 µg /mL.

*HEK293T-SMN2-Luc Cell-Based Reporter Gene Assays*

- **Seeding:** 100 µL of a HEK293T-SMN2-Luc suspension containing 20 000 cells in DMEM were seeded per well into black F-bottom 96-well cell culture microplates (655086, Greiner Bio-One) for viability assay, or into white F-bottom 96-well cell culture microplates (655083, Greiner Bio-One) for *firefly* luciferase assay. DMEM media was supplemented with FBS (5 % v/v) and penicillin-streptomycin (1 % v/v). The plates were incubated a completely humidified atmosphere of 5 % $CO_2$ for 18 h.
- **Preparation of compounds:** Each sample of corresponding compound **2-12** was dissolved in bio-grade DMSO and the concentration was determined as described in section 'Ultraviolet-visible Spectroscopy (UV-vis)', the stock solution was protected from light. The stock solution was further diluted with sterile ultrapure water and bio-grade DMSO (final DMSO content: 2.75%). Stock solutions of each concentration (11-fold higher concentrated as desired final one) were divided into two, and transferred separately into microcentrifuge tubes (Eppendorf, Germany). Isomerization was achieved as described section 'Preparation of Photostationary States (PSS)'.
- **Addition of compounds:** Under red light (Par-38 FL, red halogen lamp, 230 V, E27, 80 W, Conrad) in the dark, 10 µL of a stock solution of different concentrations of corresponding compound **2-12** (ranging from 154 to 5.5 µM) were added to the cells, and thoroughly mixed. For solvent controls, 10 µL of 2.75% DMSO in sterile ultrapure water was added. For untreated controls, 10 µL of DMEM supplemented with FBS (5 % v/v) and penicillin-streptomycin (1 % v/v) was added. The final concentrations of each isomer in the well were 14, 12, 10, 8, 6, 4, 2, 1, 0.5 µM with a final volume of 110 µL and 0.25% of DMSO. The plate was protected from light and incubated in a humidified cell-culture incubator at 37 °C in a completely humidified atmosphere of 5 % $CO_2$ for 6 h.
- **Data evaluation:** Cells were assessed by following assays:

*Resazurin fluorescence-based cell viability assay:*[5]

After 6 h incubation, 20 µL of a 1.63 mM solution of resazurin in DPBS were added to each well, and thoroughly mixed. The plate was further shaken for 30 s using the SpectraMax M5 plate reader (Molecular Devices, USA) using the shaking function. In the same device, the fluorescence was next measured with the following settings: fluorescence top read; excitation wavelength: 560 nm; emission wavelength: 590 nm; cut-off filter: 590 nm and 6 readings per well in 20 min intervals until 100 min. The slope of each individual well was calculated, and the percentage of cell survival in the untreated control was assumed as 100%. Measurements with technical triplicates were repeated three times independently, i.e., from different stock solutions and different passages of cells in triplicate. The activity of each condition was calculated with following equation:

$$Relavtive\ viability = \frac{Slop_{sample} - Slop_{media\ background}}{Slop_{untreated\ control} - Slop_{media\ background}} \times 100\%$$

where *Slop* indicates the lineal regression of obtained fluorescence intensity versus the time of corresponding measurement.

*Luciferase assay:*[6]

After the 6 h of incubation, the plate was left for additional 15 min at room temperature protected from light. 3-fold concentrated *firefly* assay buffer was prepared freshly (15 mM DTT, 0.6 mM coenzyme A, 0.45 mM ATP, 15 mM d-luciferine potassium salt, 0.1082 M Tris HCl, 0.0419 M Tris, 75 mM NaCl, 3 mM $MgCl_2$, 0.25% Triton-X in ultrapure water). 50 µL of 3x *firefly* assay buffer were added to each well, and the plate was incubated for 5 min at room temperature mixing in a shaker (Edmund Bühler, Germany). The luminescence was next measured using the Spark 20M multimode microplate reader (Tecan, Switzerland) with the following settings: luminescence top read and integration time: 1000 ms. The luminescence intensity of each individual well was measured, and the intensity of untreated control was normalized as 1. All values were obtained in triplicates per each concentration sample or control. Measurements were repeated three times independently, i.e., from different stock solutions and different passages of cells. The activity of each condition was calculated with following equation:

$$Relavtive\ efficiency = \frac{Lum_{sample} - Lum_{media\ background}}{Lum_{untreated\ control} - Lum_{media\ background}}$$

where *Lum* indicates the luminescence intensity.

**Modelling**

Prediction of the dsRNA:ligands interactions were performed according to the our previously developed protocol encompassing RNA-ligand flexible docking and molecular dynamics (MD) simulatio. After deduction of ligand's protonation states with Marvin 21.3[7], optimization of 3D structures on HF 6-31G* level of theory with Gaussian09[8] and generation of up to 200 conformers using Conformer Generator tool implemented in CSD Mercury 4.3.1[9], each ligand was docked into each of 20 structures of ensemble of dsRNA PDB ID:6HMO using RLDOCK software[10].The predicted binding poses were refined through MD simulations using Gromacs 2020.4[11]. Studied systems were solvated with TIP3P water model in octahedral periodic box and charges were neutralized using 0.15M NaCl. Ligands and dsRNA were parametrized using Amber14sb_OL15 force field with included pseudouridine parameters and GAFF2 force field for ligands with addition of previously published bonded parameter corrections for azobenzene[12,13]. Atomic charges for ligands were fitted using restrained electrostatic potential (RESP/HF-6-31G*). Upon minimization (5000 steps of steepest descent), equilibration of systems was performed firstly in NVT ensemble (298.16 K, V-rescale thermostat) and then in NPT ensemble (1.0 bar, Parrinello-Rahman barostat). Position restraints were gradually removed (starting from 1000 kJ/mol nm$^{-2}$) during 1 ns of NPT equilibration. Particle Mesh Ewald (PME) for long-range electrostatics and non-bonded cutoff of 10 Å were used. LINCS algorithm 72 was used to convert all bonds with H-atoms into constraints[14]. Production runs were performed in the NPT ensemble for 100 ns for each of studied systems. Analysis of obtained trajectories was performed using built-in Gromacs tools (gmx anaeig and gmx covar). Visual inspection of interactions were performed after trajectory clustering procedure using the gromos method implemented into the Gromacs software. Initial coordinates for the *apo* system were obtained from PDB ID:6HMI.

## Synthesis

Baeyer-Mills reaction was followed the procedure of Yu *et. al*,[15] Suzuki-Miyaura coupling reaction was followed the procedure of Engers *et. al*,[16] synthesis of 4-fluoro-2-methyl-1,3-benzoxazol-6-amine was followed the procedures of Ratni *et. al.*[17]

### Synthesis of 6-((4-bromo-2-fluorophenyl)diazenyl)-2-methylbenzo[d]oxazole (15)

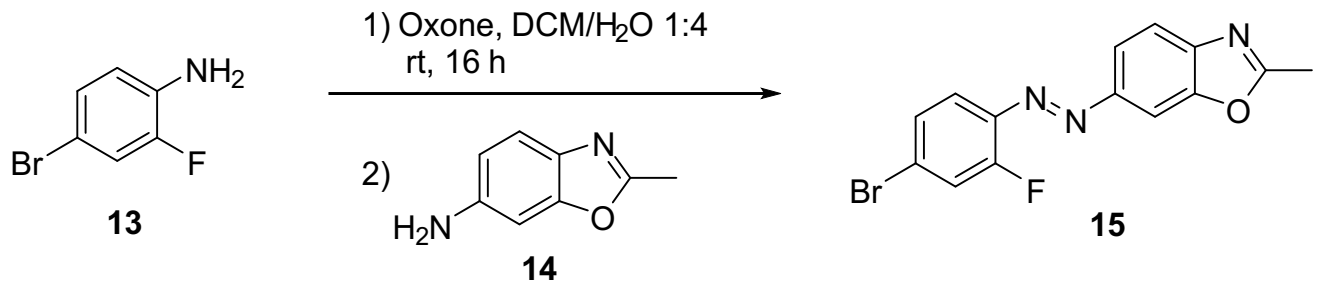


AcOH, rt, 48 h

87%

4-Bromo-2-fluoroaniline **13** (183 mg, 675 µmol, 2.00 eq) was dissolved in 5 mL DCM, Oxone® (415 mg, 1.35 mmol, 4.00 eq) was dissolved in 20 mL distilled water and added to the solution. The resulting mixture was stirred for 16 h at room temperature. The reaction mixture was then diluted with 100 mL DCM and washed with 100 mL 1 M HCl, distilled water and brine. The organic layer was dried over $MgSO_4$ and the solvent was removed under reduced pressure. The resulting brown solid and 2-methyl-1,3-benzoxazol-6-amine **14** (50 mg, 337 µmol, 1.00 eq) were suspended in 20 mL acetic acid. The reaction mixture was stirred for 48 h at room temperature. The reaction was then diluted with 200 mL distilled water and extracted with 200 mL EtOAc. The organic layer was washed with 200 mL distilled water and brine, dried over $MgSO_4$ and solvent was removed under reduced pressure. The crude product was purified by flash column chromatography (DCM) to yield the desired compound 6-((4-bromo-2-fluorophenyl)diazenyl)-2-methylbenzo[*d*]oxazole (**15**, 98 mg, 294 µmol, 87%) as an orange solid.

**TLC:** $R_f$ = 0.29 (DCM).

***trans*-isomer** (*cis*-isomer signals were labeled with * on NMR spectrum).

**$^1$H-NMR (300 MHz, $CDCl_3$, *δ*):** 8.05-8.00 (m, 2H, $H_{arom.}$, $H_{arom.}$), 7.77-7.66 (m, 2H, $H_{arom.}$, $H_{arom.}$), 7.48 (dd, $^3J$ = 9.9 Hz, $^4J$ = 2.0 Hz, 1H, $H_{arom}$), 7.38 (ddd, $^3J$ = 8.7 Hz, $^4J$ = 2.1 Hz, $^4J$ = 1.1 Hz, 1H, $H_{arom}$), 2.70 (s, 3H, C*H*$_3$) ppm.

**$^{13}$C-NMR (75 MHz, $CDCl_3$, *δ*):** 166.9 (N=*C*-O), 159.9 (d, $^1J_{FC}$ = 262.5 Hz, *C*F), 151.6 ($C_{arom.}$), 150.4 ($C_{arom.}$), 144.7 ($C_{arom.}$), 139.7 (d, $^2J_{FC}$ = 7.2 Hz, $C_{arom.}$), 128.0 (d, $^3J_{FC}$ = 3.8 Hz, $C_{arom.}$H), 125.7 (d, $^3J_{FC}$ = 9.1 Hz), 122.3 ($C_{arom.}$H), 120.9 (d, $^2J_{FC}$ = 23.0 Hz, $C_{arom.}$H), 119.6 ($C_{arom.}$H), 119.0 ($C_{arom.}$H), 104.0 ($C_{arom.}$H), 15.0 (*C*H$_3$) ppm.

**$^{19}$F-NMR (282 MHz, $CDCl_3$, *δ*)**: -121.8 (C*F*) ppm.

**HRMS-ESI (m/z):** $[M+H]^+$ calcd for $C_{14}H_{10}BrFN_3O$, 335.9966; found, 335.9977.

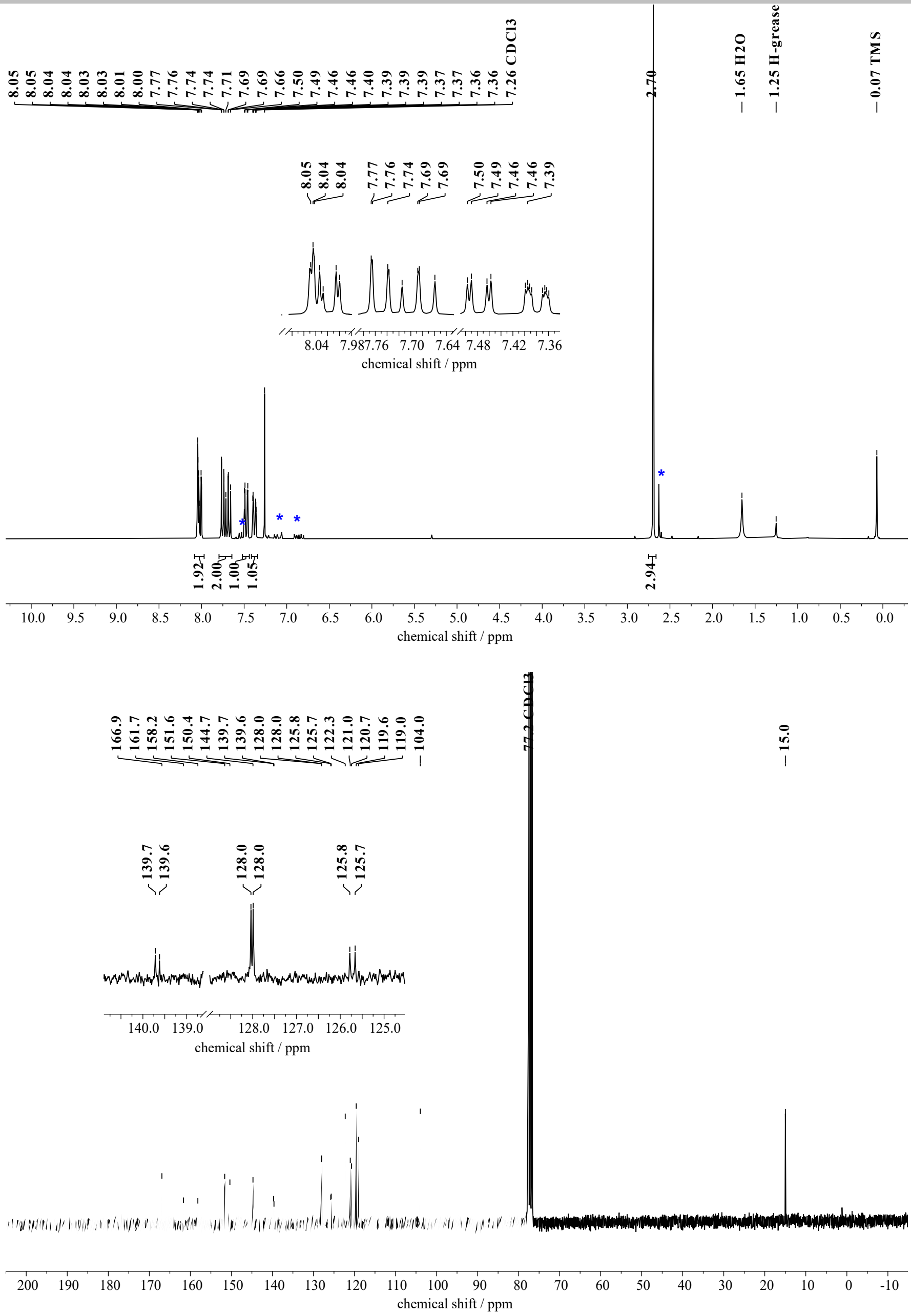

**Figure S1.** 1H (300 MHz) and $^{13}C$ (75 MHz) NMR spectra of **15** in $CDCl_3$.

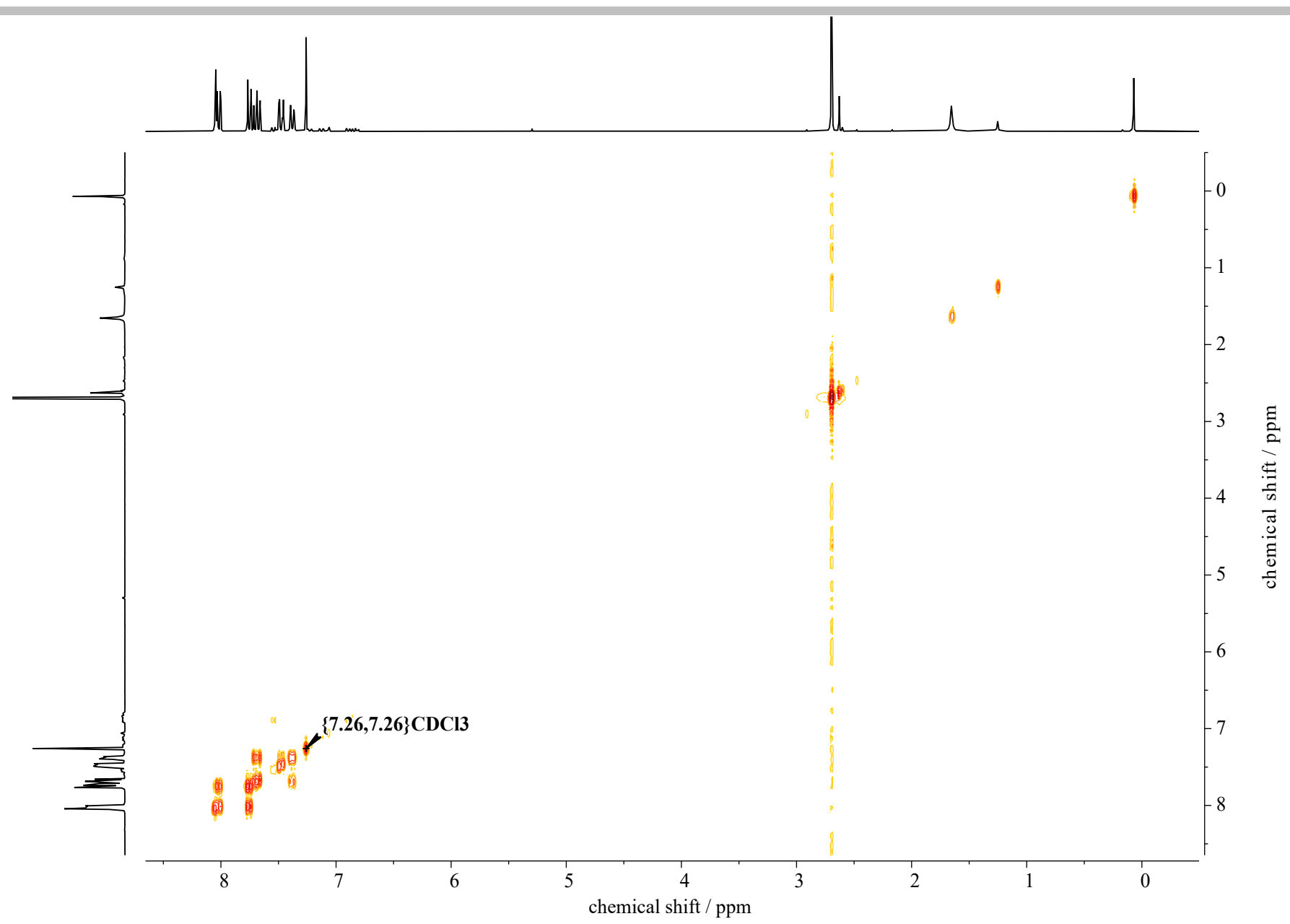


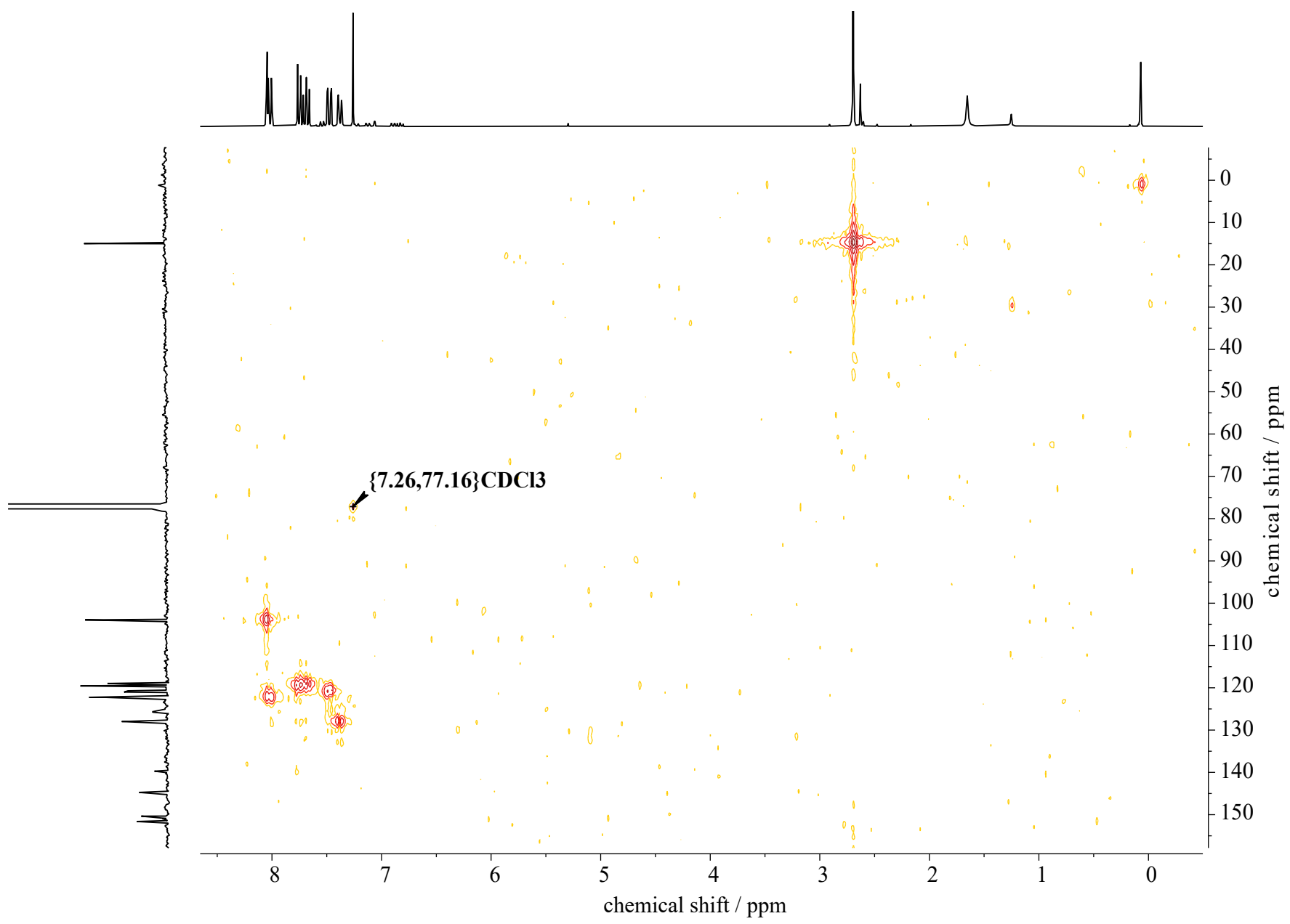


**Figure S2**. COSY and HMQC NMR spectra of **15** in $CDCl_3$.

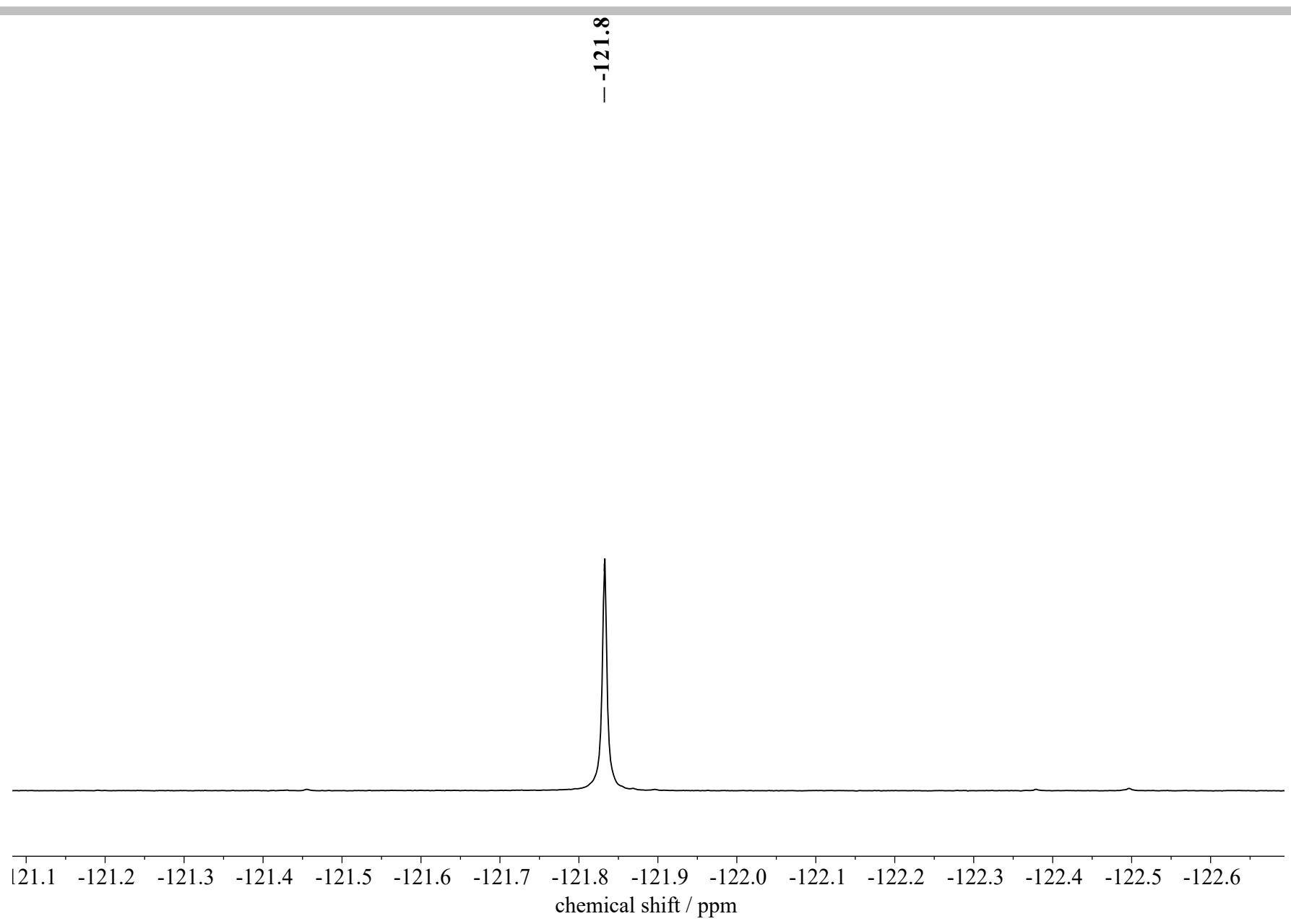


**Figure S3**. $^{19}F$ (282 MHz) NMR spectrum of **15** in $CDCl_3$.

**Synthesis of tert-butyl-4-(3-fluoro-4-((2-methylbenzo[d]oxazol-6-yl)diazenyl)phenyl)-3,6-dihydropyridine-1(2H)-carboxylate (17)**

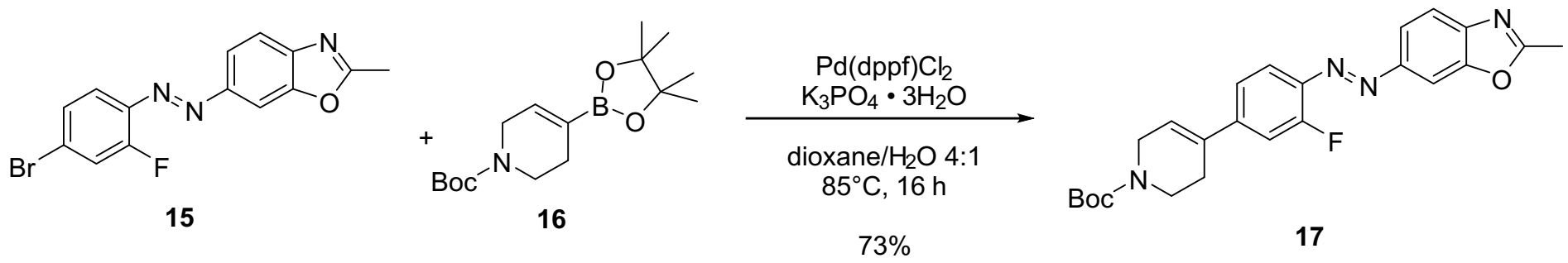


6-((4-Bromo-2-fluorophenyl)diazenyl)-2-methylbenzo[*d*]oxazole (**15**, 50 mg, 150 µmol, 1.20 eq), *N*-*tert*-butoxycarbony-1,2,3,6-tetrahydropyridine-4-boronic acid pinacol ester (**16**, 39 mg, 125 µmol, 1.00 eq), Pd(dppf)$Cl_2$ (9 mg, 12.5 µmol, 0.10 eq) and $K_3PO_4 \cdot 3H_2O$ (100 mg, 374 µmol, 3.00 eq) were dissolved in mixture of dioxane/distilled water (10 mL, 4:1, v%). The reaction was stirred under $N_2$ atmosphere at 85 °C for 16 h. The reaction was then diluted with 100 mL distilled water and extracted with 100 mL EtOAc. The organic layer was washed with 100 mL distilled water and brine, dried over $MgSO_4$ and solvent was removed under reduced pressure. The crude product was purified by flash column chromatography (DCM/EtOAc, 10:1, v%) to yield the desired compound *tert*-butyl-4-(3-fluoro-4-((2-methylbenzo[*d*]oxazol-6-yl)diazenyl)phenyl)-3,6-dihydropyridine-1(*2H*)-carboxylate (**17**, 40 mg, 91.3 µmol, 73%) as an orange solid.

**TLC:** $R_f$ = 0.45 (DCM/EtOAc, 10:1, v%).

**$^1$H-NMR (300 MHz, DMSO-$d_6$, δ):** 8.12 (d, $^4J$ = 1.7 Hz, 1H, $H_{arom.}$), 7.96 (dd, $^3J$ = 8.5 Hz, $^4J$ = 1.8 Hz, 1H, $H_{arom.}$), 7.84 (d, $^3J$ = 8.5 Hz, 1H, $H_{arom.}$), 7.74 (t, $^3J$ = 8.3 Hz, 1H, $H_{arom.}$), 7.57 (dd, $^3J$ = 12.7 Hz, $^4J$ = 1.9 Hz, 1H, $H_{arom.}$), 7.45 (dd, $^3J$ = 8.5 Hz, $^4J$ = 1.9 Hz, 1H, $H_{arom.}$), 6.45 (s, 1H, C*H*=C), 4.06-4.04 (m, 2H, C$H_2$), 3.56 (t, $^3J$ = 5.6 Hz, 2H, C$H_2$), 2.68 (s, 3H, C$H_3$), 2.52-2.50 (m, 2H, C$H_2$), 1.43 (s, 9H, 3xC$H_3$) ppm.

**$^{13}$C-NMR (75 MHz, DMSO-$d_6$, δ):** 167.0 (N=*C*-O), 159.2 (d, $^1J_{FC}$ = 256.1 Hz, *C*F), 153.8 ($C_{arom.}$), 150.9 ($C_{arom.}$), 149.6 ($C_{arom.}$), 145.1 (d, $^3J_{FC}$ = 9.7 Hz, $C_{arom.}$), 144.1 ($C_{arom.}$), 138.3 (d, $^3J_{FC}$ = 7.7 Hz, $C_{arom.}$), 132.9 ($C_{q.}$), 124.6 (*C*H=C), 121.1 (d, $^2J_{FC}$ = 13.6 Hz, $C_{arom.}$H), 120.5 ($C_{arom.}$H), 119.4 ($C_{arom.}$H), 117.4 ($C_{arom.}$H), 113.3 ($C_{arom.}$H), 113.0 ($C_{arom.}$), 104.2 ($C_{arom.}$H), 78.9 ($C_{q.}$), 28.1 (3C, 3x$CH_3$), 26.2 ($CH_2$), 14.4 ($CH_3$) ppm.

**$^{19}$F-NMR (282 MHz, DMSO-$d_6$, δ)**: -124.1 (C*F*) ppm.

**HRMS-ESI (m/z):** $[M+H]^+$ calcd for $C_{24}H_{26}FN_4O_3$, 437.1983; found, 437.1984.

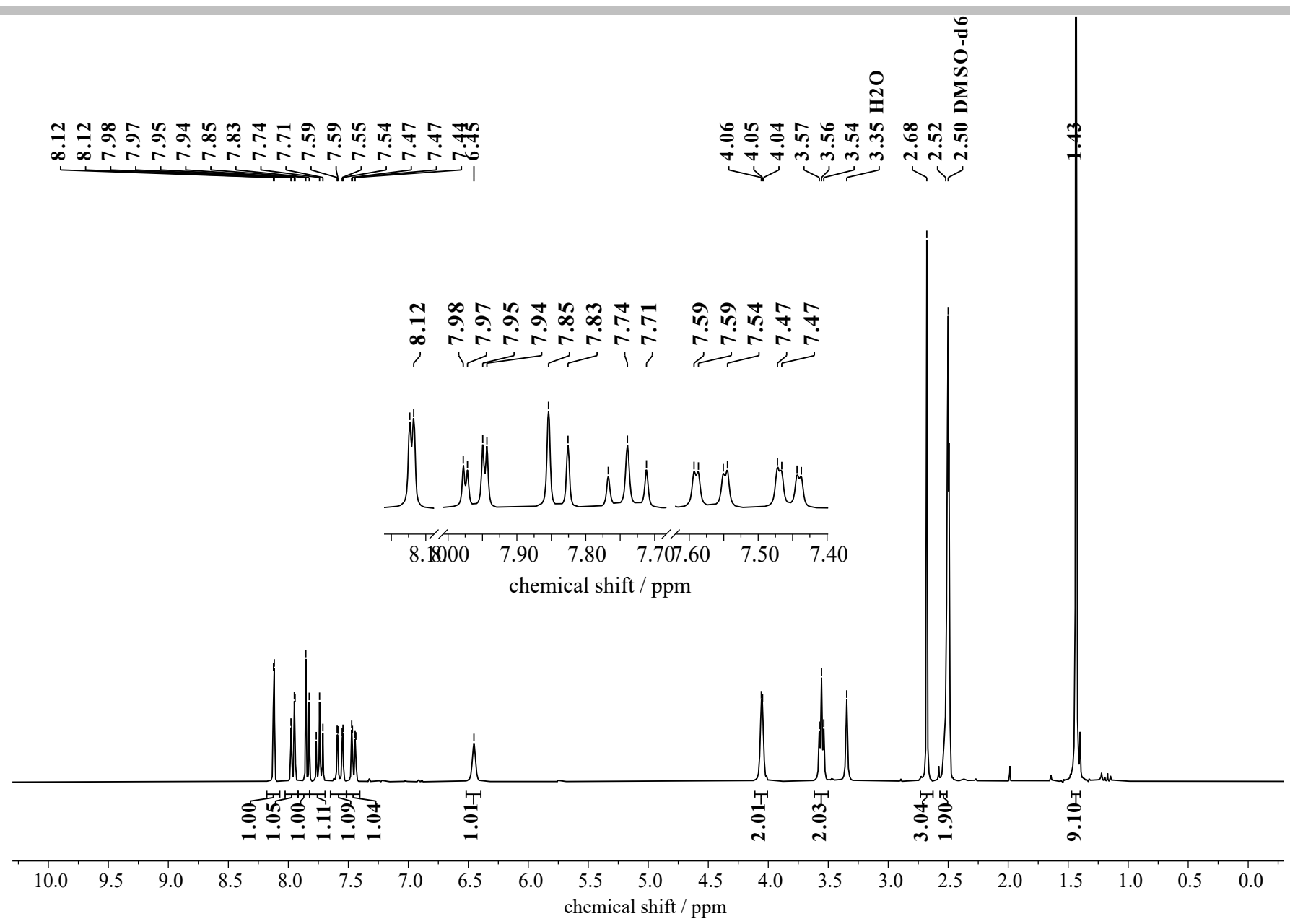


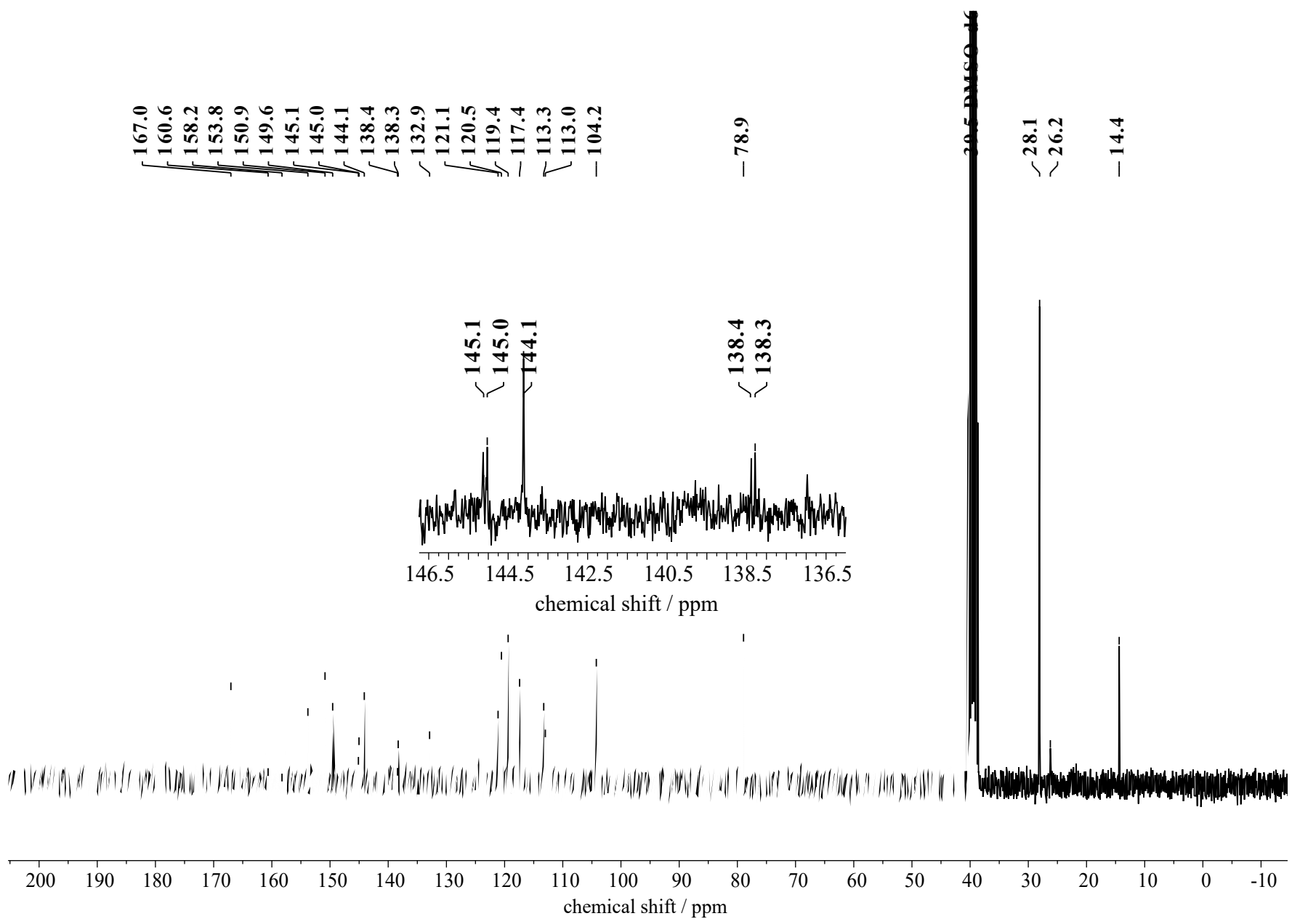


**Figure S4**. $^{1}H$ (300 MHz) and $^{13}C$ (75 MHz) NMR spectra of **17** in DMSO-$d_6$.

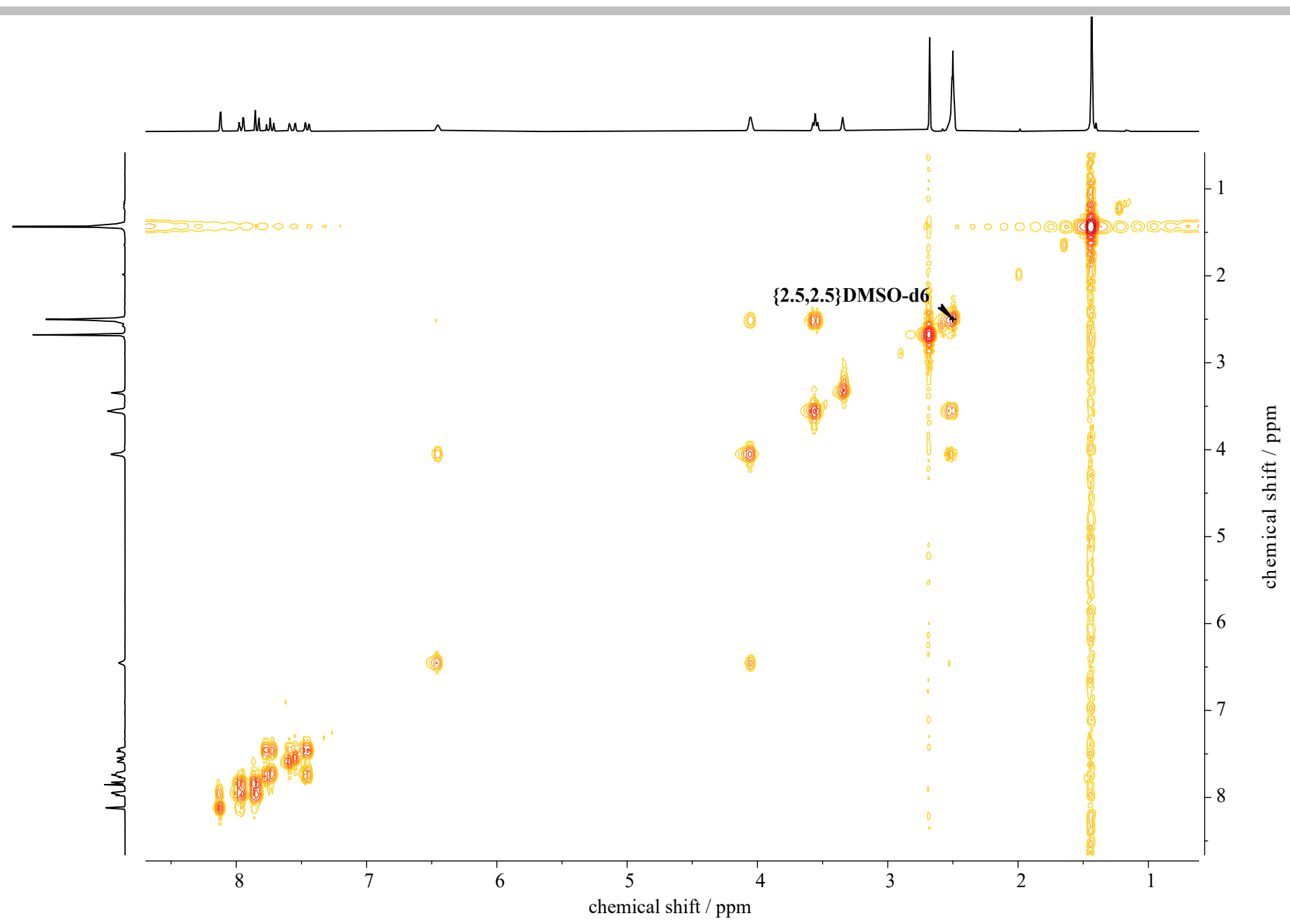


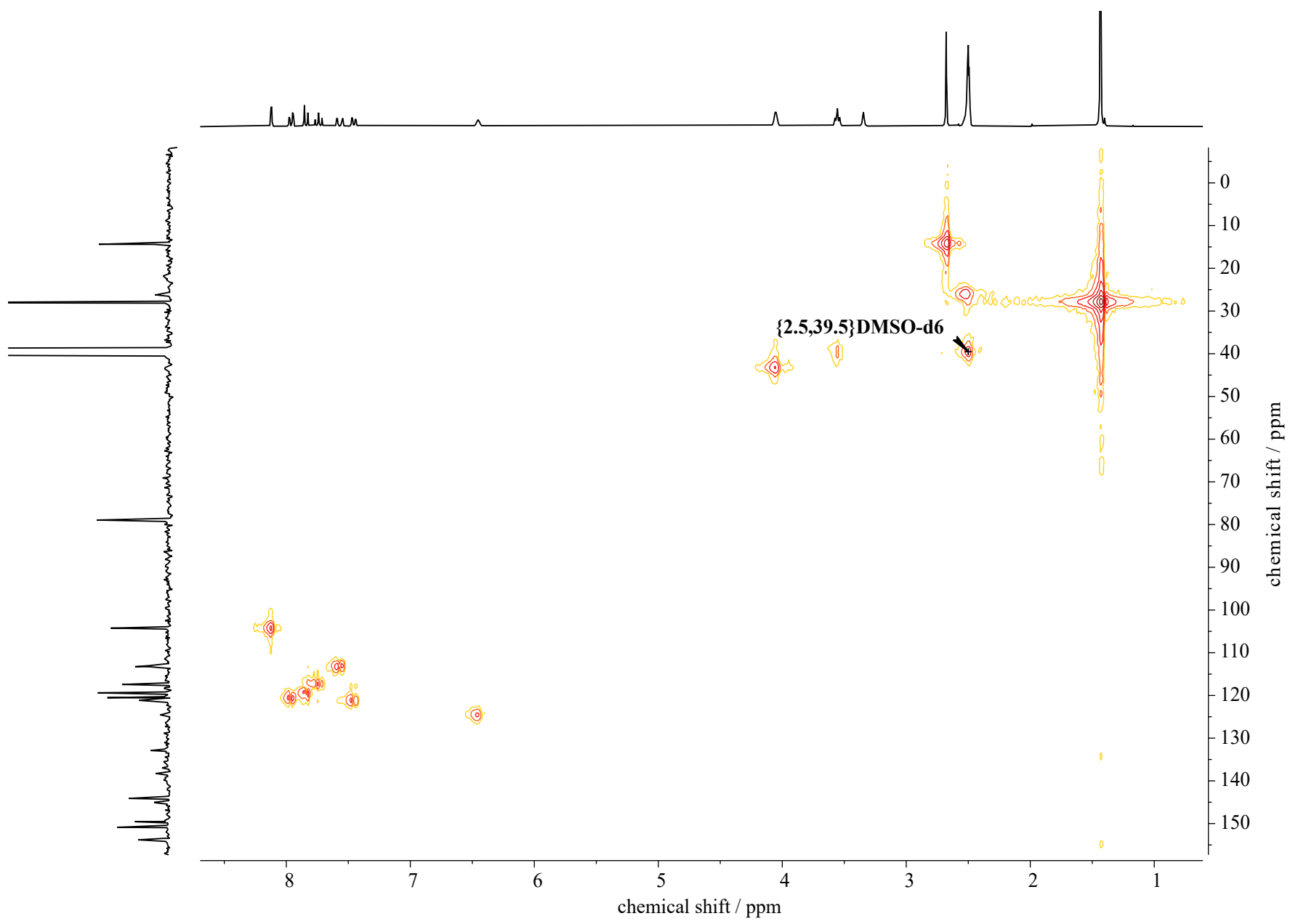


**Figure S5**. COSY and HMQC NMR spectra of **17** in DMSO-*d*$_6$.

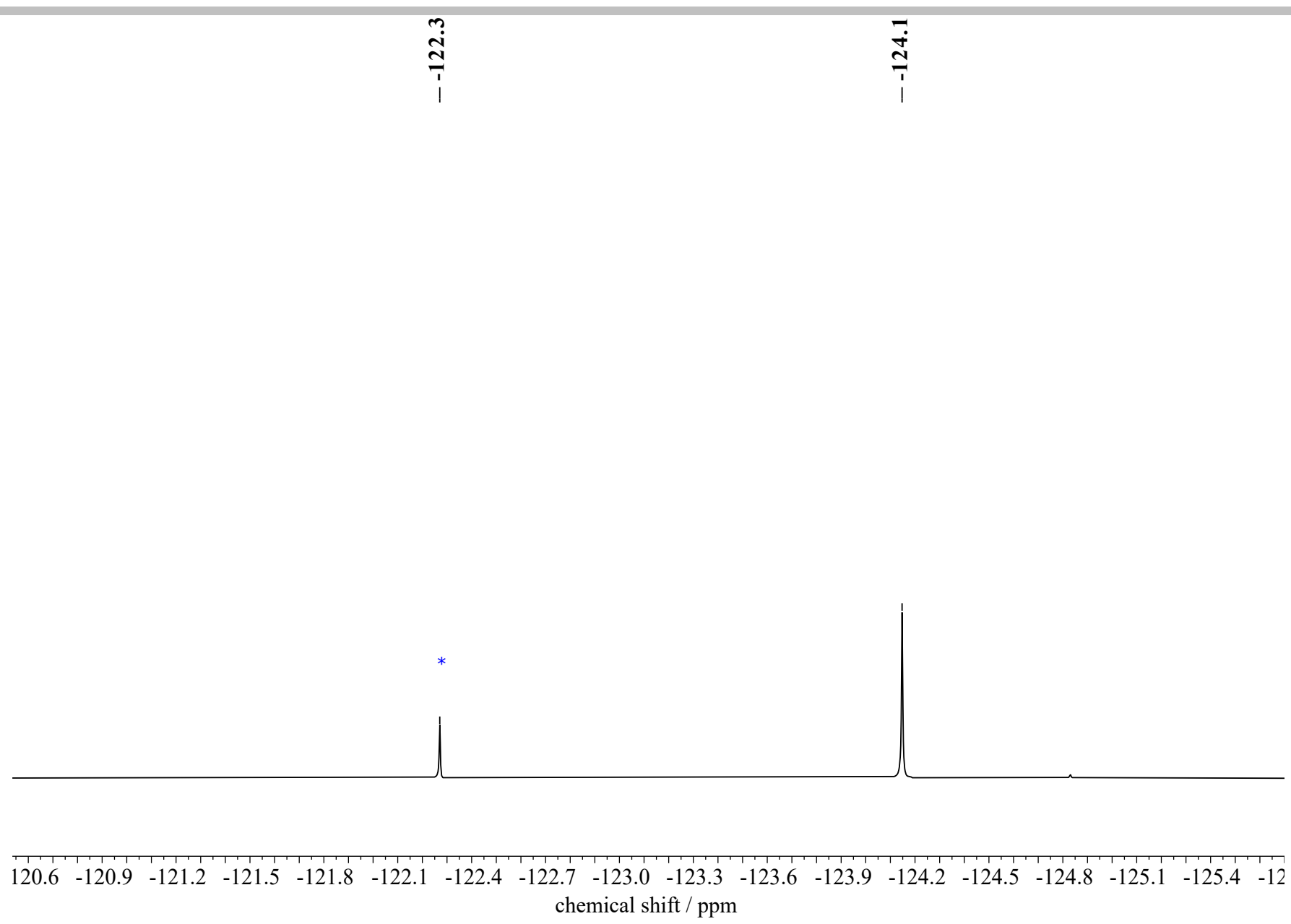


**Figure S6**. $^{19}$F (282 MHz) NMR spectra of **17** in DMSO-$d_6$.

**Synthesis of 4-(3-fluoro-4-((2-methylbenzo[d]oxazol-6-yl)diazenyl)phenyl)-1,2,3,6-tetrahydropyridin (3)**

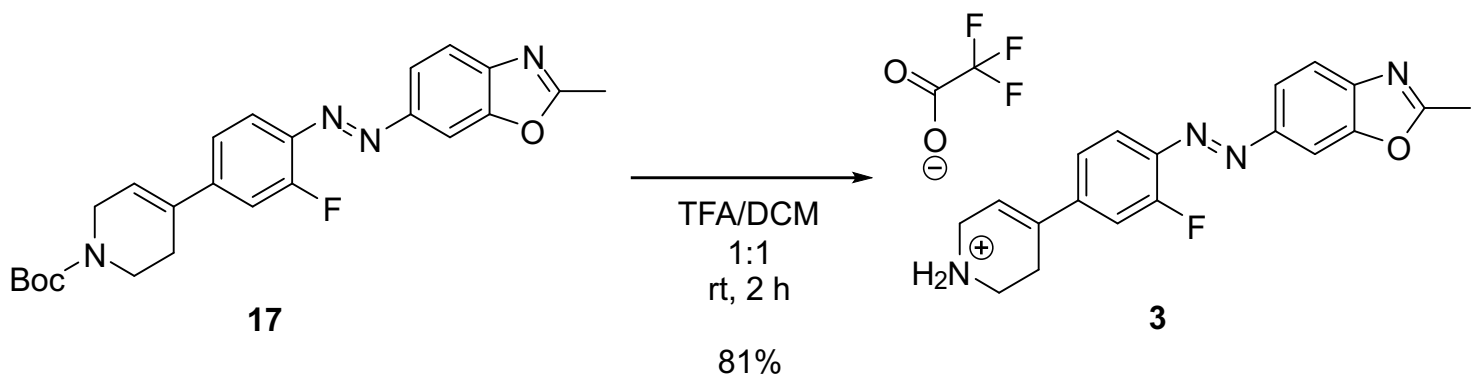


*Tert*-butyl-4-(3-fluoro-4-((2-methylbenzo[*d*]oxazol-6-yl)diazenyl)phenyl)-3,6-dihydropyridine-1(*2H*)-carboxylate (**17**, 30 mg, 68.7 µmol, 1.00 eq) was dissolved in mixture of TFA/DCM (10 mL, 1:1, v%). The reaction was stirred at room temperature for 2 h. The reaction was then diluted with 100 mL distilled water and extracted with 100 mL EtOAc. The organic layer was washed with 100 mL distilled water and brine, dried over $MgSO_4$ and solvent was removed under reduced pressure. The crude product was purified by flash column chromatography (EtOAc/MeOH, 5:1, v%) to yield the desired compound 4-(3-fluoro-4-((2-methylbenzo[*d*]oxazol-6-yl)diazenyl)phenyl)-1,2,3,6-tetrahydropyridin (**3**, 25 mg, 55.6 µmol, 81%) as an orange solid in form of a TFA salt.

**TLC:** $R_f$ = 0.21 (EtOAc/MeOH, 5:1, v%).

***trans*-isomer** (*cis*-isomer signals were labeled with * on NMR spectrum).

**$^{1}$H-NMR (300 MHz, DMSO-$d_6$, δ):** 9.03 (br, 2H, N$H_2$), 8.14 (d, $^4J$ = 1.8 Hz, 1H, $H_{arom.}$), 7.98 (dd, $^3J$ = 8.4 Hz, $^4J$ = 1.8 Hz, 1H, $H_{arom.}$), 7.85 (d, $^3J$ = 8.5 Hz, 1H, $H_{arom.}$), 7.78 (t, $^3J$ = 8.3 Hz, 1H, $H_{arom.}$), 7.66 (dd, $^3J$ = 12.6 Hz, $^4J$ = 1.9 Hz, 1H, $H_{arom.}$), 7.52 (dd, $^3J$ = 8.4 Hz, $^4J$ = 1.7 Hz, 1H, $H_{arom.}$), 6.49 (s, 1H, C*H*=C), 3.84 (s, 2H, C$H_2$), 3.37 (t, $^3J$ = 6.1 Hz, 2H, C$H_2$), 2.74 (s, 2H, C$H_2$), 2.68 (s, 3H, C$H_3$) ppm.

**$^{13}$C-NMR (75 MHz, DMSO-$d_6$, δ):** 167.2 (N=*C*-O), 159.5 (d, $^1J_{FC}$ = 254.5 Hz, *C*F), 150.9 ($C_{arom.}$), 149.5 ($C_{arom.}$), 144.3 ($C_{arom.}$), 143.6 (d, $^2J_{FC}$ = 8.4 Hz, $C_{arom.}$), 138.6 ($C_{q.}$), 132.7 (d, $^3J_{FC}$ = 2.4 Hz, $C_{arom.}$), 121.3 (d, $^3J_{FC}$ = 2.9 Hz, $C_{arom.}$H), 120.6 ($C_{arom.}$), 120.2 ($C_{arom.}$H), 119.4 ($C_{arom.}$H), 117.7 ($C_{arom.}$H), 113.5 (d, $^2J_{FC}$ = 20.9 Hz, $C_{arom.}$H), 104.3 ($C_{arom.}$H), 41.7 ($CH_2$), 22.9($CH_2$), 14.4 ($CH_3$) ppm.

**$^{19}$F-NMR (282 MHz, DMSO-$d_6$, δ):** -123.9 (C*F*) ppm.

**HRMS-ESI (m/z):** $[M+H]^+$ calcd for $C_{19}H_{18}FN_4O$, 337.1468; found, 337.1468.

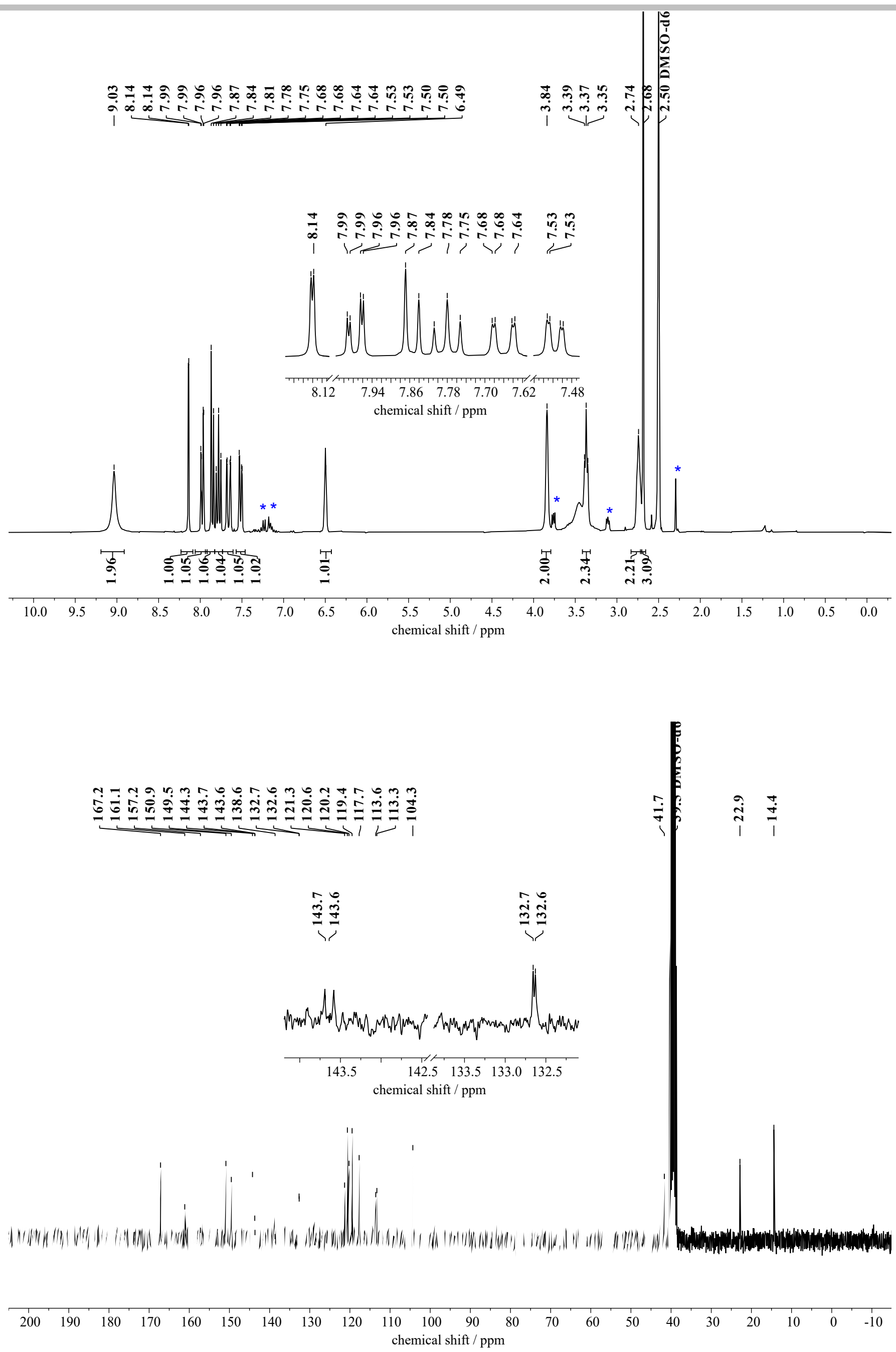


**Figure S7**. $^{1}H$ (300 MHz) and $^{13}C$ (75 MHz) NMR spectra of **3** in DMSO-$d_6$.

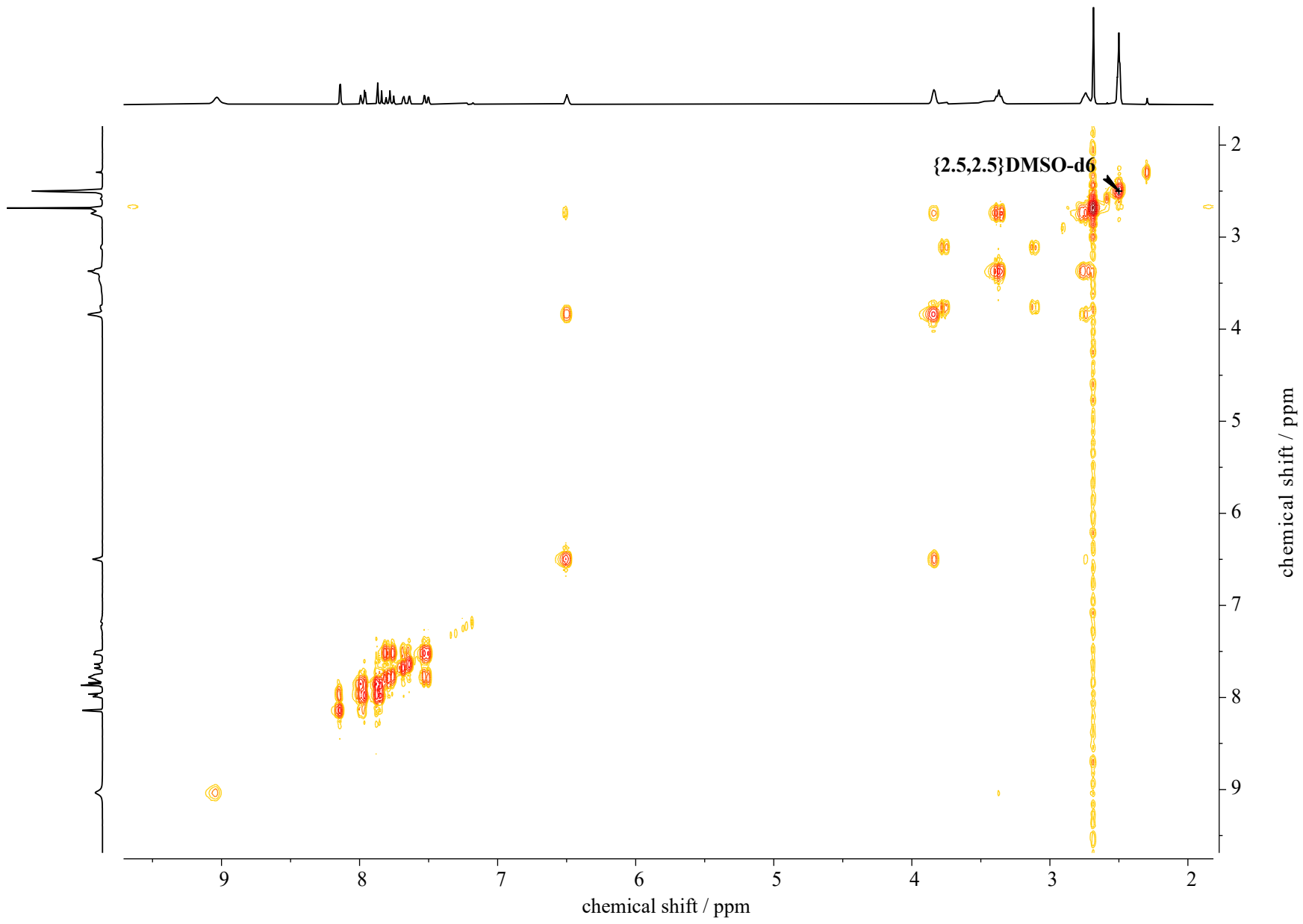


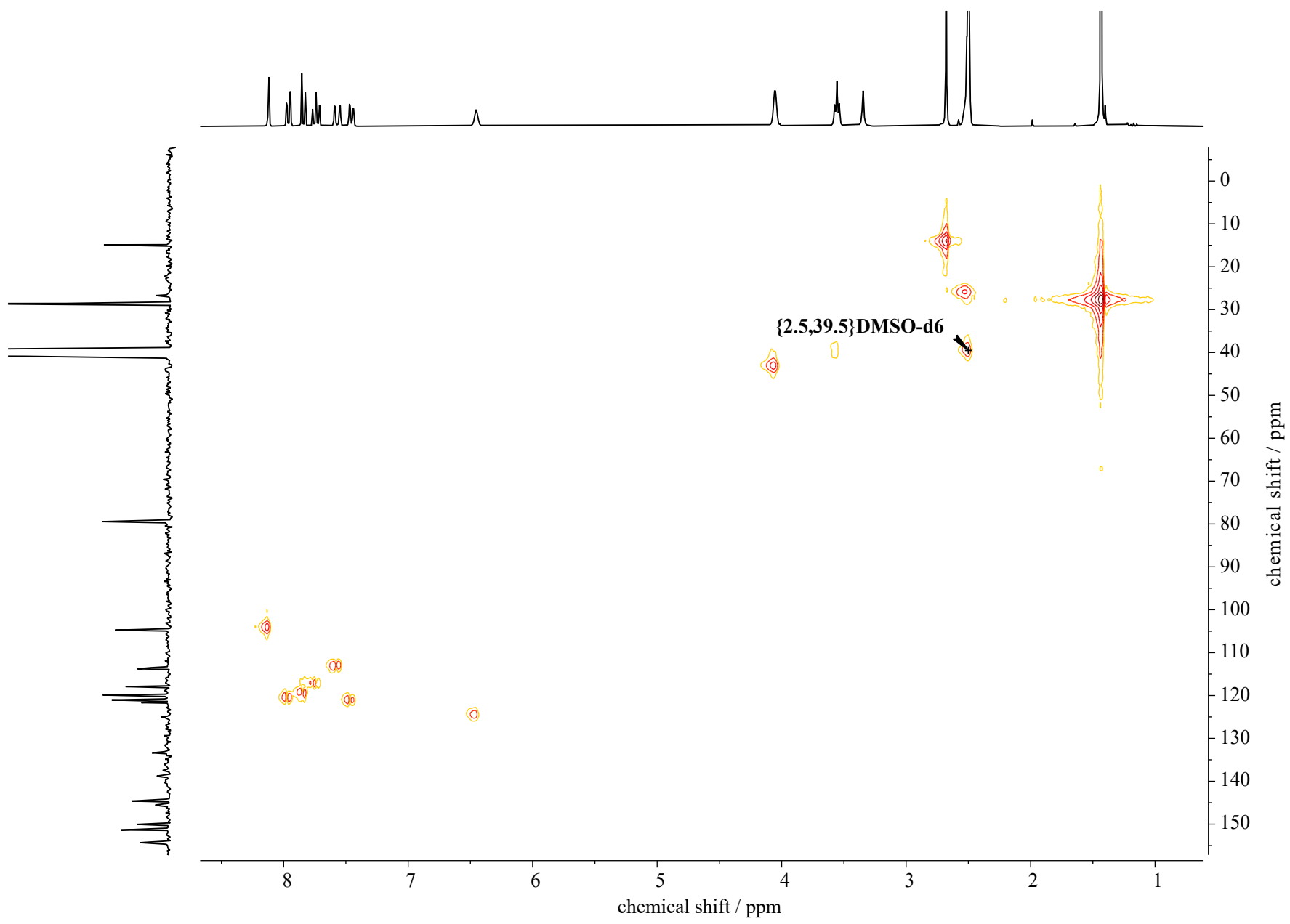


**Figure S8**. COSY and HMQC NMR spectra of **3** in DMSO-$d_6$.

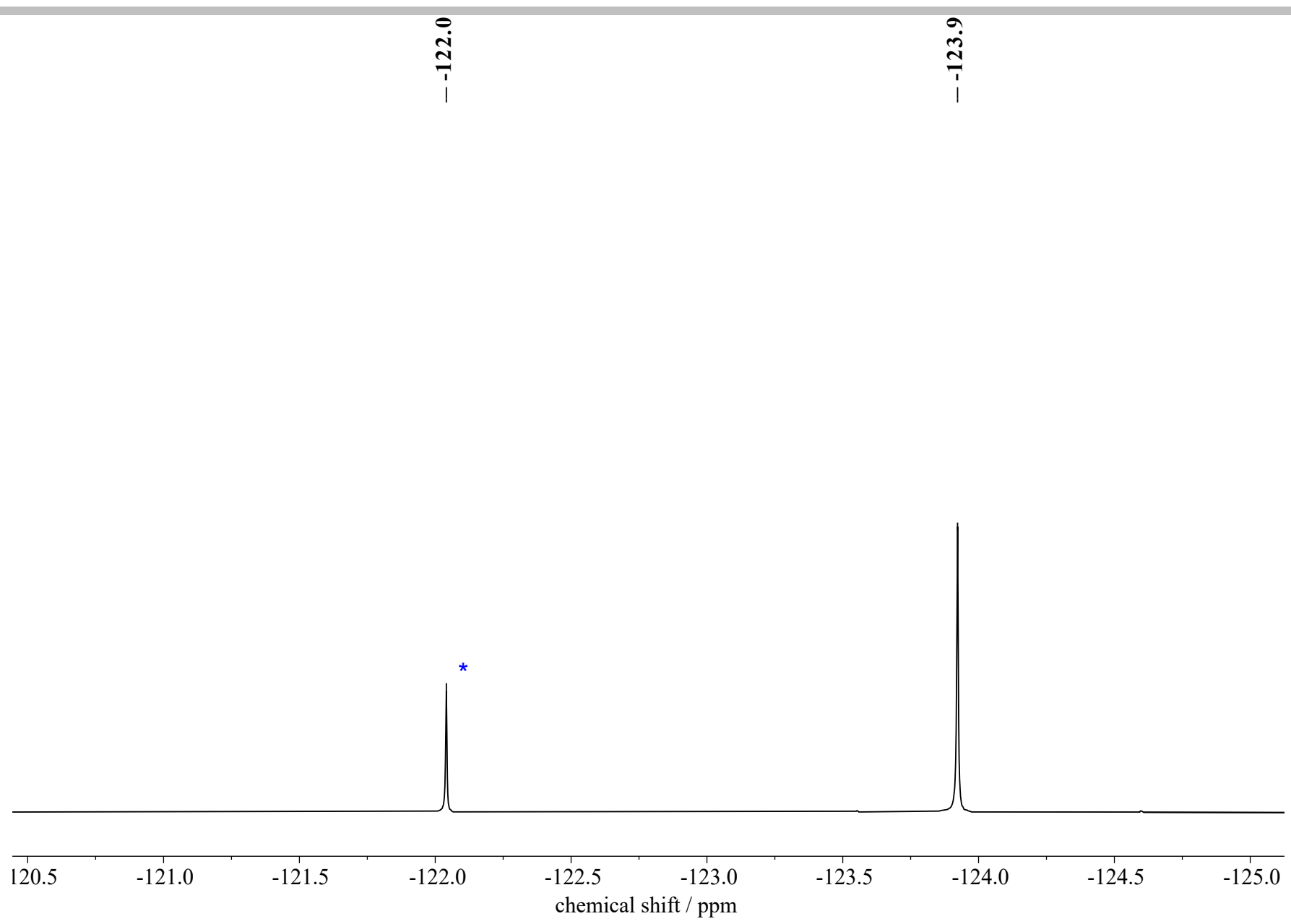


**Figure S9**. $^{19}$F (282 MHz) NMR spectra of **3** in DMSO-$d_6$.

**Synthesis of 6-((2-fluoro-4-(1-methyl-1,2,3,6-tetrahydropyridin-4-yl)phenyl)diazenyl)-2-methylbenzo[d]oxazole (7)**

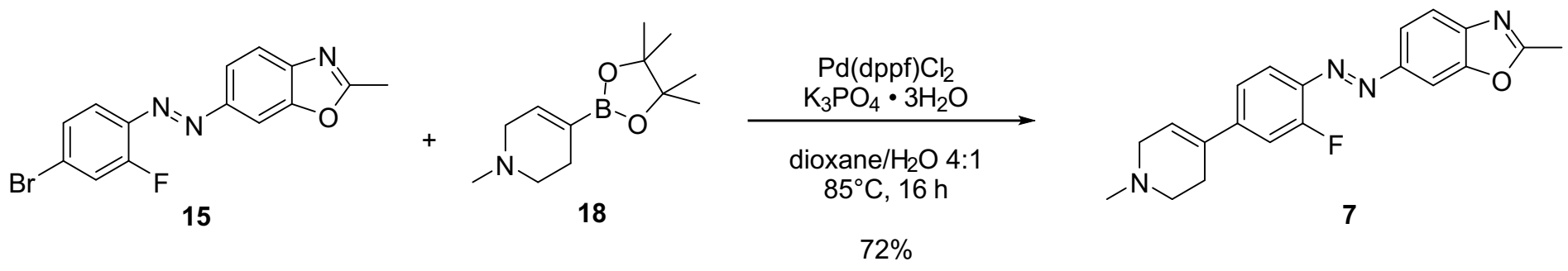


6-((4-bromo-2-fluorophenyl)diazenyl)-2-methylbenzo[*d*]oxazole **15** (50 mg, 154 µmol, 1.00 eq), 1-methyl-1,2,3,6-tetrahydropyridine-4-boronic acid pinacol ester **18** (35 mg, 154 µmol, 1.00 eq), Pd(dppf)$Cl_2$ (12 mg, 15.4 µmol, 0.10 eq) and $K_3PO_4 \cdot 3H_2O$ (123 mg, 461 µmol, 3.00 eq) were dissolved in mixture of dioxane/distilled water (20 mL, 4:1, v%). The reaction was stirred under $N_2$ atmosphere at 85 °C for 16 h. The reaction was then diluted with 100 mL distilled water and extracted with 100 mL EtOAc. The organic layer was washed with 100 mL distilled water and brine, dried over $MgSO_4$ and solvent was removed under reduced pressure. The crude product was purified by flash column chromatography (EtOAc/MeOH, 1:1, v%) to yield the desired compound 6-((2-fluoro-4-(1-methyl-1,2,3,6-tetrahydropyridin-4-yl)phenyl)diazenyl)-2-methylbenzo[*d*]oxazole (**7**, 39 mg, 111 µmol, 72%) as an orange solid.

**TLC:** $R_f$ = 0.24 (EtOAc/MeOH, 1:1, v%).

***trans*-isomer** (*cis*-isomer signals were labeled with * on NMR spectrum).

**$^1$H-NMR (300 MHz, DMSO-*d*$_6$, *δ*):** 8.14 (d, $^4J$ = 1.8 Hz, 1H, $H_{arom.}$), 7.97 (dd, $^3J$ = 8.5 Hz, $^4J$ = 1.8 Hz, 1H, $H_{arom.}$), 7.85 (d, $^3J$ = 8.5 Hz, 1H, $H_{arom.}$), 7.74 (t, $^3J$ = 8.3 Hz, 1H, $H_{arom.}$), 7.56 (dd, $^3J$ = 13.0 Hz, $^4J$ = 1.8 Hz, 1H, $H_{arom.}$), 7.48-7.45 (m, 1H, $H_{arom.}$), 6.48-6.46 (m, 1H, C*H*=C), 3.07-3.05 (m, 2H, C$H_2$), 2.68 (s, 3H, C$H_3$), 2.59-2.50 (m, 4H, C$H_2$, C$H_2$), 2.29 (s, 3H, C$H_3$) ppm.

**$^{13}$C-NMR (75 MHz, DMSO-*d*$_6$, *δ*):** 167.1 (N=*C*-O), 159.1 (d, $^1J_{FC}$ =257.6 Hz, *C*F), 150.9 ($C_{arom.}$), 149.6 ($C_{arom.}$), 145.2 (d, $^2J_{FC}$ =6.0 Hz, $C_{arom.}$), 144.1 ($C_{arom.}$), 132.1 (d, $^3J_{FC}$ =2.4 Hz, $C_{arom.}$), 130.9 ($C_{q.}$), 125.8 (*C*H=C), 120.9 (d, $^3J_{FC}$ =3.1 Hz, $C_{arom.}$H), 120.5 ($C_{arom.}$H), 119.4 ($C_{arom.}$H), 117.3 ($C_{arom.}$H), 112.9 (d, $^2J_{FC}$ = 20.9 Hz, $C_{arom.}$H), 104.3 ($C_{arom.}$H), 54.6 (*C*$H_2$), 51.5 (*C*$H_2$), 45.3 (*C*$H_3$), 27.0 (*C*$H_2$), 14.4 (*C*$H_3$) ppm.

**$^{19}$F-NMR (282 MHz, DMSO-*d*$_6$, *δ*):** -124.2 (C*F*) ppm.

**HRMS-ESI (m/z):** $[M+H]^+$ calcd for $C_{20}H_{20}FN_4O$, 351.1624; found, 351.1600.

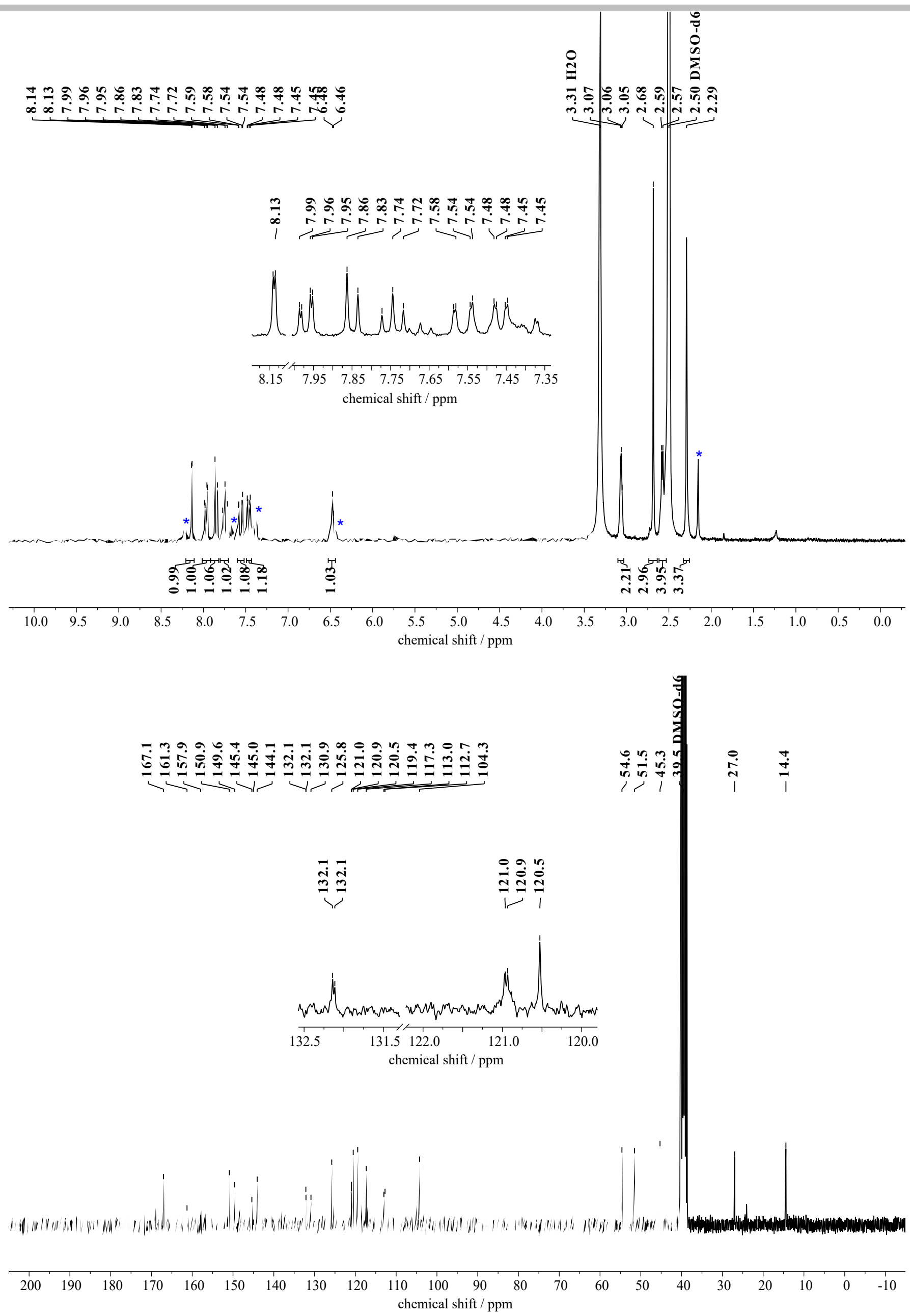

**Figure S10**. $^{1}H$ (300 MHz) and $^{13}C$ (75 MHz) NMR spectra of **7** in DMSO-$d_6$.

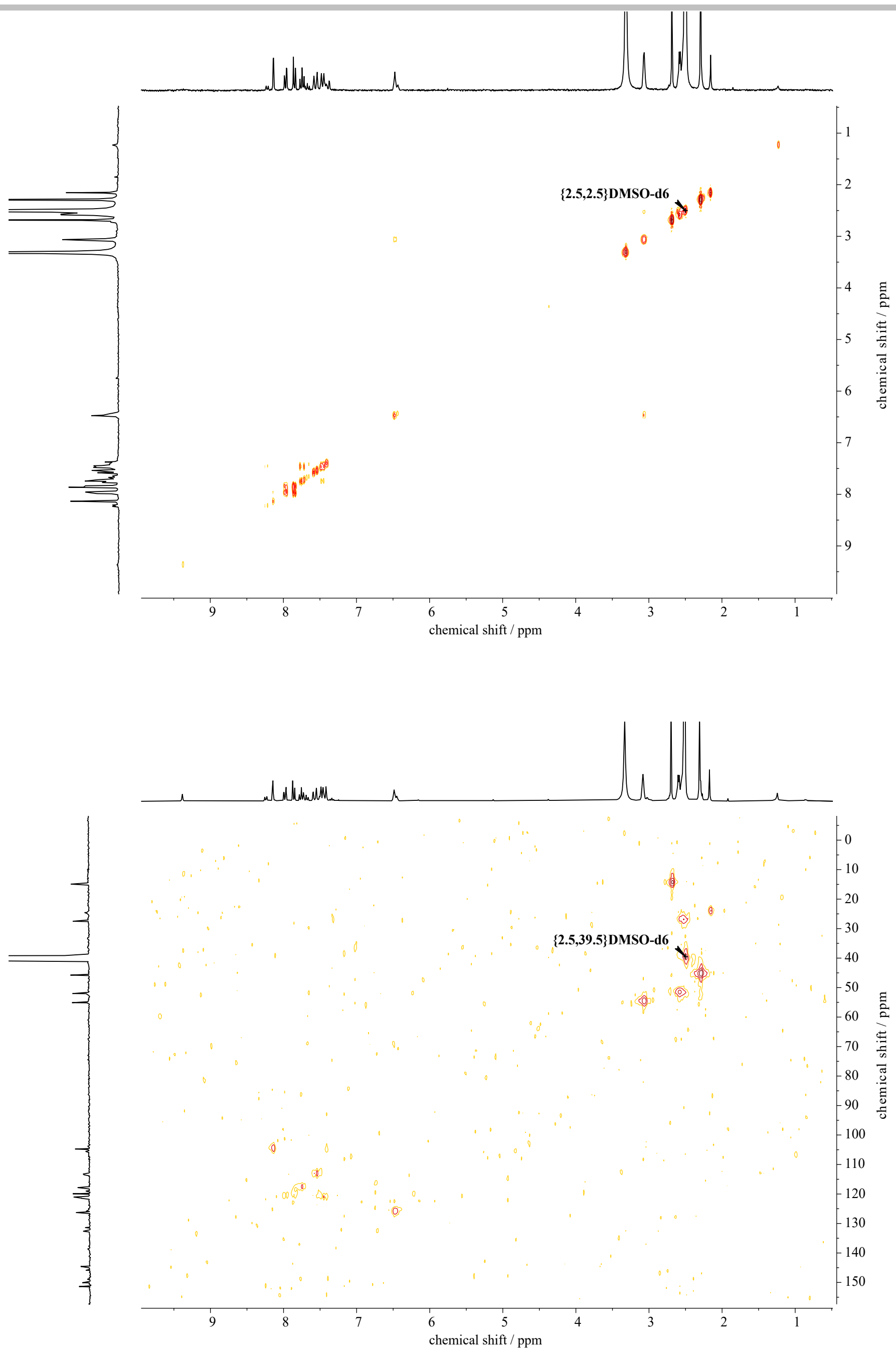


**Figure S11**. COSY and HMQC NMR spectra of **7** in DMSO-$d_6$.

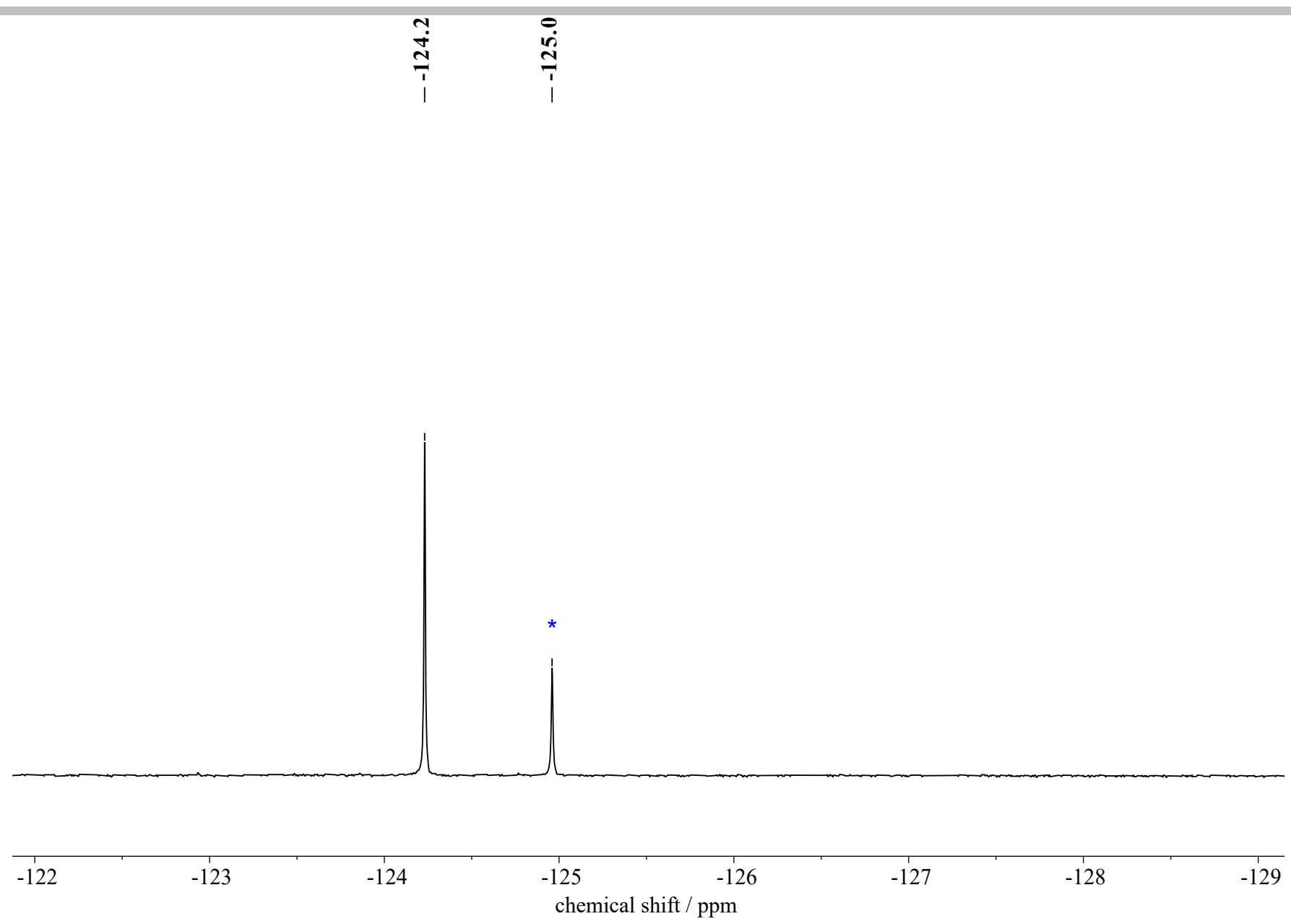


**Figure S12**. $^{19}$F (282 MHz) NMR spectra of **7** in DMSO-$d_6$.

**Synthesis of 6-((4-bromophenyl)diazenyl)-2-methylbenzo[d]oxazole (20)**

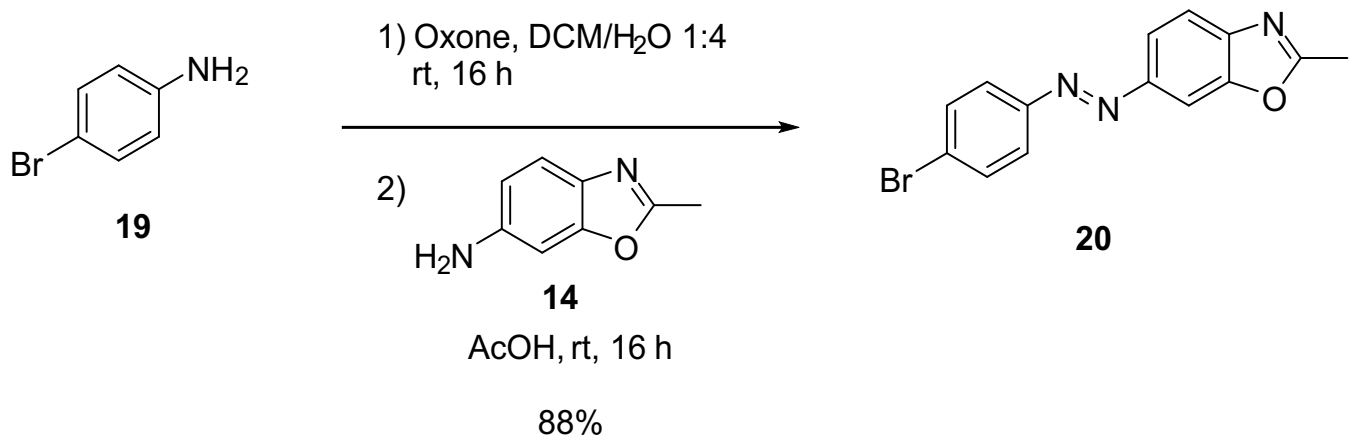


4-Bromoaniline **19** (313 mg, 1.82 mmol, 2.00 eq) was dissolved in 5 mL DCM, Oxone® (2.24 g, 7.29 mmol, 4.00 eq) was dissolved in 20 mL distilled water and added to the solution. The resulting mixture was stirred for 16 h at room temperature. The reaction mixture was then diluted with 100 mL DCM and washed with 100 mL 1 M HCl, distilled water and brine. The organic layer was dried over $MgSO_4$ and the solvent was removed under reduced pressure. The resulting brown solid and 2-methyl-1,3-benzoxazol-6-amine **14** (135 mg, 911 µmol, 1.00 eq) were suspended in 20 mL acetic acid. The reaction mixture was stirred for 16 h at room temperature. The reaction was then diluted with 200 mL distilled water and extracted with 200 mL EtOAc. The organic layer was washed with 200 mL distilled water and brine, dried over $MgSO_4$ and solvent was removed under reduced pressure. The crude product was purified by flash column chromatography (DCM) to yield the desired compound 6-((4-bromophenyl)diazenyl)-2-methylbenzo[*d*]oxazole (**20**, 253 mg, 801 µmol, 88%) as an orange solid.

**TLC:** $R_f$ = 0.39 (DCM).

***trans*-isomer** (*cis*-isomer signals were labeled with * on NMR spectrum).

**$^1$H-NMR (300 MHz, DMSO-*d*$_6$, *δ*):** 8.15 (d, $^4J$ = 1.7 Hz, 1H, $H_{arom.}$), 7.97 (dd, $^3J$ = 8.4 Hz, $^4J$ = 1.8 Hz, 1H, $H_{arom.}$), 7.87-7.79 (m, 5H, $H_{arom.}$, 2x$H_{arom.}$, 2x$H_{arom.}$), 2.68 (s, 3H, C$H_3$) ppm.

**$^{13}$C-NMR (75 MHz, DMSO-*d*$_6$, *δ*):** 167.0 (N=*C*-O), 150.9 ($C_{arom.}$), 150.7 ($C_{arom.}$), 149.1 ($C_{arom.}$), 144.1 ($C_{arom.}$), 132.5 ($C_{arom.}$H), 131.9 ($C_{arom.}$H), 124.9 ($C_{arom.}$H), 124.4 ($C_{arom.}$H), 122.1 ($C_{arom.}$), 120.7 ($C_{arom.}$H), 119.4 ($C_{arom.}$H), 104.0 ($C_{arom.}$H), 14.4 ($CH_3$) ppm.

**HRMS-ESI (m/z):** $[M+H]^+$ calcd for $C_{14}H_{11}BrN_3O$, 317.0114; found, 317.0117.

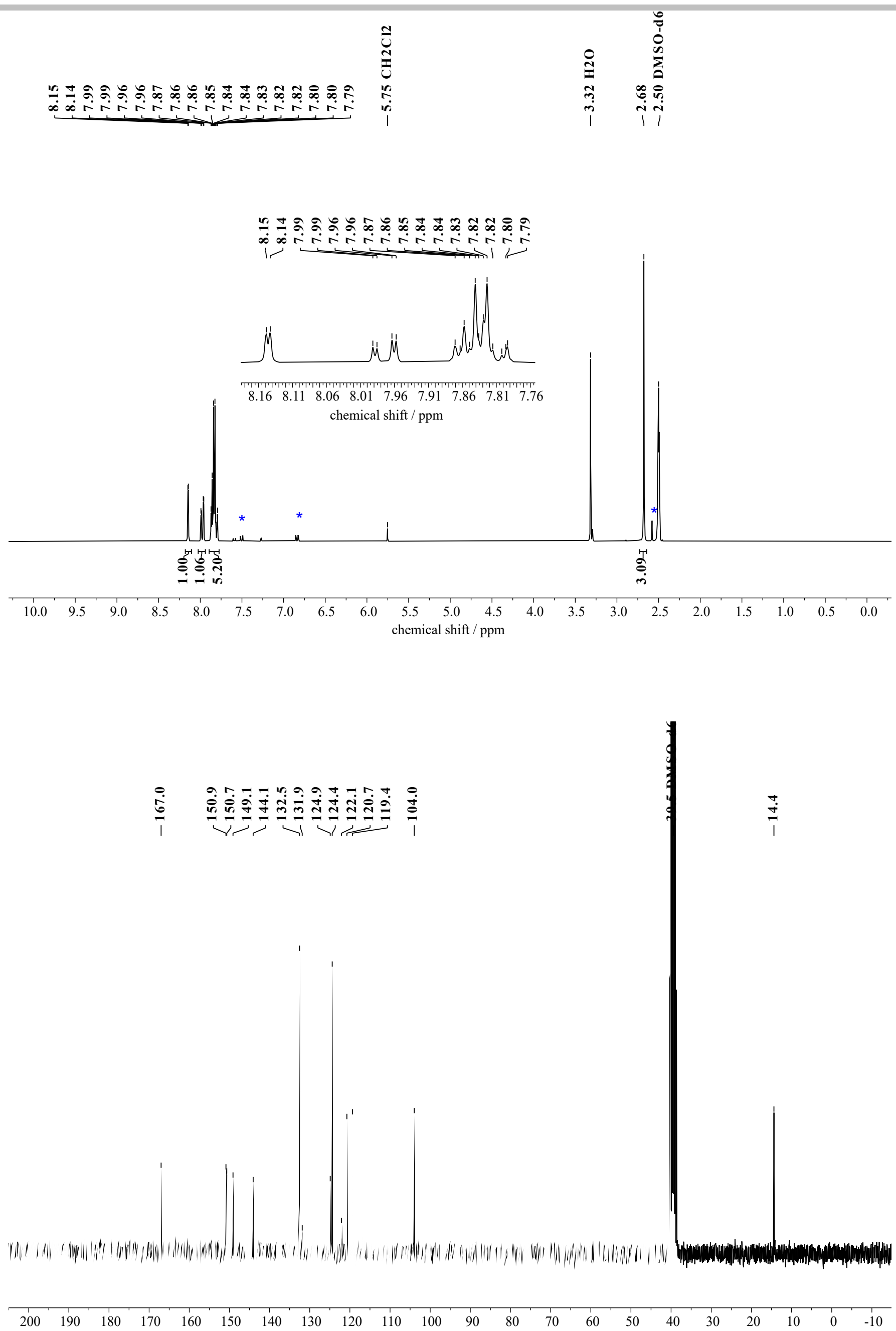


**Figure S13**. $^{1}H$ (300 MHz) and $^{13}C$ (75 MHz) NMR spectra of **20** in DMSO-$d_6$.

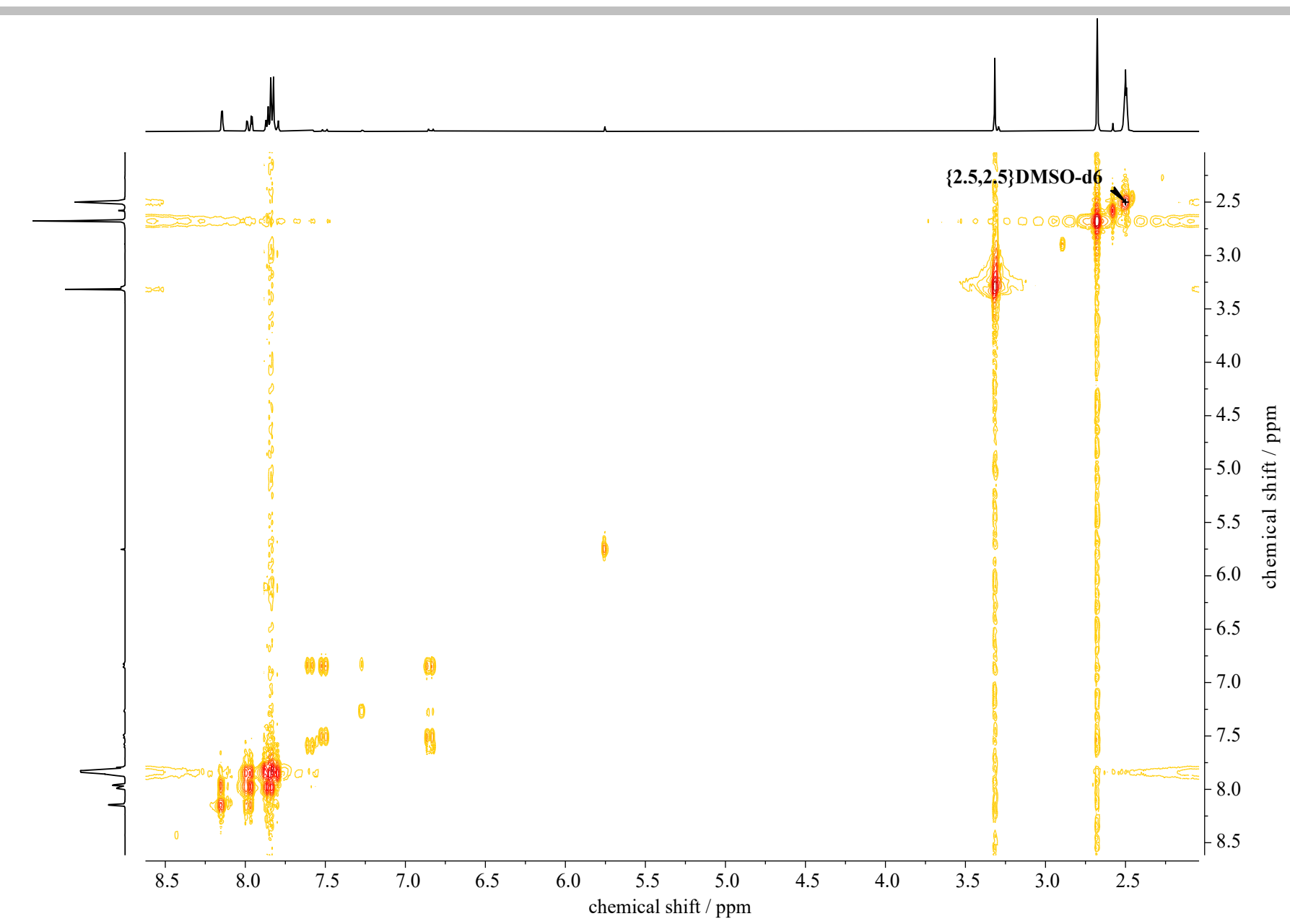


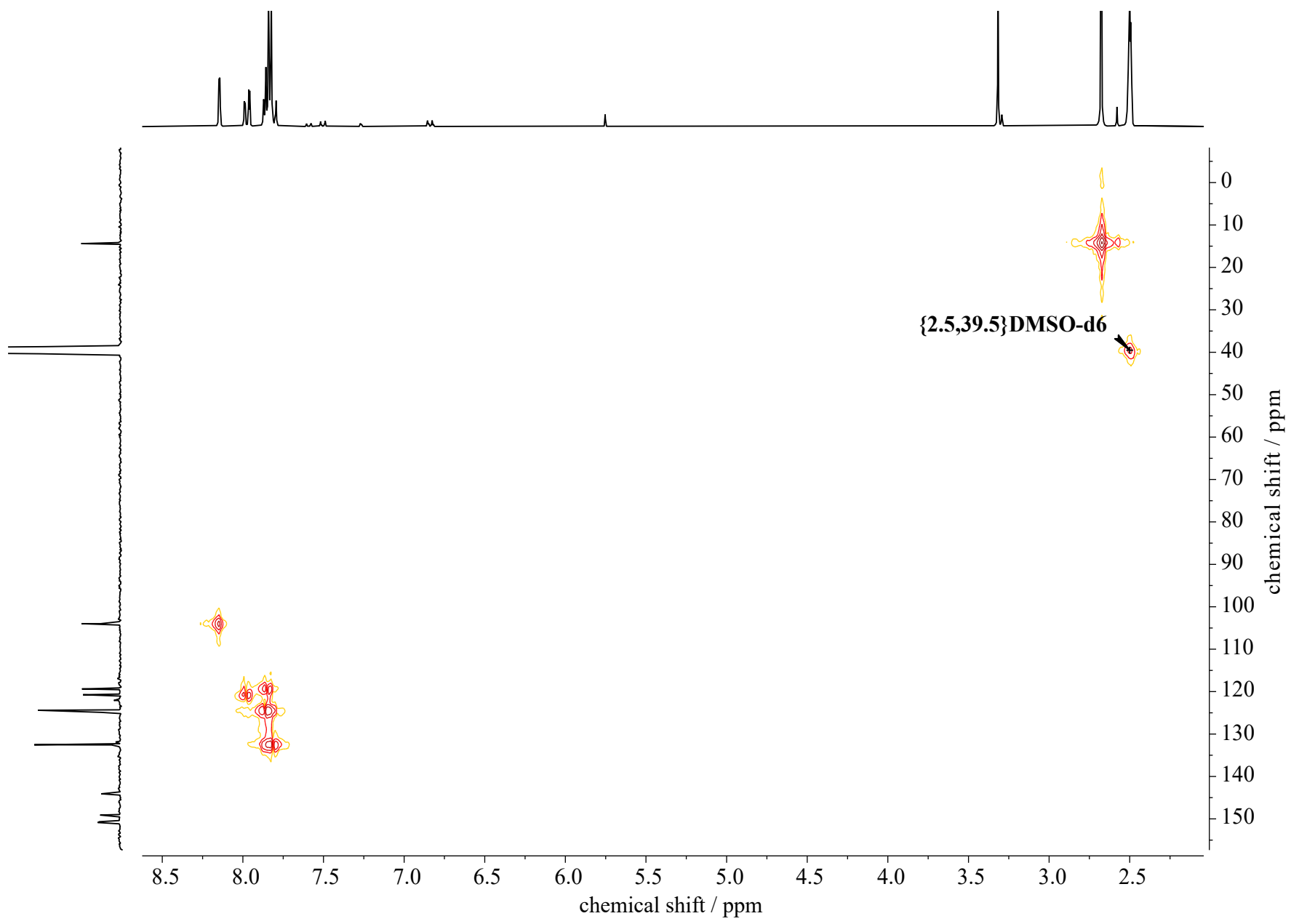


**Figure S14**. COSY and HMQC NMR spectra of **20** in DMSO-*d*$_6$.

**Synthesis of tert-butyl-4-(4-((2-methylbenzo[d]oxazol-6-yl)diazenyl)phenyl)-3,6-dihydropyridine-1(2H)-carboxylate (21)**

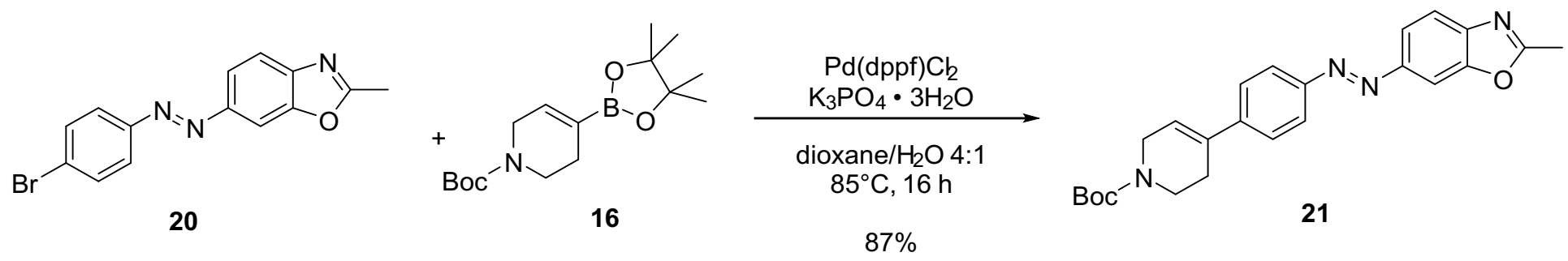


6-((4-Bromophenyl)diazenyl)-2-methylbenzo[*d*]oxazole **20** (100 mg, 316 µmol, 1.00 eq), *N*-*tert*-butoxycarbony-1,2,3,6-tetrahydropyridine-4-boronic acid pinacol ester **16** (98 mg, 316 µmol, 1.00 eq), Pd(dppf)$Cl_2$ (23 mg, 31.6 µmol, 0.10 eq) and $K_3PO_4$·$3H_2O$ (253 mg, 949 µmol, 3.00 eq) were dissolved in mixture of dioxane/distilled water (20 mL, 4:1, v%). The reaction was stirred under $N_2$ atmosphere at 85 °C for 16 h. The reaction was then diluted with 100 mL distilled water and extracted with 100 mL EtOAc. The organic layer was washed with 100 mL distilled water and brine, dried over $MgSO_4$ and solvent was removed under reduced pressure. The crude product was purified by flash column chromatography (DCM/EtOAc, 1:1, v%) to yield the desired compound *tert*-butyl-4-(4-((2-methylbenzo[d]oxazol-6-yl)diazenyl)phenyl)-3,6-dihydropyridine-1(*2H*)-carboxylate (**21**, 115 mg, 275 µmol, 87%) as an orange solid.

**TLC:** $R_f$ = 0.62 (DCM/EtOAc, 1:1, v%).

**$^1$H-NMR (300 MHz, DMSO-$d_6$, δ):** 8.14 (d, $^4J$ = 1.8 Hz, 1H, $H_{arom.}$), 7.97 (dd, $^3J$ = 8.5 Hz, $^4J$ = 1.8 Hz, 1H, $H_{arom.}$), 7.90 (d, $^3J$ = 8.6 Hz, 2H, 2x$H_{arom.}$), 7.84 (t, $^3J$ = 8.4 Hz, 1H, $H_{arom.}$), 7.68 (d, $^3J$ = 8.6 Hz, 2H, 2x$H_{arom.}$), 6.37 (s, 1H, C*H*=C), 4.06-4.05 (m, 2H, C$H_2$), 3.57 (t, $^3J$ = 5.7 Hz, 2H, C$H_2$), 2.68 (s, 3H, C$H_3$), 2.54-2.50 (m, 2H, C$H_2$), 1.44 (s, 9H, 3xC$H_3$) ppm.

**$^{13}$C-NMR (75 MHz, DMSO-$d_6$, δ):** 166.8 (N=*C*-O), 153.6 ($C_{arom.}$), 150.9 ($C_{arom.}$), 150.7 ($C_{arom.}$), 149.4 ($C_{arom.}$), 143.8 ($C_{arom.}$), 142.8 ($C_{arom.}$), 133.7 ($C_{q.}$), 125.6 (2C, 2x$C_{arom.}$H), 123.4 (*C*H=C), 122.8 (2C, 2x$C_{arom.}$H), 120.6 ($C_{arom.}$H), 119.3 ($C_{arom.}$H), 103.8 ($C_{arom.}$H), 78.9 ($C_{q.}$), 28.1 (3C, 3x$CH_3$), 26.1 ($CH_2$), 14.4 ($CH_3$) ppm. The signal of the carbon from $CH_2$ is overlapped with DMSO-$d_6$ signal, cross signal indicates this carbon is labelled on 2D-HMQC spectrum.

**HRMS-ESI (m/z):** $[M+Na]^+$ calcd for $C_{24}H_{26}N_4O_3Na$, 441.1897; found, 441.1915.

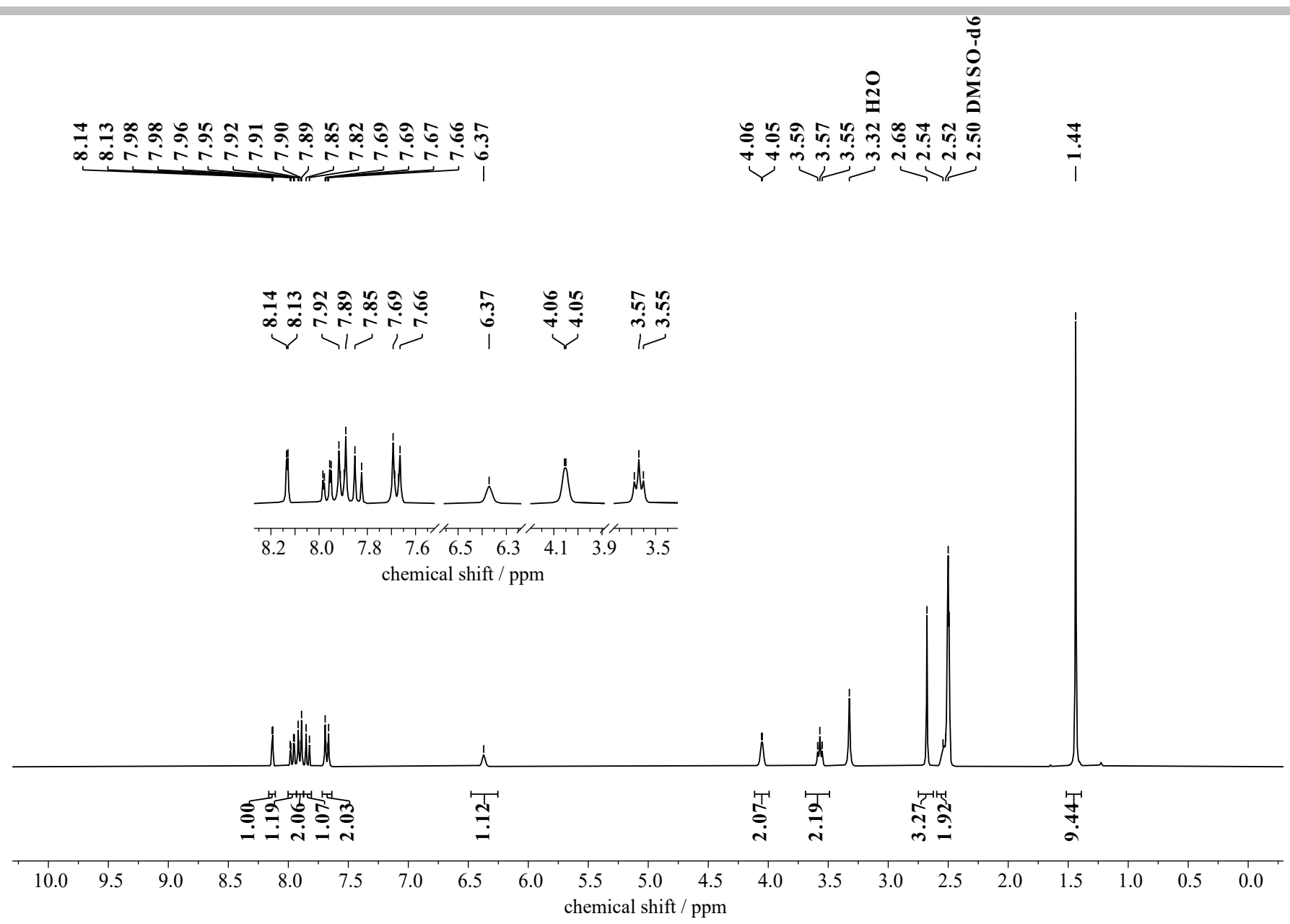


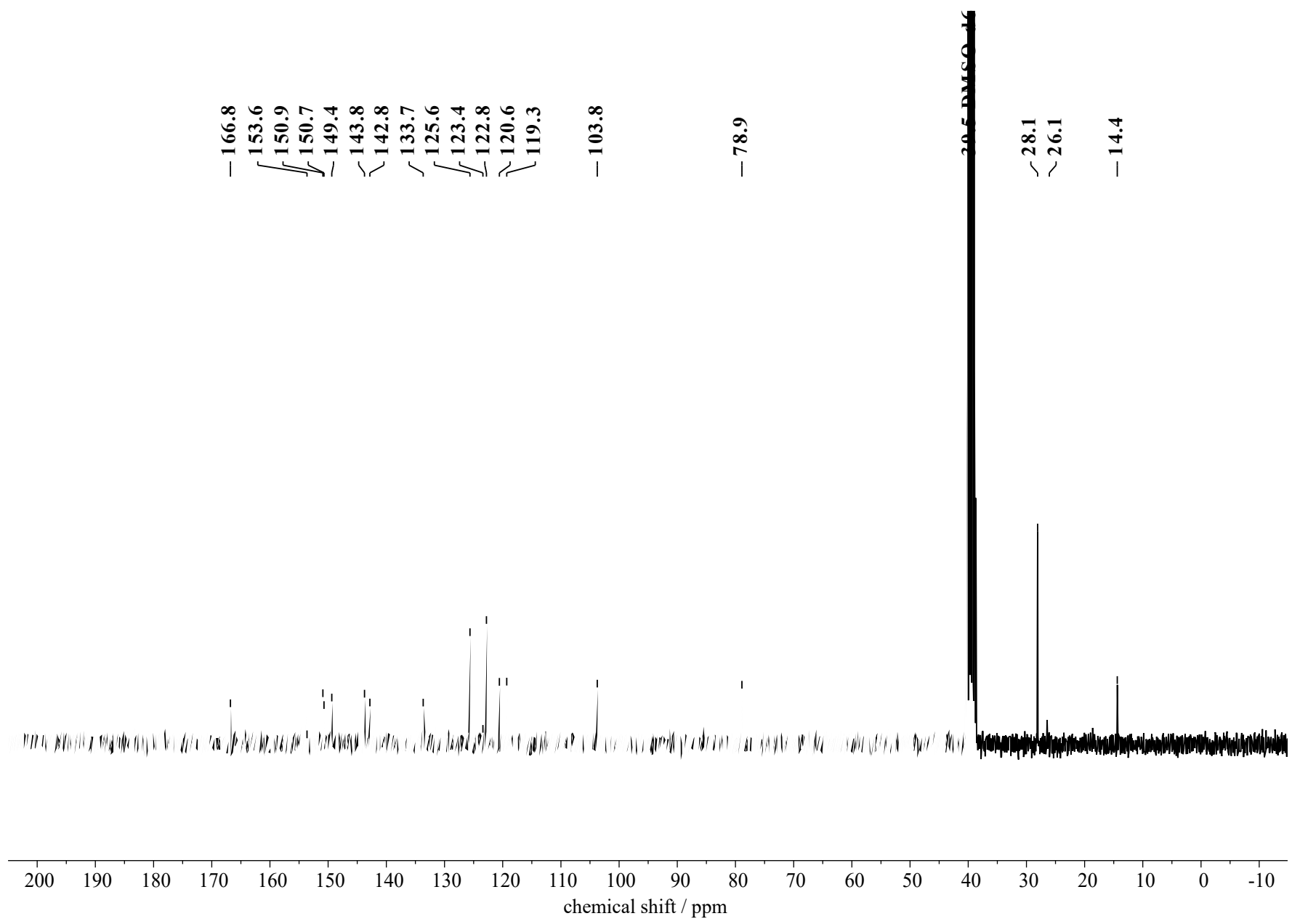


**Figure S15**. $^{1}$H (300 MHz) and $^{13}$C (75 MHz) NMR spectra of **21** in DMSO-$d_6$.

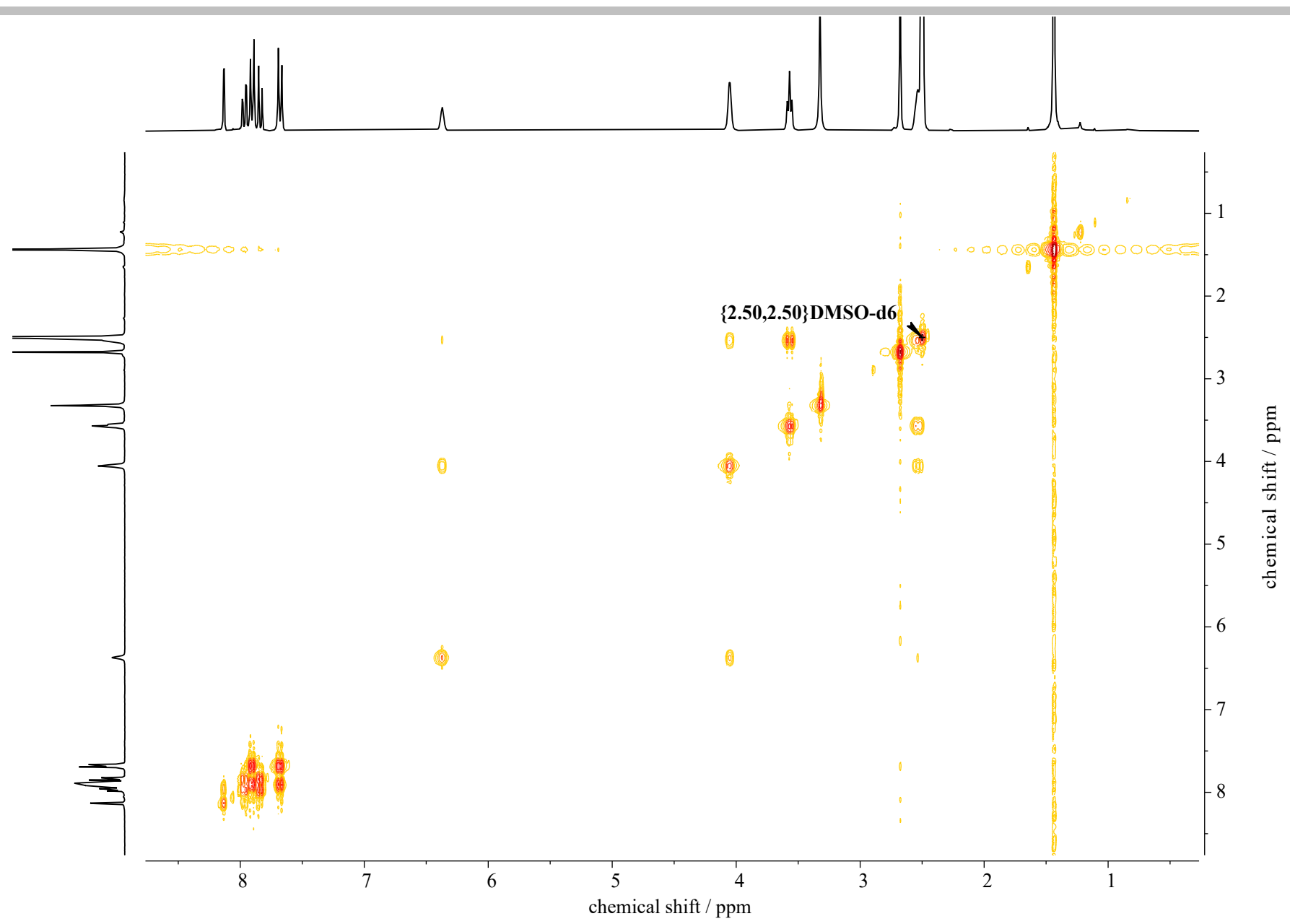


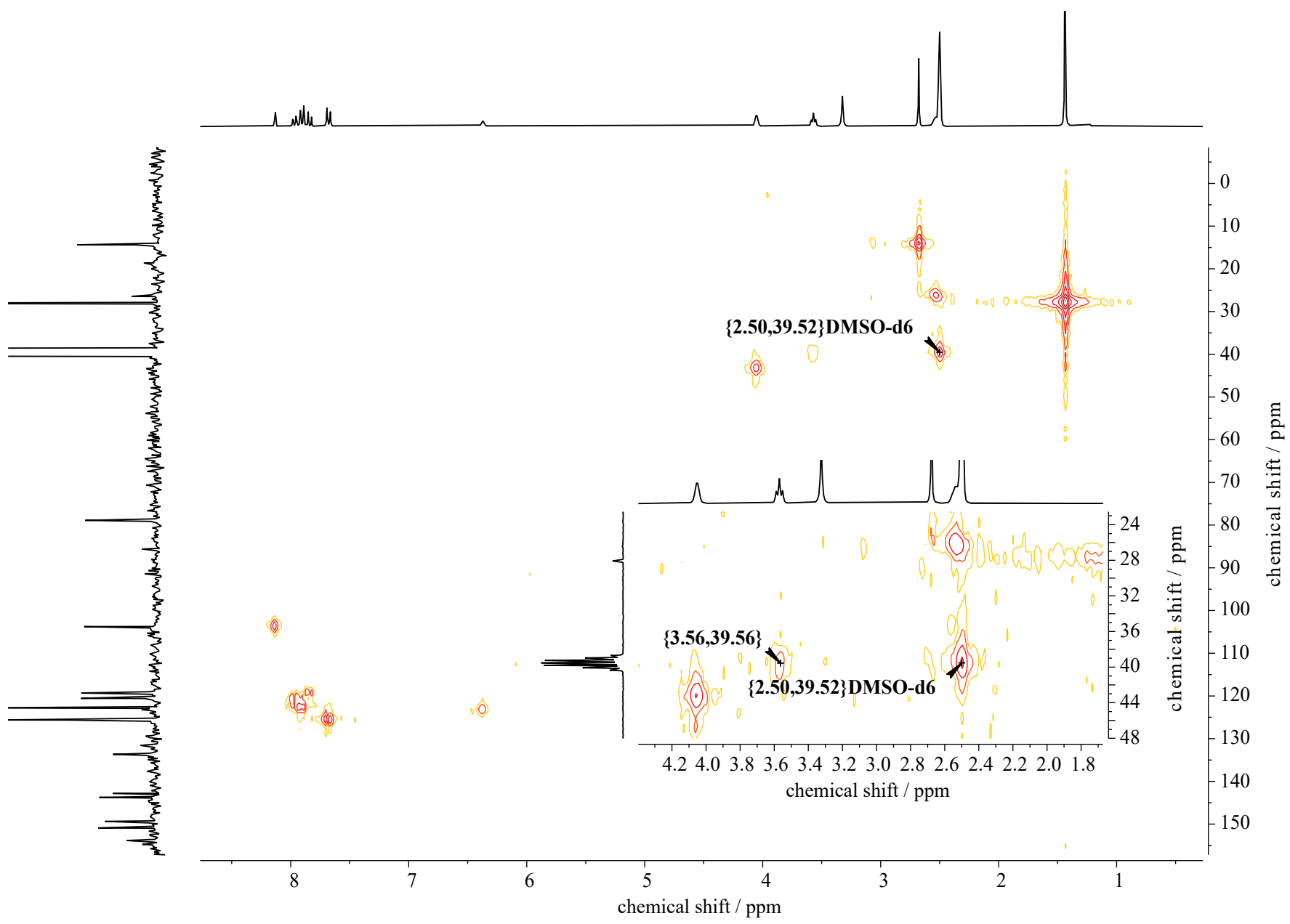


**Figure S16**. COSY and HMQC NMR spectra of **21** in DMSO-$d_6$.

**Synthesis of 4-(4-((2-methylbenzo[d]oxazol-6-yl)diazenyl)phenyl)-1,2,3,6-tetrahydropyridin (2)**

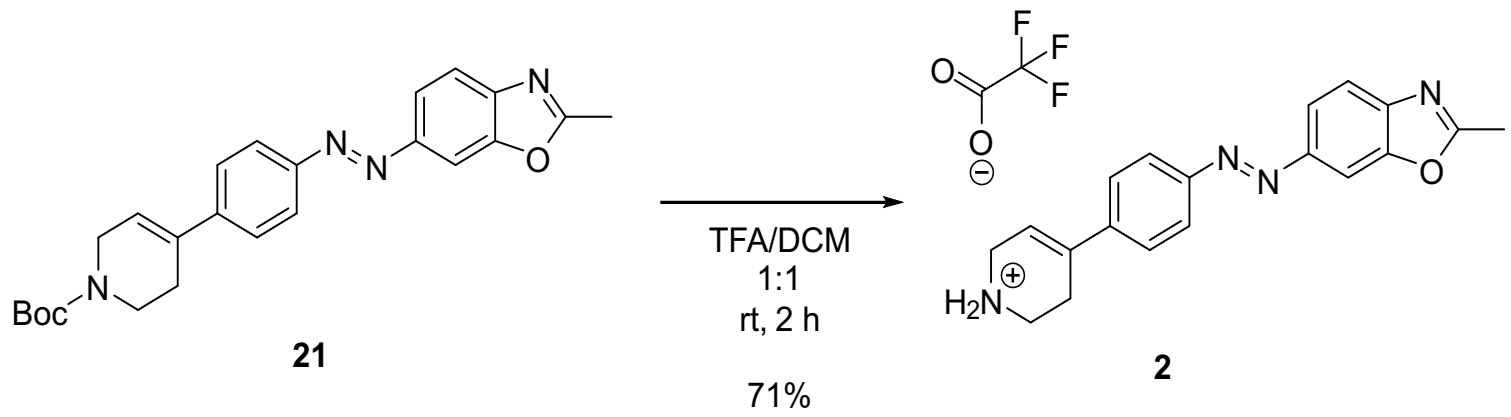


*Tert*-butyl-4-(4-((2-methylbenzo[*d*]oxazol-6-yl)diazenyl)phenyl)-3,6-dihydropyridine-1(*2H*)-carboxylate **21** (50 mg, 119 µmol, 1.00 eq) was dissolved in mixture of TFA/DCM (10 mL, 1:1, v%). The reaction was stirred at room temperature for 2 h. The reaction was then diluted with 100 mL distilled water and extracted with 100 mL EtOAc. The organic layer was washed with 100 mL distilled water and brine, dried over $MgSO_4$ and solvent was removed under reduced pressure. The crude product was purified by flash column chromatography (EtOAc/MeOH, 1:1, v%) to yield the desired compound 4-(4-((2-methylbenzo[*d*]oxazol-6-yl)diazenyl)phenyl)-1,2,3,6-tetrahydropyridin (**2**, 37 mg, 84.8 µmol, 71%) as an orange solid in form of a TFA salt.

**TLC:** $R_f$ = 0.33 (EtOAc/MeOH, 1:1, v%).

***trans*-isomer** (*cis*-isomer signals were labeled with * on NMR spectrum).

**$^1$H-NMR (300 MHz, DMSO-*d*$_6$, *δ*):** 8.82 (br, 2H, N$H_2$), 8.15 (d, $^4J$ = 1.8 Hz, 1H, $H_{arom.}$), 8.03-7.91 (m, 3H, $H_{arom.}$, $H_{arom.}$, $H_{arom.}$), 7.85 (d, $^3J$ = 8.4 Hz, 1H, $H_{arom.}$), 7.74 (t, $^3J$ = 8.6 Hz, 1H, $H_{arom.}$), 6.41-6.40 (m, 1H, C*H*=C), 3.83 (s, 2H, C$H_2$), 3.40-3.36 (t, $^3J$ = 6.0 Hz, 2H, C$H_2$), 2.76-2.73 (m, 2H, C$H_2$), 2.68 (s, 3H, C$H_3$) ppm.

**$^{13}$C-NMR (75 MHz, DMSO-*d*$_6$, *δ*):** 166.9 (N=*C*-O), 151.1 ($C_{arom.}$), 150.9 ($C_{arom.}$), 149.3 ($C_{arom.}$), 143.9 ($C_{arom.}$), 141.5 ($C_{arom.}$), 133.5 ($C_{q.}$), 125.8 (2C, 2x$C_{arom.}$H), 122.9 (2C, 2x$C_{arom.}$H), 120.7 (*C*H=C), 119.4 ($C_{arom.}$H), 118.9 ($C_{arom.}$H), 103.9 ($C_{arom.}$H), 63.5 ($C_{q.}$), 41.8 (*C*$H_2$), 23.1 (*C*$H_2$), 14.4 (*C*$H_3$) ppm.

**HRMS-ESI (m/z):** $[M+H]^+$ calcd for $C_{19}H_{19}N_4O$, 319.1553; found, 319.1562.

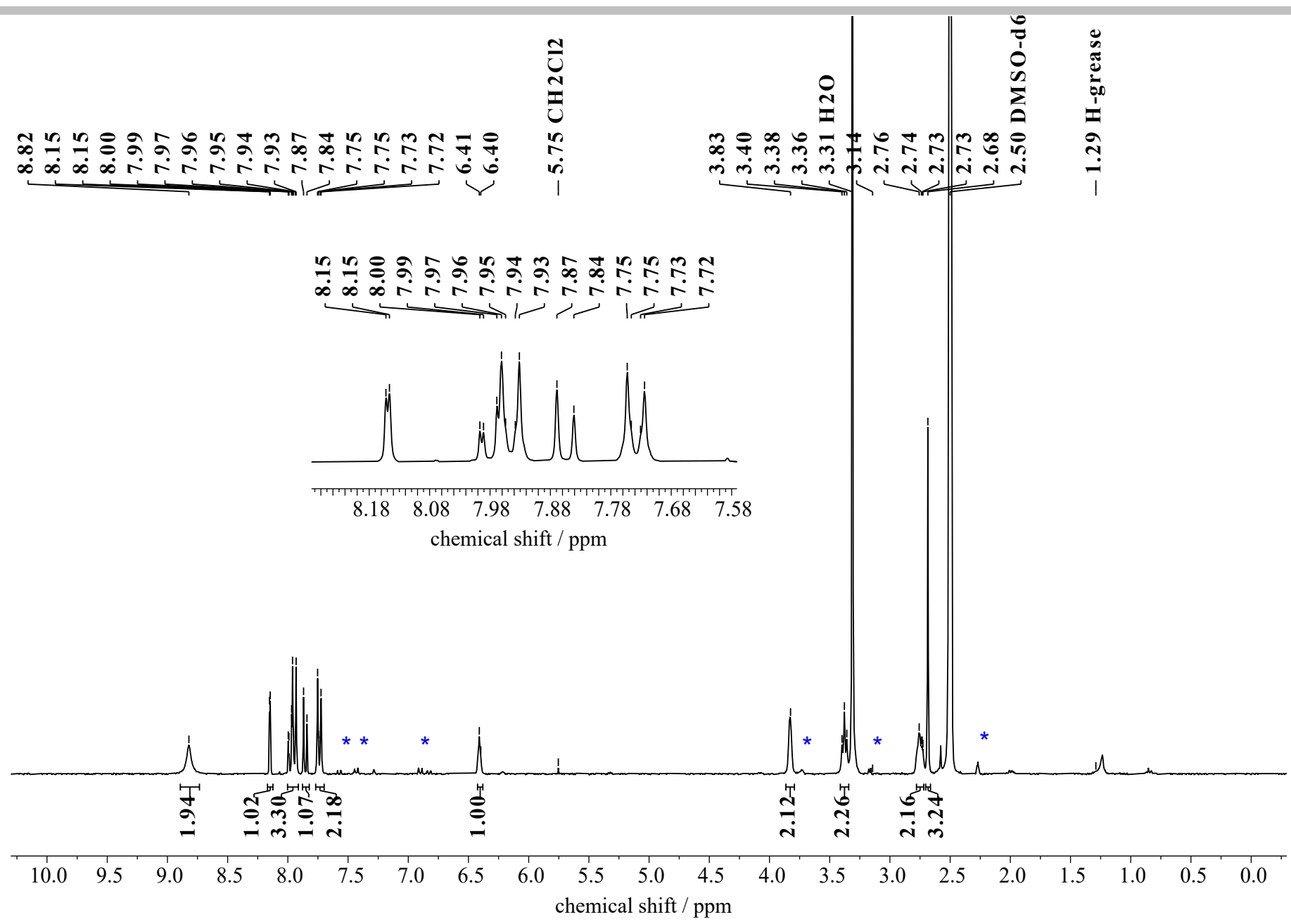


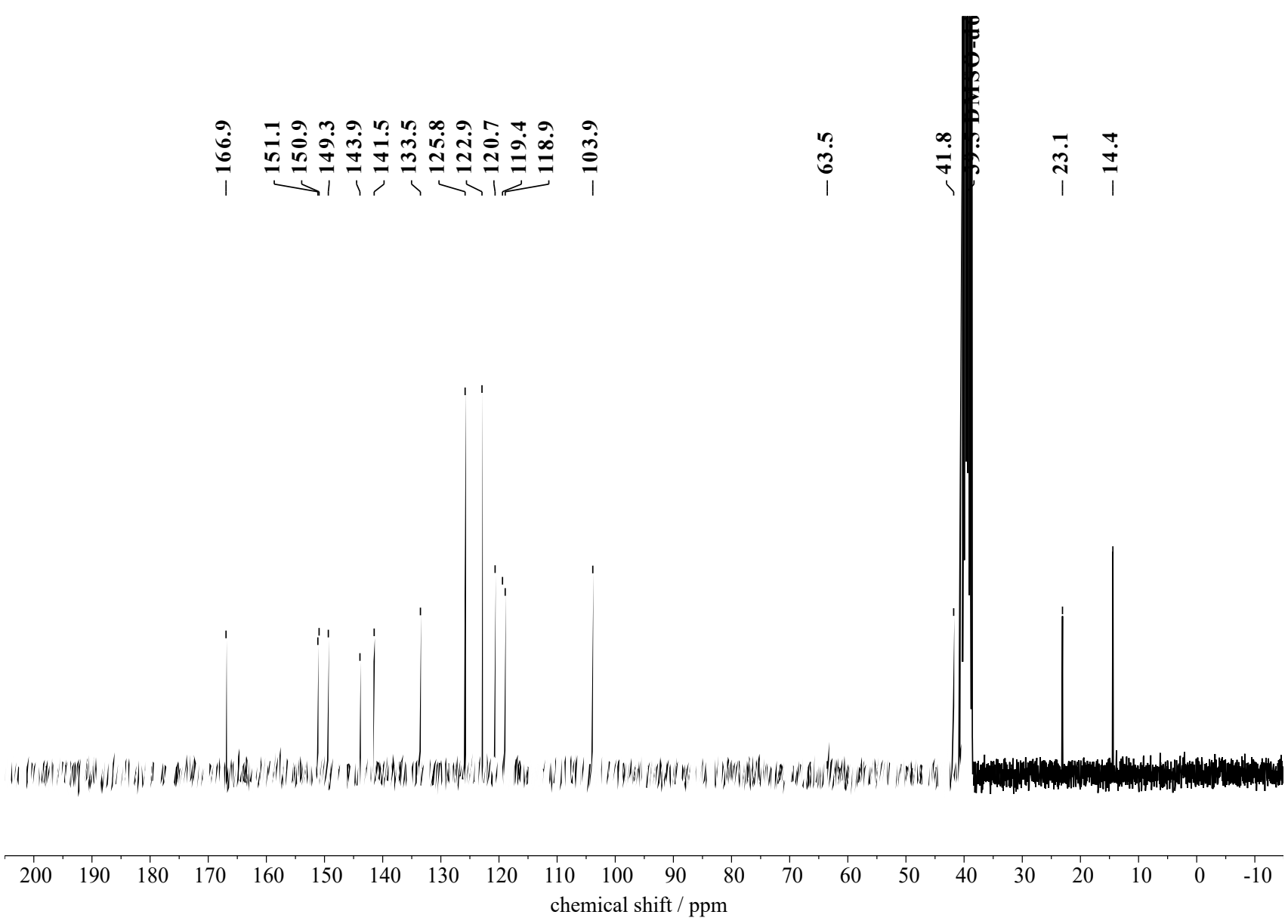


**Figure S17**. $^{1}H$ (300 MHz) and $^{13}C$ (75 MHz) NMR spectra of **2** in DMSO-$d_6$.

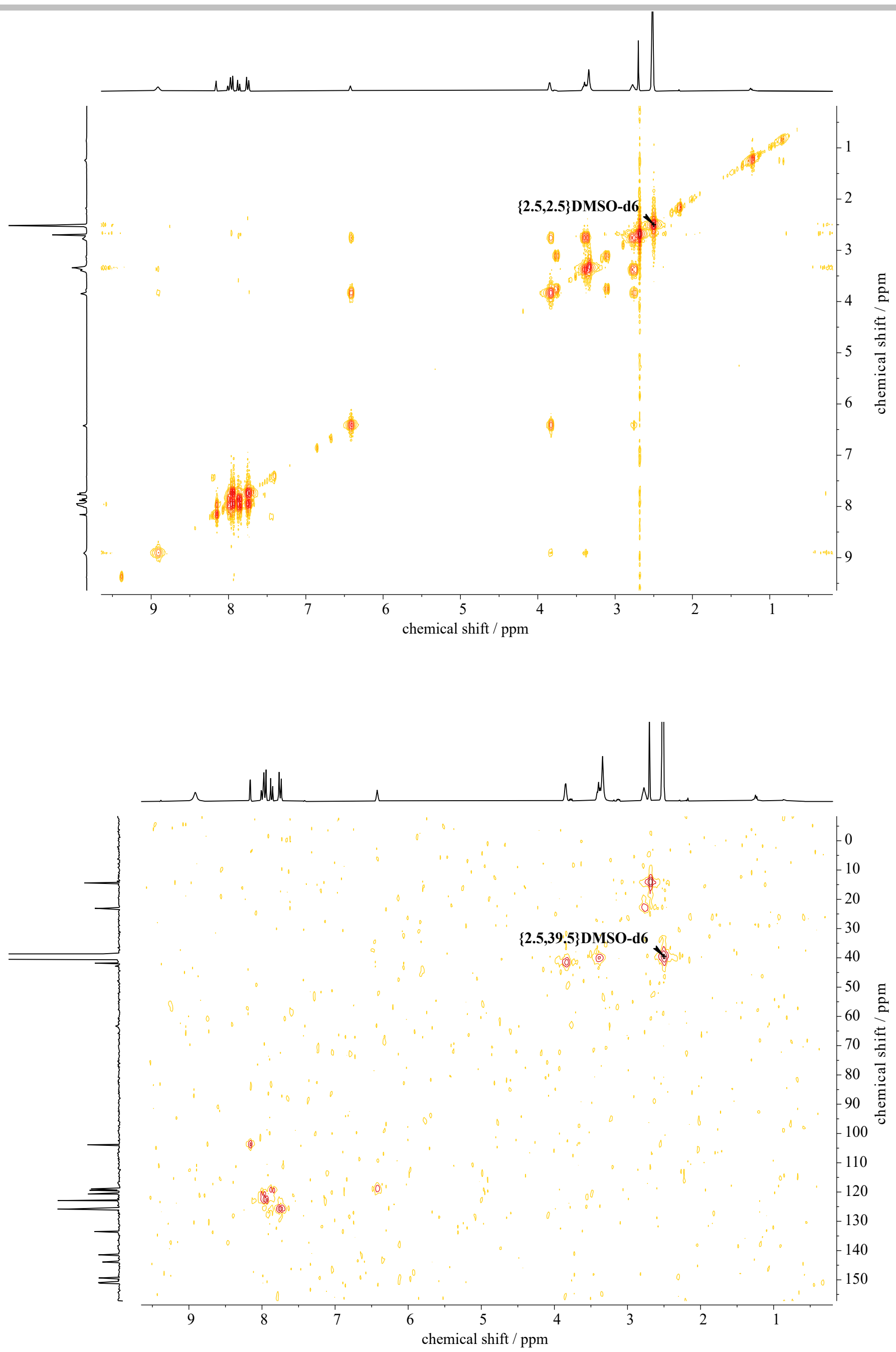


**Figure S18**. COSY and HMQC NMR spectra of **2** in DMSO-*d*$_6$.

**Synthesis of 2-methyl-6-((4-(1-methyl-1,2,3,6-tetrahydropyridin-4-yl)phenyl)diazenyl)benzo[d]oxazole (6)**

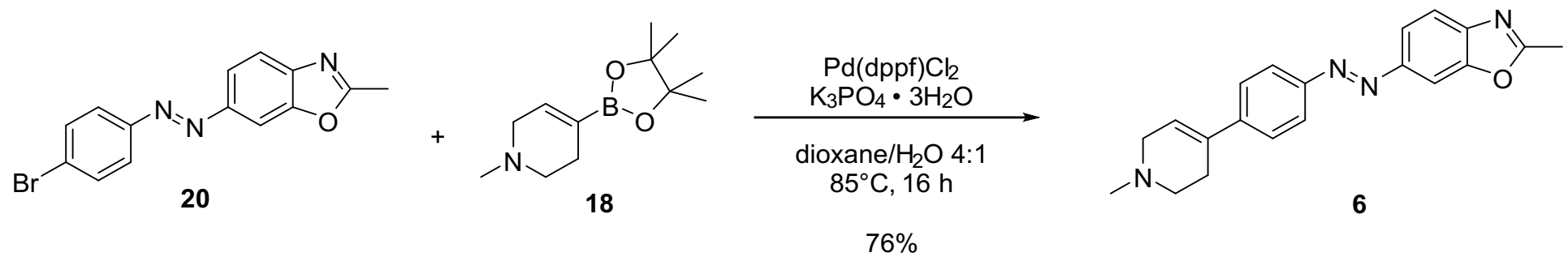


6-((4-Bromophenyl)diazenyl)-2-methylbenzo[*d*]oxazole **20** (50 mg, 158 µmol, 1.00 eq), 1-methyl-1,2,3,6-tetrahydropyridine-4-boronic acid pinacol ester **18** (36 mg, 158 µmol, 1.00 eq), Pd(dppf)$Cl_2$ (12 mg, 15.8 µmol, 0.10 eq) and $K_3PO_4 \cdot 3H_2O$ (126 mg, 474 µmol, 3.00 eq) were dissolved in mixture of dioxane/distilled water (20 mL, 4:1, v%). The reaction was stirred under $N_2$ atmosphere at 85 °C for 16 h. The reaction was then diluted with 100 mL distilled water and extracted with 100 mL EtOAc. The organic layer was washed with 100 mL distilled water and brine, dried over $MgSO_4$ and solvent was removed under reduced pressure. The crude product was purified by flash column chromatography (EtOAc/MeOH, 2:1, v%) to yield the desired compound 2-methyl-6-((4-(1-methyl-1,2,3,6-tetrahydropyridin-4-yl)phenyl)diazenyl)benzo[*d*]oxazole (**6**, 40 mg, 120 µmol, 76%) as an orange solid.

**TLC:** $R_f$ = 0.23 (EtOAc/MeOH, 2:1, v%).

**$^1$H-NMR (300 MHz, DMSO-$d_6$, $\delta$):** 8.13 (d, $^4J$ = 1.8 Hz, 1H, $H_{arom.}$), 7.96 (dd, $^3J$ = 8.5 Hz, $^4J$ = 1.8 Hz, 1H, $H_{arom.}$), 7.89 (d, $^3J$ = 8.6 Hz, 2H, 2x$H_{arom.}$), 7.84 (d, $^3J$ = 8.5 Hz, 1H, $H_{arom.}$), 7.67 (dd, $^3J$ = 8.6 Hz, 2H, 2x$H_{arom.}$), 6.39-6.37 (m, 1H, C*H*=C), 3.08-3.05 (m, 2H, C$H_2$), 2.68 (s, 3H, C$H_3$), 2.59-2.54 (m, 4H, C$H_2$, C$H_2$), 2.29 (s, 3H, C$H_3$) ppm.

**$^{13}$C-NMR (75 MHz, DMSO-$d_6$, $\delta$):** 166.8 (N=*C*-O), 150.9 ($C_{arom.}$), 150.6 ($C_{arom.}$), 149.4 ($C_{arom.}$), 143.7 ($C_{arom.}$), 143.0 ($C_{arom.}$), 132.9 ($C_{q.}$), 125.4 (2C, 2x$C_{arom.}$H), 124.4 (*C*H=C), 122.8 (2C, 2x$C_{arom.}$H), 120.6 ($C_{arom.}$H), 119.3 ($C_{arom.}$H), 103.8 ($C_{arom.}$H), 54.7 ($CH_2$), 51.7 ($CH_2$), 45.4 ($CH_3$), 27.2 ($CH_2$), 14.4 ($CH_3$) ppm.

**HRMS-ESI (m/z):** $[M+H]^+$ calcd for $C_{20}H_{21}N_4O$, 333.1710; found, 333.1720.

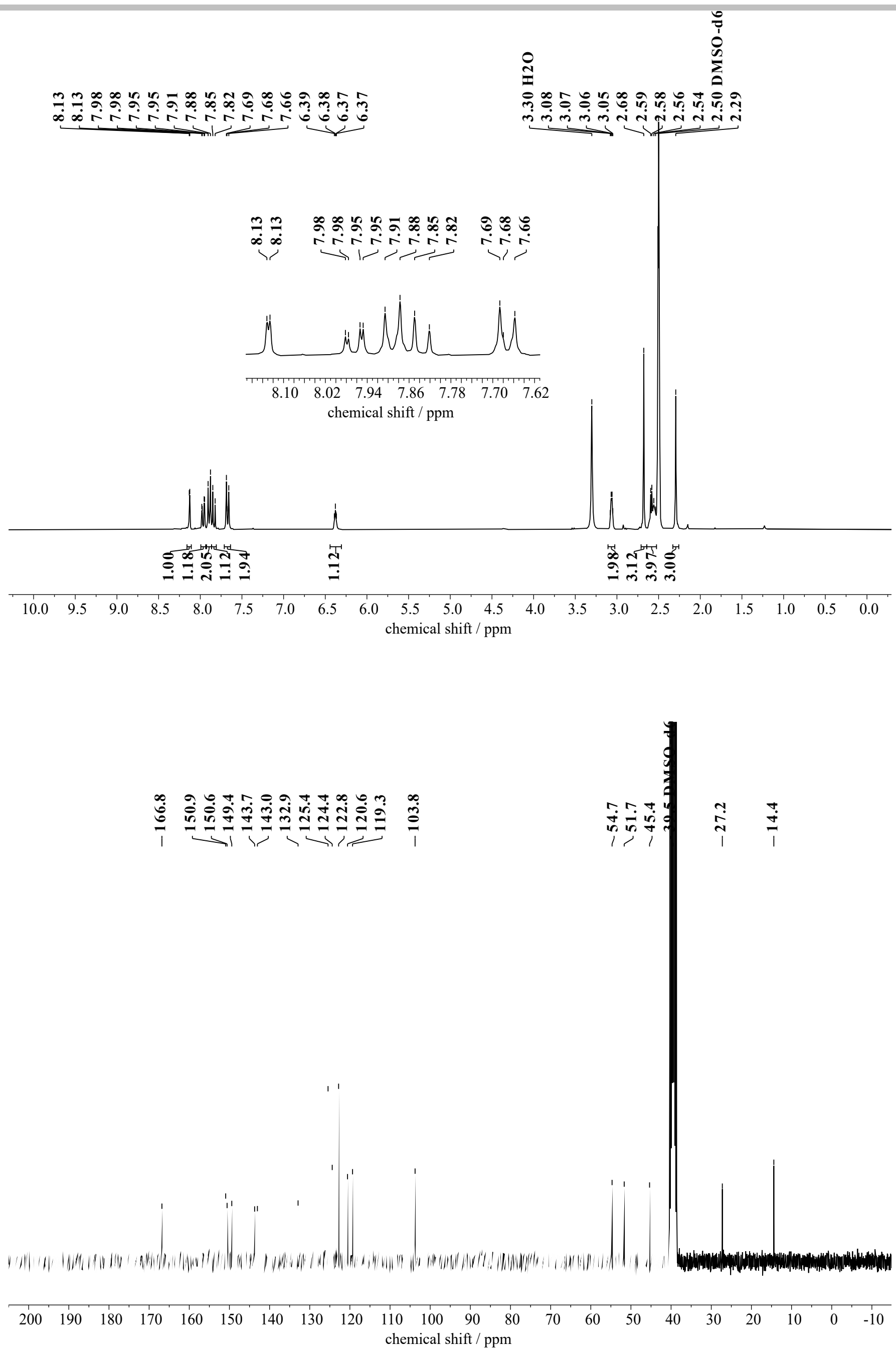


**Figure S19**. $^{1}H$ (300 MHz) and $^{13}C$ (75 MHz) NMR spectra of **6** in DMSO-$d_6$.

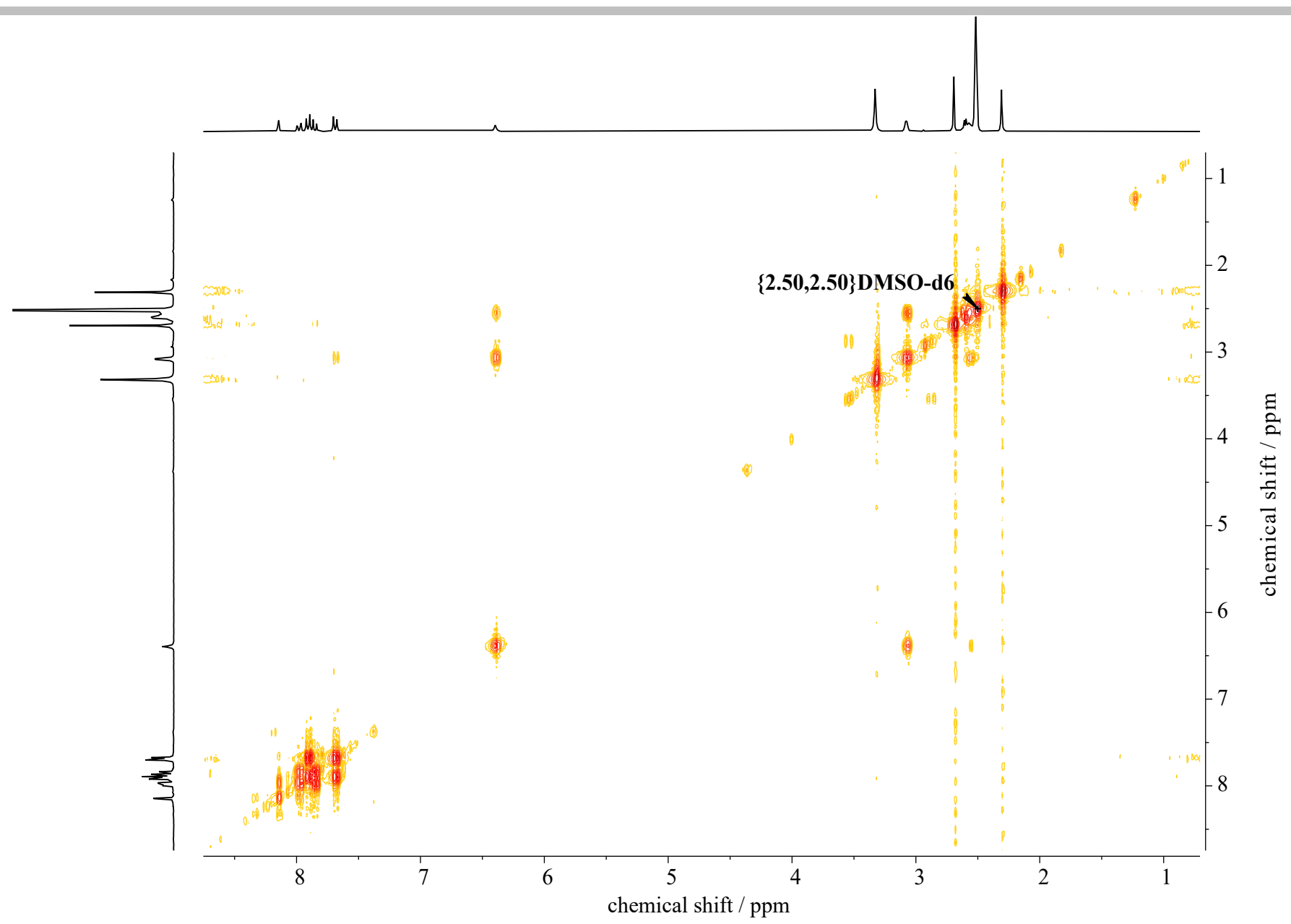


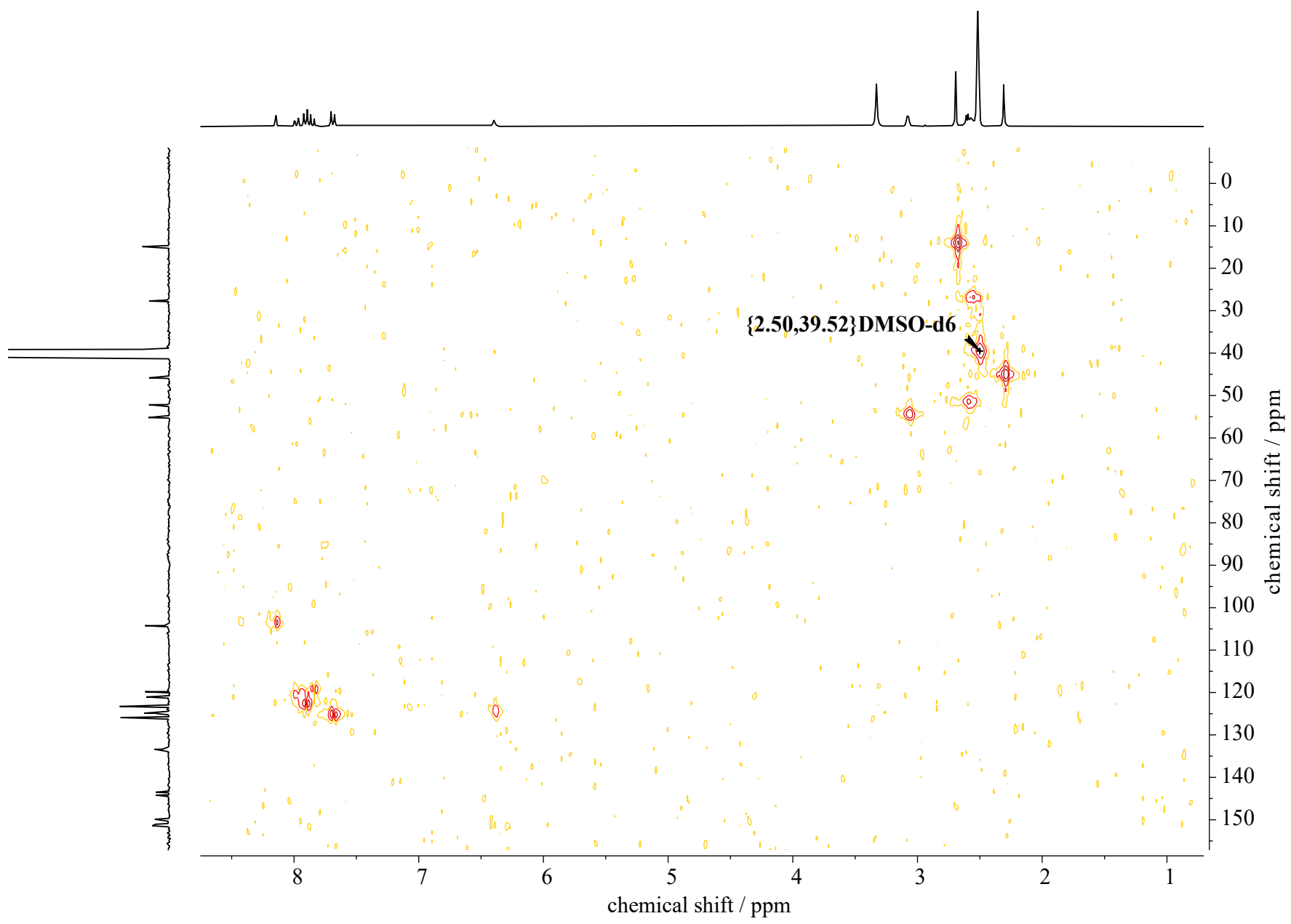


**Figure S20**. COSY and HMQC NMR spectra of **6** in DMSO-$d_6$.

**Synthesis of 6-((4-bromo-2-fluorophenyl)diazenyl)-2-methylbenzo[d]thiazole (23)**

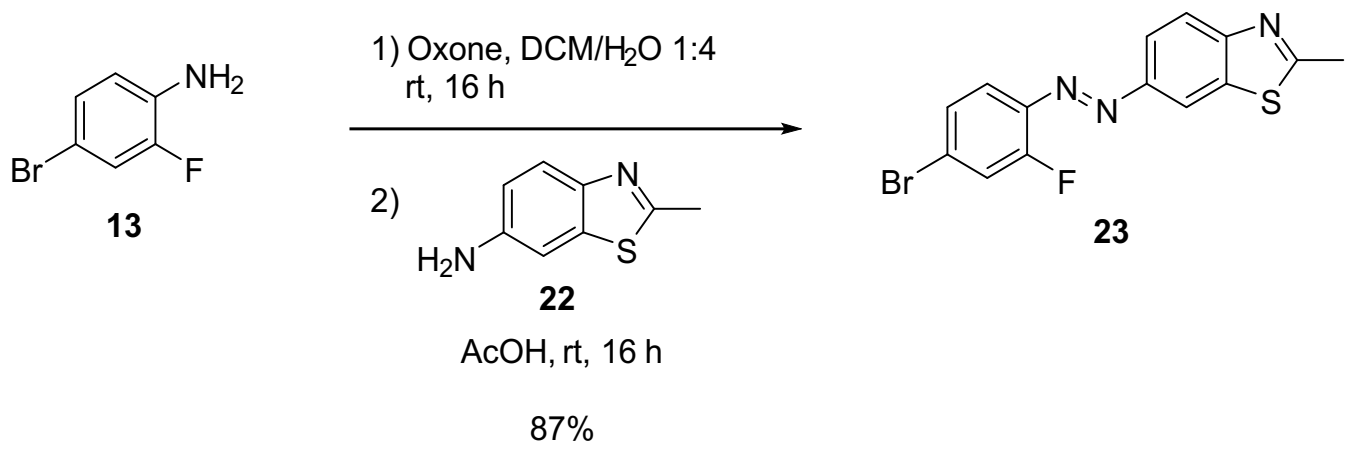


4-Bromo-2-fluoroaniline **13** (463 mg, 2.44 mmol, 2.00 eq) was dissolved in 5 mL DCM, Oxone® (1.50 g, 4.87 mmol, 4.00 eq) was dissolved in 20 mL distilled water and added to the solution. The resulting mixture was stirred for 16 h at room temperature. The reaction mixture was then diluted with 100 mL DCM and washed with 100 mL 1 M HCl, distilled water and brine. The organic layer was dried over $MgSO_4$ and the solvent was removed under reduced pressure. The resulting brown solid and 2-methyl-1,3-benzothiazol-6-amine **22** (200 mg, 1.22 mmol, 1.00 eq) were suspended in 20 mL acetic acid. The reaction mixture was stirred for 16 h at room temperature. The reaction mixture was then diluted with 200 mL distilled water and extracted with 200 mL EtOAc. The organic layer was washed with 200 mL distilled water and brine, dried over $MgSO_4$ and solvent was removed under reduced pressure. The crude product was purified by flash column chromatography (DCM) to yield the desired compound 6-((4-bromo-2-fluorophenyl)diazenyl)-2-methylbenzo[*d*]thiazole (**23**, 373 mg, 1.07 mmol, 87%) as an orange solid.
**TLC:** $R_f$ = 0.30 (DCM).
**$^1$H-NMR (300 MHz, DMSO-*d*$_6$, *δ*):** 8.66 (d, $^4J$ = 1.9 Hz, 1H, $H_{arom.}$), 8.08 (d, $^3J$ = 8.7 Hz, 1H, $H_{arom.}$), 8.03 (dd, $^3J$ = 8.7 Hz, $^4J$ = 1.9 Hz, 1H, $H_{arom.}$), 7.90 (dd, $^3J$ = 10.4 Hz, $^4J$ = 2.0 Hz, 1H, $H_{arom.}$), 7.71 (t, $^3J$ = 8.4 Hz, 1H, $H_{arom.}$), 7.62-7.58 (m, 1H, $H_{arom.}$), 2.86 (s, 3H, C$H_3$) ppm.
**$^{13}$C-NMR (75 MHz, DMSO-*d*$_6$, *δ*):** 171.4 (N=*C*-S), 159.0 (d, $^1J_{FC}$ = 260.5 Hz, *C*F), 155.4 ($C_{arom.}$), 148.9 ($C_{arom.}$), 139.0 (d, $^2J_{FC}$ = 7.3 Hz, $C_{arom.}$), 136.4 ($C_{arom.}$), 128.5 (d, $^3J_{FC}$ = 4.0 Hz, $C_{arom.}$H), 125.3 (d, $^3J_{FC}$ = 9.6 Hz, $C_{arom.}$H), 122.6 ($C_{arom.}$), 120.8 (d, $^2J_{FC}$ = 23.2 Hz, $C_{arom.}$H), 120.5 ($C_{arom.}$H), 119.0 ($C_{arom.}$H), 118.3 ($C_{arom.}$H), 20.1 ($CH_3$) ppm.
**$^{19}$F-NMR (282 MHz, DMSO-*d*$_6$, *δ*)**: -121.9 (C*F*) ppm.
**HRMS-ESI (m/z):** $[M+H]^+$ calcd for $C_{14}H_{10}BrFN_3S$, 351.9737; found, 351.9743.

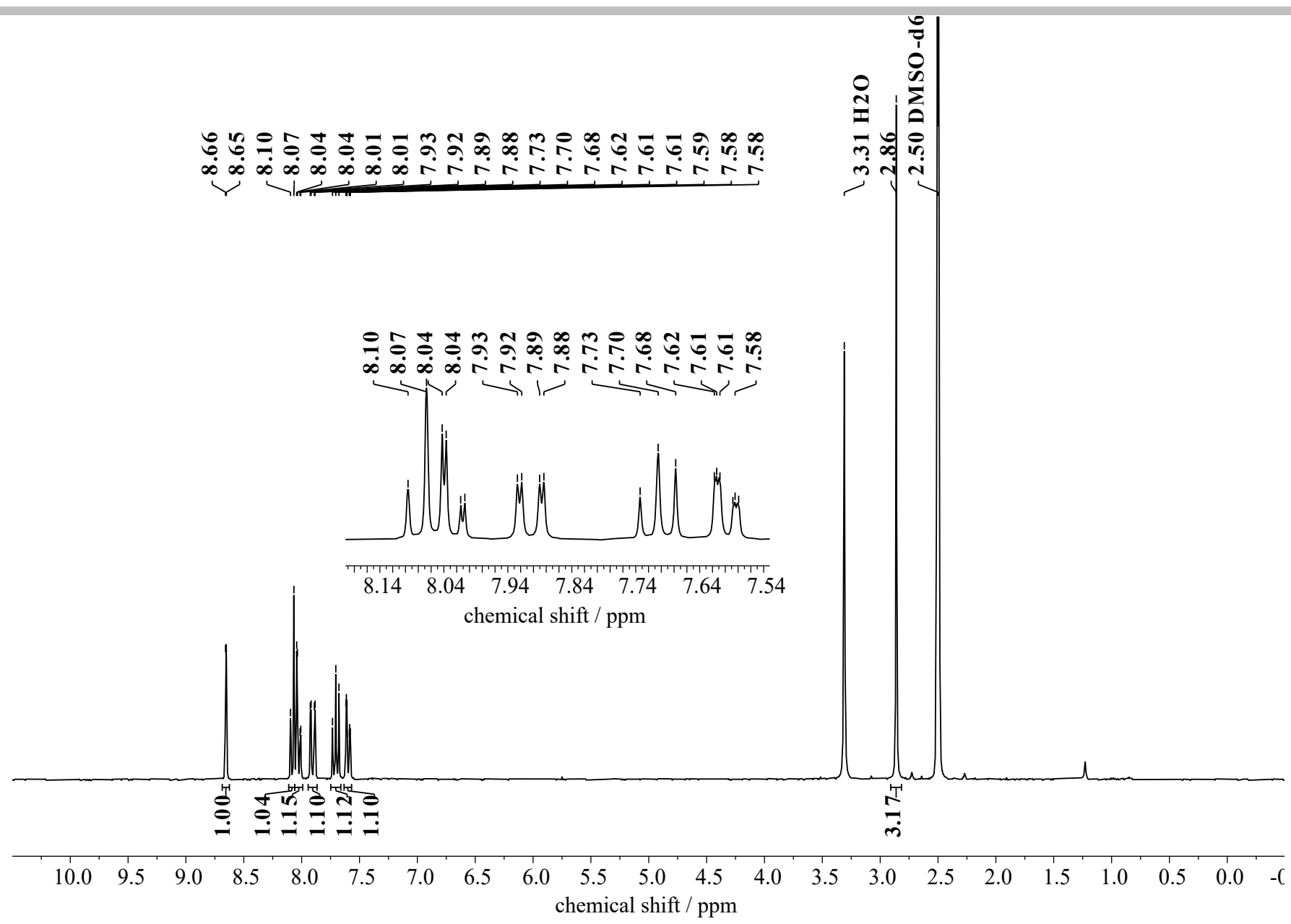


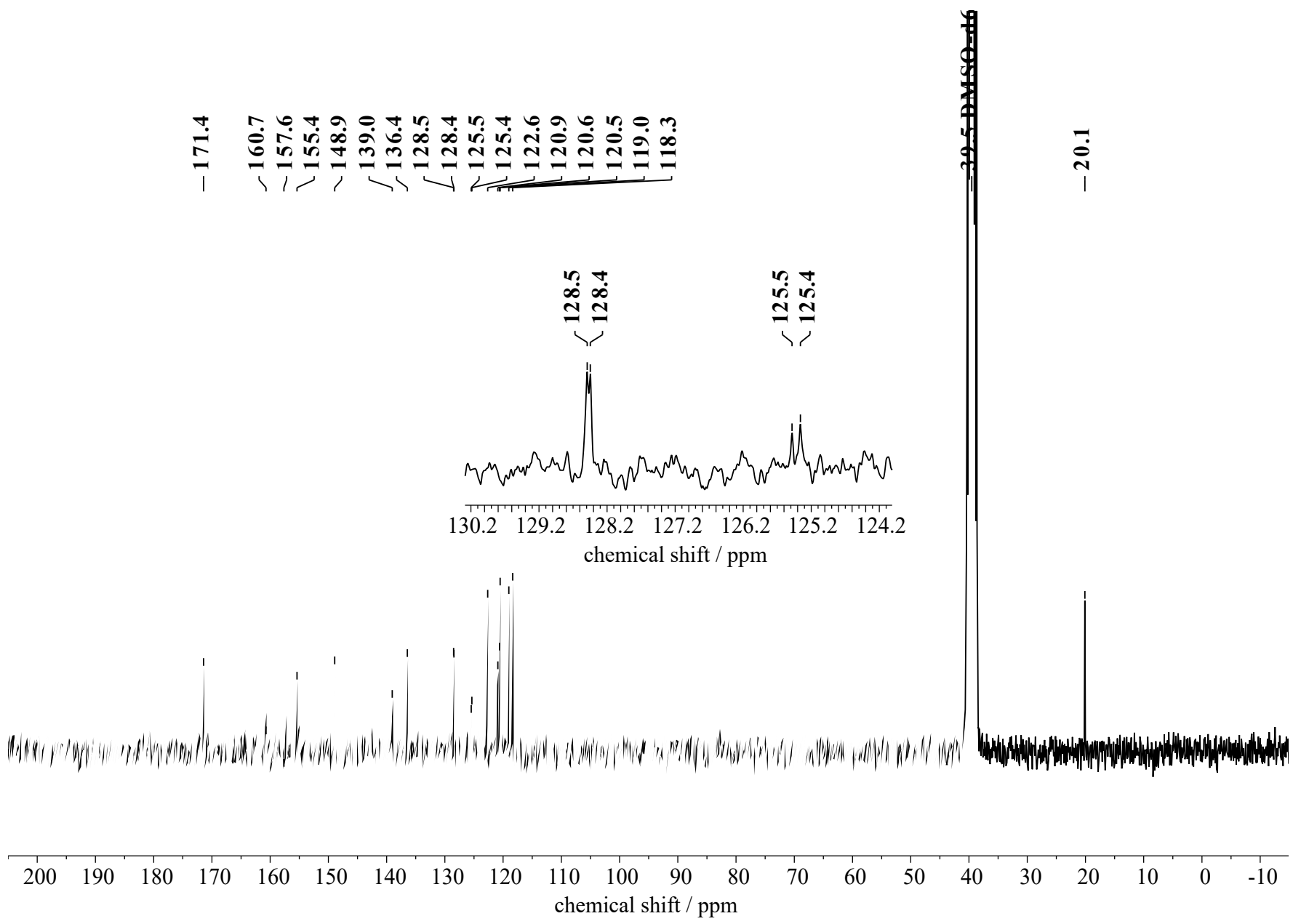


**Figure S21**. $^{1}$H (300 MHz) and $^{13}$C (75 MHz) NMR spectra of **23** in DMSO-$d_6$.

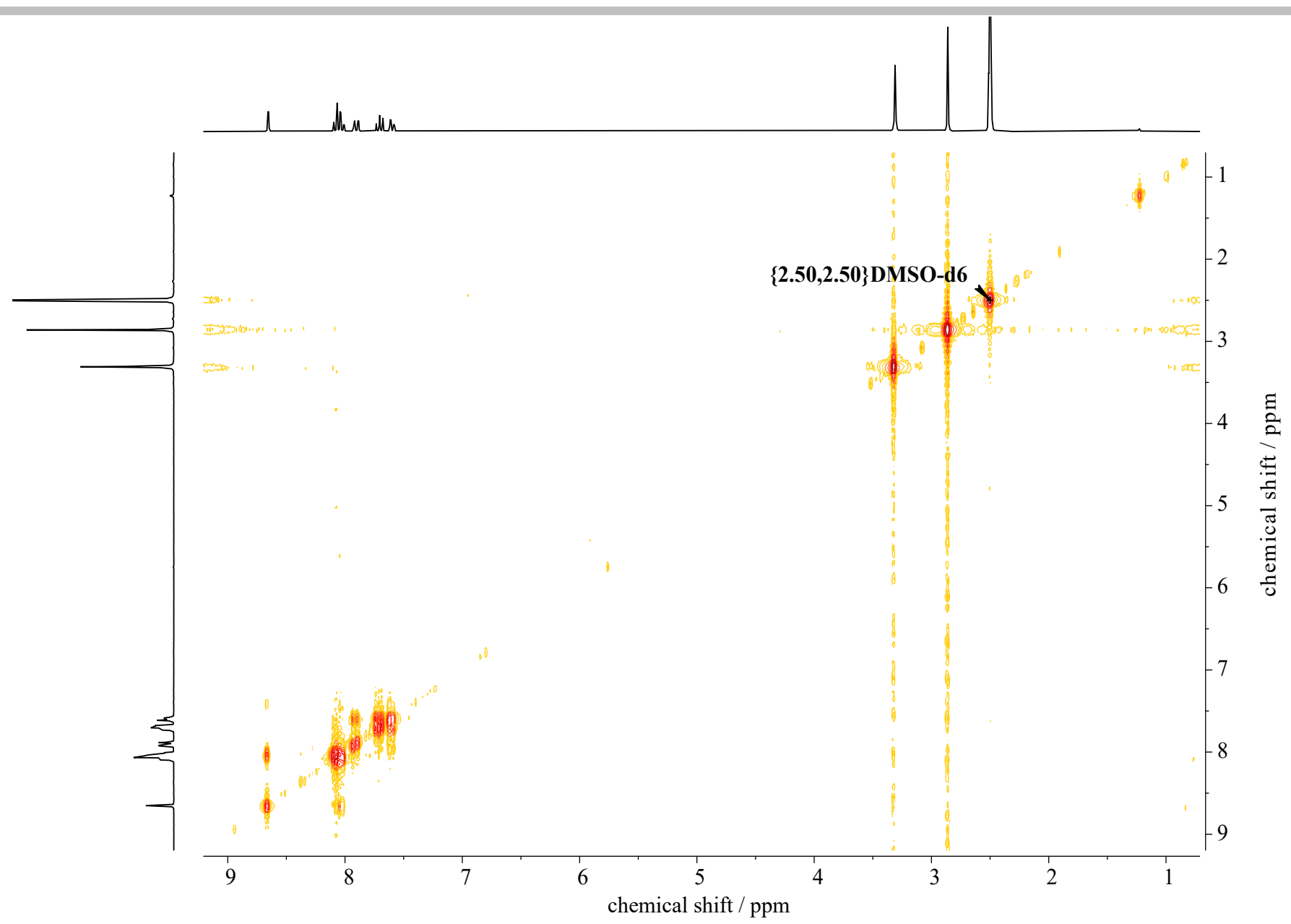


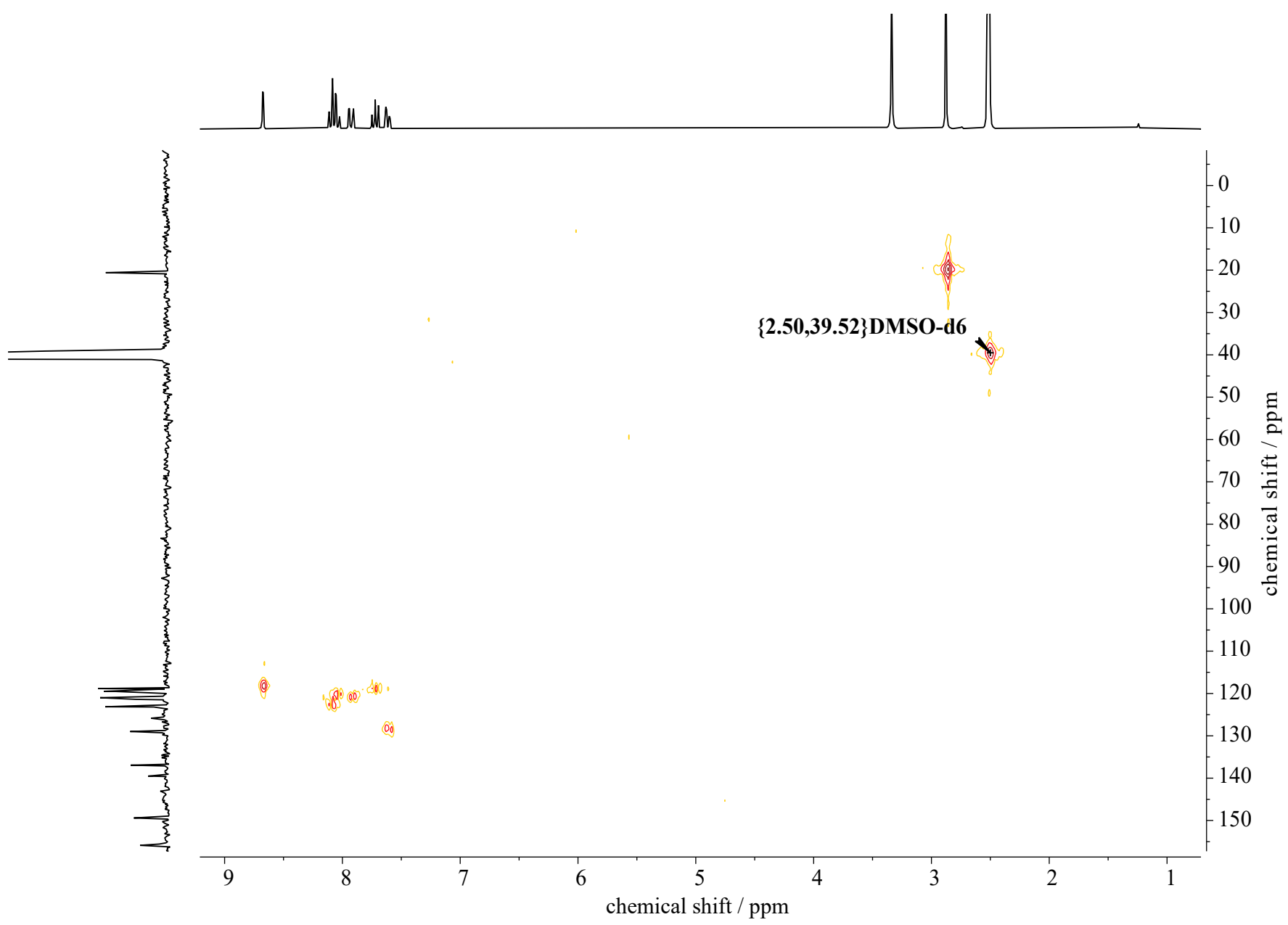


**Figure S22**. COSY and HMQC NMR spectra of **23** in DMSO-$d_6$.

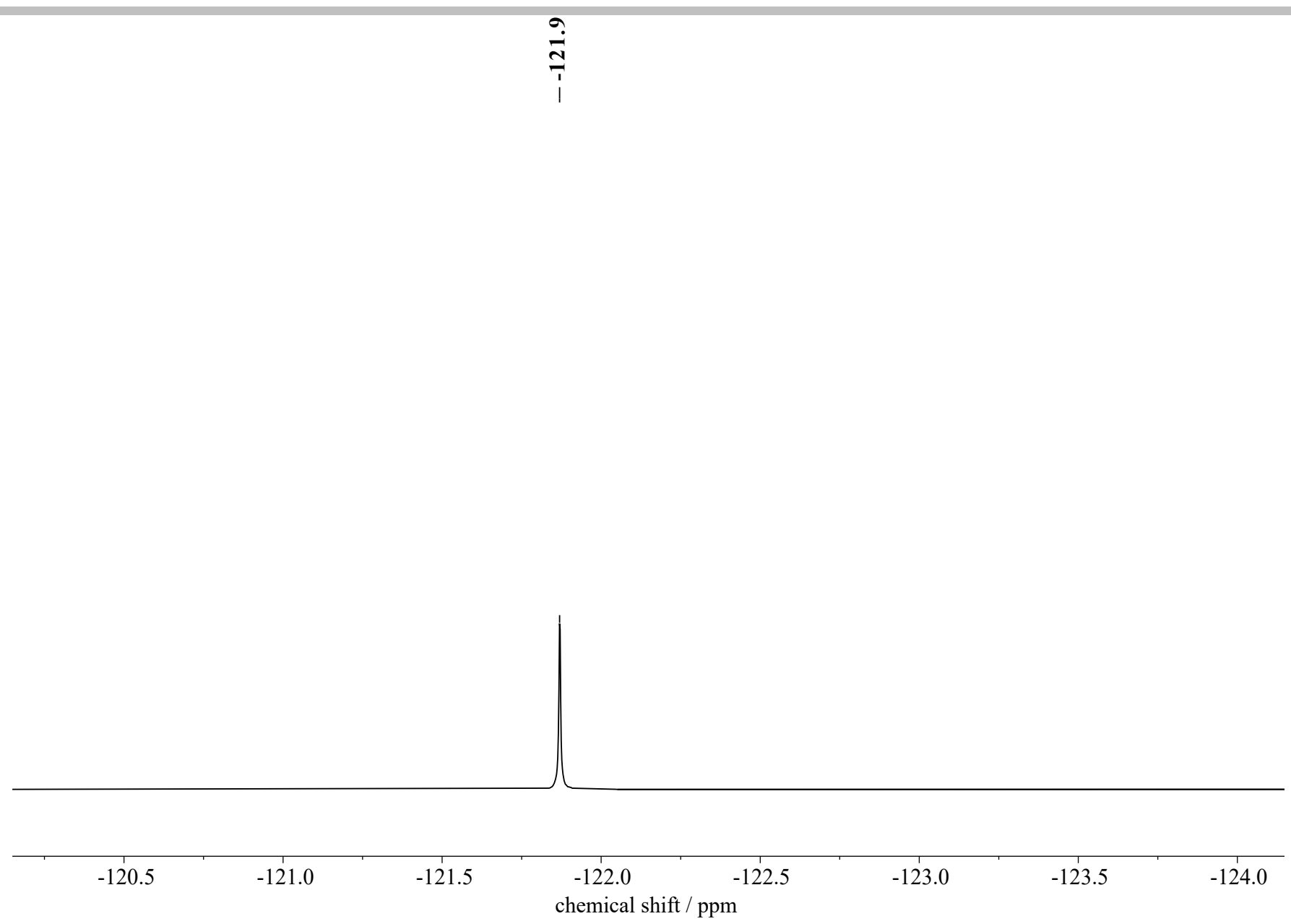


**Figure S23**. $^{19}F$ (282 MHz) NMR spectra of **23** in DMSO-$d_6$.

**Synthesis of tert-butyl-4-(3-fluoro-4-((2-methylbenzo[d]thiazol-6-yl)diazenyl)phenyl)-3,6-dihydropyridine-1(2H)-carboxylate (24)**

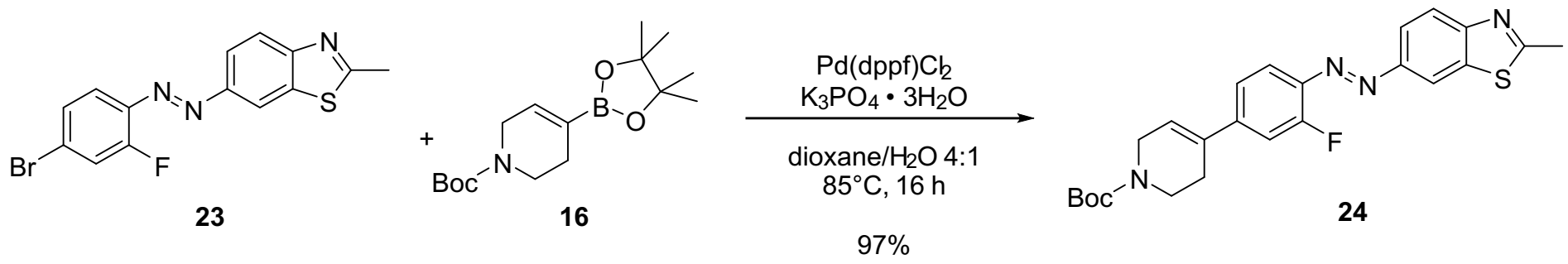


6-((4-Bromo-2-fluorophenyl)diazenyl)-2-methylbenzo[*d*]thiazole **23** (100 mg, 286 µmol, 1.00 eq), *N-tert*-butoxycarbony-1,2,3,6-tetrahydropyridine-4-boronic acid pinacol ester **16** (88 mg, 286 µmol, 1.00 eq), Pd(dppf)$Cl_2$ (21 mg, 28.6 µmol, 0.10 eq) and $K_3PO_4 \cdot 3H_2O$ (228 mg, 857 µmol, 3.00 eq) were dissolved in mixture of dioxane/distilled water (20 mL, 4:1, v%). The reaction was stirred under $N_2$ atmosphere at 85 °C for 16 h. The reaction was then diluted with 100 mL distilled water and extracted with 100 mL EtOAc. The organic layer was washed with 100 mL distilled water and brine, dried over $MgSO_4$ and solvent was removed under reduced pressure. The crude product was purified by flash column chromatography (EtOAc) to yield the desired compound *tert*-butyl-4-(3-fluoro-4-((2-methylbenzo[*d*]thiazol-6-yl)diazenyl)phenyl)-3,6-dihydropyridine-1(*2H*)-carboxylate (**24**, 125 mg, 276 µmol, 97%) as an orange solid.

**TLC:** $R_f$ = 0.79 (EtOAc).

**$^1$H-NMR (300 MHz, DMSO-*d*6, *δ*):** 8.63 (d, $^4J$ = 1.7 Hz, 1H, $H_{arom.}$), 8.08 (d, $^3J$ = 8.7 Hz, 1H, $H_{arom.}$), 8.02 (dd, $^3J$ = 8.7 Hz, $^4J$ = 1.9 Hz, 1H, $H_{arom.}$), 7.76 (t, $^3J$ = 8.3 Hz, 1H, $H_{arom.}$), 7.59 (dd, $^3J$ = 12.7 Hz, $^4J$ = 1.8 Hz, 1H, $H_{arom.}$), 7.47 (dd, $^3J$ = 8.6 Hz, $^4J$ = 1.9 Hz, 1H, $H_{arom.}$), 6.46 (s, 1H, C*H*=C), 4.06-4.05 (m, 2H, $CH_2$), 3.56 (t, $^3J$ = 5.7 Hz, 2H, $CH_2$), 2.86 (s, 3H, $CH_3$), 2.54-2.50 (m, 2H, $CH_2$), 1.44 (s, 9H, 3x$CH_3$) ppm.

**$^{13}$C-NMR (75 MHz, DMSO-*d*6, *δ*):** 170.8 (N=*C*-S), 155.1 (*C*F), 153.8 ($C_{arom.}$), 149.1 ($C_{arom.}$), 145.2 (d, $^3J_{FC}$ = 7.7 Hz, $C_{arom.}$), 136.3 ($C_{arom.}$), 133.2 ($C_{q.}$), 124.8 (*C*H=C), 122.6 ($C_{arom.}$H), 121.2 ($C_{arom.}$H), 120.5 ($C_{arom.}$H), 118.0 ($C_{arom.}$H), 117.4 ($C_{arom.}$H), 113.1 (d, $^2J_{FC}$ = 20.8 Hz, $C_{arom.}$H), 106.9 ($C_{arom.}$), 77.6 ($C_{q.}$), 43.4($CH_2$), 28.1 (3C, 3x$CH_3$), 26.3 ($CH_2$), 20.1 ($CH_3$) ppm.

**$^{19}$F-NMR (282 MHz, DMSO-*d*6, *δ*)**: -124.2 (C*F*) ppm.

**HRMS-ESI (m/z):** $[M+H]^+$ calcd for $C_{24}H_{26}FN_4O_2S$, 453.1755; found, 453.1785.

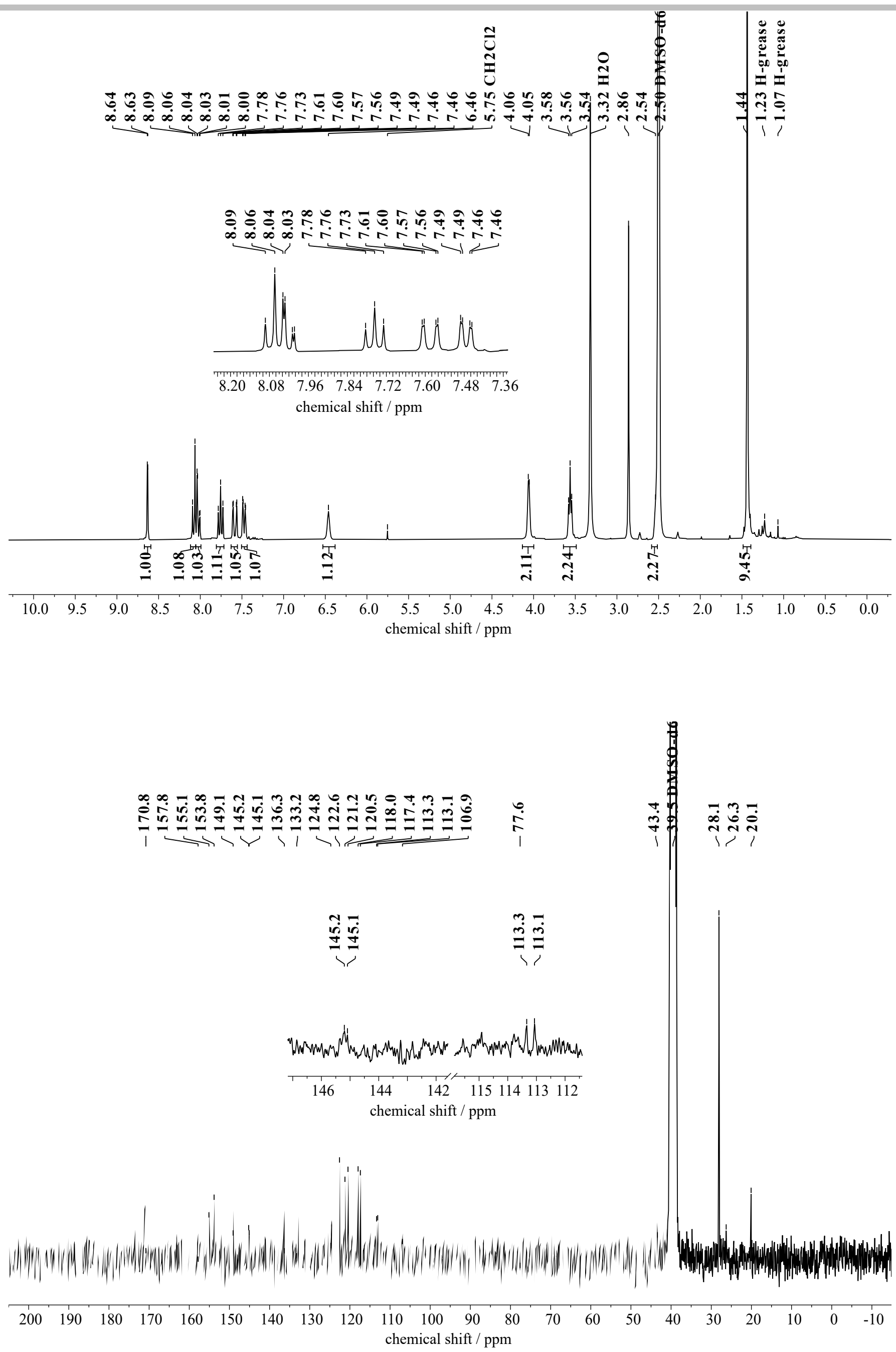


**Figure S24**. $^{1}H$ (300 MHz) and $^{13}C$ (75 MHz) NMR spectra of **24** in DMSO-$d_6$.

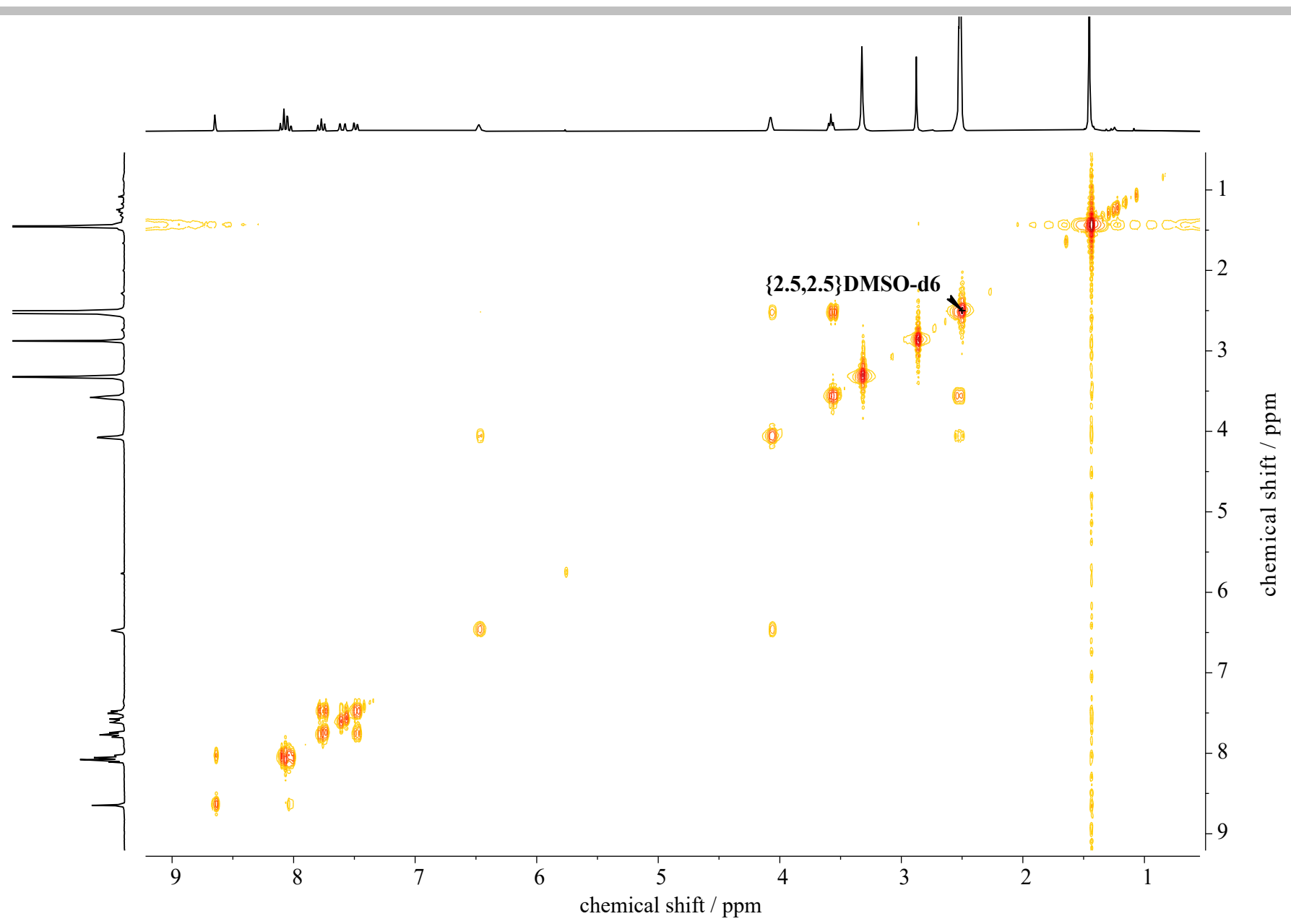


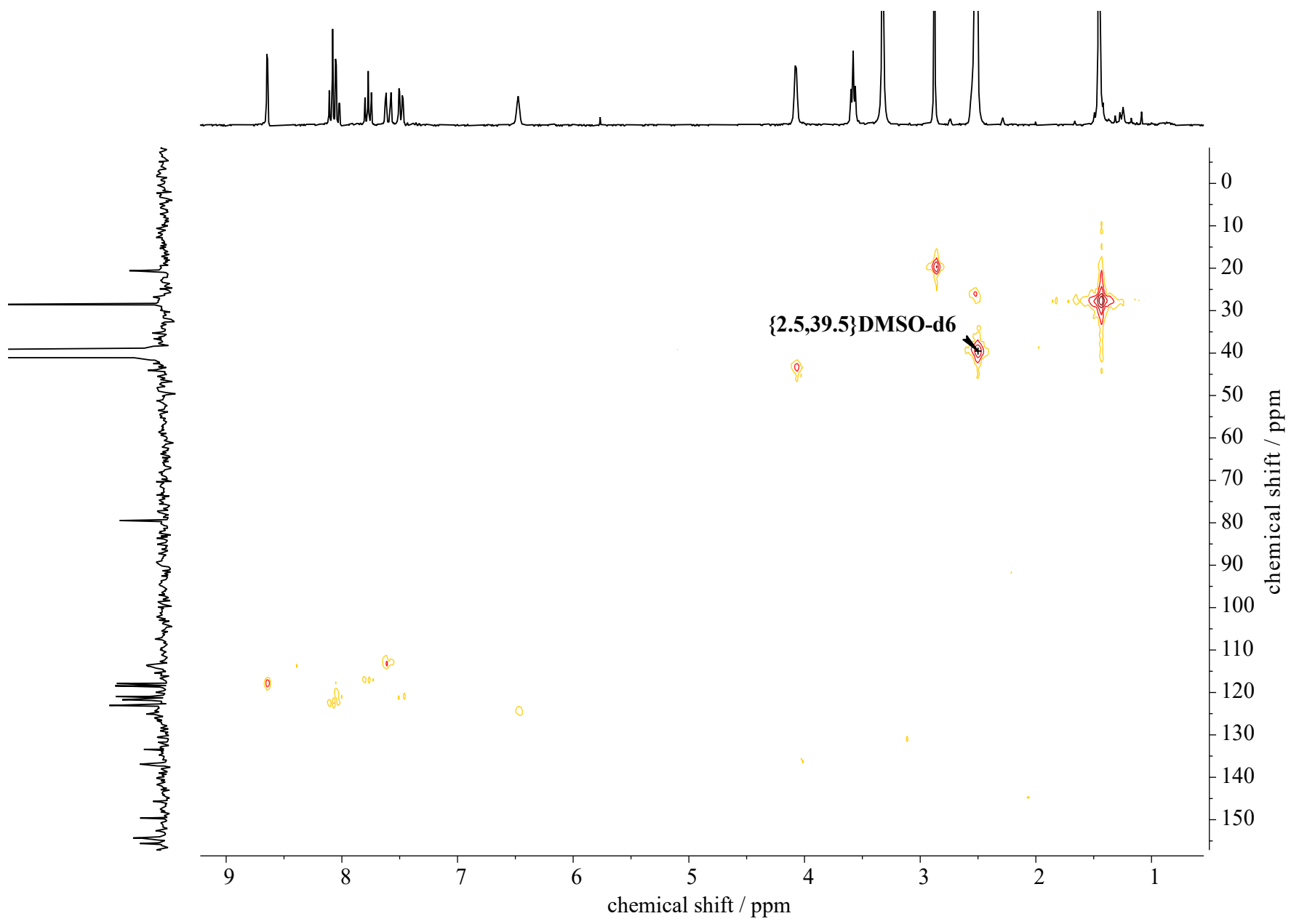


**Figure S25**. COSY and HMQC NMR spectra of **24** in DMSO-$d_6$.

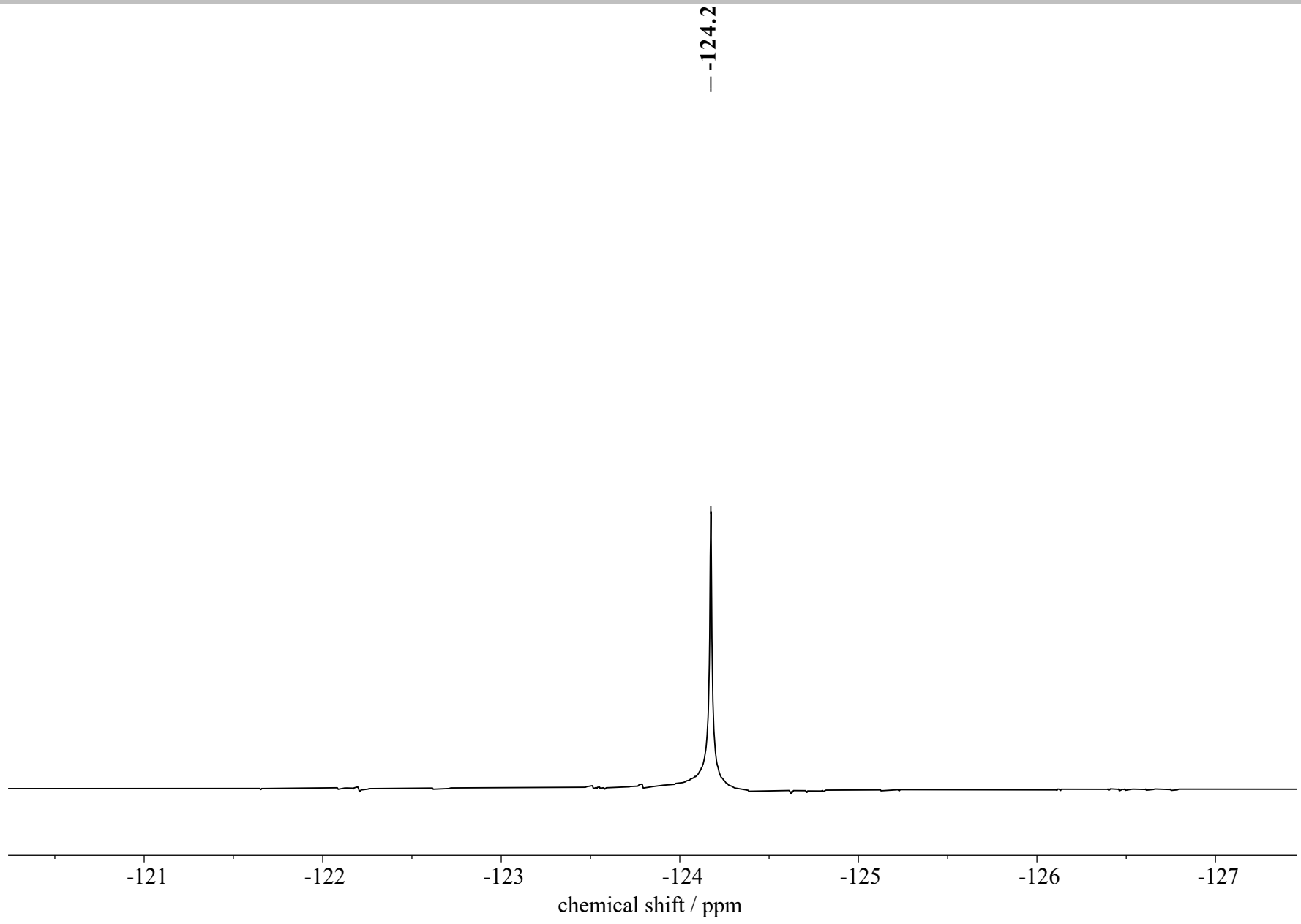


**Figure S26**. $^{19}F$ (282 MHz) NMR spectra of **24** in DMSO-$d_6$.

**Synthesis of 4-(3-fluoro-4-((2-methylbenzo[d]thiazol-6-yl)diazenyl)phenyl)-1,2,3,6-tetrahydropyridin (4)**

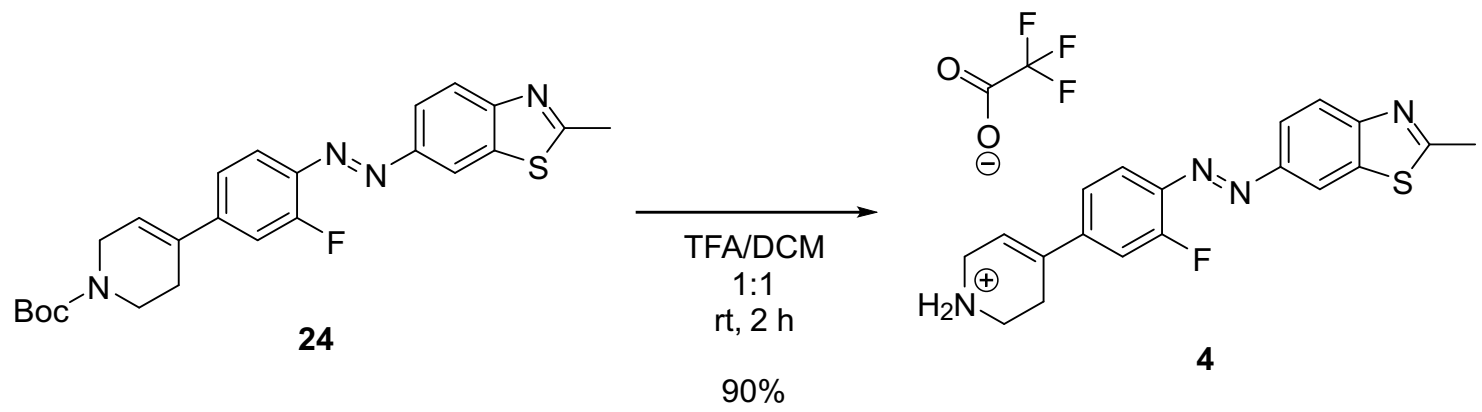


*Tert*-butyl-4-(3-fluoro-4-((2-methylbenzo[*d*]thiazol-6-yl)diazenyl)phenyl)-3,6-dihydropyridine-1(*2H*)-carboxylate **24** (50 mg, 110 µmol, 1.00 eq) was dissolved in mixture of TFA/DCM (10 mL, 1:1, v%). The reaction was stirred at room temperature for 2 h. The reaction was then diluted with 100 mL distilled water and extracted with 100 mL EtOAc. The organic layer was washed with 100 mL distilled water and brine, dried over $MgSO_4$ and solvent was removed under reduced pressure. The crude product was purified by flash column chromatography (EtOAc/MeOH, 1:1, v%) to yield the desired compound 4-(3-fluoro-4-((2-methylbenzo[*d*]thiazol-6-yl)diazenyl)phenyl)-1,2,3,6-tetrahydropyridin (**4**, 46 mg, 99.6 µmol, 90%) as an orange solid in form of a TFA salt.

**TLC:** $R_f$ = 0.25 (EtOAc/MeOH, 1:1, v%).

***trans*-isomer** (*cis*-isomer signals were labeled with * on NMR spectrum).

**$^1$H-NMR (300 MHz, DMSO-$d_6$, δ):** 8.93 (br, 2H, N$H_2$), 8.65 (d, $^4J$ = 1.9 Hz, 1H, $H_{arom.}$), 8.09 (d, $^3J$ = 8.7 Hz, 1H, $H_{arom.}$), 8.03 (dd, $^3J$ = 8.7 Hz, $^4J$ = 2.0 Hz, 1H, $H_{arom.}$), 7.79 (t, $^3J$ = 8.3 Hz, 1H, $H_{arom.}$), 7.67 (dd, $^3J$ = 12.6 Hz, $^4J$ = 1.9 Hz, 1H, $H_{arom.}$), 7.53 (dd, $^3J$ = 8.6 Hz, $^4J$ = 1.9 Hz, 1H, $H_{arom.}$), 6.51-6.49 (m, 1H, C*H*=C), 3.84 (s, 2H, C$H_2$), 3.39-3.36 (m, 2H, C$H_2$), 2.86 (s, 3H, C$H_3$), 2.77-2.72 (m, 2H, C$H_2$) ppm.

**$^{13}$C-NMR (75 MHz, DMSO-$d_6$, δ):** 171.2 (N=*C*-S), 159.5 (d, $^1J_{FC}$ = 254.7 Hz, *C*F), 155.2 ($C_{arom.}$), 149.1 ($C_{arom.}$), 143.7 (d, $^2J_{FC}$ = 8.0 Hz, $C_{arom.}$), 138.9 (d, $^2J_{FC}$ = 7.8 Hz, $C_{arom.}$), 136.4 ($C_{q.}$), 132.7 (d, $^3J_{FC}$ = 2.7 Hz, $C_{arom.}$), 122.6 ($C_{arom.}$H), 121.4 (d, $^3J_{FC}$ = 3.2 Hz, $C_{arom.}$H), 120.4 (*C*H=C), 120.2 ($C_{arom.}$H), 118.1 ($C_{arom.}$H), 117.1 ($C_{arom.}$H), 113.5 (d, $^2J_{FC}$ = 20.9 Hz, $C_{arom.}$H), 41.8 ($CH_2$), 22.9 ($CH_2$), 20.1 ($CH_3$) ppm.

**$^{19}$F-NMR (282 MHz, DMSO-$d_6$, δ)**: -124.0 (C*F*) ppm.

**HRMS-ESI (m/z):** $[M+H]^+$ calcd for $C_{19}H_{18}FN_4S$, 353.1239; found, 353.1231.

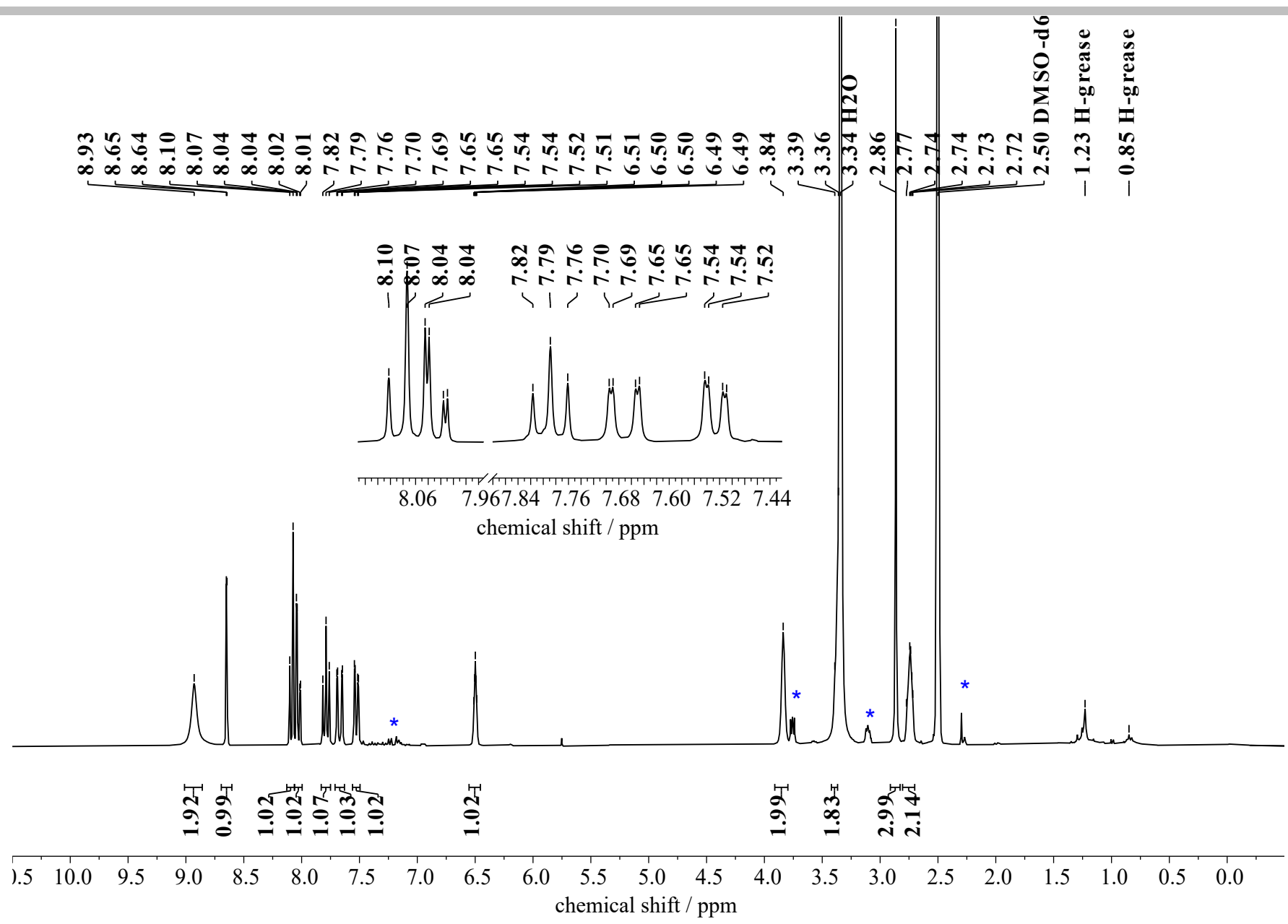

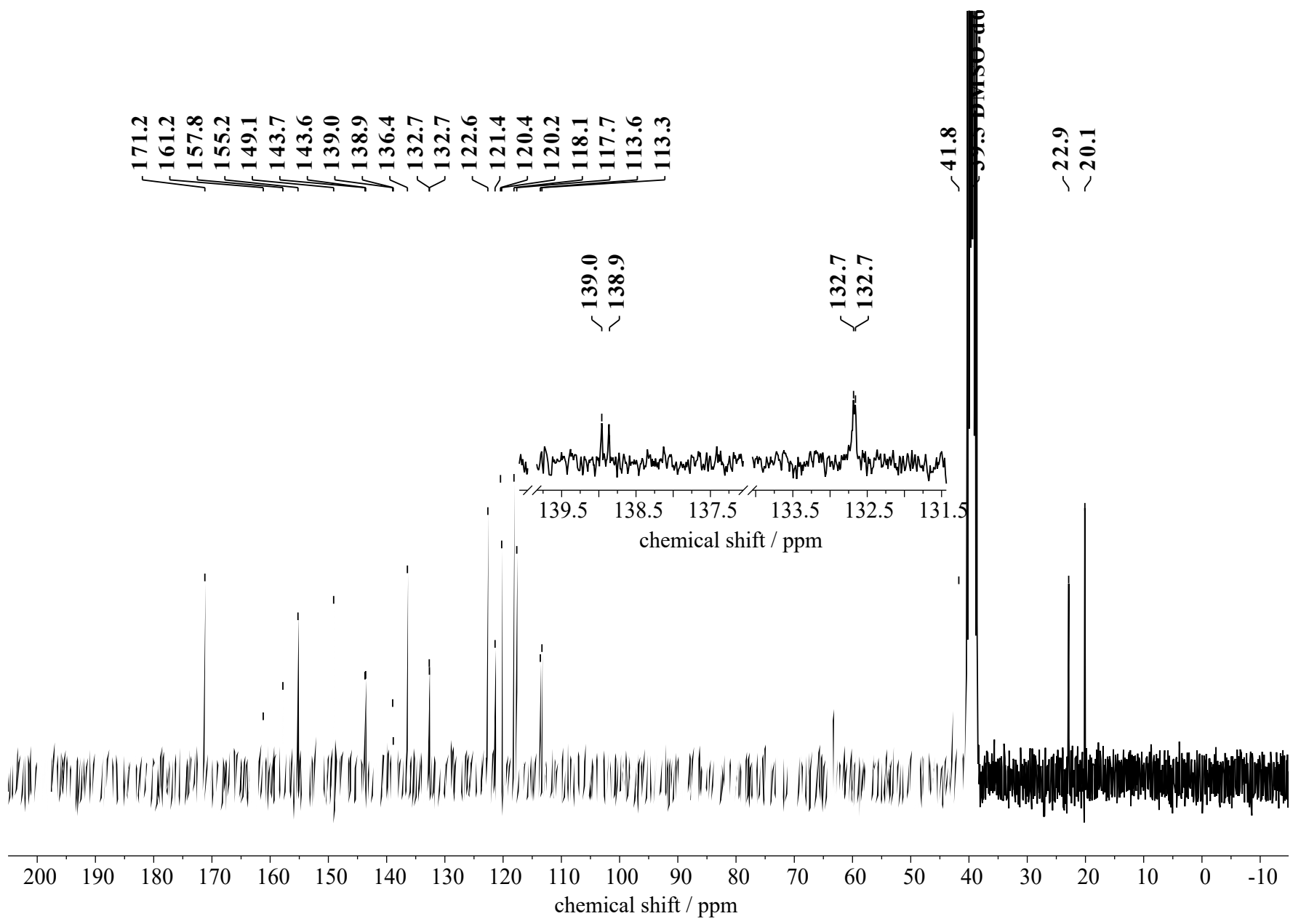

**Figure S27**. $^{1}$H (300 MHz) and $^{13}$C (75 MHz) NMR spectra of **4** in DMSO-$d_6$.

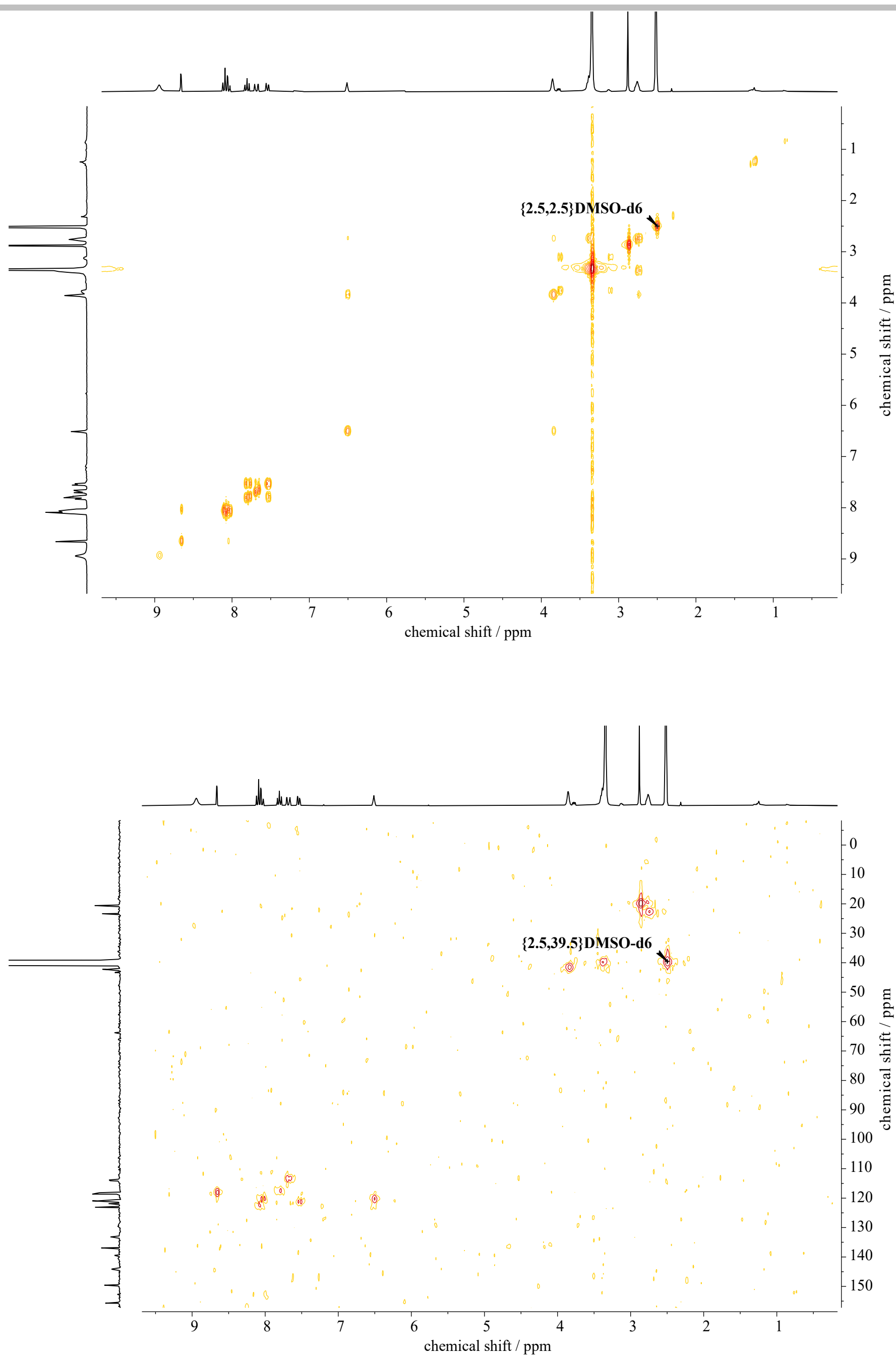


**Figure S28**. COSY and HMQC NMR spectra of **4** in DMSO-$d_6$.

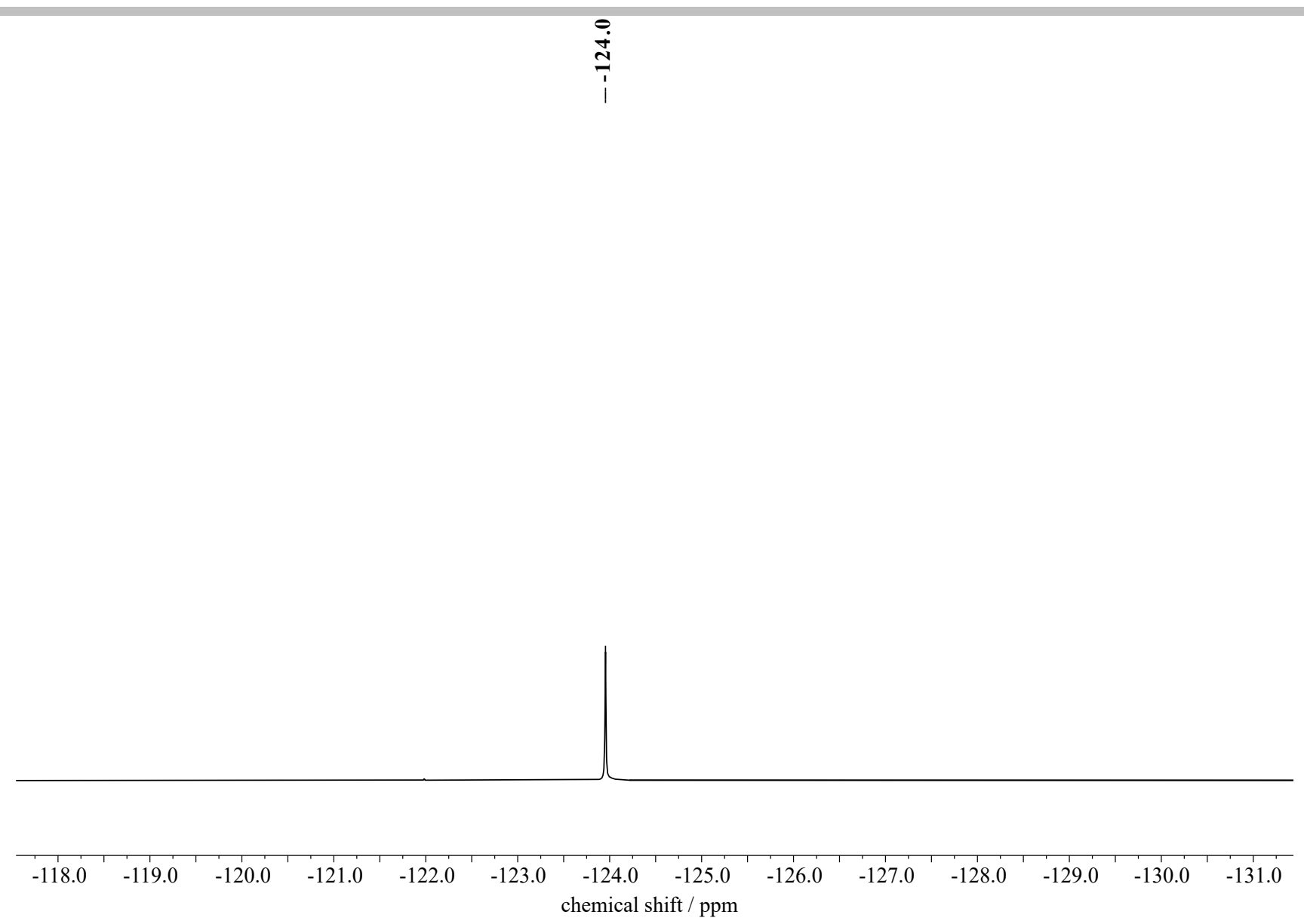


**Figure S29**. $^{19}$F (282 MHz) NMR spectra of **4** in DMSO-$d_6$.

**Synthesis of 4-((4-bromo-2-fluorophenyl)diazenyl)-2-methoxyphenol (26)**

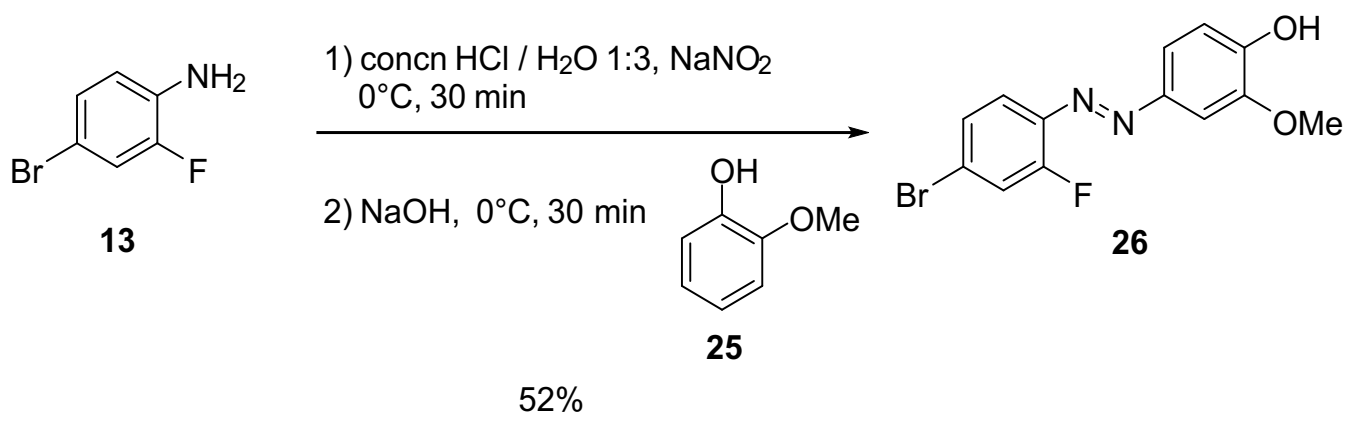

4-Bromo-2-fluoroaniline **13** (500 mg, 2.63 mmol, 1.00 eq) was dissolved in mixture of concn HCl/$H_2O$ (16 mL, 1:3, v%) at 0 °C. Sodium nitrite (236 mg, 3.42 mmol, 1.30 eq) was dissolved in 16 mL distilled water and added dropwise. The resulting mixture was stirred for 30 min at 0 °C. The pH value was then adjusted to 11 by adding 1 M aqueous NaOH. 2-Methoxyphenol **25** (425 mg, 3.42 mmol, 1.30 eq) was added and the mixture was stirred for further 30 min at 0 °C. The reaction mixture was then acidified to pH 1 with concn HCl and the orange precipitate was filtered and washed with distilled water. The crude product was purified by flash column chromatography (DCM) to yield the desired compound 4-((4-bromo-2-fluorophenyl)diazenyl)-2-methoxyphenol (**26**, 466 mg, 1.37 mmol, 52%) as an orange solid.

**TLC:** $R_f$ = 0.50 (DCM).

**$^1$H-NMR (300 MHz, DMSO-$d_6$, δ):** 10.18 (s, 1H, O*H*), 7.83 (dd, $^3J$ = 10.5 Hz, $^4J$ = 2.0 Hz, 1H, $H_{arom.}$), 7.63 (t, $^3J$ = 8.4 Hz, 1H, $H_{arom.}$), 7.56-7.51 (m, 2H, $H_{arom.}$, $H_{arom.}$), 7.44 (d, $^4J$ = 2.2 Hz, 1H, $H_{arom.}$), 6.99 (d, $^3J$ = 8.4 Hz, 1H, $H_{arom.}$), 3.86 (s, 3H, C$H_3$) ppm.

**$^{13}$C-NMR (75 MHz, DMSO-$d_6$, δ):** 152.1 ($C_{arom.}$), 148.5 ($C_{arom.}$), 145.2 ($C_{arom.}$), 139.3 ($C_{arom.}$), 131.4 ($C_{arom.}$), 128.3 (d, $^3J_{FC}$ = 4.1 Hz, $C_{arom.}$H), 123.7 (d, $^3J_{FC}$ = 9.8 Hz, $C_{arom.}$H), 121.3 ($C_{arom.}$H), 120.3 (d, $^2J_{FC}$ = 22.9 Hz, $C_{arom.}$H), 119.0 ($C_{arom.}$H), 115.4 ($C_{arom.}$H), 103.3 ($C_{arom.}$H), 55.6 ($C$H$_3$) ppm.

**$^{19}$F-NMR (282 MHz, DMSO-$d_6$, δ)**: -122.6 (C*F*) ppm.

**HRMS-ESI (m/z):** $[M-H]^-$ calcd for $C_{13}H_9BrFN_2O_2$, 322.9839; found, 322.9837.

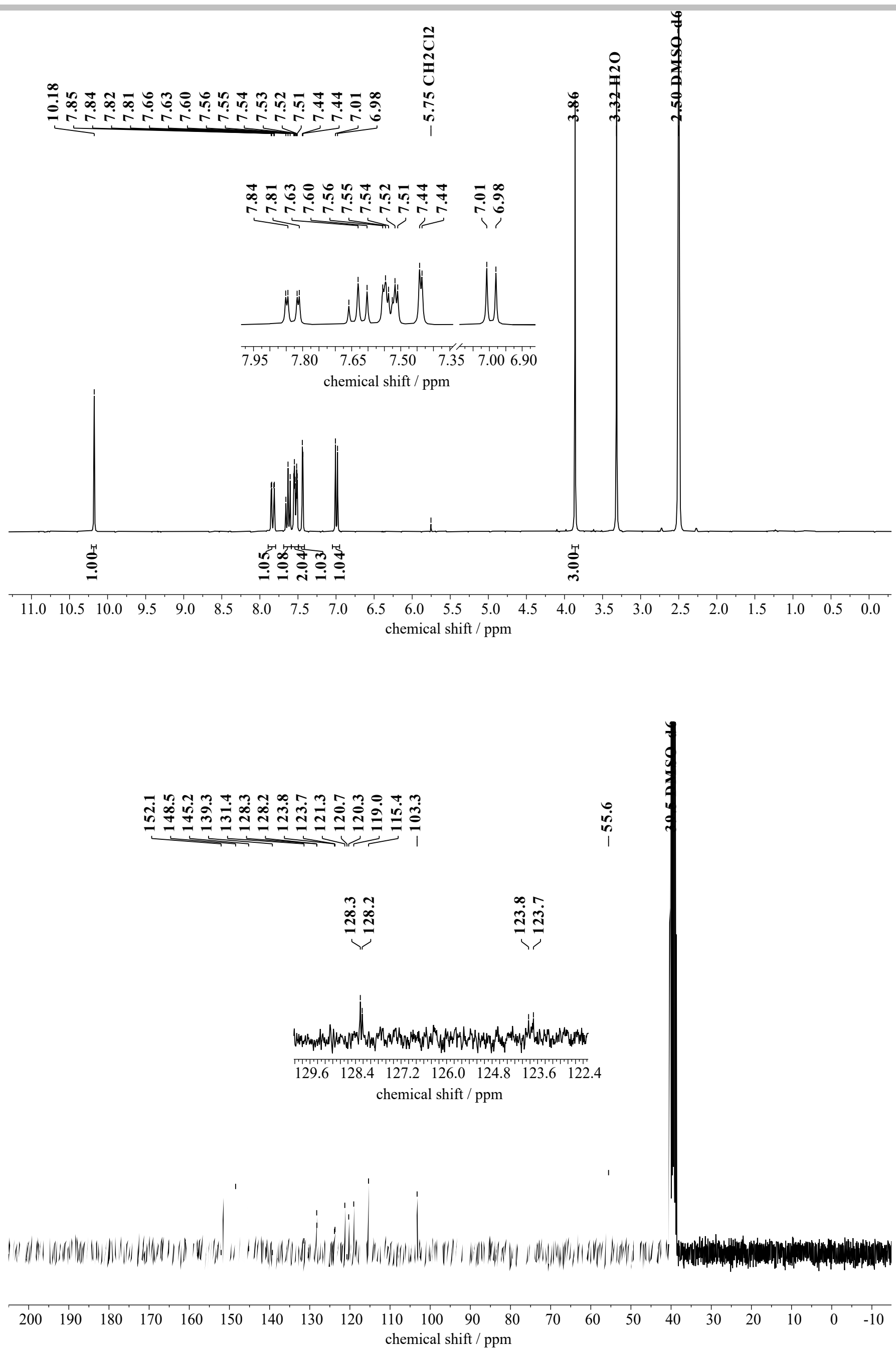


**Figure S30**. $^{1}$H (300 MHz) and $^{13}$C (75 MHz) NMR spectra of **26** in DMSO-$d_6$.

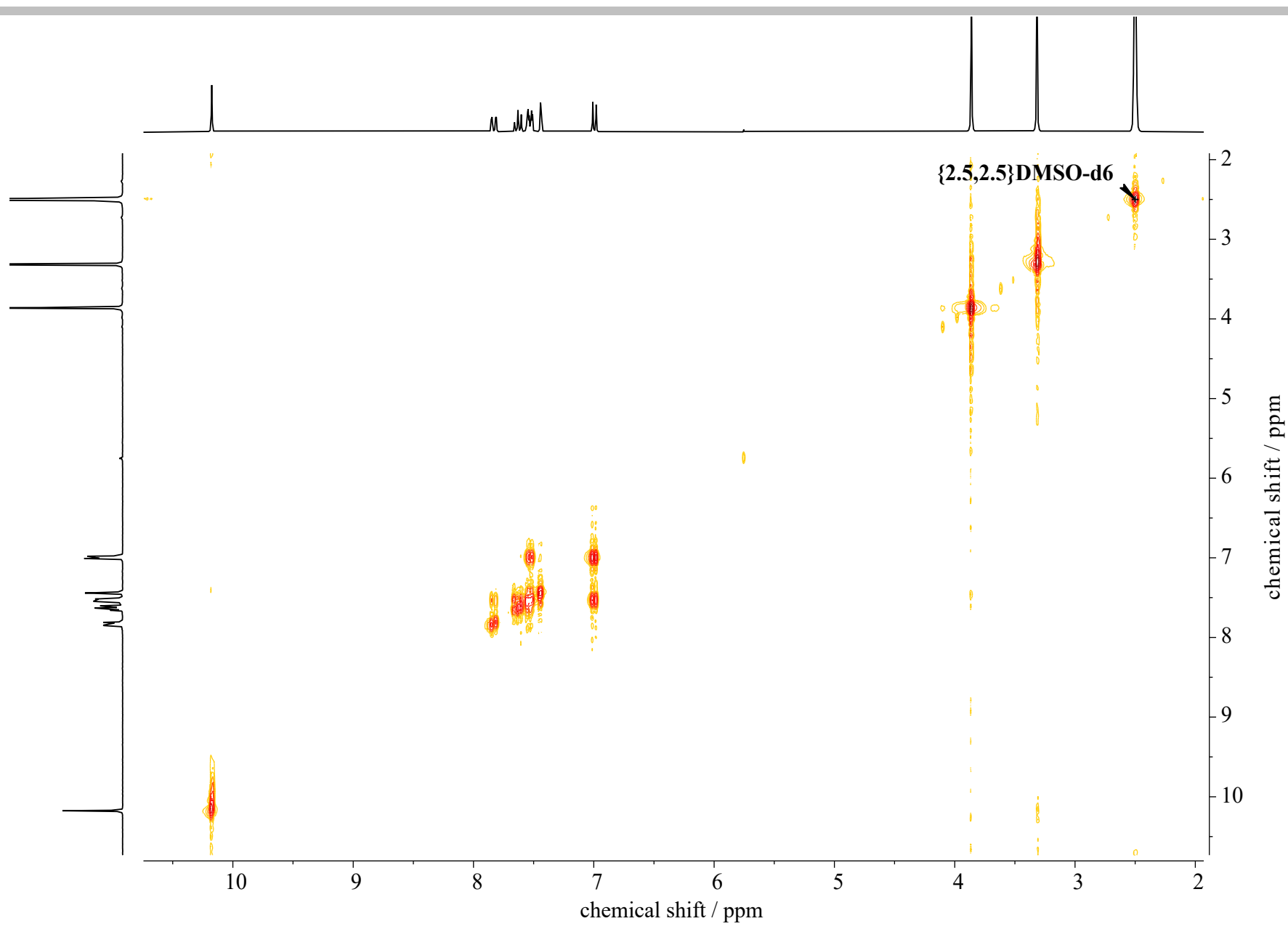


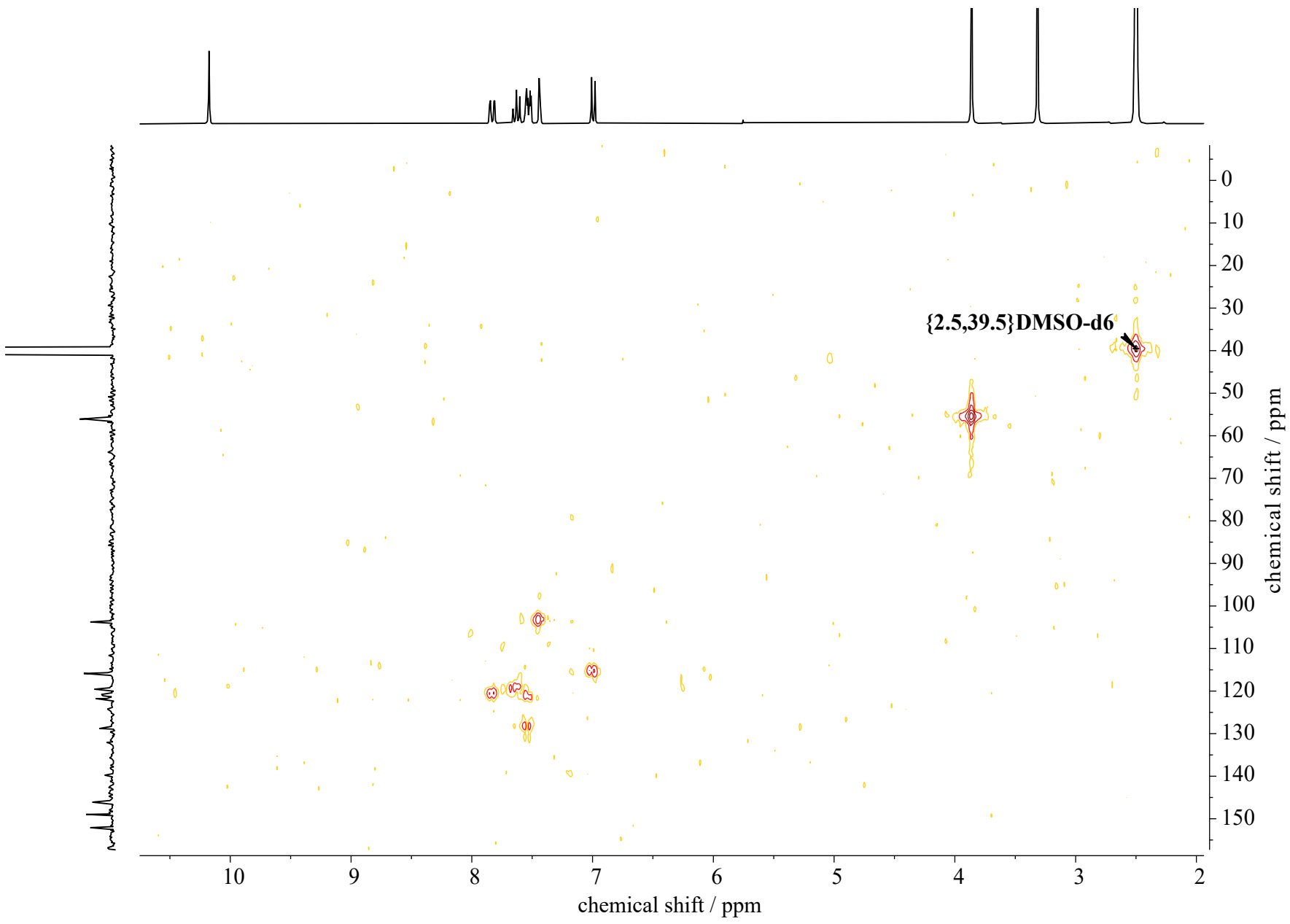


**Figure S31**. COSY and HMQC NMR spectra of **26** in DMSO-$d_6$.

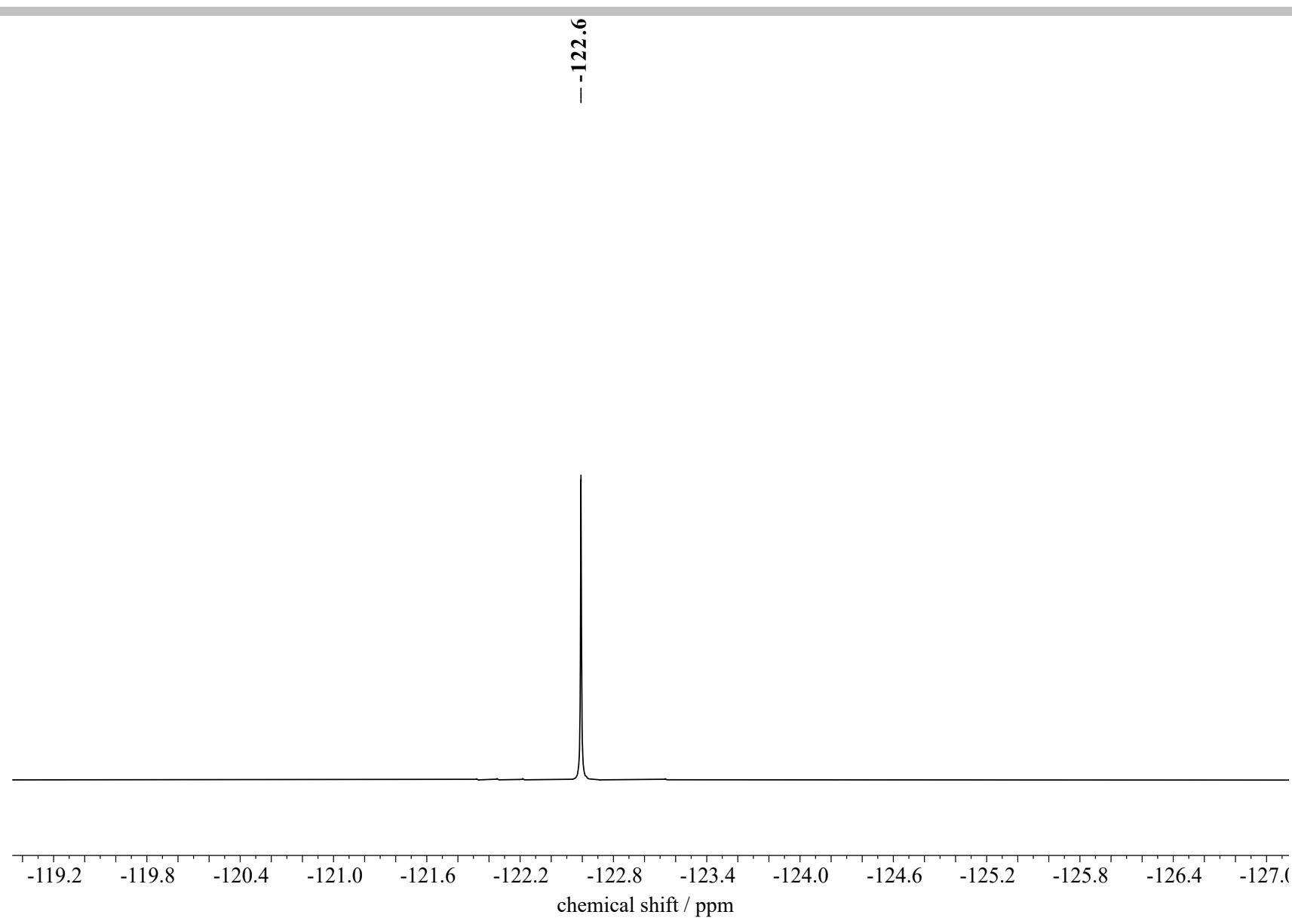


**Figure S32**. $^{19}F$ (282 MHz) NMR spectra of **26** in DMSO-$d_6$.

**Synthesis of 1-(4-bromo-2-fluorophenyl)-2-(3,4-dimethoxyphenyl)diazene (27)**

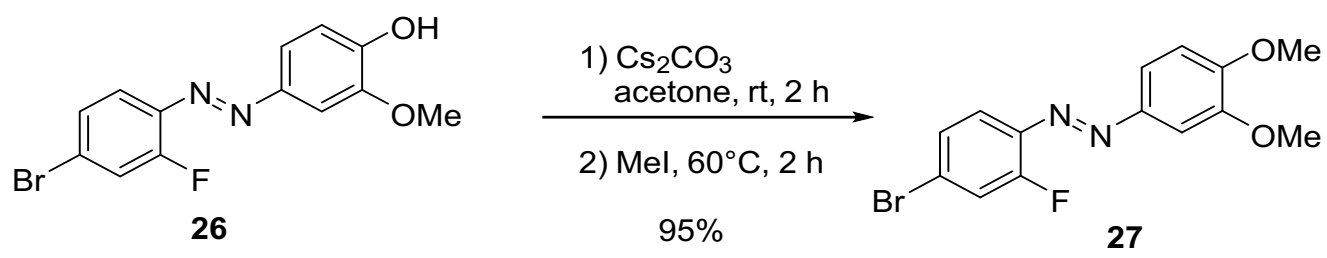


4-((4-Bromo-2-fluorophenyl)diazenyl)-2-methoxyphenol **26** (100 mg, 308 µmol, 1.00 eq) was dissolved in 20 mL acetone, and cesium carbonate (201 mg, 615 µmol, 2.00 eq) was added. The resulting suspension was stirred for 2 h at room temperature. Iodomethane (131 mg, 923 µmol, 3.00 eq) was added and the reaction mixture was refluxed at 60 °C for 2 h. The reaction mixture was cooled to room temperature. The reaction was then diluted with 100 mL distilled water and extracted with 100 mL EtOAc. The organic layer was washed with 100 mL distilled water and brine, dried over $MgSO_4$ and solvent was removed under reduced pressure. The crude product was purified by flash column chromatography (DCM) to yield the desired compound 1-(4-bromo-2-fluorophenyl)-2-(3,4-dimethoxyphenyl)diazene (**27**, 99 mg, 293 µmol, 95%) as an orange solid.

**TLC:** $R_f$ = 0.50 (DCM).

**$^1$H-NMR (300 MHz, DMSO-*d*$_6$, *δ*):** 7.85 (dd, $^3J$ = 10.5 Hz, $^4J$ = 2.0 Hz, 1H, $H_{arom.}$), 7.68-7.62 (m, 2H, $H_{arom.}$, $H_{arom.}$), 7.57-7.53 (m, 2H, $H_{arom.}$), 7.43 (d, $^4J$ = 2.3 Hz, 1H, $H_{arom.}$), 7.20 (d, $^3J$ = 8.6 Hz, 1H, $H_{arom.}$), 3.89 (s, 3H, C*H*$_3$) , 3.85 (s, 3H, C*H*$_3$) ppm.

**$^{13}$C-NMR (75 MHz, DMSO-*d*$_6$, *δ*):** 156.5 ($C_{arom.}$), 152.8 ($C_{arom.}$), 150.0 ($C_{arom.}$), 146.3 ($C_{arom.}$), 139.1 (d, $^3J_{FC}$ = 6.7 Hz, $C_{arom.}$), 128.3 (d, $^3J_{FC}$ = 3.3 Hz, $C_{arom.}$H), 124.2 ($C_{arom.}$H), 121.4 ($C_{arom.}$H), 120.7 (d, $^2J_{FC}$ = 23.6 Hz, $C_{arom.}$H), 119.1 ($C_{arom.}$H), 110.2 ($C_{arom.}$H), 102.0 ($C_{arom.}$H), 55.9 (*C*H$_3$), 55.5 (*C*H$_3$) ppm.

**$^{19}$F-NMR (282 MHz, DMSO-*d*$_6$, *δ*)**: -122.4 (C*F*) ppm.

**HRMS-ESI (m/z):** $[M+H]^+$ calcd for $C_{14}H_{13}BrFN_2O_2$, 339.0139; found, 339.0145.

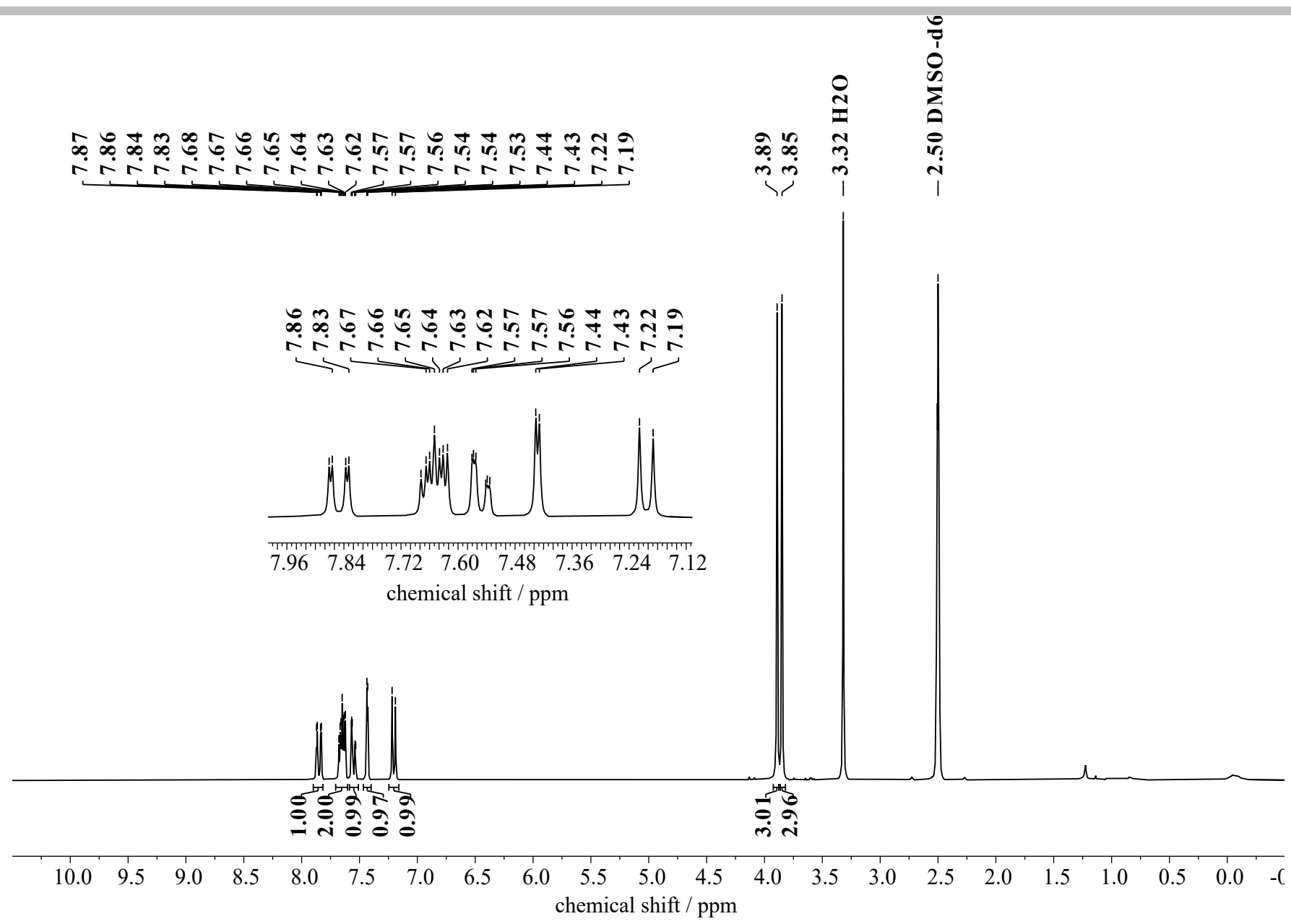


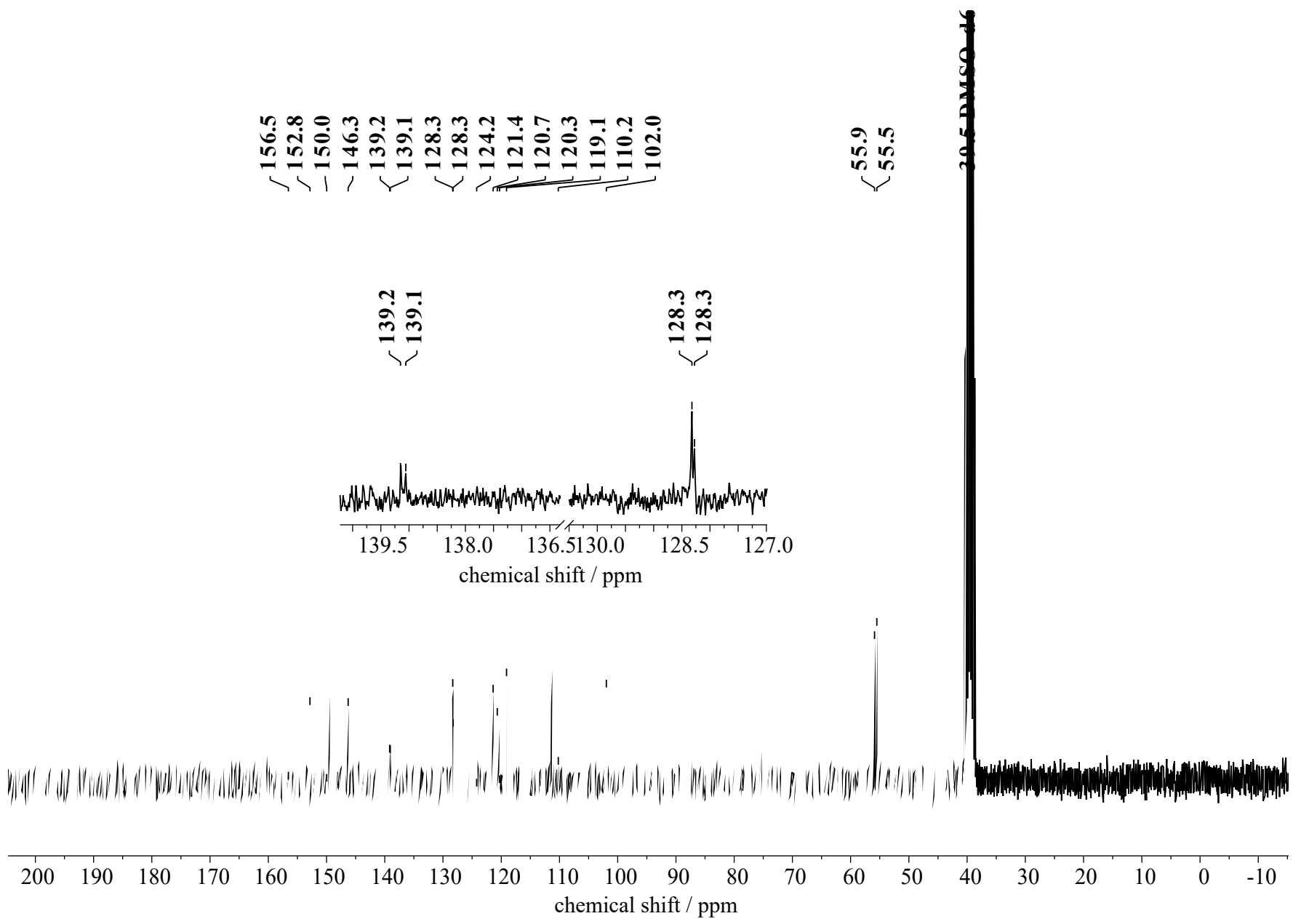


**Figure S33**. $^{1}$H (300 MHz) and $^{13}$C (75 MHz) NMR spectra of **27** in DMSO-$d_6$.

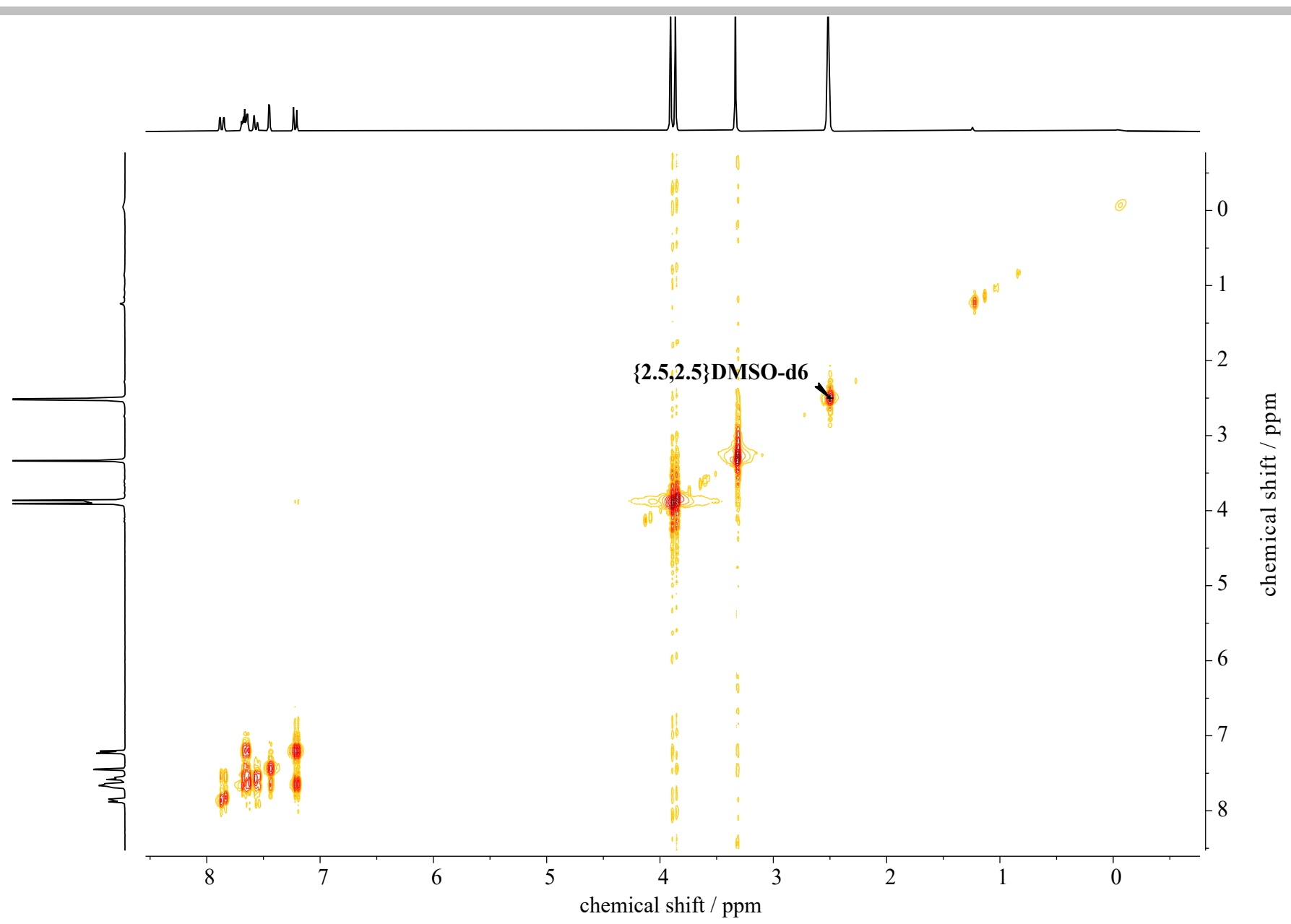


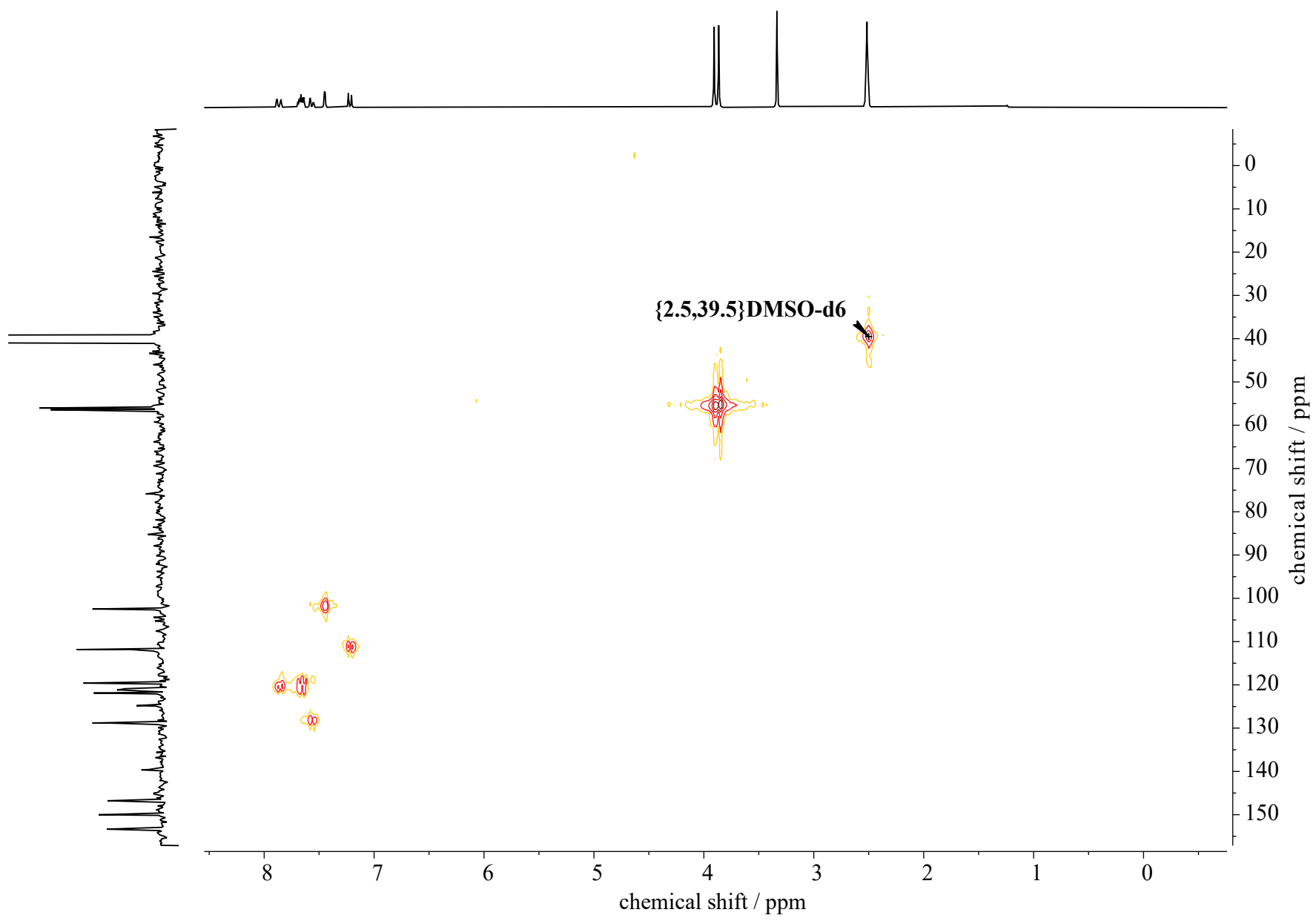


**Figure S34**. COSY and HMQC NMR spectra of **27** in DMSO-$d_6$.

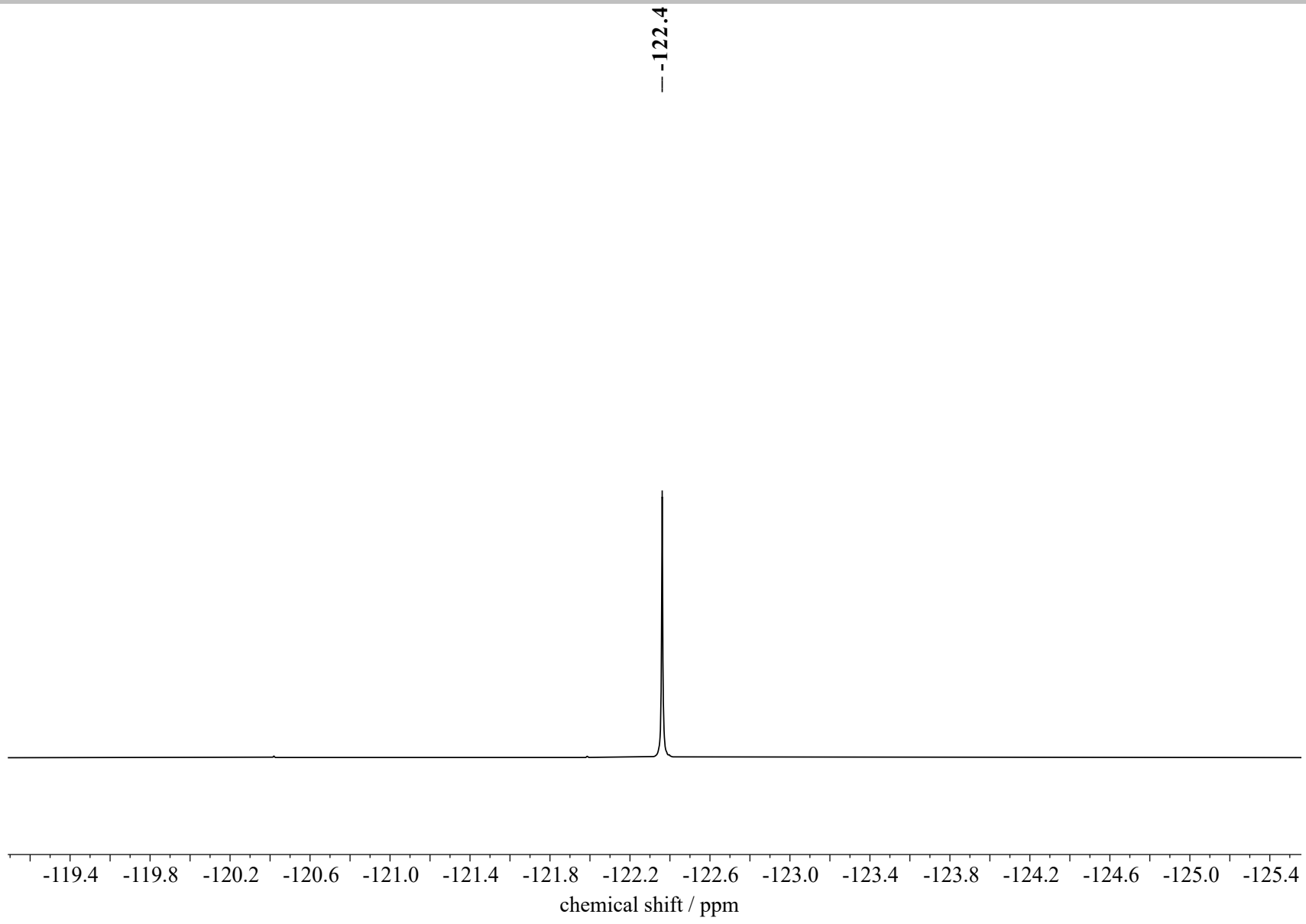


**Figure S35**. $^{19}F$ (282 MHz) NMR spectra of **27** in DMSO-$d_6$.

**Synthesis of tert-butyl-4-(4-((3,4-dimethoxyphenyl)diazenyl)-3-fluorophenyl)-3,6-dihydropyridine-1(2H)-carboxylate (28)**

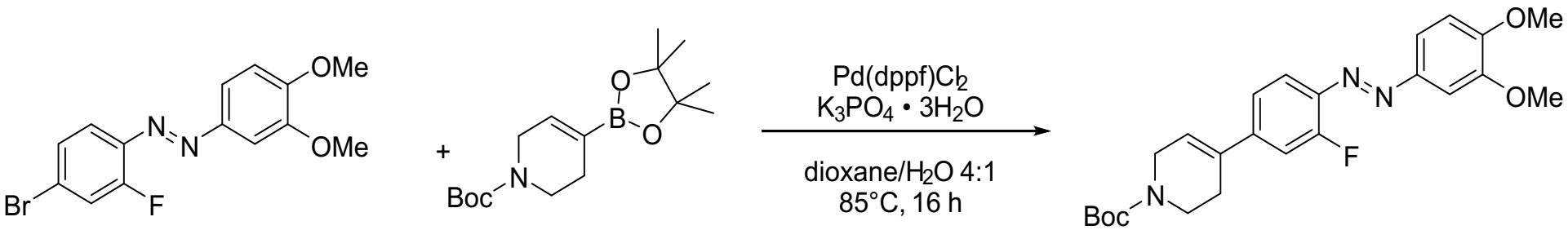


**27** **16** **28**

1-(4-Bromo-2-fluorophenyl)-2-(3,4-dimethoxyphenyl)diazene **27** (99 mg, 292 µmol, 1.20 eq), *N-tert*-butoxycarbony-1,2,3,6-tetrahydropyridine-4-boronic acid pinacol ester **16** (75 mg, 243 µmol, 1.00 eq), Pd(dppf)$Cl_2$ (18 mg, 24.3 µmol, 0.10 eq) and $K_3PO_4$·$3H_2O$ (194 mg, 730 µmol, 3.00 eq) were dissolved in mixture of dioxane/distilled water (20 mL, 4:1, v%). The reaction was stirred under $N_2$ atmosphere at 85 °C for 16 h. The reaction was then diluted with 100 mL distilled water and extracted with 100 mL EtOAc. The organic layer was washed with 100 mL distilled water and brine, dried over $MgSO_4$ and solvent was removed under reduced pressure. The crude product was purified by flash column chromatography (DCM) to yield the desired compound *tert*-butyl-4-(4-((3,4-dimethoxyphenyl)diazenyl)-3-fluorophenyl)-3,6-dihydropyridine-1(*2H*)-carboxylate (**28**, 94 mg, 213 µmol, 88%) as an orange solid.
**TLC:** $R_f$ = 0.12 (DCM).
**$^1$H-NMR (300 MHz, DMSO-$d_6$, δ):** 7.67 (t, $^3J$ = 8.3 Hz, 1H, $H_{arom.}$), 7.62 (dd, $^3J$ = 8.5 Hz, $^4J$ = 2.2 Hz, 1H, $H_{arom.}$), 7.53 (dd, $^3J$ = 12.7 Hz, $^4J$ = 1.9 Hz, 1H, $H_{arom.}$), 7.44-7.41 (m, 2H, $H_{arom.}$), 7.19 (d, $^3J$ = 8.6 Hz, 1H, $H_{arom.}$), 6.42 (s, 1H, C*H*=C), 4.06-4.04 (m, 2H, C$H_2$), 3.89 (s, 3H, C$H_3$), 3.85 (s, 3H, C$H_3$), 3.55 (t, $^3J$ = 5.6 Hz, 2H, C$H_2$), 2.53-2.51 (m, 2H, C$H_2$), 1.43 (s, 9H, 3xC$H_3$) ppm.
**$^{13}$C-NMR (75 MHz, DMSO-$d_6$, δ):** 160.9 ($C_{arom.}$), 157.9 ($C_{arom.}$), 153.8 ($C_{arom.}$), 152.5 ($C_{arom.}$), 149.5 ($C_{arom.}$), 146.4 ($C_{arom.}$), 144.2 (d, $^3J_{FC}$ = 8.0 Hz, $C_{arom.}$), 138.6 (d, $^3J_{FC}$ = 7.4 Hz, $C_{arom.}$), 132.9 ($C_{arom.}$H), 124.2 (*C*H=C), 120.9 (d, $^2J_{FC}$ = 20.8 Hz, $C_{arom.}$H), 117.4 ($C_{arom.}$H), 113.0 (d, $^2J_{FC}$ = 20.9 Hz, $C_{arom.}$H), 111.3 ($C_{arom.}$H), 102.0 ($C_{arom.}$H), 78.9 ($C_{q.}$), 55.9 ($CH_3$), 55.5 ($CH_3$), 43.2 ($CH_2$), 28.1 (3C, 3x$CH_3$), 25.9 ($CH_2$) ppm.
**$^{19}$F-NMR (282 MHz, DMSO-$d_6$, δ)**: -124.7 (C*F*) ppm.
**HRMS-ESI (m/z):** $[M+H]^+$ calcd for $C_{24}H_{29}FN_3O_4$, 442.2137; found, 442.2140.

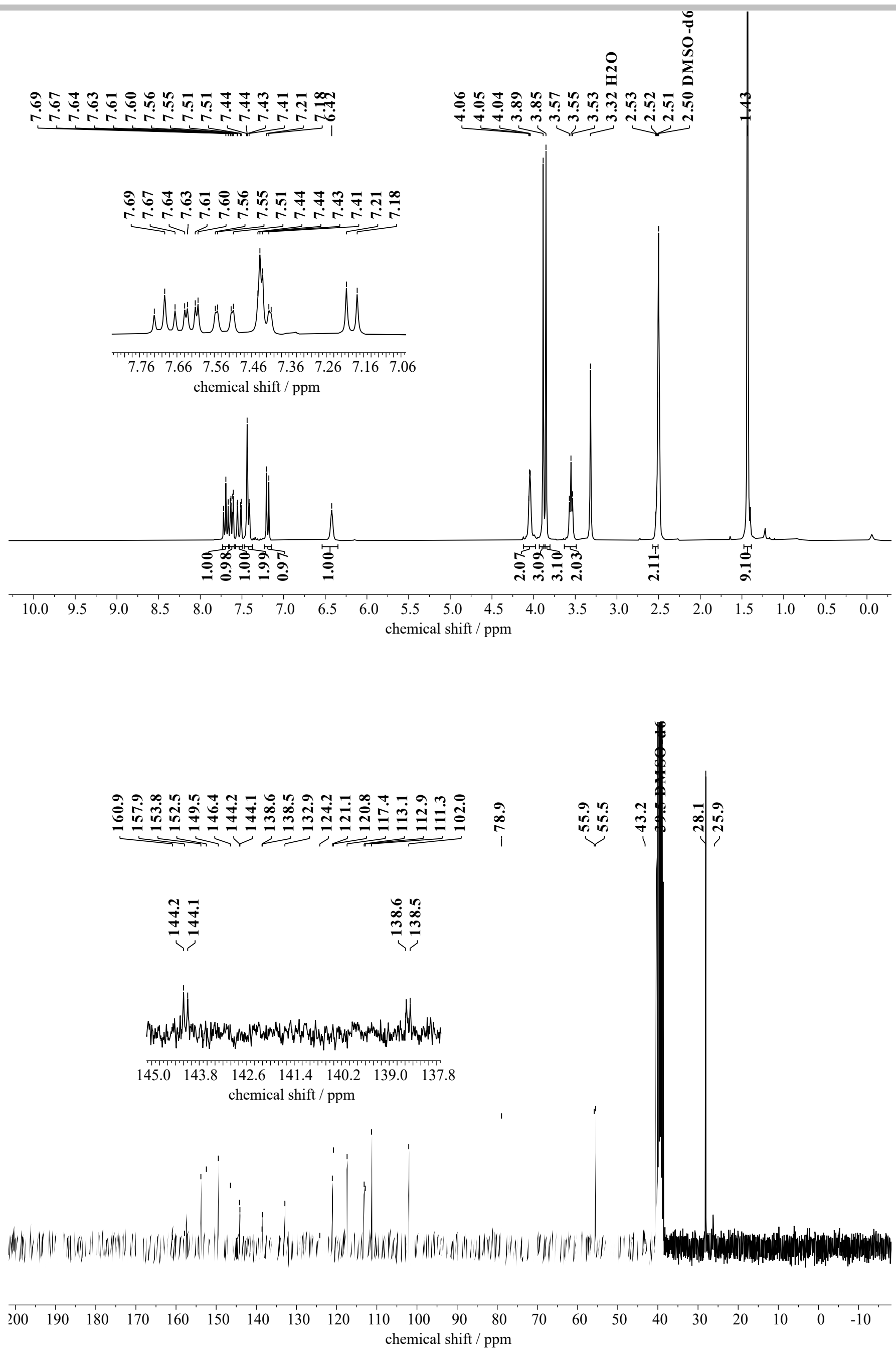


**Figure S36**. $^{1}H$ (300 MHz) and $^{13}C$ (75 MHz) NMR spectra of **28** in DMSO-$d_6$.

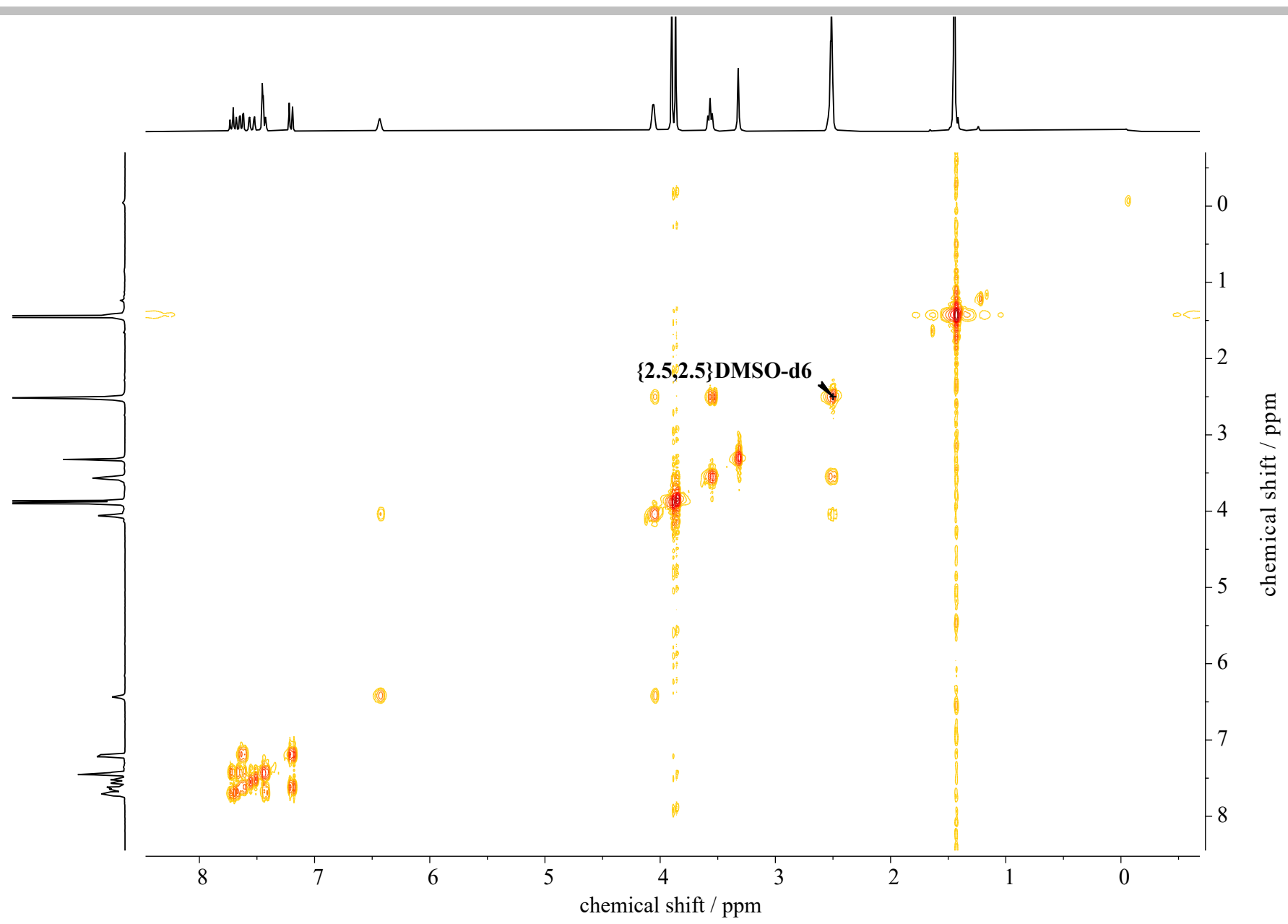


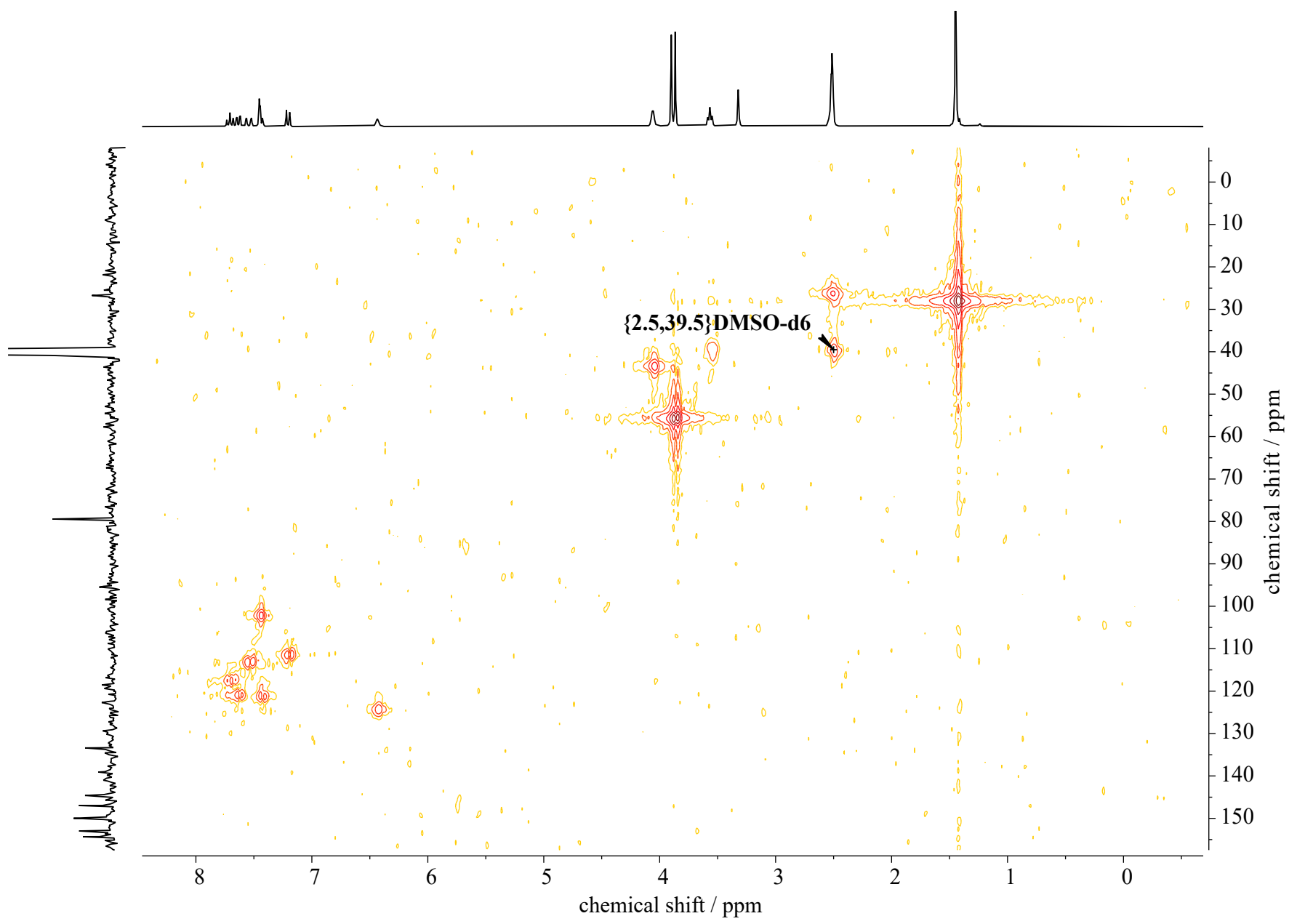


**Figure S37**. COSY and HMQC NMR spectra of **28** in DMSO-$d_6$.

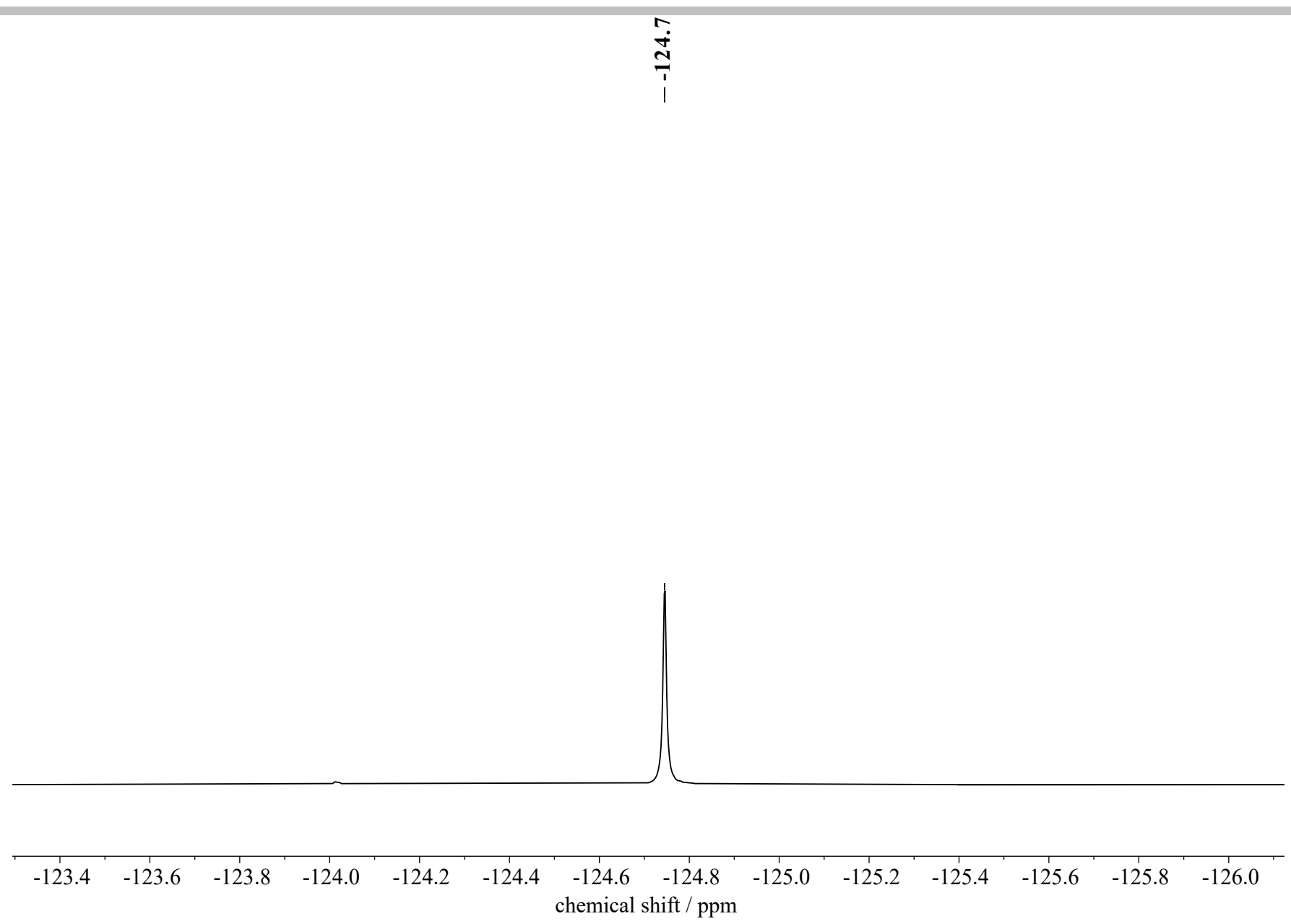


**Figure S38**. $^{19}$F (282 MHz) NMR spectra of **28** in DMSO-$d_6$.

**Synthesis of 4-(4-((3,4-dimethoxyphenyl)diazenyl)-3-fluorophenyl)-1,2,3,6-tetrahydropyridin (11)**

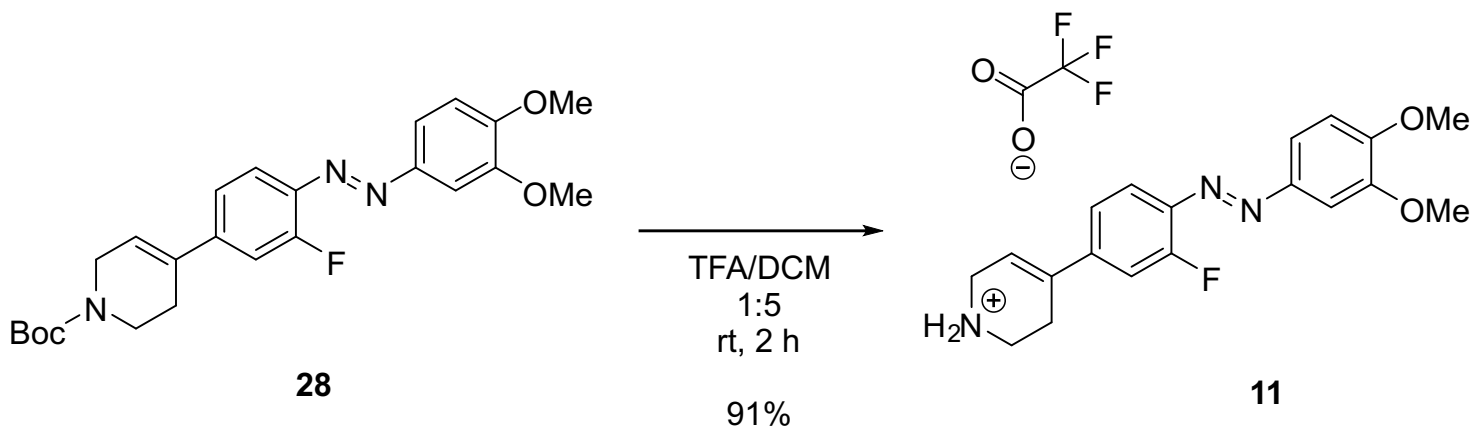


*Tert*-butyl-4-(4-((3,4-dimethoxyphenyl)diazenyl)-3-fluorophenyl)-3,6-dihydropyridine-1(*2H*)-carboxylate **28** (50 mg, 113 µmol, 1.00 eq) was dissolved in mixture of TFA/DCM (12 mL, 1:5, v%). The reaction was stirred at room temperature for 2 h. The reaction was then diluted with 100 mL distilled water and extracted with 100 mL EtOAc. The organic layer was washed with 100 mL distilled water and brine, dried over $MgSO_4$ and solvent was removed under reduced pressure. The crude product was purified by flash column chromatography (EtOAc/MeOH, 1:1, v%) to yield the desired compound 4-(4-((3,4-dimethoxyphenyl)diazenyl)-3-fluorophenyl)-1,2,3,6-tetrahydropyridin (**11**, 47 mg, 103 µmol, 91%) as an orange solid in form of a TFA salt.

**TLC:** $R_f$ = 0.30 (EtOAc/MeOH, 1:1, v%).

**$^1$H-NMR (300 MHz, DMSO-*d*$_6$, *δ*):** 8.88 (br, 2H, N$H_2$), 7.73 (t, $^3J$ = 8.3 Hz, 1H, $H_{arom.}$), 7.66-7.60 (m, 2H, $H_{arom.}$, $H_{arom.}$),7.48 (dd, $^3J$ = 8.6 Hz, $^4J$ = 1.9 Hz, 1H, $H_{arom.}$), 7.44 (d, $^4J$ = 2.3 Hz, 1H, $H_{arom.}$), 7.21 (d, $^3J$ = 8.1 Hz, 1H, $H_{arom.}$), 6.47 (s, 1H, C*H*=C), 3.89 (s, 3H, C$H_3$), 3.85 (s, 3H, C$H_3$), 3.87-3.83 (m, 2H, C$H_2$), 3.38-3.33 (m, 2H, C$H_2$), 2.76-2.73 (m, 2H, C$H_2$) ppm.

**$^{13}$C-NMR (75 MHz, DMSO-*d*$_6$, *δ*):** 152.6 ($C_{arom.}$), 149.1 ($C_{arom.}$), 146.4 ($C_{arom.}$), 142.7 (d, $^3J_{FC}$ = 7.6 Hz, $C_{arom.}$), 139.1 ($C_{arom.}$), 137.8 ($C_{arom.}$), 132.7 ($C_{arom.}$), 121.3 ($C_{arom.}$H), 121.1 ($C_{arom.}$H), 119.8 (*C*H=C), 117.7 ($C_{arom.}$H), 113.3 (d, $^2J_{FC}$ = 21.1 Hz, $C_{arom.}$H), 111.3 ($C_{arom.}$H), 102.0 ($C_{arom.}$H), 55.9 ($CH_3$), 55.5 ($CH_3$), 41.8 ($CH_2$), 22.9 ($CH_2$) ppm.

**$^{19}$F-NMR (282 MHz, DMSO-*d*$_6$, *δ*)**: -124.5 (C*F*) ppm.

**HRMS-ESI (m/z):** $[M+H]^+$ calcd for $C_{19}H_{21}FN_3O_2$, 342.1618; found, 342.1612.

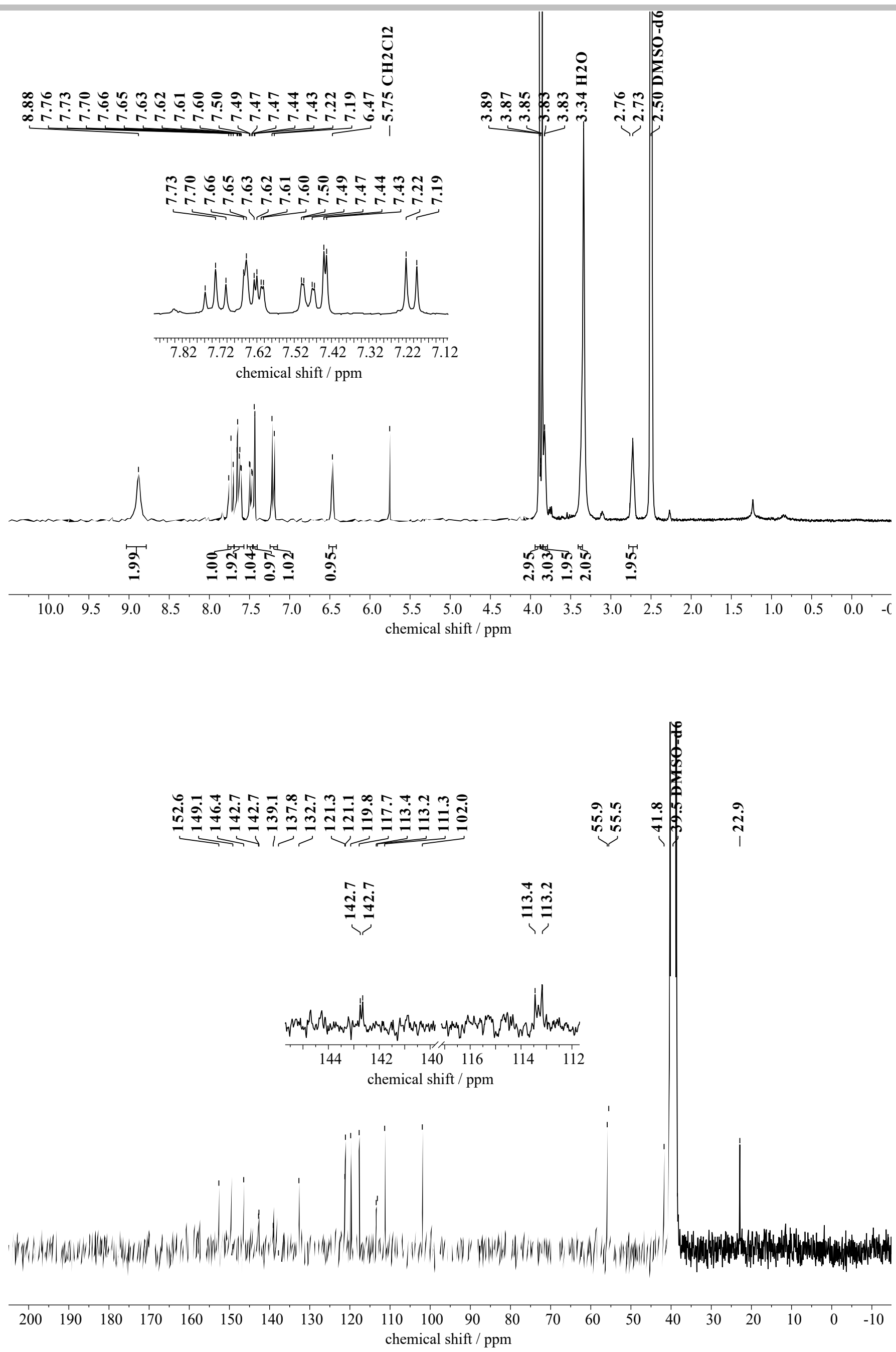


**Figure S39**. $^{1}$H (300 MHz) and $^{13}$C (75 MHz) NMR spectra of **11** in DMSO-$d_6$.

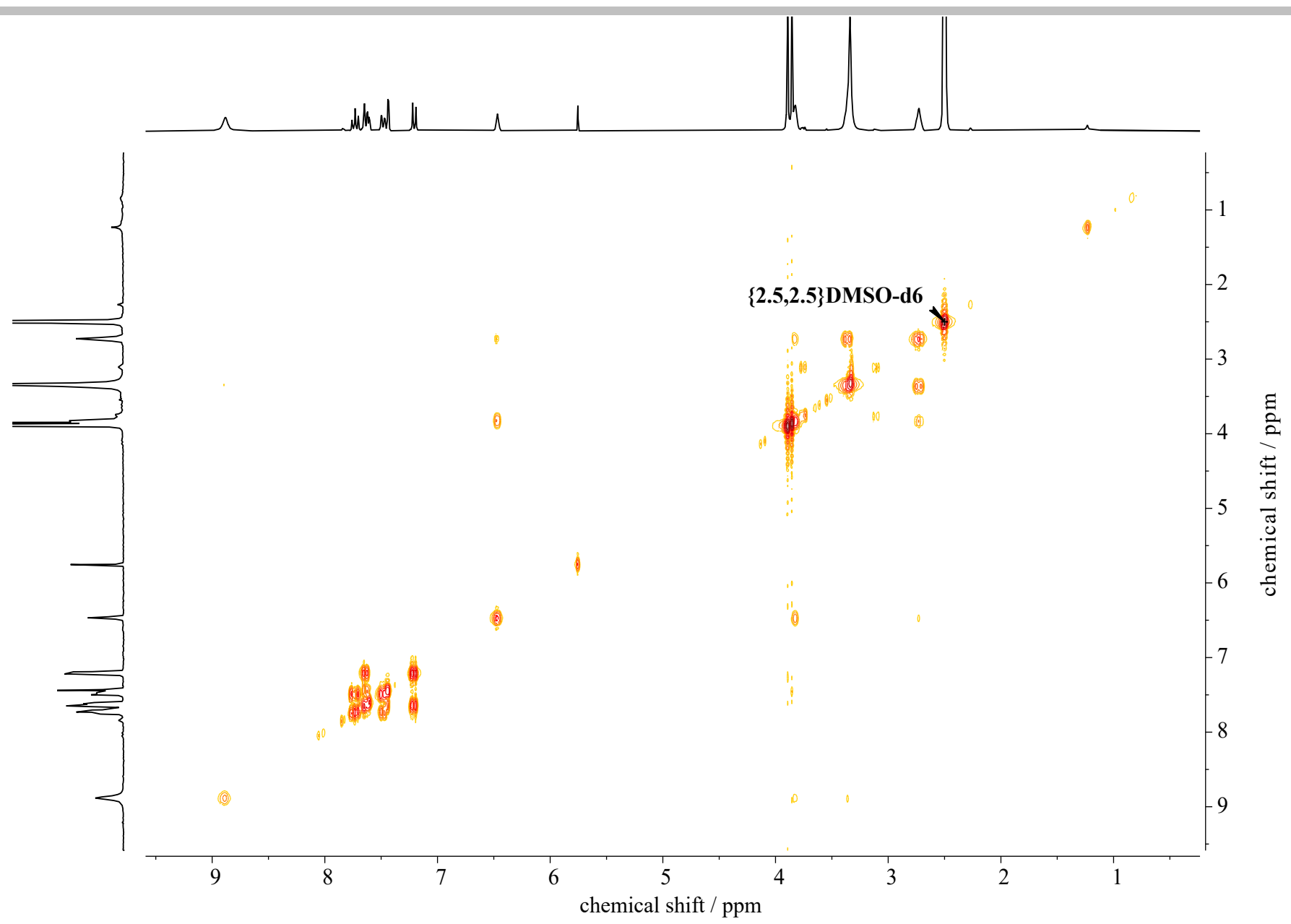


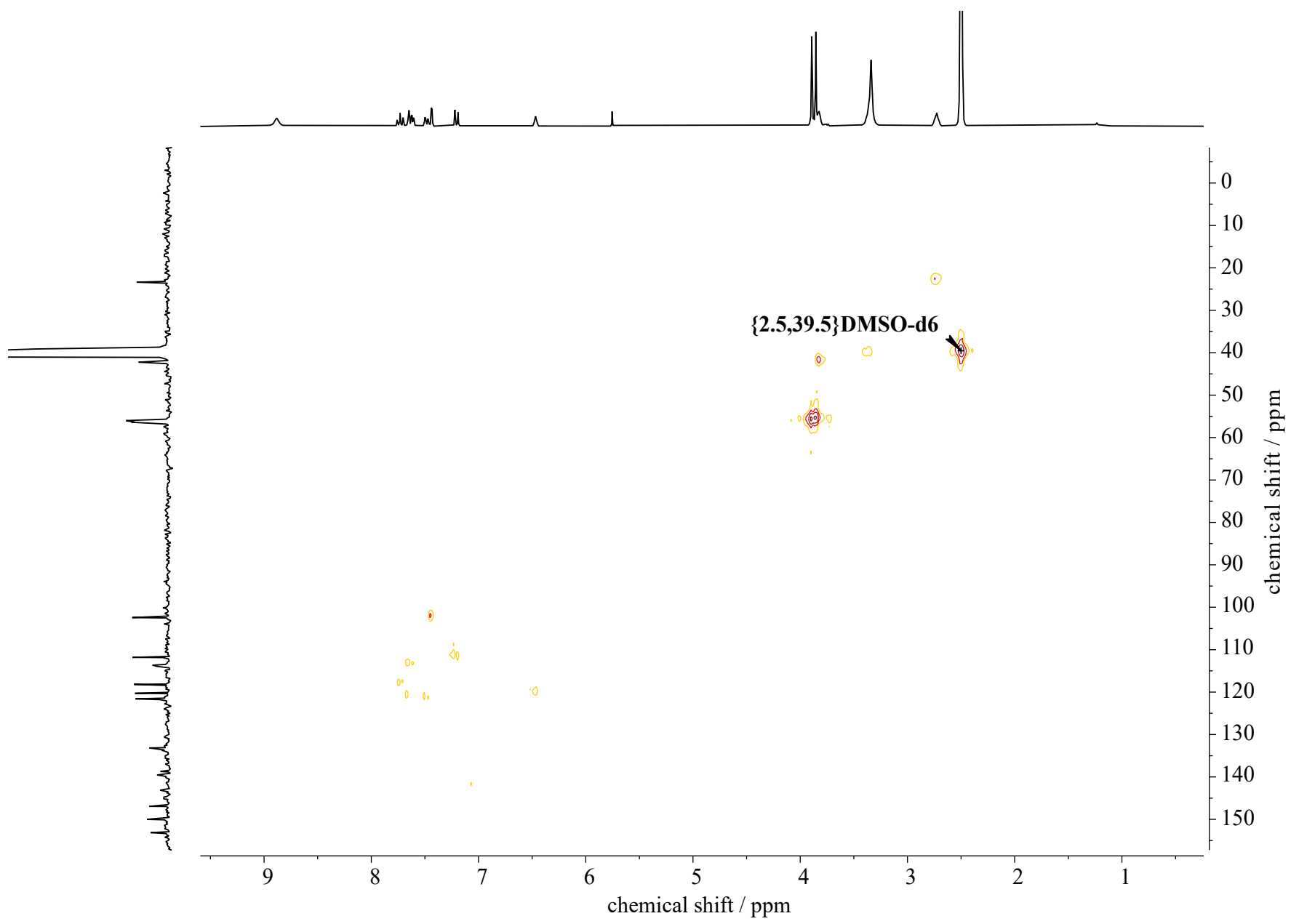


**Figure S40**. COSY and HMQC NMR spectra of **11** in DMSO-$d_6$.

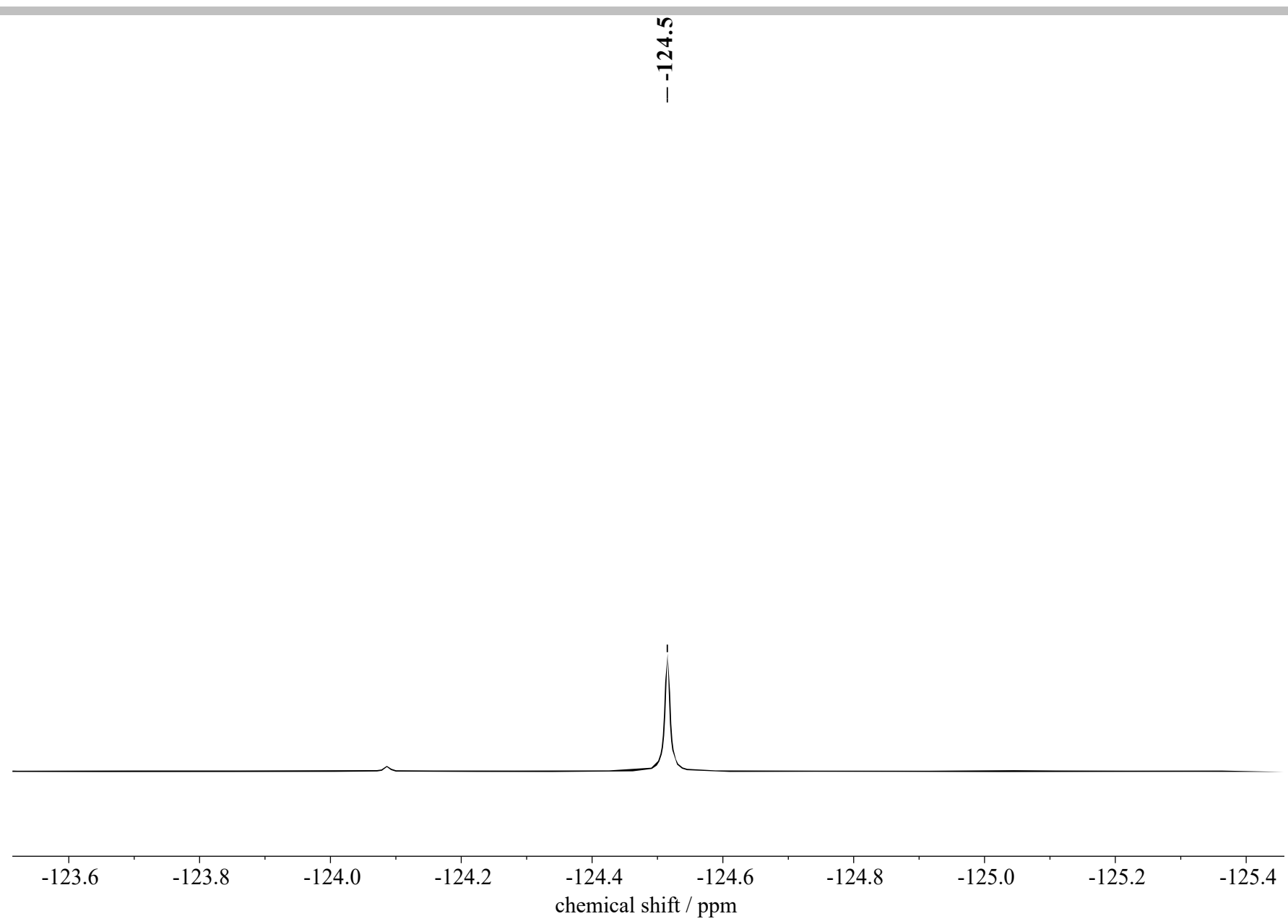


**Figure S41**. $^{19}$F (282 MHz) NMR spectra of **11** in DMSO-$d_6$.

**Synthesis of 4-bromo-2,6-difluoroacetanilide (30)**

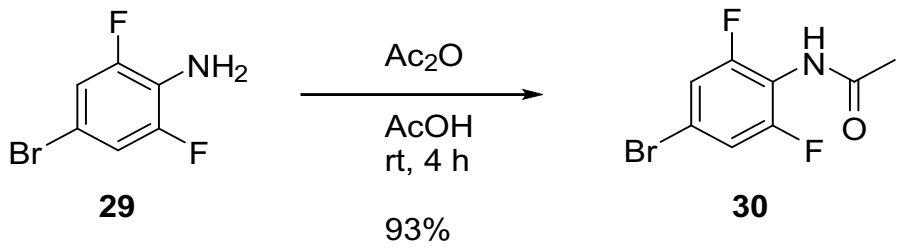


4-Bromo-2,6-difluoroaniline **29** (6.00 g, 28.8 μmol, 1.00 eq) was dissolved in 100 mL acetic acid at room temperature, acetic anhydride (18 mL, 190 mmol, 6.60 eq) was added dropwise via dropping funnel. The resulting mixture was stirred for 4 h. The reaction mixture was poured into 500 mL ice cold distilled water. The precipitate was filtered and washed with 500 mL distilled water. The residue was dissolved in 500 mL DCM and washed with 500 mL saturated solution of sodium bicarbonate and brine. The organic layer was dried over $MgSO_4$ and solvent was removed under reduced pressure affording product 4-bromo-2,6-difluoroacetanilide as colorless solid (**30**, 6.71 g, 26.8 mmol, 93%).

**TLC:** $R_f$ = 0.70 (DCM).

**$^1$H-NMR (300 MHz, DMSO-$d_6$, $\delta$):** 9.73 (s, 1H, N*H*), 7.53 (d, $^3J$ = 7.3 Hz, 2H, 2x$H_{arom.}$), 2.06 (s, 3H, C$H_3$) ppm.

**$^{13}$C-NMR (75 MHz, DMSO-$d_6$, $\delta$):** 168.3 (*C*=O), 157.6 (dd, $^1J_{FC}$ = 252.2 Hz, $^3J_{FC}$ = 6.3 Hz, 2C, *C*F), 118.4 (d, $^2J_{FC}$ = 11.5 Hz, $C_{arom.}$), 115.7 (dd, $^2J_{FC}$ = 25.3 Hz, $^3J_{FC}$ = 2.5 Hz, 2C, $C_{arom.}$H), 114.5 (d, $^2J_{FC}$ = 16.9 Hz, $C_{arom.}$), 22.3 ($CH_3$) ppm.

**$^{19}$F-NMR (282 MHz, DMSO-$d_6$, $\delta$)**: -115.7 (C*F*) ppm.

**HRMS-ESI (m/z):** $[M+H]^+$ calcd for $C_8H_6BrF_2NO$, 251.9654; found, 251.9659.

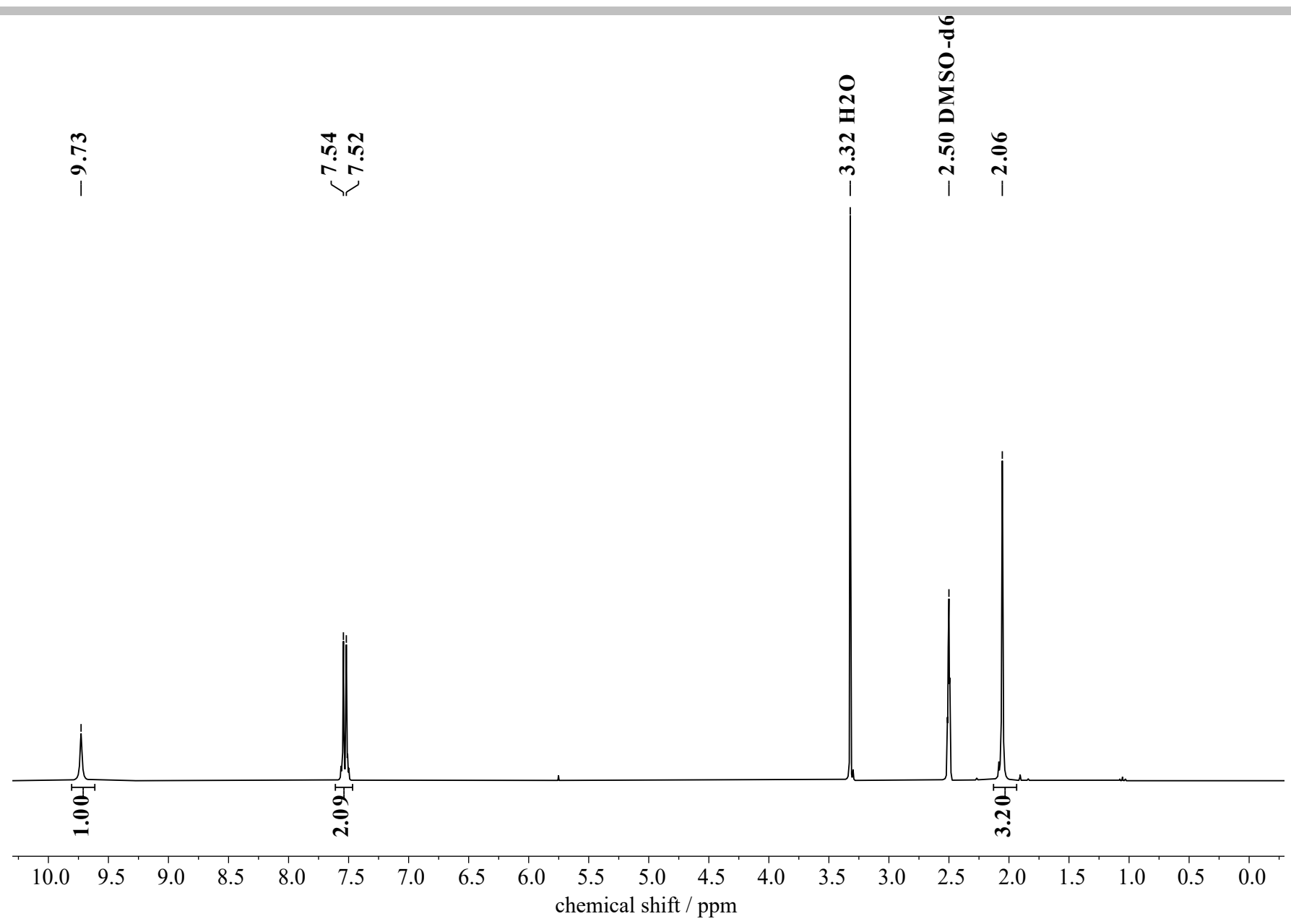


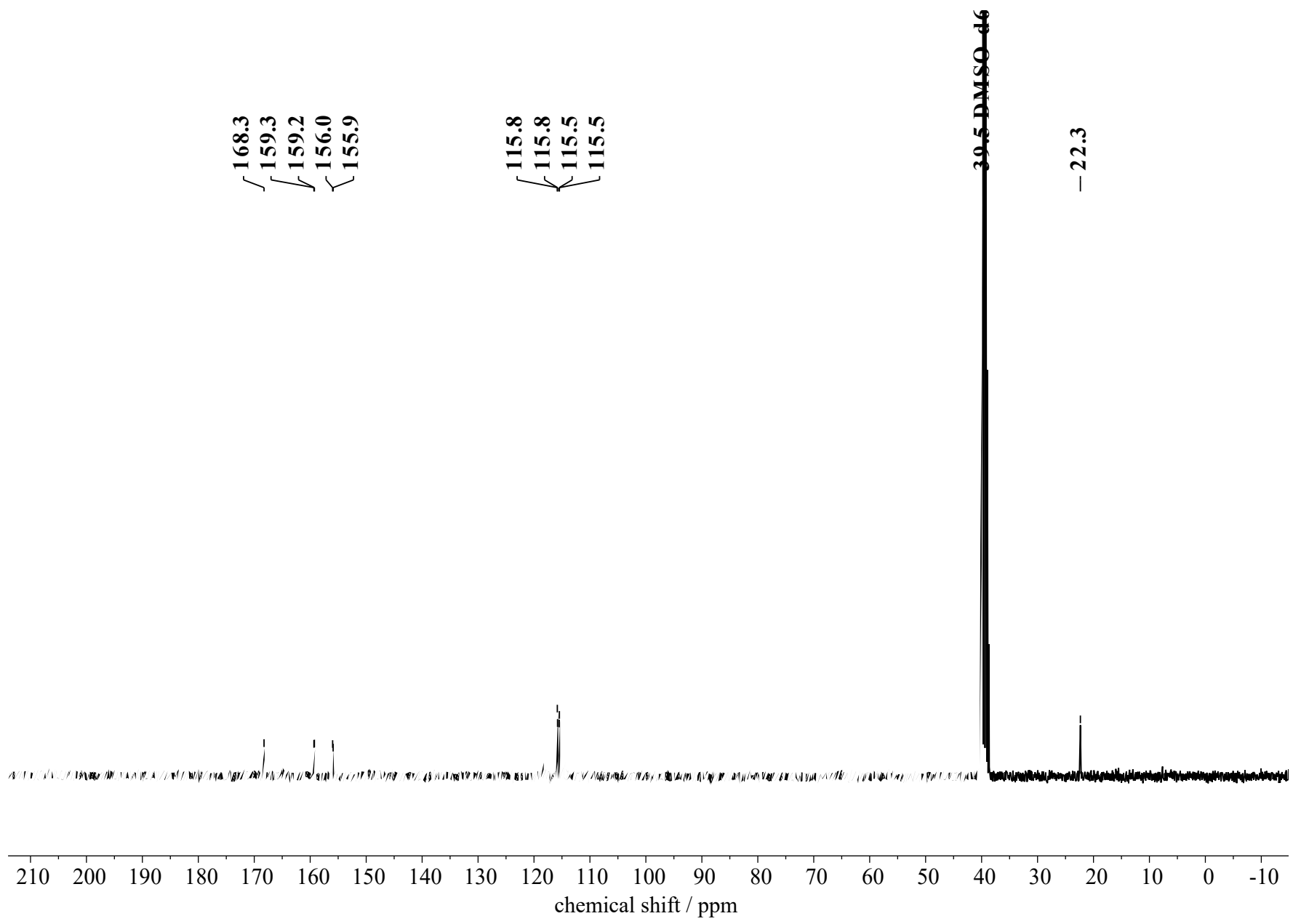


**Figure S42**. $^{1}$H (300 MHz) and $^{13}$C (75 MHz) NMR spectra of **30** in DMSO-$d_6$.

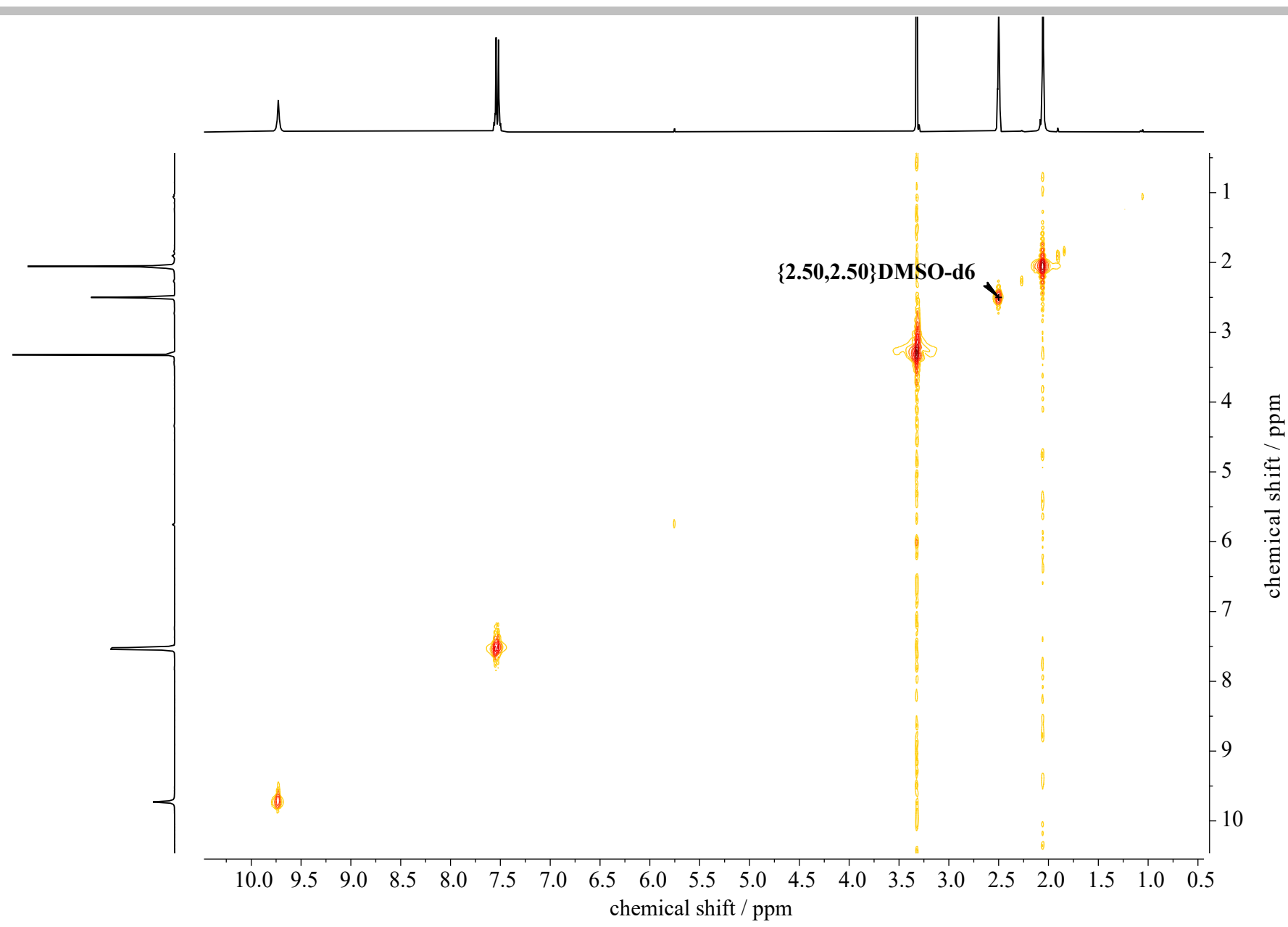


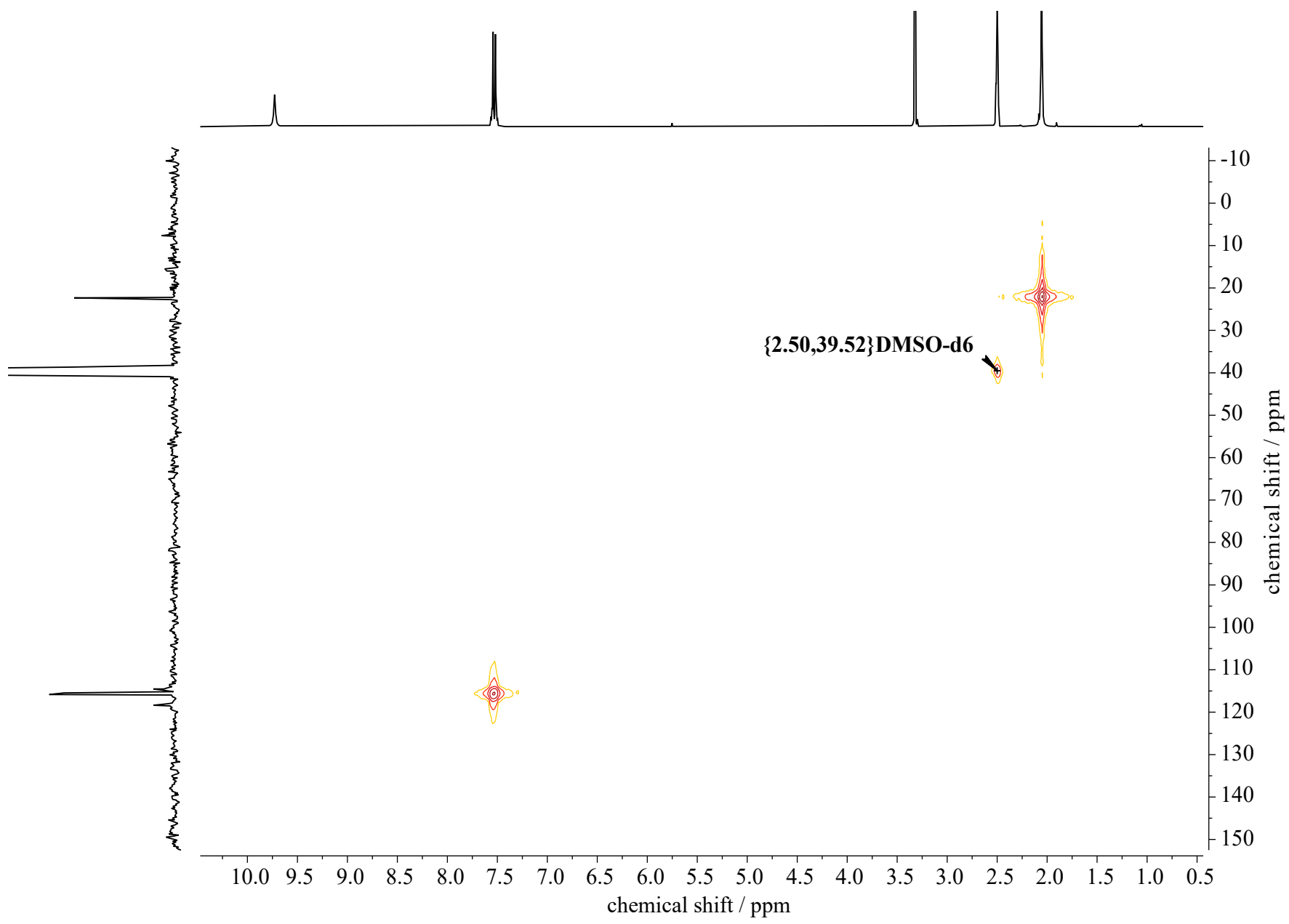


**Figure S43**. COSY and HMQC NMR spectra of **30** in DMSO-$d_6$.

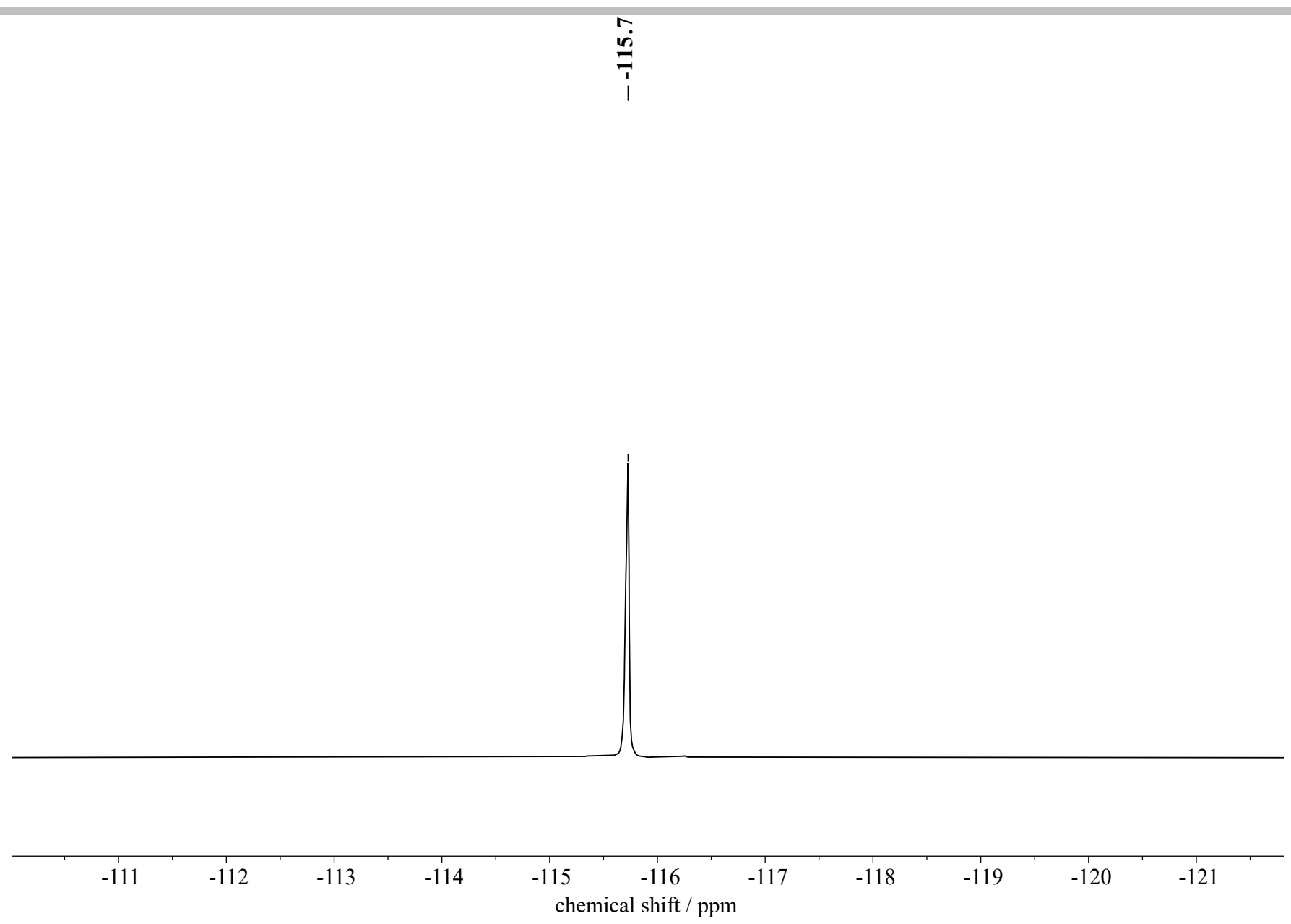


**Figure S44**. $^{19}$F (282 MHz) NMR spectra of **30** in DMSO-$d_6$.

**Synthesis of 6-bromo-4-fluoro-2-methylbenzo[d]oxazole (31)**

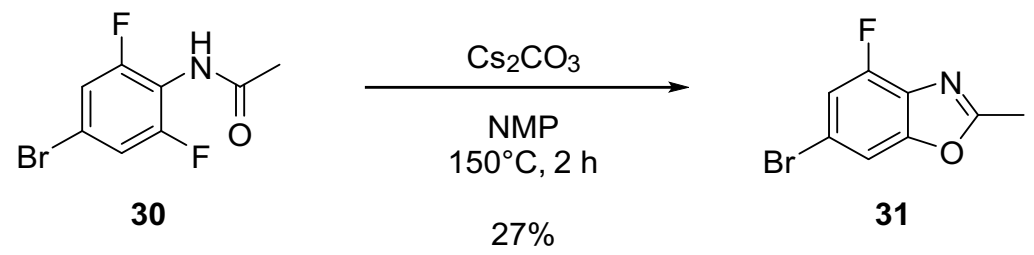


4-Bromo-2,6-difluoroacetanilide **30** (5.00 g, 20.0 mmol, 1.00 eq) was dissolved in 20 mL NMP. Cesium carbonate (13.0 g, 40.0 mmol, 2.00 eq) was added and the mixture heated to 150 °C for 2 h. The reaction was cooled to room temperature, diluted with 200 mL distilled water, and extracted three times with 200 mL EtOAc. The organic phase was combined and washed with brine, dried over $Na_2SO_4$ and solvent was removed under reduced pressure. The crude product was purified by flash column chromatography (*n*-pentane/EtOAc, 10:1, v%) to yield the desired compound 6-bromo-4-fluoro-2-methylbenzo[*d*]oxazole (**31**, 1.22 g, 5.40 mmol, 27%) as a brown solid.

**TLC:** $R_f$ = 0.50 (*n*-pentane/EtOAc, 10:1, v%).

**$^1$H-NMR (300 MHz, $CDCl_3$, *δ*):** 7.47 (d, $^4J$ = 1.6 Hz, 1H, $H_{arom.}$), 7.21 (dd, $^3J_{HF}$ = 9.1 Hz, $^4J$ = 1.5 Hz, 1H, $H_{arom.}$), 2.64 (s, 3H, C$H_3$) ppm.

**$^{13}$C-NMR (75 MHz, $CDCl_3$, *δ*):** 164.7 (N=*C*-O), 154.3 (d, $^1J_{FC}$ = 261.7 Hz, *C*F), 153.6 (d, $^3J_{FC}$ = 8.4 Hz, $C_{arom.}$), 150.9 ($C_{arom.}$), 129.8 (d, $^2J_{FC}$ = 16.5 Hz, $C_{arom.}$), 117.0 (d, $^2J_{FC}$ = 8.5 Hz, $C_{arom.}$), 114.9 (d, $^2J_{FC}$ = 21.0 Hz, $C_{arom.}$H), 110.4 (d, $^4J_{FC}$ = 4.8 Hz, $C_{arom.}$H), 14.5 ($CH_3$) ppm.

**$^{19}$F-NMR (282 MHz, $CDCl_3$, *δ*)**: -123.6 (C*F*) ppm.

**HRMS-ESI (m/z):** $[M+H]^+$ calcd for $C_8H_5BrFNO$, 231.9591; found, 231.9596.

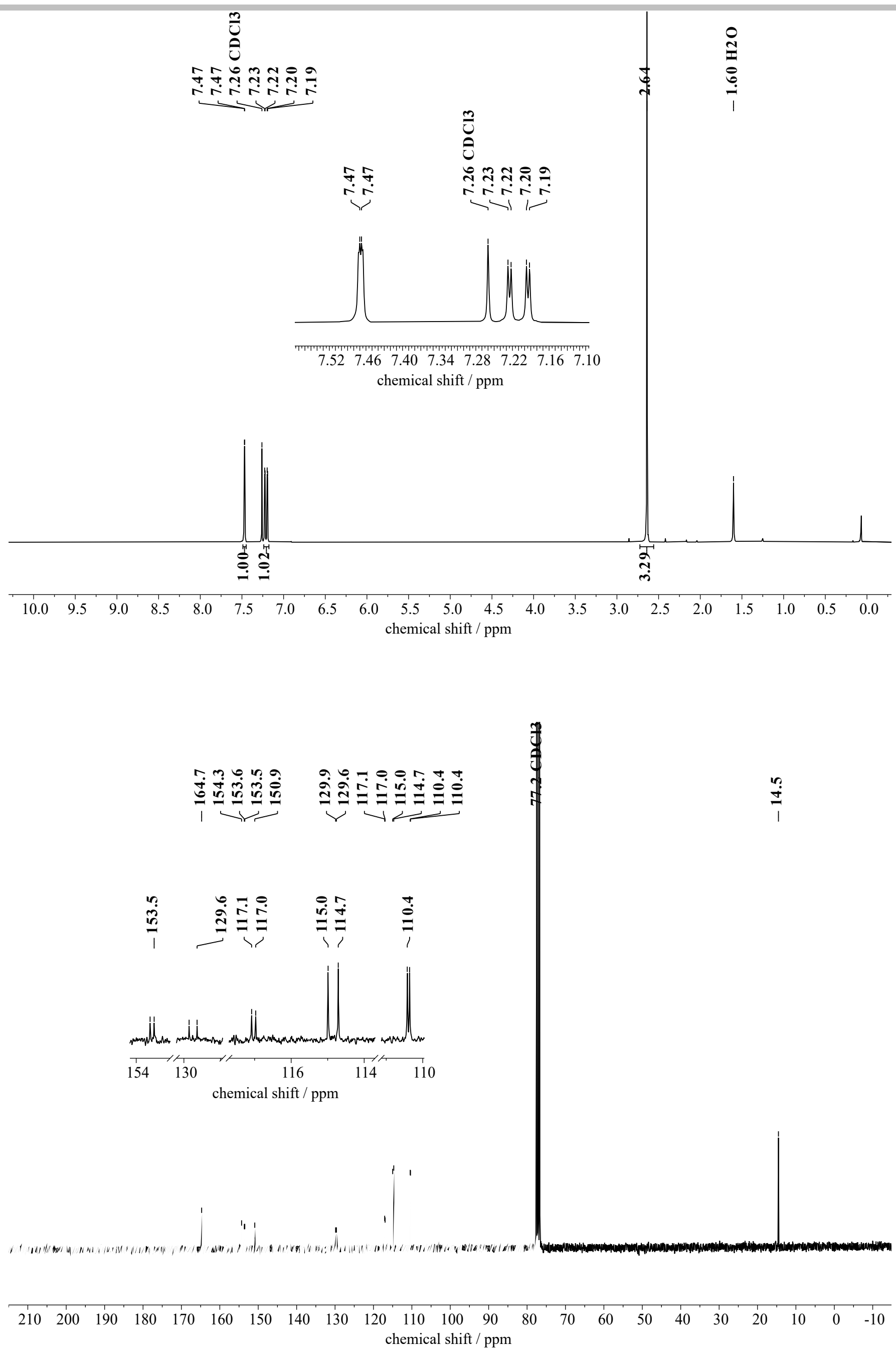


**Figure S45.** $^{1}H$ (300 MHz) and $^{13}C$ (75 MHz) NMR spectra of **31** in $CDCl_3$.

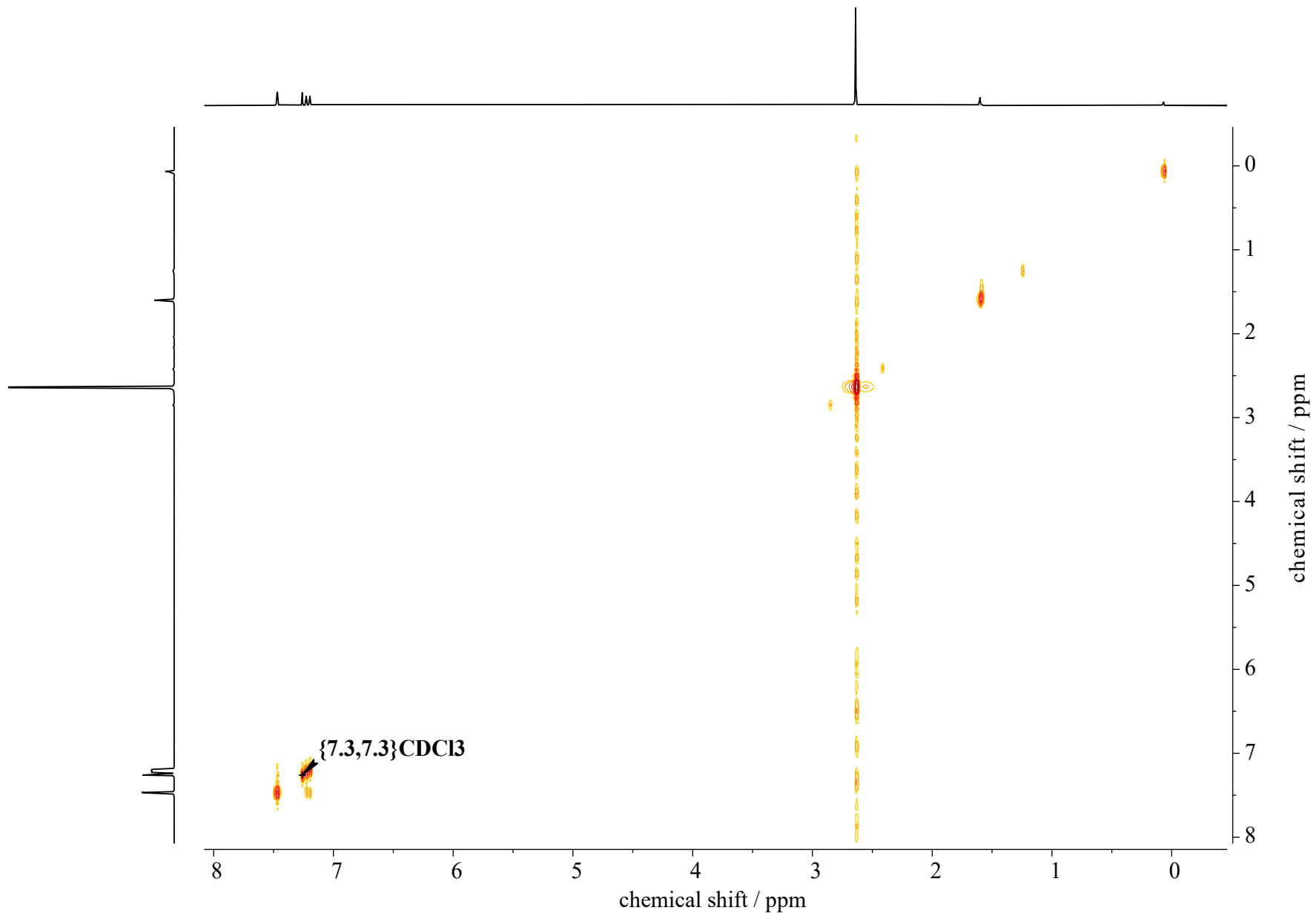


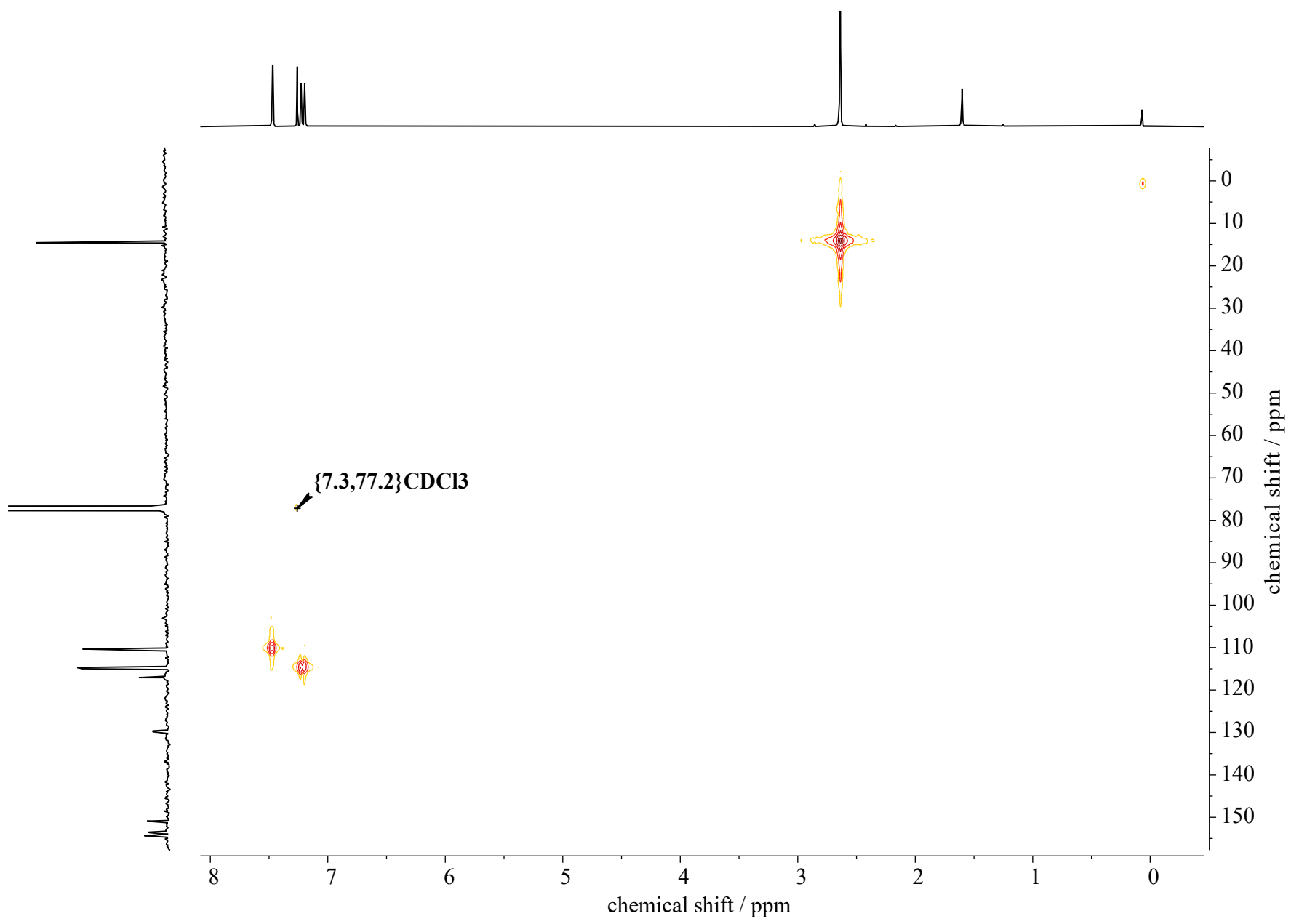


**Figure S46**. COSY and HMQC NMR spectra of **31** in $CDCl_3$.

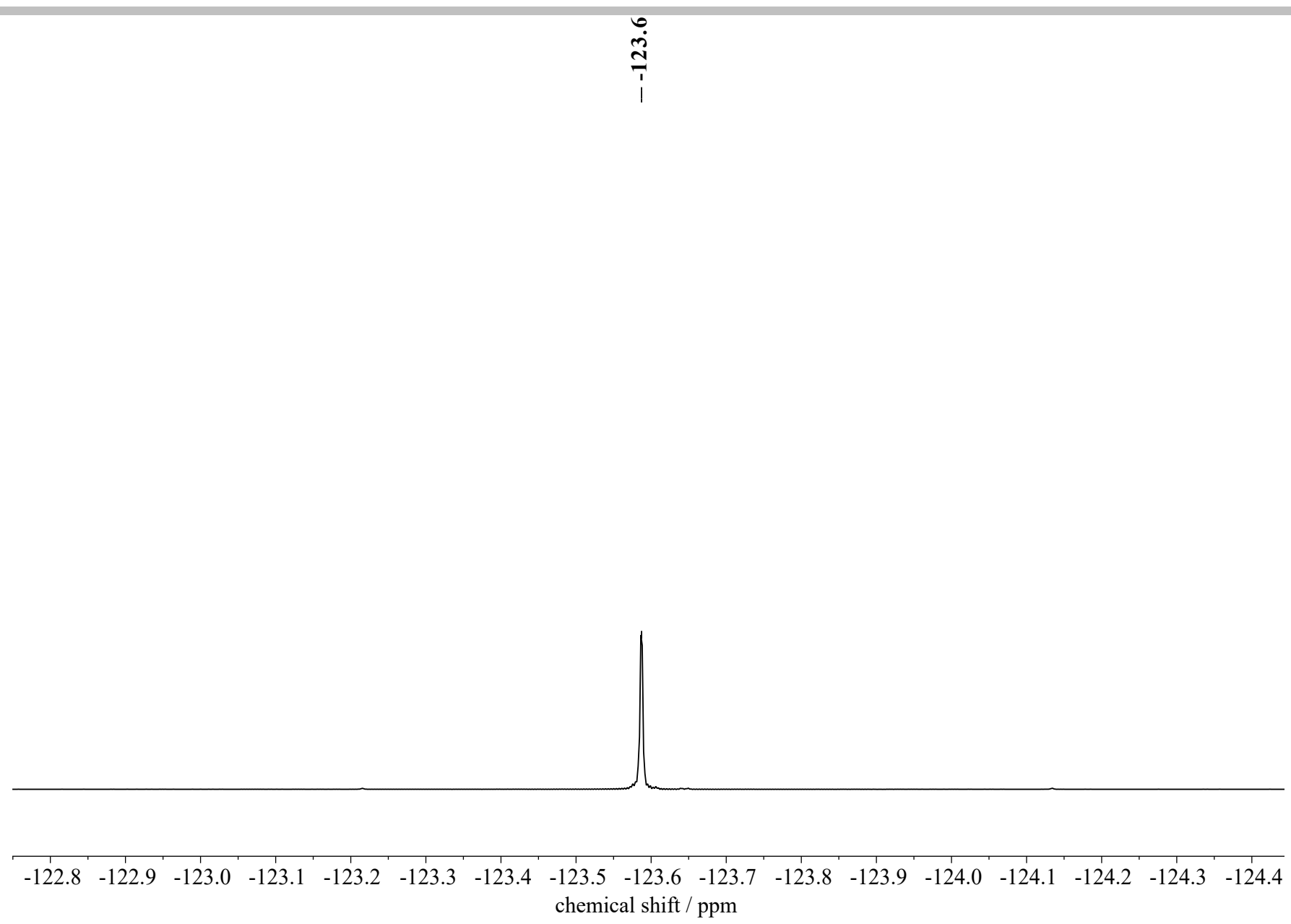


**Figure S47**. $^{19}F$ (282 MHz) NMR spectrum of **31** in $CDCl_3$.

**Synthesis of N-(4-fluoro-2-methylbenzo[d]oxazol-6-yl)-1,1-diphenylmethanimine (33)**

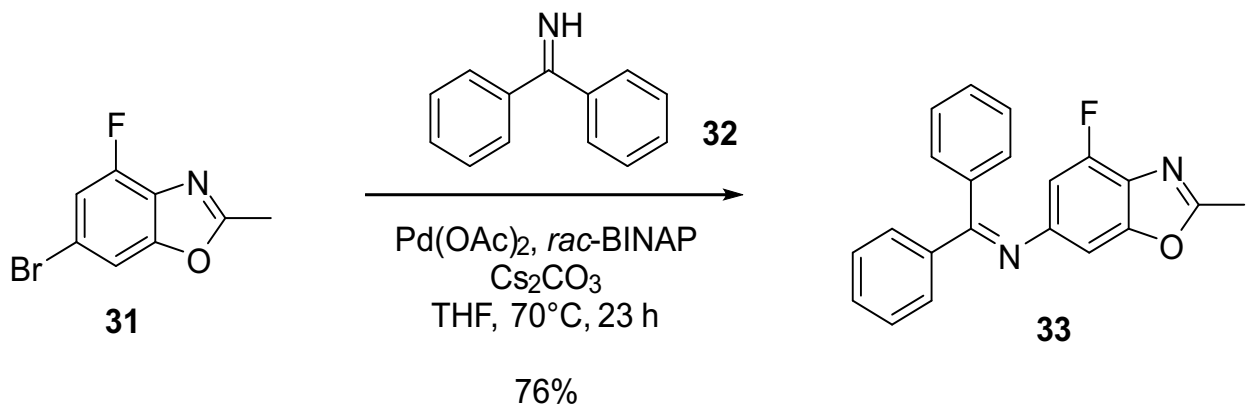


4-Fluoro-2-methyl-1,3-benzoxazol-6-amine **31** (300 mg, 1.30 mmol, 1.00 eq), palladium acetate (30 mg, 130 µmol, 0.10 eq), *rac*-BINAP (82 mg, 130 µmol, 0.10 eq), diphenylmethanimine **32** (308 mg, 1.30 mmol, 1.00 eq) and cesium carbonate (1.06 g, 3.26 mmol, 2.50 eq) were suspended in 15 mL THF under nitrogen atmosphere. The reaction mixture was heated to 70 °C and stirred for 23 h. The mixture was cooled to room temperature and diluted with 200 mL distill distilled water and extracted with 200 mL EtOAc. The organic layer was dried over $MgSO_4$ and solvent was removed under reduced pressure. The crude product was purified by flash column chromatography (*n*-pentane/EtOAc, 10:1, v%) to yield the desired compound *N*-(4-fluoro-2-methylbenzo[*d*]oxazol-6-yl)-1,1-diphenylmethanimine (**33**, 326 mg, 988 µmol, 76%) as a yellow solid.

**TLC:** $R_f$ = 0.18 (*n*-pentane/EtOAc, 10:1, v%).

**$^1$H-NMR (300 MHz, DMSO-*d*$_6$, *δ*):** 7.74-7.66 (m, 2H, $H_{arom.}$), 7.56-7.45 (m, 3H, $H_{arom.}$), 7.34-7.31 (m, 3H, $H_{arom.}$), 7.22-7.19 (m, 2H, $H_{arom.}$), 6.87 (d, $^4J$ = 1.6 Hz, 1H, $H_{arom.}$), 6.63 (dd, $^3J_{HF}$ = 11.3 Hz, $^4J$ = 1.6 Hz, 1H, $H_{arom.}$), 2.53 (s, 3H, C$H_3$) ppm.

**$^{13}$C-NMR (75 MHz, DMSO-*d*$_6$, *δ*):** 169.2 (N=*C*), 163.6 (N=*C*-O), 152.5 (d, $^3J_{FC}$ = 9.5 Hz, $C_{arom.}$), 151.2 (d, $^1J_{FC}$ = 250.6 Hz, *C*F), 149.3 (d, $^3J_{FC}$ = 8.6 Hz, $C_{arom.}$), 138.3 ($C_{arom.}$), 135.4 ($C_{arom.}$), 131.3 ($C_{arom.}$H), 128.9 (2C, 2x$C_{arom.}$H), 128.7 ($C_{arom.}$H), 128.7 (2C, 2x $C_{arom.}$H), 128.4 (2C, 2x$C_{arom.}$H), 128.1 (2C, 2x$C_{arom.}$H), 124.7 (d, $^2J_{FC}$ = 17.0 Hz, $C_{arom.}$), 104.2 (d, $^2J_{FC}$ = 19.5 Hz, $C_{arom.}$H), 98.8 (d, $^4J_{FC}$ = 4.2 Hz, $C_{arom.}$H), 13.9 (*C*$H_3$) ppm.

**$^{19}$F-NMR (282 MHz, DMSO-*d*$_6$, *δ*)**: -126.9 (C*F*) ppm.

**HRMS-ESI (m/z):** $[M+H]^+$ calcd for $C_{21}H_{16}FN_2O$, 331.1241; found, 331.1263.

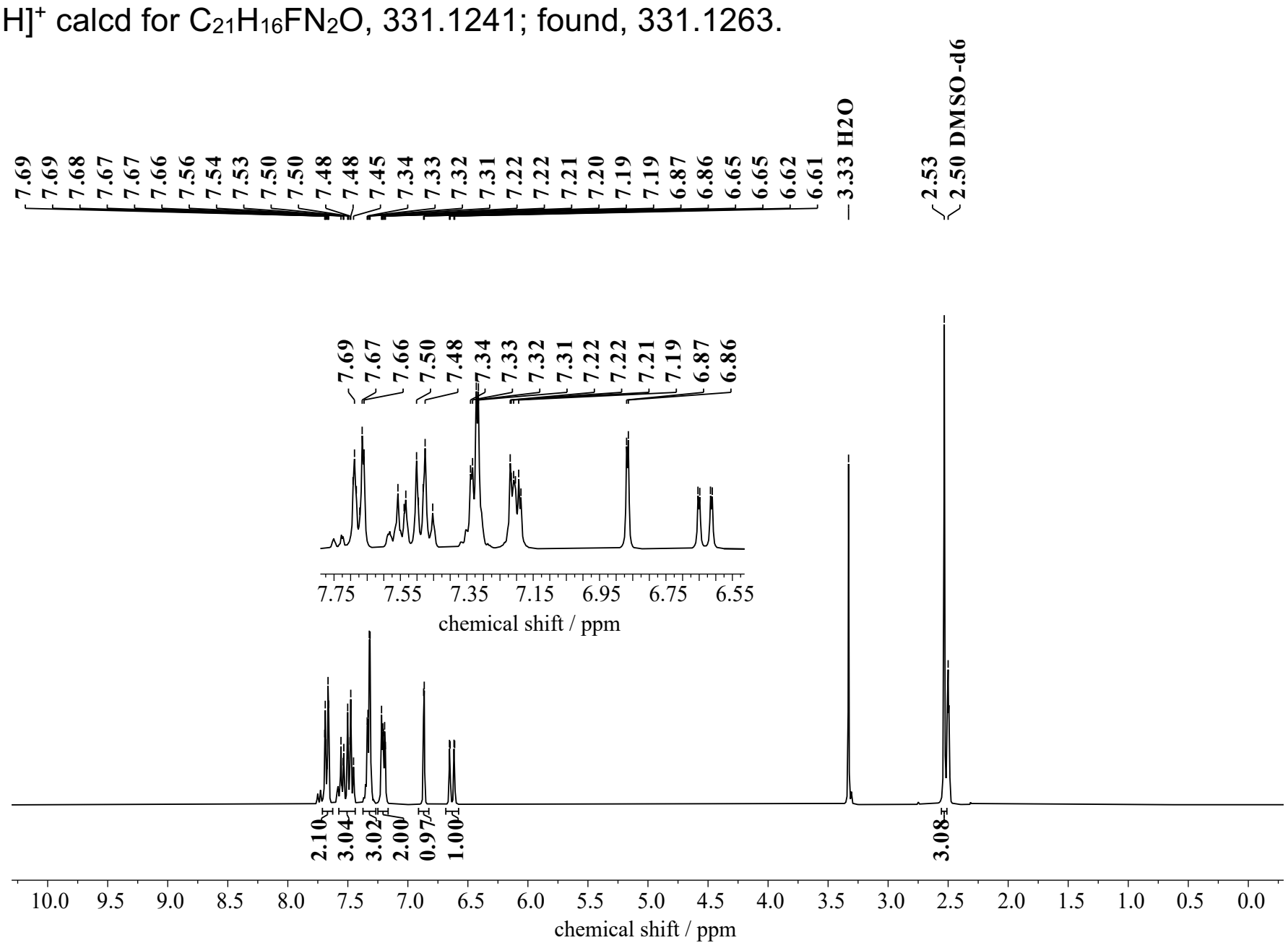

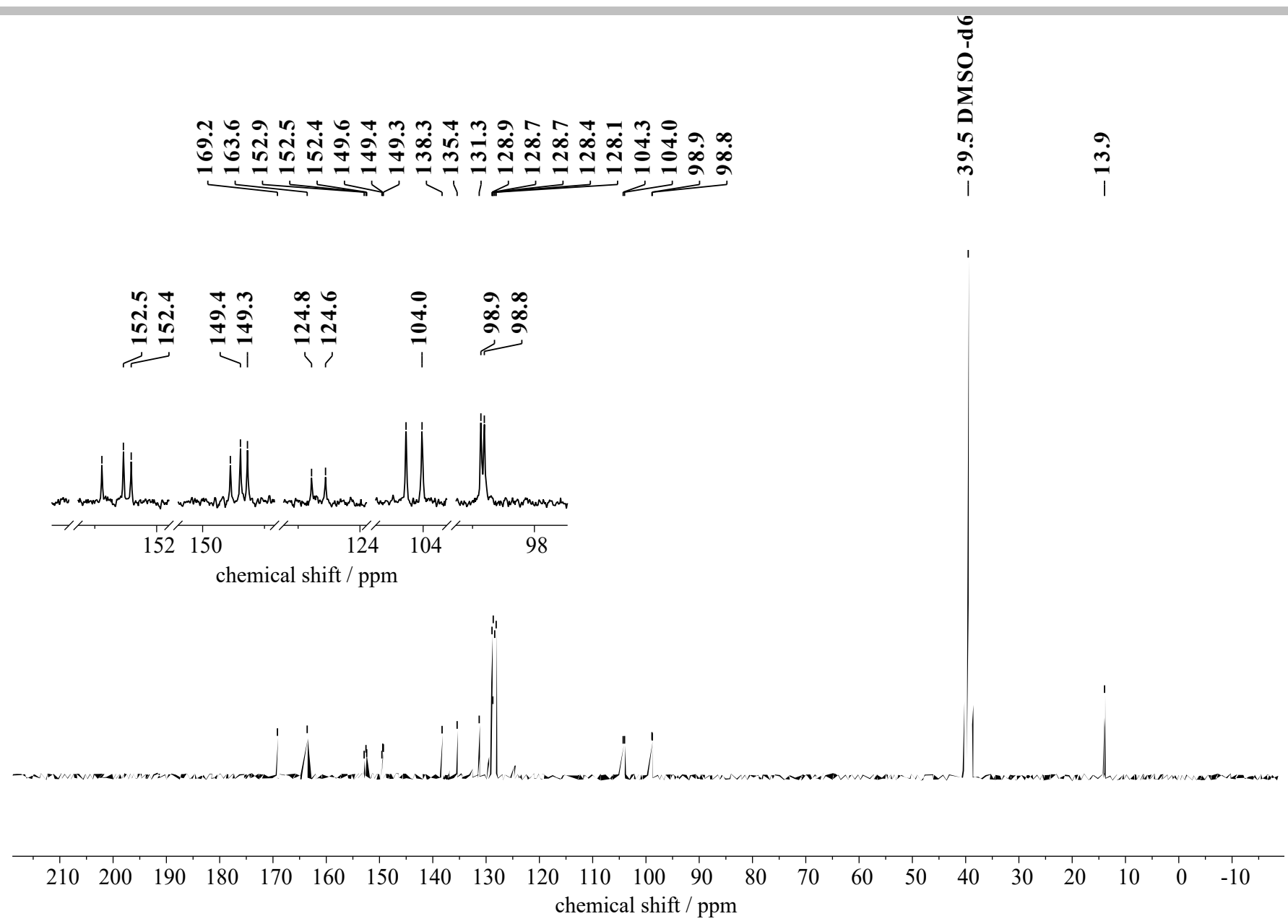


**Figure S48**. 1H (300 MHz) and 13C (75 MHz) NMR spectra of **33** in DMSO-$d_6$.

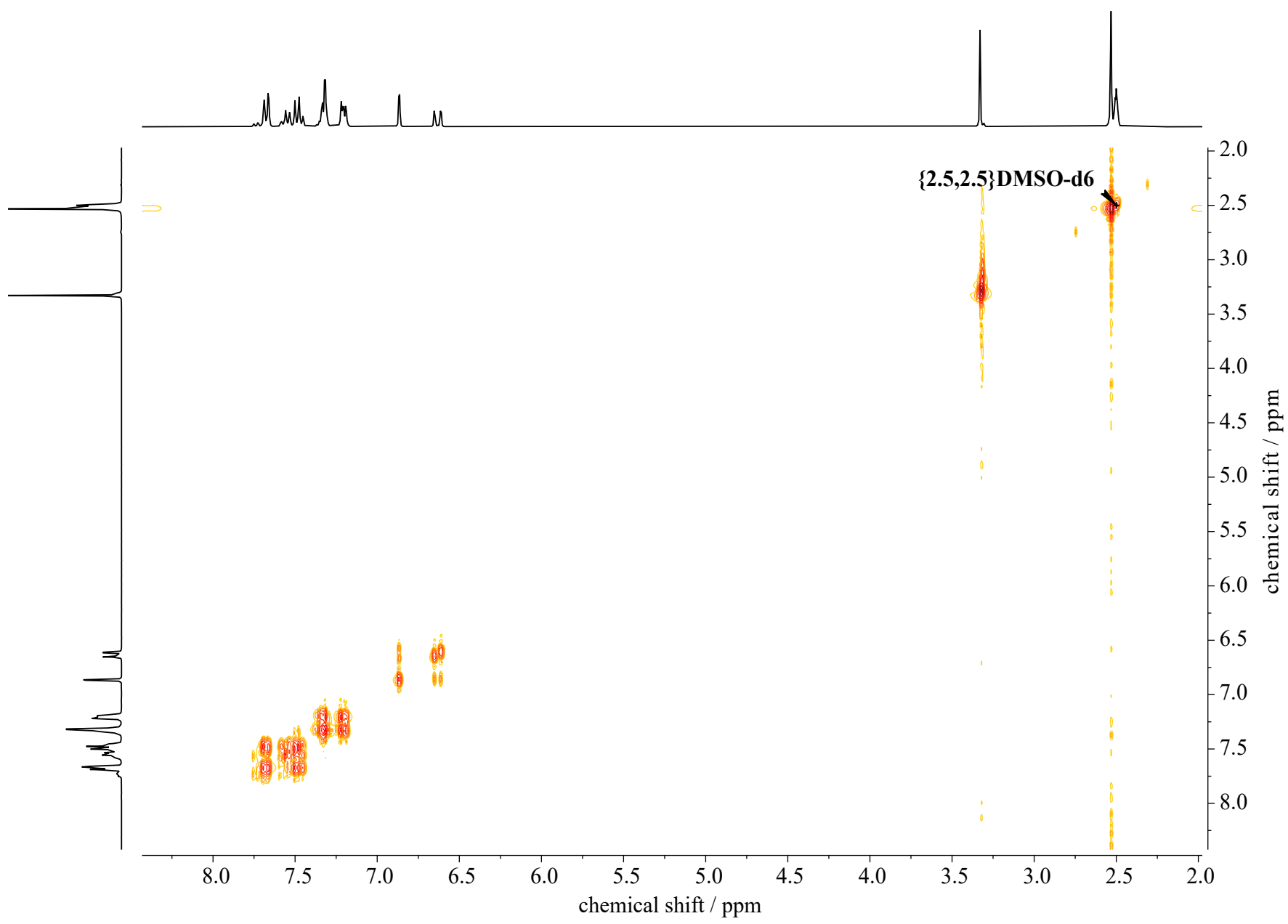

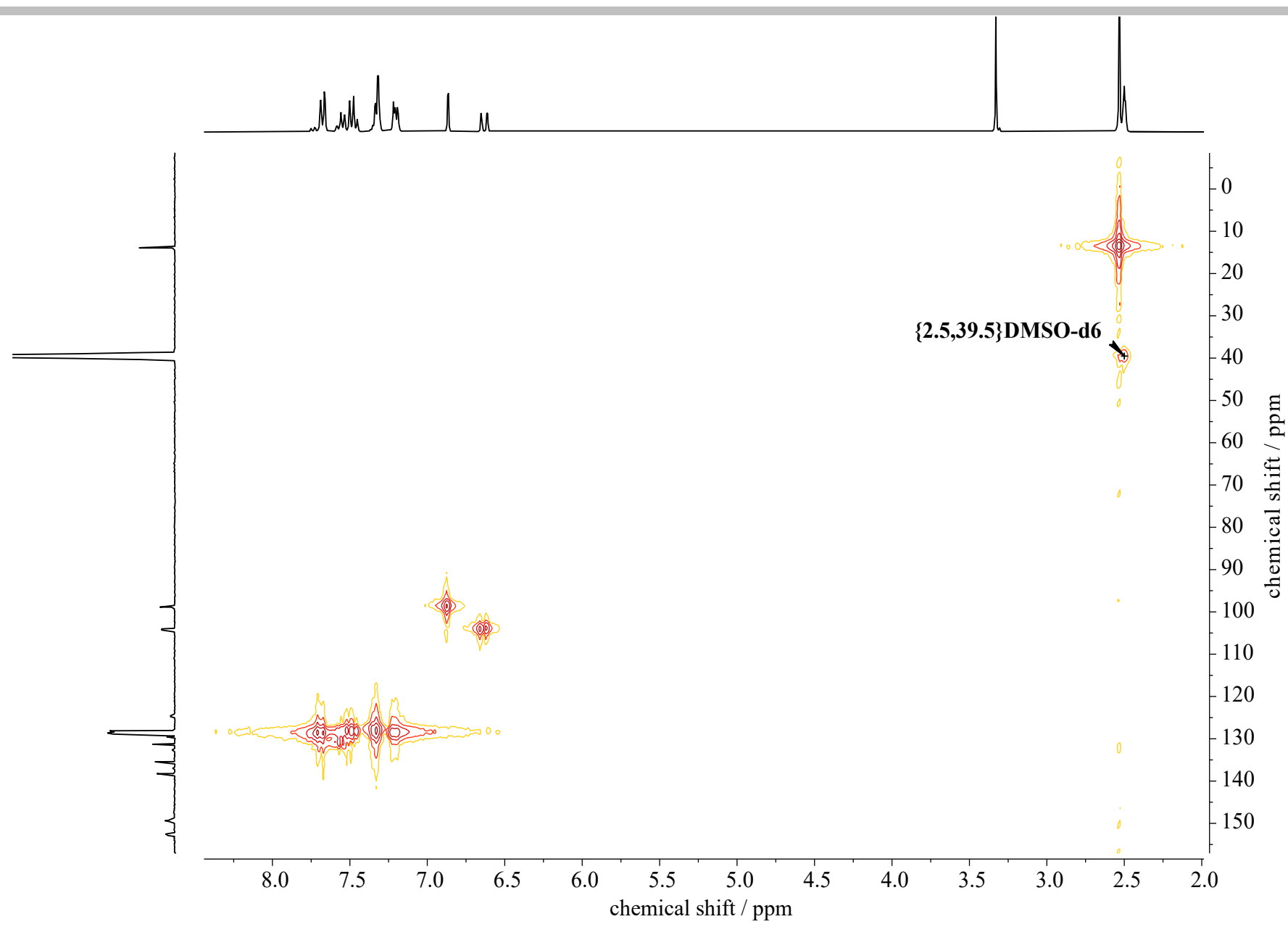


**Figure S49**. COSY and HMQC NMR spectra of **33** in DMSO-$d_6$.

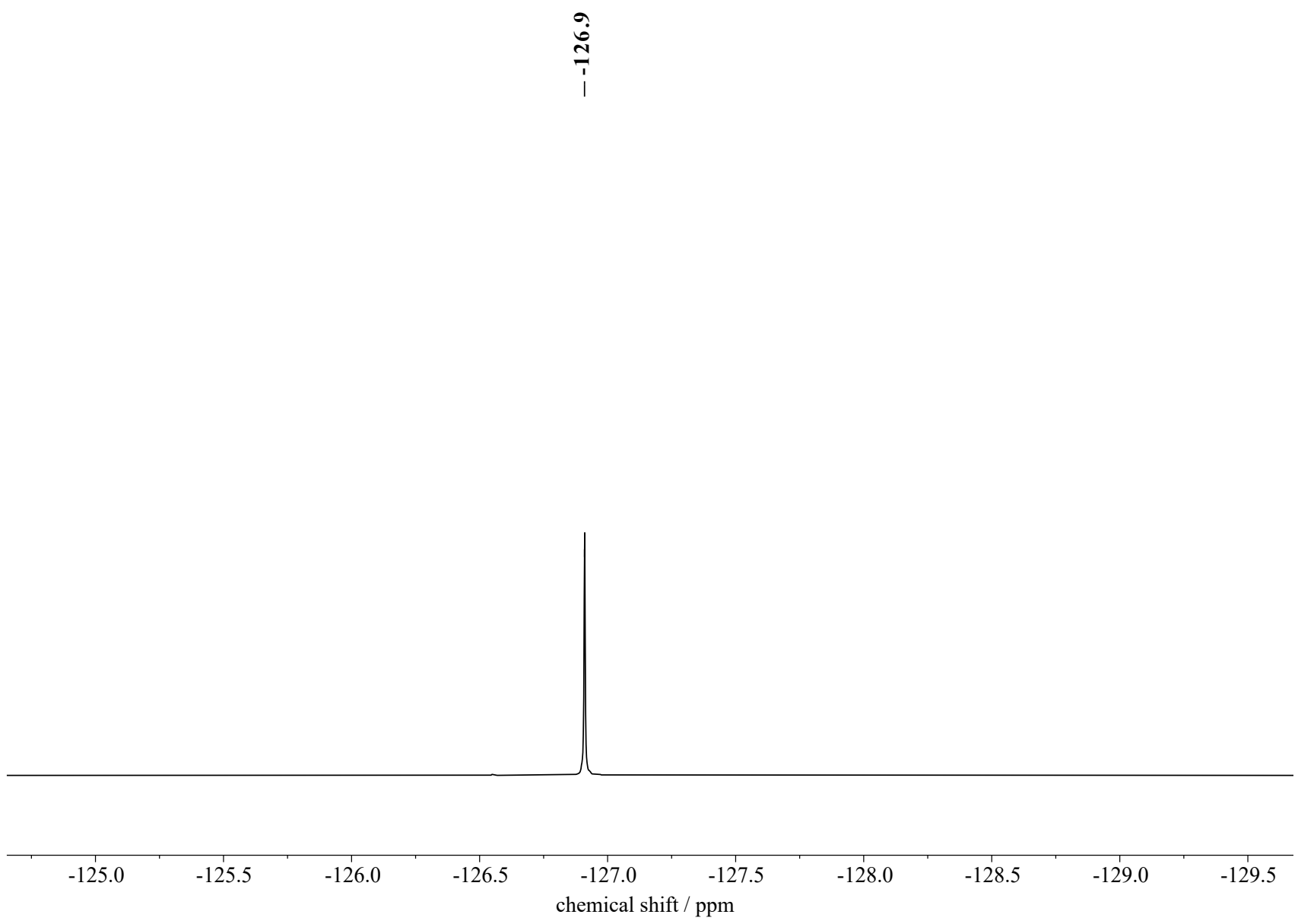


**Figure S50**. $^{19}$F (282 MHz) NMR spectra of **33** in DMSO-$d_6$.

**Synthesis of 4-fluoro-2-methylbenzo[d]oxazol-6-amine (34)**

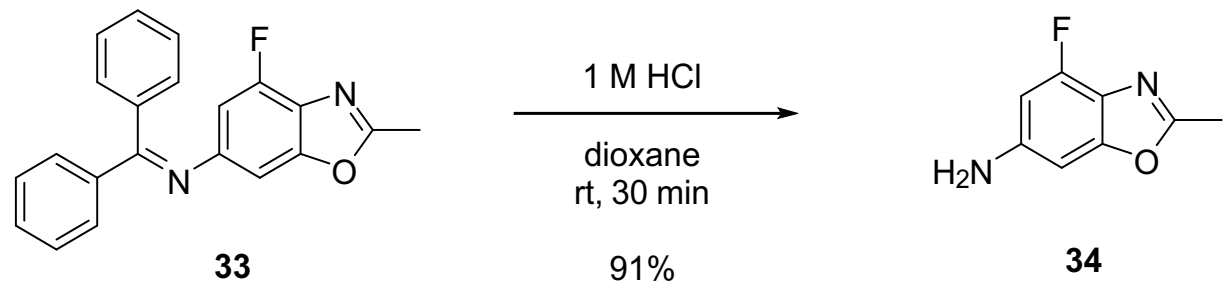


*N*-(4-fluoro-2-methylbenzo[*d*]oxazol-6-yl)-1,1-diphenylmethanimine **33** (327 mg, 988 µmol, 1.00 eq) was dissolved in 10 mL dioxane at room temperature. 1 M HCl (1.24 mL, 1.24 mmol, 1.25 eq) was added dropwise. The reaction mixture was stirred at room temperature for 30 min. The solvent was removed under reduced pressure. The residue was washed with 100 mL acetonitrile yielding product 4-fluoro-2-methylbenzo[*d*]oxazol-6-amine as colorless solid (**34**, 149 mg, 899 µmol, 91%).

**TLC:** $R_f$ = 0.64 (*n*-pentane/EtOAc, 1:1, v%).

**$^{1}$H-NMR (300 MHz, DMSO-$d_6$, δ):** 6.54 (d, $^{4}J$ = 1.8 Hz, 1H, $H_{arom.}$), 6.37 (dd, $^{3}J_{HF}$ = 12.7 Hz, $^{4}J$ = 1.8 Hz, 1H, $H_{arom.}$), 5.50 (s, 2H, N$H_2$), 2.48 (s, 3H, C$H_3$) ppm.

**$^{13}$C-NMR (75 MHz, DMSO-$d_6$, δ):** 160.4 (N=*C*-O), 153.9 (d, $^{3}J_{FC}$ = 10.6 Hz, $C_{arom.}$), 150.5 ($C_{arom.}$), 147.9 (d, $^{3}J_{FC}$ = 10.5 Hz, $C_{arom.}$), 119.0 ($C_{arom.}$), 97.4 (d, $^{2}J_{FC}$ = 20.6 Hz, $C_{arom.}$H), 90.6 (d, $^{4}J_{FC}$ = 4.2 Hz, $C_{arom.}$H), 13.8 ($C$H$_3$) ppm.

**$^{19}$F-NMR (282 MHz, DMSO-$d_6$, δ)**: -129.7 (C*F*) ppm.

**HRMS-ESI (m/z):** $[M+H]^+$ calcd for $C_8H_8FN_2O$, 167.0615; found, 167.0616.

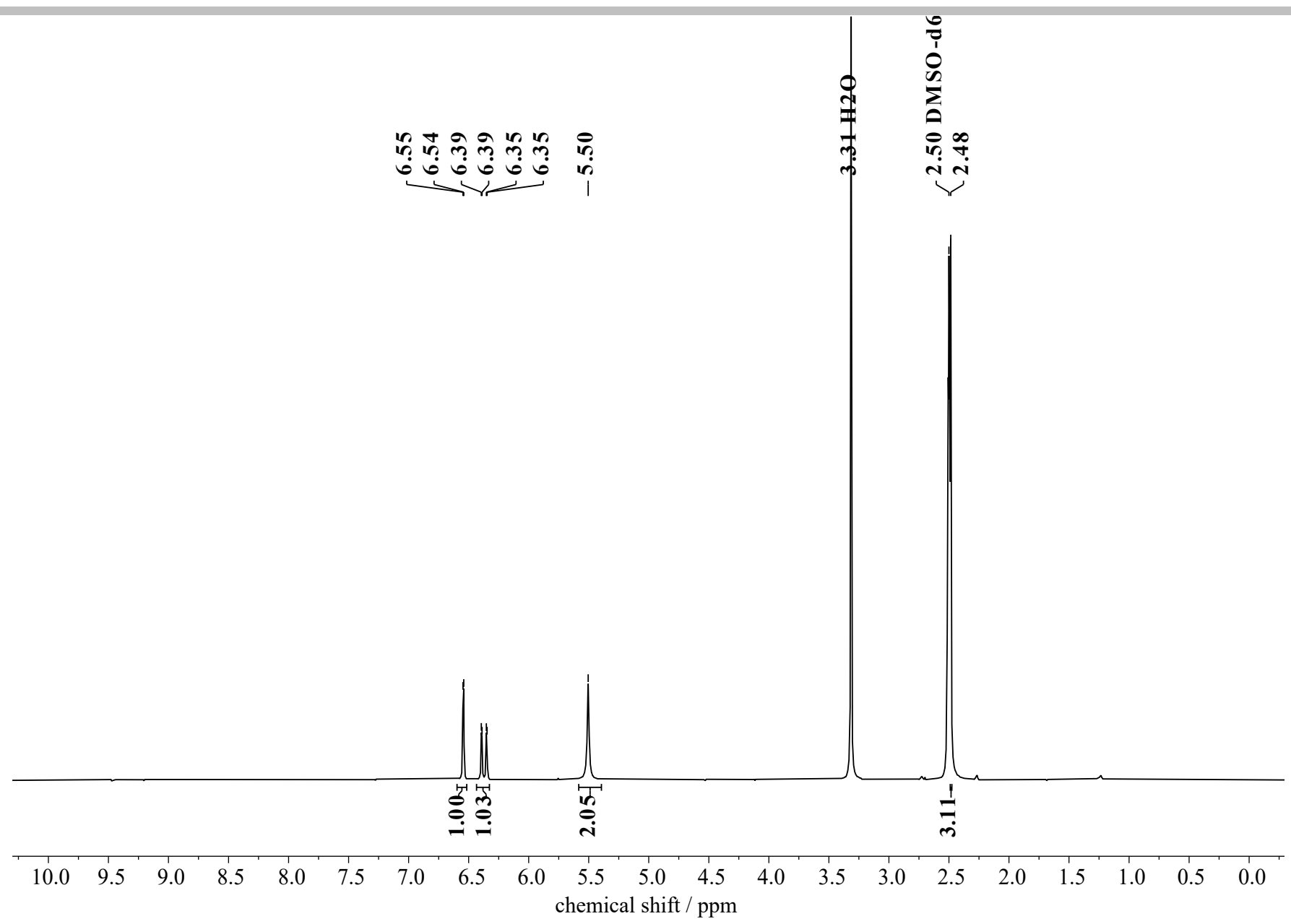

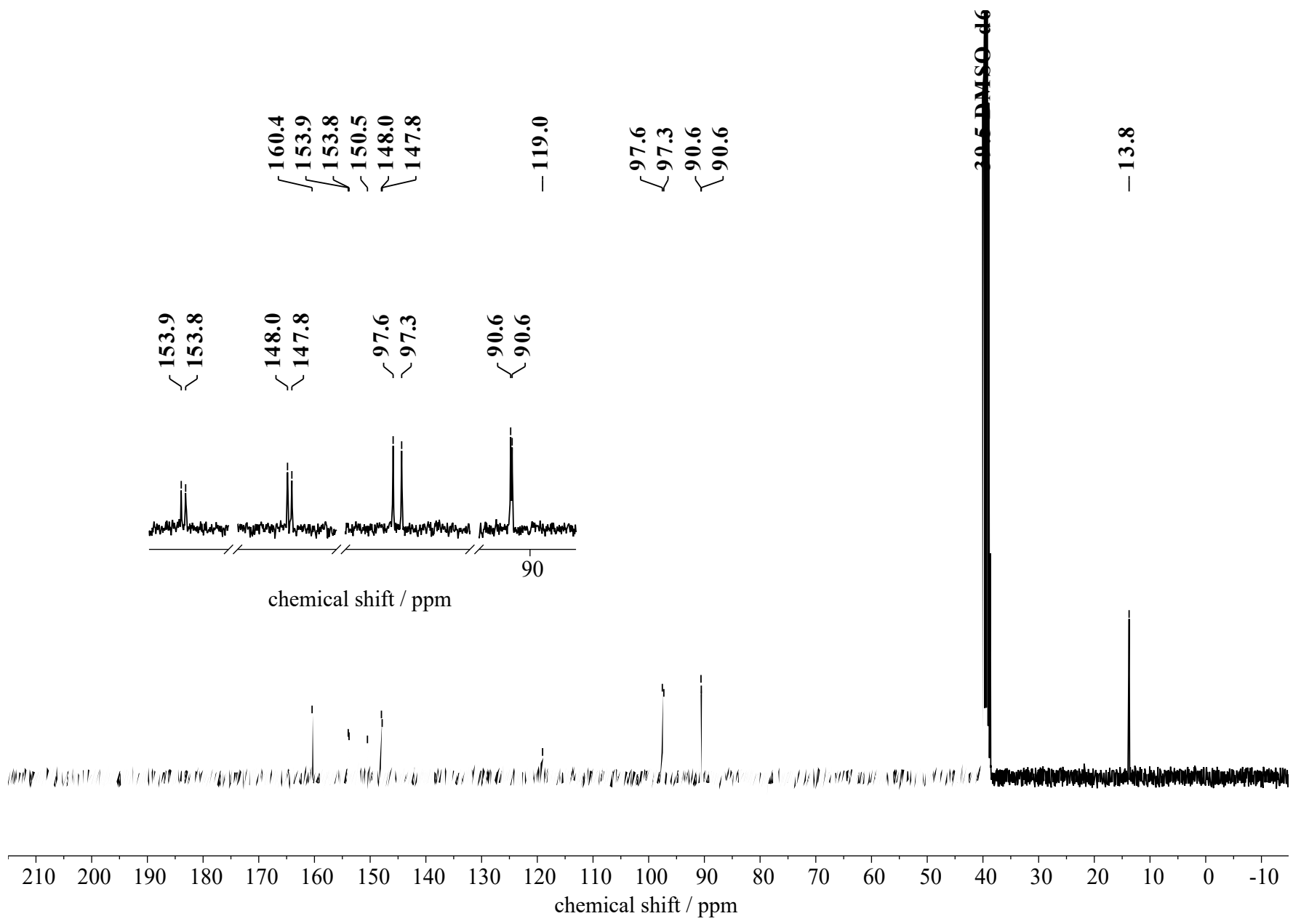

**Figure S51**. $^1$H (300 MHz) and $^{13}$C (75 MHz) NMR spectra of **34** in DMSO-$d_6$.

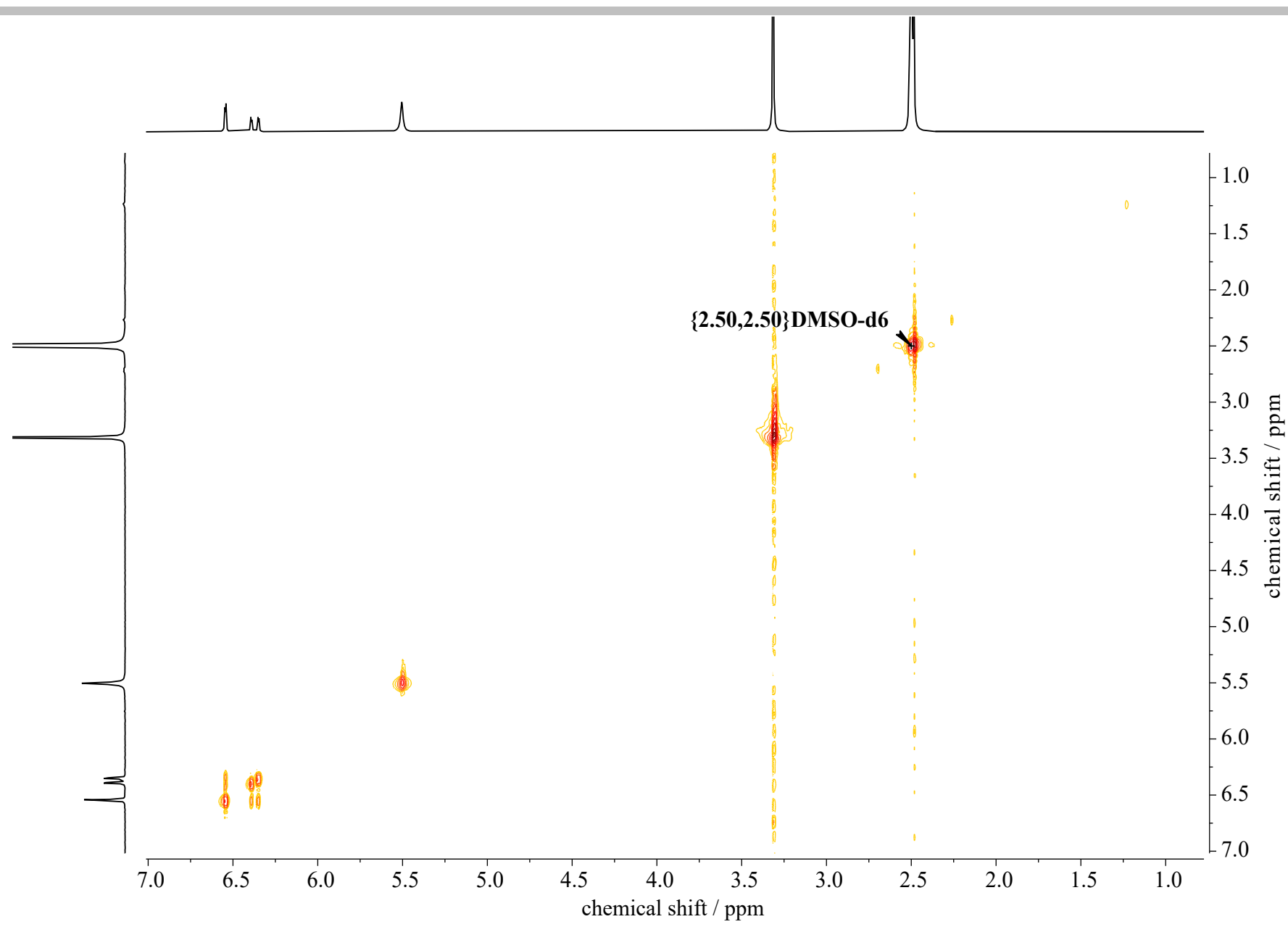


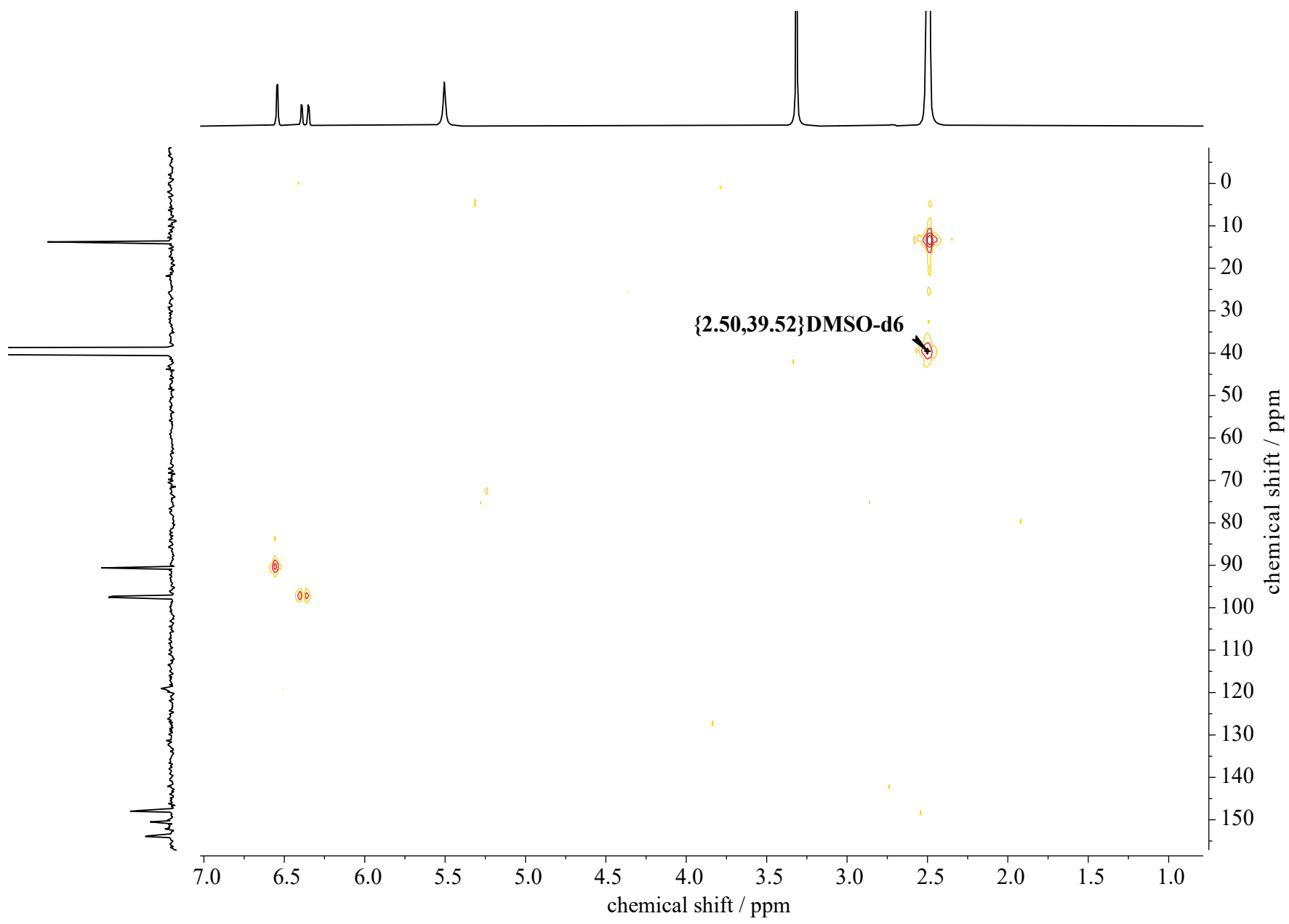


**Figure S52**. COSY and HMQC NMR spectra of **34** in DMSO-$d_6$.

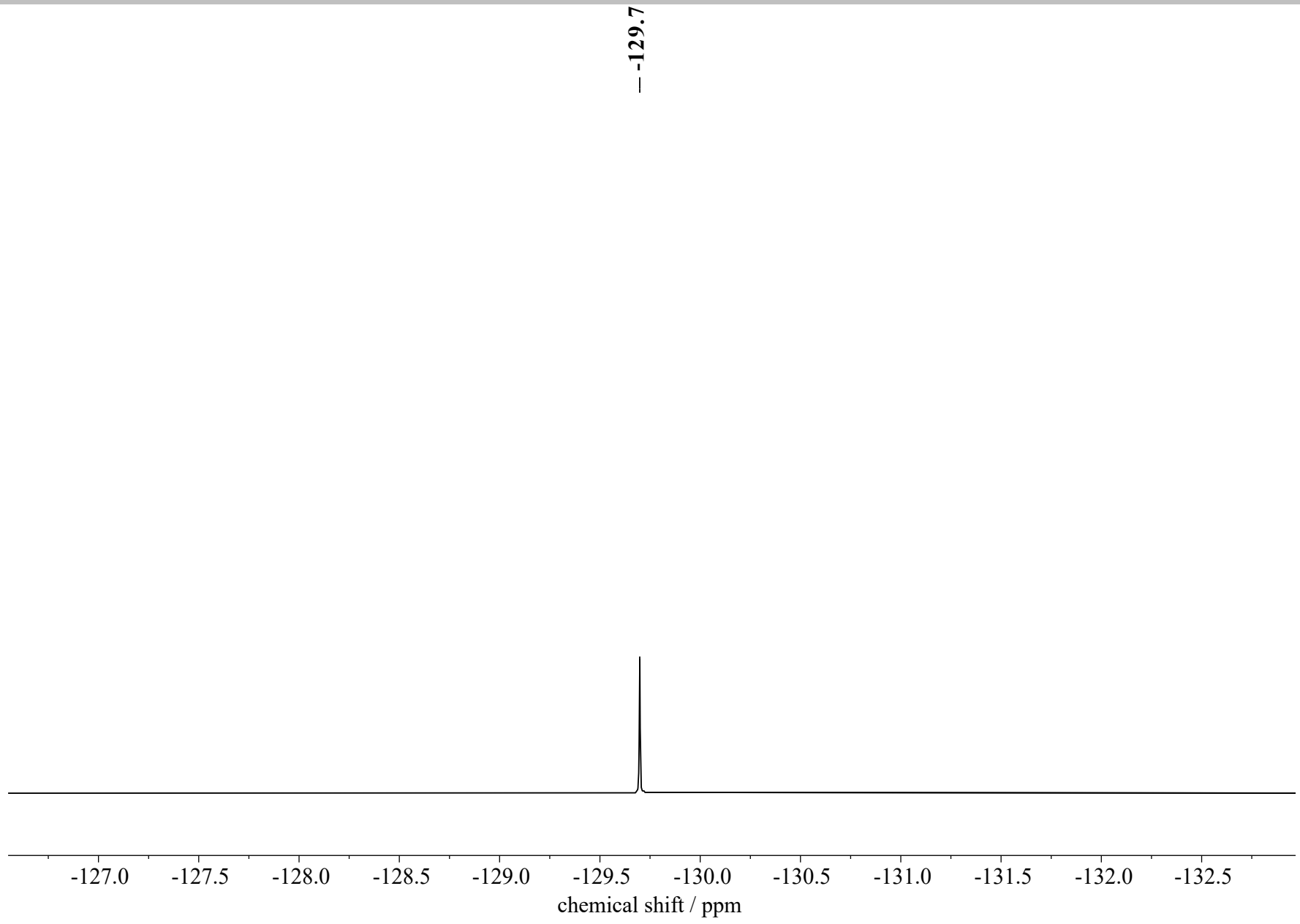


**Figure S53**. $^{19}F$ (282 MHz) NMR spectra of **34** in DMSO-$d_6$.

**Synthesis of 6-((4-bromo-2-fluorophenyl)diazenyl)-4-fluoro-2-methylbenzo[d]oxazole (35)**

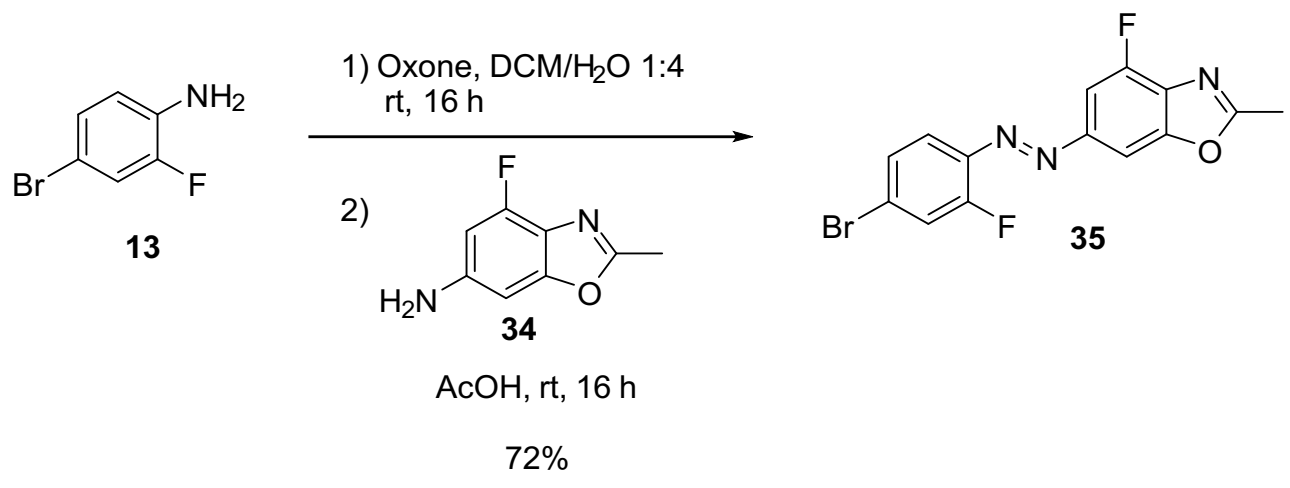


4-Bromo-2-fluoroaniline **13** (570 mg, 3.00 mmol, 2.00 eq) was dissolved in 10 mL DCM, Oxone® (1.84 g, 6.00 mmol, 4.00 eq) was dissolved in 40 mL distilled water and added to the solution. The resulting mixture was stirred for 16 h at room temperature. The reaction mixture was then diluted with 100 mL DCM and washed with 100 mL 1 M HCl, distilled water and brine. The organic layer was dried over $MgSO_4$ and the solvent was removed under reduced pressure. The resulting brown solid and 4-fluoro-2-methyl-1,3-benzoxazol-6-amine **34** (250 mg, 1.51 mmol, 1.00 eq) were suspended in 20 mL acetic acid. The reaction mixture was stirred for 16 h at room temperature. The reaction was then diluted with 200 mL distilled water and extracted with 200 mL EtOAc. The organic layer was washed with 200 mL distilled water and brine, dried over $MgSO_4$ and solvent was removed under reduced pressure. The crude product was purified by flash column chromatography (EtOAc) to yield the desired compound 6-((4-bromo-2-fluorophenyl)diazenyl)-4-fluoro-2-methylbenzo[*d*]oxazole (**35**, 380 mg, 1.08 mmol, 72%) as an orange solid.

**TLC:** $R_f$ = 0.57 (DCM).

***trans*-isomer** (*cis*-isomer signals were labeled with * on NMR spectrum).

**$^{1}$H-NMR (300 MHz, DMSO-*d*$_6$, *δ*):** 8.13 (d, $^{4}J$ = 1.6 Hz, 1H, $H_{arom.}$), 7.91 (dd, $^{3}J$ = 10.4 Hz, $^{4}J$ = 2.0 Hz, 1H, $H_{arom.}$), 7.73 (dd, $^{3}J$ = 10.8 Hz, $^{4}J$ = 1.6 Hz, 1H, $H_{arom.}$), 7.66 (d, $^{3}J$ = 7.9 Hz, 1H, $H_{arom.}$), 7.59 (dd, $^{3}J$ = 8.8 Hz, $^{4}J$ = 1.6 Hz, 1H, $H_{arom.}$), 2.71 (s, 3H, C$H_3$) ppm.

**$^{13}$C-NMR (75 MHz, DMSO-*d*$_6$, *δ*):** 160.7 (N=*C*-O), 157.3 (d, $^{1}J_{FC}$ = 262.3 Hz, *C*F), 152.7 (d, $^{3}J_{FC}$ = 8.8 Hz, $C_{arom.}$), 150.2 ($C_{arom.}$), 149.5 (d, $^{3}J_{FC}$ = 6.0 Hz, $C_{arom.}$), 138.6 (d, $^{3}J_{FC}$ = 6.8 Hz, $C_{arom.}$), 128.5 (d, $^{3}J_{FC}$ = 3.6 Hz, $C_{arom.}$H), 126.0 (d, $^{3}J_{FC}$ = 9.5 Hz, $C_{arom.}$H), 121.0 ($C_{arom.}$H), 120.7 ($C_{arom.}$H), 119.1 ($C_{arom.}$H), 104.6 (d, $^{2}J_{FC}$ = 19.4 Hz, $C_{arom.}$H), 103.7 (d, $^{3}J_{FC}$ = 4.7 Hz, $C_{arom.}$H), 14.4 (*C*$H_3$) ppm.

**$^{19}$F-NMR (282 MHz, DMSO-*d*$_6$, *δ*):** -121.4 (C*F*), -125.3 (C*F*) ppm.

**HRMS-ESI (m/z):** $[M+H]^+$ calcd for $C_{14}H_9BrF_2N_3O$, 353.9872; found, 353.9871.

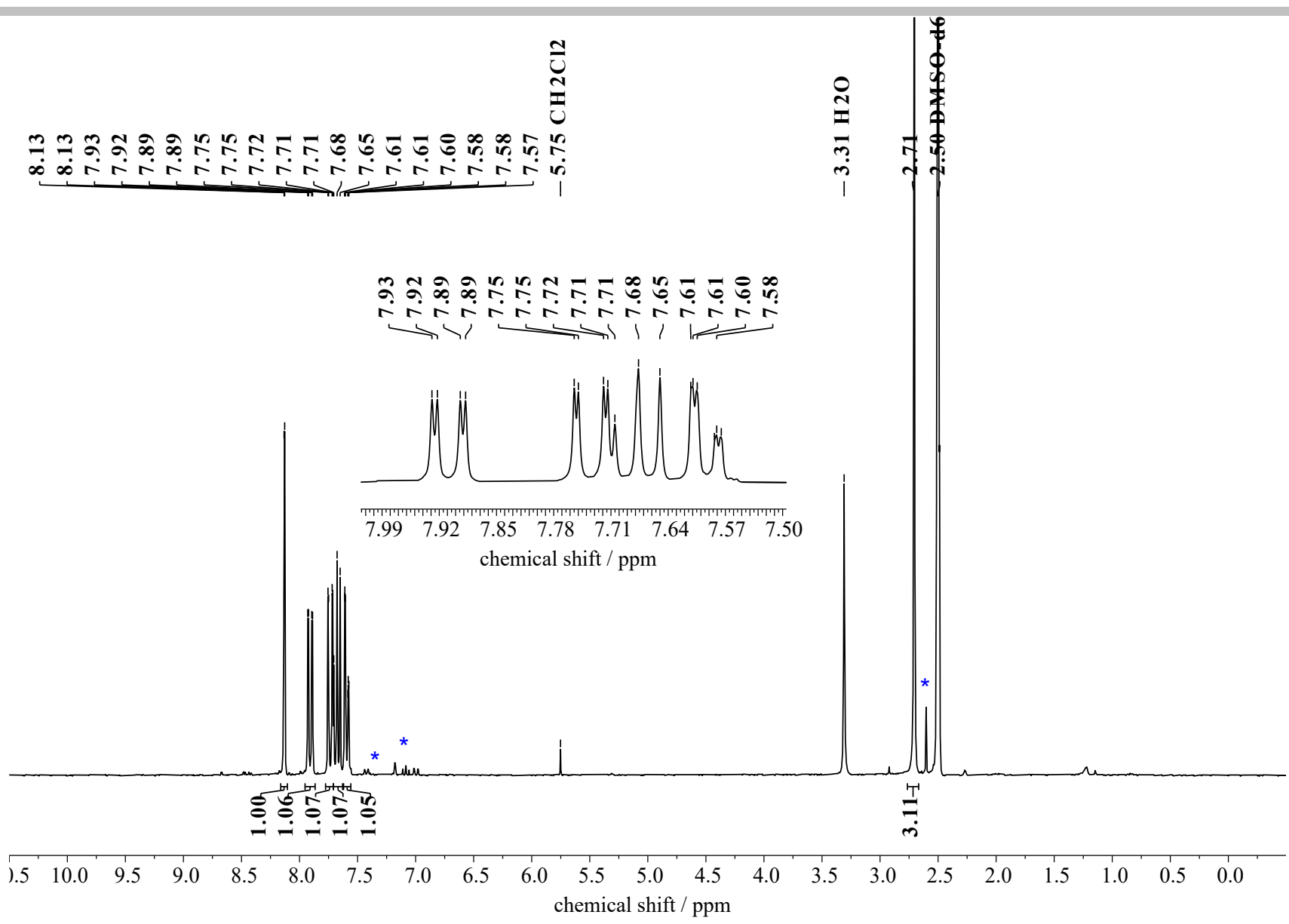


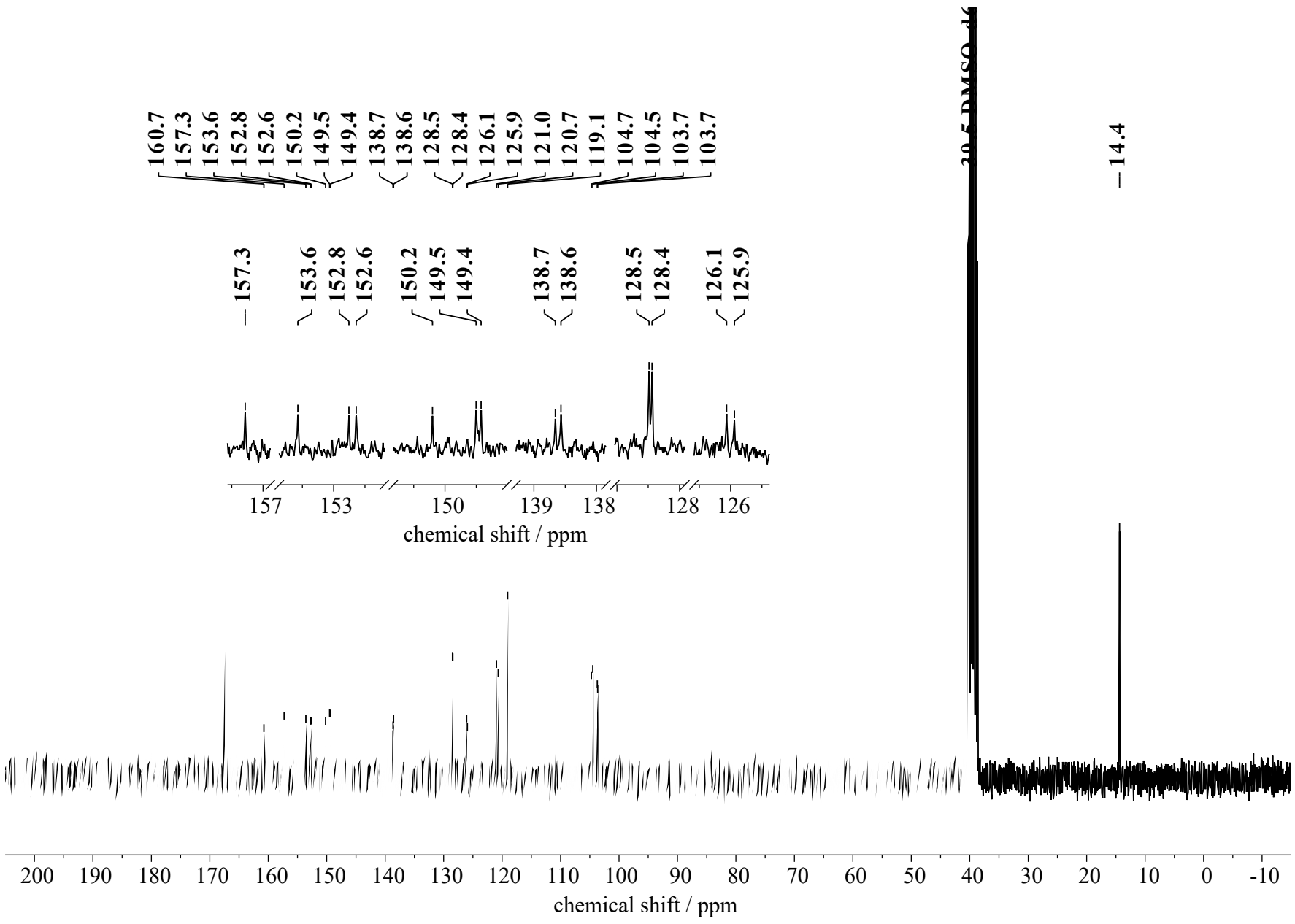


**Figure S54**. $^{1}H$ (300 MHz) and $^{13}C$ (75 MHz) NMR spectra of **35** in DMSO-$d_6$.

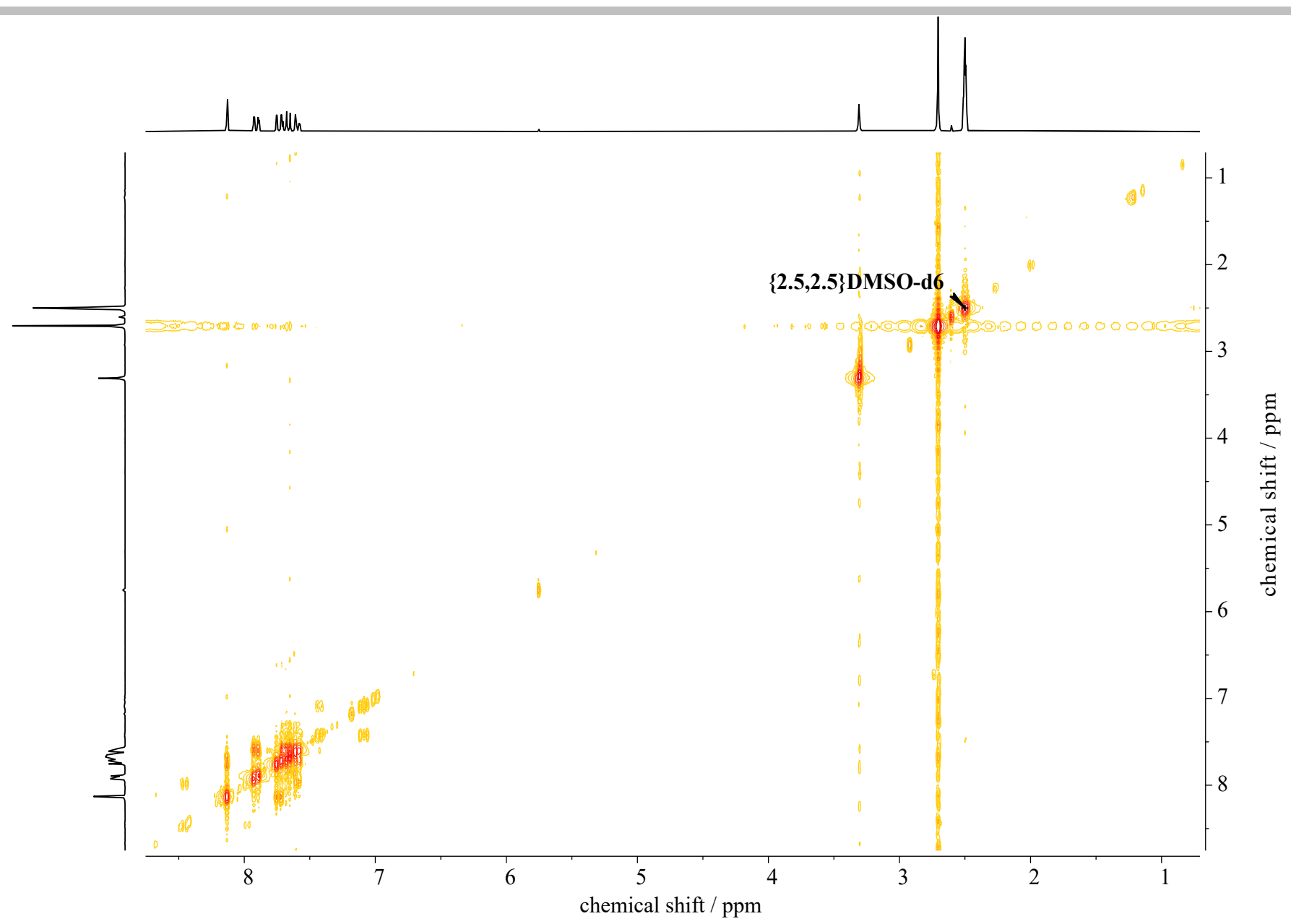


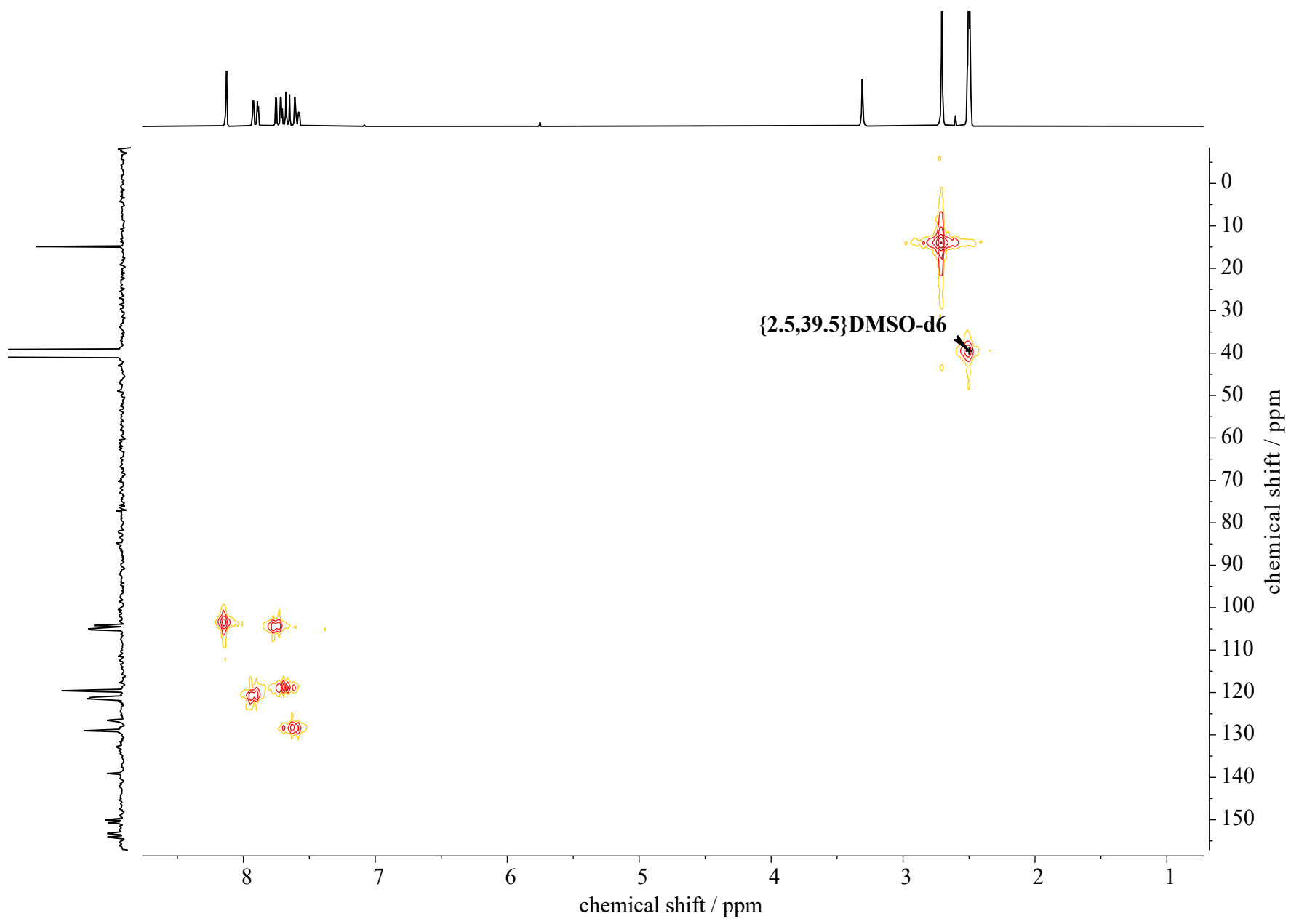


**Figure S55**. COSY and HMQC NMR spectra of **35** in DMSO-$d_6$.

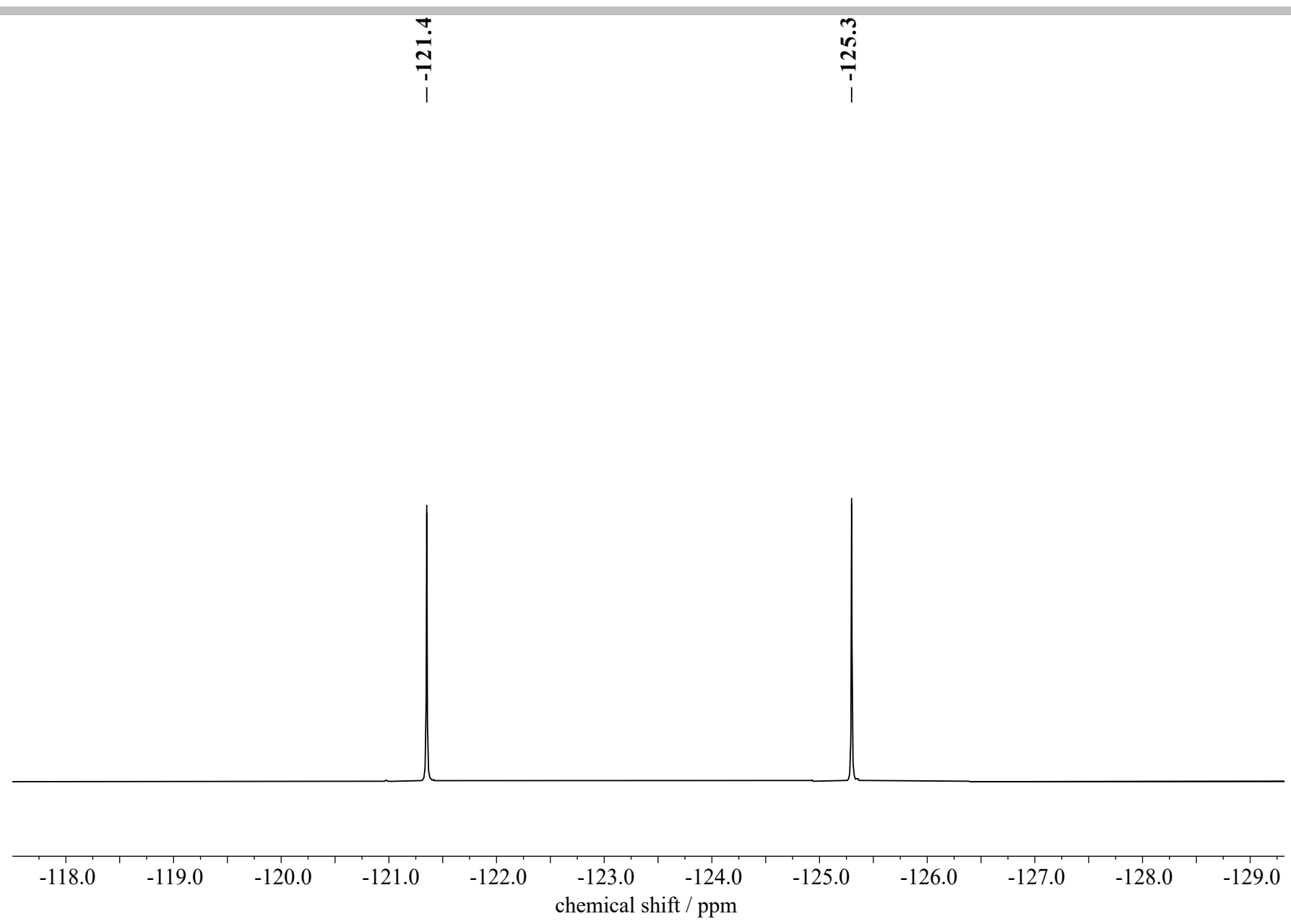


**Figure S56**. $^{19}$F (282 MHz) NMR spectra of **35** in DMSO-$d_6$.

**Synthesis of tert-butyl-4-(3-fluoro-4-((4-fluoro-2-methylbenzo[d]oxazol-6-yl)diazenyl)phenyl)-3,6-dihydropyridine-1(2H)-carboxylate (36)**

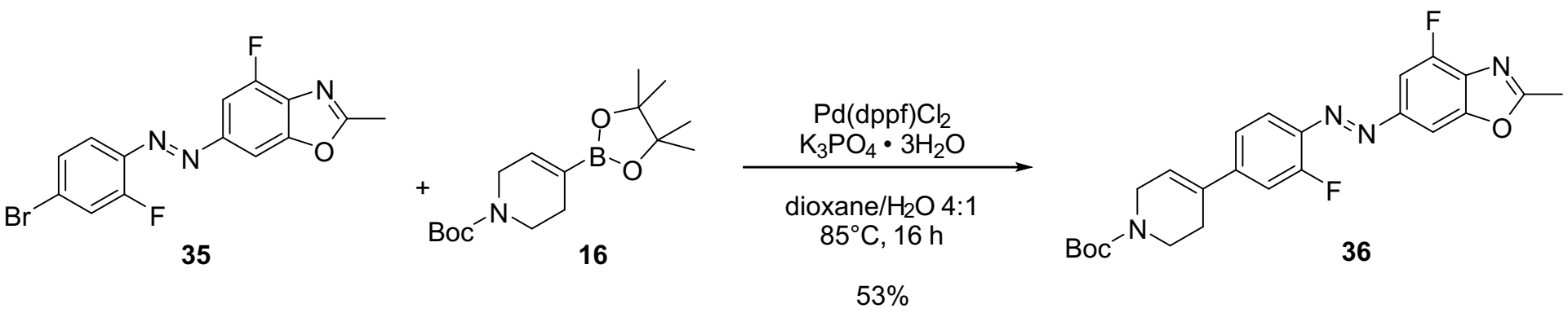


6-((4-Bromo-2-fluorophenyl)diazenyl)-4-fluoro-2-methylbenzo[*d*]oxazole **35** (150 mg, 426 µmol, 1.00 eq), *N-tert*-butoxycarbony-1,2,3,6-tetrahydropyridine-4-boronic acid pinacol ester **16** (132 mg, 426 µmol, 1.00 eq), Pd(dppf)$Cl_2$ (16 mg, 21 µmol, 0.05 eq) and $K_3PO_4 \cdot 3H_2O$ (295 mg, 1.28 mmol, 3.00 eq) were dissolved in mixture of dioxane/distilled water (20 mL, 4:1, v%). The reaction was stirred under $N_2$ atmosphere at 85 °C for 16 h. The reaction was then diluted with 100 mL distilled water and extracted with 100 mL EtOAc. The organic layer was washed with 100 mL distilled water and brine, dried over $MgSO_4$ and solvent was removed under reduced pressure. The crude product was purified by flash column chromatography (EtOAc/MeOH, 5:1, v%) to yield the desired compound *tert*-butyl-4-(3-fluoro-4-((4-fluoro-2-methylbenzo[*d*]oxazol-6-yl)diazenyl)phenyl)-3,6-dihydropyridine-1(*2H*)-carboxylate (**36**, 103 mg, 227 µmol, 53%) as an orange solid.

**TLC:** $R_f$ = 0.90 (EtOAc/MeOH, 5:1, v%).

**$^1$H-NMR (300 MHz, DMSO-$d_6$, δ):** 8.13 (d, $^4J$ = 1.5 Hz, 1H, $H_{arom.}$), 7.78-7.72 (m, 2H, $H_{arom.}$, $H_{arom.}$), 7.58 (dd, $^3J$ = 12.6 Hz, $^4J$ = 1.8 Hz, 1H, $H_{arom.}$), 7.48 (dd, $^3J$ = 8.6 Hz, $^4J$ = 1.9 Hz, 1H, $H_{arom.}$), 6.48 (s, 1H, C*H*=C), 4.06-4.05 (m, 2H, C$H_2$), 3.56 (t, $^3J$ = 5.7 Hz, 2H, C$H_2$), 2.71 (s, 3H, C$H_3$), 2.54-2.52 (m, 2H, C$H_2$), 1.44 (s, 9H, 3xC$H_3$) ppm.

**$^{13}$C-NMR (75 MHz, DMSO-$d_6$, δ):** 165.8 (N=*C*-O), 161.3 (*C*F), 158.0 (*C*F), 153.9 ($C_{arom.}$), 153.6 ($C_{arom.}$), 152.8 ($C_{arom.}$), 149.6 ($C_{arom.}$), 144.9 ($C_{arom.}$), 138.2 ($C_{arom.}$), 132.8 ($C_{q.}$), 124.9 (*C*H=C), 121.3 ($C_{arom.}$H), 117.5 ($C_{arom.}$H), 113.3 (d, $^2J_{FC}$ = 20.6 Hz, $C_{arom.}$H), 104.5 (d, $^2J_{FC}$ = 19.4 Hz, $C_{arom.}$H), 103.5 (d, $^3J_{FC}$ = 4.7 Hz, $C_{arom.}$H), 79.0 ($C_{q.}$), 43.3 ($CH_2$), 27.5 (3C, 3x$CH_3$), 25.9 ($CH_2$), 13.5 ($CH_3$) ppm.

**$^{19}$F-NMR (282 MHz, DMSO-$d_6$, δ):** -123.6 (C*F*), -125.4 (C*F*) ppm.

**HRMS-ESI (m/z):** $[M+Na]^+$ calcd for $C_{24}H_{24}F_2N_4O_3Na$, 477.1709; found, 477.1712.

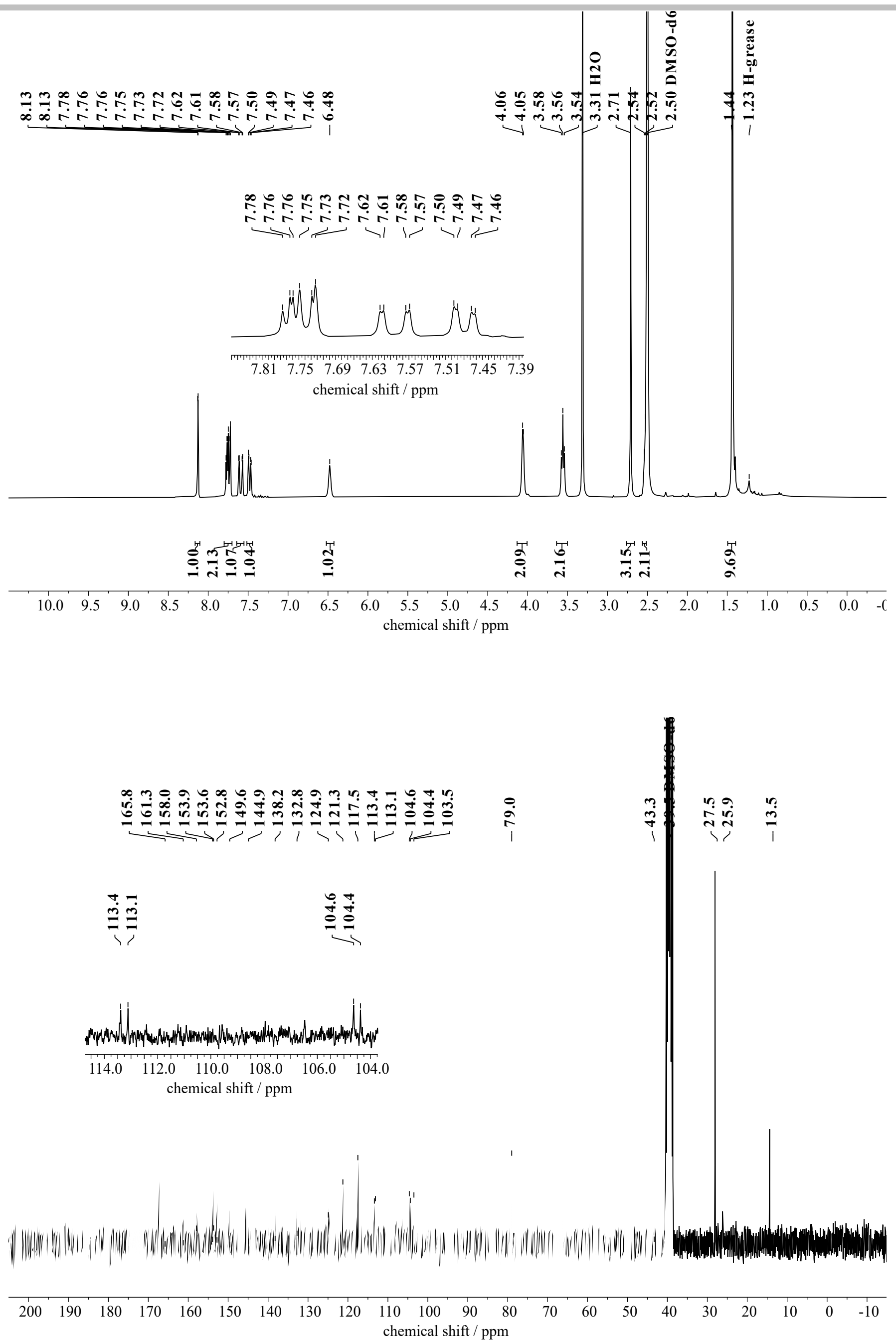

**Figure S57**. $^{1}H$ (300 MHz) and $^{13}C$ (75 MHz) NMR spectra of **36** in DMSO-$d_6$.

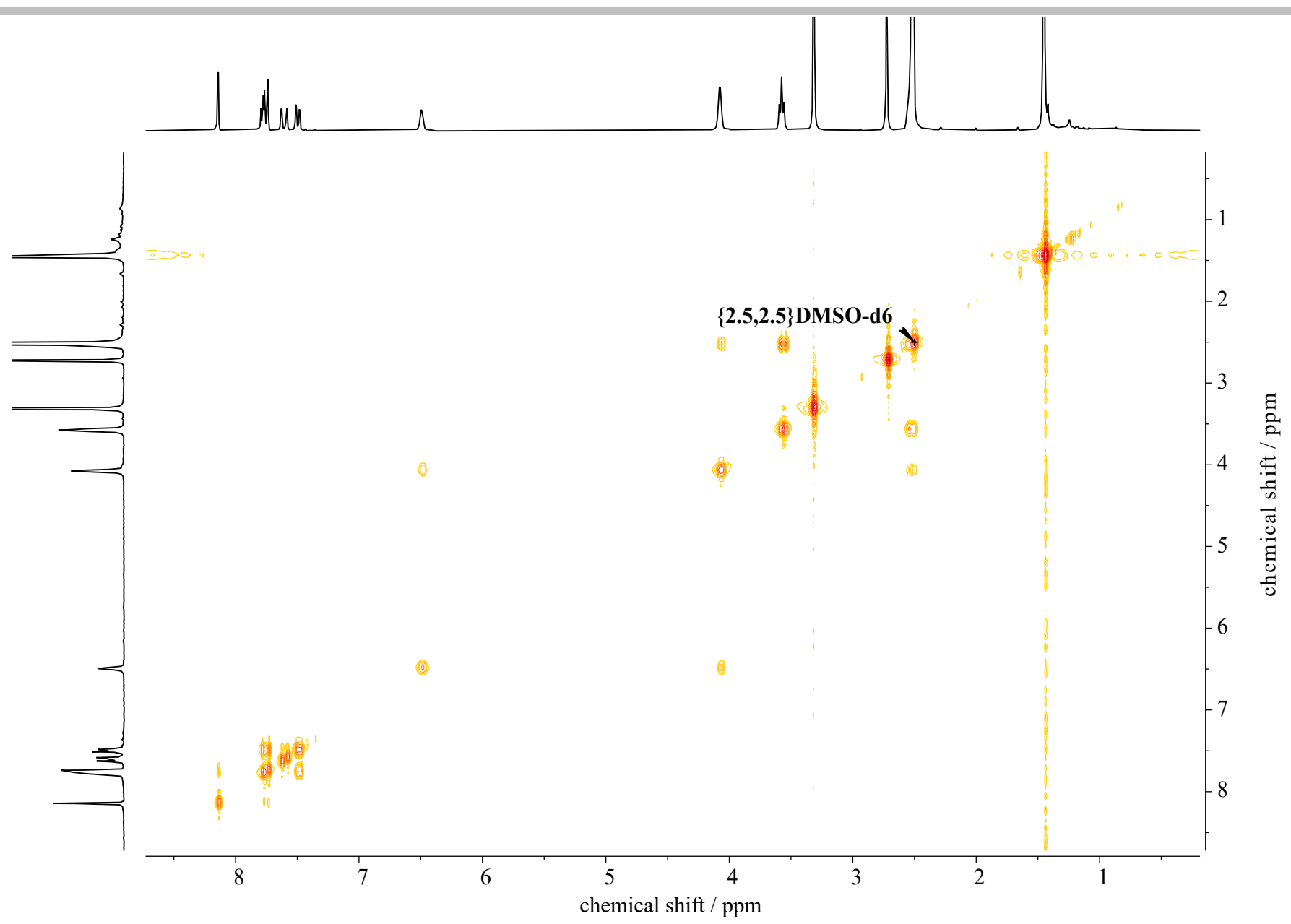


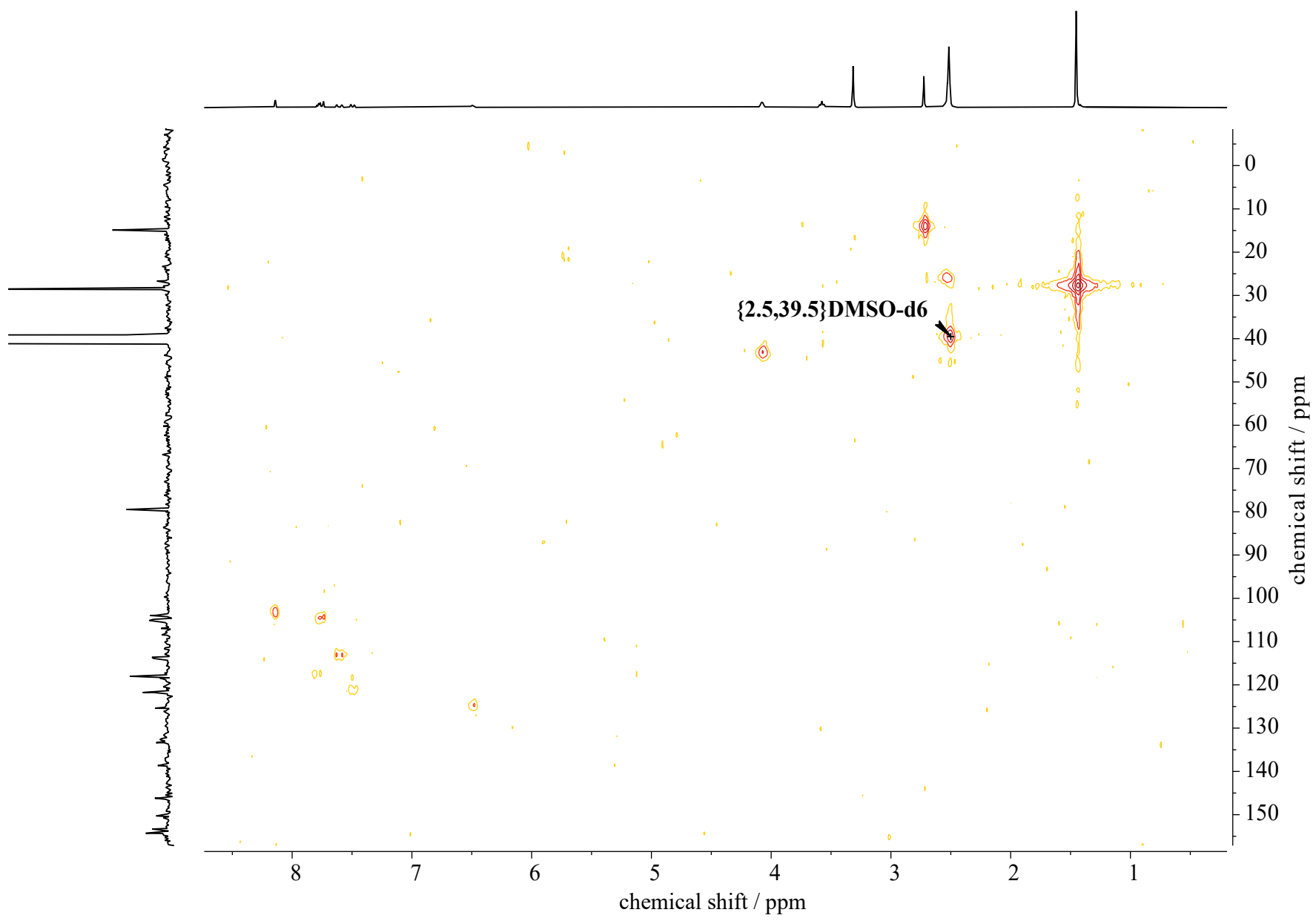


**Figure S58**. COSY and HMQC NMR spectra of **36** in DMSO-$d_6$.

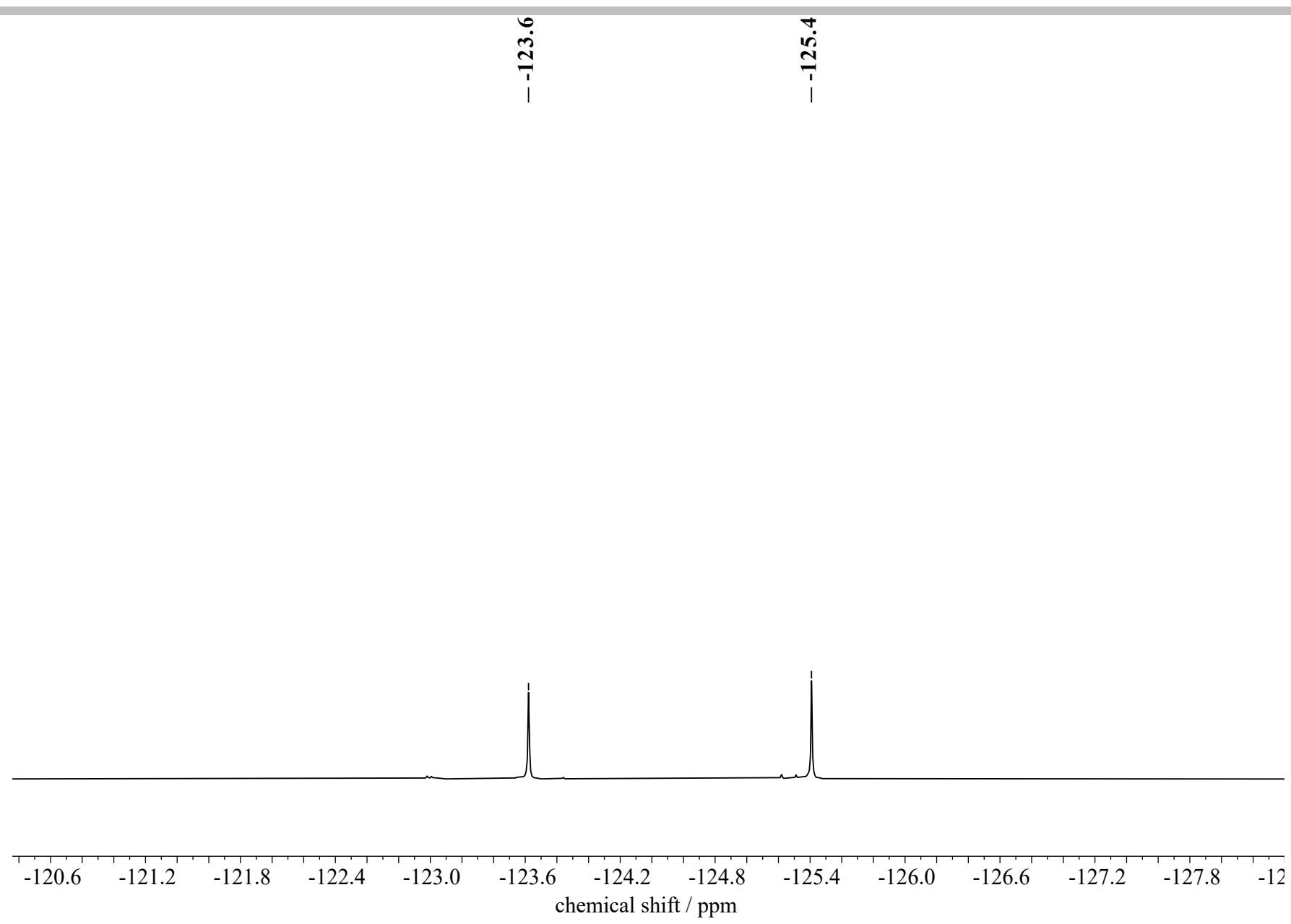


**Figure S59**. $^{19}F$ (282 MHz) NMR spectra of **36** in DMSO-$d_6$.

**Synthesis of 4-(3-fluoro-4-((4-fluoro-2-methylbenzo[d]oxazol-6-yl)diazenyl)phenyl)-1,2,3,6-tetrahydropyridin (5)**

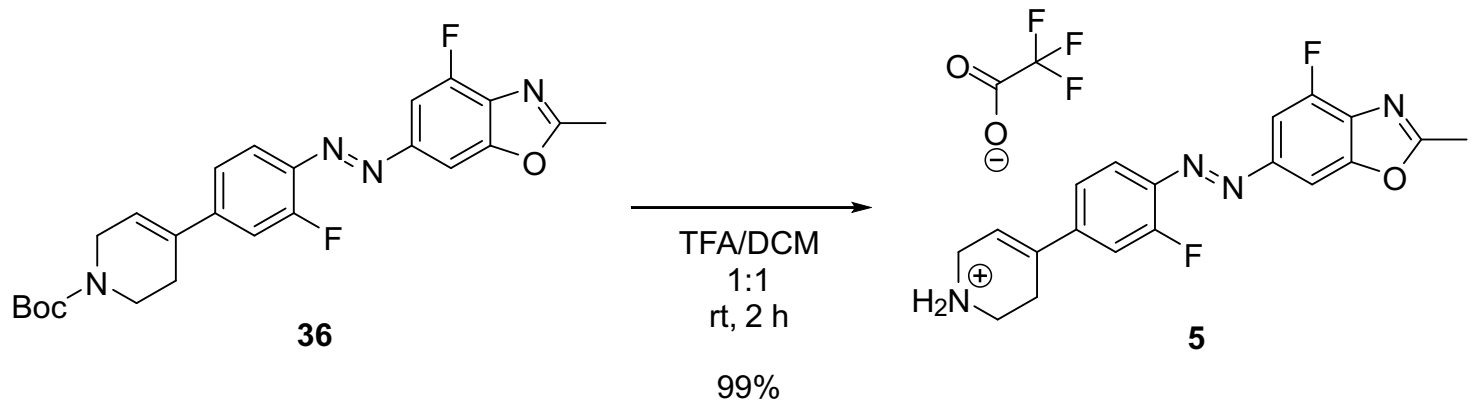


*Tert*-butyl-4-(3-fluoro-4-((4-fluoro-2-methylbenzo[*d*]oxazol-6-yl)diazenyl)phenyl)-3,6-dihydropyridine-1(*2H*)-carboxylate **36** (60 mg, 131 µmol, 1.00 eq) was dissolved in mixture of TFA/DCM (10 mL, 1:1, v%). The reaction was stirred at room temperature for 2 h. The reaction was then diluted with 100 mL distilled water and extracted with 100 mL EtOAc. The organic layer was washed with 100 mL distilled water and brine, dried over $MgSO_4$ and solvent was removed under reduced pressure. The crude product was purified by flash column chromatography (EtOAc/MeOH, 1:1, v%) to yield the desired compound 4-(3-fluoro-4-((4-fluoro-2-methylbenzo[*d*]oxazol-6-yl)diazenyl)phenyl)-1,2,3,6-tetrahydropyridin (**5**, 61 mg, 131 µmol, 99%) as an orange solid in form of a TFA salt.

**TLC:** $R_f$ = 0.19 (EtOAc/MeOH, 1:1, v%).

***trans*-isomer** (*cis*-isomer signals were labeled with * on NMR spectrum).

**$^1$H-NMR (300 MHz, DMSO-$d_6$, δ):** 9.05 (br, 2H, N$H_2$), 8.13 (d, $^4J$ = 1.5 Hz, 1H, $H_{arom.}$), 7.80-7.65 (m, 3H, $H_{arom.}$, $H_{arom.}$, $H_{arom.}$), 7.53 (dd, $^3J$ = 8.6 Hz, $^4J$ = 1.9 Hz, 1H, $H_{arom.}$), 6.53-6.50 (m, 1H, C*H*=C), 3.85-3.84 (m, 2H, C$H_2$), 3.39-3.37 (m, 2H, C$H_2$), 2.74-2.71 (m, 5H, C$H_2$, C$H_3$), ppm.

**$^{13}$C-NMR (75 MHz, DMSO-$d_6$, δ):** 167.4 (N=*C*-O), 161.3 ($C_{arom.}$), 157.9 ($C_{arom.}$), 152.8 (d, $^1J_{FC}$ = 231.5 Hz, *C*F), 152.7 (d, $^1J_{FC}$ = 226.9 Hz, *C*F), 149.7 (d, $^3J_{FC}$ = 5.9 Hz, $C_{arom.}$), 144.2 (d, $^3J_{FC}$ = 8.1 Hz, $C_{arom.}$), 138.5 (d, $^3J_{FC}$ = 7.1 Hz, $C_{arom.}$), 132.6 (d, $^3J_{FC}$ = 2.1 Hz, $C_{arom.}$), 132.0 (d, $^2J_{FC}$ = 17.4 Hz, $C_{arom.}$), 121.4 (d, $^3J_{FC}$ = 3.1 Hz, $C_{arom.}$H), 120.5 (*C*H=C), 117.8 ($C_{arom.}$H), 113.4 (d, $^2J_{FC}$ = 21.0 Hz, $C_{arom.}$H), 104.5 (d, $^2J_{FC}$ = 19.5 Hz, $C_{arom.}$H), 103.5 (d, $^3J_{FC}$ = 4.0 Hz, $C_{arom.}$H), 42.9 ($CH_2$), 41.7 ($CH_2$), 22.8 ($CH_2$), 14.4 ($CH_3$) ppm.

**$^{19}$F-NMR (282 MHz, DMSO-$d_6$, δ):** -123.4 (C*F*), -125.4 (C*F*) ppm.

**HRMS-ESI (m/z):** $[M+H]^+$ calcd for $C_{19}H_{17}F_2N_4O$, 355.1365; found, 355.1376.

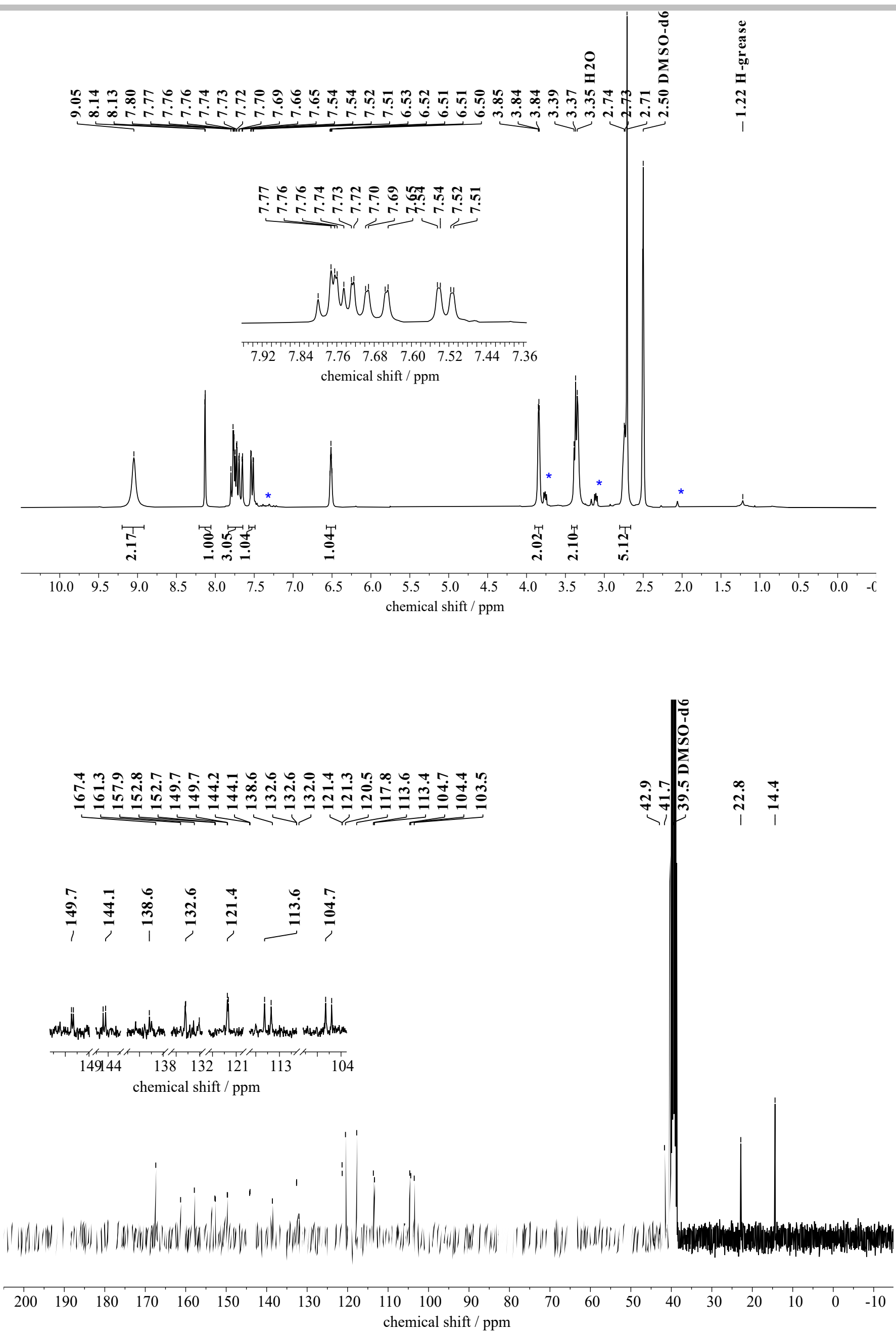


**Figure S60**. $^{1}H$ (300 MHz) and $^{13}C$ (75 MHz) NMR spectra of **5** in DMSO-$d_6$.

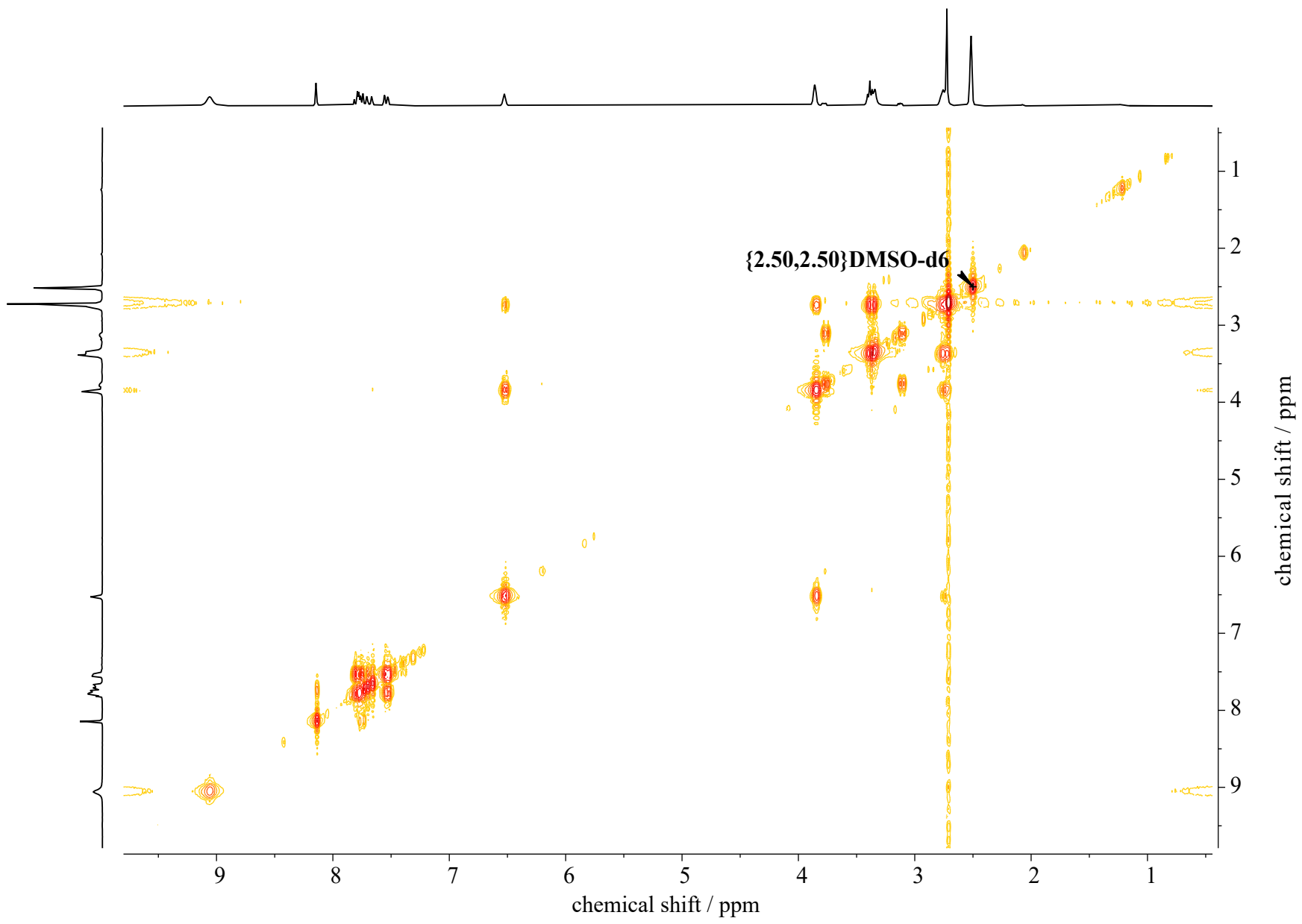


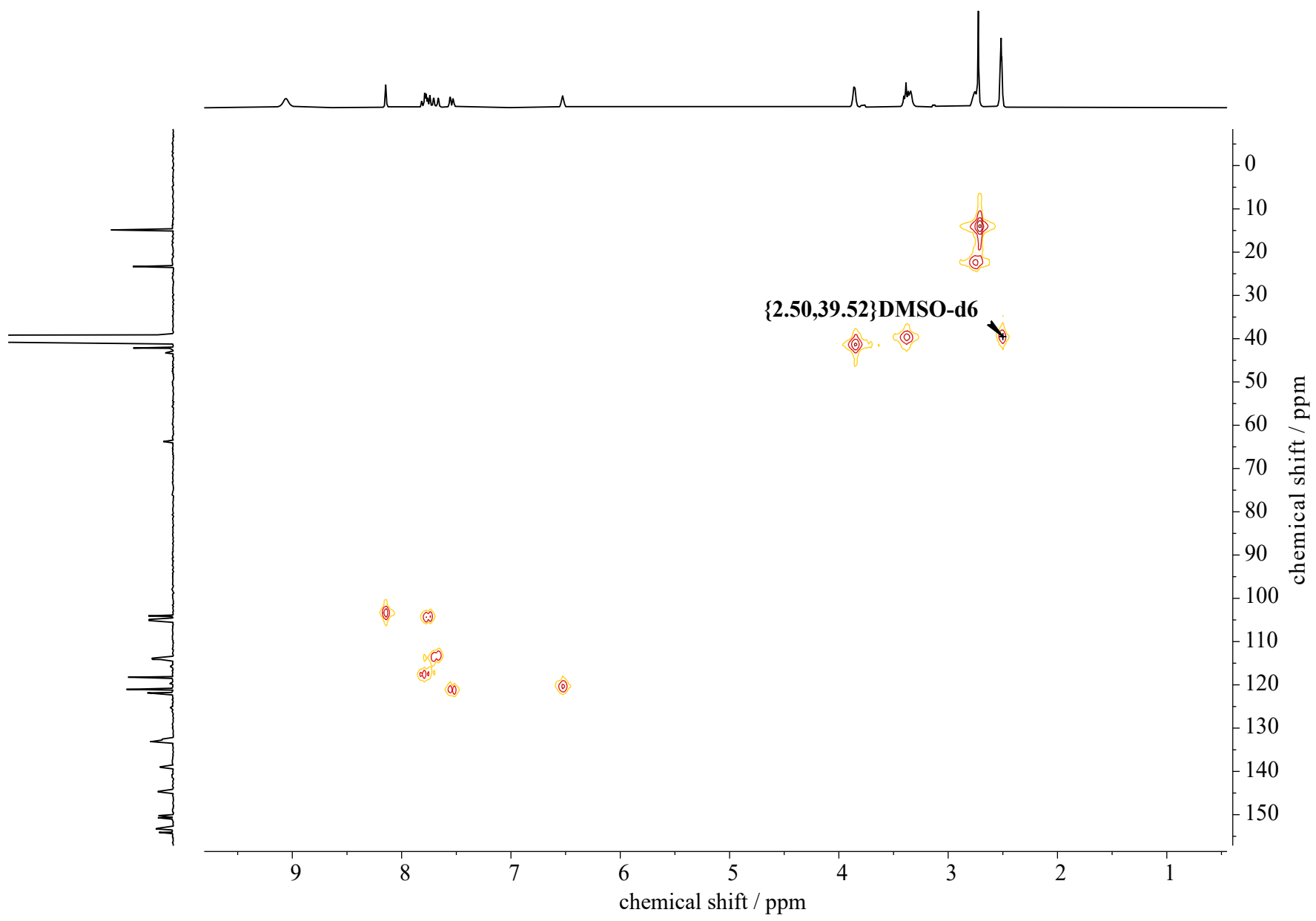


**Figure S61**. COSY and HMQC NMR spectra of **5** in DMSO-$d_6$.

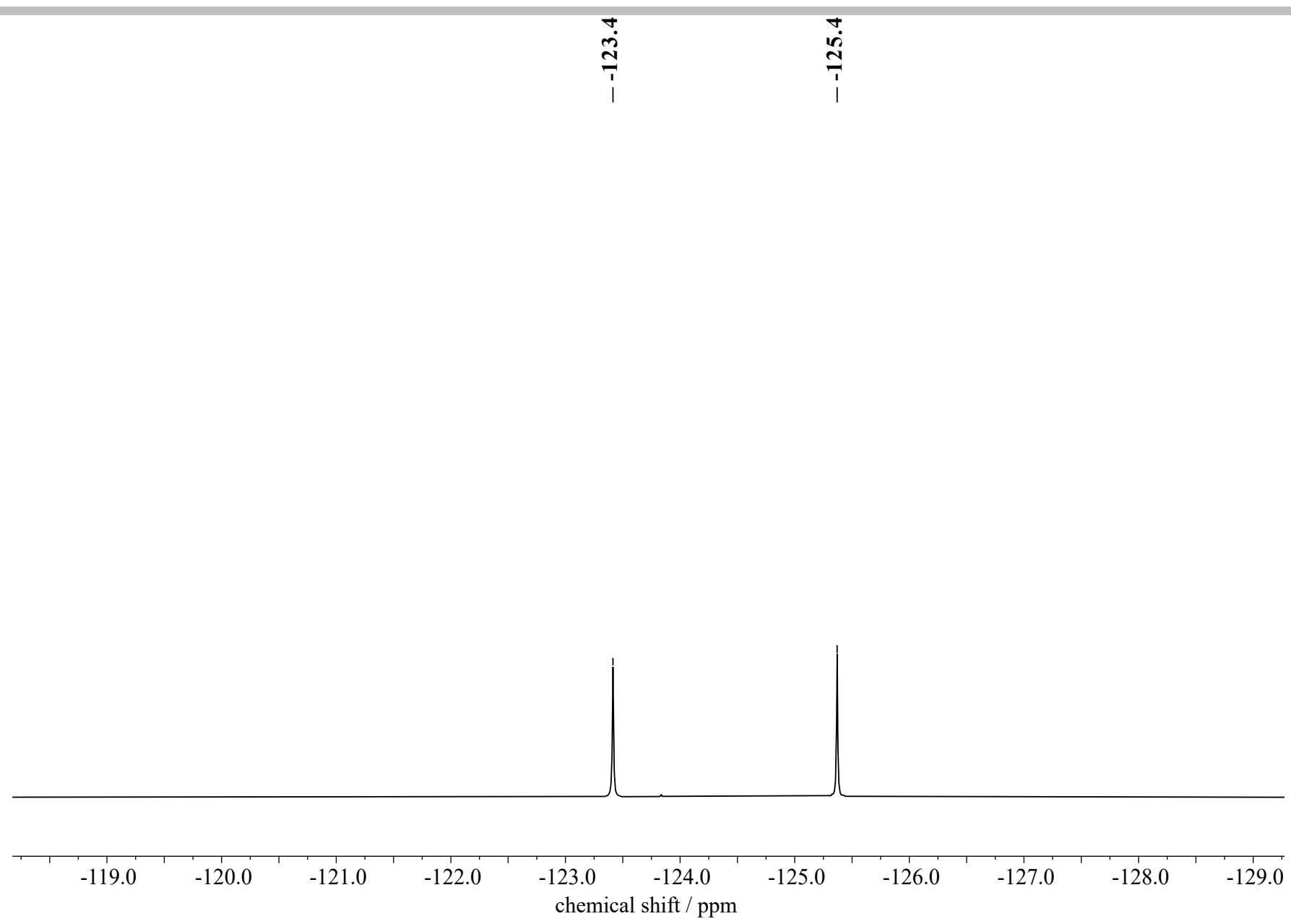


**Figure S62**. $^{19}F$ (282 MHz) NMR spectra of **5** in DMSO-$d_6$.

**Synthesis of 4-fluoro-6-((2-fluoro-4-(1-methyl-1,2,3,6-tetrahydropyridin-4-yl)phenyl)diazenyl)-2-methylbenzo[d]oxazole (8)**

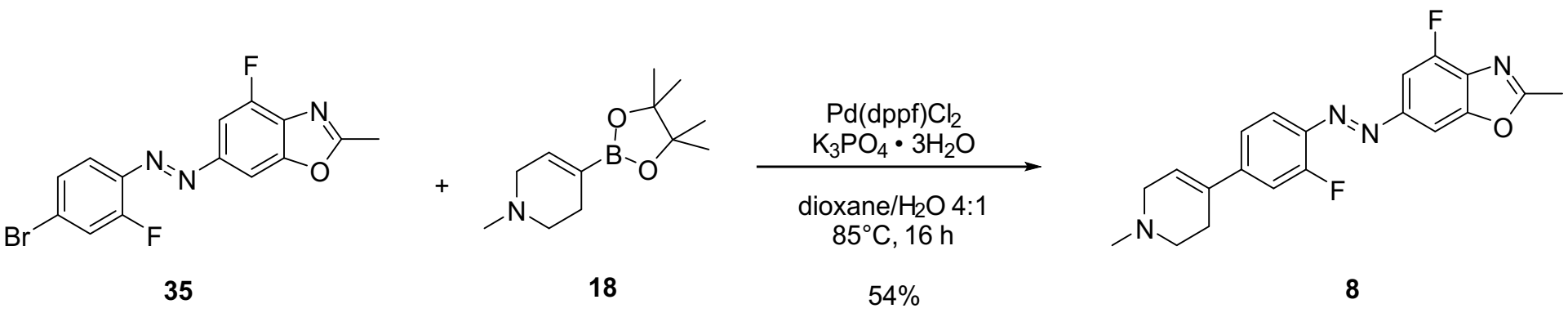


6-((4-Bromo-2-fluorophenyl)diazenyl)-4-fluoro-2-methylbenzo[d]oxazole **35** (100 mg, 284 µmol, 1.00 eq), 1-methyl-1,2,3,6-tetrahydropyridine-4-boronic acid pinacol ester **18** (64 mg, 284 µmol, 1.00 eq), Pd(dppf)$Cl_2$ (10 mg, 14.2 µmol, 0.05 eq) and $K_3PO_4{\cdot}3H_2O$ (196 mg, 851 µmol, 3.00 eq) were dissolved in mixture of dioxane/distilled water (20 mL, 4:1, v%). The reaction was stirred under $N_2$ atmosphere at 85 °C for 16 h. The reaction was then diluted with 100 mL distilled water and extracted with 100 mL EtOAc. The organic layer was washed with 100 mL distilled water and brine, dried over $MgSO_4$ and solvent was removed under reduced pressure. The crude product was purified by flash column chromatography (EtOAc/MeOH, 1:1, v%) to yield the desired compound 4-fluoro-6-((2-fluoro-4-(1-methyl-1,2,3,6-tetrahydropyridin-4-yl)phenyl)diazenyl)-2-methylbenzo[*d*]oxazole (**8**, 57 mg, 155 µmol, 54%) as an orange solid.

**TLC:** $R_f$ = 0.20 (EtOAc/MeOH, 1:1, v%).

***trans*-isomer** (*cis*-isomer signals were labeled with * on NMR spectrum).

**$^1$H-NMR (300 MHz, DMSO-*d*$_6$, *δ*):** 8.12 (d, $^4J$ = 1.6 Hz, 1H, $H_{arom.}$), 7.76-7.71 (m, 2H, $H_{arom.}$, $H_{arom.}$), 7.57 (dd, $^3J$ = 12.8 Hz, $^4J$ = 1.9 Hz, 1H, $H_{arom.}$), 7.47 (dd, $^3J$ = 8.6 Hz, $^4J$ = 1.9 Hz, 1H, $H_{arom.}$), 6.50-6.48 (m, 1H, C*H*=C), 3.09-3.07 (m, 2H, C$H_2$), 2.71 (s, 3H, C$H_3$), 2.60-2.52 (m, 4H, C$H_2$, C$H_2$), 2.29 (s, 3H, C$H_3$), ppm.

**$^{13}$C-NMR (75 MHz, DMSO-*d*$_6$, *δ*):** 167.3 (N=*C*-O), 164.4 ($C_{arom.}$), 161.7 ($C_{arom.}$), 153.6 ($C_{arom.}$), 152.8 (d, $^1J_{FC}$ = 226.6 Hz, *C*F), 149.8 (d, $^1J_{FC}$ = 224.6 Hz, *C*F), 145.9($C_{arom.}$), 138.1 ($C_{arom.}$), 132.2 ($C_{arom.}$), 126.1 (*C*H=C), 121.0 ($C_{arom.}$H), 117.4 ($C_{arom.}$H), 113.1 (d, $^2J_{FC}$ = 19.4 Hz, $C_{arom.}$H), 104.5 (d, $^2J_{FC}$ = 19.2 Hz, $C_{arom.}$H), 103.4 ($C_{arom.}$H), 54.6 ($CH_2$), 51.5 ($CH_2$), 45.3 ($CH_3$), 27.0 ($CH_2$), 14.4 ($CH_3$) ppm.

**$^{19}$F-NMR (282 MHz, DMSO-*d*$_6$, *δ*):** -123.7 (C*F*), -125.4 (C*F*) ppm.

**HRMS-ESI (m/z):** $[M+H]^+$ calcd for $C_{20}H_{19}F_2N_4O$, 369.1521; found, 369.1534.

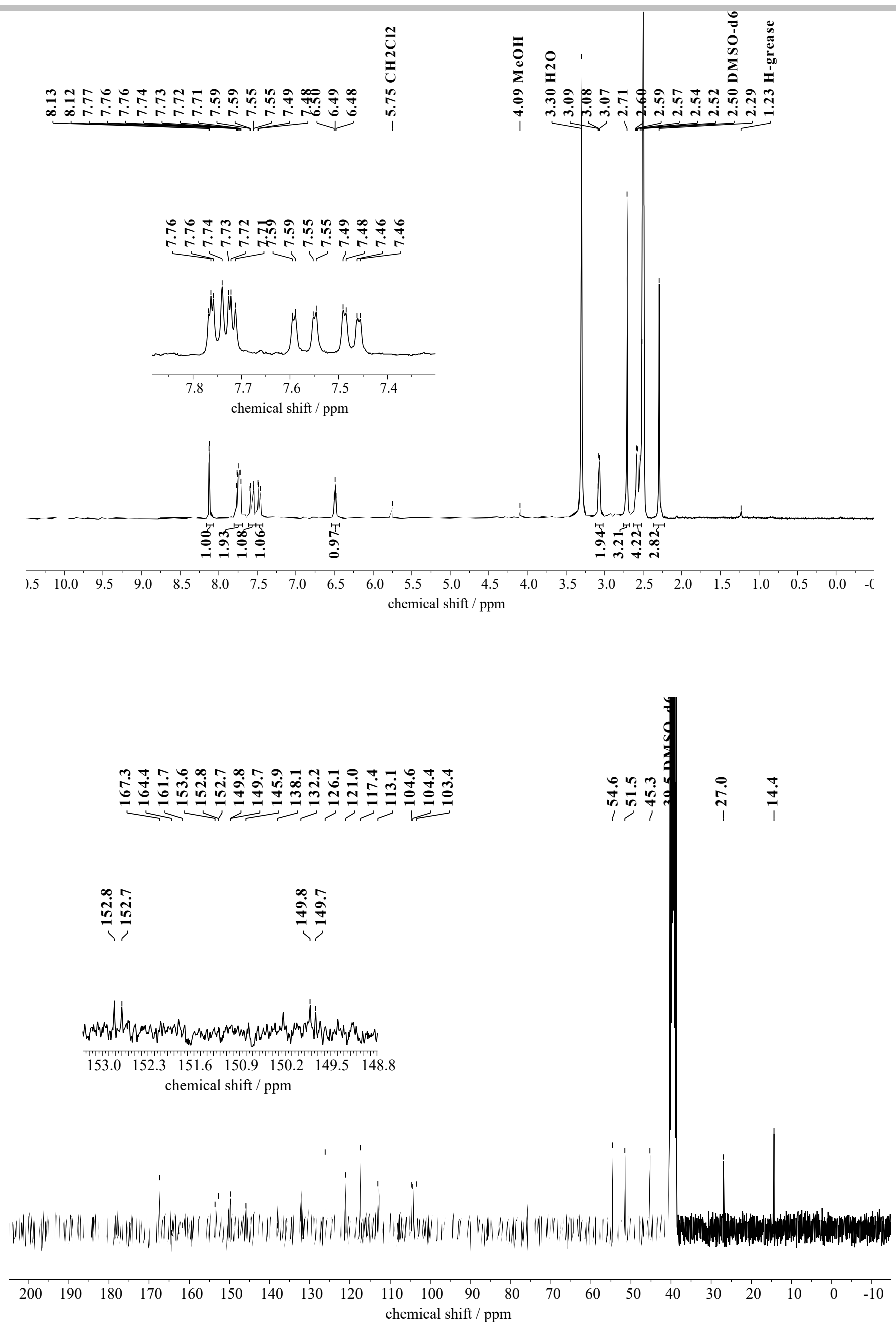


**Figure S63**. $^{1}H$ (300 MHz) and $^{13}C$ (75 MHz) NMR spectra of **8** in DMSO-$d_6$.

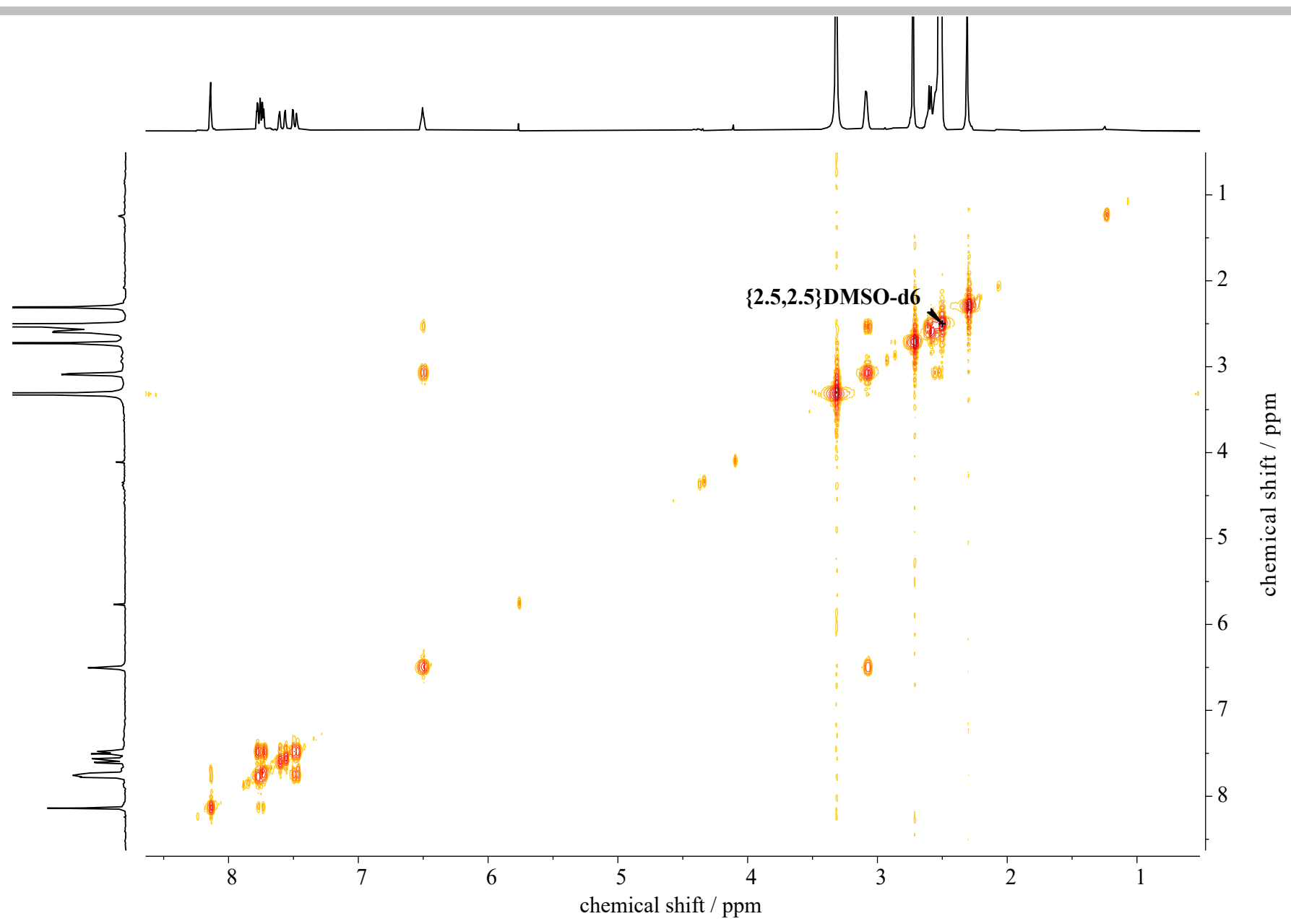


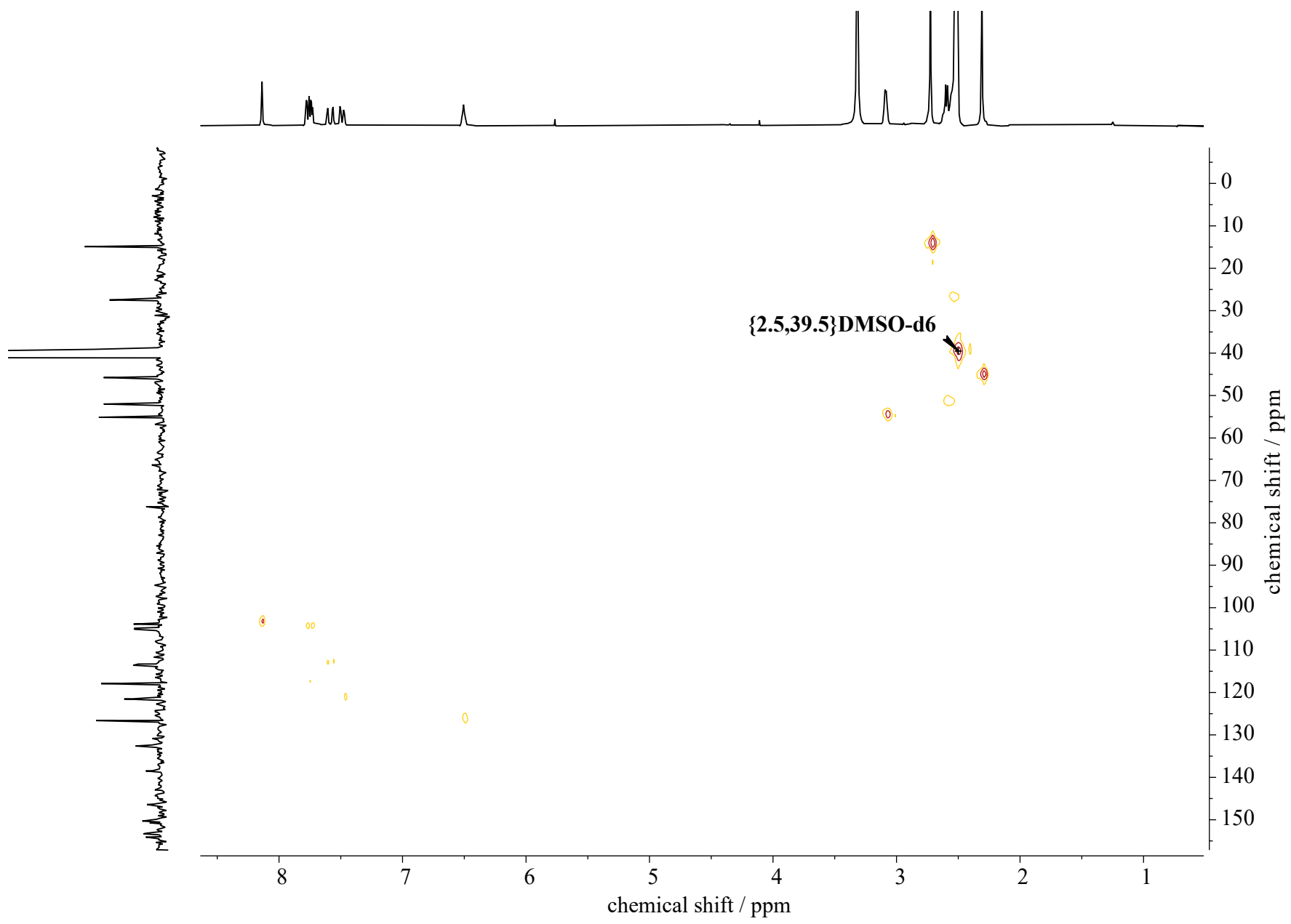


**Figure S64**. COSY and HMQC NMR spectra of **8** in DMSO-$d_6$.

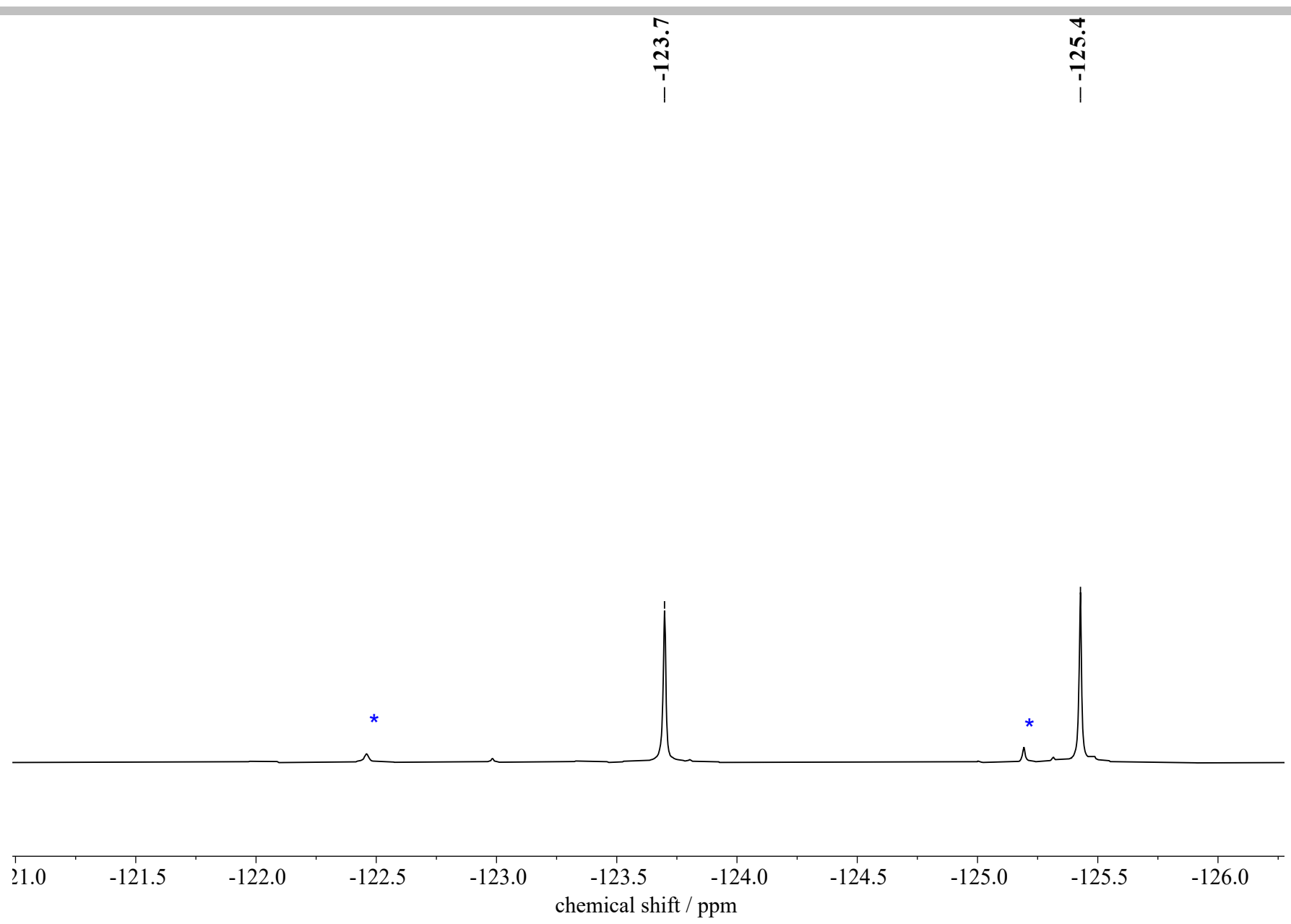


**Figure S65**. $^{19}F$ (282 MHz) NMR spectra of **8** in DMSO-$d_6$.

**Synthesis of 6-((4-bromo-2,6-difluorophenyl)diazenyl)-4-fluoro-2-methylbenzo[d]oxazole (37)**

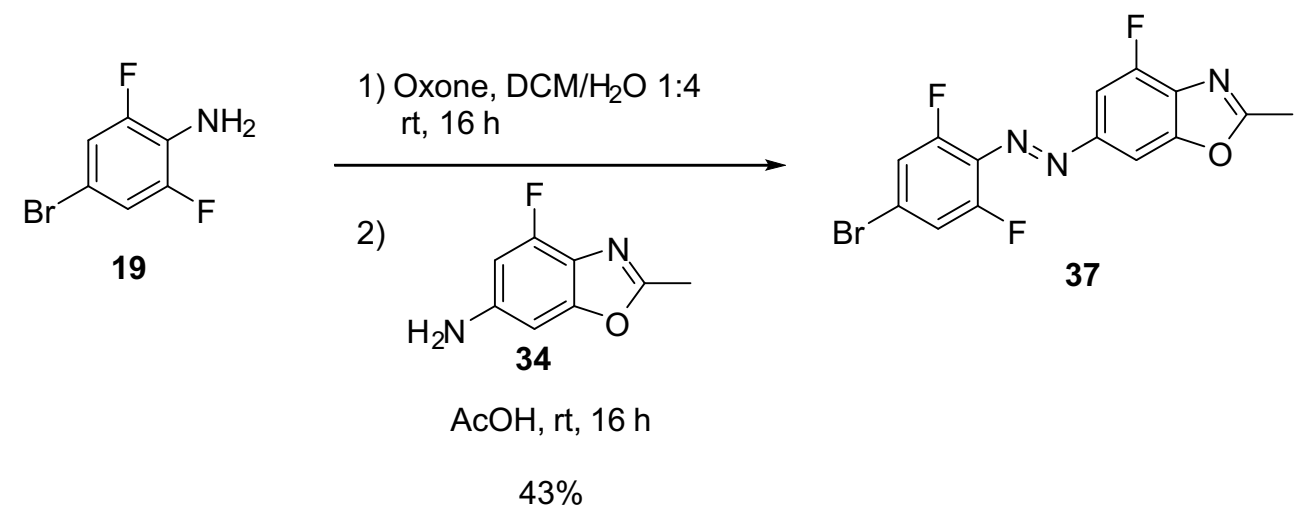


4-Bromo-2,6-difluoroaniline **19** (626 mg, 3.01 mmol, 2.00 eq) was dissolved in 5 mL DCM, Oxone® (1.85 g, 6.02 mmol, 4.00 eq) was dissolved in 20 mL distilled water and added to the solution. The resulting mixture was stirred for 16 h at room temperature. The reaction mixture was then diluted with 100 mL DCM and washed with 100 mL 1 M HCl, distilled water and brine. The organic layer was dried over $MgSO_4$ and the solvent was removed under reduced pressure. The resulting brown solid and 4-fluoro-2-methyl-1,3-benzoxazol-6-amine **34** (250 mg, 1.51 mmol, 1.00 eq) were suspended in 20 mL acetic acid. The reaction mixture was stirred for 16 h at room temperature. The reaction was then diluted with 200 mL distilled water and extracted with 200 mL EtOAc. The organic layer was washed with 200 mL distilled water and brine, dried over $MgSO_4$ and solvent was removed under reduced pressure. The crude product was purified by flash column chromatography (DCM) to yield the desired compound 6-((4-bromo-2,6-difluorophenyl)diazenyl)-4-fluoro-2-methylbenzo[*d*]oxazole (**37**, 238 mg, 643 µmol, 43%) as an orange solid.

**TLC:** $R_f$ = 0.50 (DCM).

***trans*-isomer** (*cis*-isomer signals were labeled with * on NMR spectrum).

**$^1$H-NMR (300 MHz, DMSO-*d*$_6$, *δ*):** 8.14 (d, $^4J$ = 1.6 Hz, 1H, $H_{arom.}$), 7.80-7.73 (m, 2H, $H_{arom.}$, $H_{arom.}$), 7.71 (dd, $^3J$ = 10.7 Hz, $^4J$ = 1.6 Hz, 1H, $H_{arom.}$), 2.71 (s, 3H, C*H*$_3$) ppm.

**$^{13}$C-NMR (75 MHz, DMSO-*d*$_6$, *δ*):** 167.7 (N=*C*-O), 156.7 (d, $^1J_{FC}$ = 294.7 Hz, 2C, 2x*C*F), 152.7 (d, $^1J_{FC}$ = 298.5 Hz, *C*F), 153.6 ($C_{arom.}$), 149.9 (d, $^3J_{FC}$ = 6.3 Hz, $C_{arom.}$), 132.8 (d, $^2J_{FC}$ = 17.5 Hz, $C_{arom.}$), 123.7 ($C_{arom.}$), 117.2 (d, $^3J_{FC}$ = 3.5 Hz, 2C, $C_{arom.}$H), 116.8 (d, $^3J_{FC}$ = 3.7 Hz, $C_{arom.}$H), 104.3 (d, $^2J_{FC}$ = 19.4 Hz, $C_{arom.}$H), 103.7 (d, $^3J_{FC}$ = 4.3 Hz, $C_{arom.}$H), 14.4 (*C*H$_3$) ppm.

**$^{19}$F-NMR (282 MHz, DMSO-*d*$_6$, *δ*):** -119.3 (2F, 2xC*F*). -125.2 (C*F*) ppm.

**HRMS-ESI (m/z):** $[M+H]^+$ calcd for $C_{14}H_8BrF_3N_3O$, 371.9778; found, 371.9777.

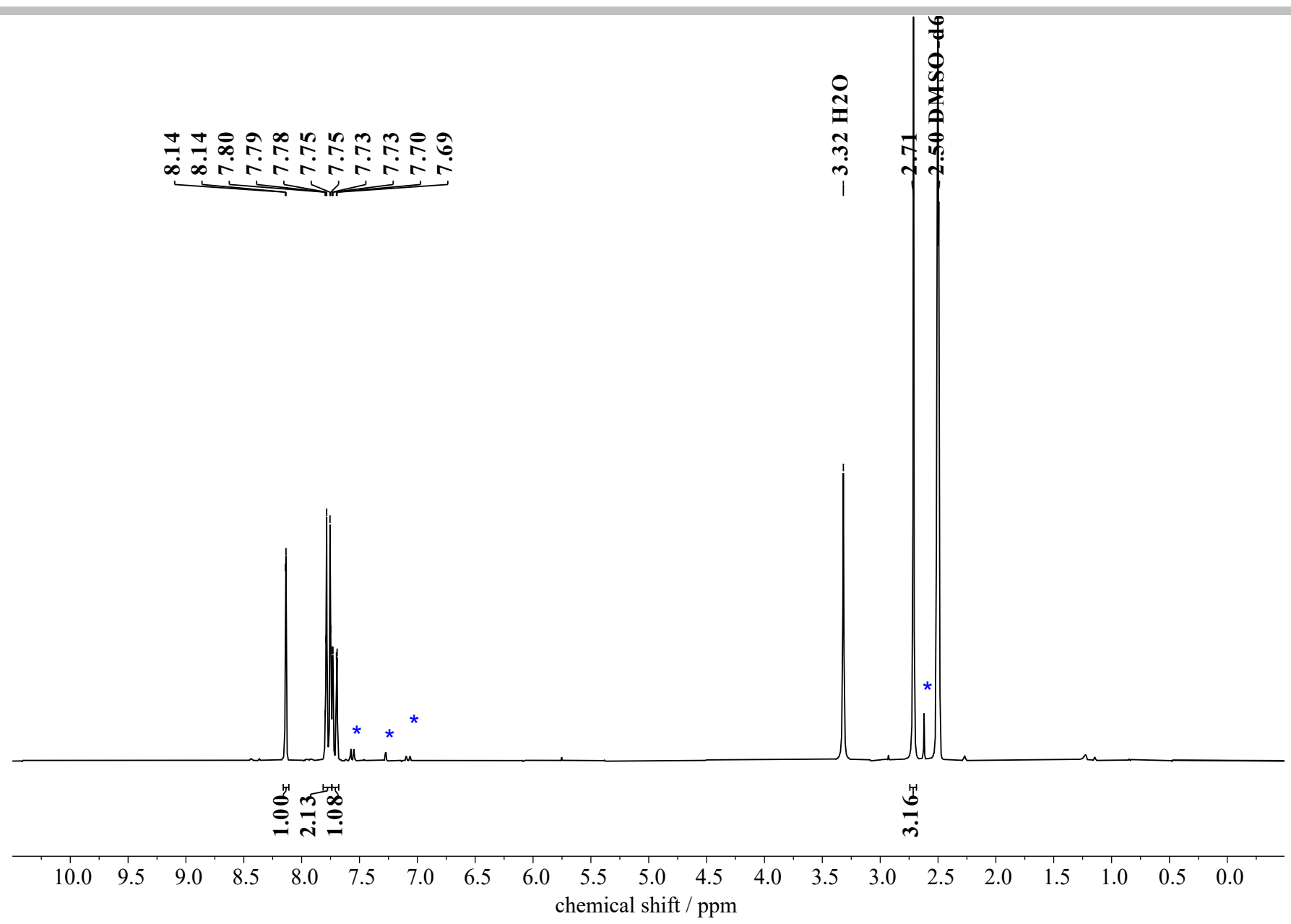


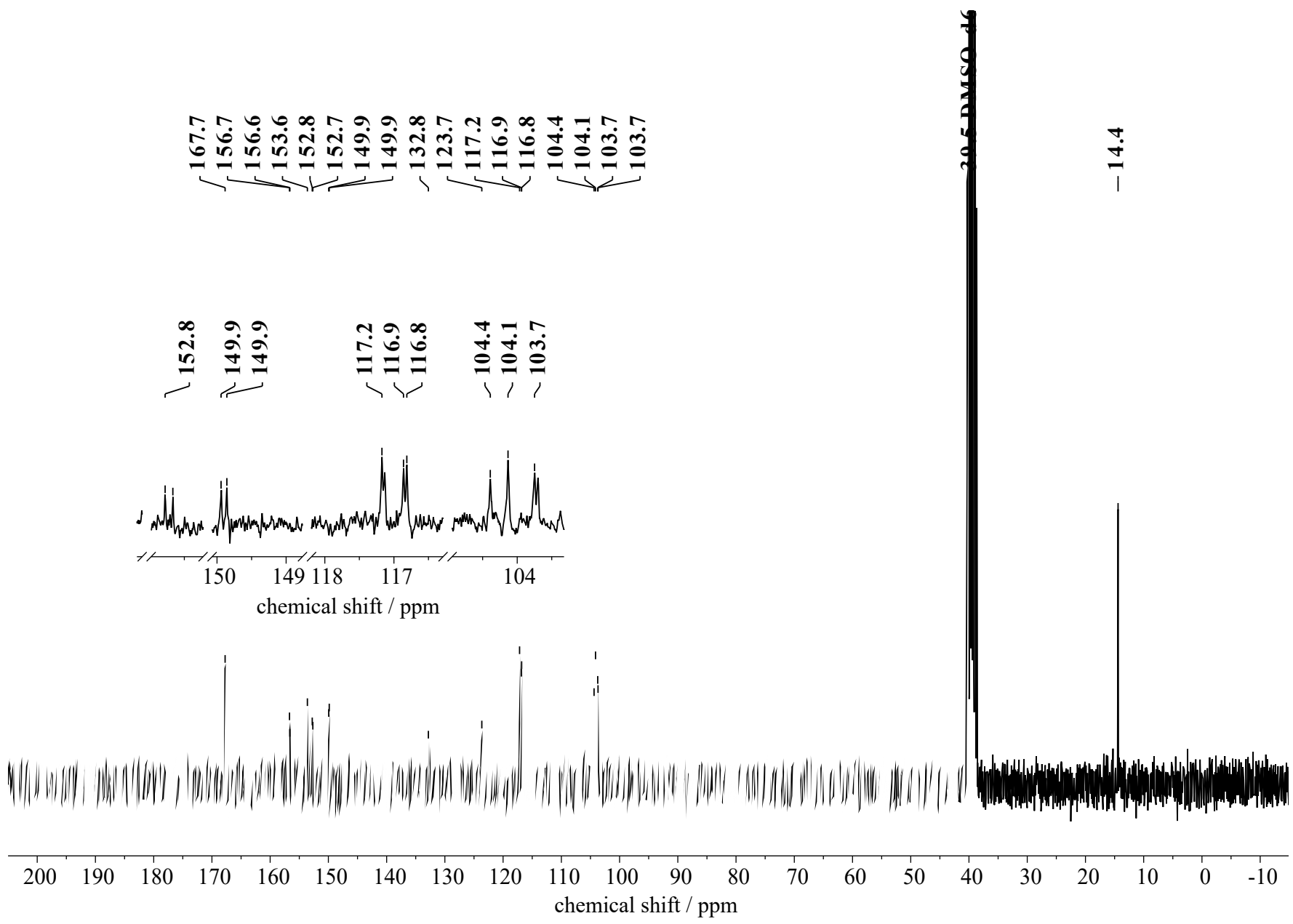


**Figure S66**. $^{1}H$ (300 MHz) and $^{13}C$ (75 MHz) NMR spectra of **37** in DMSO-$d_6$.

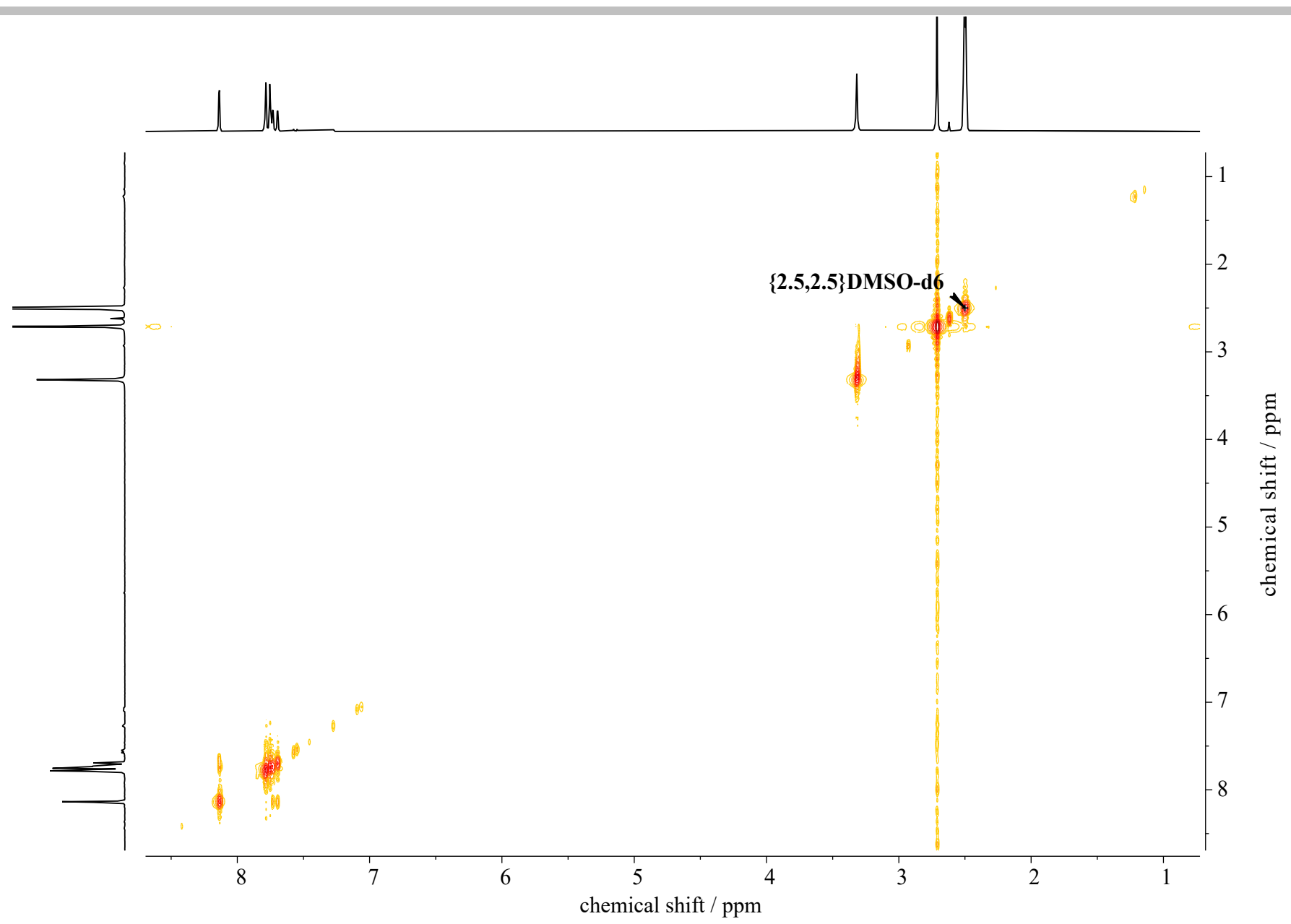


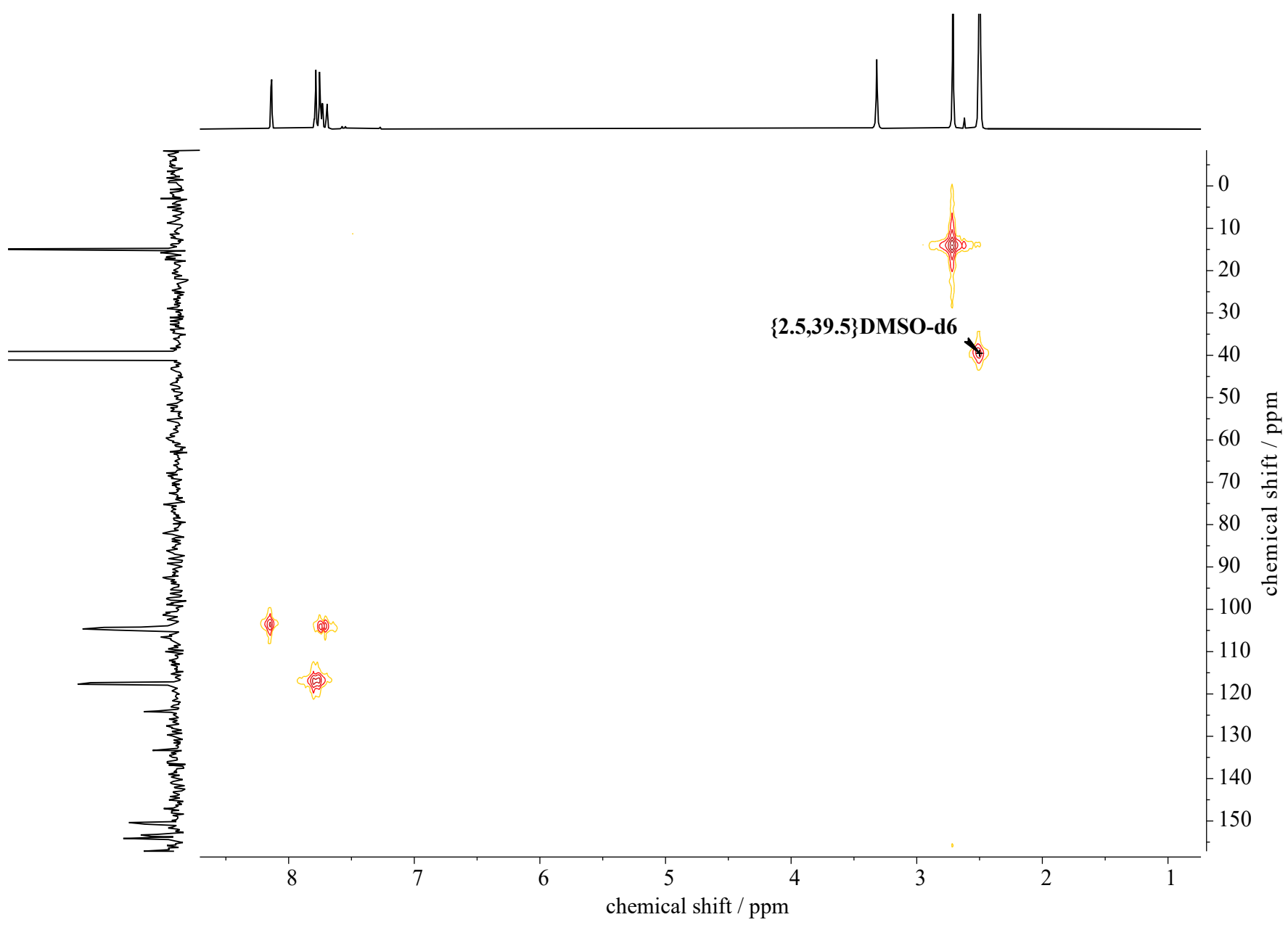


**Figure S67**. COSY and HMQC NMR spectra of **37** in DMSO-$d_6$.

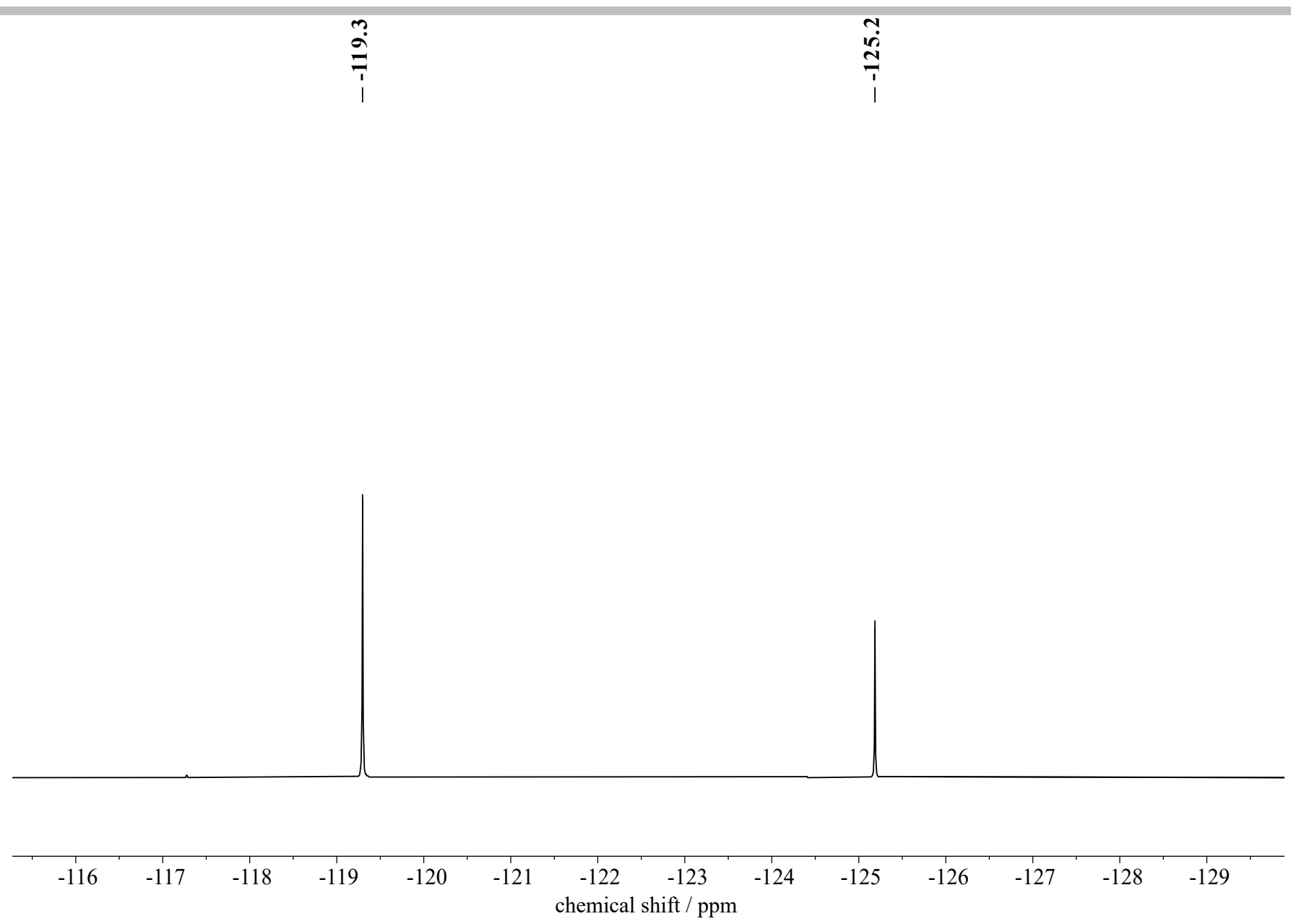


**Figure S68**. $^{19}F$ (282 MHz) NMR spectra of **37** in DMSO-$d_6$.

**Synthesis of 6-((2,6-difluoro-4-(1-methyl-1,2,3,6-tetrahydropyridin-4-yl)phenyl)diazenyl)-4-fluoro-2-methylbenzo[d]oxazole (9)**

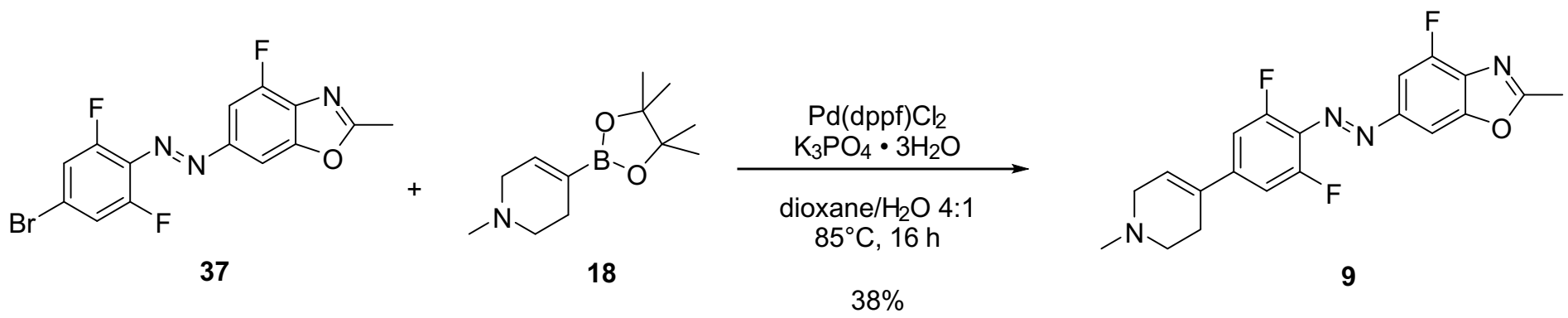


6-((4-Bromo-2,6-difluorophenyl)diazenyl)-4-fluoro-2-methylbenzo[*d*]oxazole **37** (80 mg, 216 µmol, 1.00 eq), 1-methyl-1,2,3,6-tetrahydropyridine-4-boronic acid pinacol ester **18** (48 mg, 216 µmol, 1.00 eq), Pd(dppf)$Cl_2$ (8 mg, 10.8 µmol, 0.05 eq) and $K_3PO_4 \cdot 3H_2O$ (156 mg, 648 µmol, 3.00 eq) were dissolved in mixture of dioxane/distilled water (20 mL, 4:1, v%). The reaction was stirred under $N_2$ atmosphere at 85 °C for 16 h. The reaction was then diluted with 100 mL distilled water and extracted with 100 mL EtOAc. The organic layer was washed with 100 mL distilled water and brine, dried over $MgSO_4$ and solvent was removed under reduced pressure. The crude product was purified by flash column chromatography (EtOAc/MeOH, 1:1, v%) to yield the desired compound 6-((2,6-difluoro-4-(1-methyl-1,2,3,6-tetrahydropyridin-4-yl)phenyl)diazenyl)-4-fluoro-2-methylbenzo[*d*]oxazole (**9**, 32 mg, 82.8 µmol, 38%) as an orange solid.

**TLC:** $R_f$ = 0.12 (EtOAc/MeOH, 1:1, v%).

***trans*-isomer** (*cis*-isomer signals were labeled with * on NMR spectrum).

**$^1$H-NMR (300 MHz, DMSO-*d*$_6$, *δ*):** 8.11 (d, $^4J$ = 1.5 Hz, 1H, $H_{arom.}$), 7.70 (dd, $^3J$ = 10.8 Hz, $^4J$ = 1.6 Hz, 1H, $H_{arom.}$), 7.44 (d, $^3J$ = 11.8 Hz, 2H, $H_{arom.}$, $H_{arom.}$), 6.57 (m, 1H, C*H*=C), 3.08-3.06 (m, 2H, C$H_2$), 2.71 (s, 3H, C$H_3$), 2.60-2.56 (m, 4H, C$H_2$, C$H_2$), 2.29 (s, 3H, C$H_3$), ppm.

**$^{13}$C-NMR (75 MHz, DMSO-*d*$_6$, *δ*):** 167.5 (N=*C*-O), 153.6 ($C_{arom.}$), 152.7 (2C, 2x*C*F), 150.3 (*C*F), 144.6 ($C_{arom.}$), 144.3 ($C_{arom.}$), 131.4 ($C_{q.}$), 127.2 (*C*H=C), 108.8 (d, $^2J_{FC}$ = 19.6 Hz, 2C, $C_{arom.}$H), 104.1 (d, $^2J_{FC}$ = 19.2 Hz, $C_{arom.}$H), 103.4 (d, $^3J_{FC}$ = 4.2 Hz, $C_{arom.}$H), 54.6 ($CH_2$), 51.4 ($CH_2$), 45.2 ($CH_3$), 26.7 ($CH_2$), 14.4 ($CH_3$) ppm.

**$^{19}$F-NMR (282 MHz, DMSO-*d*$_6$, *δ*):** -120.4 (2F, 2xC*F*). -125.3 (C*F*) ppm.

**HRMS-ESI (m/z):** $[M+H]^+$ calcd for $C_{20}H_{18}F_3N_4O$, 387.1433; found, 387.1433.

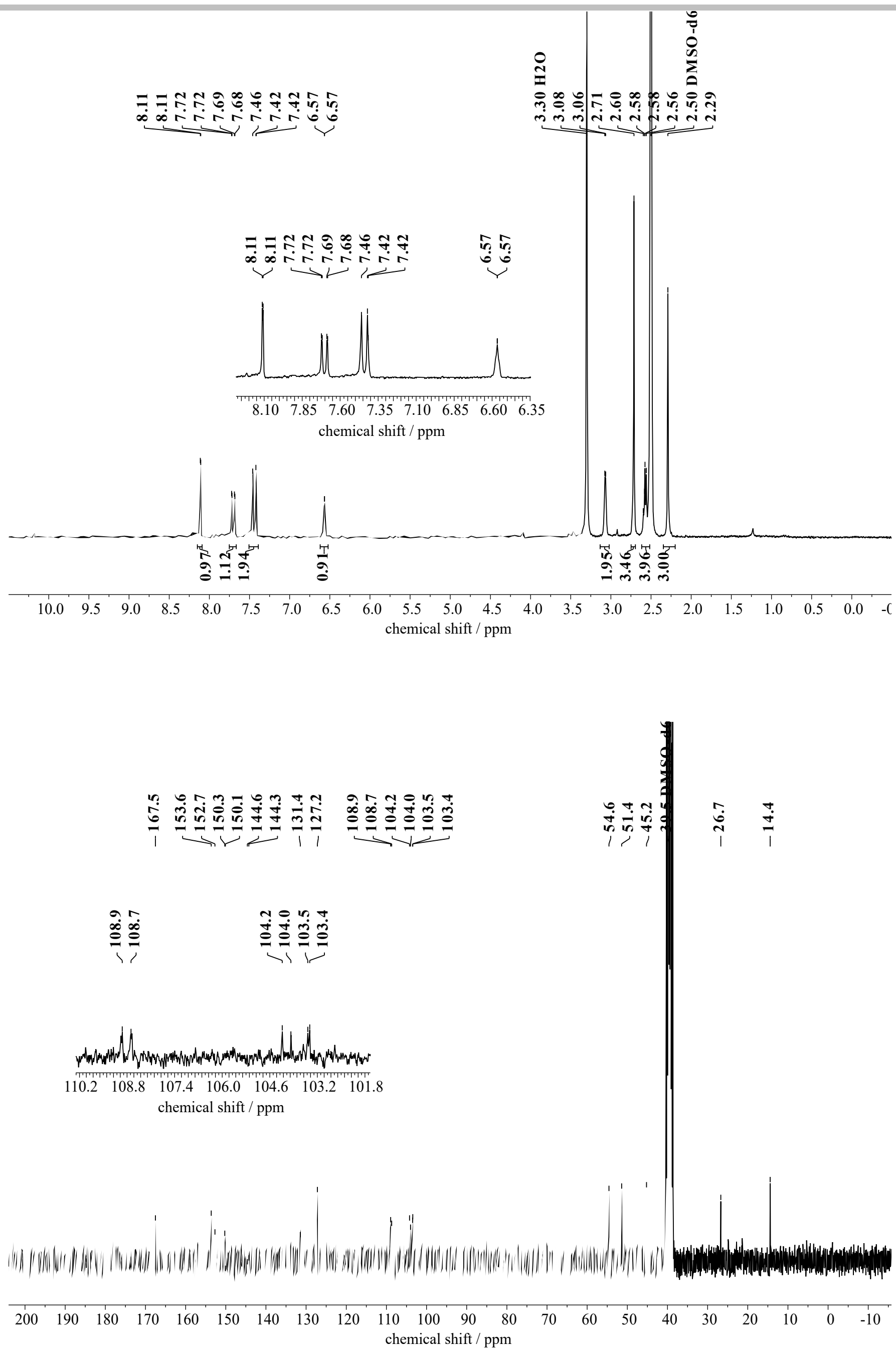


**Figure S69**. $^{1}H$ (300 MHz) and $^{13}C$ (75 MHz) NMR spectra of **9** in DMSO-$d_6$.

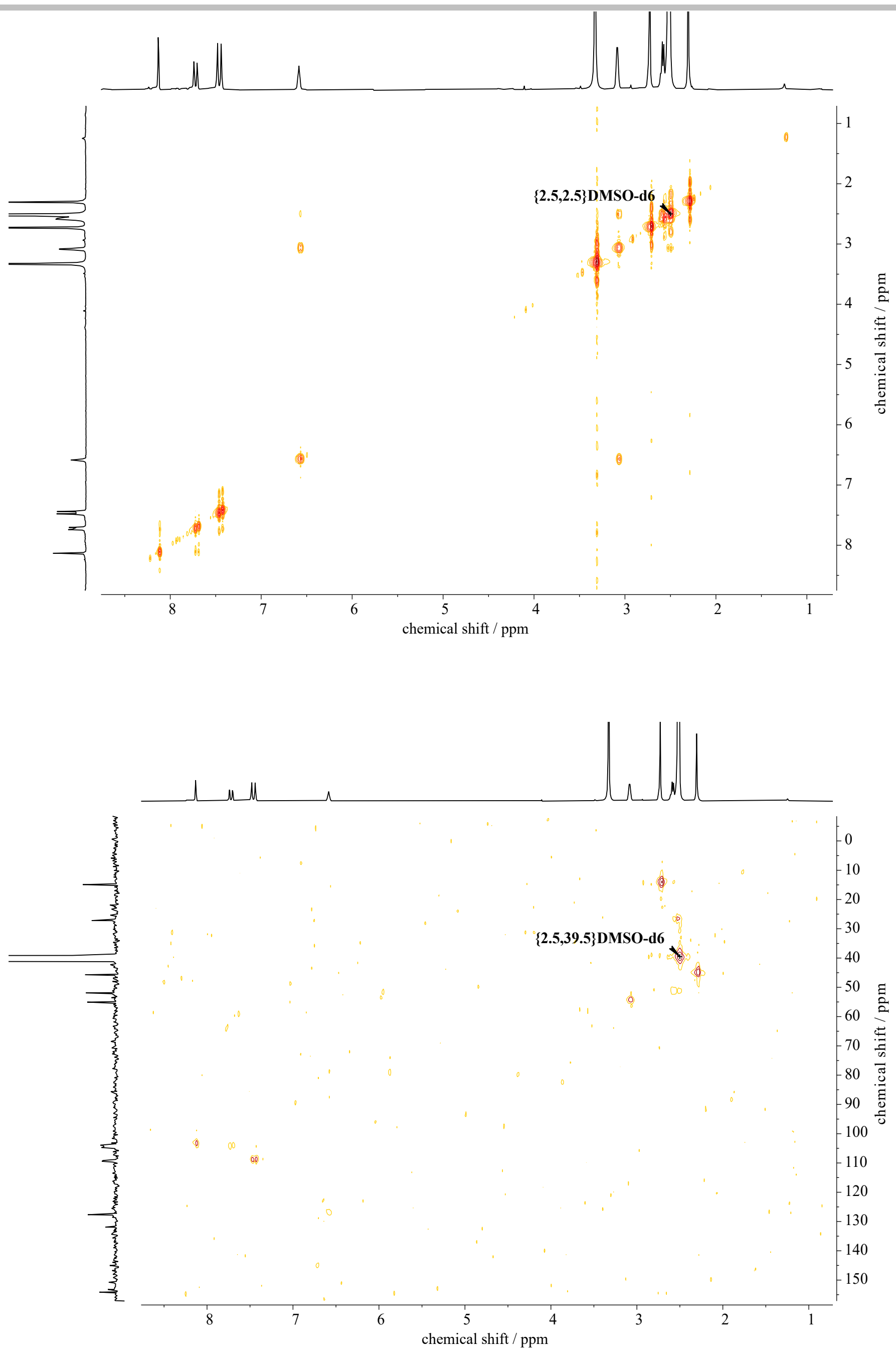


**Figure S70**. COSY and HMQC NMR spectra of **9** in DMSO-$d_6$.

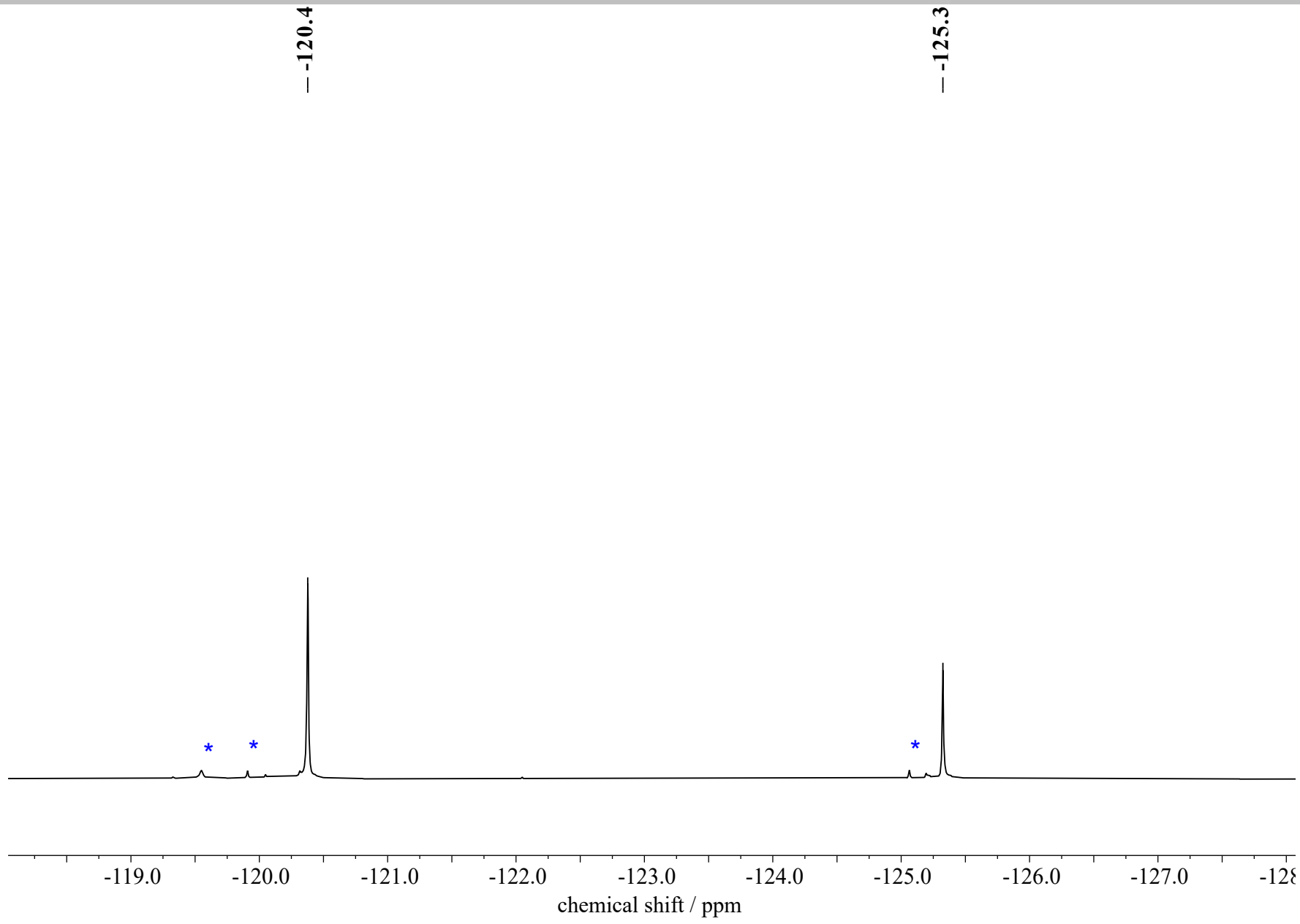


**Figure S71**. $^{19}F$ (282 MHz) NMR spectra of **9** in DMSO-$d_6$.

**Synthesis of 2-fluoro-4-(1-methyl-1,2,3,6-tetrahydropyridin-4-yl)aniline (38)**

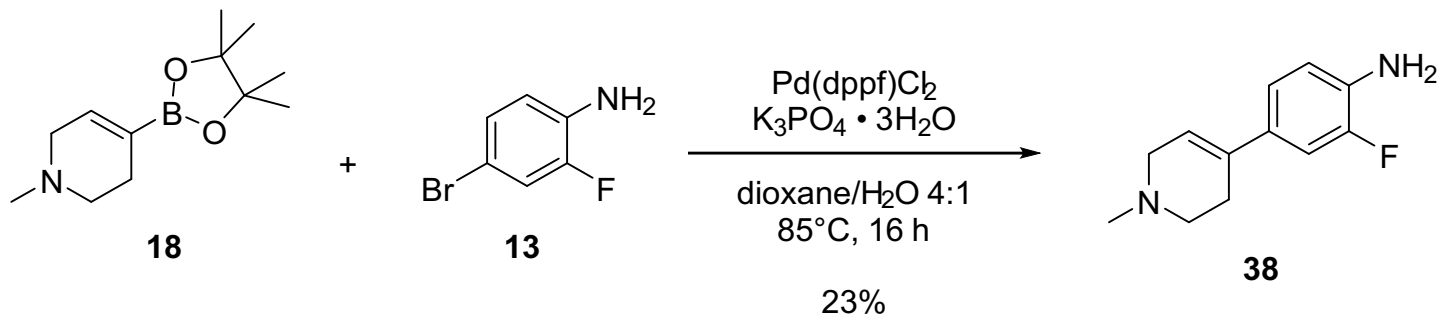


4-Bromo-2-fluoroaniline **13** (170 mg, 896 µmol, 1.00 eq), 1-methyl-1,2,3,6-tetrahydropyridine-4-boronic acid pinacol ester **18** (200 mg, 896 µmol, 1.00 eq), Pd(dppf)$Cl_2$ (33 mg, 44.8 µmol, 0.05 eq) and $K_3PO_4 \cdot 3H_2O$ (716 mg, 2.69 mmol, 3.00 eq) were dissolved in mixture of dioxane/distilled water (20 mL, 4:1, v%). The reaction was stirred under $N_2$ atmosphere at 85 °C for 16 h. The reaction was then diluted with 100 mL distilled water and extracted with 100 mL EtOAc. The organic layer was washed with 100 mL distilled water and brine, dried over $MgSO_4$ and solvent was removed under reduced pressure. The crude product was purified by flash column chromatography (DCM/MeOH, 10:1, v%) to yield the desired compound 2-fluoro-4-(1-methyl-1,2,3,6-tetrahydropyridin-4-yl)aniline (**38**, 43 mg, 206 µmol, 23%) as an orange solid.

**TLC:** $R_f$ = 0.13 (DCM/MeOH, 10:1, v%).

**$^1$H-NMR (300 MHz, DMSO-*d*$_6$, *δ*):** 7.06 (dd, $^3J$ = 13.5 Hz, $^4J$ = 2.1 Hz, 1H, $H_{arom.}$), 6.97 (dd, $^3J$ = 8.3 Hz, $^4J$ = 2.1 Hz, 1H, $H_{arom.}$), 6.70 (dd, $^3J$ = 9.7 Hz, $^3J$ = 8.3 Hz, 1H, $H_{arom.}$), 5.97-5.95 (m, 1H, C*H*=C), 5.13 (s, 2H, N$H_2$), 3.02-2.99 (m, 2H, C$H_2$), 2.57 (t, 2H, C$H_2$), 2.42-2.37 (m, 2H, C$H_2$), 2.28 (s, 3H, C$H_3$) ppm.

**$^{13}$C-NMR (75 MHz, DMSO-*d*$_6$, *δ*):** 150.5 (d, $^1J_{FC}$ = 236.0 Hz, *C*F), 135.4 (d, $^2J_{FC}$ = 13.2 Hz, $C_{arom.}$), 132.5 (d, $^3J_{FC}$ = 2.1 Hz, $C_{arom.}$), 128.4 (d, $^3J_{FC}$ = 6.0 Hz, $C_{arom.}$H), 120.5 (d, $^3J_{FC}$ = 2.7 Hz, $C_{arom.}$H), 118.3 (*C*H=C), 115.8 (d, $^3J_{FC}$ = 5.0 Hz, $C_{arom.}$H), 110.9 (d, $^2J_{FC}$ = 18.9 Hz, $C_{arom.}$H), 54.3 ($CH_2$), 51.7 ($CH_2$), 45.2 ($CH_3$), 27.0 ($CH_2$) ppm.

**$^{19}$F-NMR (282 MHz, DMSO-*d*$_6$, *δ*)**: -135.2 (C*F*) ppm.

**HRMS-ESI (m/z):** $[M+H]^+$ calcd for $C_{12}H_{16}FN_2$, 207.1292; found, 207.1292.

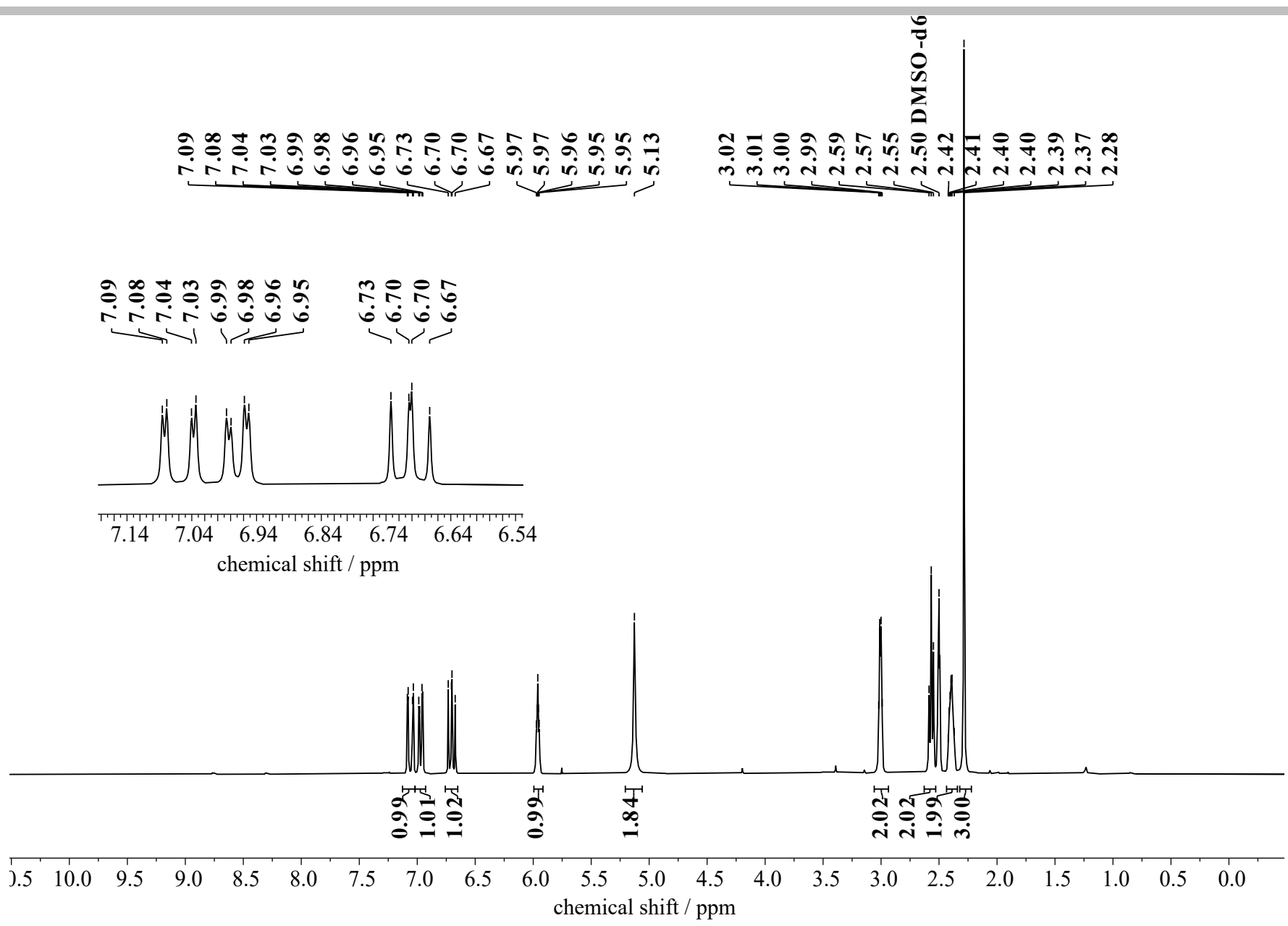


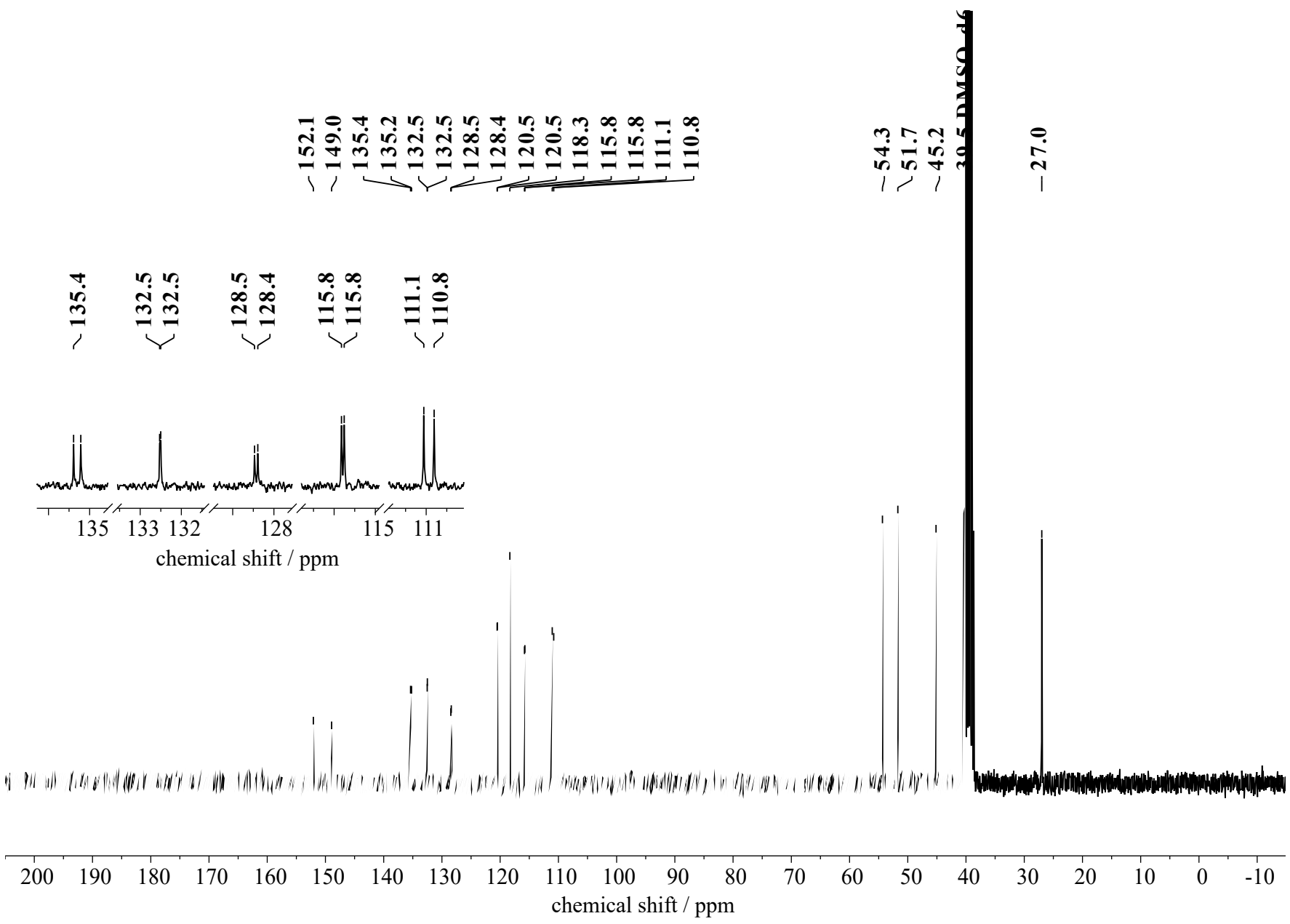


**Figure S72**. $^{1}H$ (300 MHz) and $^{13}C$ (75 MHz) NMR spectra of **38** in DMSO-$d_6$.

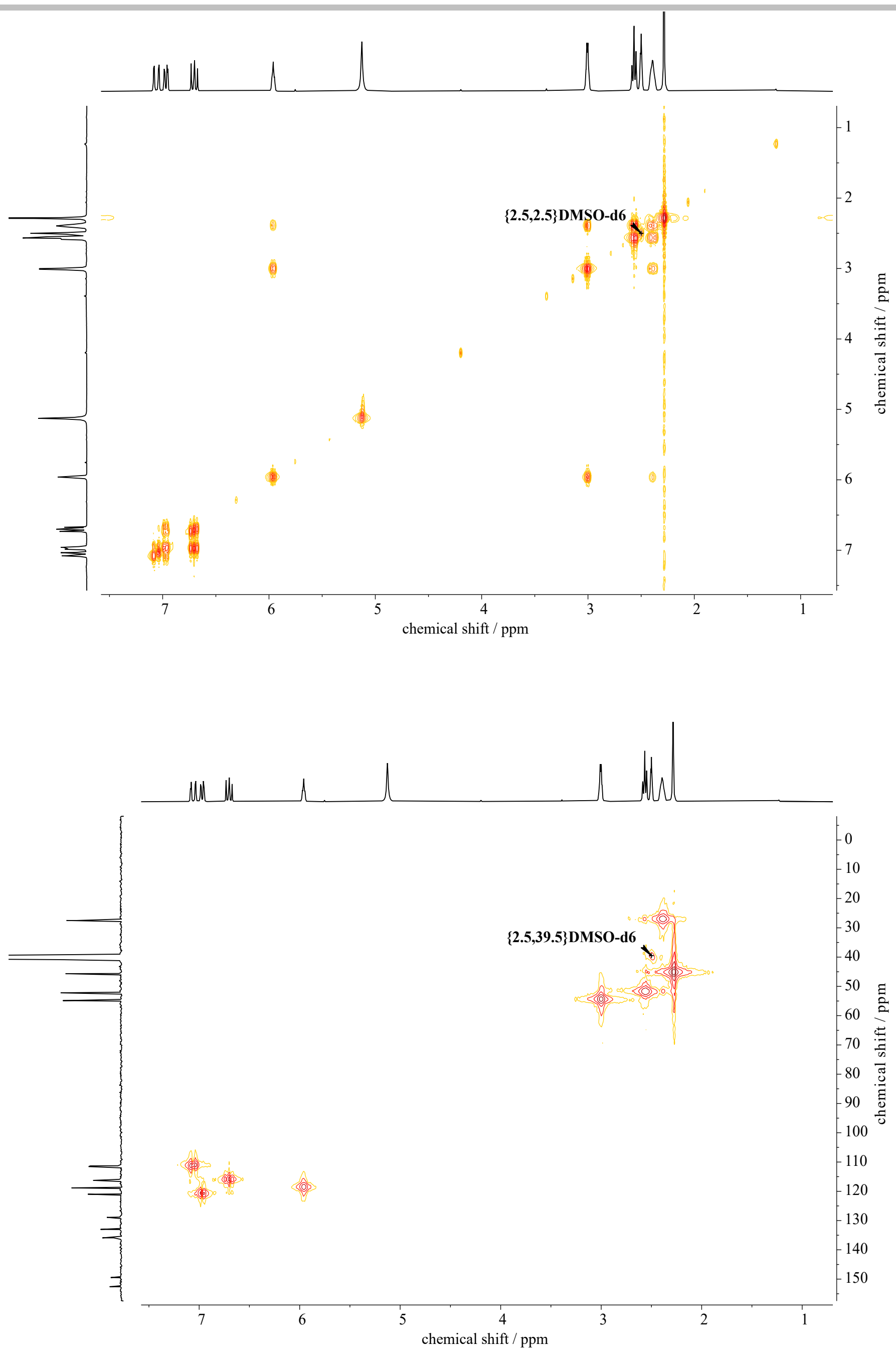


**Figure S73**. COSY and HMQC NMR spectra of **38** in DMSO-$d_6$.

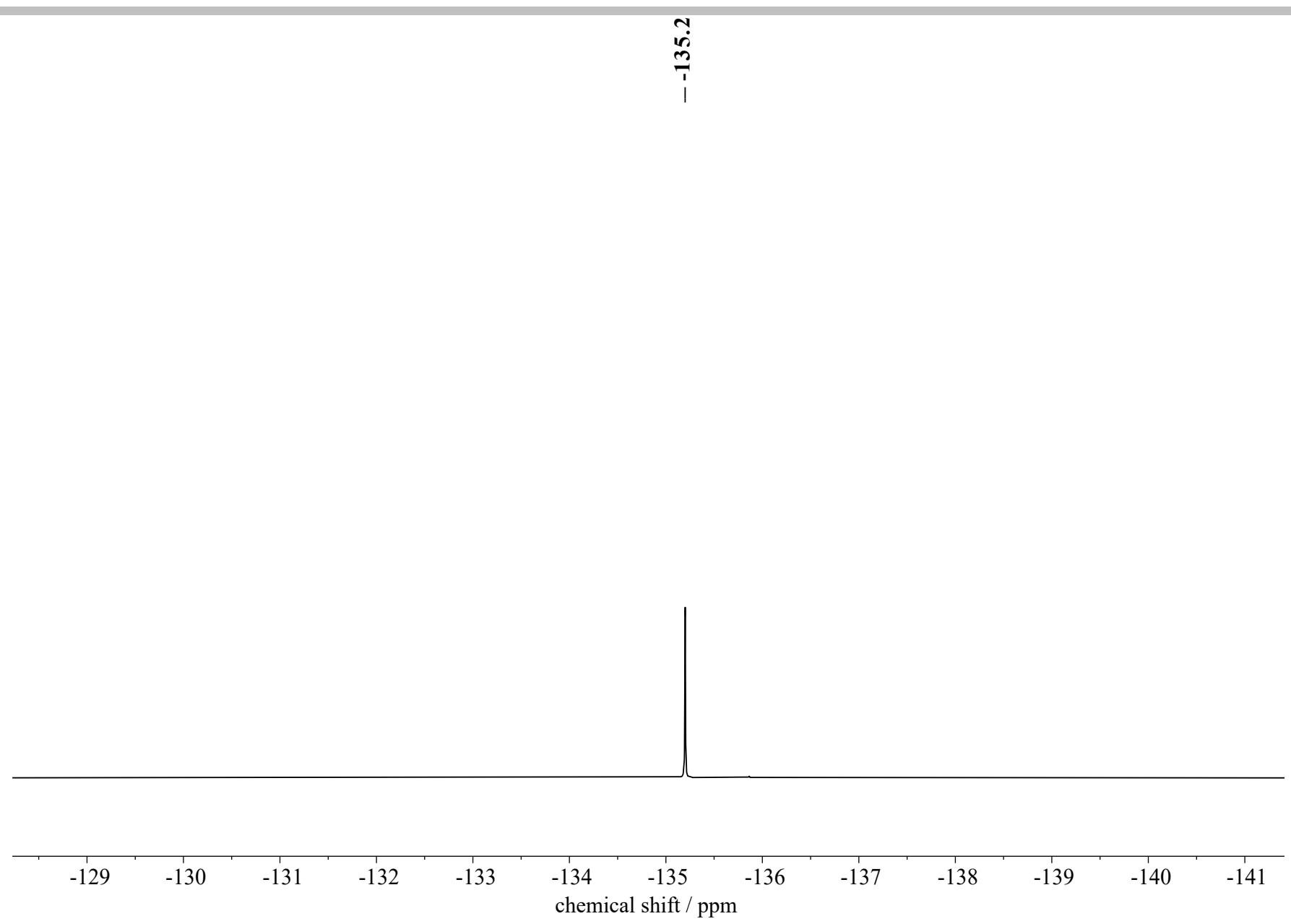


**Figure S74**. $^{19}$F (282 MHz) NMR spectra of **38** in DMSO-$d_6$.

**Synthesis of 2-fluoro-4-(1-methylpiperidin-4-yl)aniline (39)**

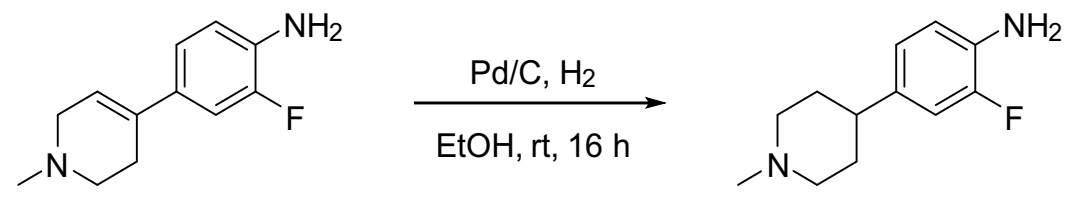


2-Fluoro-4-(1-methyl-1,2,3,6-tetrahydropyridin-4-yl)aniline **38** (62 mg, 300 µmol, 1.00 eq) was dissolved in 20 mL absolute ethanol at room temperature. Palladium on carbon (15 mg, 140 µmol, 0.47 eq) was added to the solution. The reaction was flushed with hydrogen gas using a balloon filled with hydrogen and stirred under hydrogen gas for 16 h. The reaction mixture was filtered over celite and concentrated under reduced pressure affording product 2-fluoro-4-(1-methylpiperidin-4-yl)aniline as beige oil (**39**, 60.0 mg, 288 µmol, 96%).

**TLC:** $R_f$ = 0.23 (DCM/MeOH, 4:1, v%).

**$^1$H-NMR (300 MHz, DMSO-*d*$_6$, *δ*):** 6.83 (dd, $^3J$ = 12.9 Hz, $^4J$ = 1.9 Hz, 1H, $H_{arom.}$), 6.75-6.64 (m, 2H, $H_{arom.}$, $H_{arom.}$), 4.86 (s, 2H, N$H_2$), 2.83-2.79 (m, 2H, C$H_2$), 2.50-2.27 (m, 1H, C*H*), 2.16 (s, 3H, C$H_3$), 1.90 (td, $^3J$ = 11.6 Hz, $^4J$ = 2.6 Hz, 2H, C$H_2$), 1.68-1.51 (m, 4H, C$H_2$, C$H_2$) ppm.

**$^{13}$C-NMR (75 MHz, DMSO-*d*$_6$, *δ*):** 150.6 (d, $^1J_{FC}$ = 236.3 Hz, *C*F), 134.5 (d, $^3J_{FC}$ = 5.3 Hz, $C_{arom.}$), 134.1 (d, $^2J_{FC}$ = 12.9 Hz, $C_{arom.}$H), 122.4 (d, $^3J_{FC}$ = 4.9 Hz, $C_{arom.}$H), 116.2 (d, $^3J_{FC}$ = 4.9 Hz, $C_{arom.}$H), 113.0 (d, $^2J_{FC}$ = 18.1 Hz, $C_{arom.}$H), 55.7 (2C, $CH_2$, $CH_2$), 46.0 ($CH_3$), 39.9 (*C*H), 33.0 (2C, $CH_2$, $CH_2$) ppm.

**$^{19}$F-NMR (282 MHz, DMSO-*d*$_6$, *δ*)**: -135.0 (C*F*) ppm.

**HRMS-ESI (m/z):** $[M+H]^+$ calcd for $C_{12}H_{18}FN_2$, 209.1449; found, 209.1451.

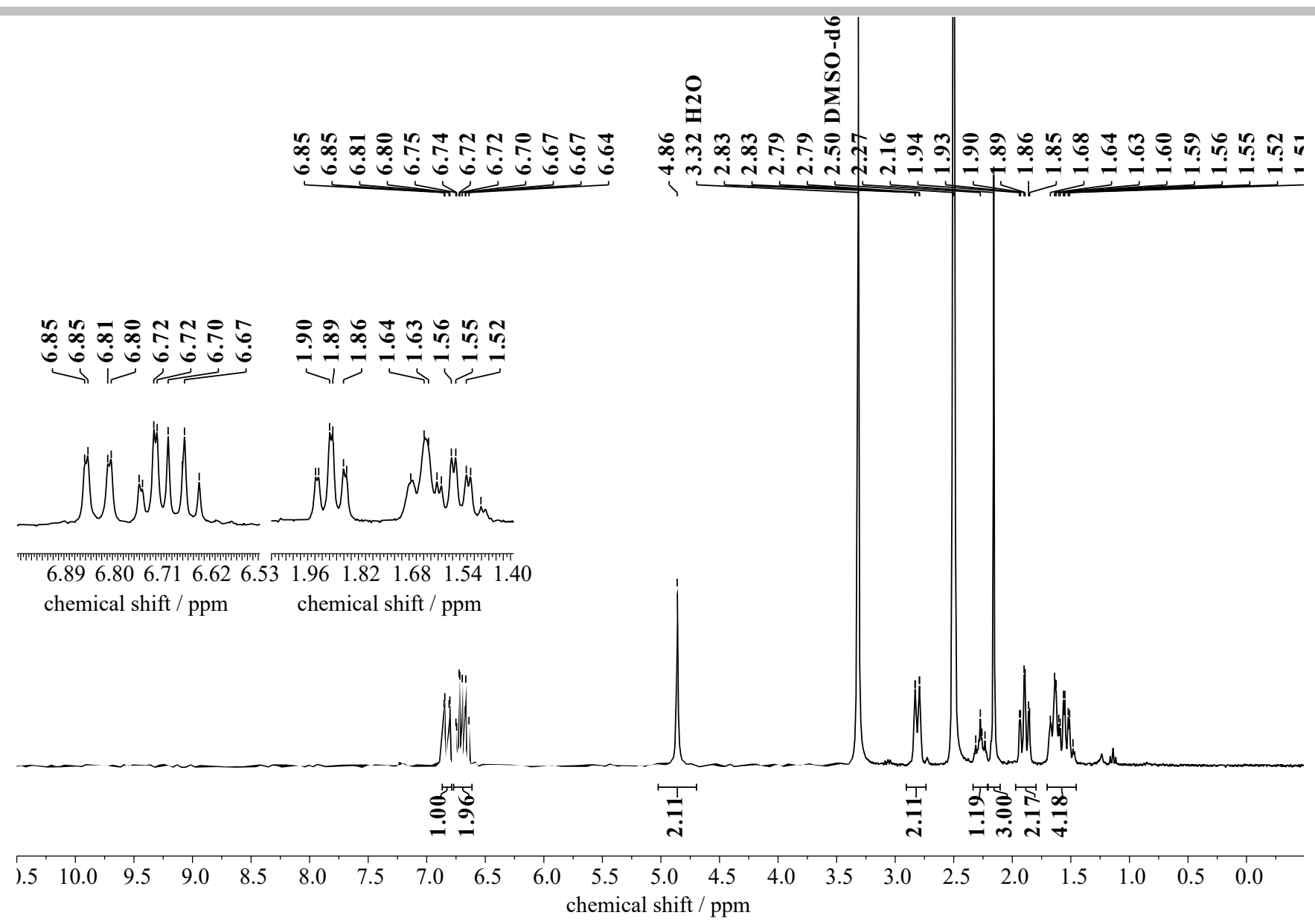


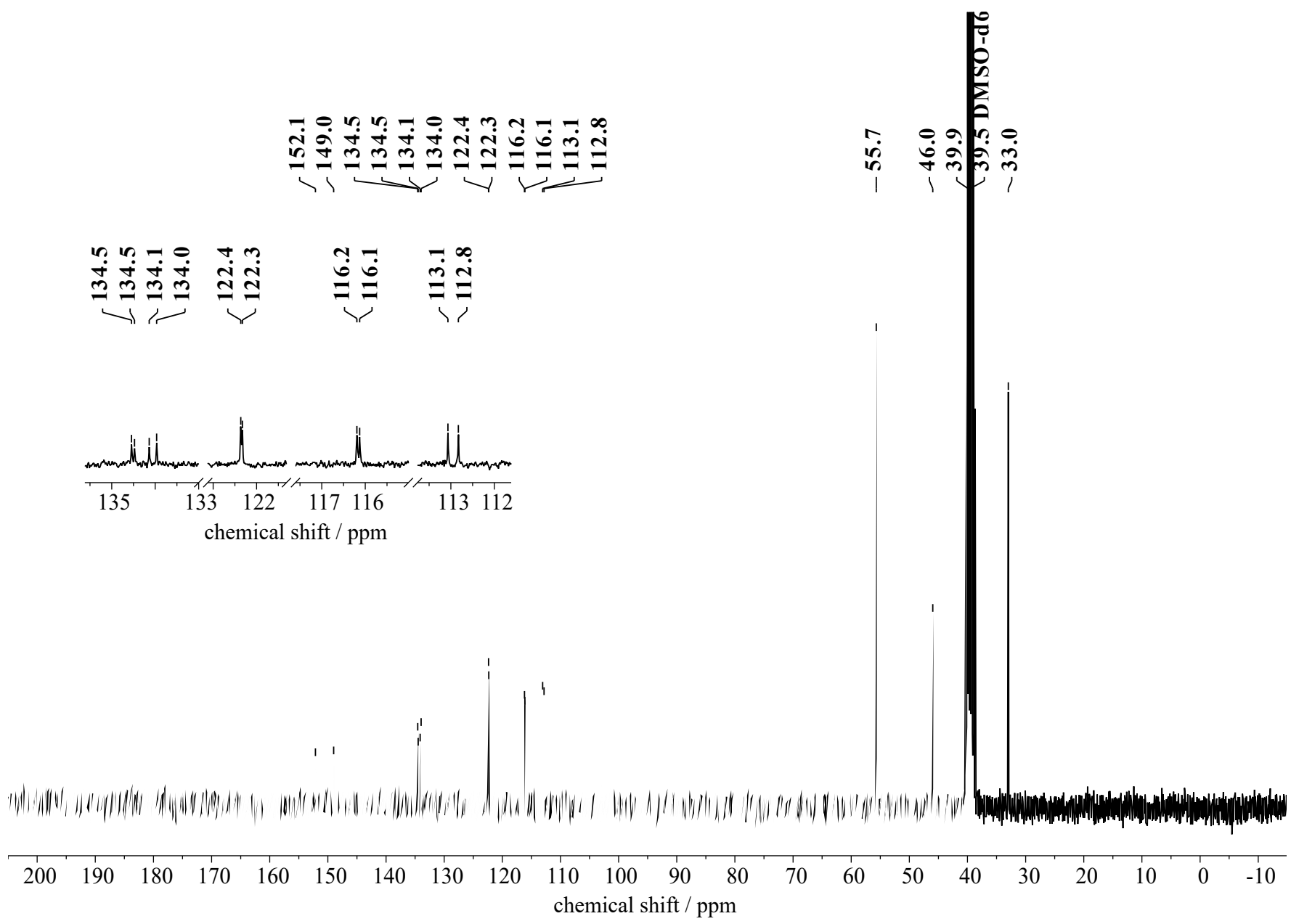


**Figure S75**. $^{1}H$ (300 MHz) and $^{13}C$ (75 MHz) NMR spectra of **39** in DMSO-$d_6$.

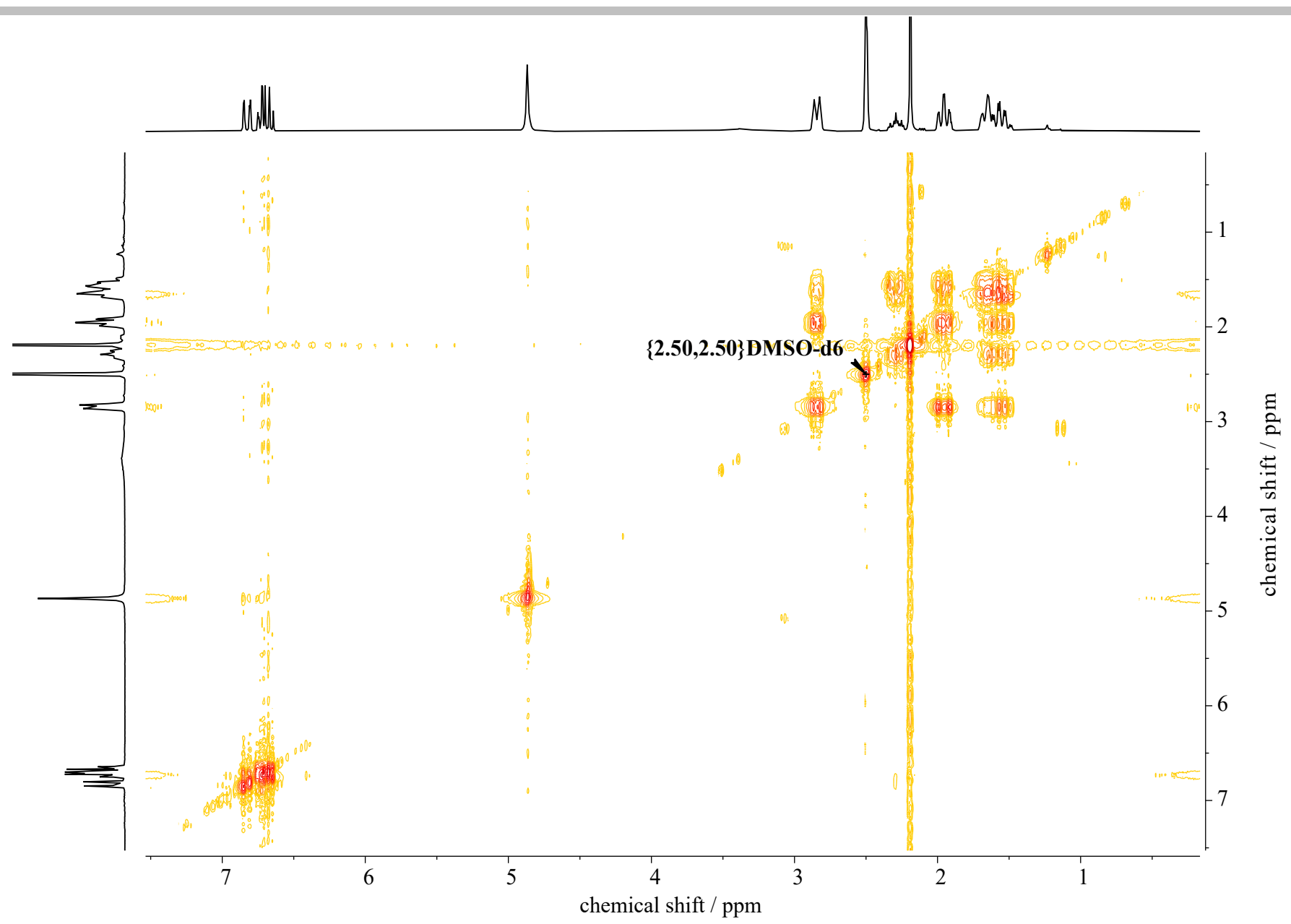


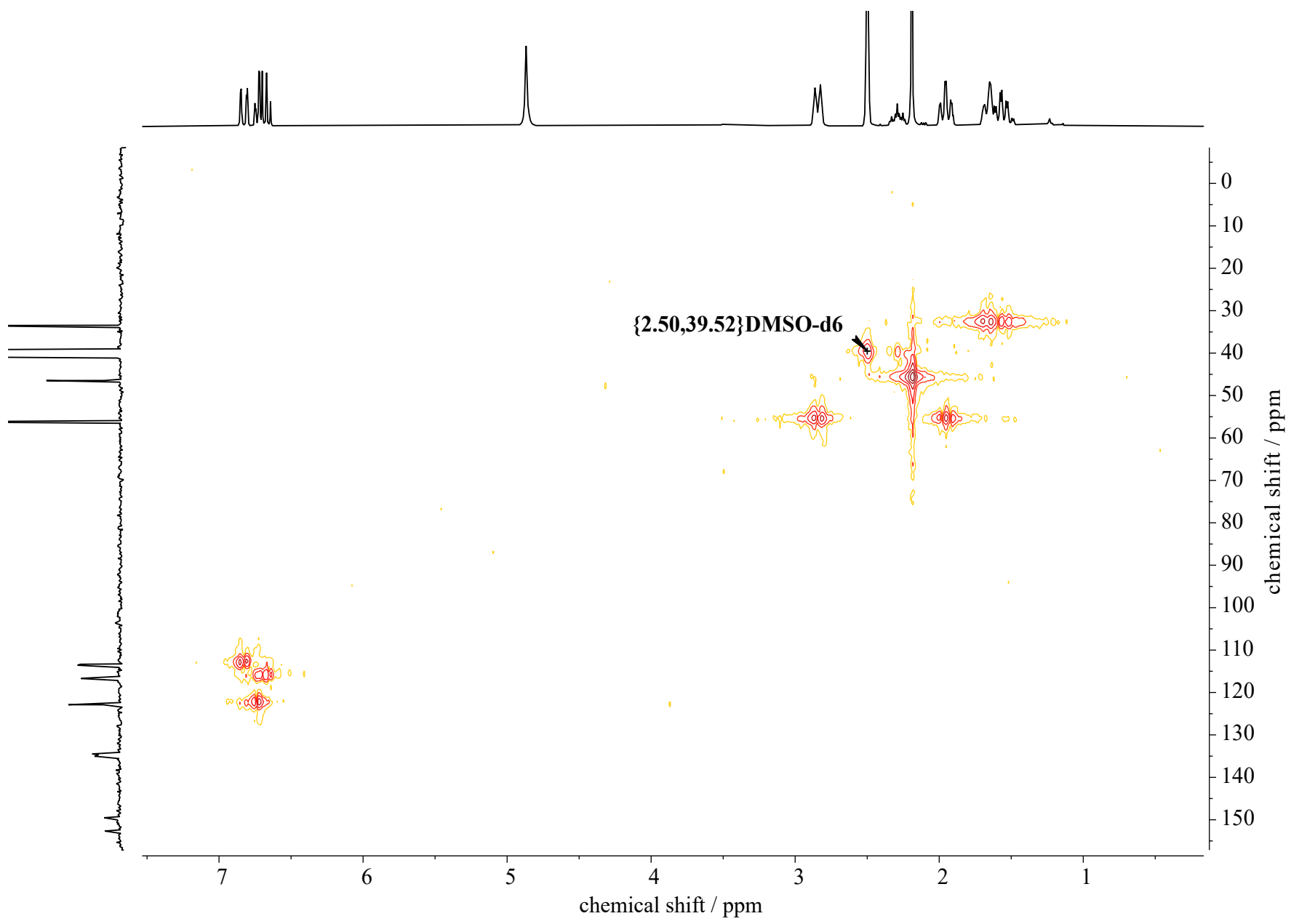


**Figure S76**. COSY and HMQC NMR spectra of **39** in DMSO-$d_6$.

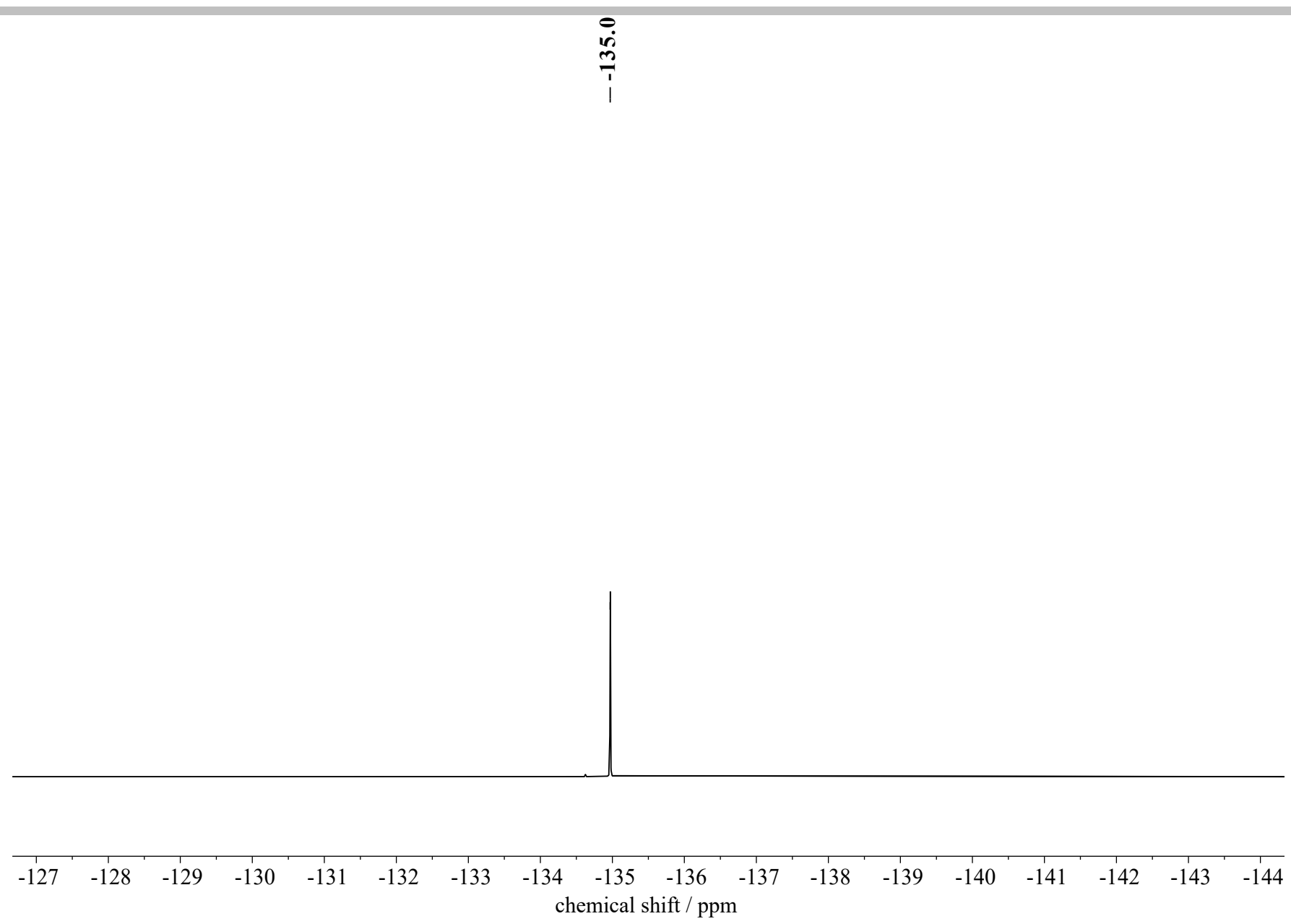


**Figure S77**. $^{19}F$ (282 MHz) NMR spectra of **39** in DMSO-$d_6$.

**Synthesis of 6-((2-fluoro-4-(1-methylpiperidin-4-yl)phenyl)diazenyl)-2-methylbenzo[d]oxazole (10)**

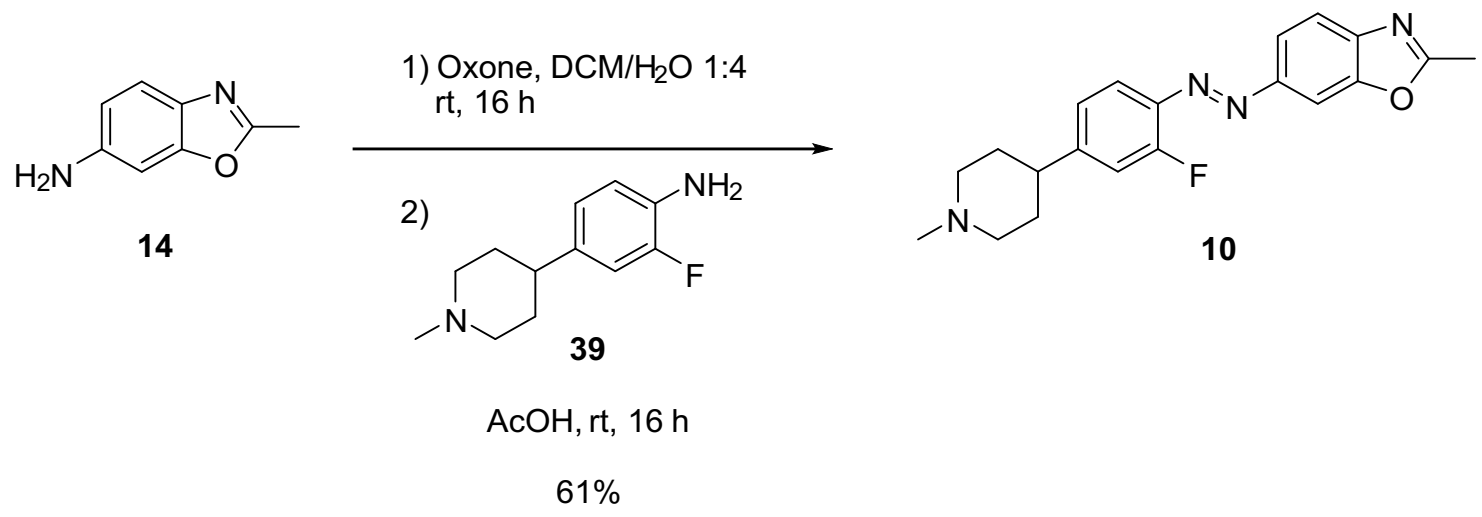


2-Methyl-1,3-benzoxazol-6-amine **14** (57 mg, 384 µmol, 2.00 eq) was dissolved in 5 mL DCM, Oxone® (236 mg, 768 µmol, 4.00 eq) was dissolved in 20 mL distilled water and added to the solution. The resulting mixture was stirred for 16 h at room temperature. The reaction mixture was then diluted with 100 mL DCM and washed with 100 mL 1 M HCl, distilled water and brine. The organic layer was dried over $MgSO_4$ and the solvent was removed under reduced pressure. The resulting brown solid and 2-fluoro-4-(1-methylpiperidin-4-yl)aniline **39** (40 mg, 192 µmol, 1.00 eq) were suspended in 20 mL acetic acid. The reaction mixture was stirred for 16 h at room temperature. The reaction was then diluted with 200 mL distilled water and extracted with 200 mL EtOAc. The organic layer was washed with 200 mL distilled water and brine, dried over $MgSO_4$ and solvent was removed under reduced pressure. The crude product was purified by flash column chromatography (EtOAc/MeOH, 1:1, v%) to yield the desired compound 6-((2-fluoro-4-(1-methylpiperidin-4-yl)phenyl)diazenyl)-2-methylbenzo[*d*]oxazole (**10**, 41 mg, 117 µmol, 61%) as an orange solid.

**TLC:** $R_f$ = 0.12 (EtOAc/MeOH, 1:1, v%).

***trans*-isomer** (*cis*-isomer signals were labeled with * on NMR spectrum).

**$^1$H-NMR (300 MHz, DMSO-*d*$_6$, *δ*):** 8.12 (d, $^4J$ = 1.8 Hz, 1H, $H_{arom.}$), 7.95 (dd, $^3J$ = 8.5 Hz, $^4J$ = 1.8 Hz, 1H, $H_{arom.}$), 7.84 (dd, $^3J$ = 8.5 Hz, 1H, $H_{arom.}$), 7.71 (t, $^3J$ = 8.2 Hz, 1H, $H_{arom.}$), 7.40 (dd, $^3J$ = 12.3 Hz, $^4J$ = 1.8 Hz, 1H, $H_{arom.}$), 7.26 (dd, $^3J$ = 8.3 Hz, $^4J$ = 1.8 Hz, 1H, $H_{arom.}$), 2.90-2.86 (m, 2H, C$H_2$), 2.68 (s, 3H, C$H_3$), 2.60-2.55 (m, 1H, C*H*), 2.20 (s, 3H, C$H_3$), 2.01-1.93 (m, 2H, C$H_2$), 1.82-1.67 (m, 4H, C$H_2$, C$H_2$), ppm.

**$^{13}$C-NMR (75 MHz, DMSO-*d*$_6$, *δ*):** 167.0 (N=*C*-O), 159.4 (d, $^1J_{FC}$ = 256.0 Hz, *C*F), 153.1 (d, $^3J_{FC}$ = 7.3 Hz, $C_{arom.}$), 150.9 ($C_{arom.}$), 149.5 ($C_{arom.}$), 144.0 ($C_{arom.}$),138.0 (d, $^3J_{FC}$ = 8.0 Hz, $C_{arom.}$), 123.5 (d, $^3J_{FC}$ = 3.0 Hz, $C_{arom.}$H), 120.5 ($C_{arom.}$H), 119.4 ($C_{arom.}$H), 117.3 ($C_{arom.}$H), 115.4 (d, $^2J_{FC}$ = 19.5 Hz, $C_{arom.}$H), 104.3 ($C_{arom.}$H), 55.5 (2C, $CH_2$, $CH_2$), 46.1 ($CH_3$), 41.1 (*C*H), 32.4 (2C, $CH_2$, $CH_2$), 14.4 ($CH_3$) ppm.

**$^{19}$F-NMR (282 MHz, DMSO-*d*$_6$, *δ*)**: -124.4 (C*F*) ppm.

**HRMS-ESI (m/z):** $[M+H]^+$ calcd for $C_{20}H_{22}FN_4O$, 353.1772; found, 353.1782.

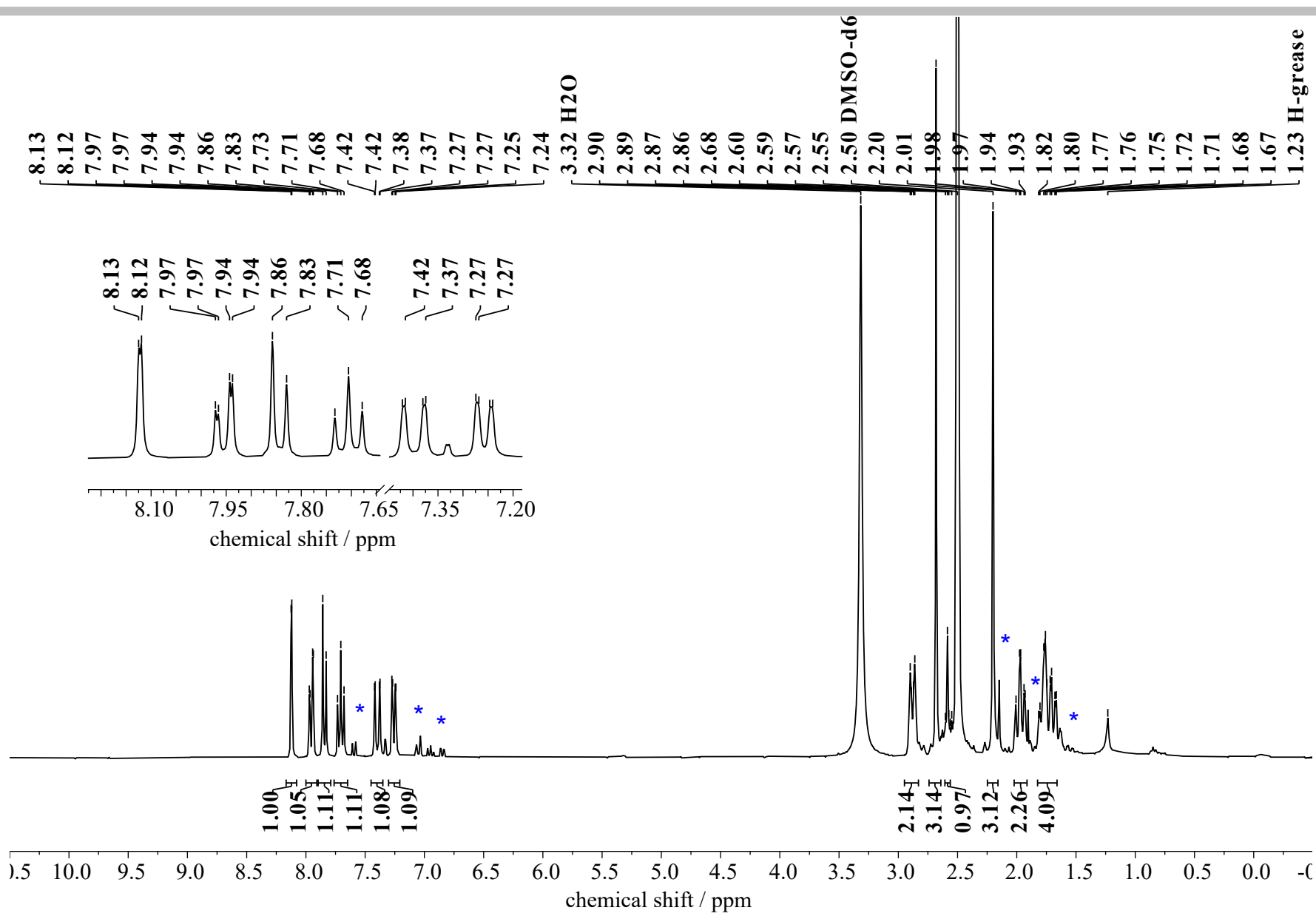

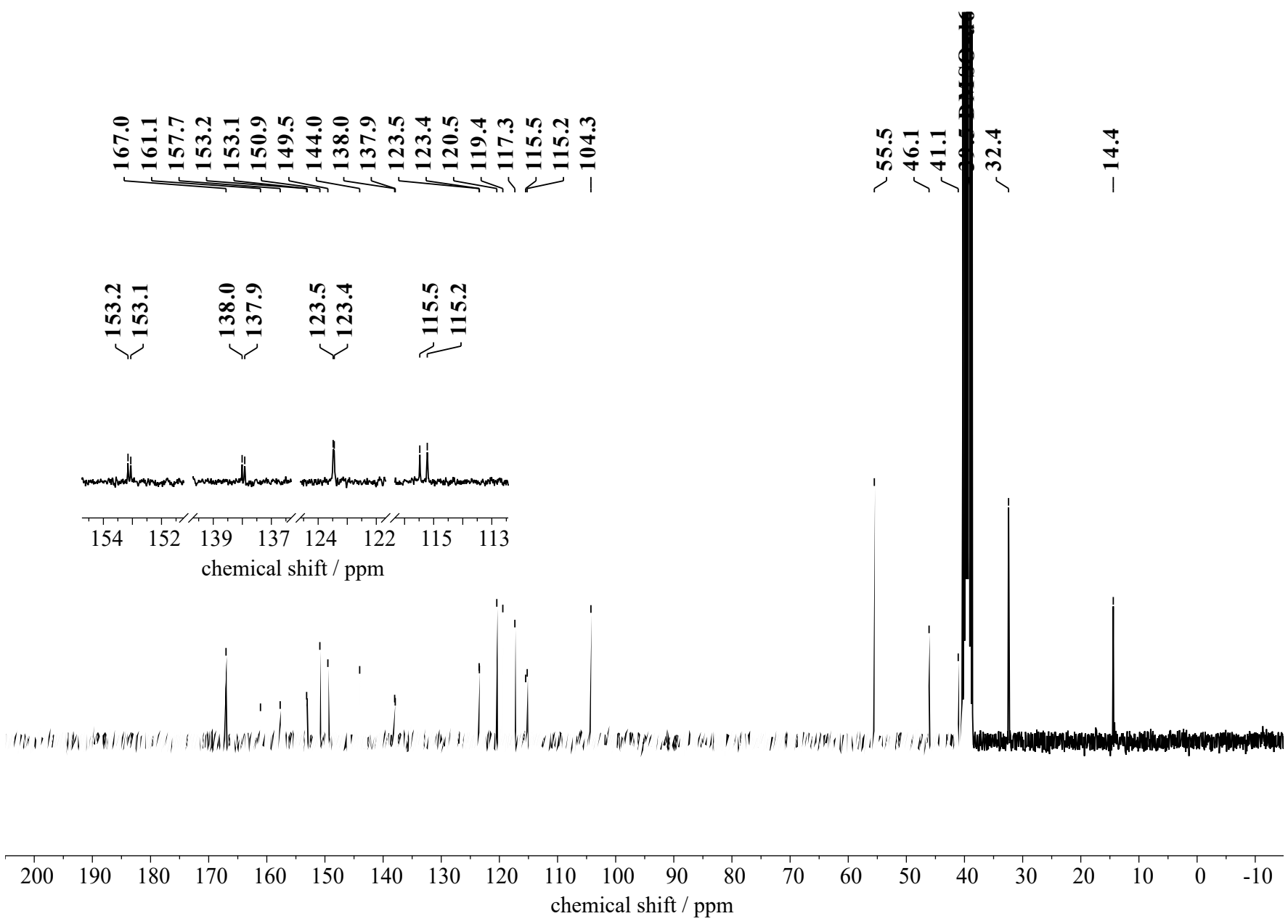

**Figure S78**. $^1H$ (300 MHz) and $^{13}C$ (75 MHz) NMR spectra of **10** in DMSO-$d_6$.

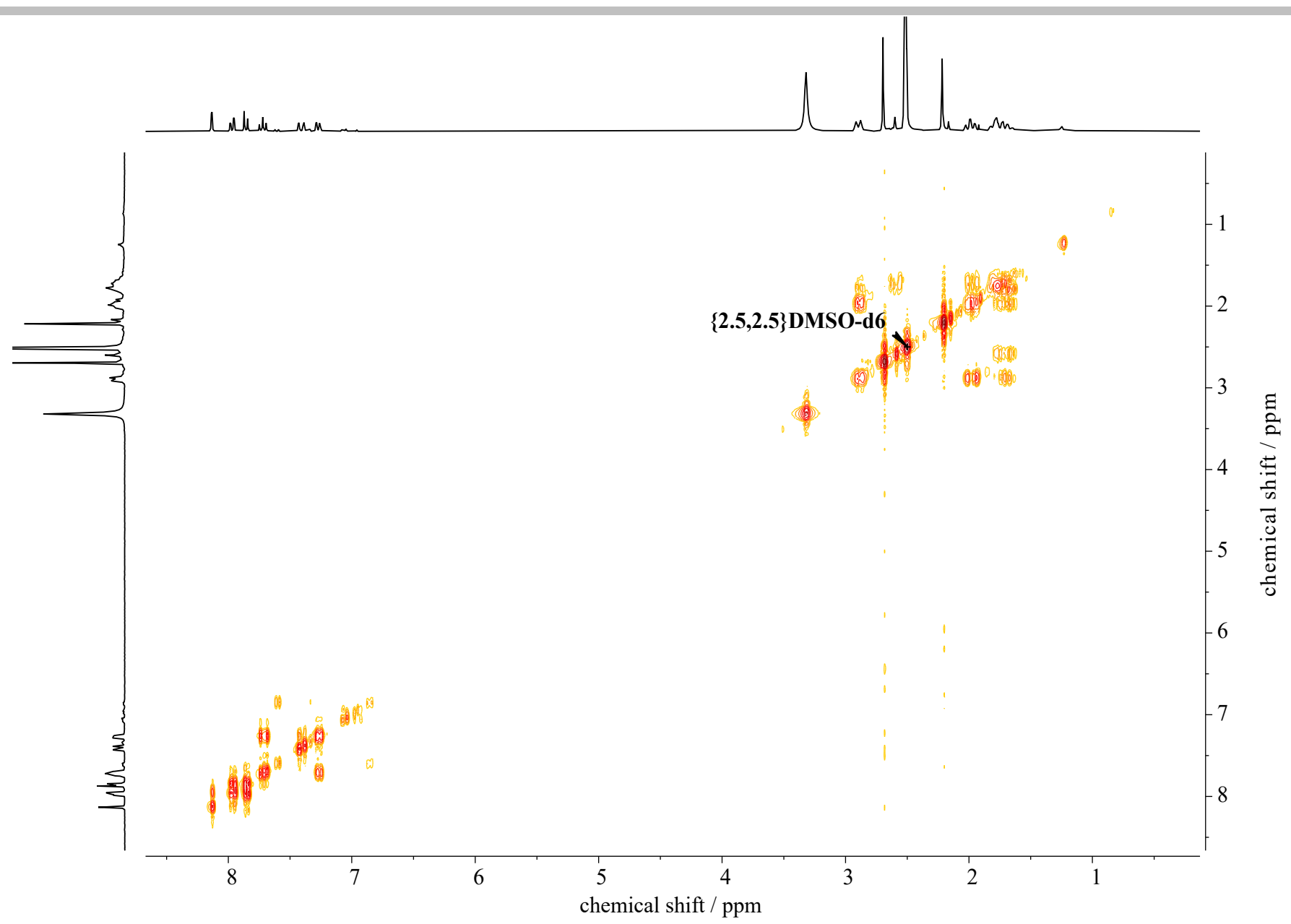


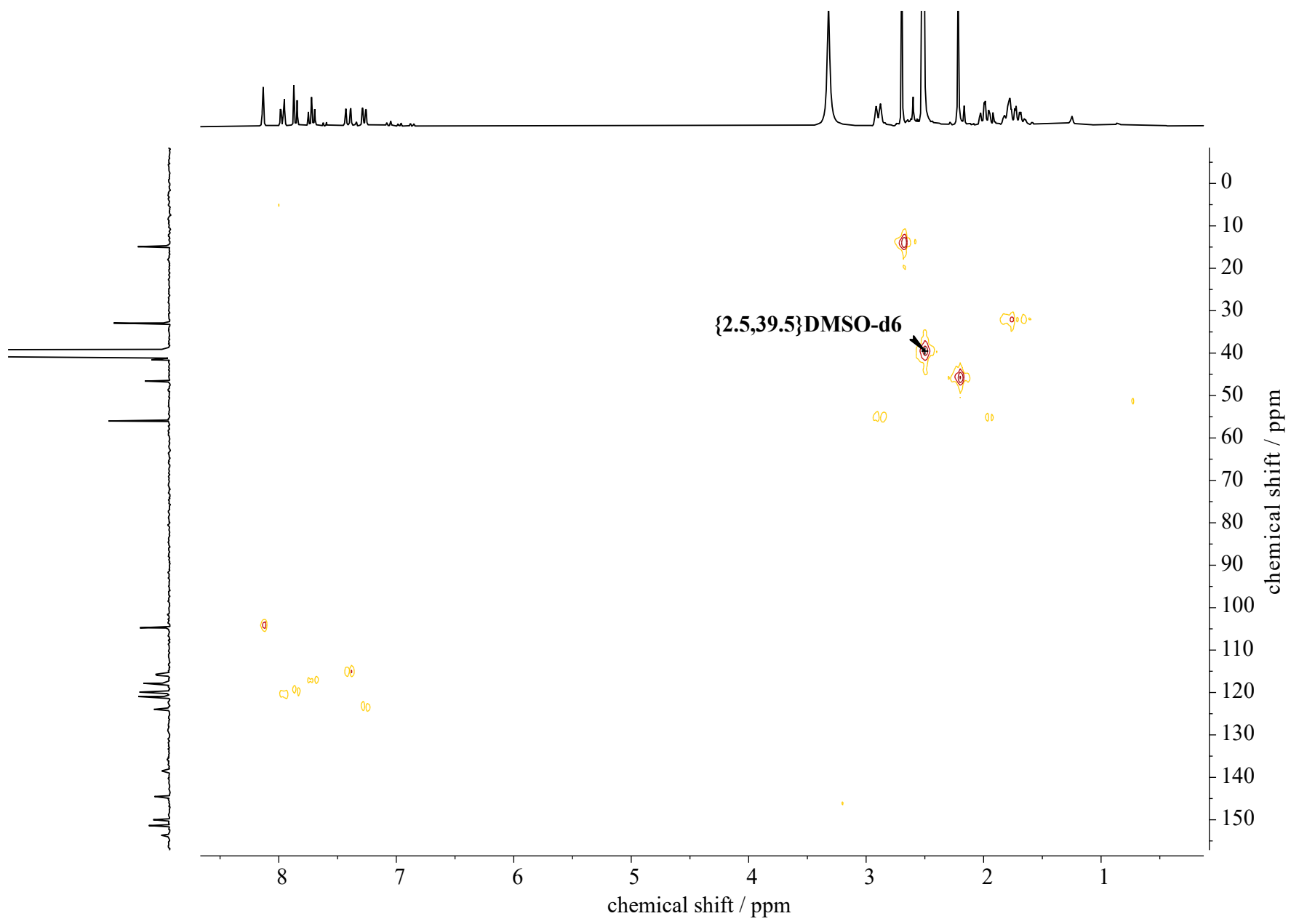


**Figure S79**. COSY and HMQC NMR spectra of **10** in DMSO-$d_6$.

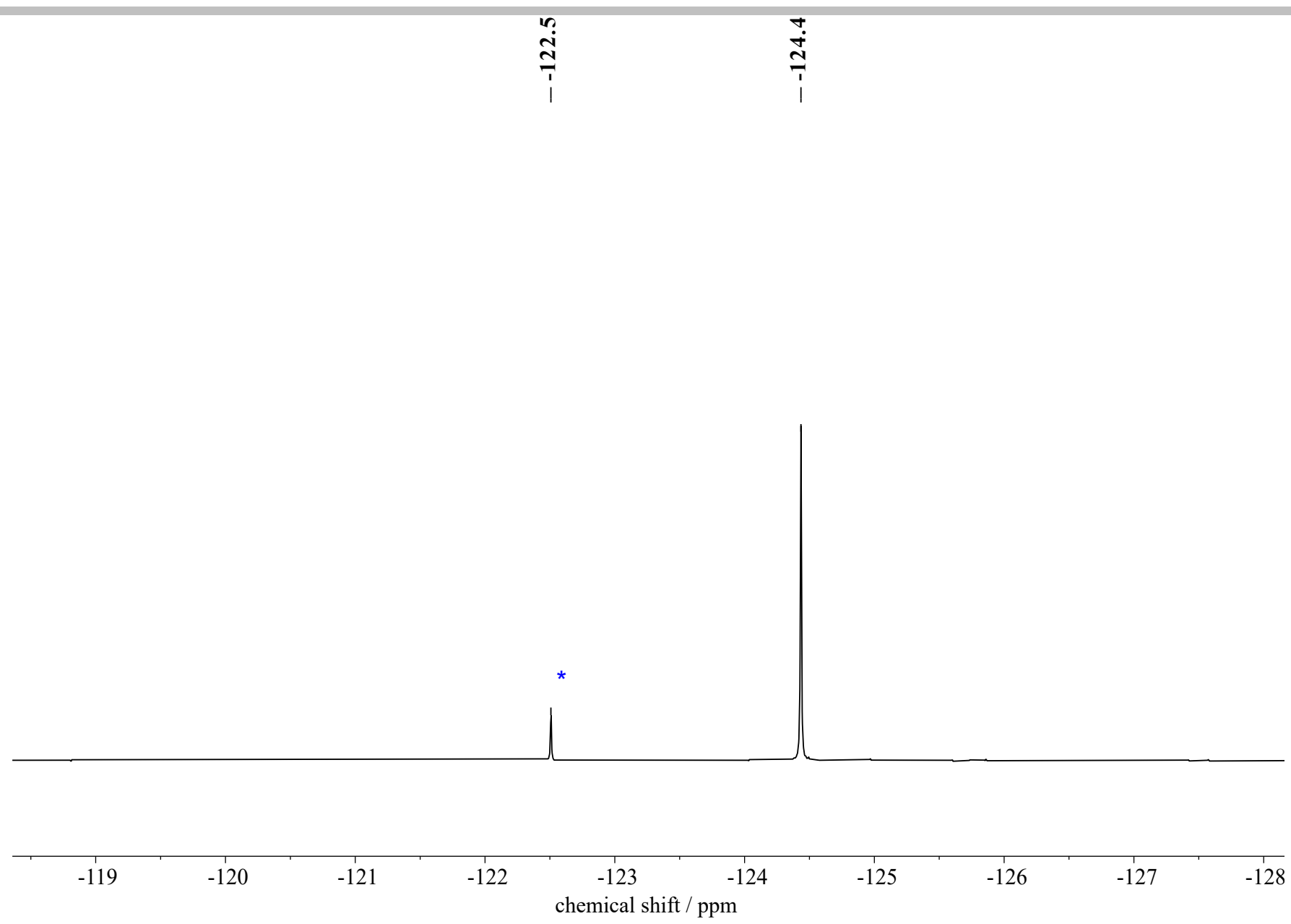


**Figure S80**. $^{19}F$ (282 MHz) NMR spectra of **10** in DMSO-$d_6$.

**Synthesis of 4-((4-bromo-2-fluorophenyl)diazenyl)-2-fluorophenol (41)**

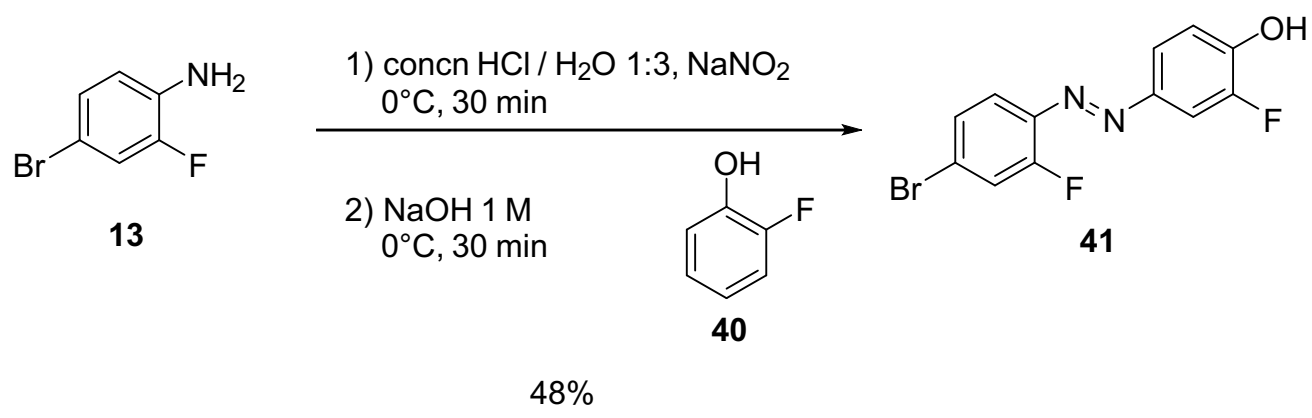

4-Bromo-2-fluoroaniline **13** (500 mg, 2.63 mmol, 1.00 eq) was dissolved in mixture of concn HCl/$H_2O$ (16 mL, 1:3, v%) at 0 °C. Sodium nitrite (236 mg, 3.42 mmol, 1.30 eq) was dissolved in 16 mL distilled water and added dropwise. The resulting mixture was stirred for 30 min at 0 °C. The pH value was then adjusted to 11 by adding 1 M aqueous NaOH. 2-Fluorophenol **40** (380 mg, 3.42 mmol, 1.30 eq) was added and the mixture was stirred for further 30 min at 0 °C. The reaction mixture was then acidified to pH 1 with concn HCl and the orange precipitate was filtered and washed with distilled water. The crude product was purified by flash column chromatography (DCM) to yield the desired compound 4-((4-bromo-2-fluorophenyl)diazenyl)-2-fluorophenol (**41**, 397 mg, 1.64 mmol, 48%) as an orange solid.
**TLC:** $R_f$ = 0.37 (DCM).
***trans*-isomer** (*cis*-isomer signals were labeled with * on NMR spectrum).
**$^1$H-NMR (300 MHz, DMSO-*d*$_6$, *δ*):** 10.99 (s, 1H, O*H*), 7.84 (dd, $^3J$ = 10.4 Hz, $^4J$ = 2.0 Hz, 1H, $H_{arom.}$), 7.73-7.59 (m, 3H, $H_{arom.}$, $H_{arom.}$, $H_{arom.}$), 7.56-7.52 (m, 1H, $H_{arom.}$), 7.16 (t, $^3J$ = 8.8 Hz, 1H, $H_{arom.}$) ppm.
**$^{13}$C-NMR (75 MHz, DMSO-*d*$_6$, *δ*):** 158.7 (d, $^1J_{FC}$ = 259.7 Hz, *C*F), 153.0 (d, $^1J_{FC}$ = 245.3 Hz, *C*F), 145.1 (d, $^3J_{FC}$ = 5.4 Hz, $C_{arom.}$), 138.9 (d, $^3J_{FC}$ = 6.9 Hz, $C_{arom.}$), 129.2 ($C_{arom.}$), 128.3 (d, $^3J_{FC}$ = 3.6 Hz, $C_{arom.}$), 124.6 (d, $^3J_{FC}$ = 9.6 Hz, $C_{arom.}$H), 123.0 (d, $^3J_{FC}$ = 2.5 Hz, $C_{arom.}$H), 120.6 (d, $^2J_{FC}$ = 23.1 Hz, $C_{arom.}$H), 119.0 ($C_{arom.}$H), 117.7 (d, $^3J_{FC}$ = 3.5 Hz, $C_{arom.}$H), 108.7 (d, $^2J_{FC}$ = 19.1 Hz, $C_{arom.}$H) ppm.
**$^{19}$F-NMR (282 MHz, DMSO-*d*$_6$, *δ*):** -122.3 (C*F*), -134.4 (C*F*) ppm.
**HRMS-ESI (m/z):** $[M-H]^-$ calcd for $C_{12}H_6BrF_2N_2O$, 310.9637; found, 310.9637.

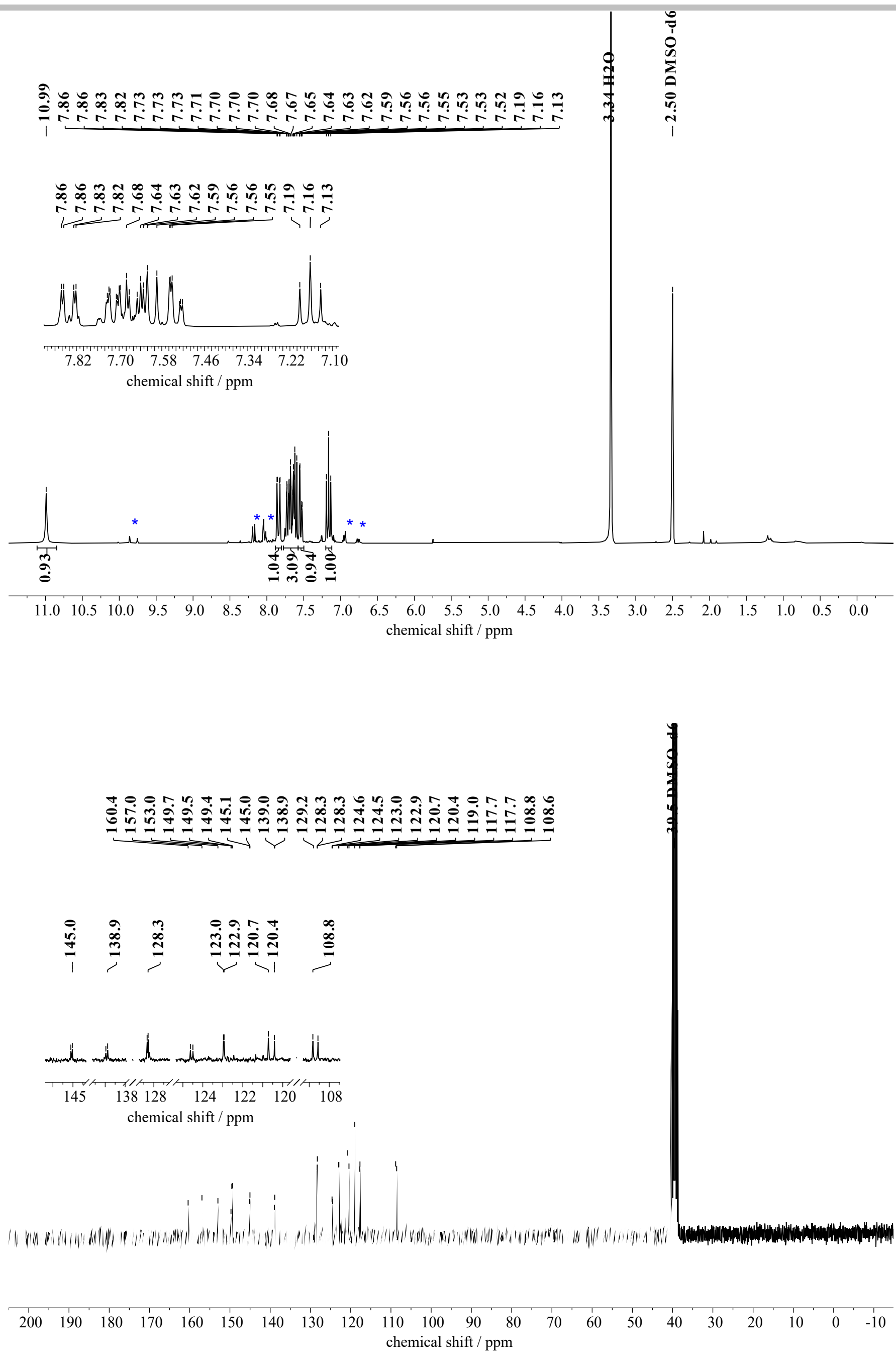

**Figure S81**. $^{1}$H (300 MHz) and $^{13}$C (75 MHz) NMR spectra of **41** in DMSO-$d_6$.

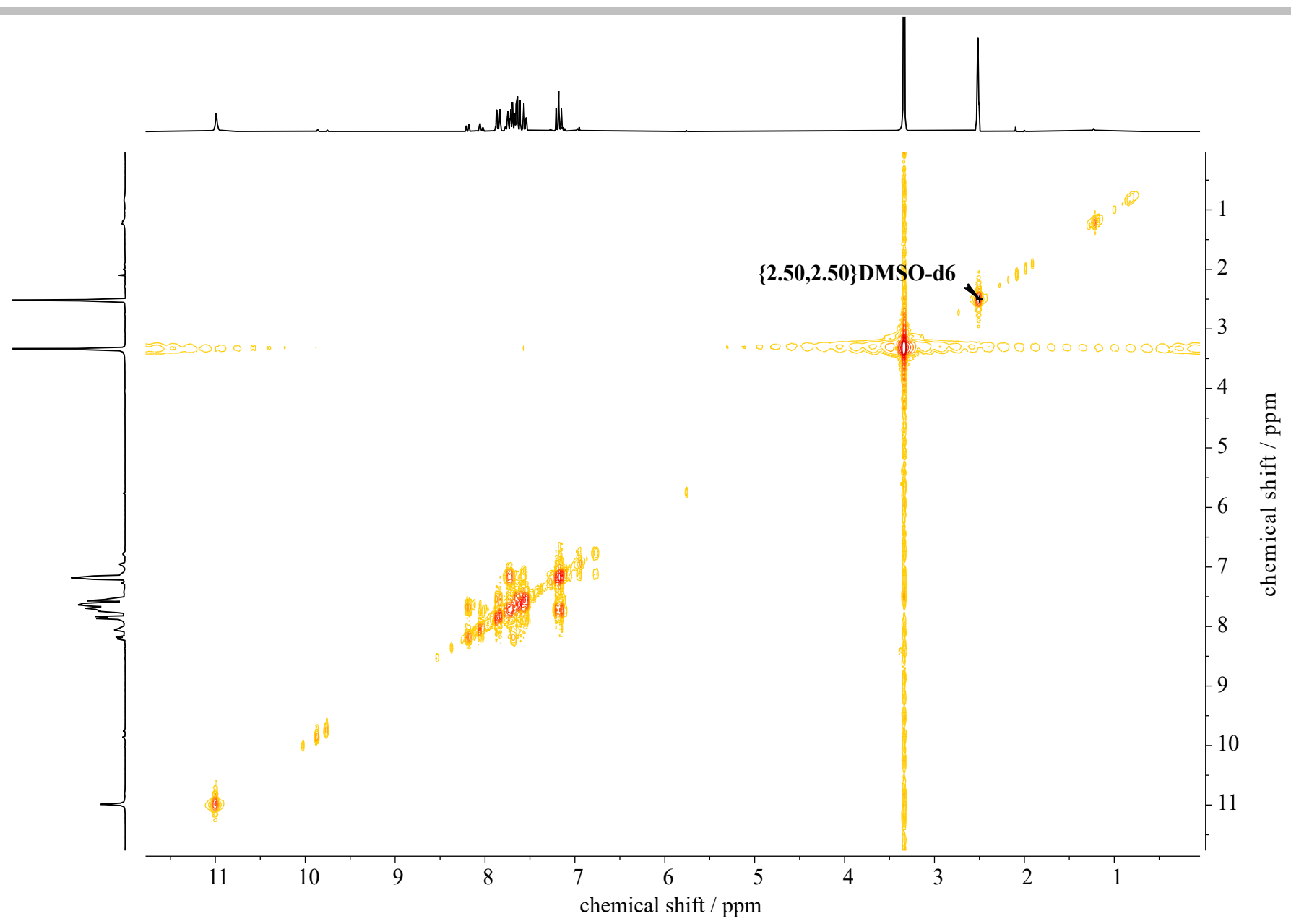


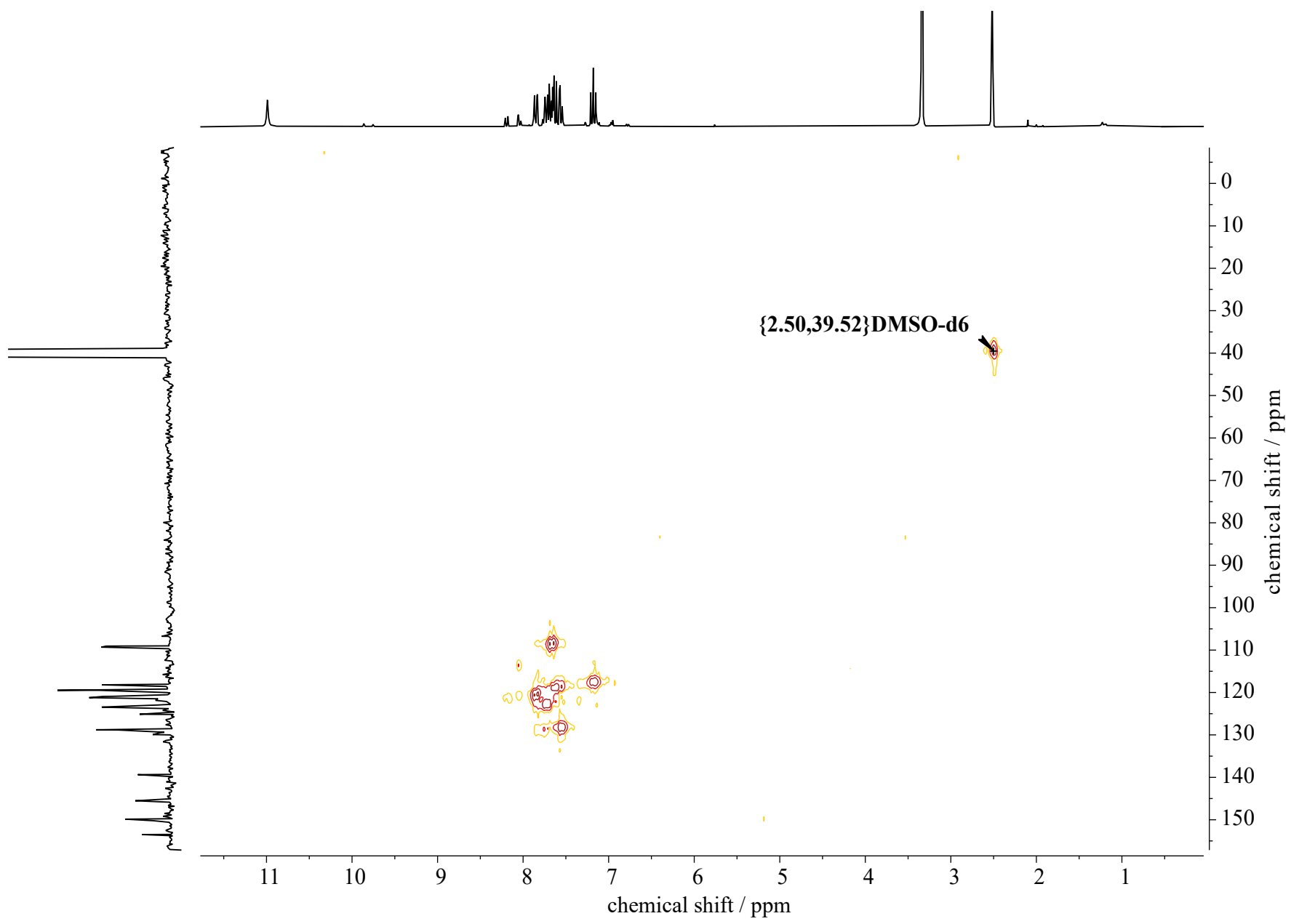


**Figure S82**. COSY and HMQC NMR spectra of **41** in DMSO-$d_6$.

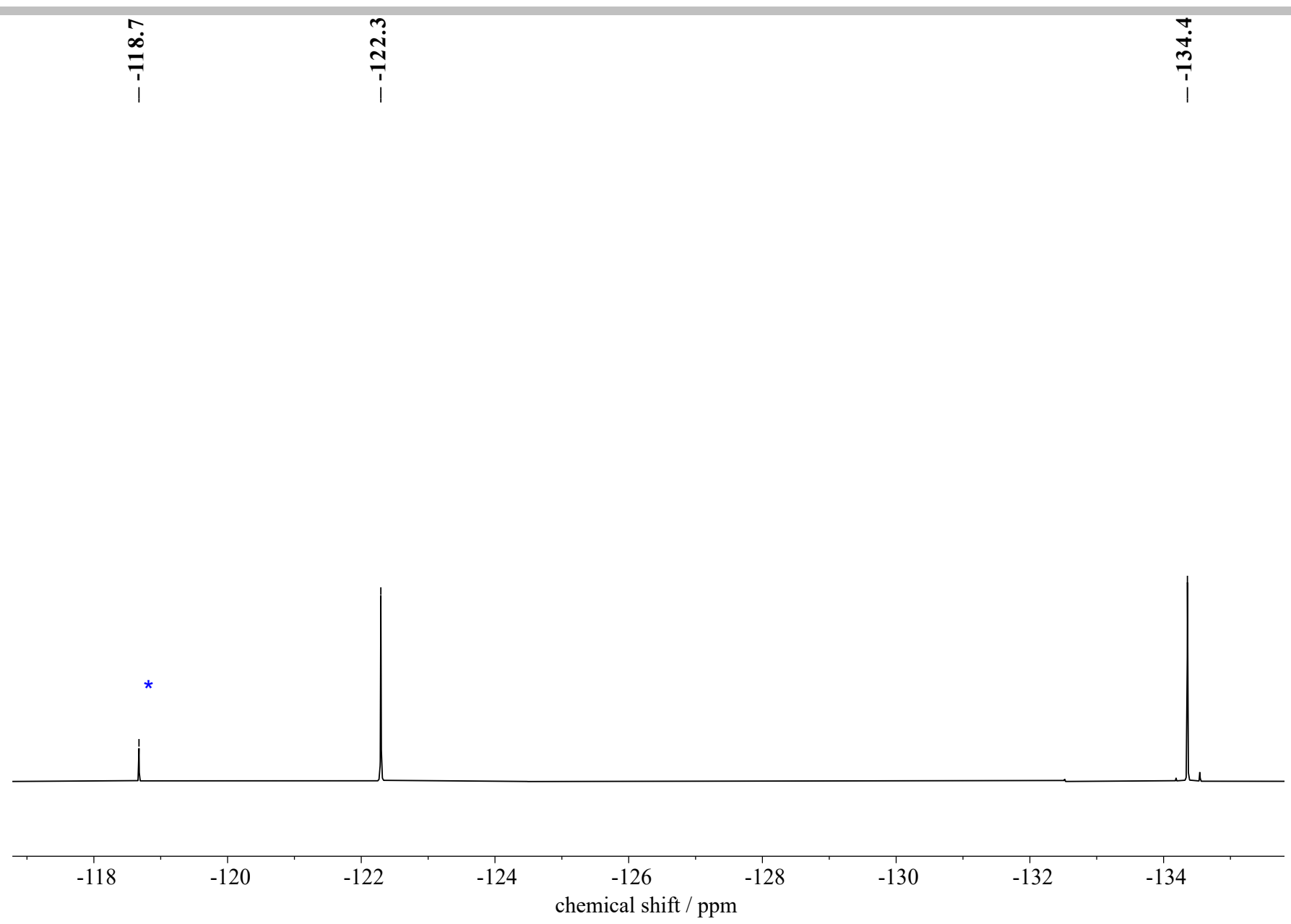


**Figure S83**. $^{19}$F (282 MHz) NMR spectra of **41** in DMSO-$d_6$.

**Synthesis of 1-(4-bromo-2-fluorophenyl)-2-(3-fluoro-4-methoxyphenyl)diazene (42)**

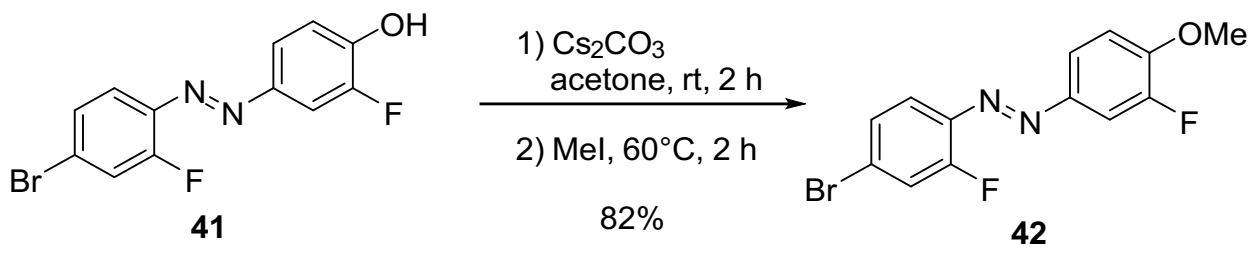

4-((4-Bromo-2-fluorophenyl)diazenyl)-2-fluorophenol **41** (500 mg, 1.60 mmol, 1.00 eq) was dissolved in 20 mL acetone, and cesium carbonate (1.04 g, 3.19 mmol, 2.00 eq) was added. The resulting suspension was stirred for 2 h at room temperature. Iodomethane (0.30 mL, 4.79 µmol, 3.00 eq) was added and the reaction mixture was refluxed at 60 °C for 2 h. Reaction mixture was cooled to room temperature. The reaction was then diluted with 100 mL distilled water and extracted with 100 mL EtOAc. The organic layer was washed with 100 mL distilled water and brine, dried over $MgSO_4$ and solvent was removed under reduced pressure. The crude product was purified by flash column chromatography (DCM) to yield the desired compound 1-(4-bromo-2-fluorophenyl)-2-(3-fluoro-4-methoxyphenyl)diazene (**42**, 429 mg, 1.31 mmol, 82%) as an orange solid.
**TLC:** $R_f$ = 0.40 (DCM).
**$^1$H-NMR (300 MHz, DMSO-$d_6$, δ):** 7.88-7.83 (m, 2H, $H_{arom.}$), 7.70-7.61 (m, 2H, $H_{arom.}$, $H_{arom.}$), 7.57-7.53 (m, 1H, $H_{arom.}$), 7.41 (t, $^3J$ = 8.9 Hz, 1H, $H_{arom.}$), 3.97 (s, 3H, C$H_3$) ppm.
**$^{13}$C-NMR (75 MHz, DMSO-$d_6$, δ):** 158.8 (d, $^1J_{FC}$ = 260.3 Hz, *C*F), 153.5 (d, $^1J_{FC}$ = 248.3 Hz, *C*F), 150.8 (d, $^2J_{FC}$ = 11.2 Hz, $C_{arom.}$), 145.8 (d, $^3J_{FC}$ = 5.5 Hz, $C_{arom.}$), 138.8 (d, $^3J_{FC}$ = 7.1 Hz, $C_{arom.}$), 128.6 (d, $^3J_{FC}$ = 3.8 Hz, $C_{arom.}$), 125.0 (d, $^3J_{FC}$ = 9.4 Hz, $C_{arom.}$H), 123.6 (d, $^3J_{FC}$ = 2.8 Hz, $C_{arom.}$H), 120.6 (d, $^2J_{FC}$ = 23.0 Hz, $C_{arom.}$H), 119.0 ($C_{arom.}$H), 113.7 (d, $^3J_{FC}$ = 2.2 Hz, $C_{arom.}$H), 107.4 (d, $^2J_{FC}$ = 19.0 Hz, $C_{arom.}$H), 56.5 ($CH_3$) ppm.
**$^{19}$F-NMR (282 MHz, DMSO-$d_6$, δ):** -122.0 (C*F*), -133.1 (C*F*) ppm.
**HRMS-ESI (m/z):** $[M+H]^+$ calcd for $C_{13}H_{10}BrF_2N_2O$, 326.9939; found, 326.9946.

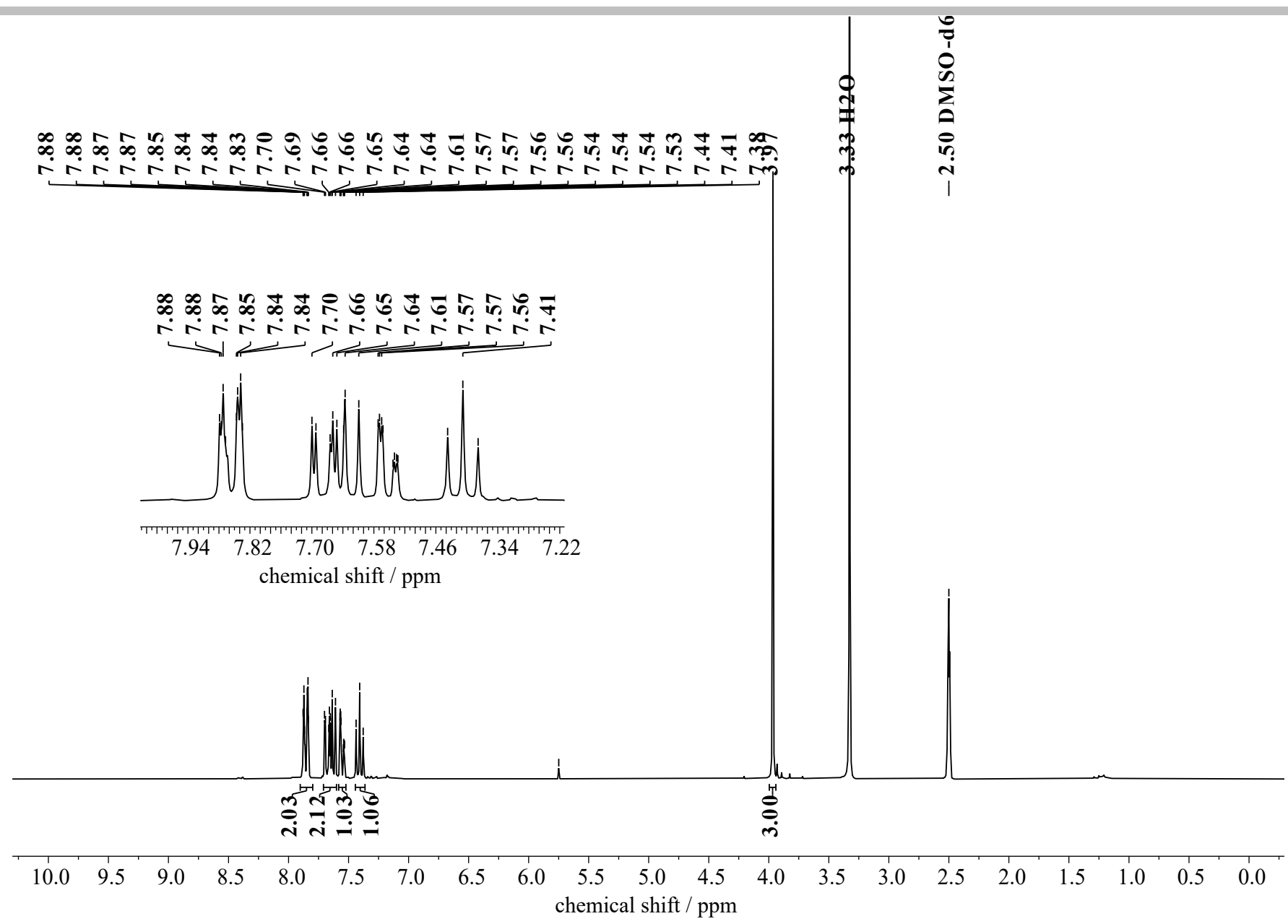


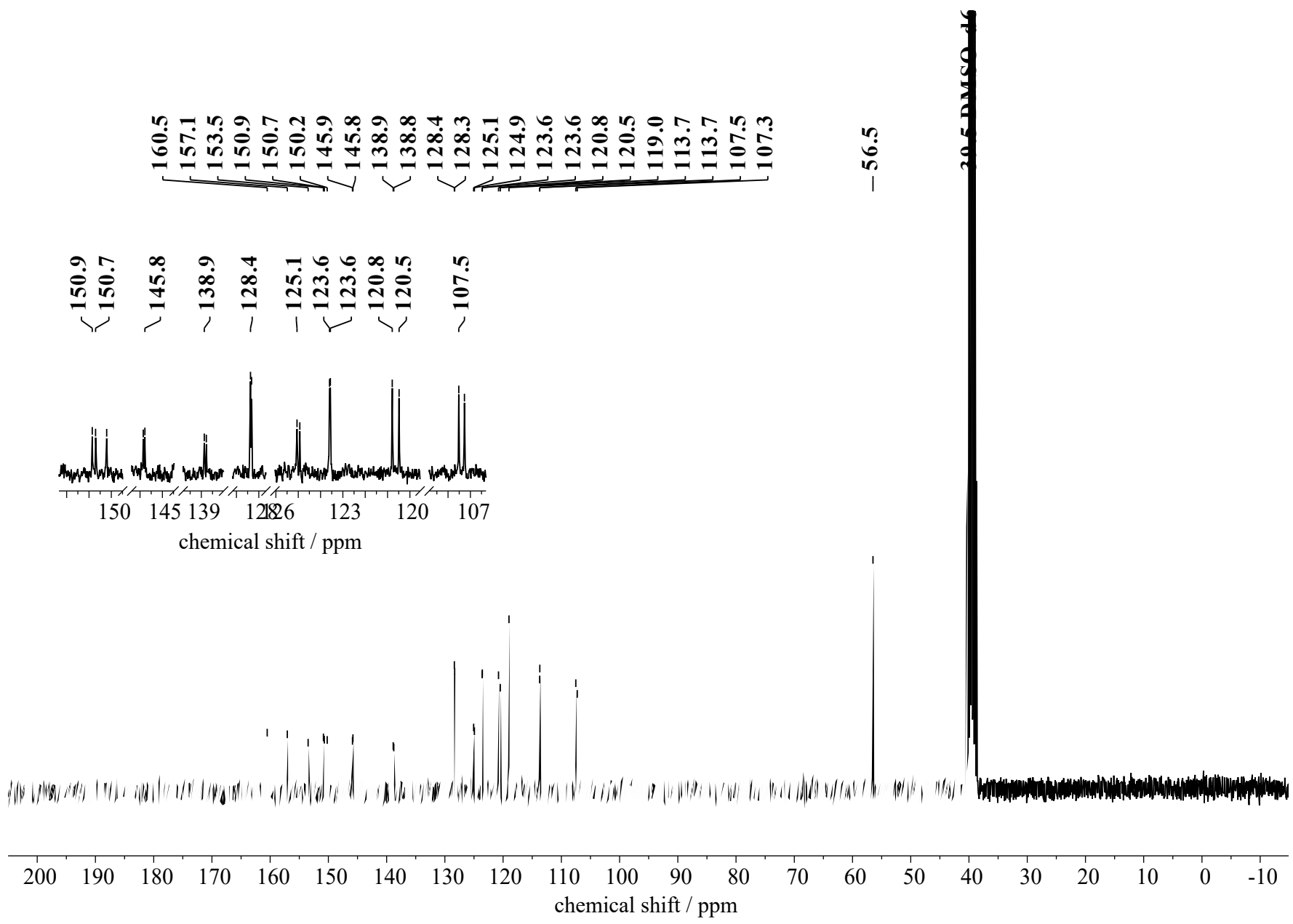


**Figure S84**. $^{1}H$ (300 MHz) and $^{13}C$ (75 MHz) NMR spectra of **42** in DMSO-$d_6$.

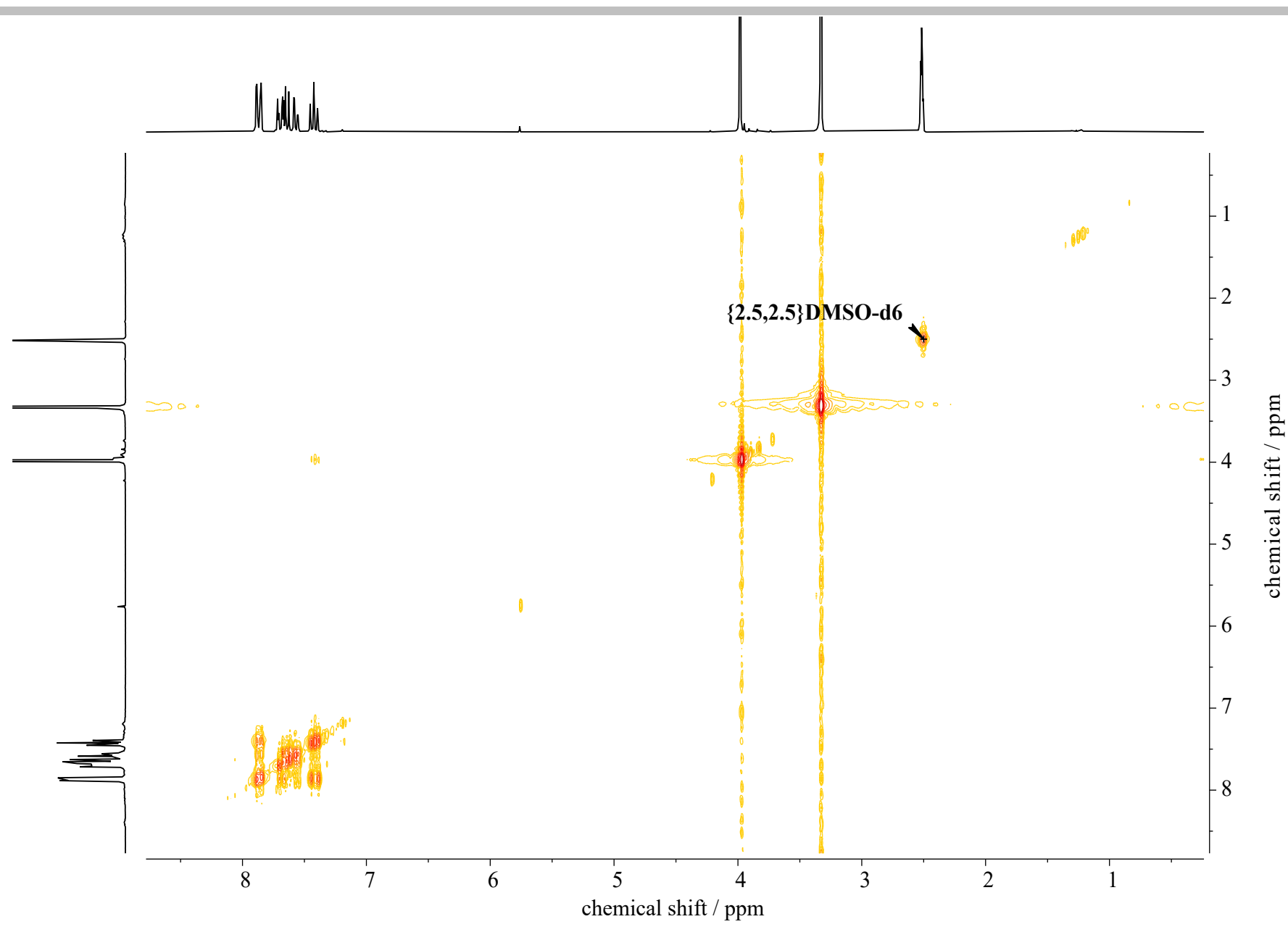


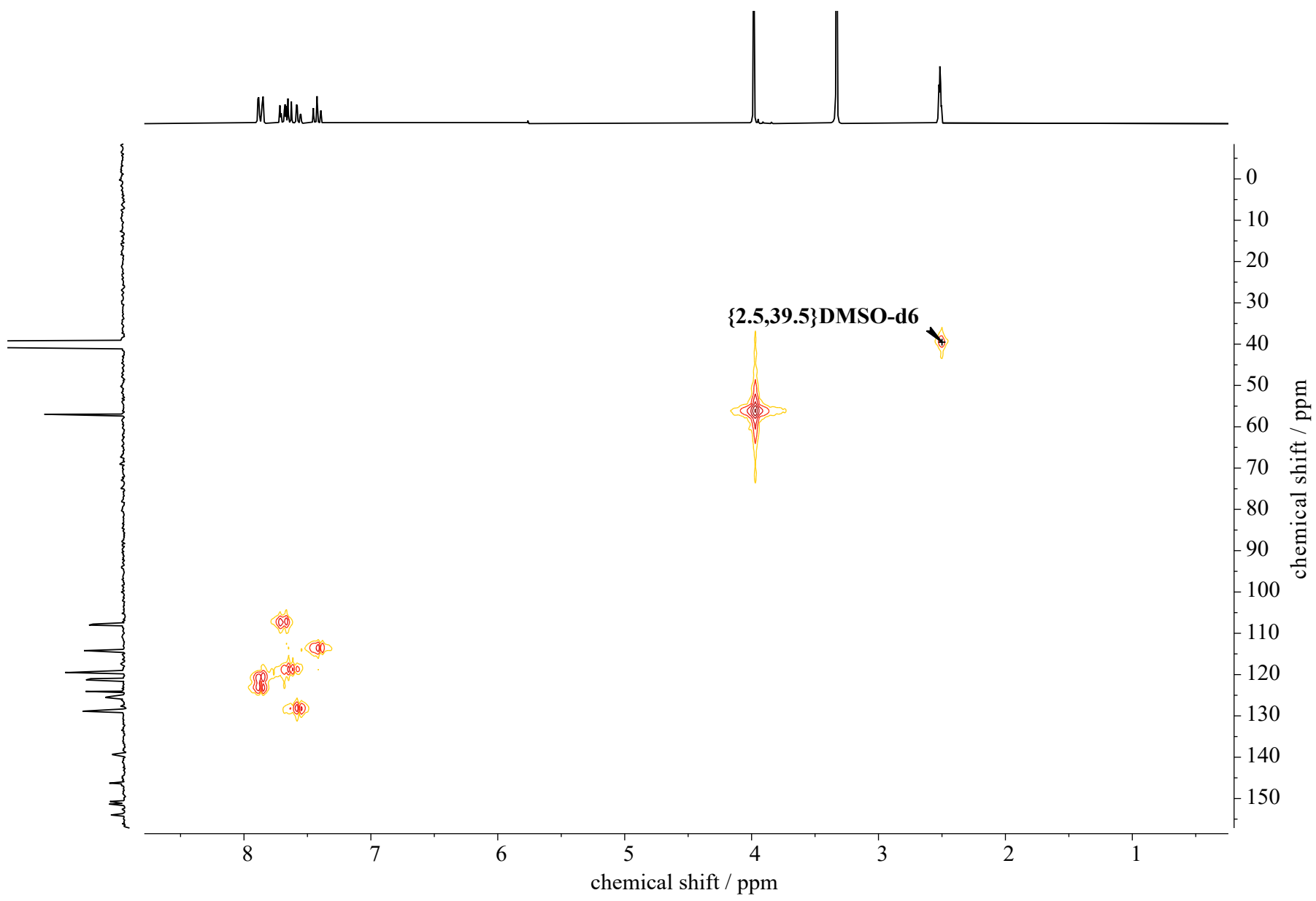


**Figure S85**. COSY and HMQC NMR spectra of **42** in DMSO-*d*6.

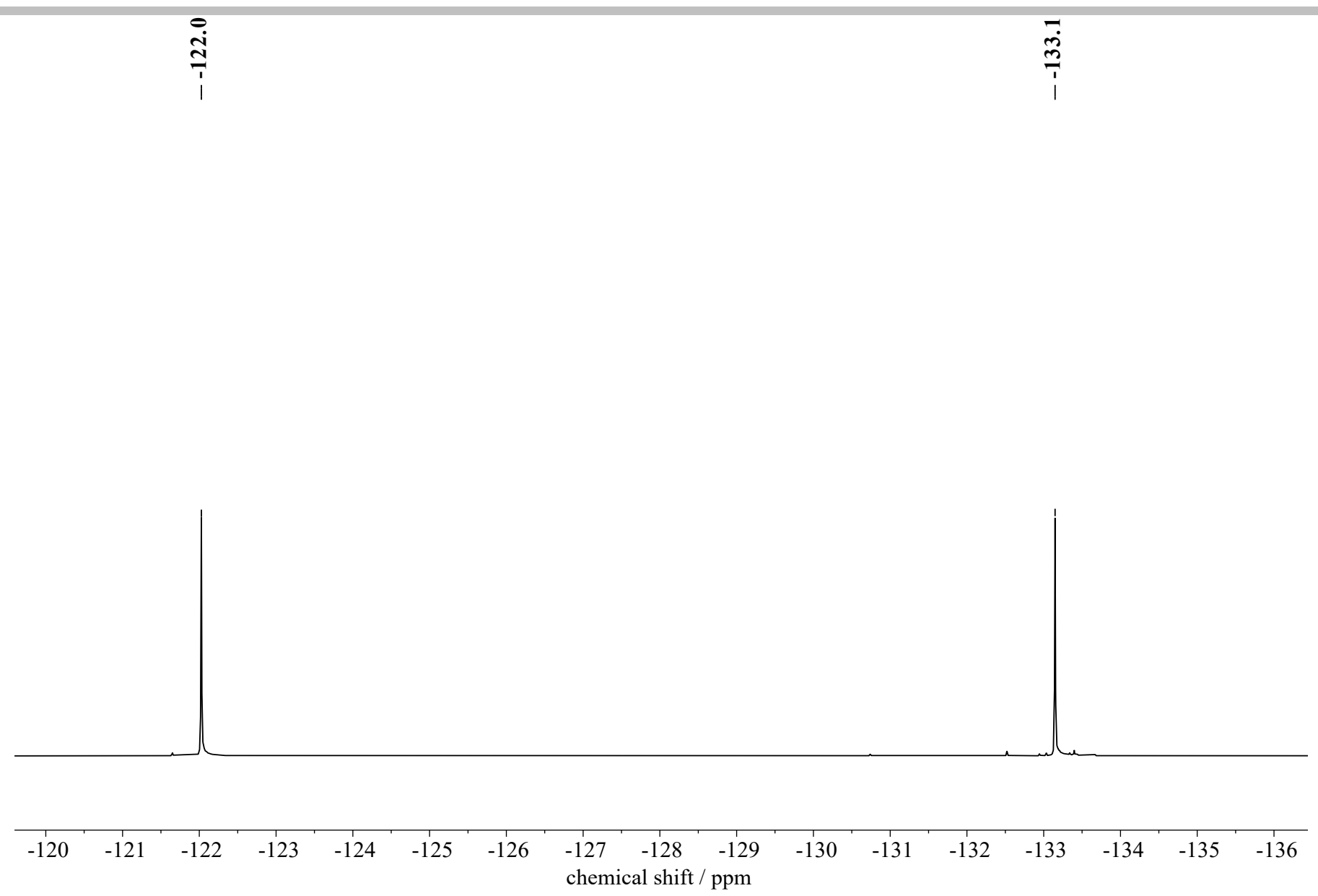


**Figure S86**. $^{19}F$ (282 MHz) NMR spectra of **42** in DMSO-$d_6$.

**Synthesis of tert-butyl-4-(3-fluoro-4-((3-fluoro-4-methoxyphenyl)diazenyl)phenyl)-3,6-dihydropyridine-1(2H)-carboxylate (43)**

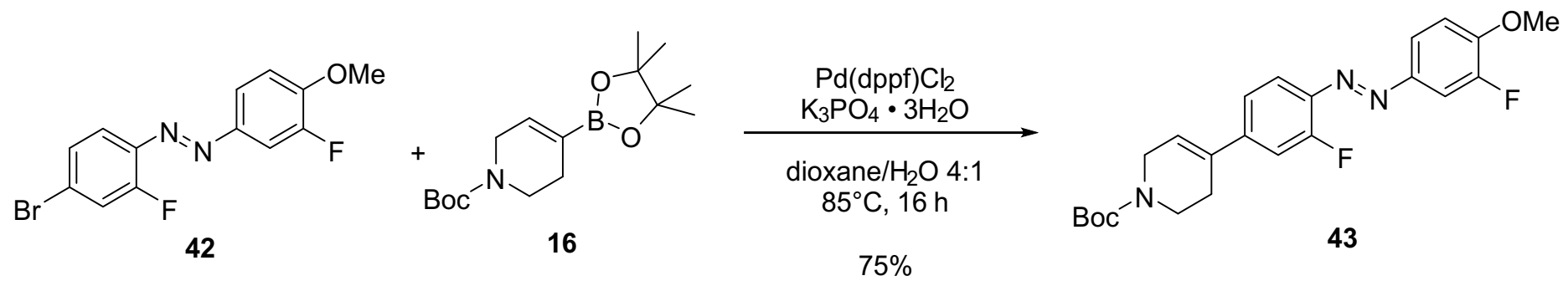


1-(4-Bromo-2-fluorophenyl)-2-(3-fluoro-4-methoxyphenyl)diazene **42** (100 mg, 306 µmol, 1.00 eq), *N*-*tert*-butoxycarbony-1,2,3,6-tetrahydropyridine-4-boronic acid pinacol ester **16** (95 mg, 306 µmol, 1.00 eq), Pd(dppf)$Cl_2$ (23 mg, 30.6 µmol, 0.10 eq) and $K_3PO_4{\cdot}3H_2O$ (244 mg, 917 µmol, 3.00 eq) were dissolved in mixture of dioxane/distilled water (20 mL, 4:1, v%). The reaction was stirred under $N_2$ atmosphere at 85 °C for 16 h. The reaction was then diluted with 100 mL distilled water and extracted with 100 mL EtOAc. The organic layer was washed with 100 mL distilled water and brine, dried over $MgSO_4$ and solvent was removed under reduced pressure. The crude product was purified by flash column chromatography (EtOAc) to yield the desired compound *tert*-butyl-4-(3-fluoro-4-((3-fluoro-4-methoxyphenyl)diazenyl)phenyl)-3,6-dihydropyridine-1(*2H*)-carboxylate (**43**, 101 mg, 230 µmol, 75%) as an orange solid.
**TLC:** $R_f$ = 0.60 (EtOAc).
**$^1$H-NMR (300 MHz, DMSO-*d*$_6$, *δ*):** 7.87-7.82 (m, 1H, $H_{arom.}$), 7.72-7.65 (m, 2H, $H_{arom.}$, $H_{arom.}$), 7.55 (dd, $^3J$ = 12.7 Hz, $^4J$ = 1.9 Hz, 1H, $H_{arom.}$), 7.46-7.38 (m, 2H, $H_{arom.}$, $H_{arom.}$), 6.44 (s, 1H, C*H*=C), 4.06-4.03 (m, 2H, C$H_2$), 3.97 (s, 3H, C$H_3$), 3.55 (t, $^3J$ = 5.6 Hz, 2H, C$H_2$), 2.52-2.51 (m, 2H, C$H_2$), 1.43 (s, 9H, 3xC$H_3$) ppm.
**$^{13}$C-NMR (75 MHz, DMSO-*d*$_6$, *δ*):** 159.4 (d, $^1J_{FC}$ = 255.1 Hz, *C*F), 153.8 ($C_{arom.}$), 153.5 (d, $^1J_{FC}$ = 237.7 Hz, *C*F), 150.5 ($C_{arom.}$), 150.2 ($C_{arom.}$), 146.0 (d, $^3J_{FC}$ = 5.3 Hz, $C_{arom.}$), 144.8 (d, $^3J_{FC}$ = 8.1 Hz, $C_{arom.}$), 138.3 (d, $^3J_{FC}$ = 7.2 Hz, $C_{arom.}$), 132.9 (d, $^3J_{FC}$ = 2.2 Hz, $C_{arom.}$), 124.4 (*C*H=C), 123.2 (d, $^3J_{FC}$ = 2.7 Hz, $C_{arom.}$H), 121.1 (d, $^3J_{FC}$ = 2.8 Hz, $C_{arom.}$H), 117.4 ($C_{arom.}$H), 113.7 (d, $^3J_{FC}$ = 2.3 Hz, $C_{arom.}$H), 113.1 (d, $^2J_{FC}$ = 20.5 Hz, $C_{arom.}$H), 107.3 (d, $^2J_{FC}$ = 19.0 Hz, $C_{arom.}$H), 79.0 ($C_{q.}$), 56.5 ($CH_3$), 43.4 ($CH_2$), 28.1 (3C, 3x$CH_3$), 26.2 ($CH_2$) ppm.
**$^{19}$F-NMR (282 MHz, DMSO-*d*$_6$, *δ*):** -124.4 (C*F*), -133.3 (C*F*) ppm.
**HRMS-ESI (m/z):** $[M+H]^+$ calcd for $C_{23}H_{26}F_2N_3O_3$, 430.1937; found, 430.1945.

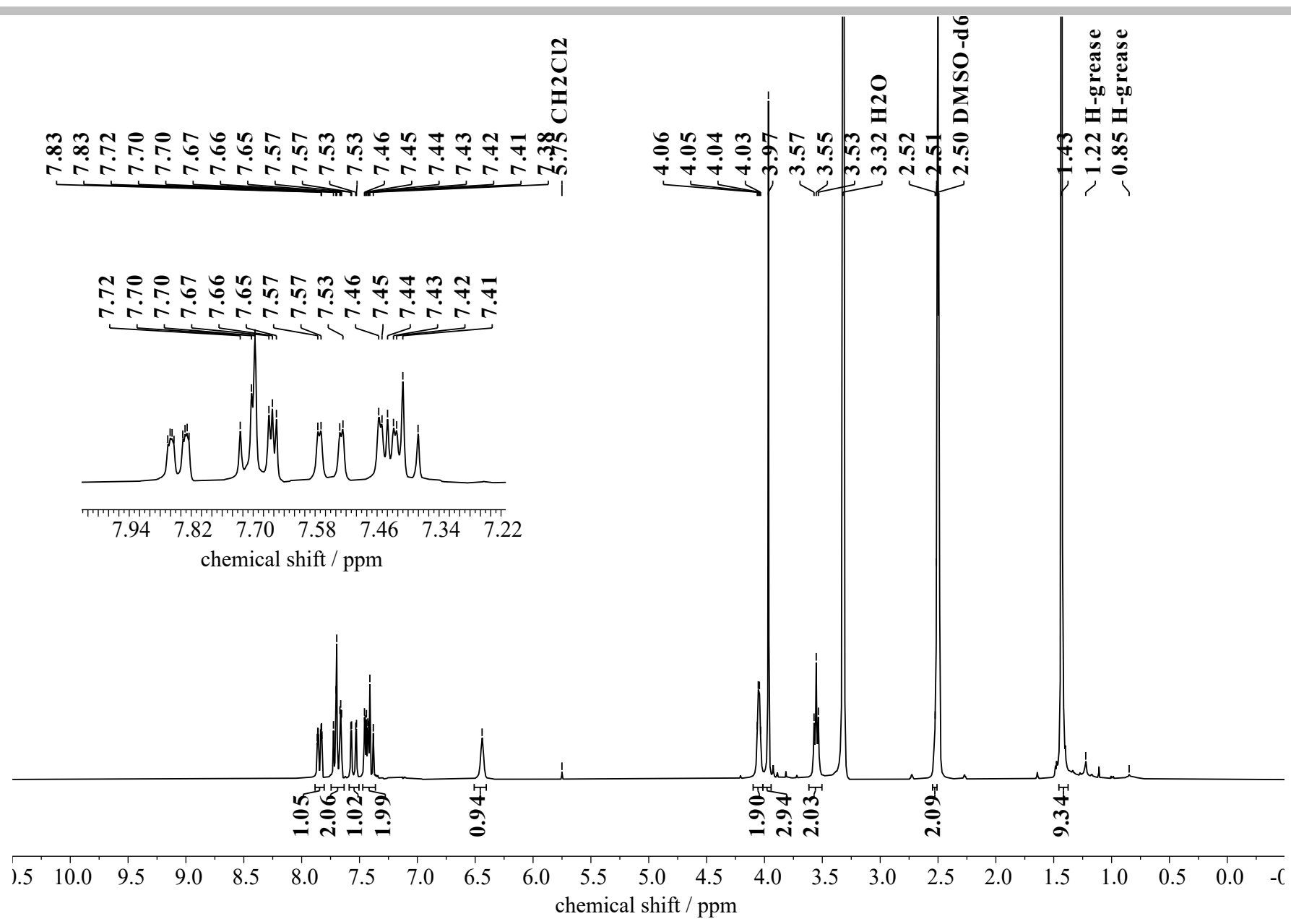


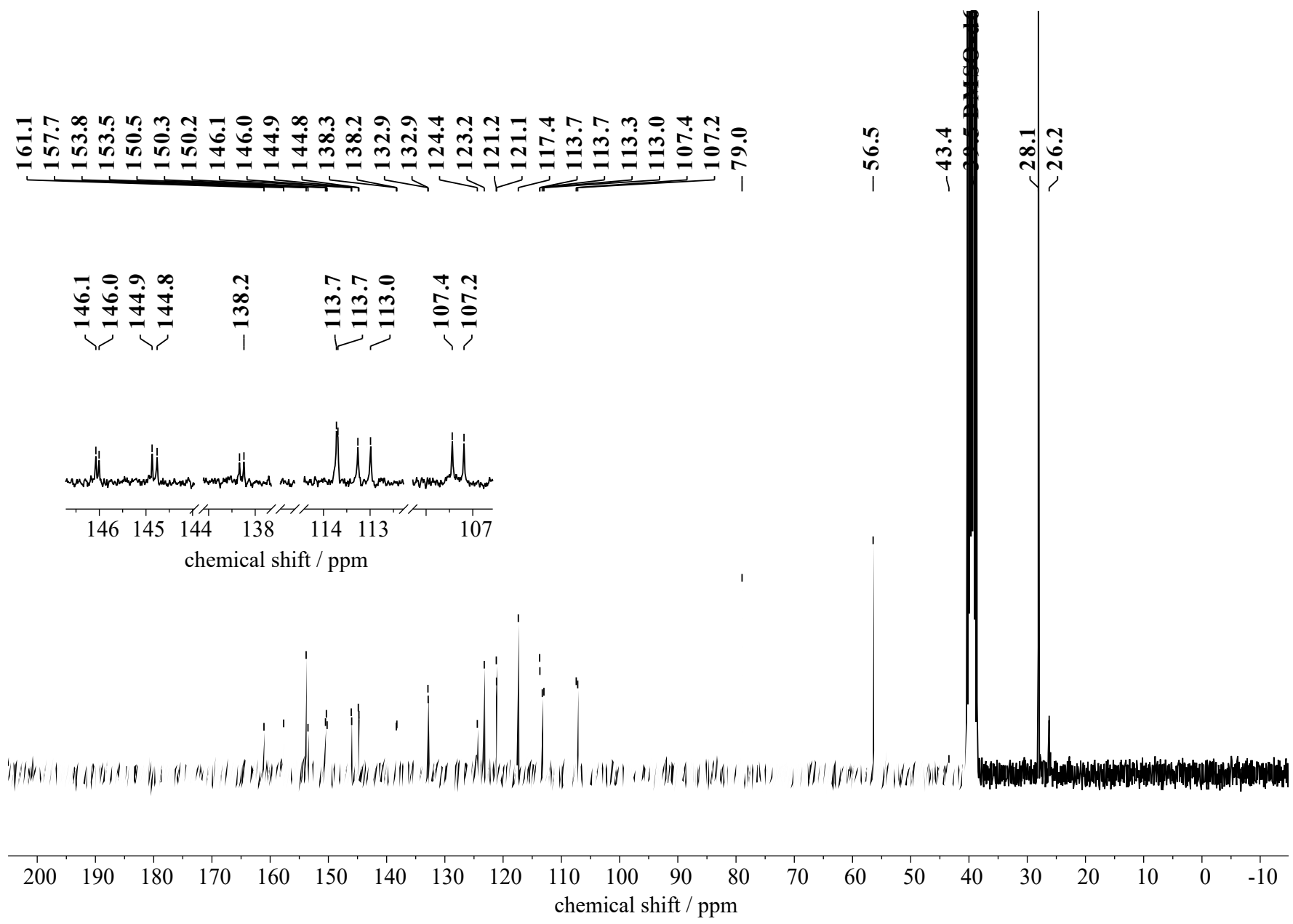


**Figure S87**. $^{1}H$ (300 MHz) and $^{13}C$ (75 MHz) NMR spectra of **43** in DMSO-$d_6$.

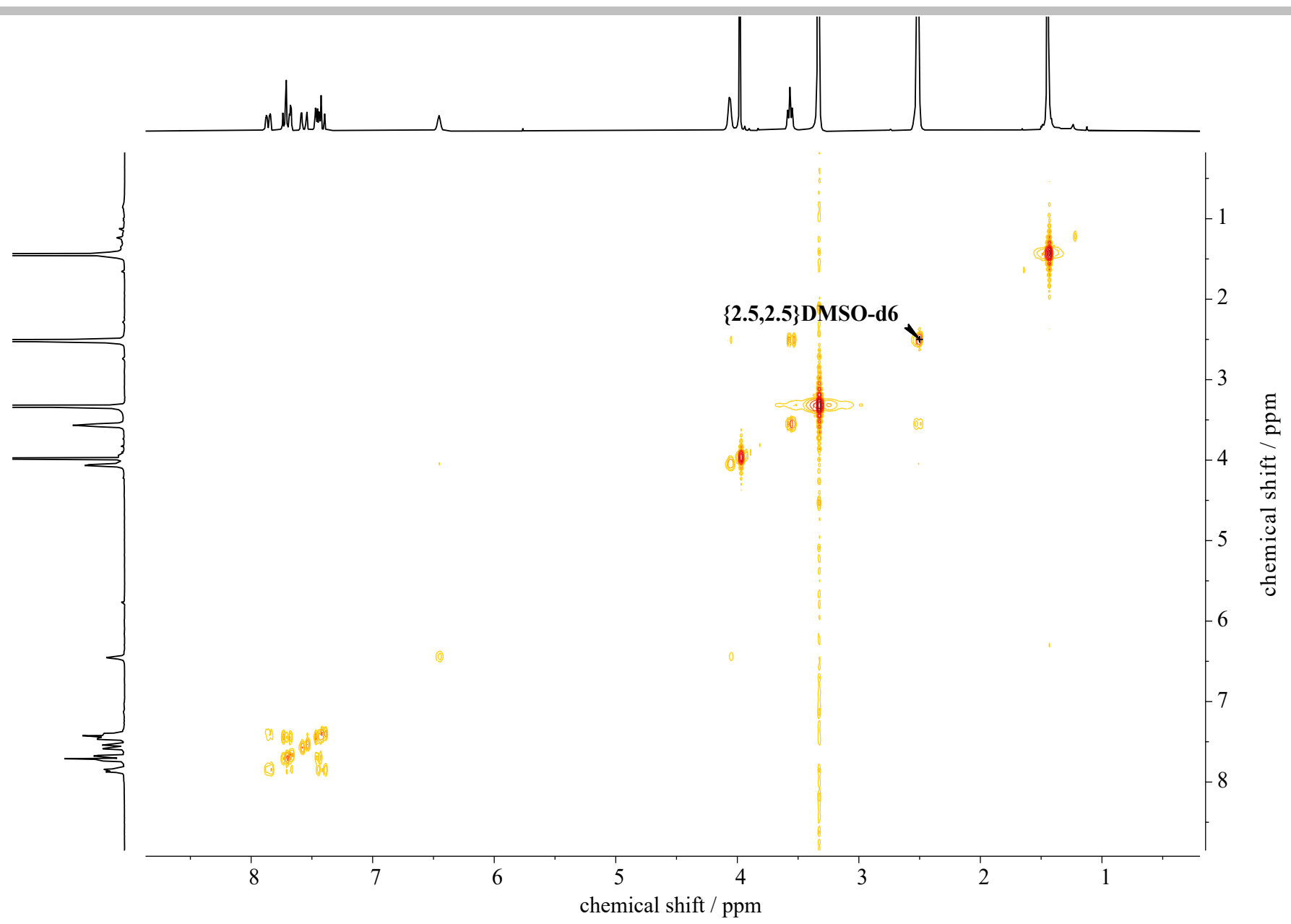


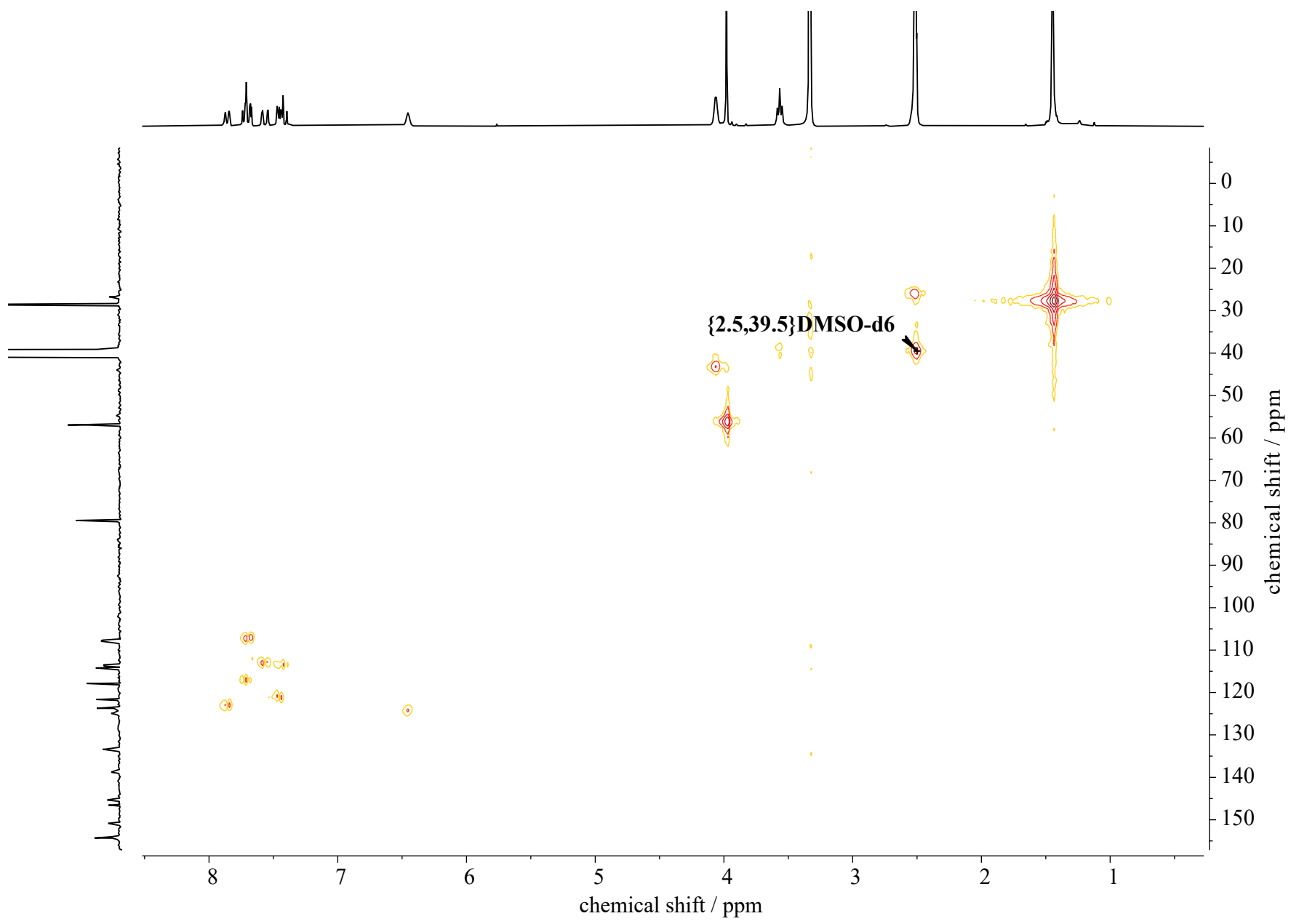


**Figure S88**. COSY and HMQC NMR spectra of **43** in DMSO-$d_6$.

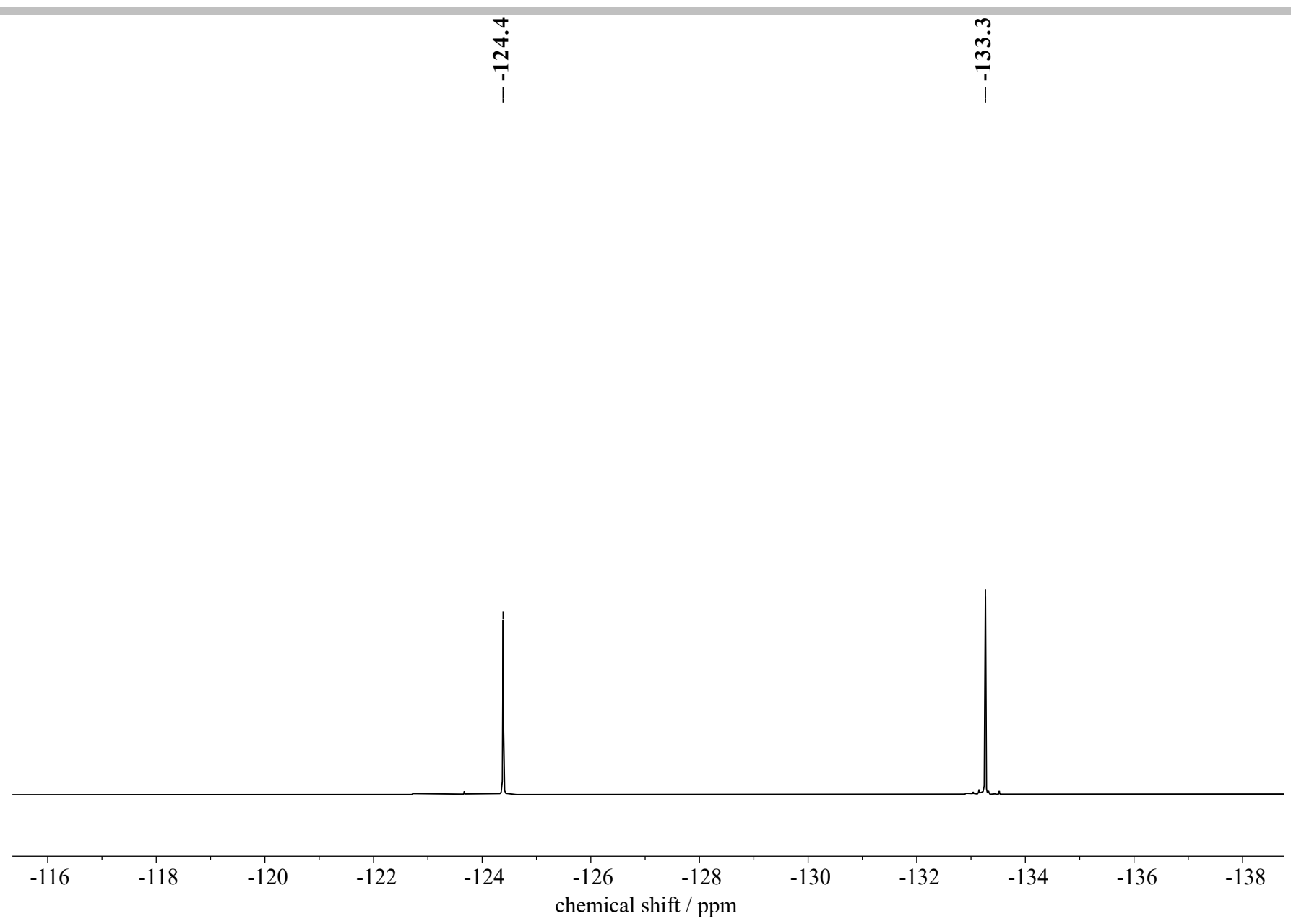


**Figure S89**. $^{19}$F (282 MHz) NMR spectra of **43** in DMSO-$d_6$.

**Synthesis of 4-(3-fluoro-4-((3-fluoro-4-methoxyphenyl)diazenyl)phenyl)-1,2,3,6-tetrahydropyridin (12)**

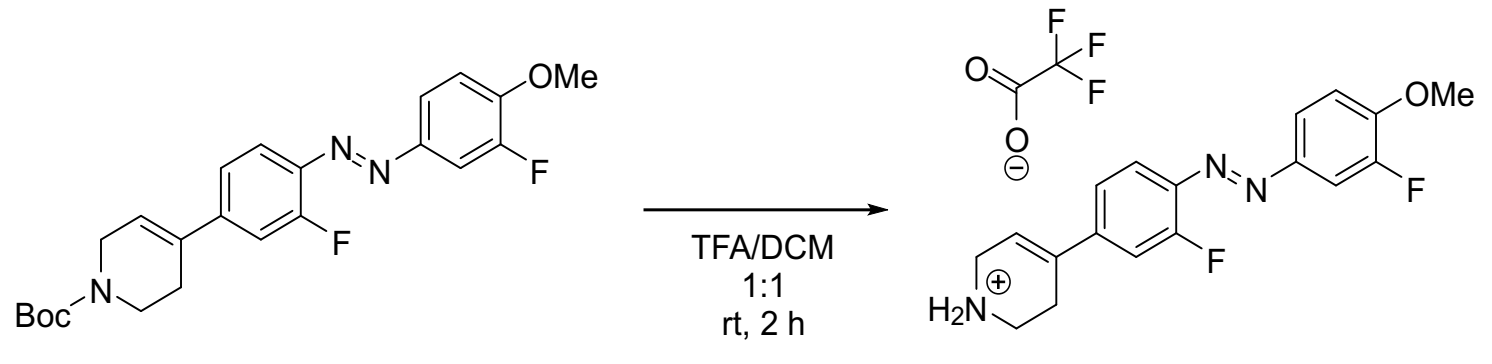


43 → 12

*Tert*-butyl-4-(3-fluoro-4-((3-fluoro-4-methoxyphenyl)diazenyl)phenyl)-3,6-dihydropyridine-1(*2H*)-carboxylate **43** (50 mg, 117 µmol, 1.00 eq) was dissolved in mixture of TFA/DCM (12 mL, 1:5, v%). The reaction was stirred at room temperature for 2 h. The reaction was then diluted with 100 mL distilled water and extracted with 100 mL EtOAc. The organic layer was washed with 100 mL distilled water and brine, dried over $MgSO_4$ and solvent was removed under reduced pressure. The crude product was purified by flash column chromatography (EtOAc/MeOH, 1:1, v%) to yield the desired compound 4-(3-fluoro-4-((3-fluoro-4-methoxyphenyl)diazenyl)phenyl)-1,2,3,6-tetrahydropyridin (**12**, 40 mg, 90.9 µmol, 78%) as an orange solid in form of a TFA salt.

**TLC:** $R_f$ = 0.22 (EtOAc/MeOH, 1:1, v%).

***trans*-isomer** (*cis*-isomer signals were labeled with * on NMR spectrum).

**$^1$H-NMR (300 MHz, DMSO-*d*$_6$, *δ*):** 8.95 (s, 2H, N$H_2$), 7.88-7.84 (m, 1H, $H_{arom.}$), 7.76-7.61 (m, 3H, $H_{arom.}$, $H_{arom.}$, $H_{arom.}$), 7.49 (dd, $^3J$ = 8.6 Hz, $^4J$ = 1.9 Hz, 1H, $H_{arom.}$), 7.42 (t, $^3J$ = 8.9 Hz, 1H, $H_{arom.}$), 6.49-6.47 (m, 1H, C*H*=C), 3.97 (s, 3H, C$H_3$), 3.83-3.82 (m, 2H, C$H_2$), 3.38-3.86 (m, 2H, C$H_2$), 2.75-2.71 (m, 2H, C$H_2$) ppm.

**$^{13}$C-NMR (75 MHz, DMSO-*d*$_6$, *δ*):** 159.3 (d, $^1J_{FC}$ = 255.1 Hz, *C*F), 153.5 ($C_{arom.}$), 153.5 (d, $^1J_{FC}$ = 227.8 Hz, *C*F), 150.6 ($C_{arom.}$), 146.1 (d, $^3J_{FC}$ = 5.4 Hz, $C_{arom.}$), 143.4 (d, $^3J_{FC}$ = 7.7 Hz, $C_{arom.}$), 138.8 ($C_{arom.}$), 132.7 ($C_{arom.}$), 123.4 (d, $^3J_{FC}$ = 2.7 Hz, $C_{arom.}$H), 121.3 (d, $^3J_{FC}$ = 3.1 Hz, $C_{arom.}$H), 120.1 (*C*H=C), 117.7 ($C_{arom.}$H), 113.7 (d, $^3J_{FC}$ = 2.2 Hz, $C_{arom.}$H), 113.4 (d, $^2J_{FC}$ = 20.9 Hz, $C_{arom.}$H), 107.3 (d, $^2J_{FC}$ = 19.0 Hz, $C_{arom.}$H), 56.5 (*C*$H_3$), 42.8 (*C*$H_2$), 41.7 (*C*$H_2$), 22.9 (*C*$H_2$) ppm.

**$^{19}$F-NMR (282 MHz, DMSO-*d*$_6$, *δ*):** -124.2 (C*F*), -133.2 (C*F*) ppm.

**HRMS-ESI (m/z):** $[M+H]^+$ calcd for $C_{18}H_{18}F_2N_3O$, 330.1418; found, 330.1412.

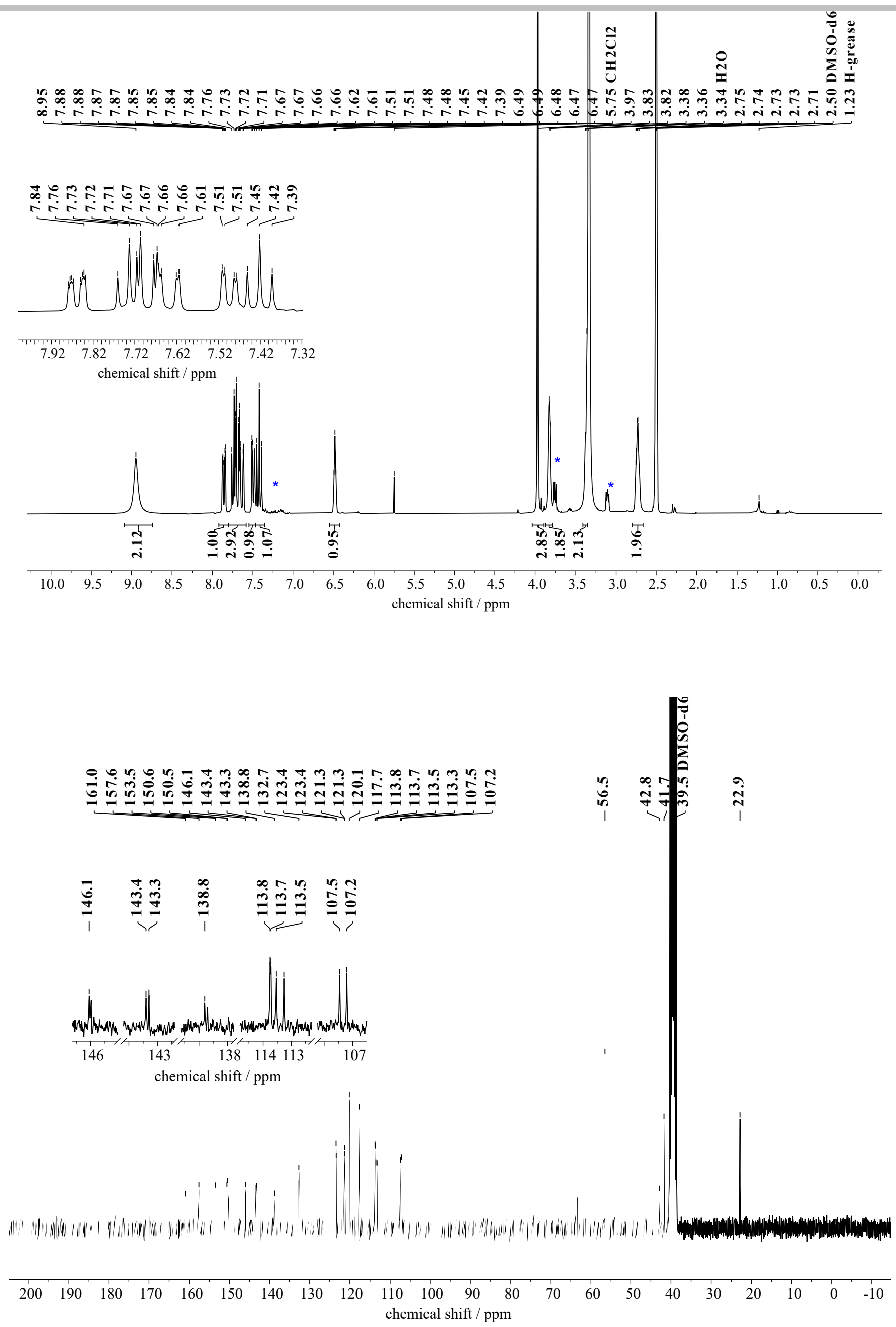

**Figure S90**. $^{1}H$ (300 MHz) and $^{13}C$ (75 MHz) NMR spectra of **12** in DMSO-$d_6$.

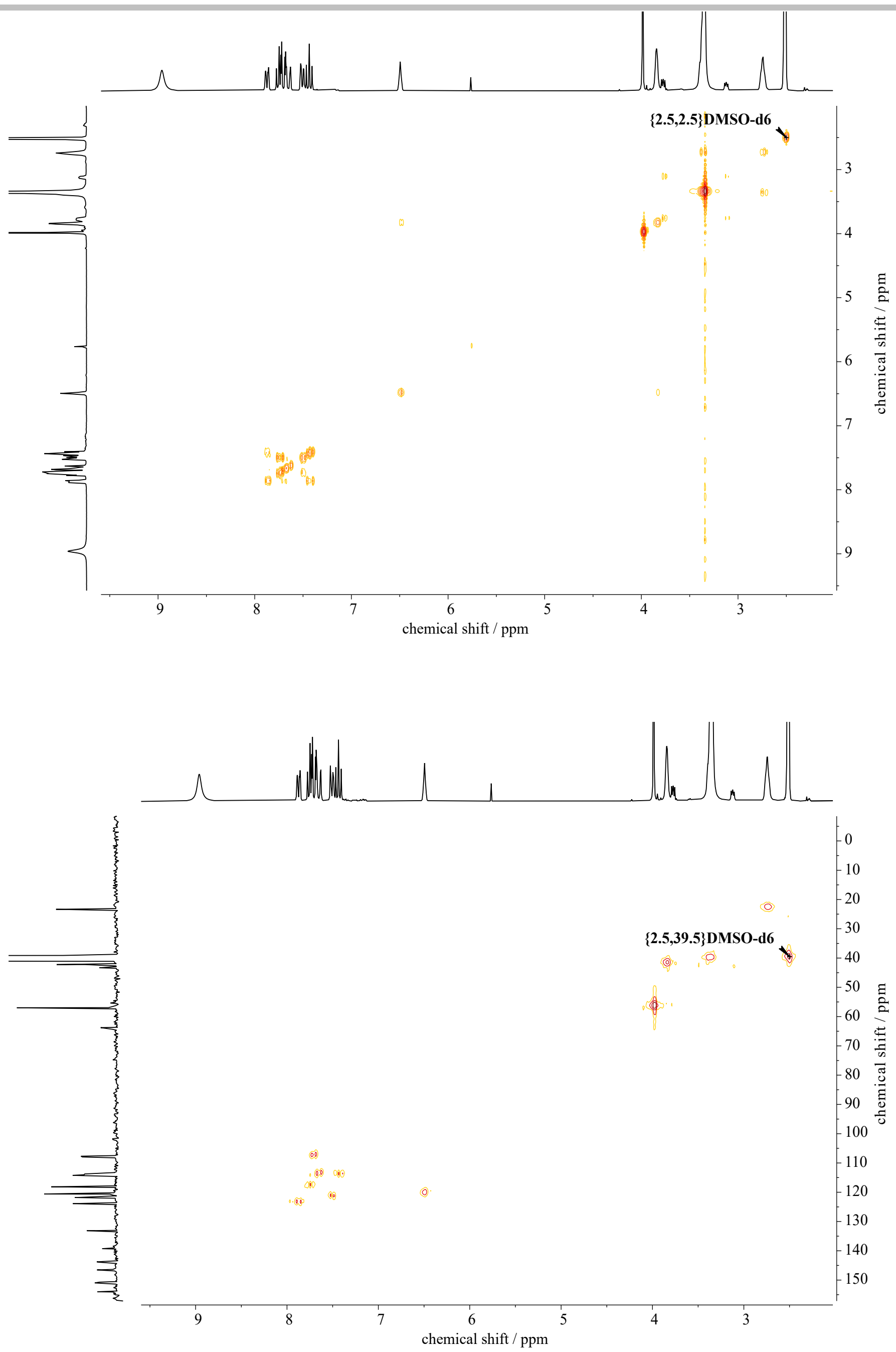


**Figure S91**. COSY and HMQC NMR spectra of **12** in DMSO-$d_6$.

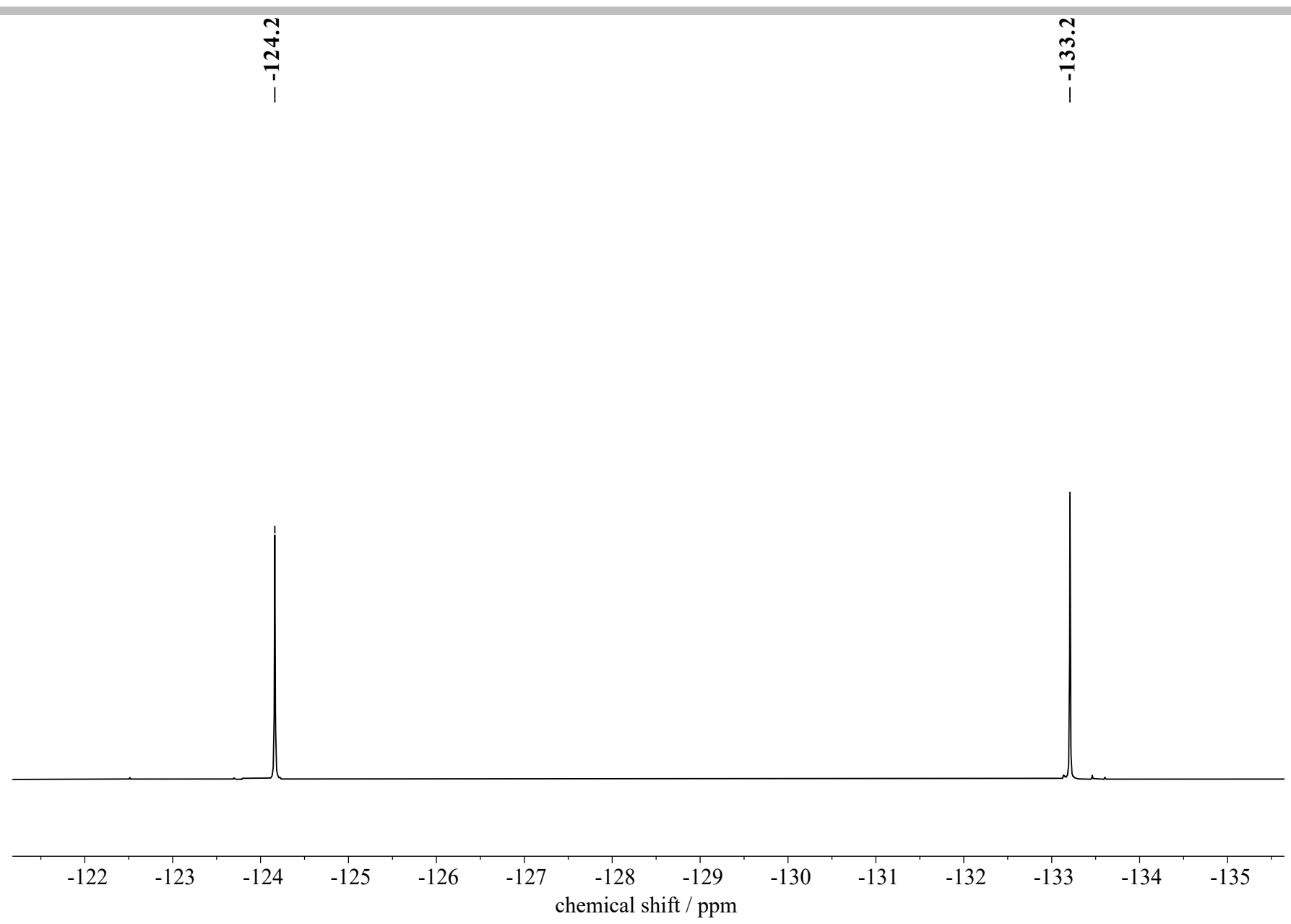


**Figure S92**. $^{19}F$ (282 MHz) NMR spectra of **12** in DMSO-$d_6$.

## Results and Discussion

### Characterization

#### *Molar Extinction Coefficient*

UV-vis spectra were obtained with samples of corresponding compound prepared in phosphate buffer (10 mM $NaH_2PO_4$, 150 mM NaCl, pH 7.4) with 5% bio-grade DMSO with a final concentration of 50 µM.
The extinction coefficient was determined in bio-grade DMSO at isosbestic wavelength of corresponding compound **2-12** following the general procedure explain in section 'Ultraviolet-visible Spectroscopy (UV-vis)'. Data analysis was performed by linear regression with software Graphpad Prism 8.0. Determination was repeated three times independently, i.e., from three different stock solutions. For all values, the corresponding background signal was subtracted.

**Table S1**: Overlaid UV-vis spectra of both isomers of corresponding compounds **2-12** in phosphate buffer (10 mM $NaH_2PO_4$, 150 mM NaCl, pH 7.4) with a final concentration of 50 µM, green: without irradiation, purple: for compound **2-8**, **10-12** after irradiation at 365 nm for 1 min; for compound **9**, 6 min. And determination of the molar extinction coefficients. The determination was repeated three times independently i.e., from three different stock solutions. For all values, the corresponding background signal was subtracted.

| Compound | UV-vis spectra | Extinction Coefficient |
|---|---|---|
| 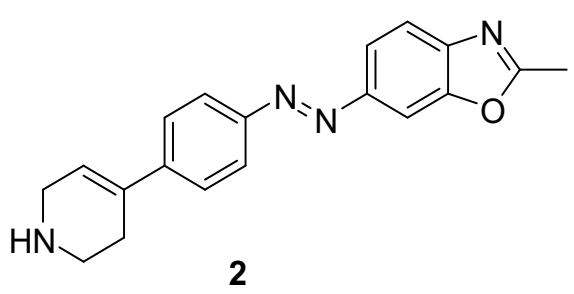 **2** | 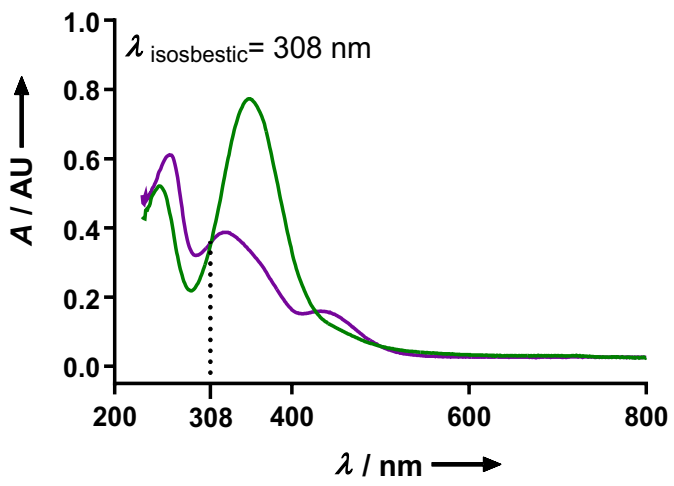  | 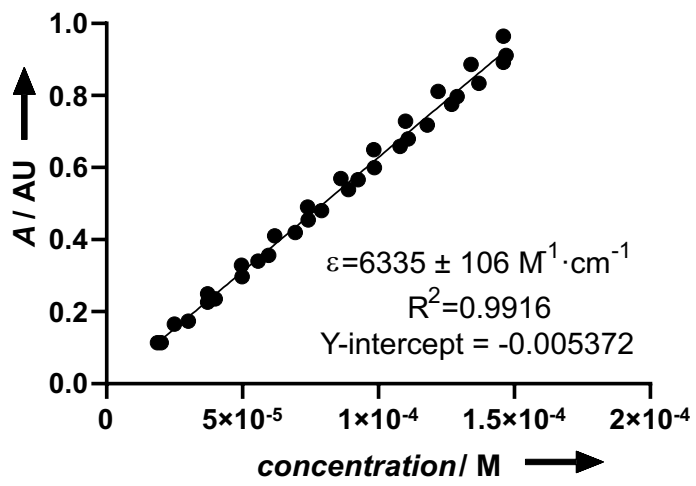  |
| 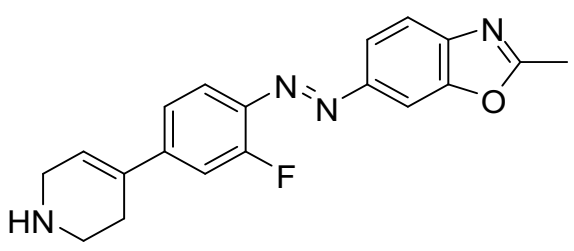 **3** | 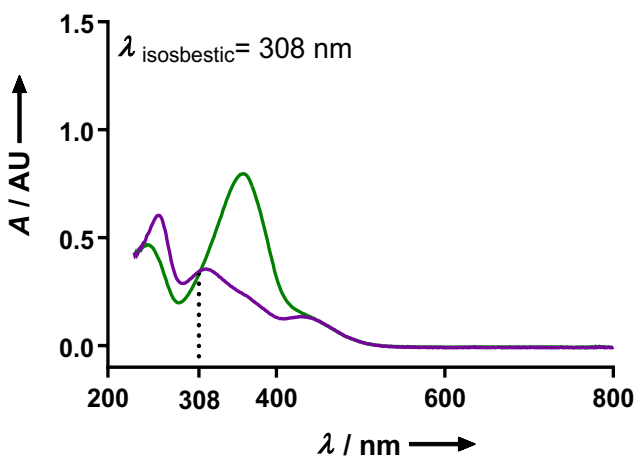  | 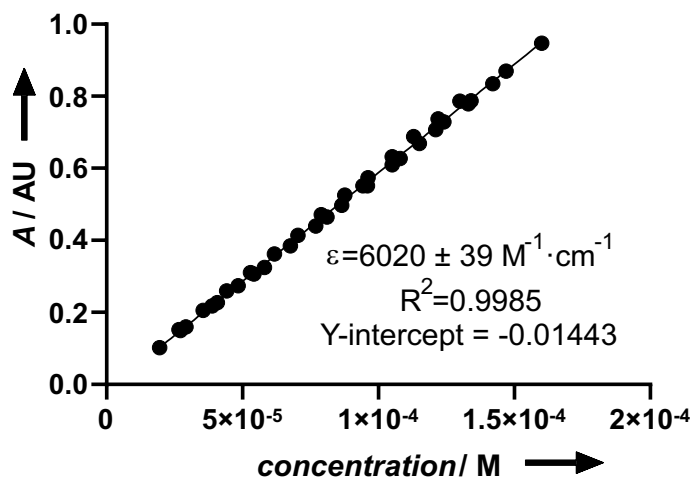  |
| 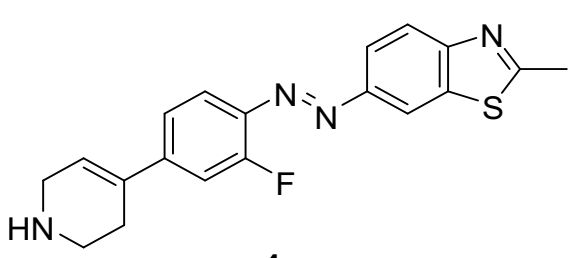 **4** | 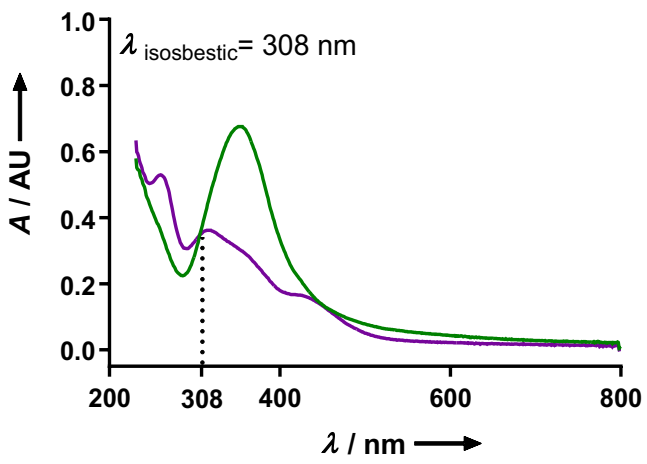  | 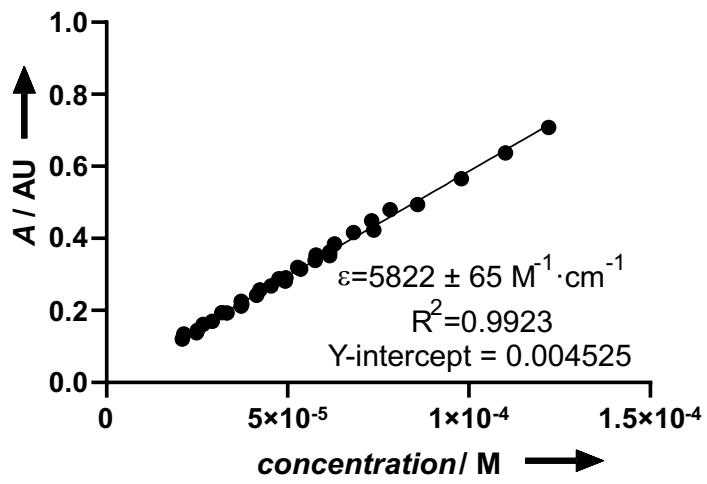  |

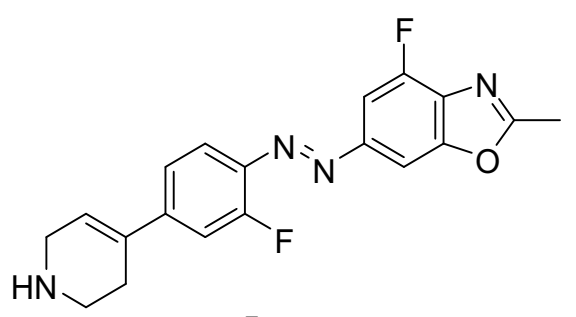
F
N
O
N
N
F
HN

5

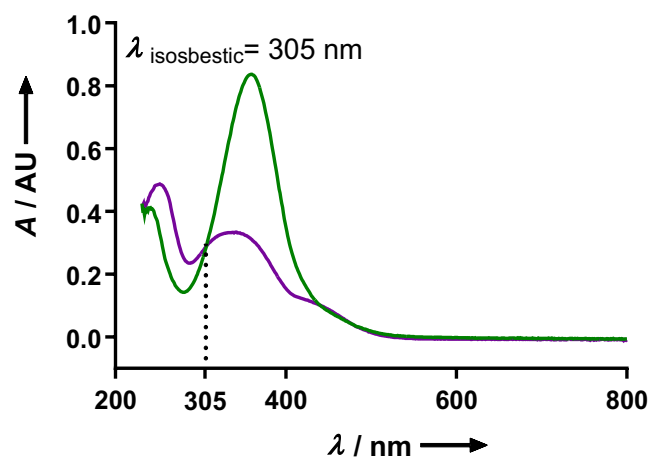
λ isosbestic= 305 nm
A / AU
λ / nm

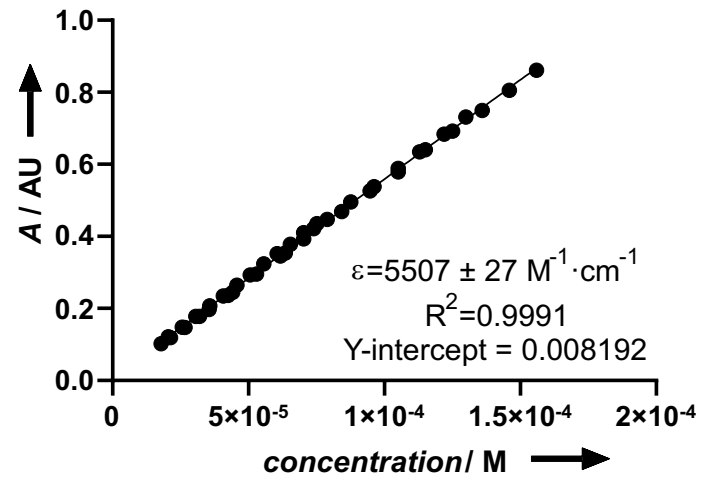
A / AU
ε=5507 ± 27 M$^{-1}$·cm$^{-1}$
$R^2$=0.9991
Y-intercept = 0.008192
concentration/ M

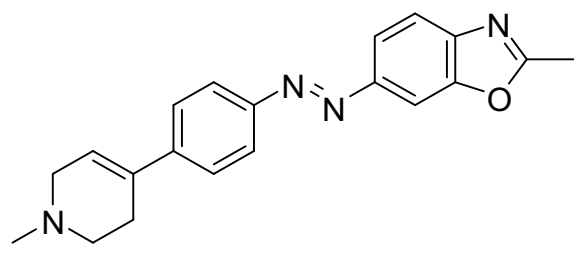
N
O
N
N
N

6

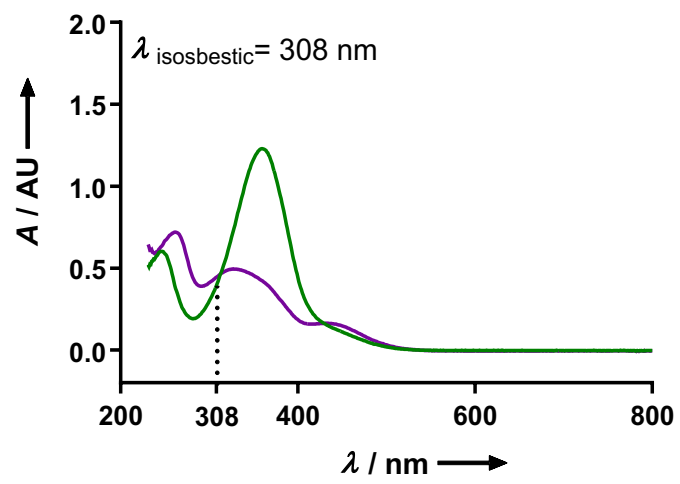
λ isosbestic= 308 nm
A / AU
λ / nm

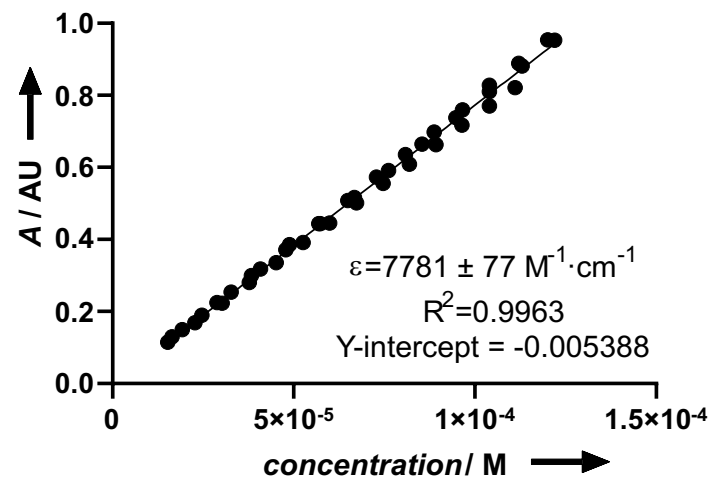
A / AU
ε=7781 ± 77 M$^{-1}$·cm$^{-1}$
$R^2$=0.9963
Y-intercept = -0.005388
concentration/ M

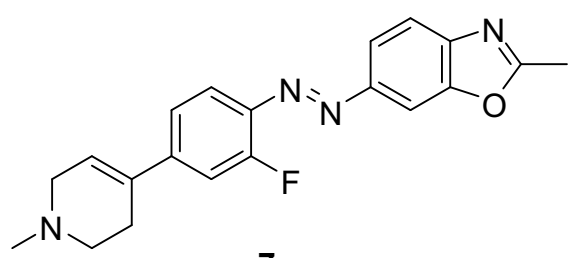
N
O
N
N
F
N

7

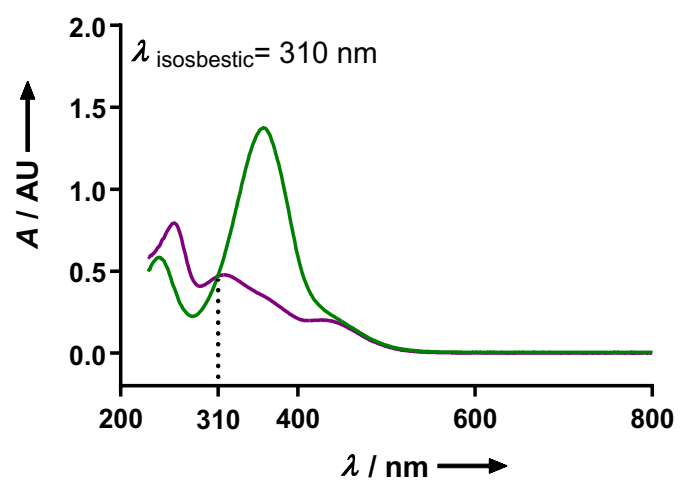
λ isosbestic= 310 nm
A / AU
λ / nm

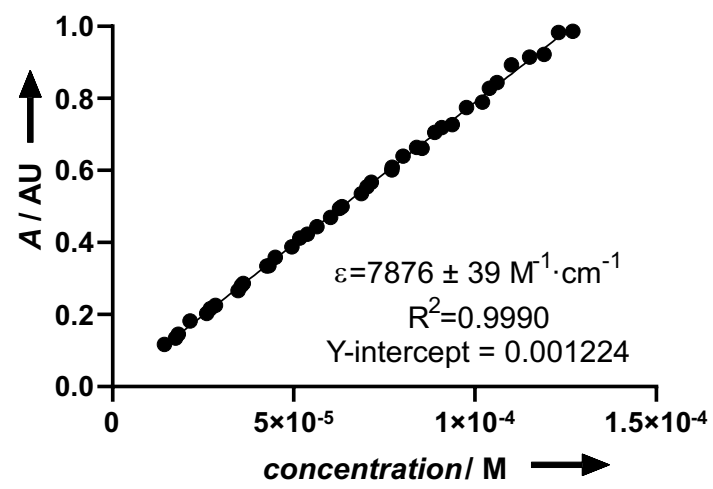
A / AU
ε=7876 ± 39 M$^{-1}$·cm$^{-1}$
$R^2$=0.9990
Y-intercept = 0.001224
concentration/ M

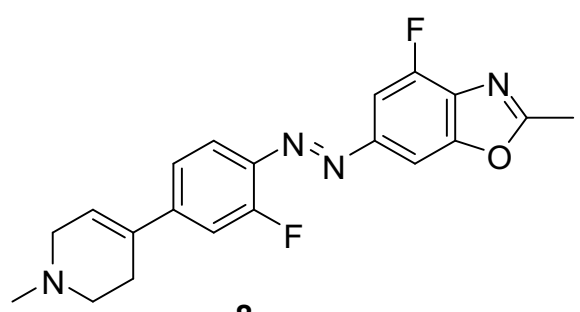
F
N
O
N
N
F
N

8

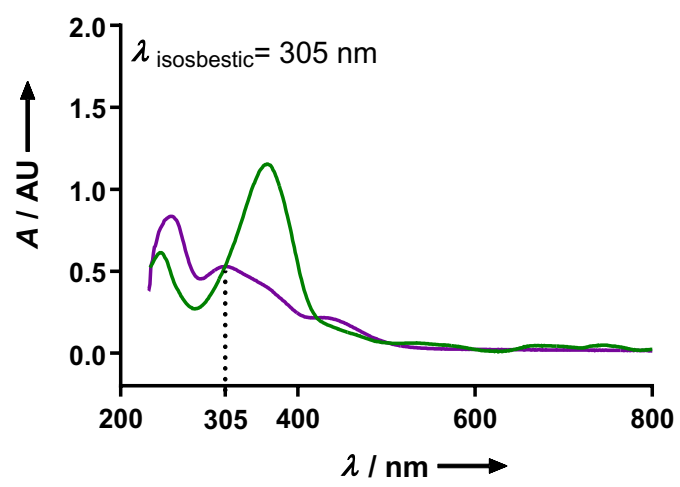
λ isosbestic= 305 nm
A / AU
λ / nm

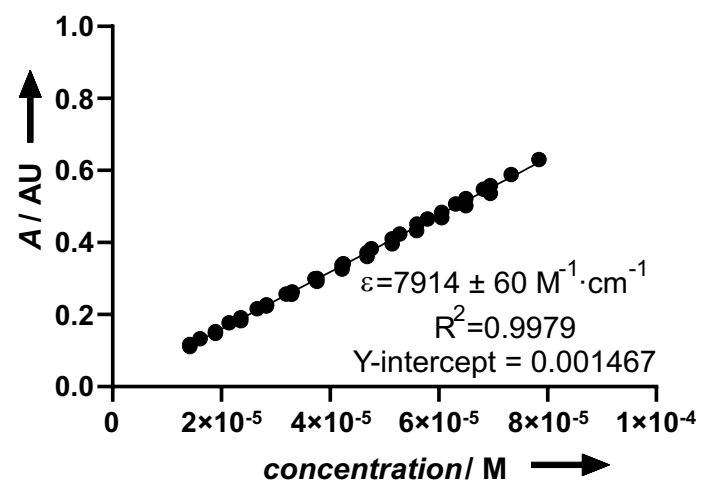
A / AU
ε=7914 ± 60 M$^{-1}$·cm$^{-1}$
$R^2$=0.9979
Y-intercept = 0.001467
concentration/ M

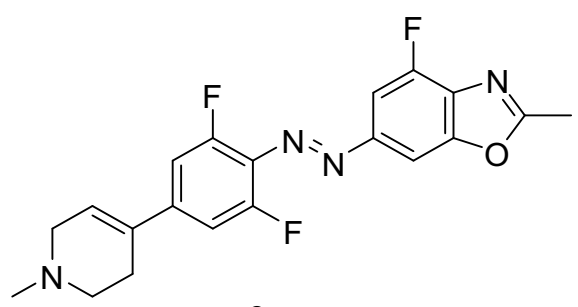
F
F
N
O
N
N
F
N

9

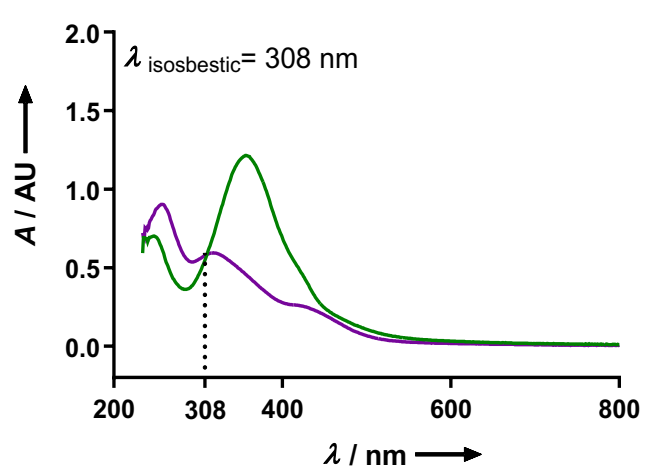
λ isosbestic= 308 nm
A / AU
λ / nm

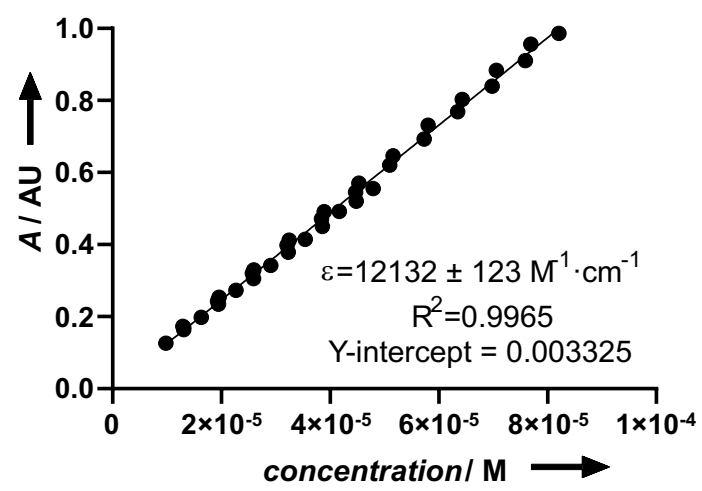
A / AU
ε=12132 ± 123 M$^{-1}$·cm$^{-1}$
$R^2$=0.9965
Y-intercept = 0.003325
concentration/ M

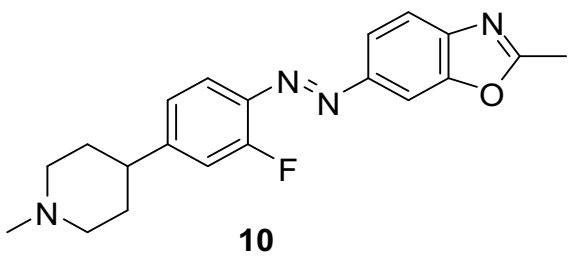

**10**

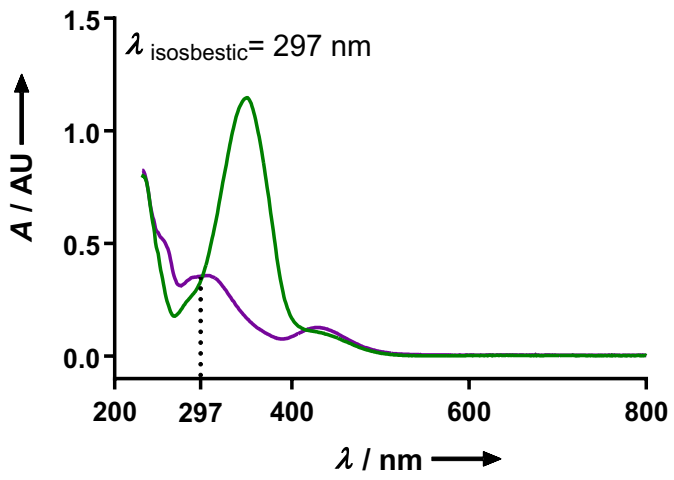


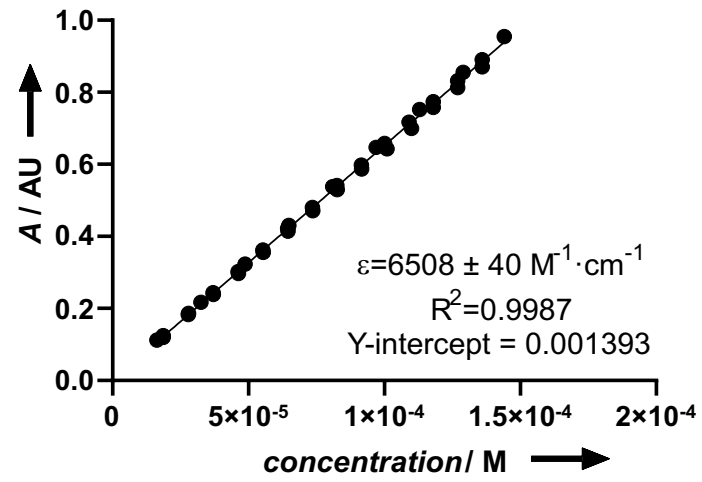


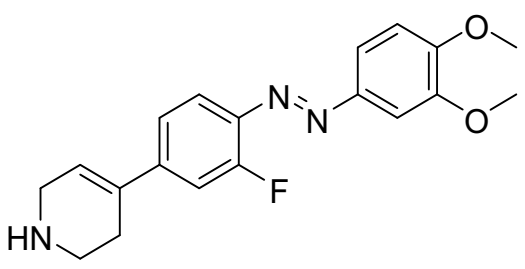

**11**

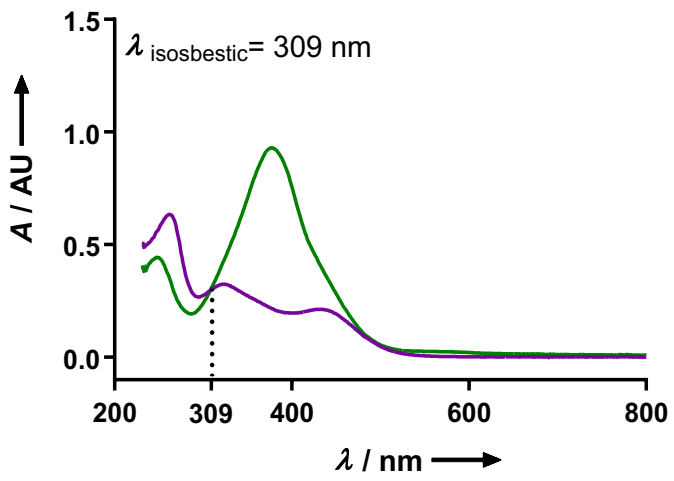


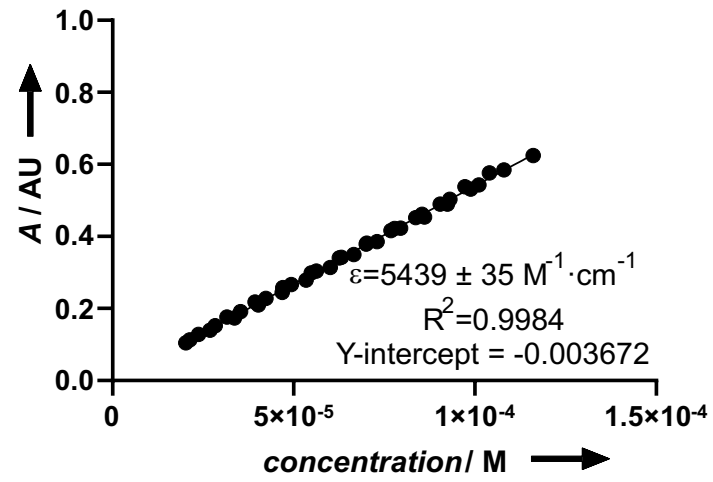


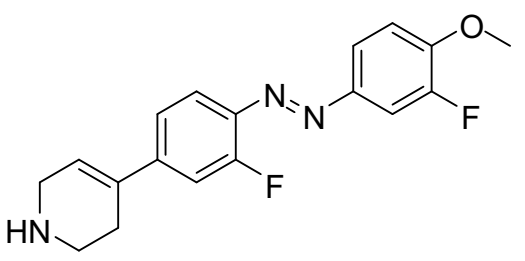

**12**

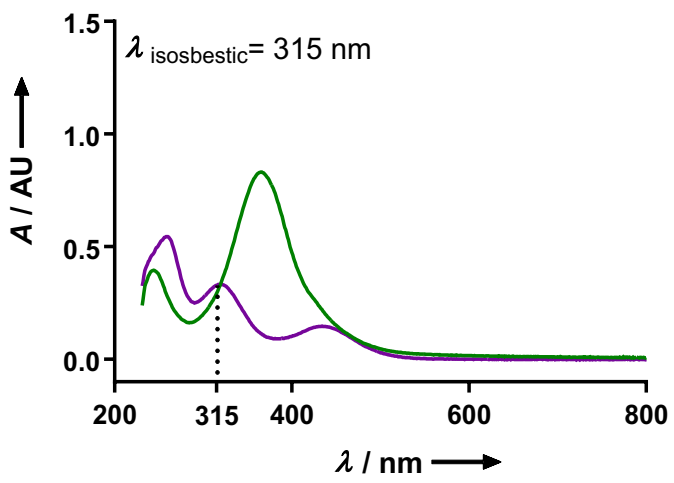


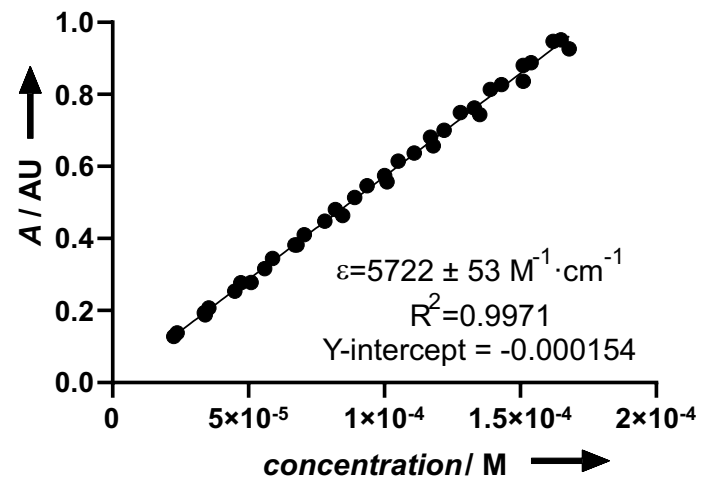


## *Isomerization*

**Table S2:** UV–vis spectra of 50 μM solution of corresponding compound **2-8**, **10-12** in PBS buffer (140 mM NaCl, 10 mM $Na_2HPO_4$, 2.7 mM KCl, 1.8 mM $KH_2PO_4$, pH 7.4) with 5% bio-grade DMSO. A) Freshly prepared sample followed by irradiation at 365 nm at different time intervals: 10 s / 20 s / 30 s / 40 s / 50 s / 60 s / 90 s, for compound **9** 10 s / 20 s / 30 s / 40 s / 50 s / 60 s / 90 s / 120 s / 150 s / 180 s / 240 s / 300 s / 360 s. B) Final irradiation conditions of A), followed by irradiation at 520 nm at the time intervals: 5 s / 10 s / 20 s / 30 s. The plots resulted from two independent measurements, i.e., from different stock solutions.

| Compound | A) | B) |
|---|---|---|
| **2** | 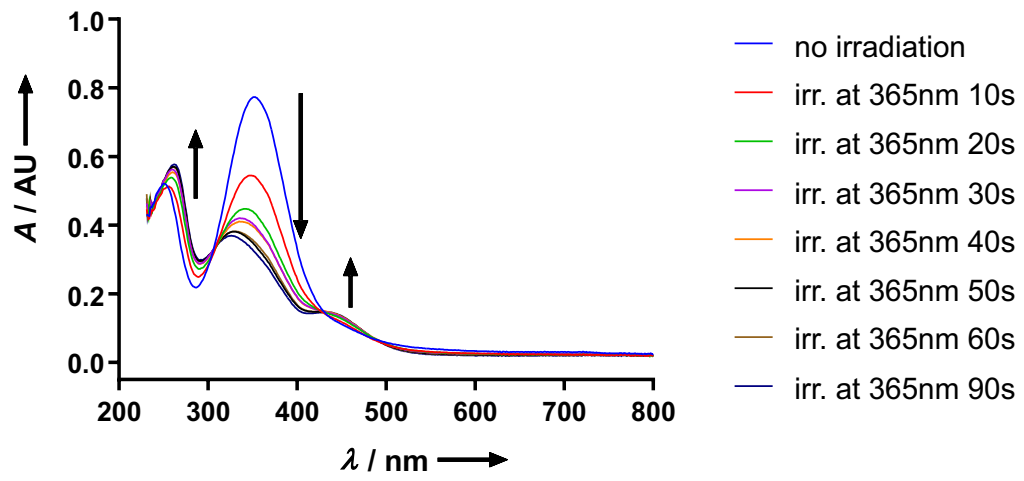  | 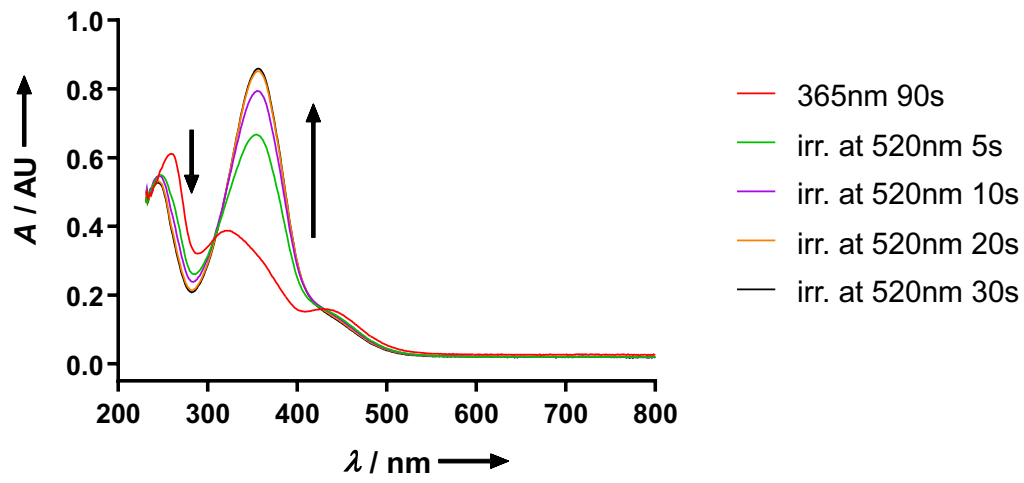  |
| **3** | 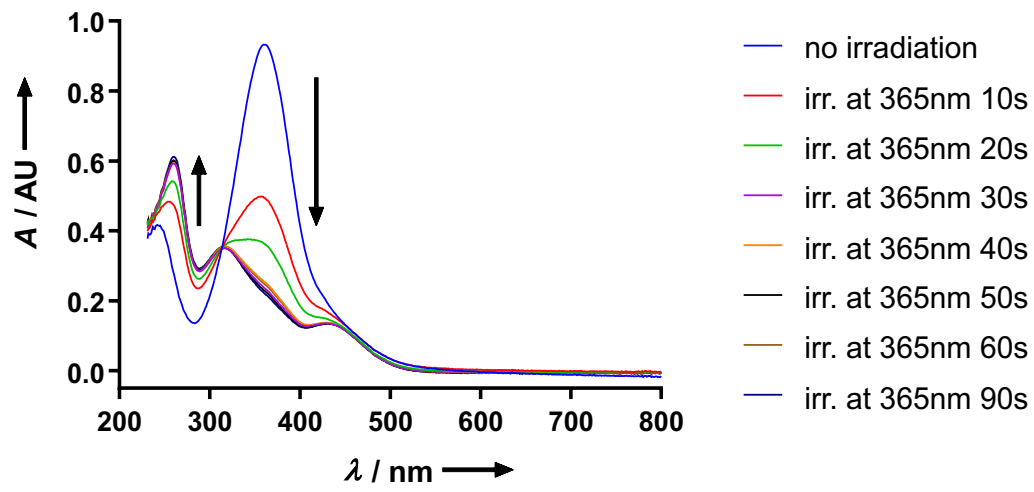  | 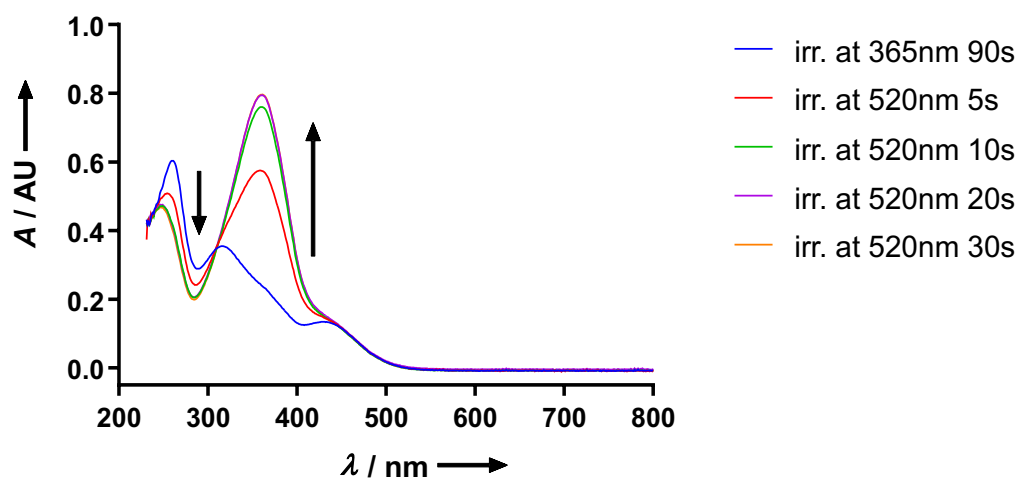  |

4

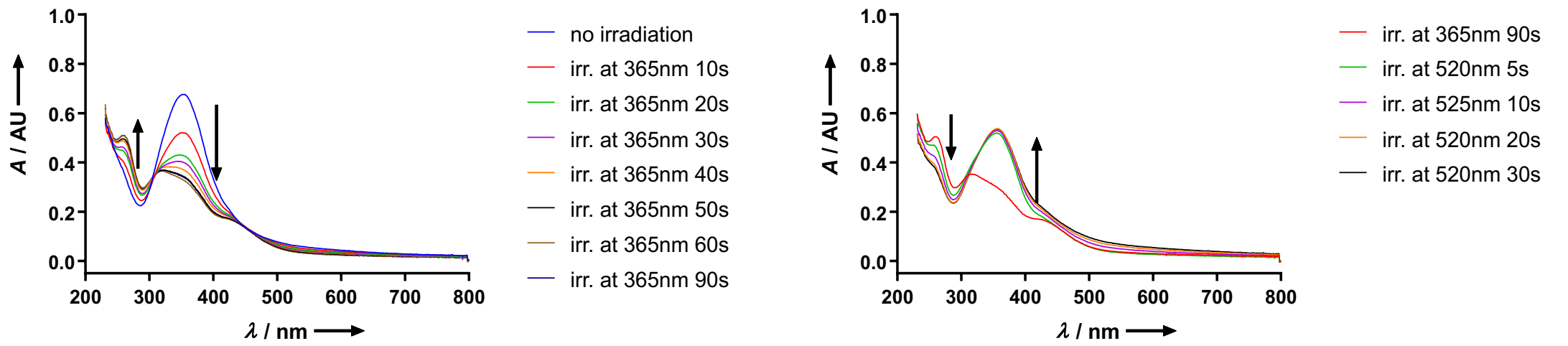
A / AU
λ / nm
no irradiation
irr. at 365nm 10s
irr. at 365nm 20s
irr. at 365nm 30s
irr. at 365nm 40s
irr. at 365nm 50s
irr. at 365nm 60s
irr. at 365nm 90s
A / AU
λ / nm
irr. at 365nm 90s
irr. at 520nm 5s
irr. at 525nm 10s
irr. at 520nm 20s
irr. at 520nm 30s

5

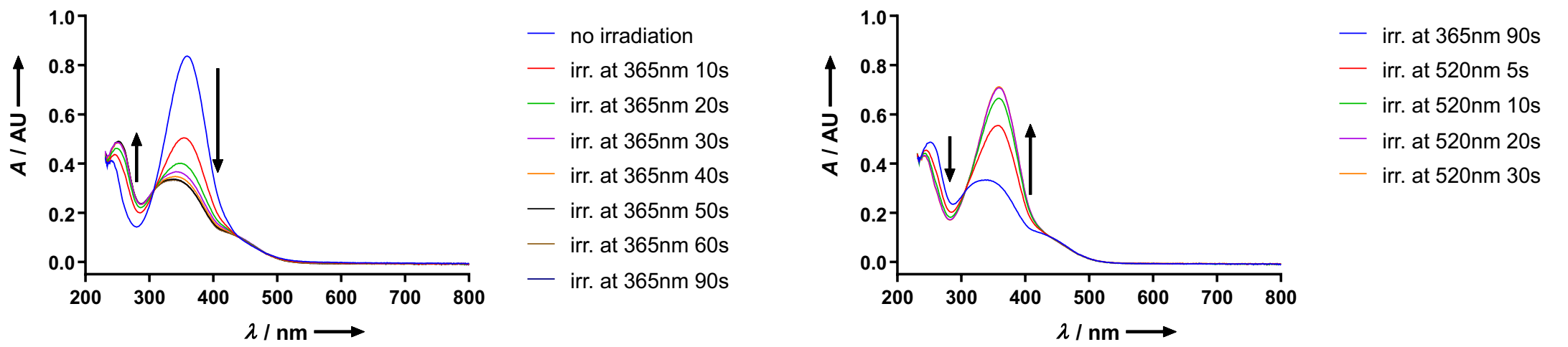
A / AU
λ / nm
no irradiation
irr. at 365nm 10s
irr. at 365nm 20s
irr. at 365nm 30s
irr. at 365nm 40s
irr. at 365nm 50s
irr. at 365nm 60s
irr. at 365nm 90s
A / AU
λ / nm
irr. at 365nm 90s
irr. at 520nm 5s
irr. at 520nm 10s
irr. at 520nm 20s
irr. at 520nm 30s

6

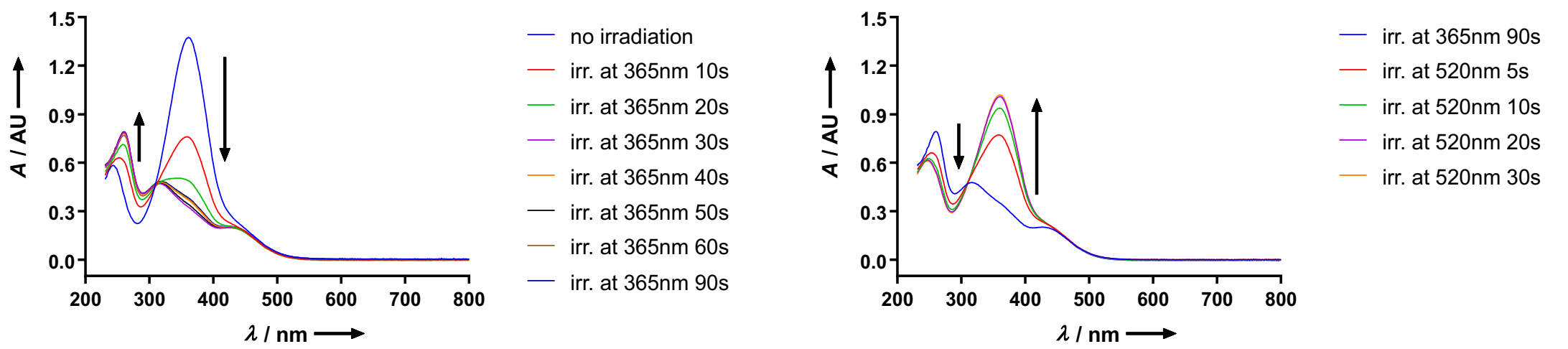
A / AU
λ / nm
no irradiation
irr. at 365nm 10s
irr. at 365nm 20s
irr. at 365nm 30s
irr. at 365nm 40s
irr. at 365nm 50s
irr. at 365nm 60s
irr. at 365nm 90s
A / AU
λ / nm
irr. at 365nm 90s
irr. at 520nm 5s
irr. at 520nm 10s
irr. at 520nm 20s
irr. at 520nm 30s

7

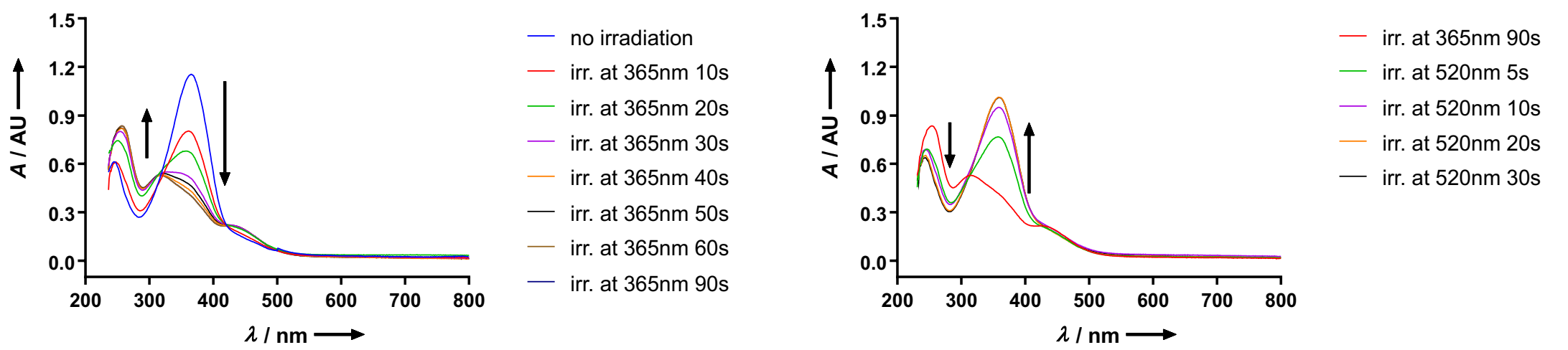
A / AU
λ / nm
no irradiation
irr. at 365nm 10s
irr. at 365nm 20s
irr. at 365nm 30s
irr. at 365nm 40s
irr. at 365nm 50s
irr. at 365nm 60s
irr. at 365nm 90s
A / AU
λ / nm
irr. at 365nm 90s
irr. at 520nm 5s
irr. at 520nm 10s
irr. at 520nm 20s
irr. at 520nm 30s

8

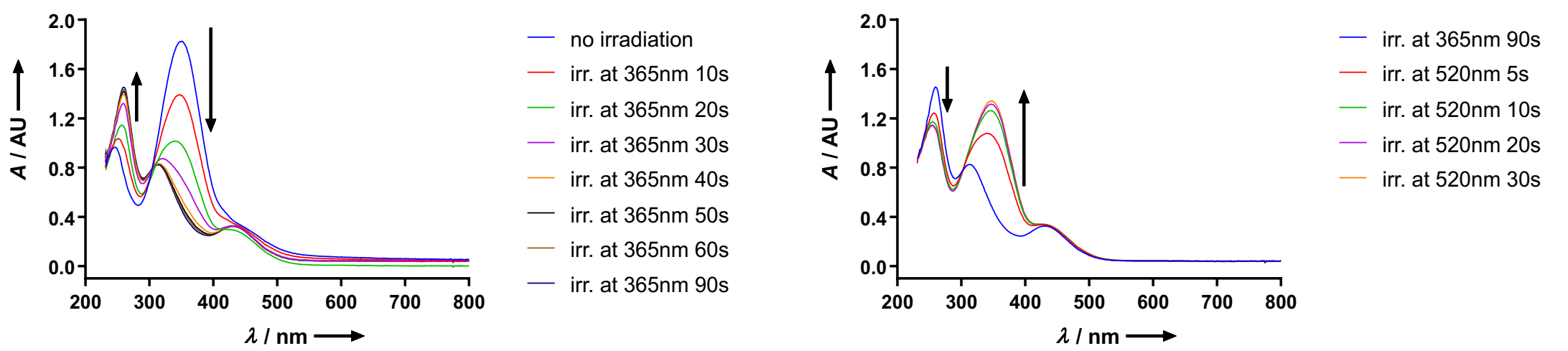
A / AU
λ / nm
no irradiation
irr. at 365nm 10s
irr. at 365nm 20s
irr. at 365nm 30s
irr. at 365nm 40s
irr. at 365nm 50s
irr. at 365nm 60s
irr. at 365nm 90s
A / AU
λ / nm
irr. at 365nm 90s
irr. at 520nm 5s
irr. at 520nm 10s
irr. at 520nm 20s
irr. at 520nm 30s

9

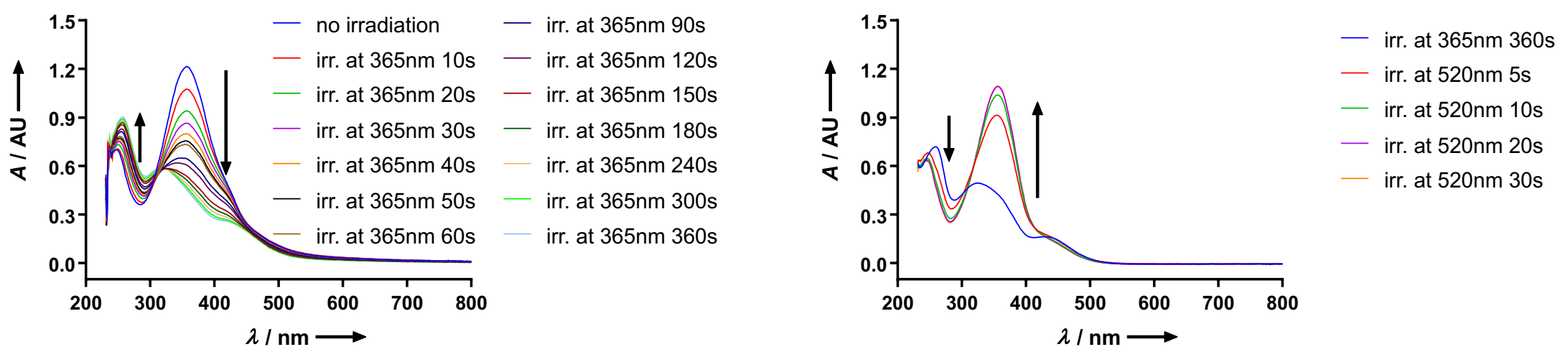
A / AU
λ / nm
no irradiation
irr. at 365nm 10s
irr. at 365nm 20s
irr. at 365nm 30s
irr. at 365nm 40s
irr. at 365nm 50s
irr. at 365nm 60s
irr. at 365nm 90s
irr. at 365nm 120s
irr. at 365nm 150s
irr. at 365nm 180s
irr. at 365nm 240s
irr. at 365nm 300s
irr. at 365nm 360s
A / AU
λ / nm
irr. at 365nm 360s
irr. at 520nm 5s
irr. at 520nm 10s
irr. at 520nm 20s
irr. at 520nm 30s

| | | |
|---|---|---|
| **10** | 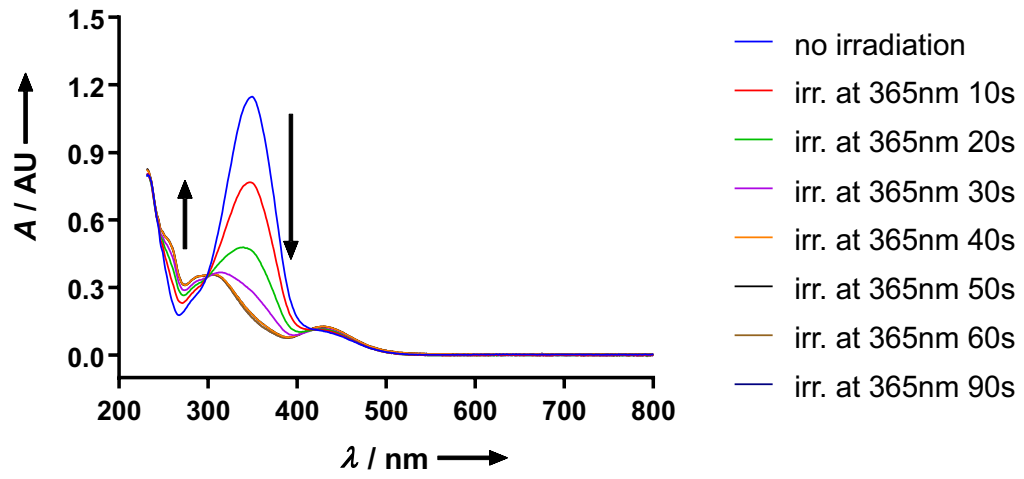 | 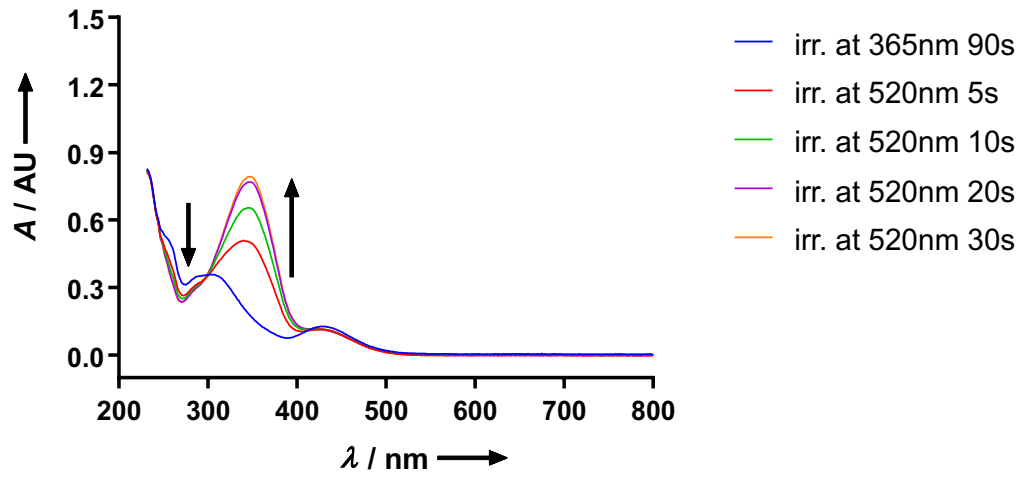 |
| **11** | 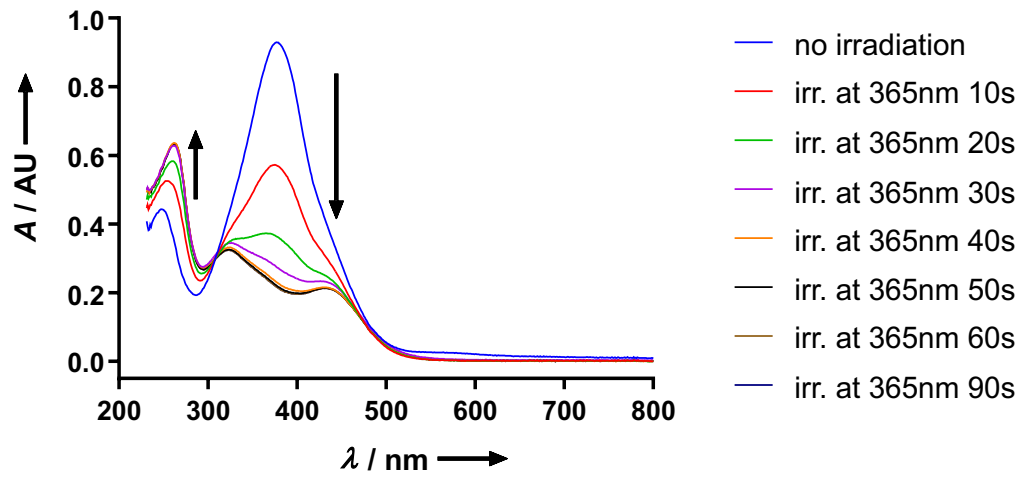 | 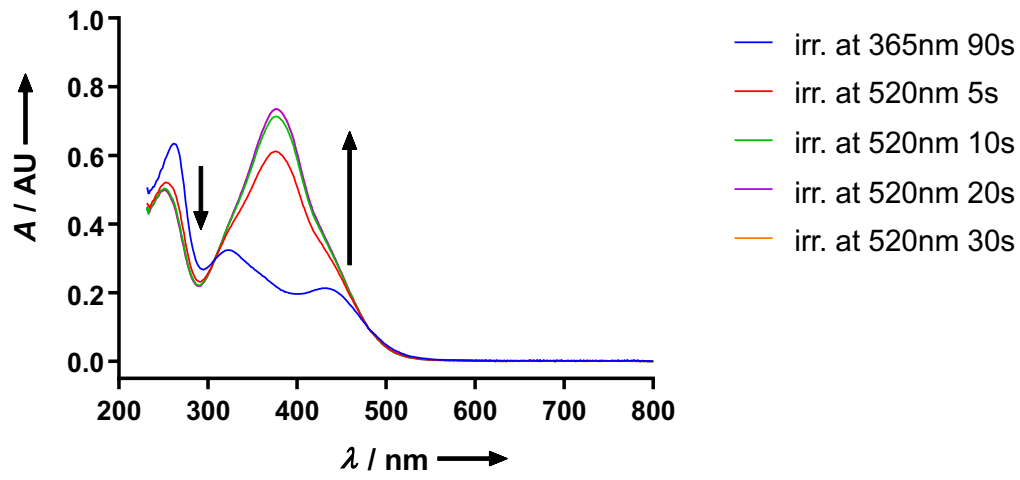 |
| **12** | 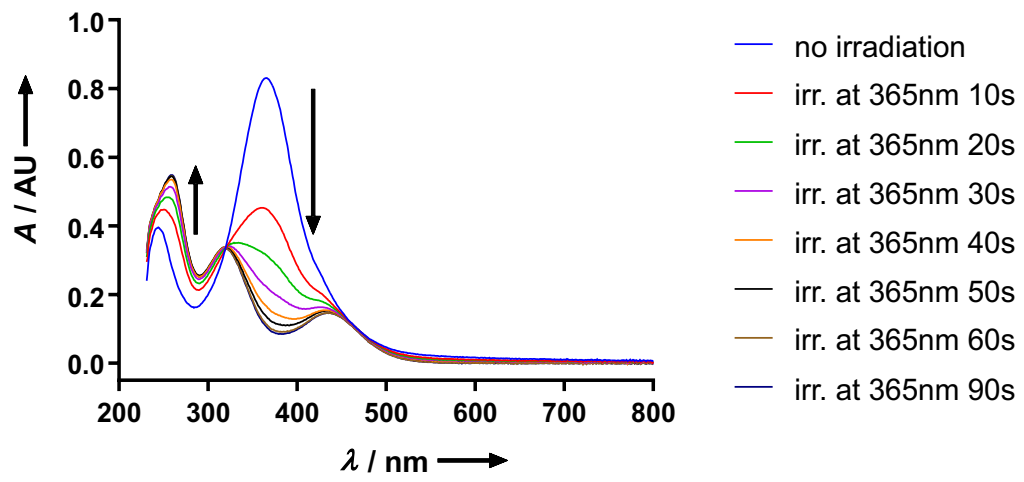 | 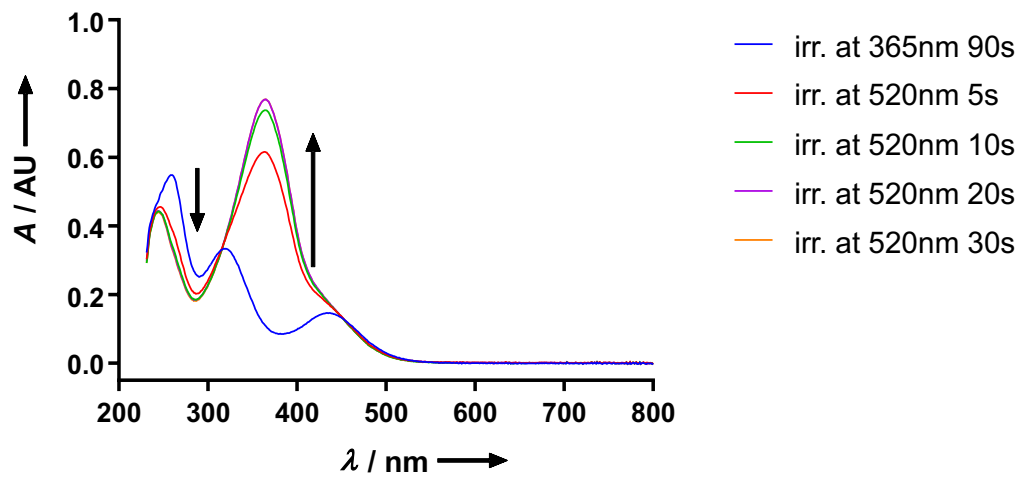 |

*Reversibility*

The reversibility of the isomerization of compounds **2-12** was verified for 20 cycles. Samples did not display any photodegradation or photochemical fatigue. The plots resulted from two independent measurements, i.e., from different stock solutions.

**Table S3:** Reversible photochromism of a 50 µM solution of corresponding compound in PBS buffer (140 mM NaCl, 10 mM $Na_2HPO_4$, 2.7 mM KCl, 1.8 mM $KH_2PO_4$, pH 7.4) with 5% bio-grade DMSO upon alternating intervals of irradiation, purple: at 365 nm for 1 min to obtain the *cis* isomer for compound **1-8**, **10-12** (for **9**, 6 min), and green: at 520 nm for 30 s.

| Compound | Reversible Photochromism |
|---|---|
| **2** | 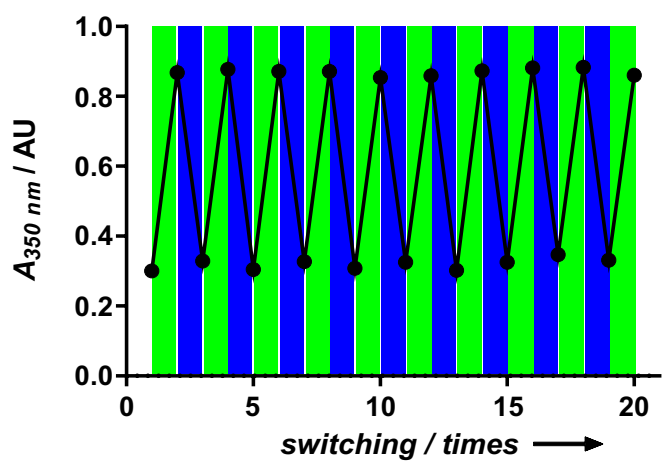 |
| **3** | 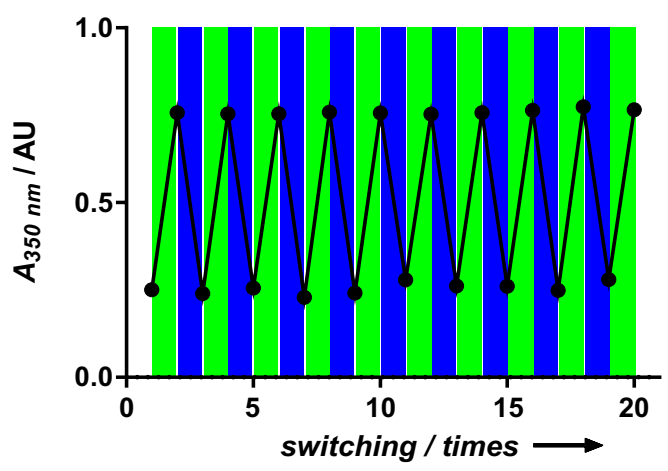 |

**4**

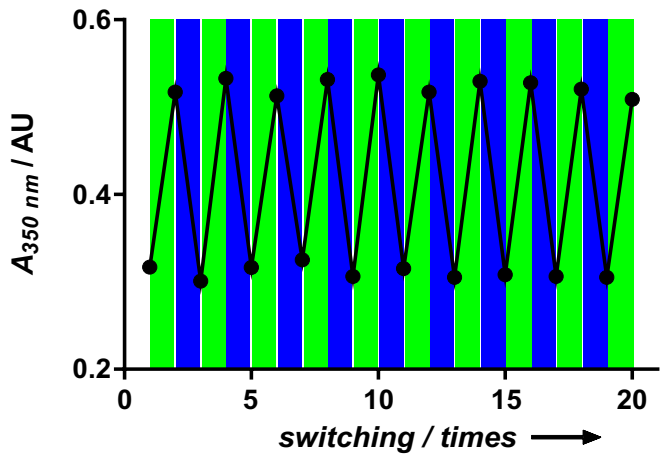
$A_{350\ nm}$ / AU
0.6
0.4
0.2
0
5
10
15
20
switching / times

**5**

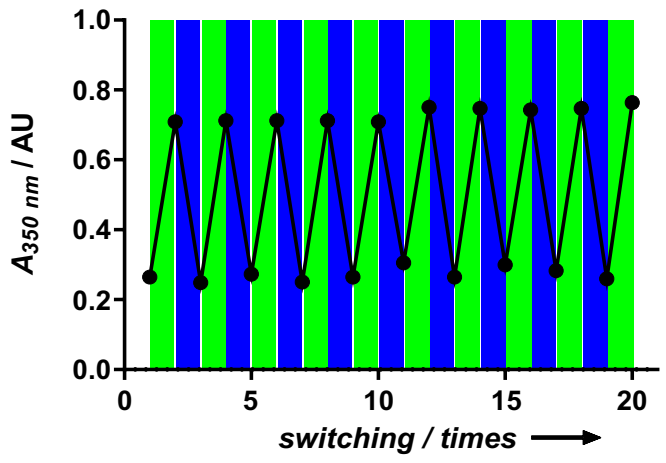
$A_{350\ nm}$ / AU
1.0
0.8
0.6
0.4
0.2
0.0
0
5
10
15
20
switching / times

**6**

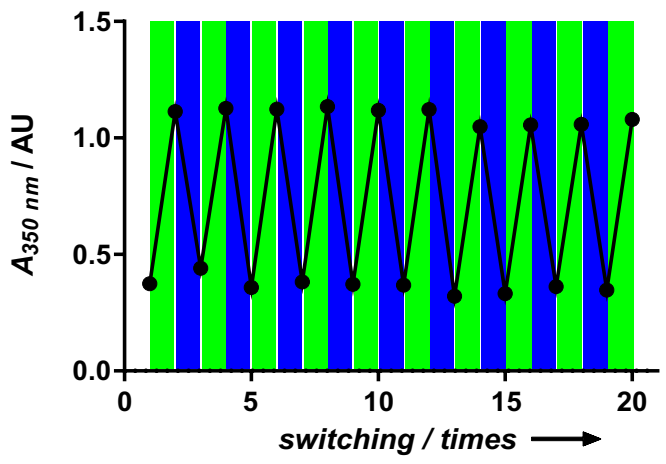
$A_{350\ nm}$ / AU
1.5
1.0
0.5
0.0
0
5
10
15
20
switching / times

**7**

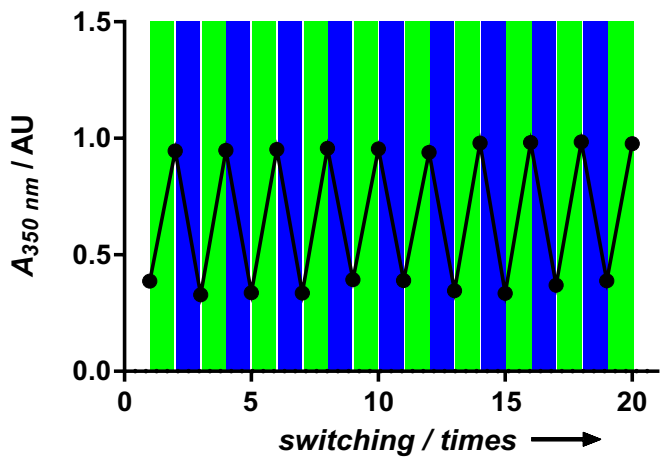
$A_{350\ nm}$ / AU
1.5
1.0
0.5
0.0
0
5
10
15
20
switching / times

**8**

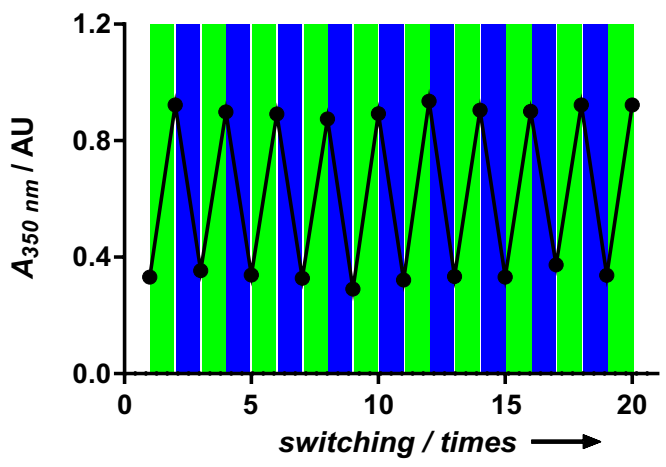
$A_{350\ nm}$ / AU
1.2
0.8
0.4
0.0
0
5
10
15
20
switching / times

**9**

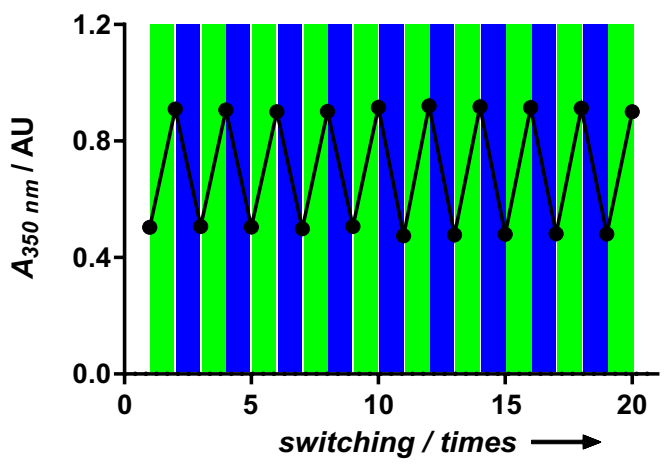
$A_{350\ nm}$ / AU
1.2
0.8
0.4
0.0
0
5
10
15
20
switching / times

**10**

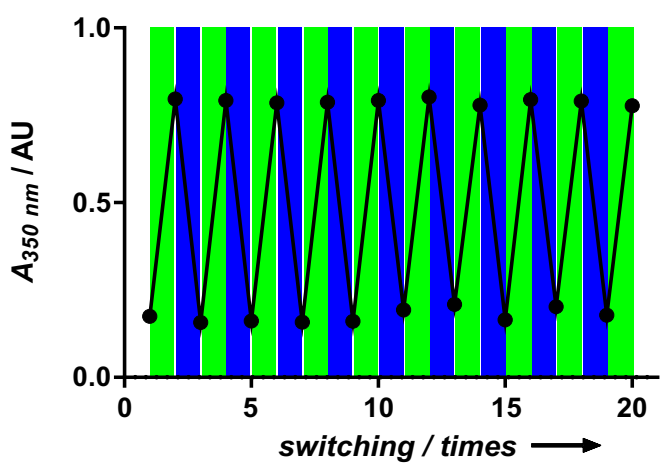
$A_{350\ nm}$ / AU
1.0
0.5
0.0
0
5
10
15
20
switching / times

| | |
|---|---|
| **11** | 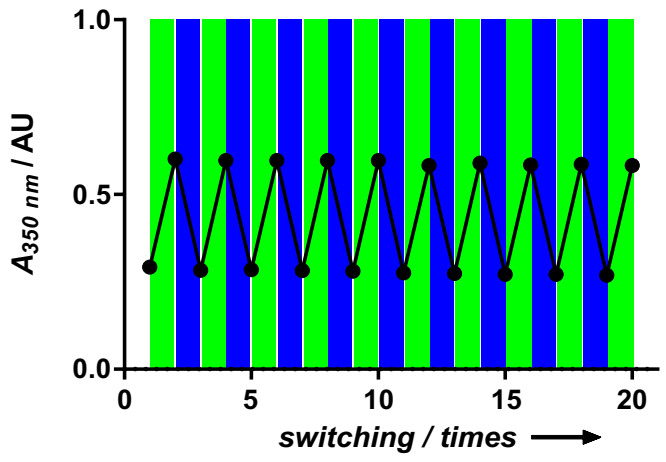  |
| **12** | 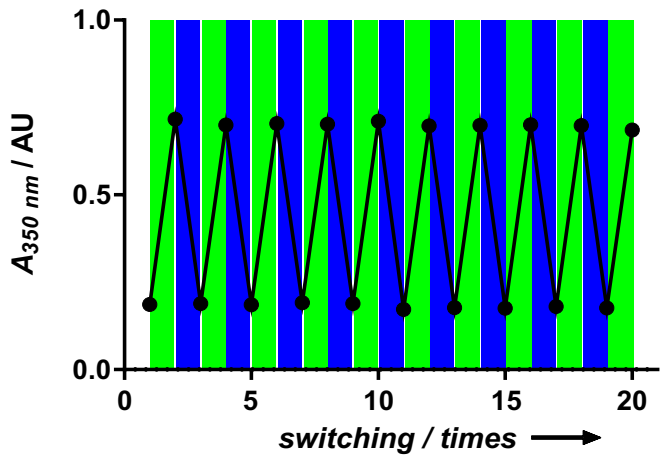  |

*Isomer Ratio Quantification*

Samples were prepared in phosphate buffer (10 mM $NaH_2PO_4$, 150 mM NaCl, pH 7.4) with 5% bio-grade DMSO with a final concentration of 10 µM in the dark. To obtain *cis* isomer, the samples were irradiated at 365 nm for 1 min, for **9**, 6 min. For *trans* conversion, after 365 nm irradiation, the sample was irradiated again at 520 nm for 30 s. The plots resulted from two independent measurements, i.e., from different stock solutions.

**Table S4:** HPLC chromatograms of compounds. Black: sample without irradiation; purple: PSS at 365 nm; green: PSS at 520 nm. HPLC chromatograms were recorded at corresponding isosbestic wavelength. The *cis-trans* ratio was calculated using OpenLab ChemStation software (Agilent Technologies) by calculating the area of the integrated peak.

| Compound | HPLC chromatograms |
|---|---|
| **2** | 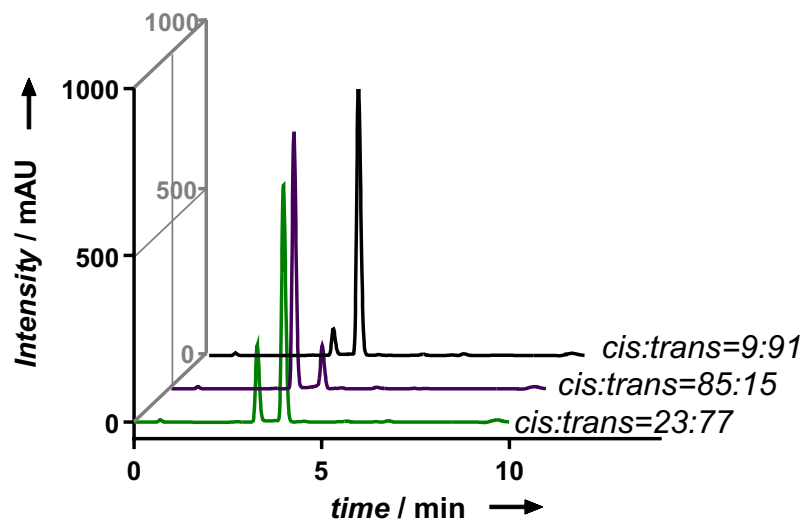  |
| **3** | 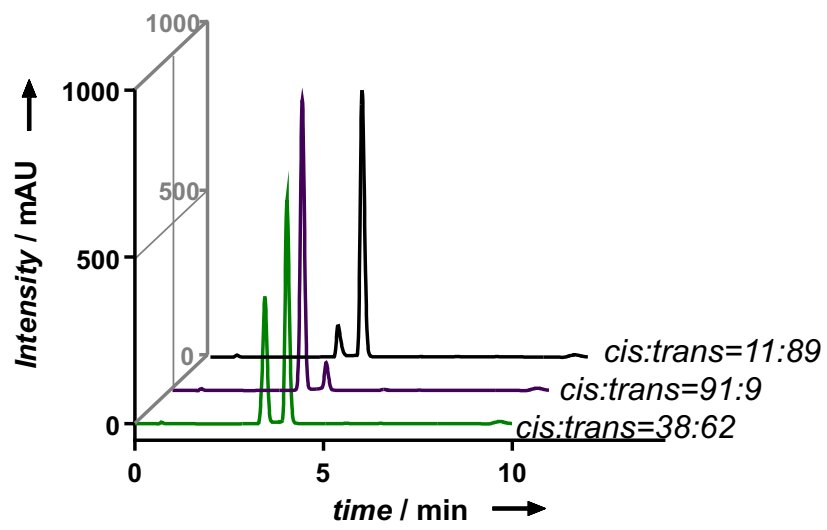  |
| **4** | 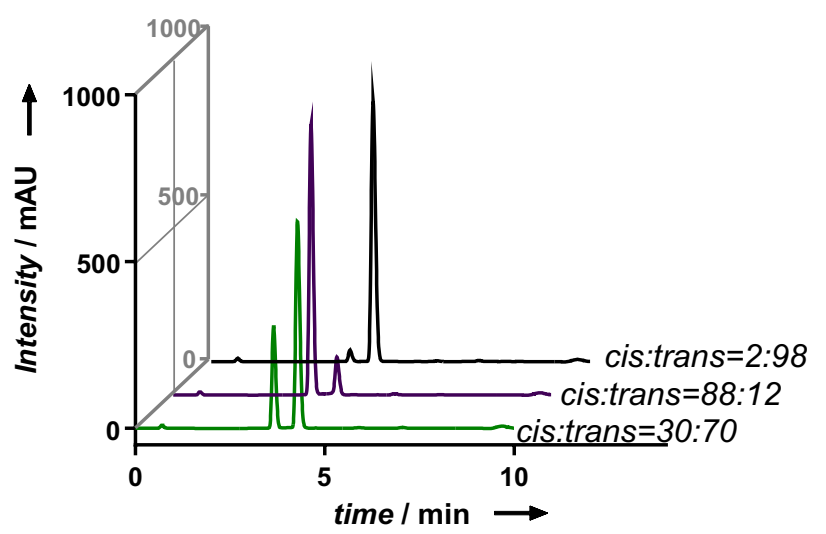  |

**5**

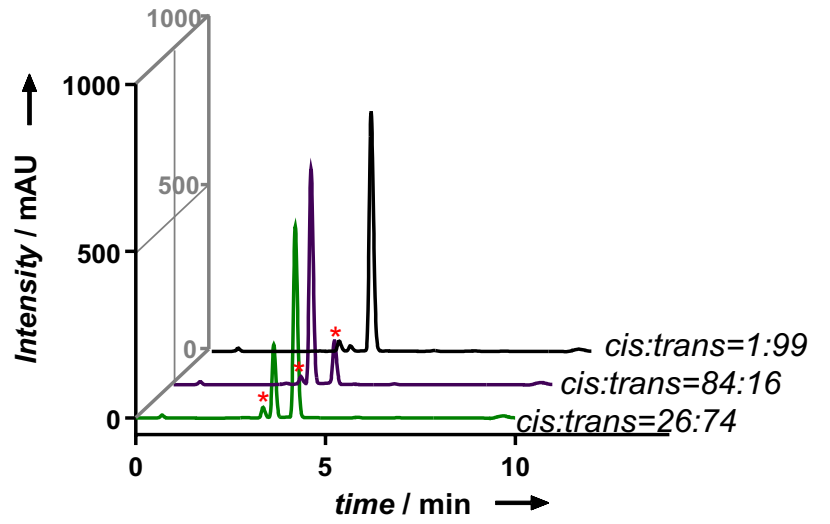
Intensity / mAU
1000
500
0
cis:trans=1:99
cis:trans=84:16
cis:trans=26:74
0
5
10
time / min

**6**

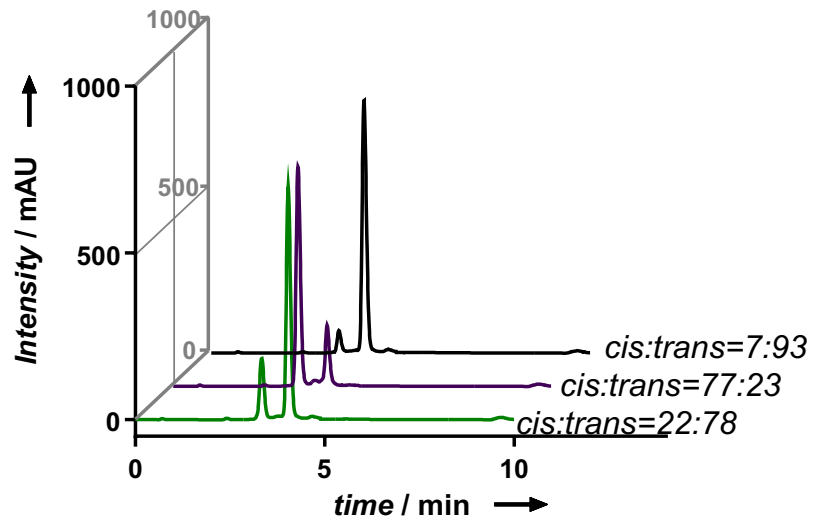
Intensity / mAU
1000
500
0
cis:trans=7:93
cis:trans=77:23
cis:trans=22:78
0
5
10
time / min

**7**

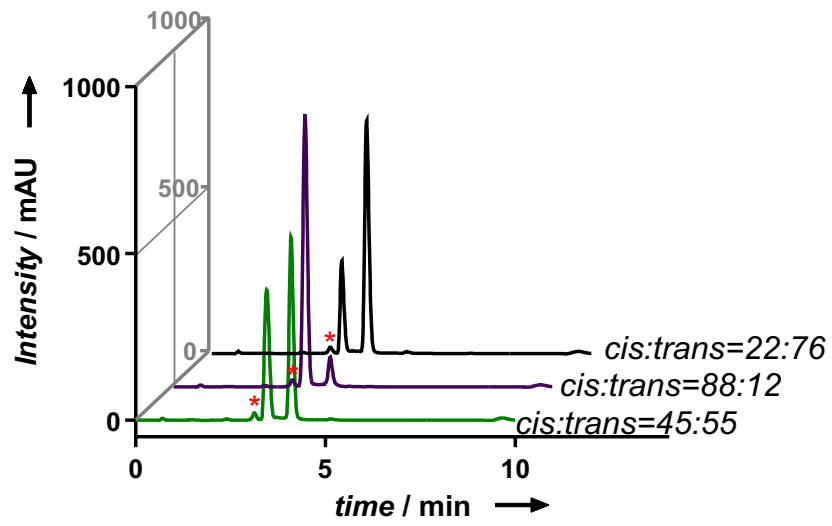
Intensity / mAU
1000
500
0
cis:trans=22:76
cis:trans=88:12
cis:trans=45:55
0
5
10
time / min

**8**

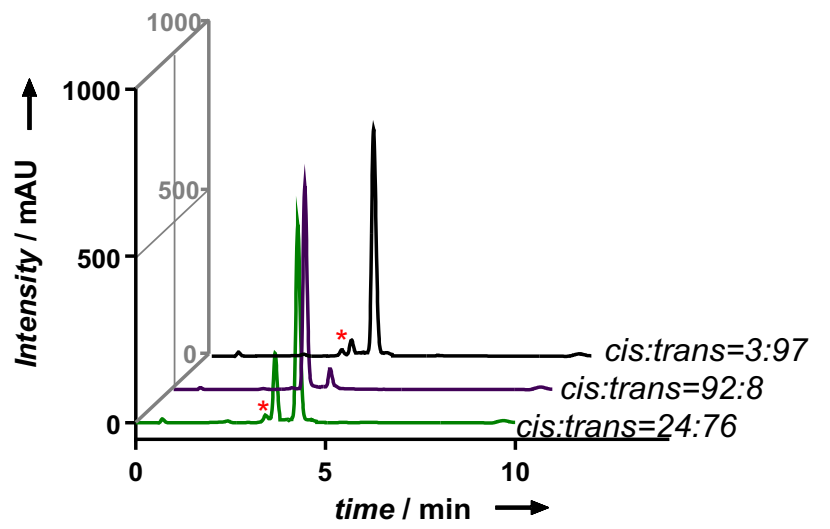
Intensity / mAU
1000
500
0
cis:trans=3:97
cis:trans=92:8
cis:trans=24:76
0
5
10
time / min

**9**

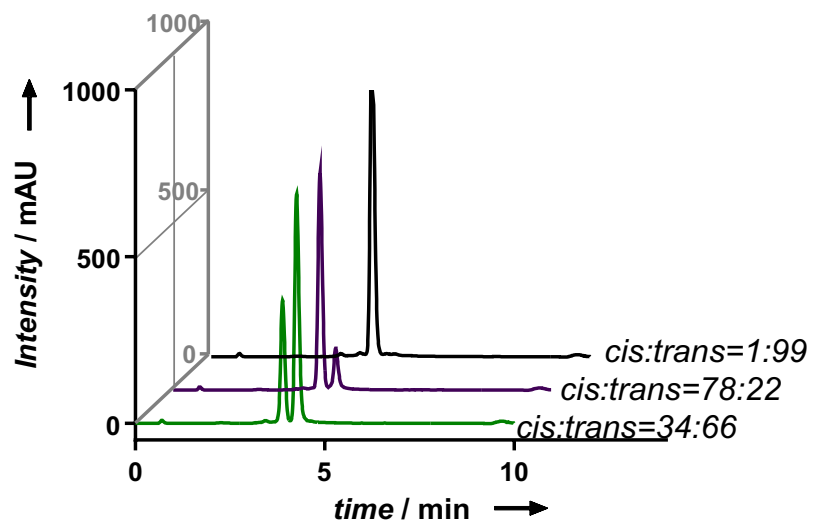
Intensity / mAU
1000
500
0
cis:trans=1:99
cis:trans=78:22
cis:trans=34:66
0
5
10
time / min

**10**

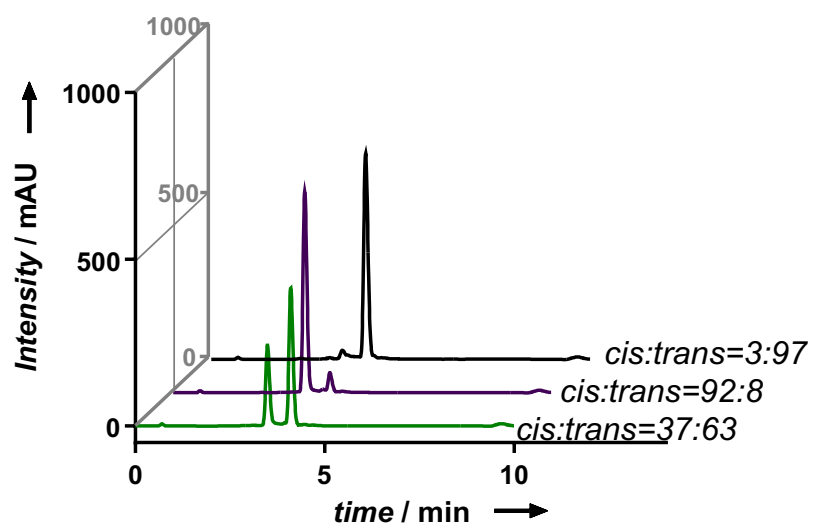
Intensity / mAU
1000
500
0
cis:trans=3:97
cis:trans=92:8
cis:trans=37:63
0
5
10
time / min

| | |
|---|---|
| **11** | 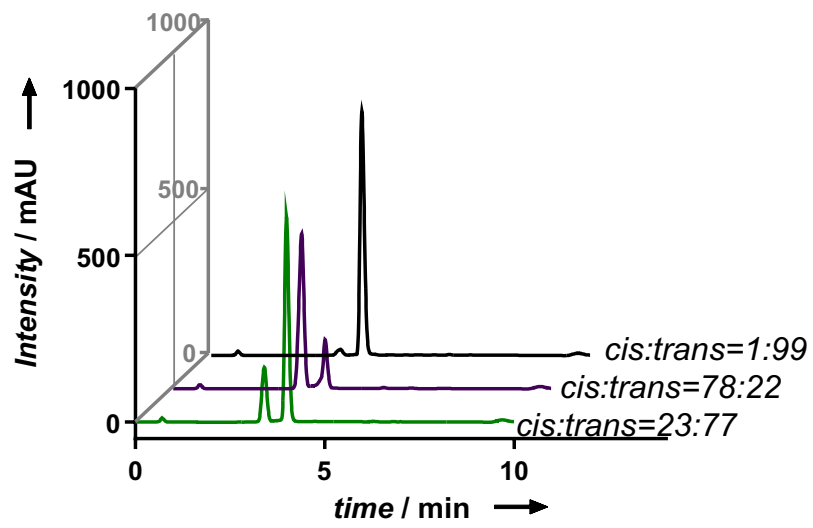  |
| **12** | 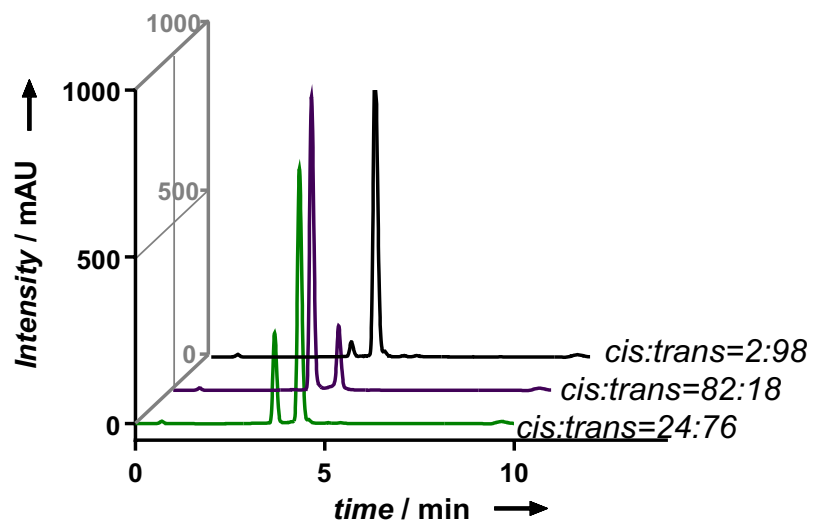  |
| ***Oxidized species during HPLC analytic due to TFA contenting eluent** | |

*Thermal Stability*

The thermal stability of both isomers of each compound was studies at room temperature in the dark. Samples were prepared in ultrapure water/acetonitrile 1:1 with a final concentration of 10 µM in the dark. For *trans* isomer, the sample was protected from light and analyzed with HPLC directly after preparation and after 6 and 24 h. To obtain *cis* isomer, the samples were irradiated at 365 nm for 1 min, for **9**, 6 min, and analyzed with HPLC directly after irradiation and after 3, 6 and 24 h. The plots resulted from two independent measurements, i.e., from different stock solutions.

**Table S5:** HPLC chromatograms of compounds. Stability PSS at 365 nm: after preparation and irradiation, the sample was protected from light and analyzed with HPLC directly after irradiation and after 3, 6 and 24 h; thermal stable photostationary state without irradiation: after preparation, the sample was protected from light and analyzed with HPLC directly after preparation and after 6 and 24 h. HPLC chromatograms were recorded at corresponding isosbestic wavelengths. The values were measured in duplicates. The *cis-trans* ratio was calculated using OpenLab ChemStation software (Agilent Technologies) by calculating the area of the integrated peak.

| Compound | HPLC chromatograms | |
|---|---|---|
| | *PSS$_{365\ nm}$* | *photostationary state without irradiation* |
| **2** | 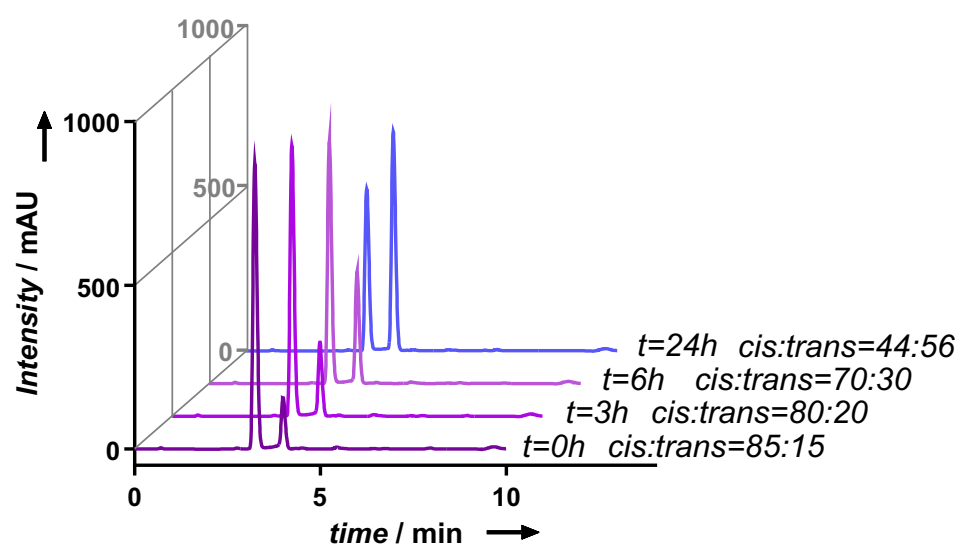  | 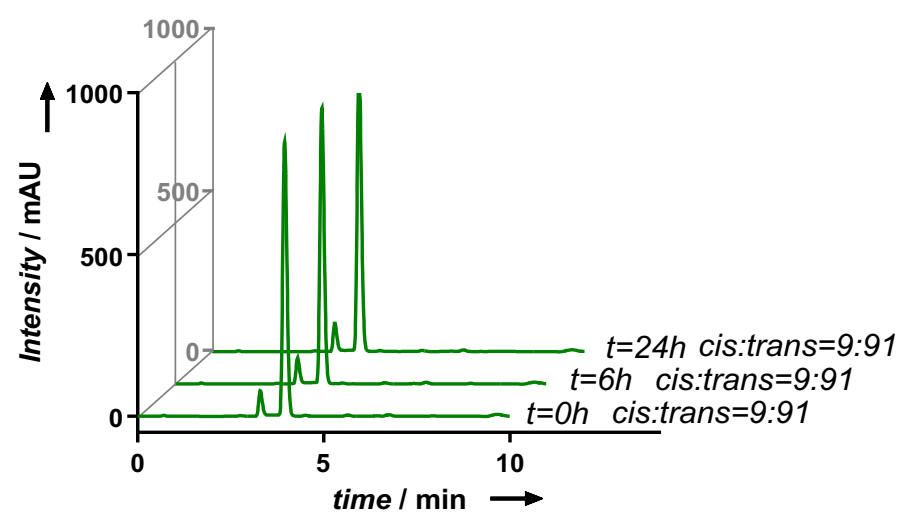  |
| **3** | 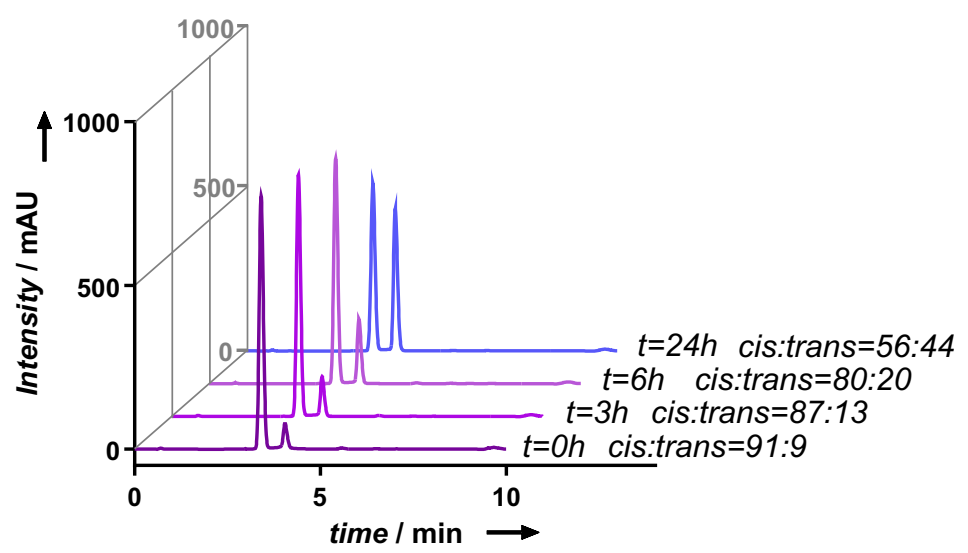  | 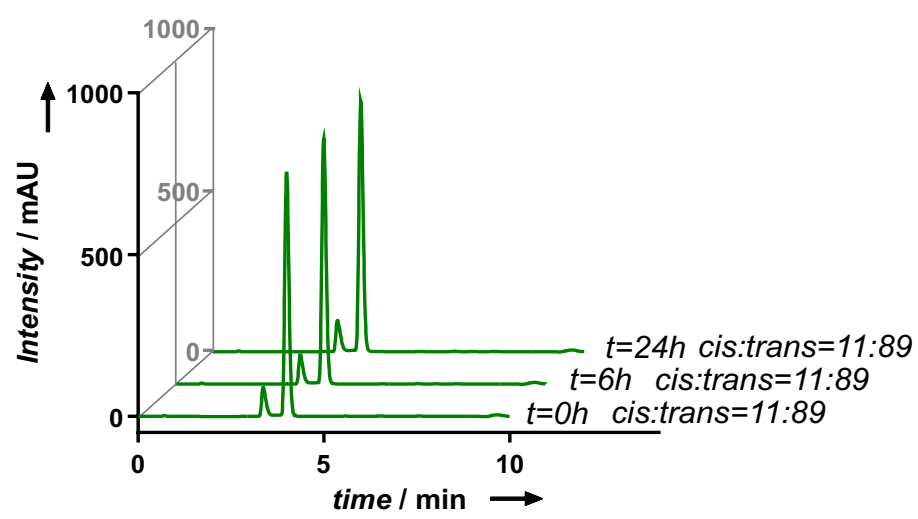  |

**4**

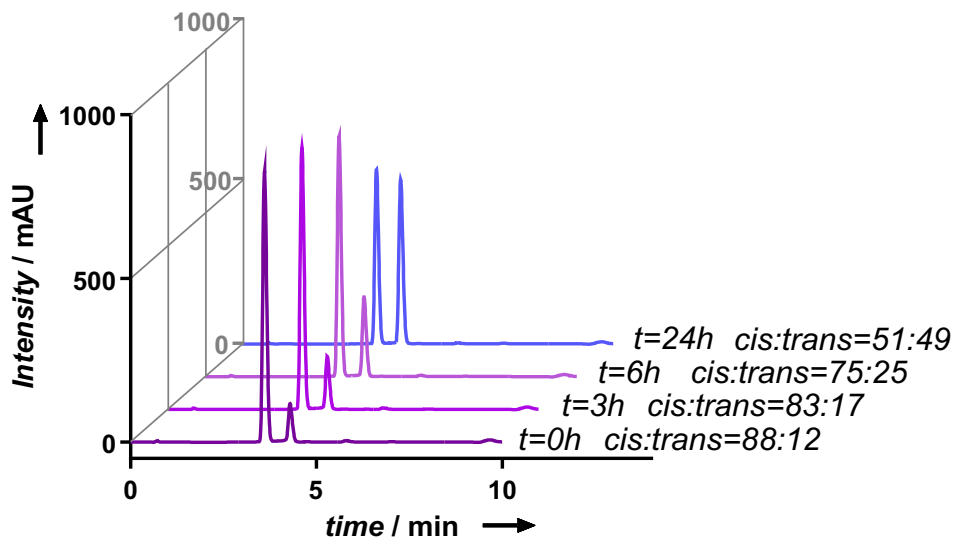
Intensity / mAU
time / min
t=24h cis:trans=51:49
t=6h cis:trans=75:25
t=3h cis:trans=83:17
t=0h cis:trans=88:12

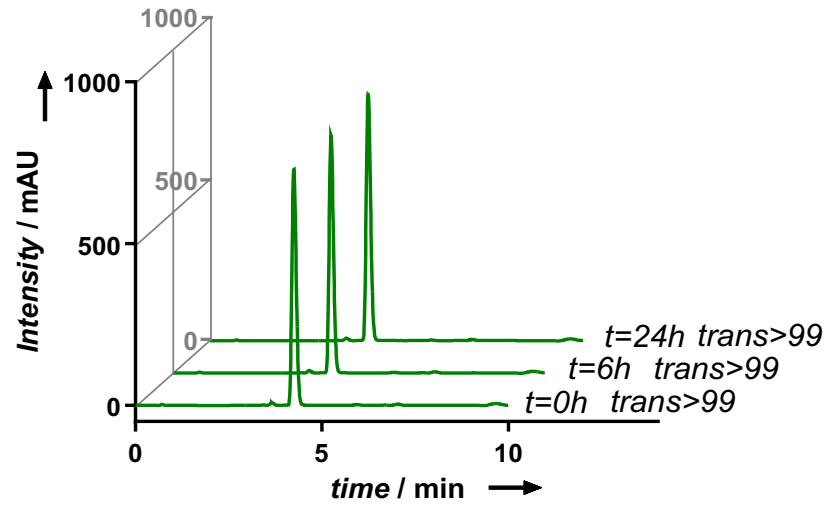
Intensity / mAU
time / min
t=24h trans>99
t=6h trans>99
t=0h trans>99

**5**

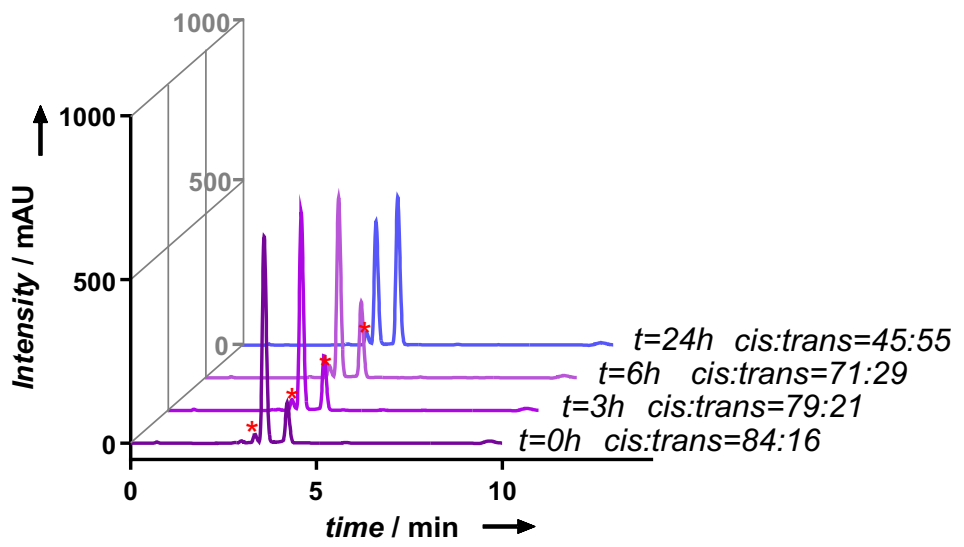
Intensity / mAU
time / min
t=24h cis:trans=45:55
t=6h cis:trans=71:29
t=3h cis:trans=79:21
t=0h cis:trans=84:16

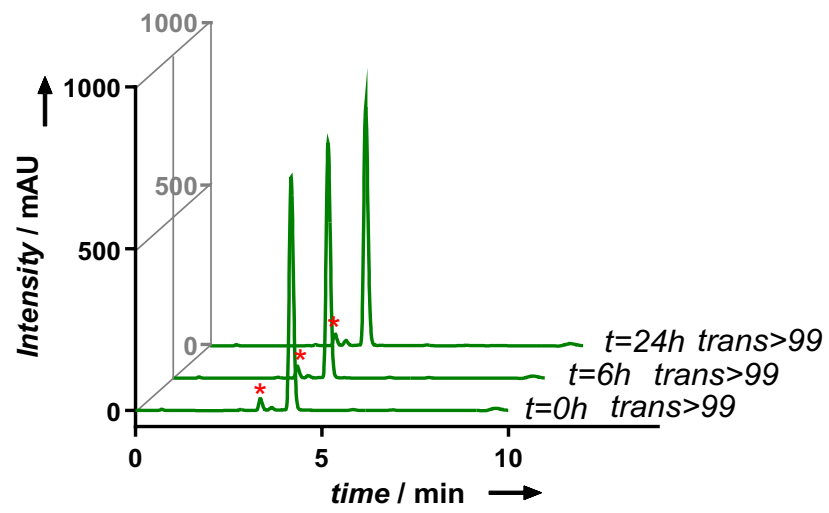
Intensity / mAU
time / min
t=24h trans>99
t=6h trans>99
t=0h trans>99

**6**

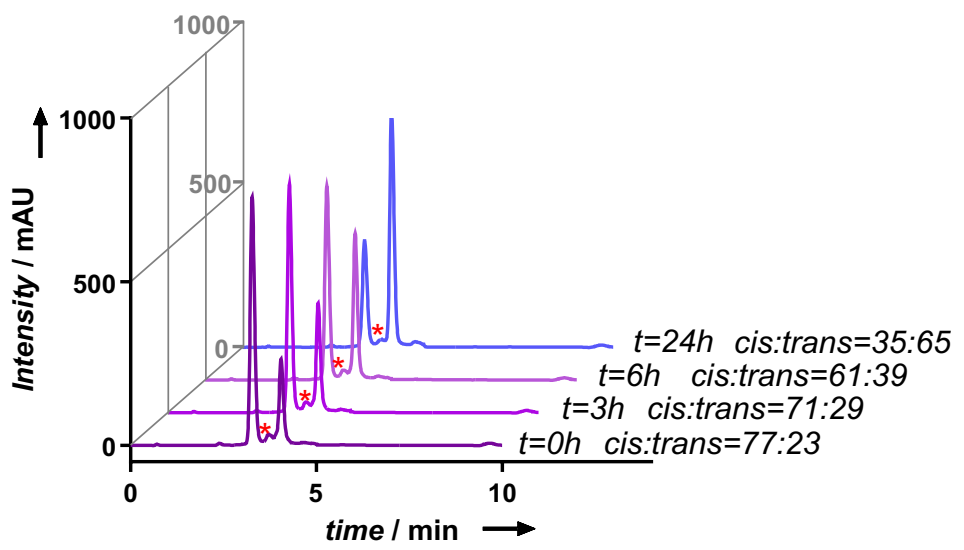
Intensity / mAU
time / min
t=24h cis:trans=35:65
t=6h cis:trans=61:39
t=3h cis:trans=71:29
t=0h cis:trans=77:23

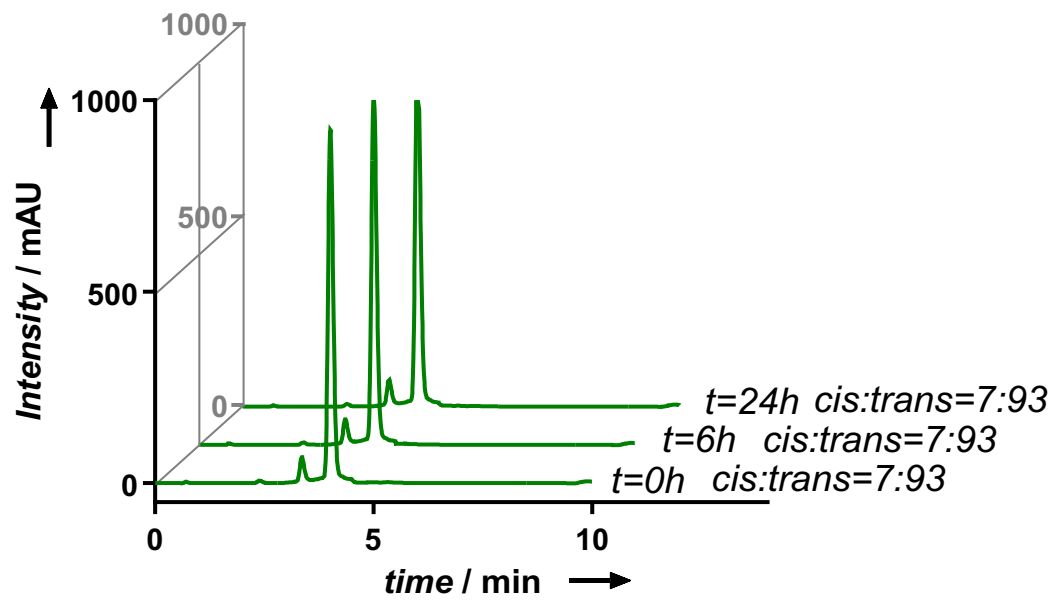
Intensity / mAU
time / min
t=24h cis:trans=7:93
t=6h cis:trans=7:93
t=0h cis:trans=7:93

**7**

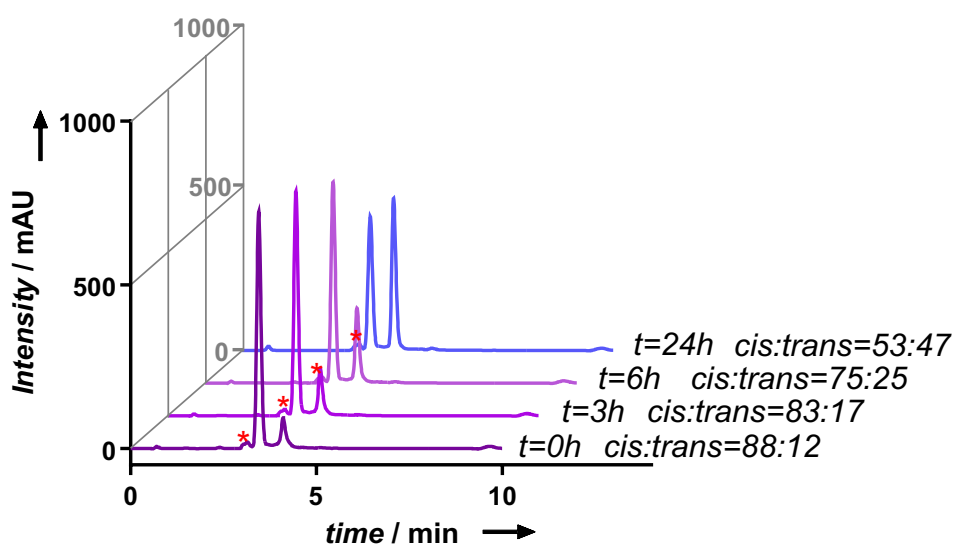
Intensity / mAU
time / min
t=24h cis:trans=53:47
t=6h cis:trans=75:25
t=3h cis:trans=83:17
t=0h cis:trans=88:12

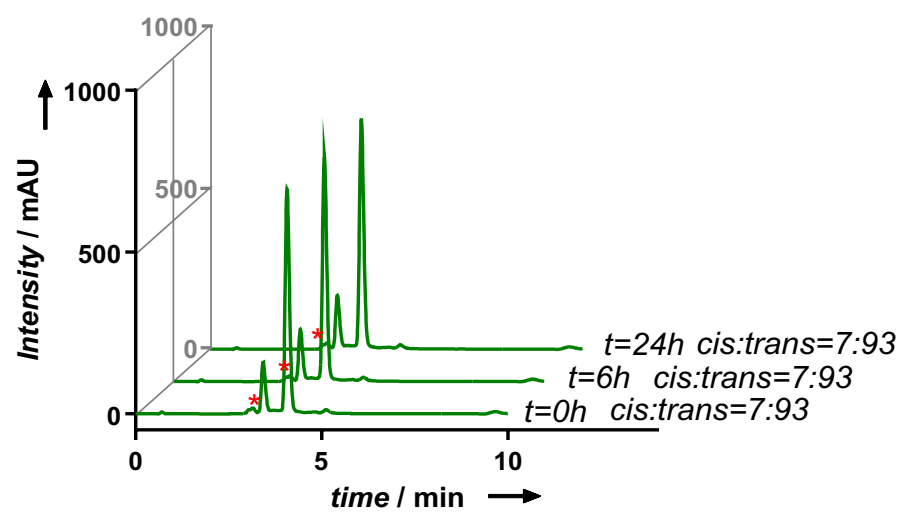
Intensity / mAU
time / min
t=24h cis:trans=7:93
t=6h cis:trans=7:93
t=0h cis:trans=7:93

**8**

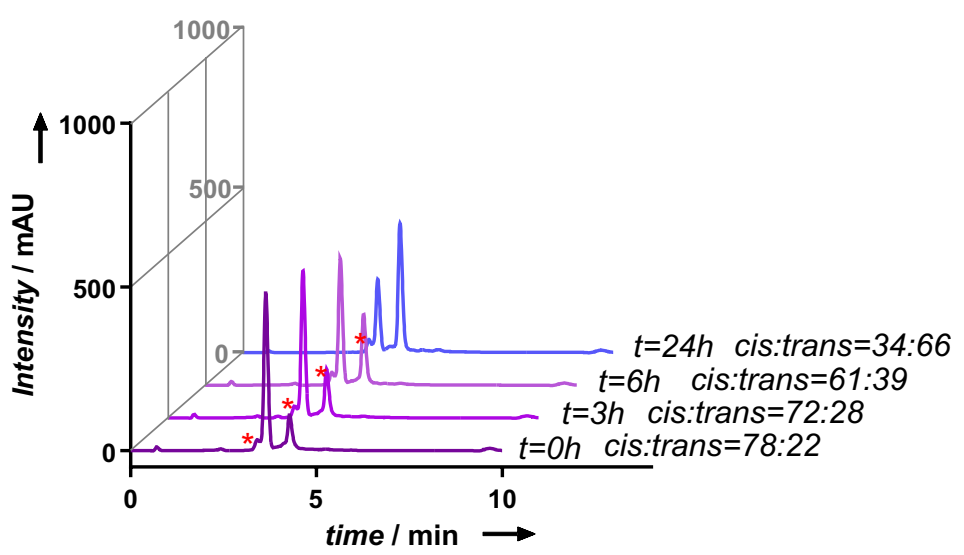
Intensity / mAU
time / min
t=24h cis:trans=34:66
t=6h cis:trans=61:39
t=3h cis:trans=72:28
t=0h cis:trans=78:22

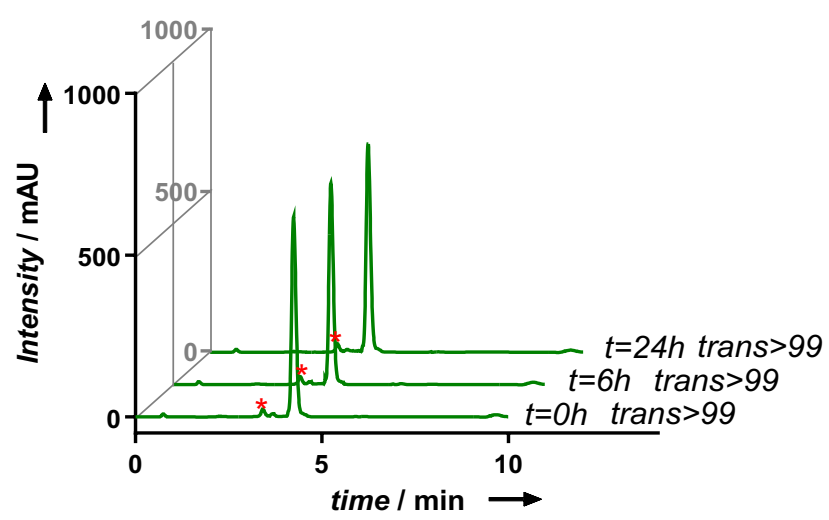
Intensity / mAU
time / min
t=24h trans>99
t=6h trans>99
t=0h trans>99

**9**

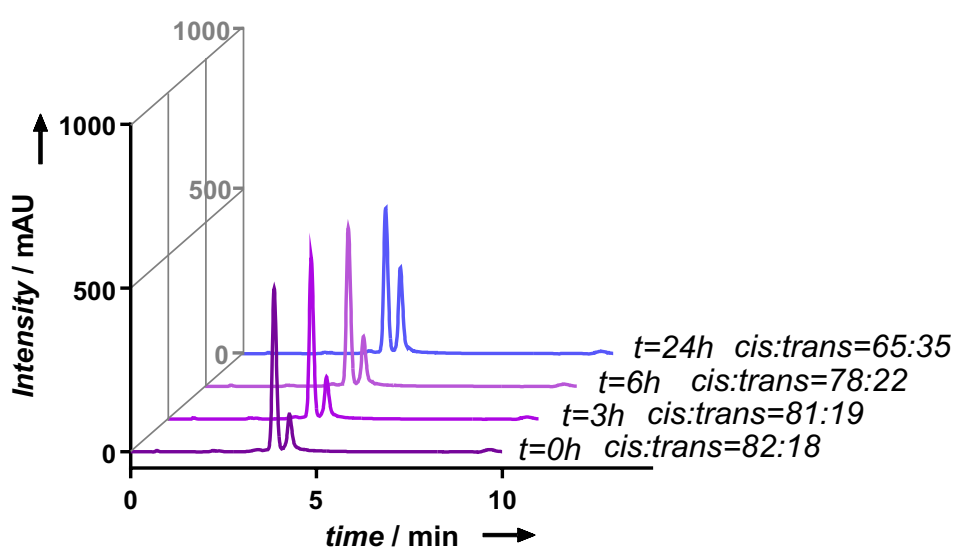
Intensity / mAU
time / min
t=24h cis:trans=65:35
t=6h cis:trans=78:22
t=3h cis:trans=81:19
t=0h cis:trans=82:18

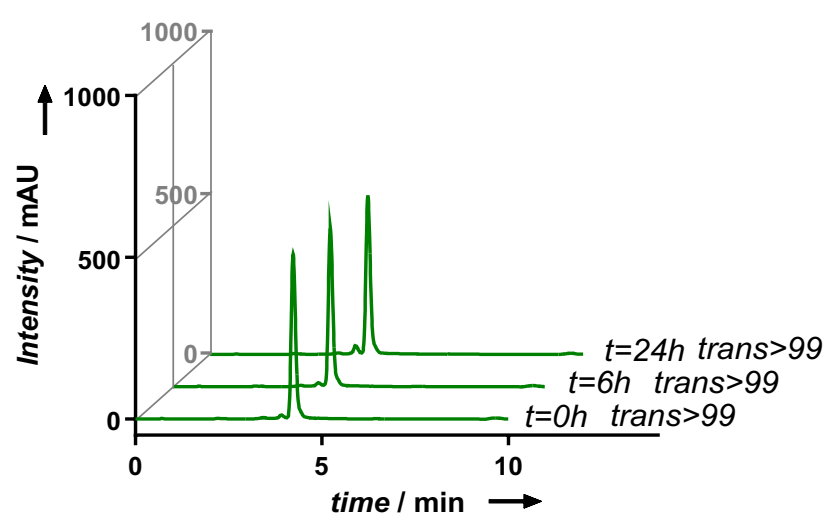
Intensity / mAU
time / min
t=24h trans>99
t=6h trans>99
t=0h trans>99

**10**

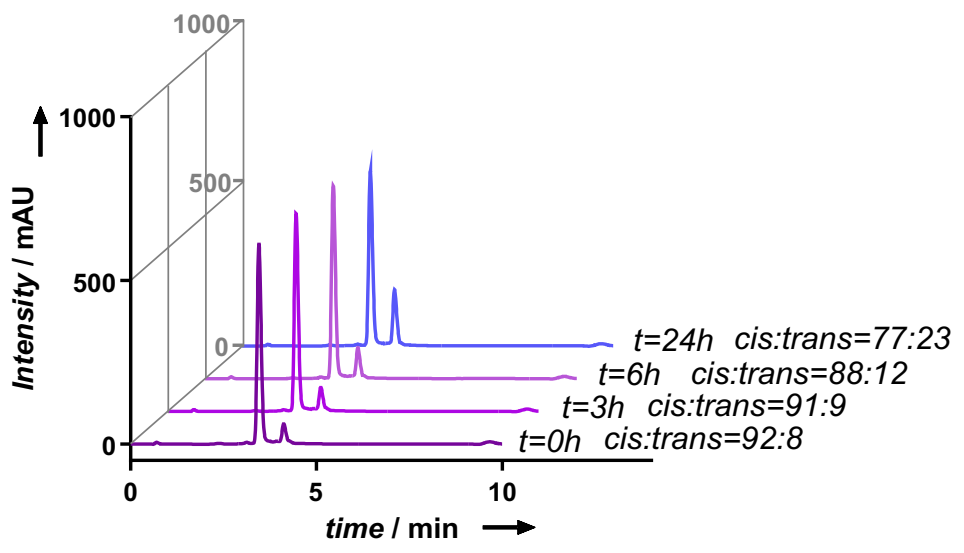


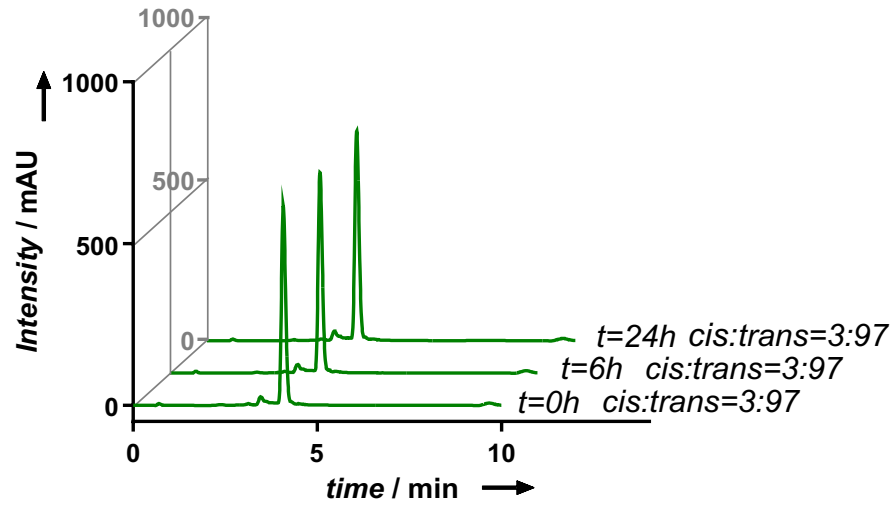


**11**

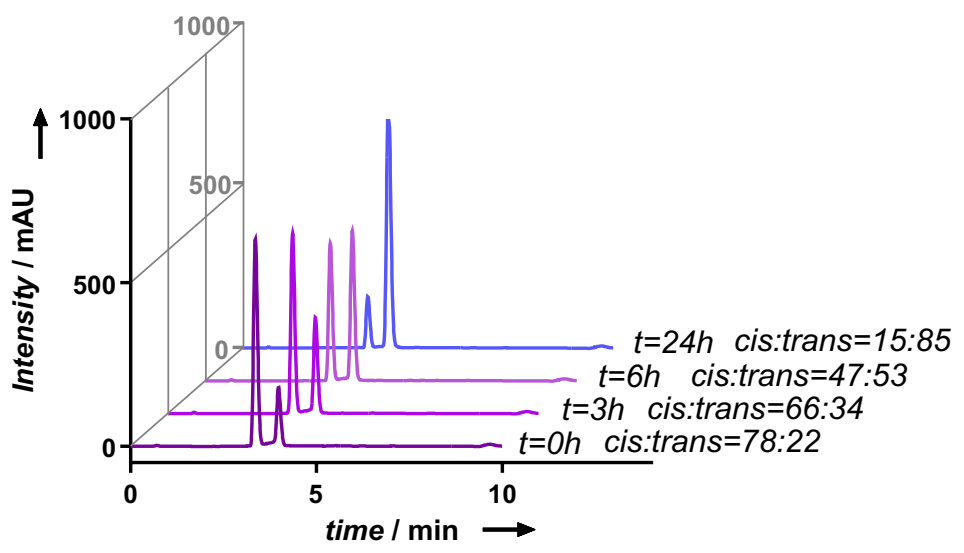


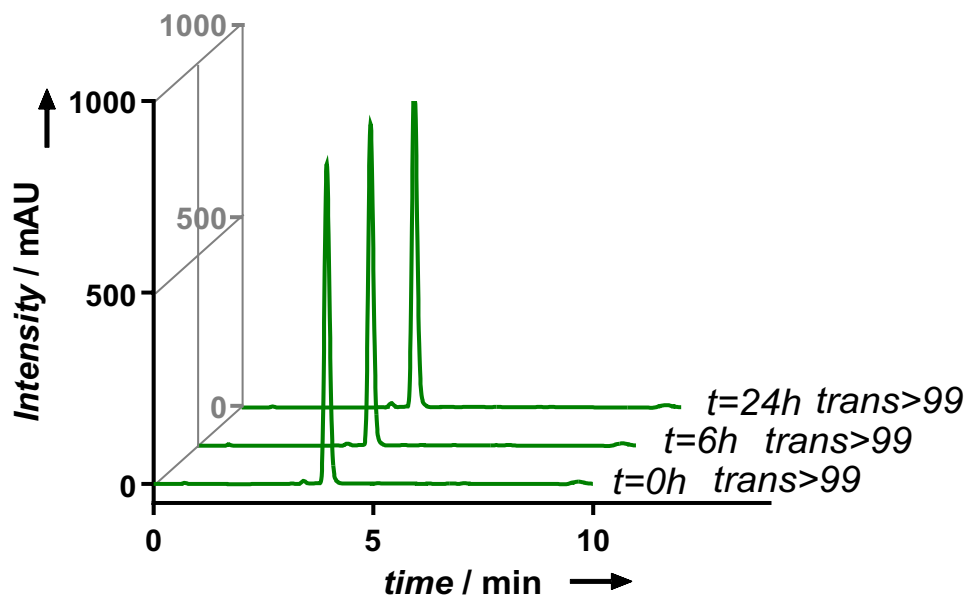


**12**

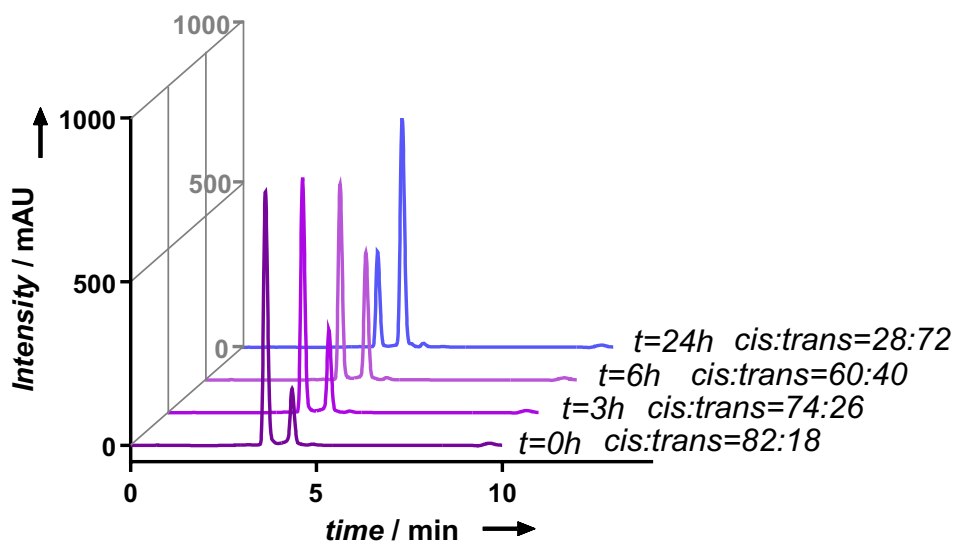


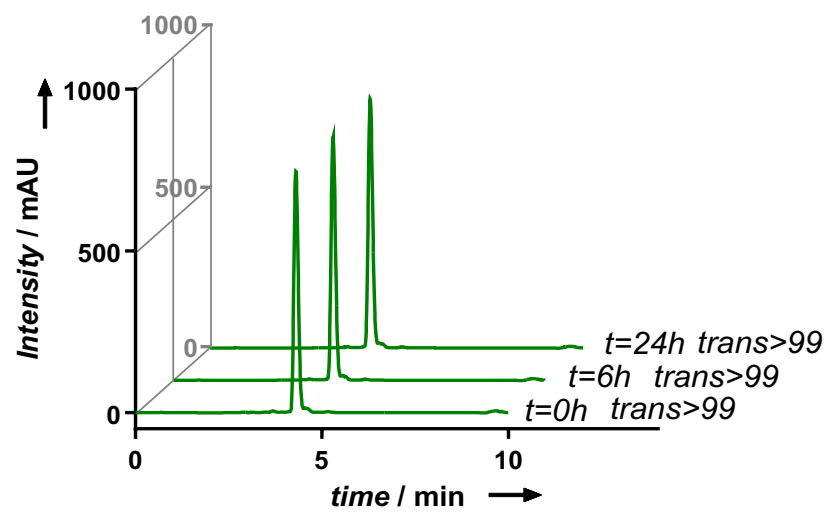


***Oxidized species during HPLC analytic due to TFA contenting eluent.**

*Photoisomerization Quantum Yield*

The absorbance of tris-phenanthroline iron (II) complex after irradiation at 365 nm at different time intervals at 510 nm was plotted against time. Data analysis was performed by linear regression with software Graphpad Prism 8.0. (Figure S93)
The slope was determined as $(7.721\pm0.256)\cdot10^{-3}$ /s and photon flux *I* of 365 nm LED was obtained as $(2.156\pm0.071)\cdot10^{-8}$ mol/s following the general procedure and calculation explained in section 'Ultraviolet-visible Spectroscopy (UV-vis)'.

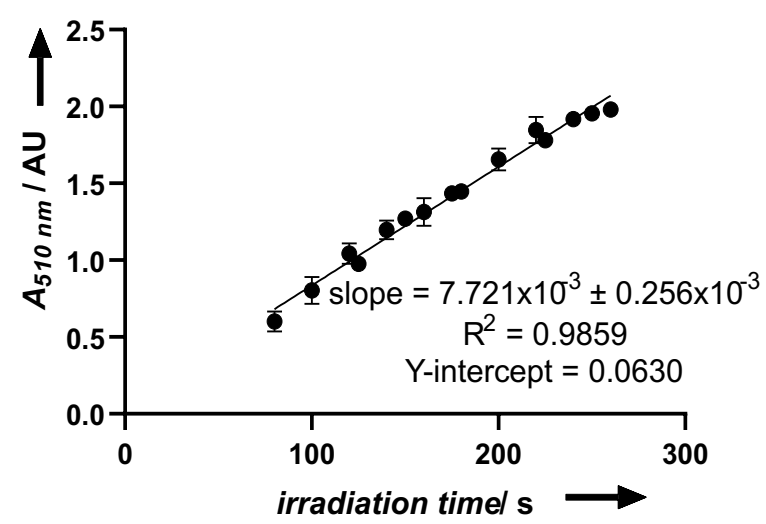


**Figure S93.** Absorbance of the tris-phenanthroline iron (II) complex as a function of irradiation time. The plots resulted from three independent measurements, i.e., from different stock solutions. For all values, the corresponding background signal was subtracted.

Aberchrome 670 was irradiated at 525 nm at different time intervals. The concentration of ring-closed aberchrome 670 changes upon 520 nm irradiation was plotted against irradiation time. Data analysis was performed by linear regression with software Graphpad Prism 8.0. (Figure S94)

The photon flux *I* of 525 nm LED was obtained as (6.11±0.29)·$10^{-9}$ mol/s following the general procedure and calculation explained in section 'Photoisomerization Quantum Yield'.

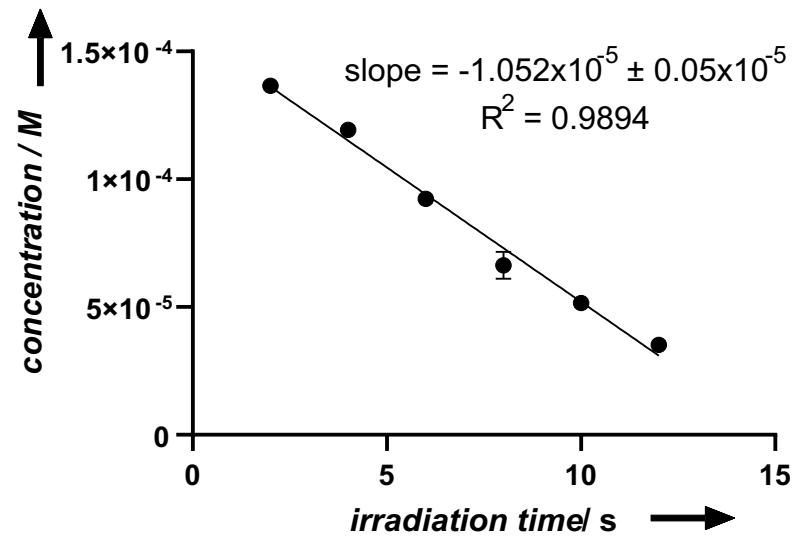


**Figure S94.** Concentration of the ring-closed aberchrome 670 as a function of irradiation time. The plots resulted from three independent measurements, i.e., from different stock solutions. For all values, the corresponding background signal was subtracted.

Photoisomerization quantum yields were calculated and summarized in table S6.

**Table S6**: Calculated photoisomerization quantum yields of each compound. Samples were prepared with a concentration of 50 µM freshly in PBS buffer (140 mM NaCl, 10 mM $Na_2HPO_4$, 2.7 mM KCl, 1.8 mM $KH_2PO_4$, pH 7.4) with 5% bio-grade DMSO. Changes of concentration of PSSs of each compound were calculated with ratio of two PSSs, which were analyzed via HPLC (Table S5). The determination was repeated three times independently i.e., from three different stock solutions.

| compound | $\Phi_{trans\to cis}$ | $\Phi_{cis\to trans}$ |
|---|---|---|
| **2** | 0.317±0.011 | 0.451±0.021 |
| **3** | 0.324±0.011 | 0.284±0.014 |
| **4** | 0.340±0.011 | 0.241±0.011 |
| **5** | 0.478±0.016 | 0.364±0.017 |
| **6** | 0.272±0.009 | 0.528±0.025 |
| **7** | 0.331±0.011 | 0.152±0.007 |
| **8** | 0.383±0.013 | 0.487±0.023 |
| **9** | 0.083±0.003 | 0.228±0.011 |
| **10** | 0.468±0.016 | 0.581±0.028 |
| **11** | 0.537±0.018 | 0.438±0.021 |
| **12** | 0.375±0.012 | 0.489±0.023 |

## Cell-based Assays

### Cell Viability Assays

The viability of HEK293T-SMN2-Luc cells in presence of corresponding compound at different concentrations are summarized in table S7. Mean data-points and standard deviations are derived from three independent experiments, each concentration in triplicate.

**Table S7**: Effect of different concentrations of corresponding compound in HEK293T-SMN2-Luc cell viability. Mean data-points and standard deviations are derived from three independent experiments, each concentration in triplicate. Purple: treatment with PPS at 365 nm, green: treatment with PPS without irradiation; gray: treatment with 0.25% bio-grade DMSO in sterile water; black: untreated.

| Compound | Cell Viability | |
|---|---|---|
| **2** | 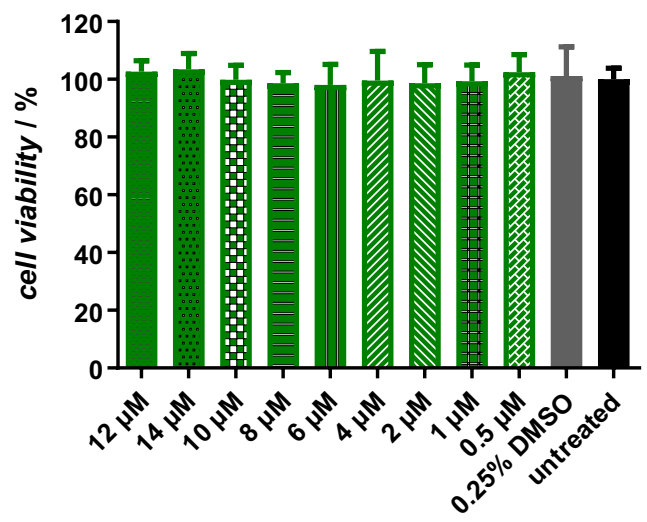 | 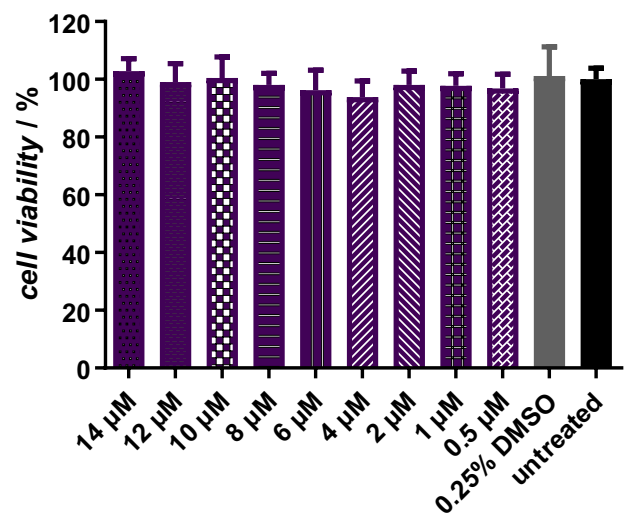 |
| **3** | 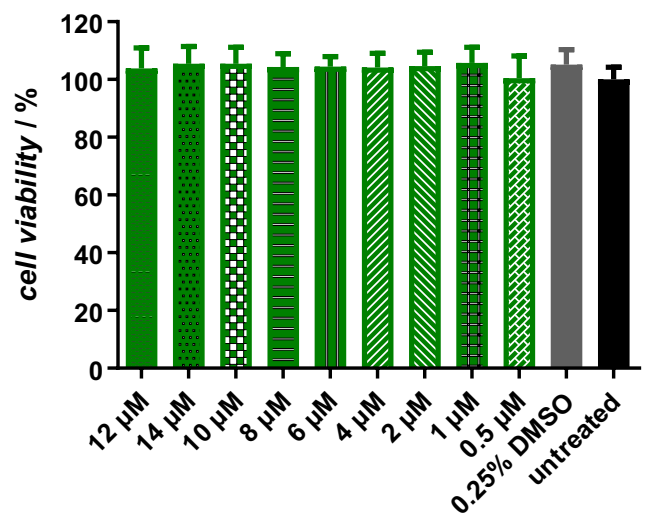 | 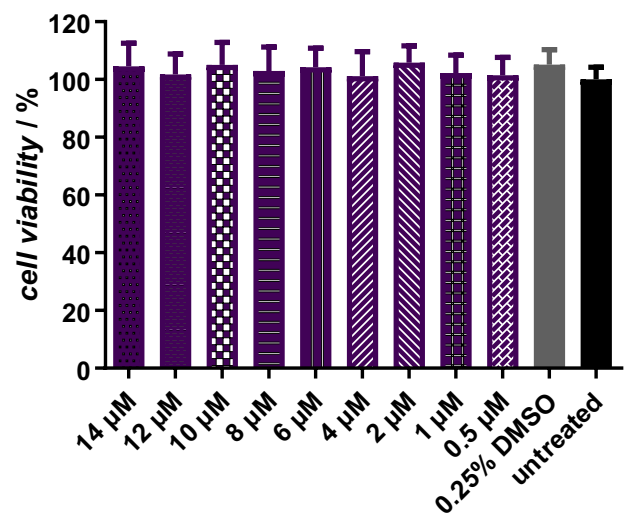 |
| **4** | 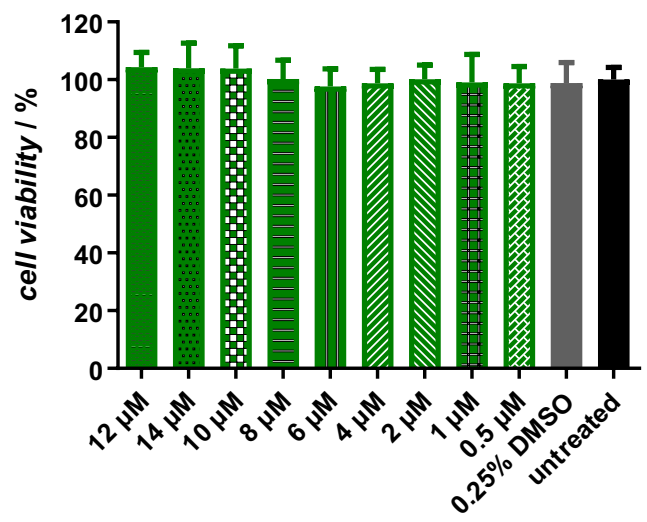 | 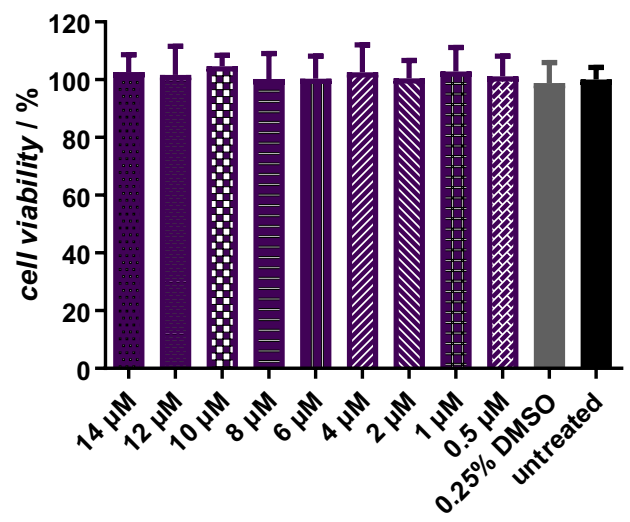 |
| **5** | 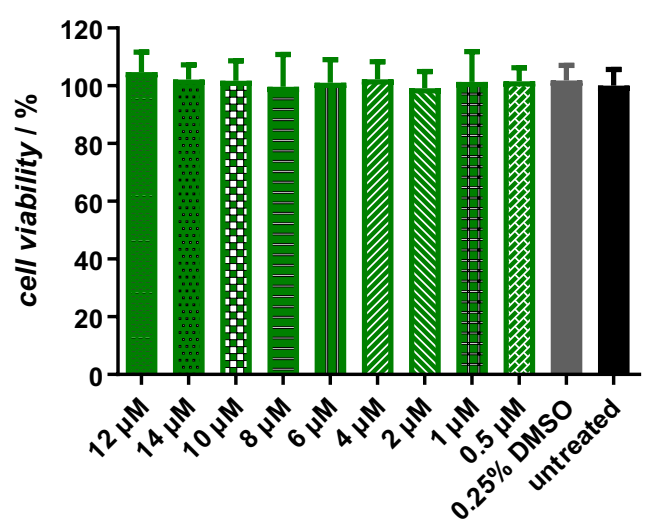 | 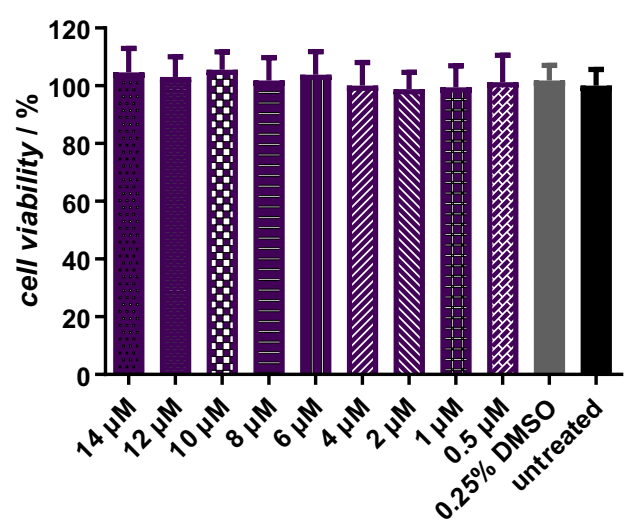 |

**6**

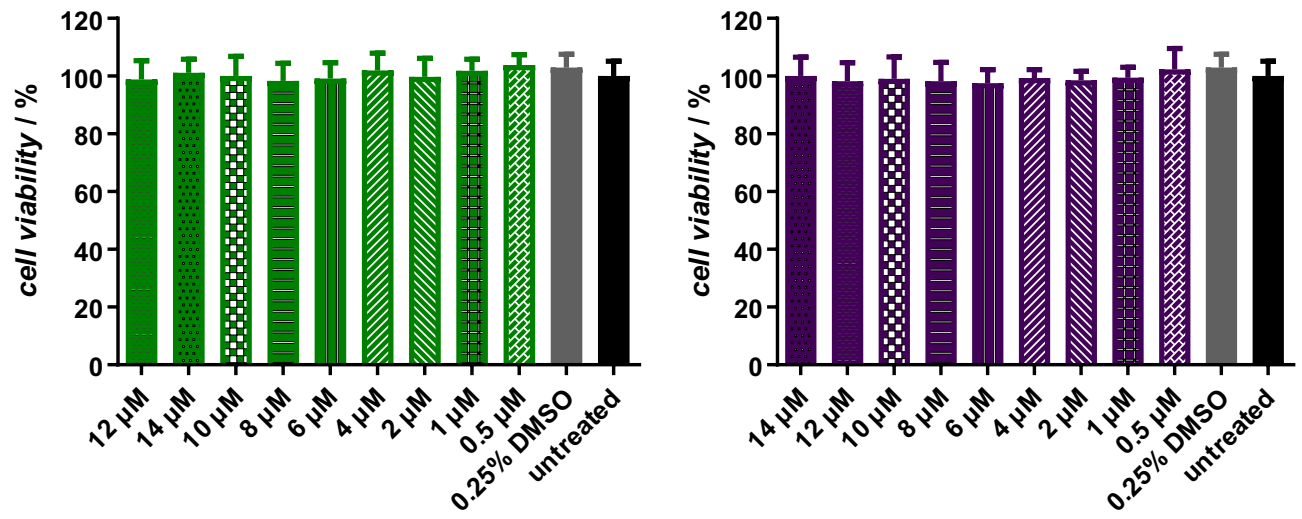
cell viability / %
0
20
40
60
80
100
120
12 µM
14 µM
10 µM
8 µM
6 µM
4 µM
2 µM
1 µM
0.5 µM
0.25% DMSO
untreated
cell viability / %
0
20
40
60
80
100
120
14 µM
12 µM
10 µM
8 µM
6 µM
4 µM
2 µM
1 µM
0.5 µM
0.25% DMSO
untreated

**7**

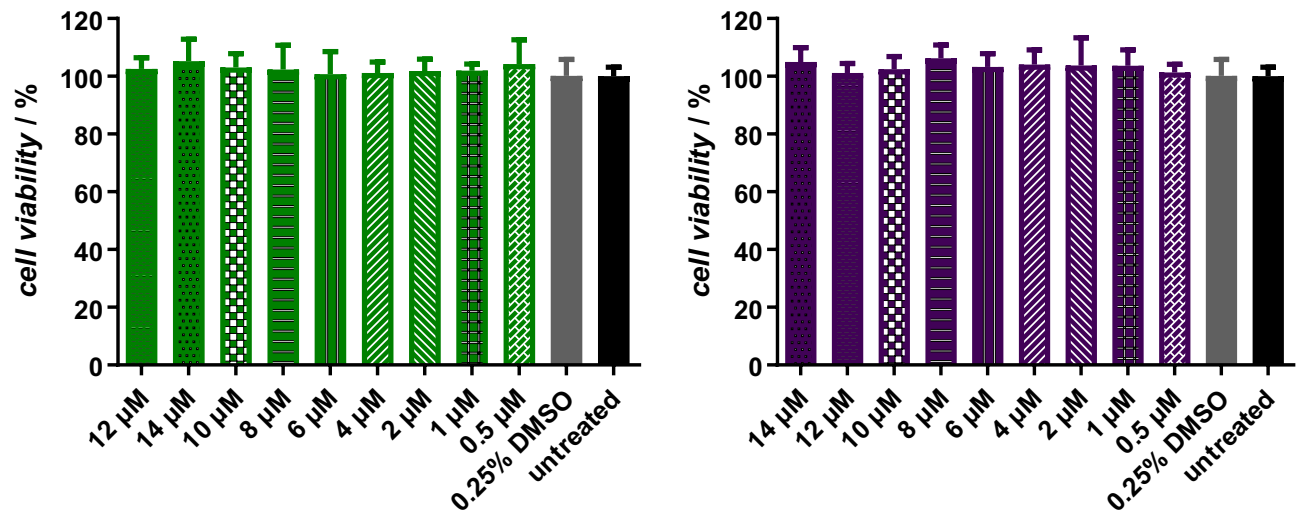
cell viability / %
0
20
40
60
80
100
120
12 µM
14 µM
10 µM
8 µM
6 µM
4 µM
2 µM
1 µM
0.5 µM
0.25% DMSO
untreated
cell viability / %
0
20
40
60
80
100
120
14 µM
12 µM
10 µM
8 µM
6 µM
4 µM
2 µM
1 µM
0.5 µM
0.25% DMSO
untreated

**8**

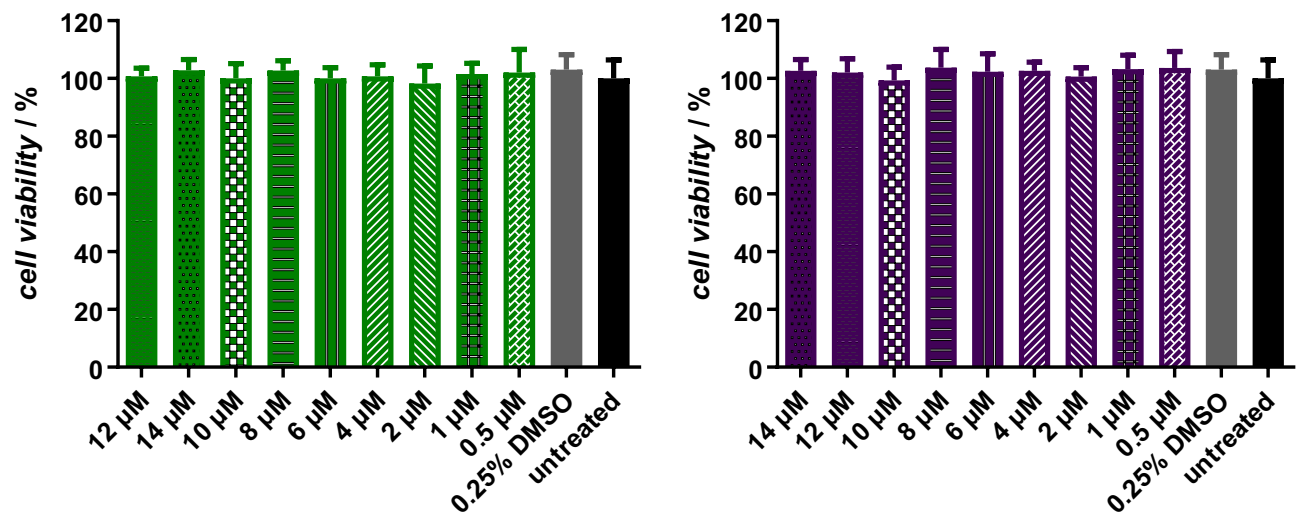
cell viability / %
0
20
40
60
80
100
120
12 µM
14 µM
10 µM
8 µM
6 µM
4 µM
2 µM
1 µM
0.5 µM
0.25% DMSO
untreated
cell viability / %
0
20
40
60
80
100
120
14 µM
12 µM
10 µM
8 µM
6 µM
4 µM
2 µM
1 µM
0.5 µM
0.25% DMSO
untreated

**9**

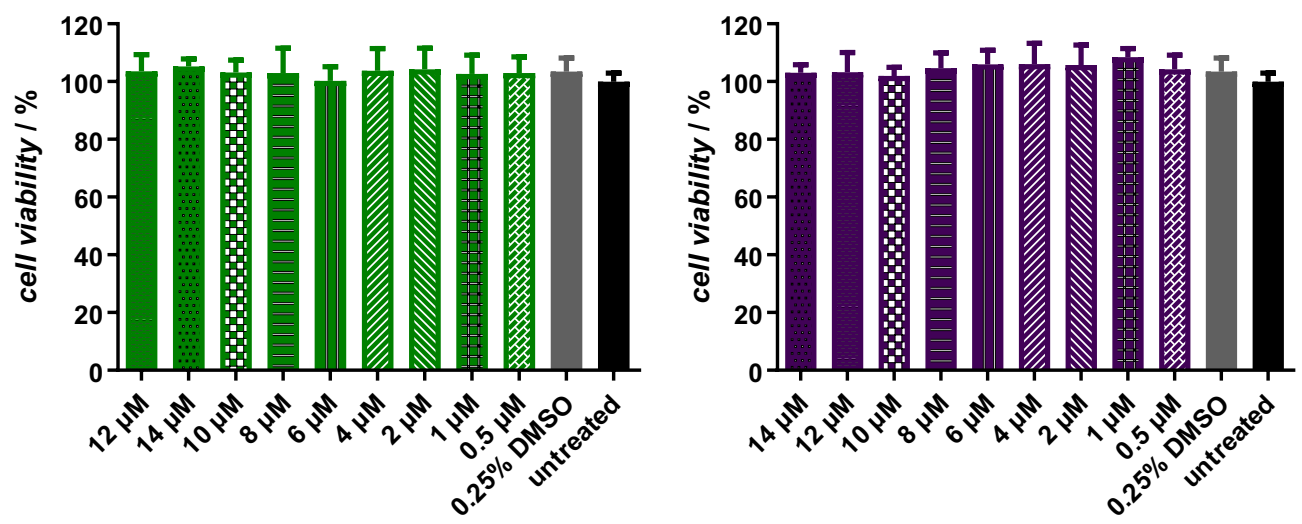
cell viability / %
0
20
40
60
80
100
120
12 µM
14 µM
10 µM
8 µM
6 µM
4 µM
2 µM
1 µM
0.5 µM
0.25% DMSO
untreated
cell viability / %
0
20
40
60
80
100
120
14 µM
12 µM
10 µM
8 µM
6 µM
4 µM
2 µM
1 µM
0.5 µM
0.25% DMSO
untreated

**10**

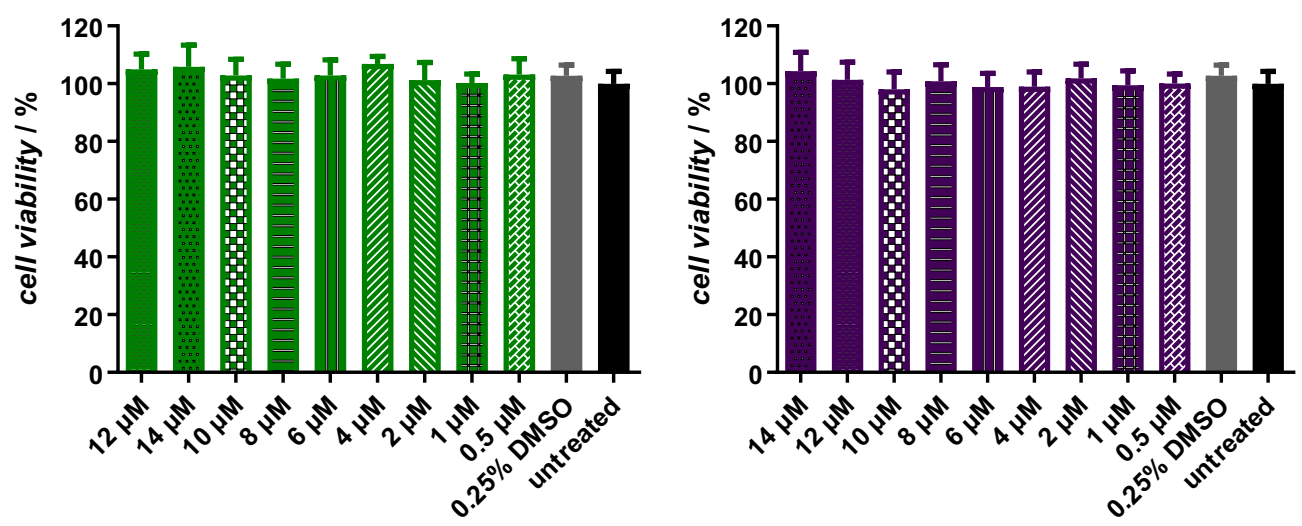
cell viability / %
0
20
40
60
80
100
120
12 µM
14 µM
10 µM
8 µM
6 µM
4 µM
2 µM
1 µM
0.5 µM
0.25% DMSO
untreated
cell viability / %
0
20
40
60
80
100
120
14 µM
12 µM
10 µM
8 µM
6 µM
4 µM
2 µM
1 µM
0.5 µM
0.25% DMSO
untreated

**11**

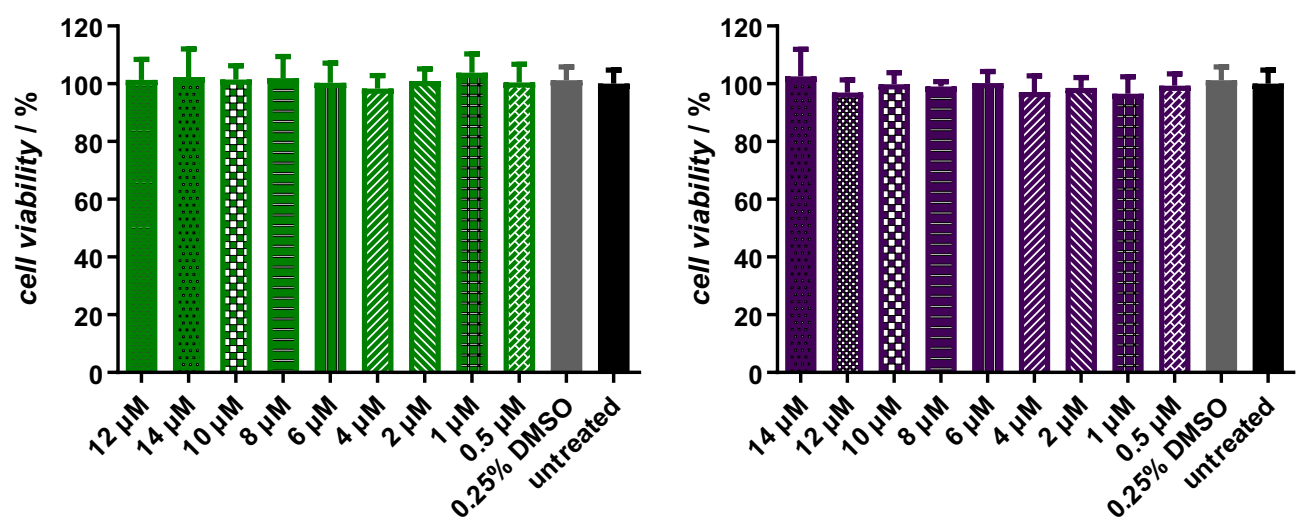
cell viability / %
0
20
40
60
80
100
120
12 µM
14 µM
10 µM
8 µM
6 µM
4 µM
2 µM
1 µM
0.5 µM
0.25% DMSO
untreated
cell viability / %
0
20
40
60
80
100
120
14 µM
12 µM
10 µM
8 µM
6 µM
4 µM
2 µM
1 µM
0.5 µM
0.25% DMSO
untreated

| | |
|---|---|
| 12 | 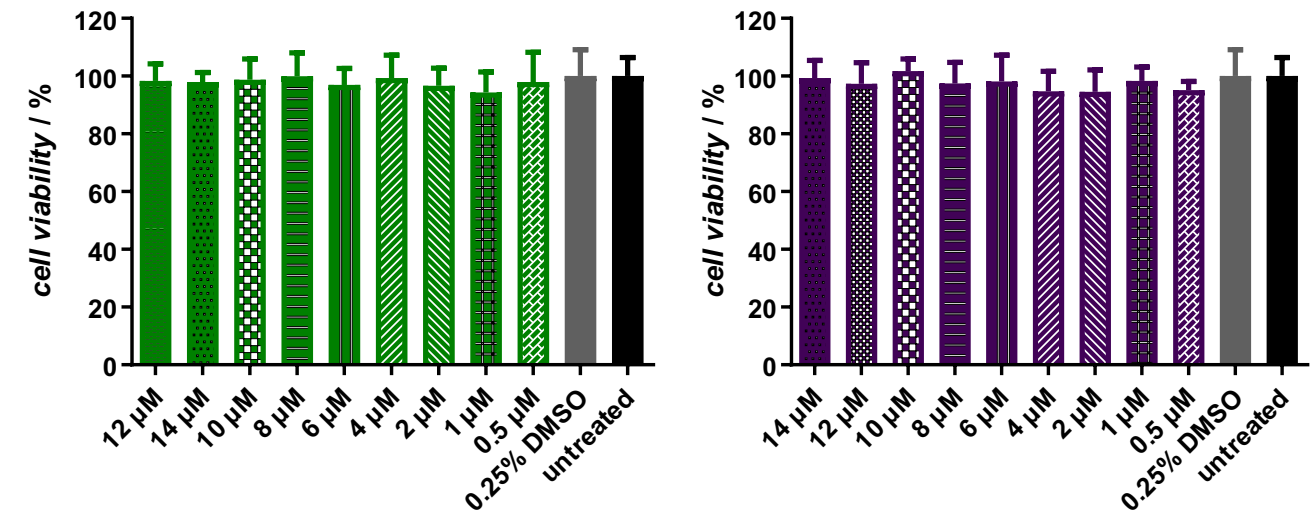  |

## Cell-based Luciferase Assay

Results of *firefly* luciferase assay in HEK293T-SMN2-Luc cells in presence of corresponding compound at different concentrations are summarized in table S8. Mean data-points and standard deviations are derived from three independent experiments, each concentration in triplicate. The curves were fitted by using log(agonist) vs. response (four parameters) function with software Graphpad Prism 8.0.

**Table S8**: Effect of SMN2 expression stimulation of different concentrations of corresponding compound. Mean data-points and standard deviations are derived from three independent experiments, each concentration in triplicate. Purple: treatment with PPS at 365 nm, green: treatment with PPS without irradiation.

| Compound | SMN2 expression stimulation |
|---|---|
| 2 | 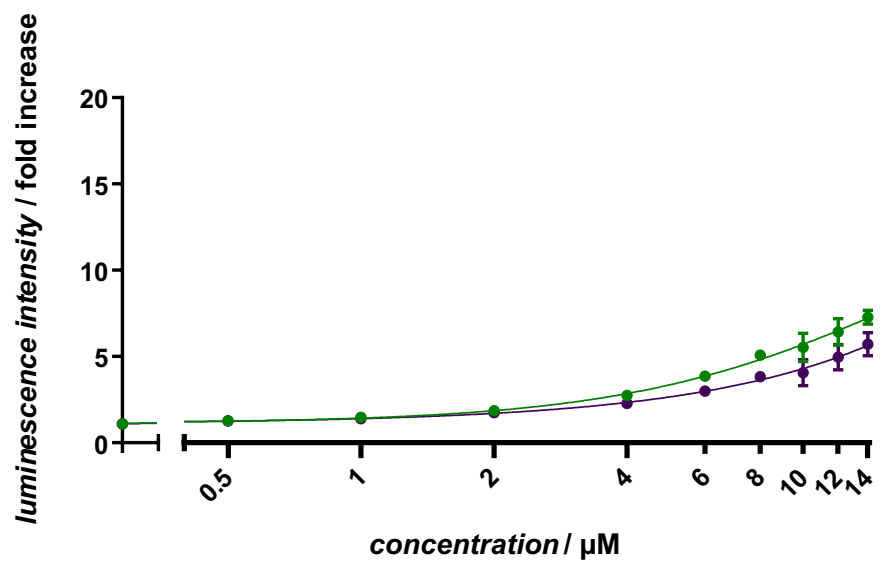  |
| 3 | 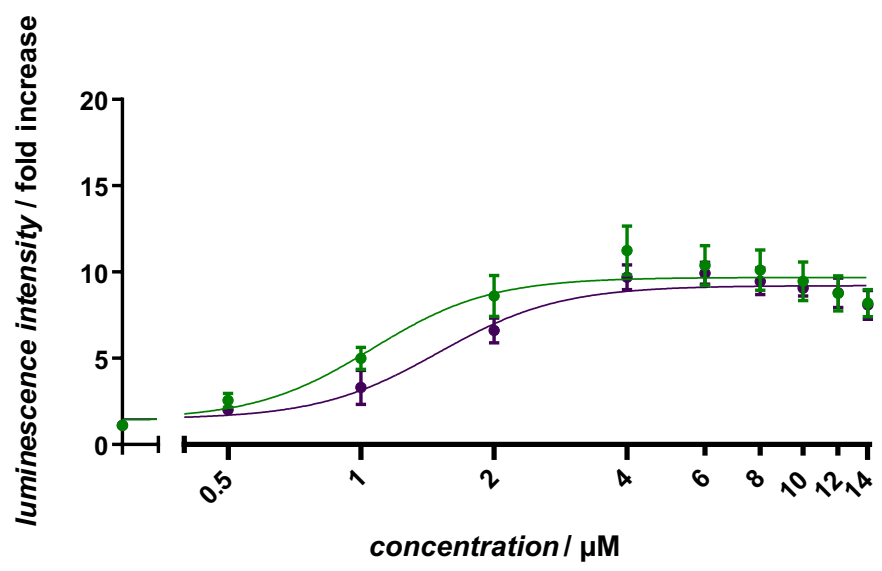  |
| 4 | 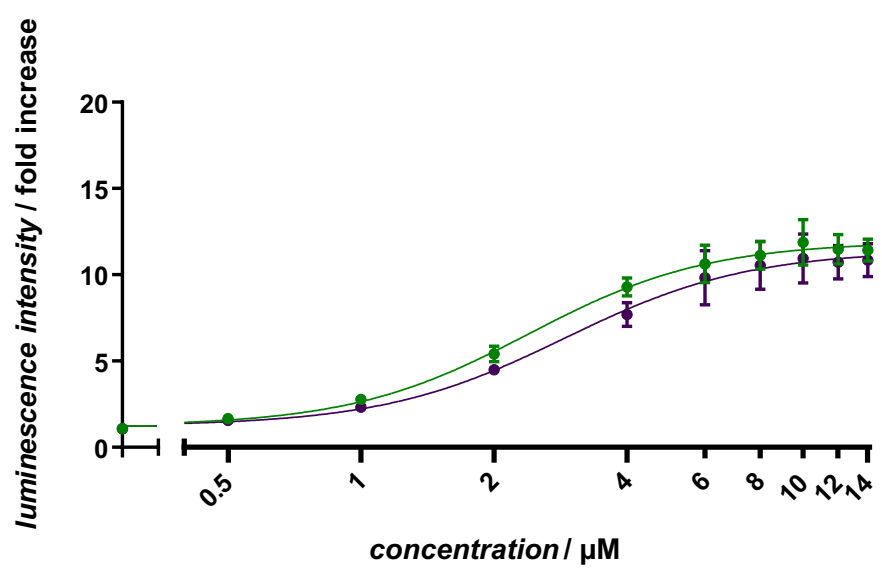  |

5

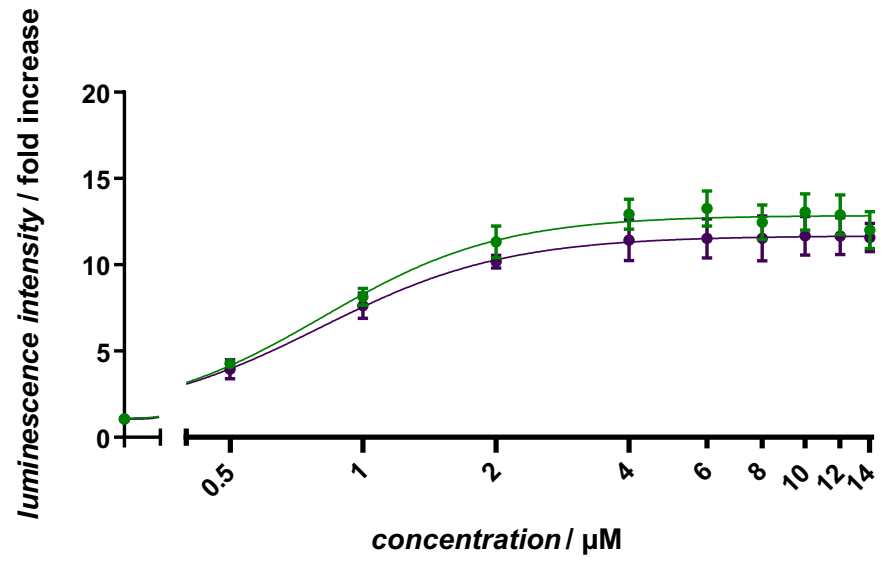
luminescence intensity / fold increase
0
5
10
15
20
0.5
1
2
4
6
8
10
12
14
concentration / µM

6

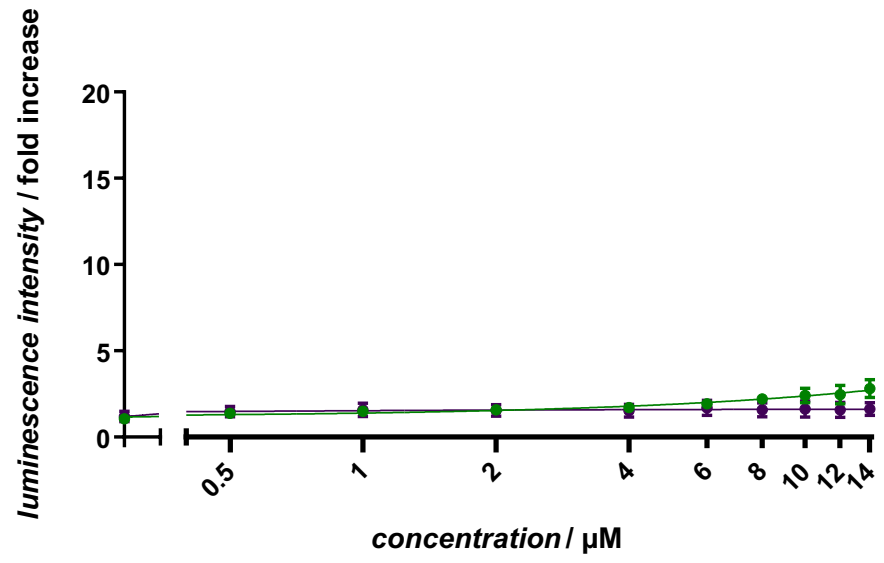
luminescence intensity / fold increase
0
5
10
15
20
0.5
1
2
4
6
8
10
12
14
concentration / µM

7

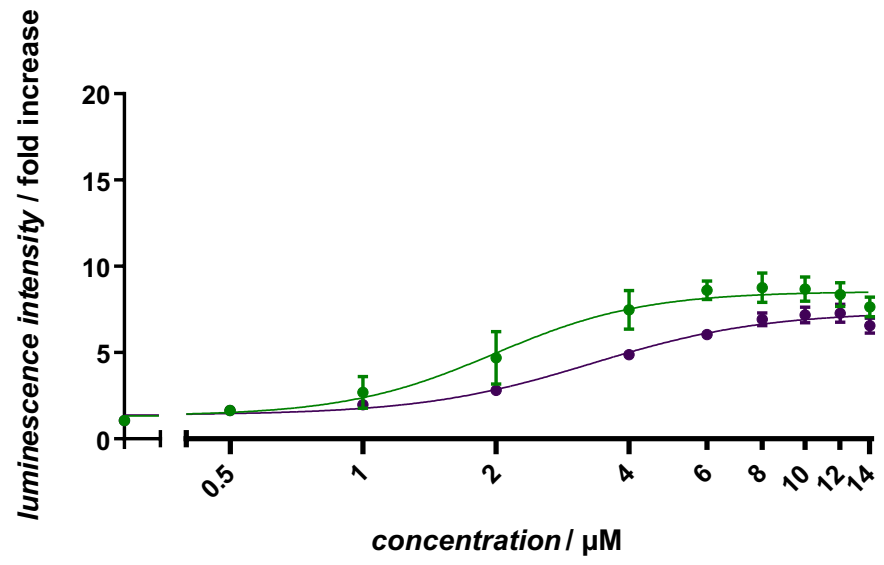
luminescence intensity / fold increase
0
5
10
15
20
0.5
1
2
4
6
8
10
12
14
concentration / µM

8

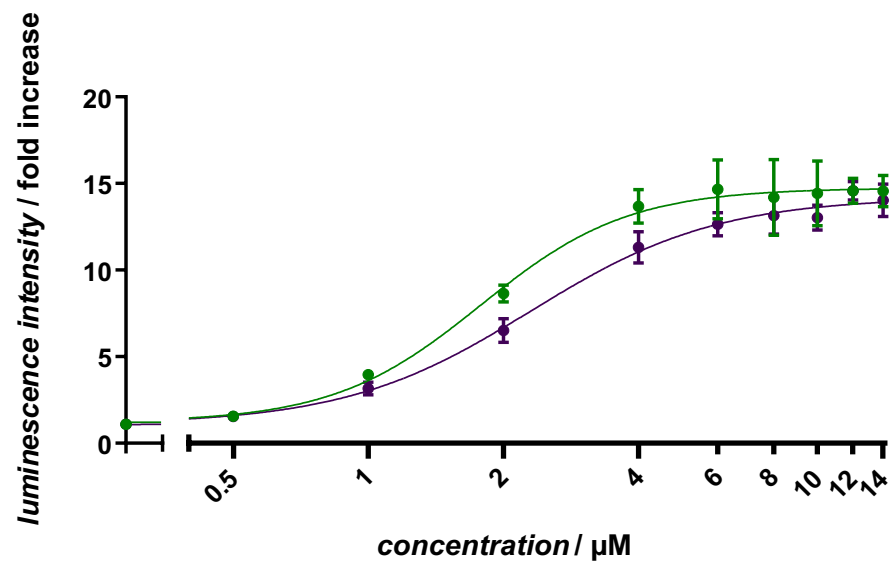
luminescence intensity / fold increase
0
5
10
15
20
0.5
1
2
4
6
8
10
12
14
concentration / µM

9

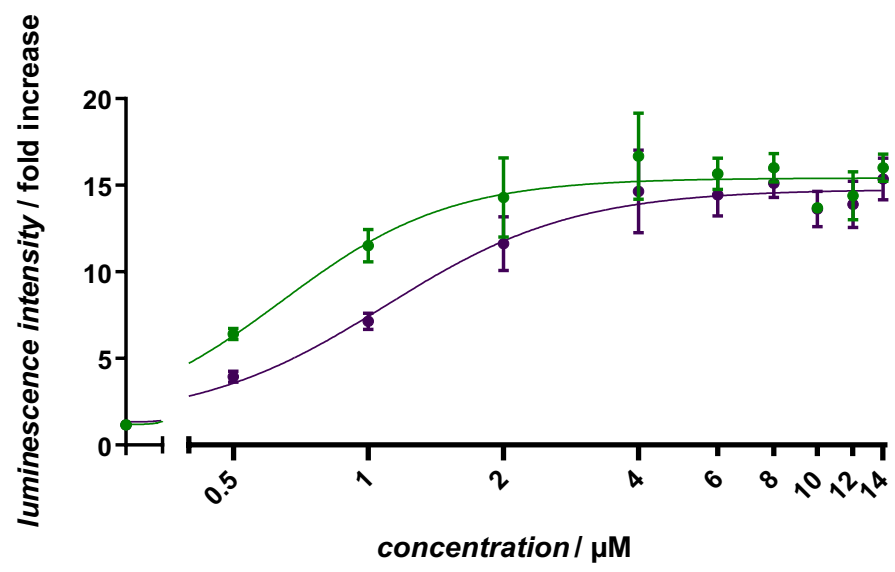
luminescence intensity / fold increase
0
5
10
15
20
0.5
1
2
4
6
8
10
12
14
concentration / µM

10

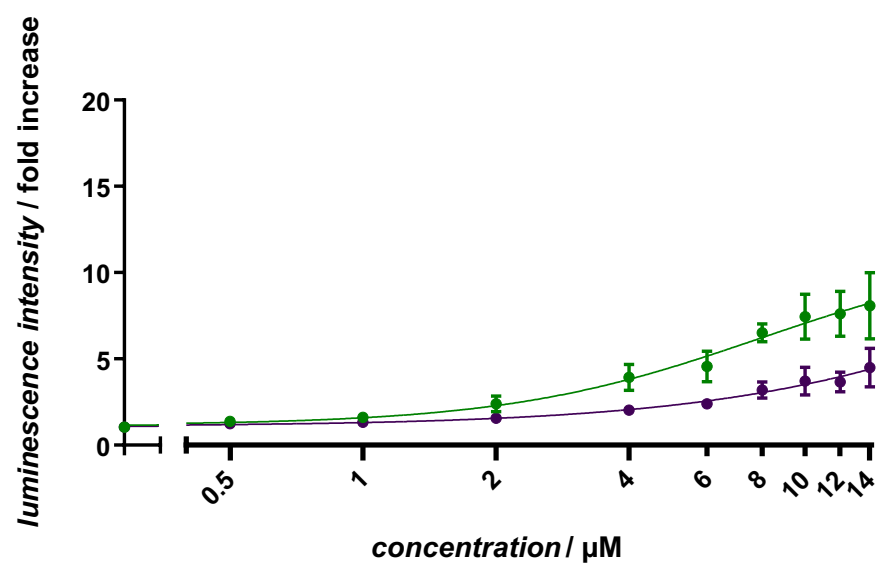
luminescence intensity / fold increase
0
5
10
15
20
0.5
1
2
4
6
8
10
12
14
concentration / µM

| | |
|---|---|
| **11** | 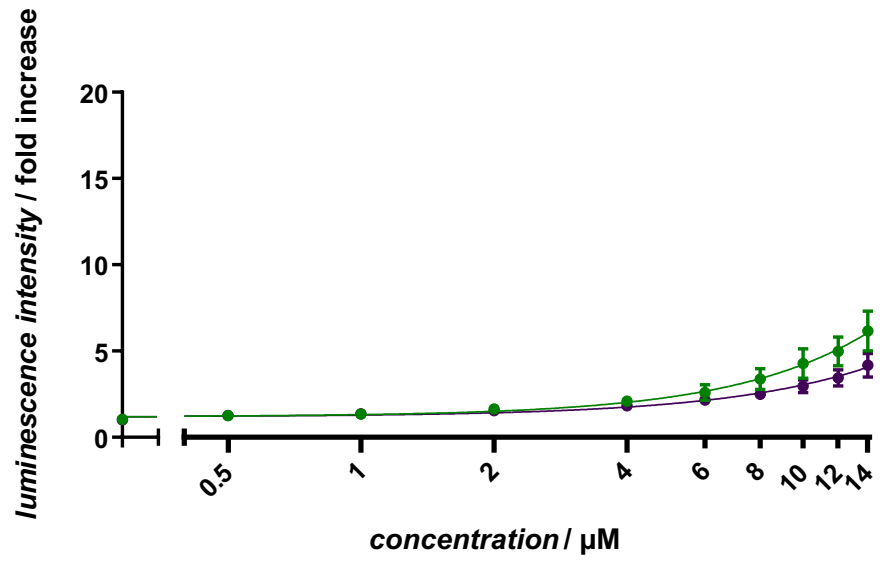luminescence intensity / fold increase
20
15
10
5
0
0.5
1
2
4
6
8
10
12
14
concentration / µM |
| **12** | 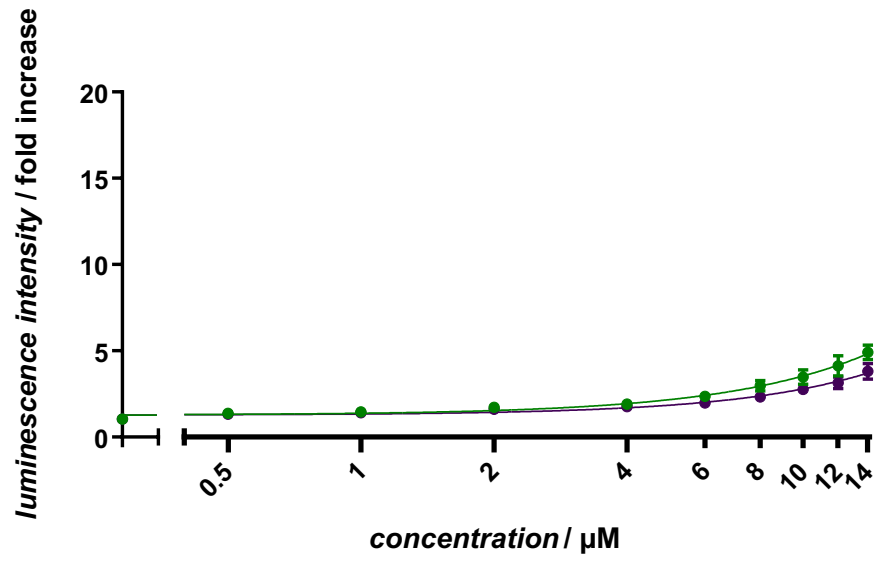luminescence intensity / fold increase
20
15
10
5
0
0.5
1
2
4
6
8
10
12
14
concentration / µM |

## Computational Model Generation

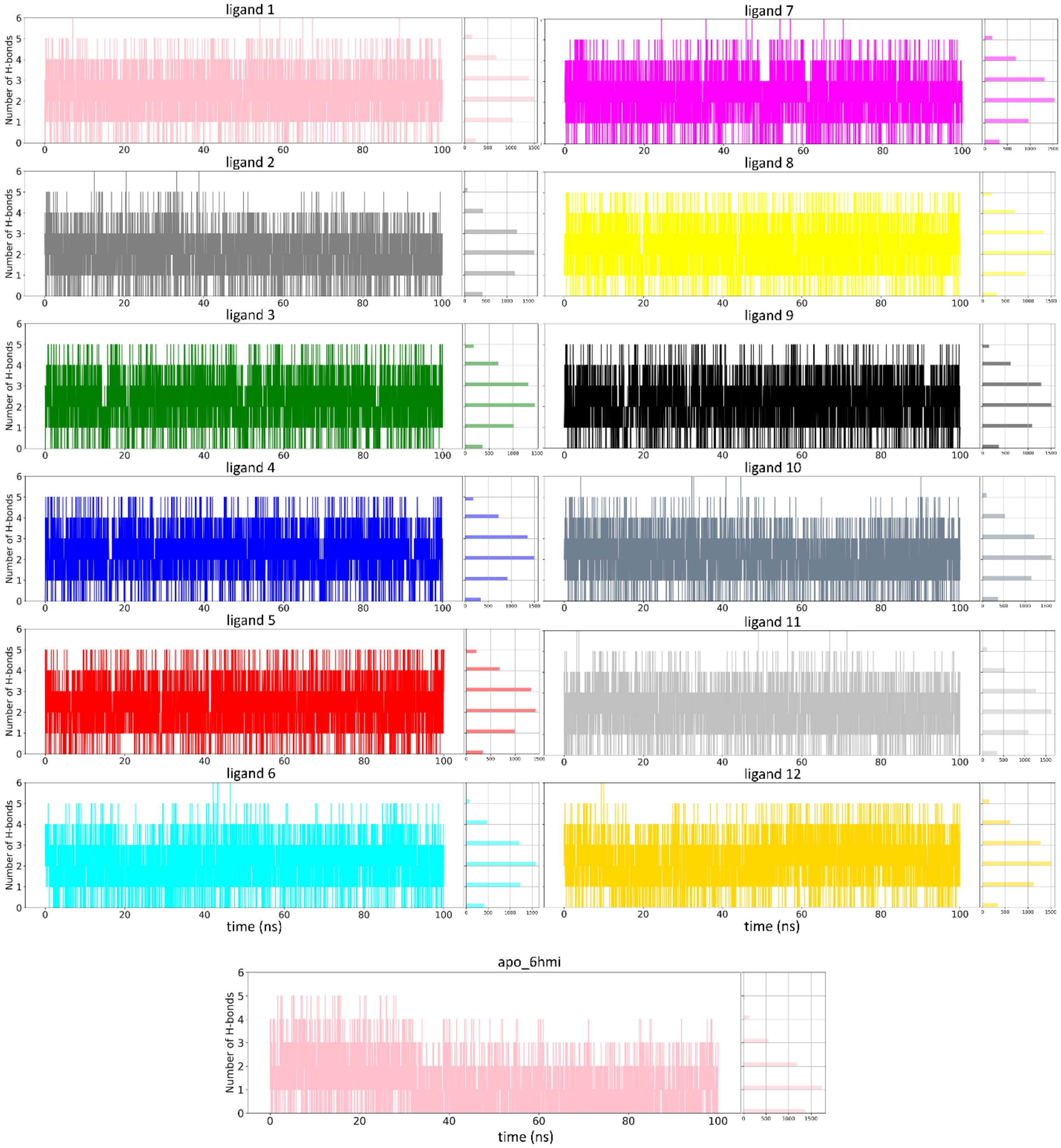


**Figure S95**. Comparison of number of established hydrogen bonds within of $G_{-3}$, $G_{-2}$, $A_{-1}/G_{11}$, $U_{10}$, $C_9$ segment of dsRNA for *holo* and *apo* during the course of molecular dynamics simulations.

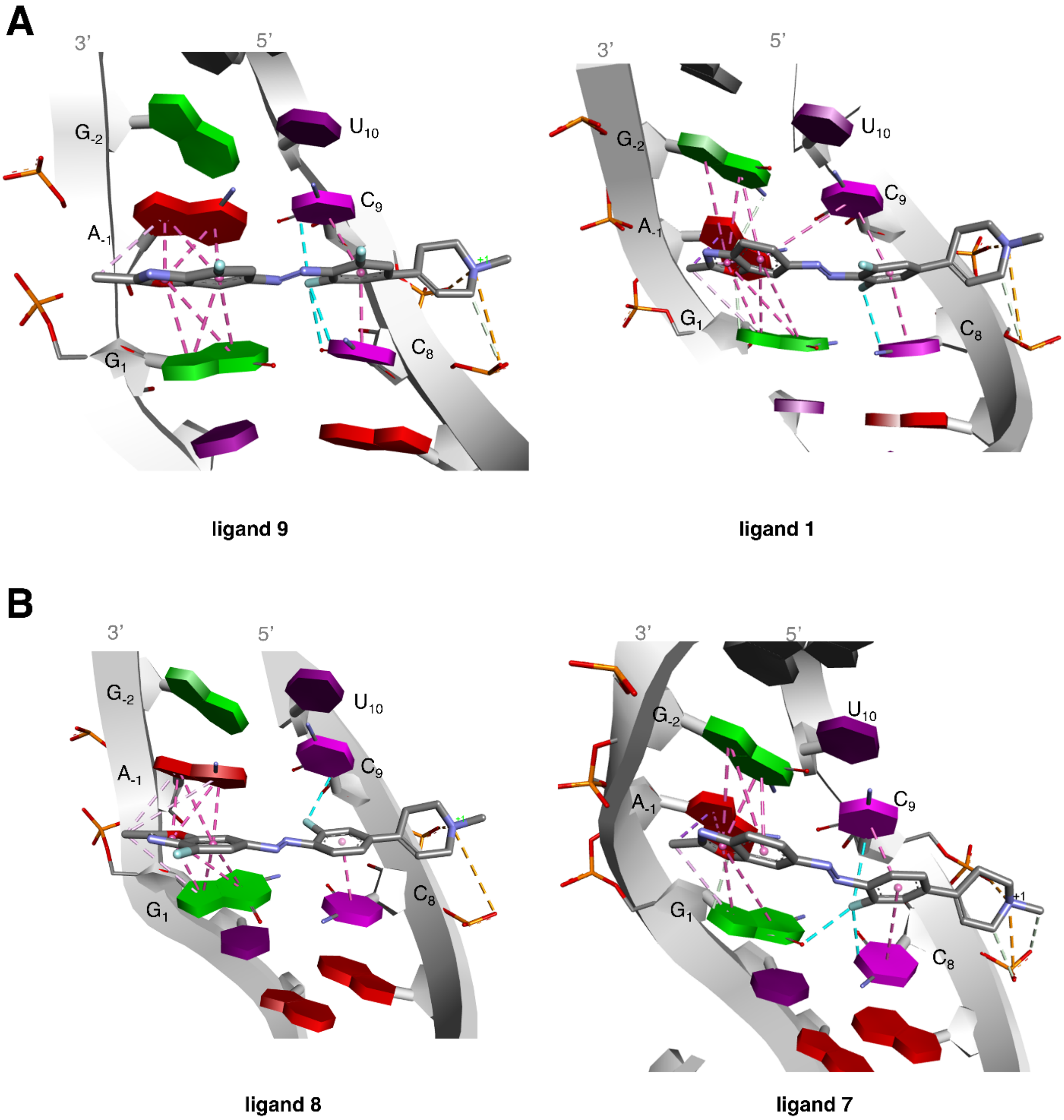
A
3’
5’
G-2
U10
A-1
C9
G1
C8
ligand 9
3’
5’
G-2
U10
A-1
C9
G1
C8
ligand 1
B
3’
5’
G-2
U10
A-1
C9
G1
C8
ligand 8
3’
5’
G-2
U10
A-1
C9
G1
C8
ligand 7

**C**

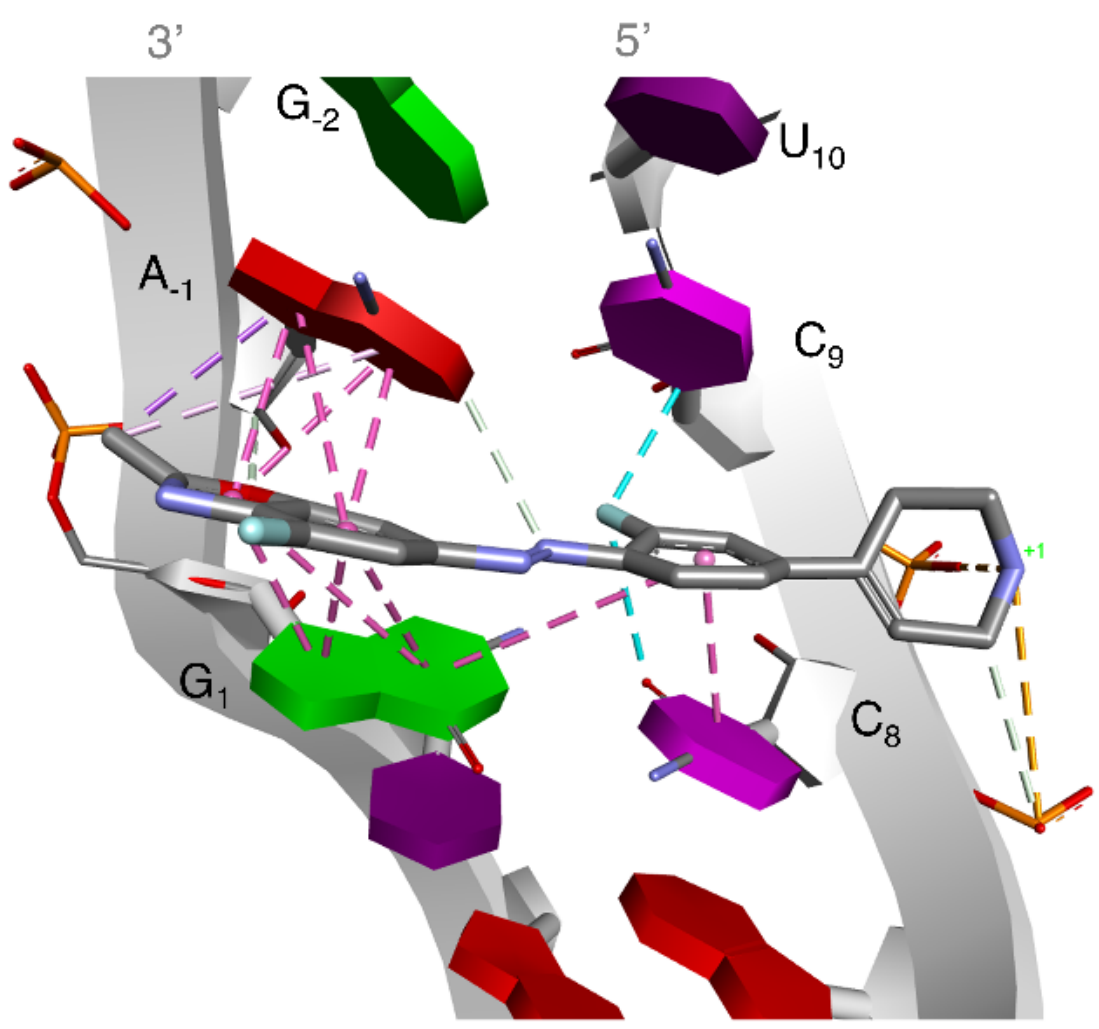


**ligand 5**

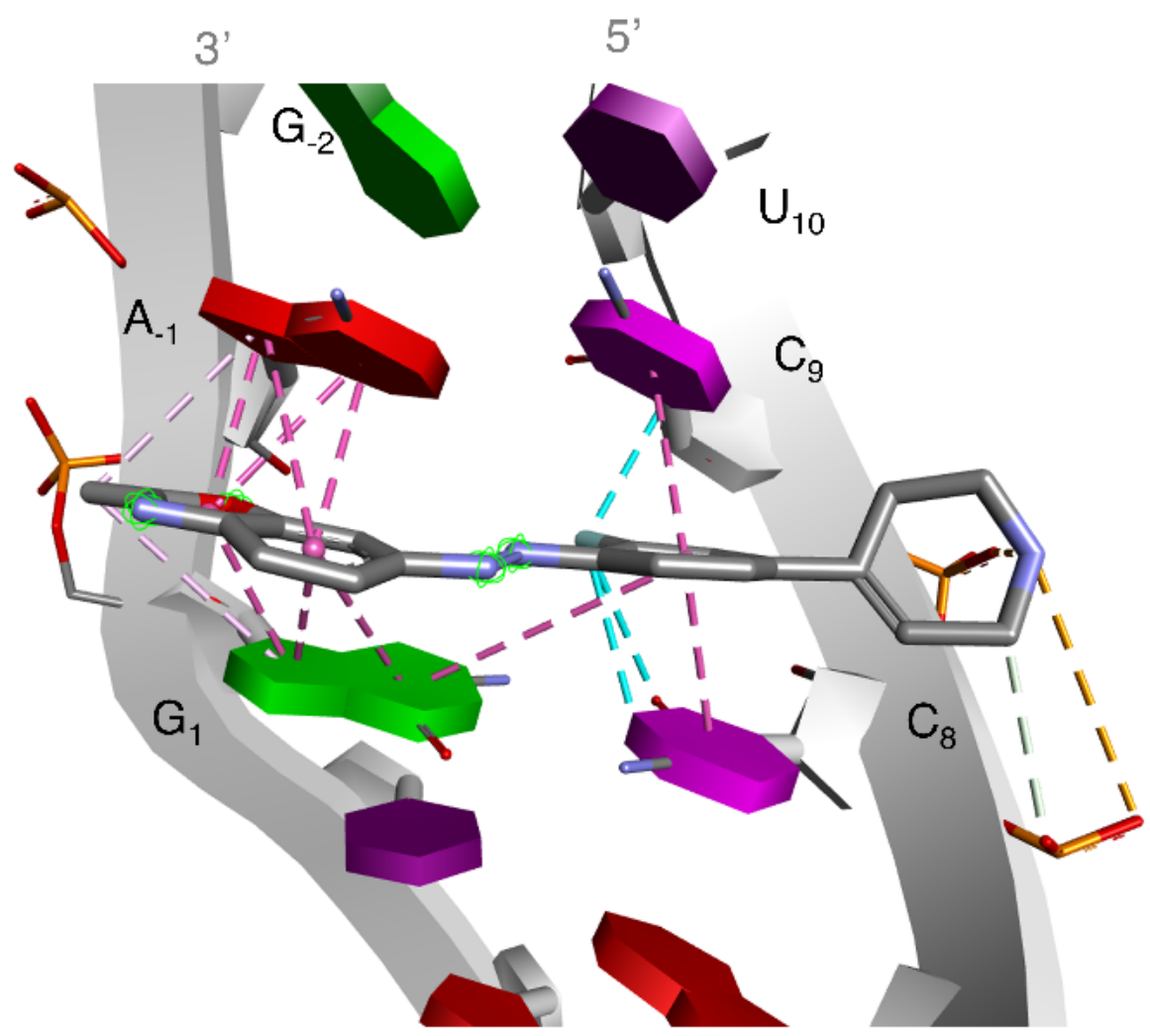


**ligand 3**

**D**

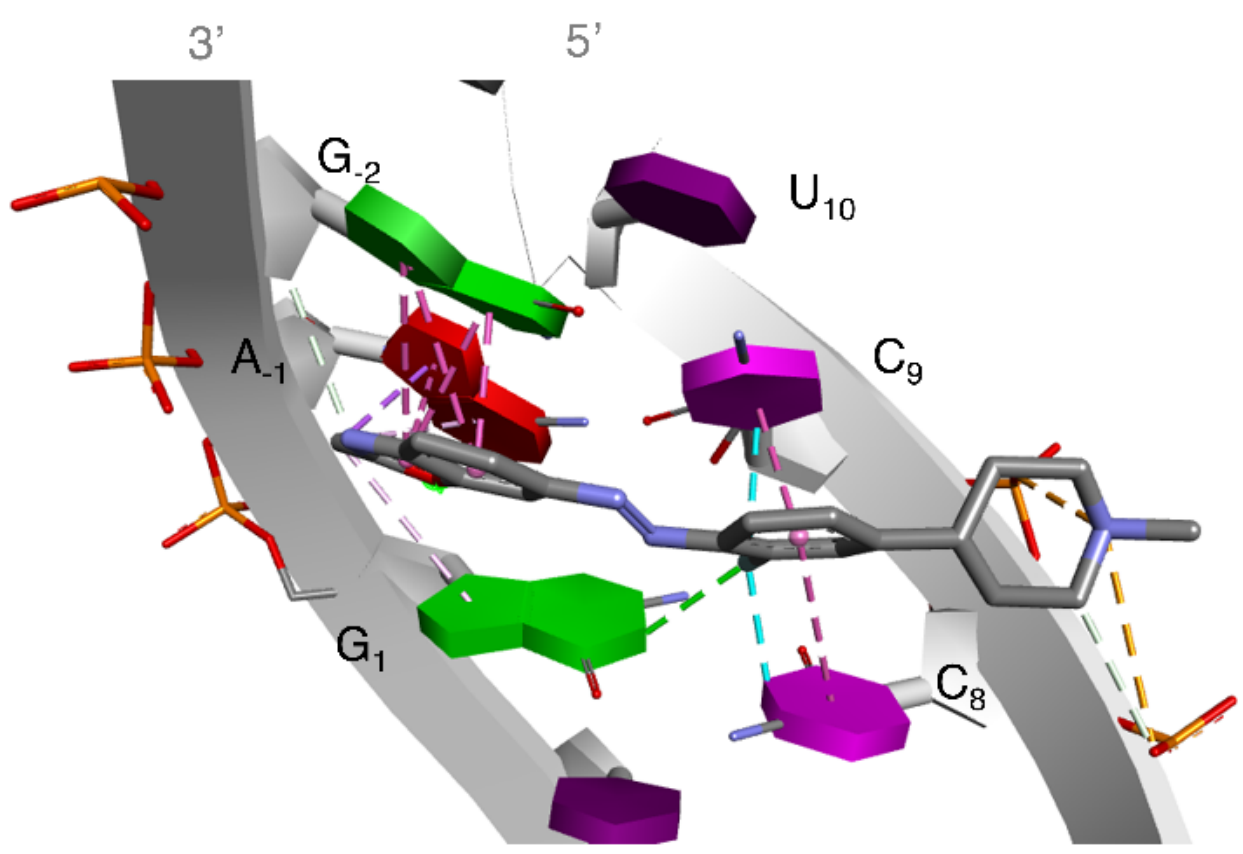


**ligand 10**

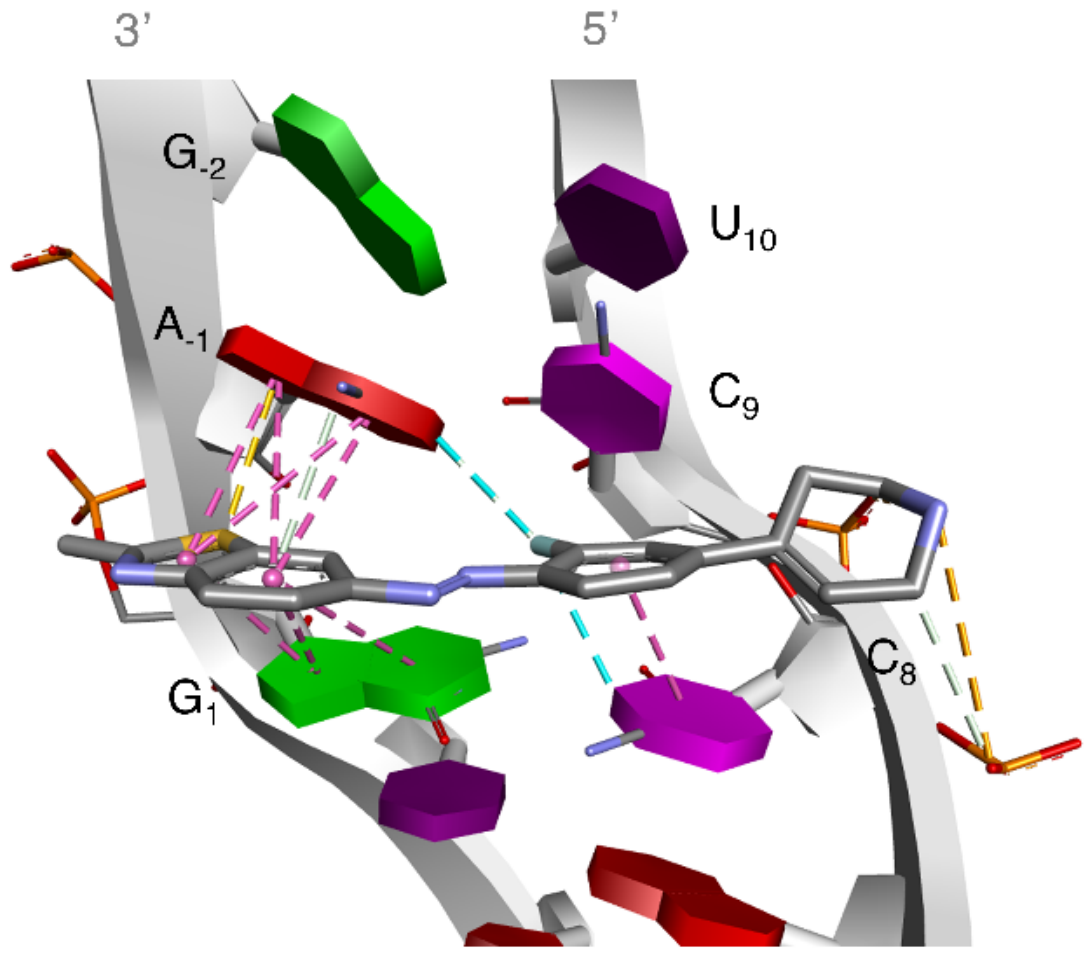


**ligand 4**

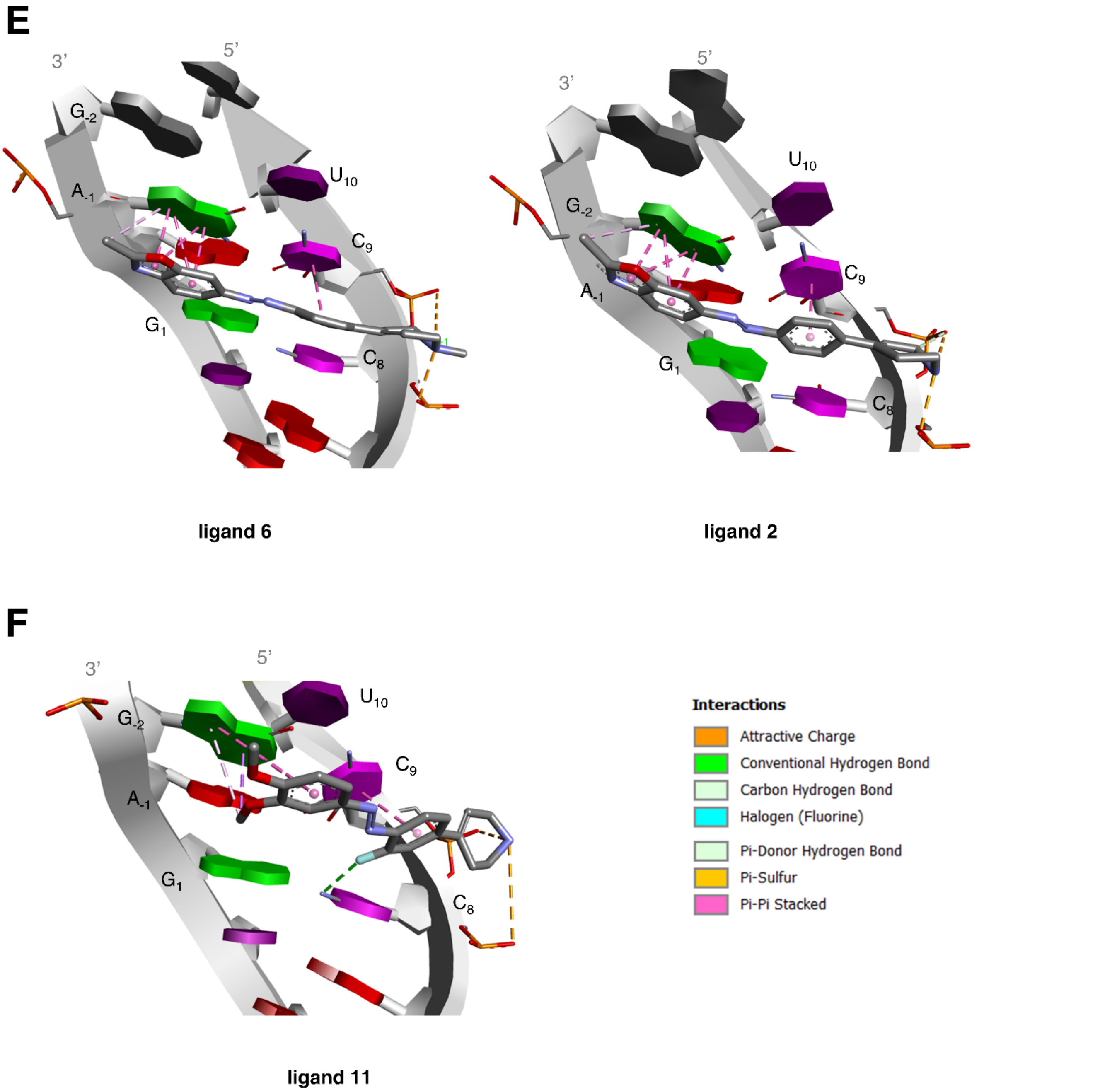


**Figure S96.** Pairwise comparisons of the predicted binding modes and intermolecular interactions between the most similar ligands from the library. Ligand 12 was excluded from analysis due to the unbinding from the active site during the course of molecular dynamics simulation.

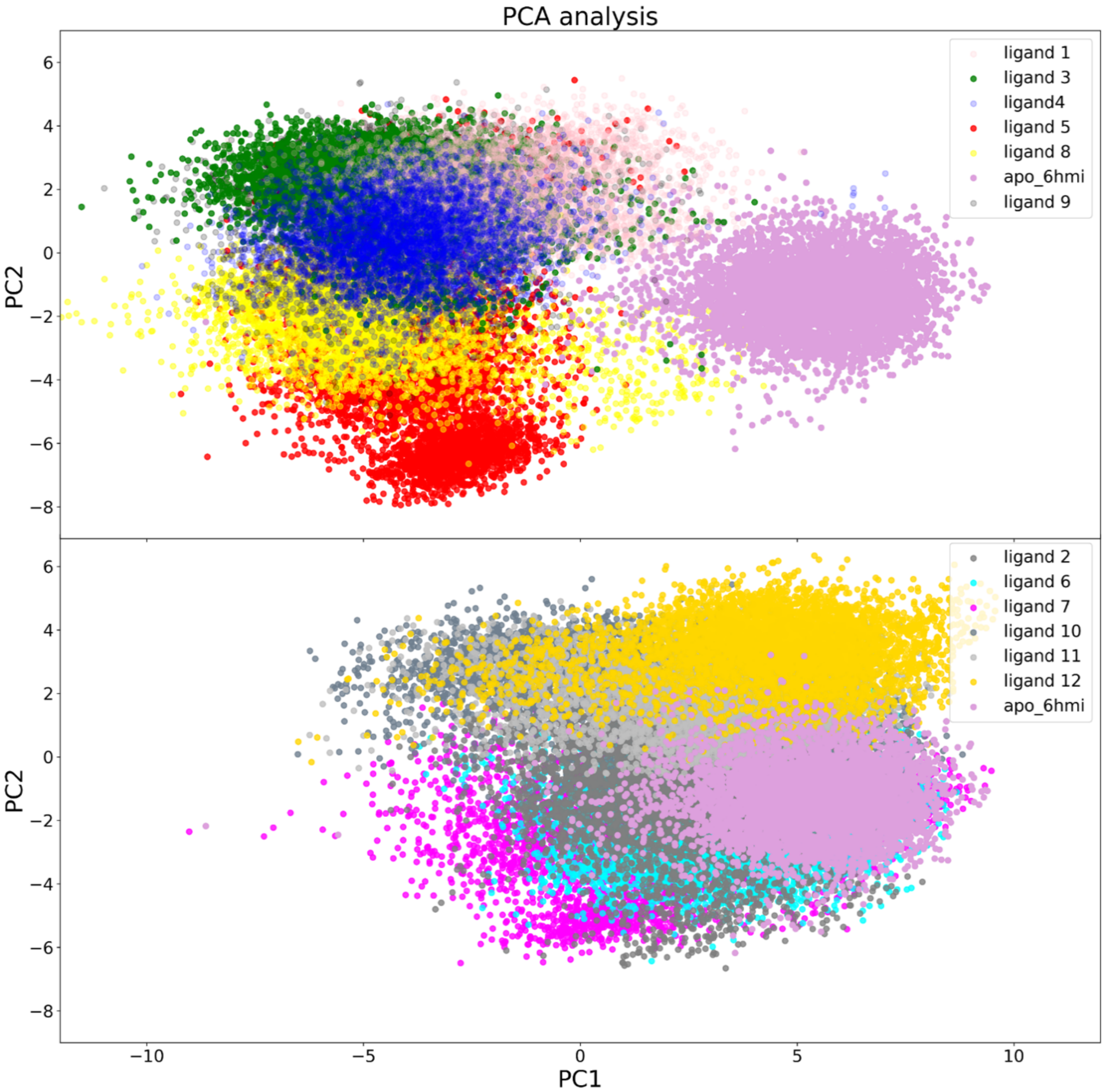


**Figure S97.** Results of principal component analysis of studied trajectories. Trajectories were projected on first two principal components calculated for all atoms in dsRNA. Upper plot indicates clear differentiation in conformational behavior between *apo* and *holo* states for the most potent ligands. Less potent ligands (lower plot) were partially overlapping with *apo* state.

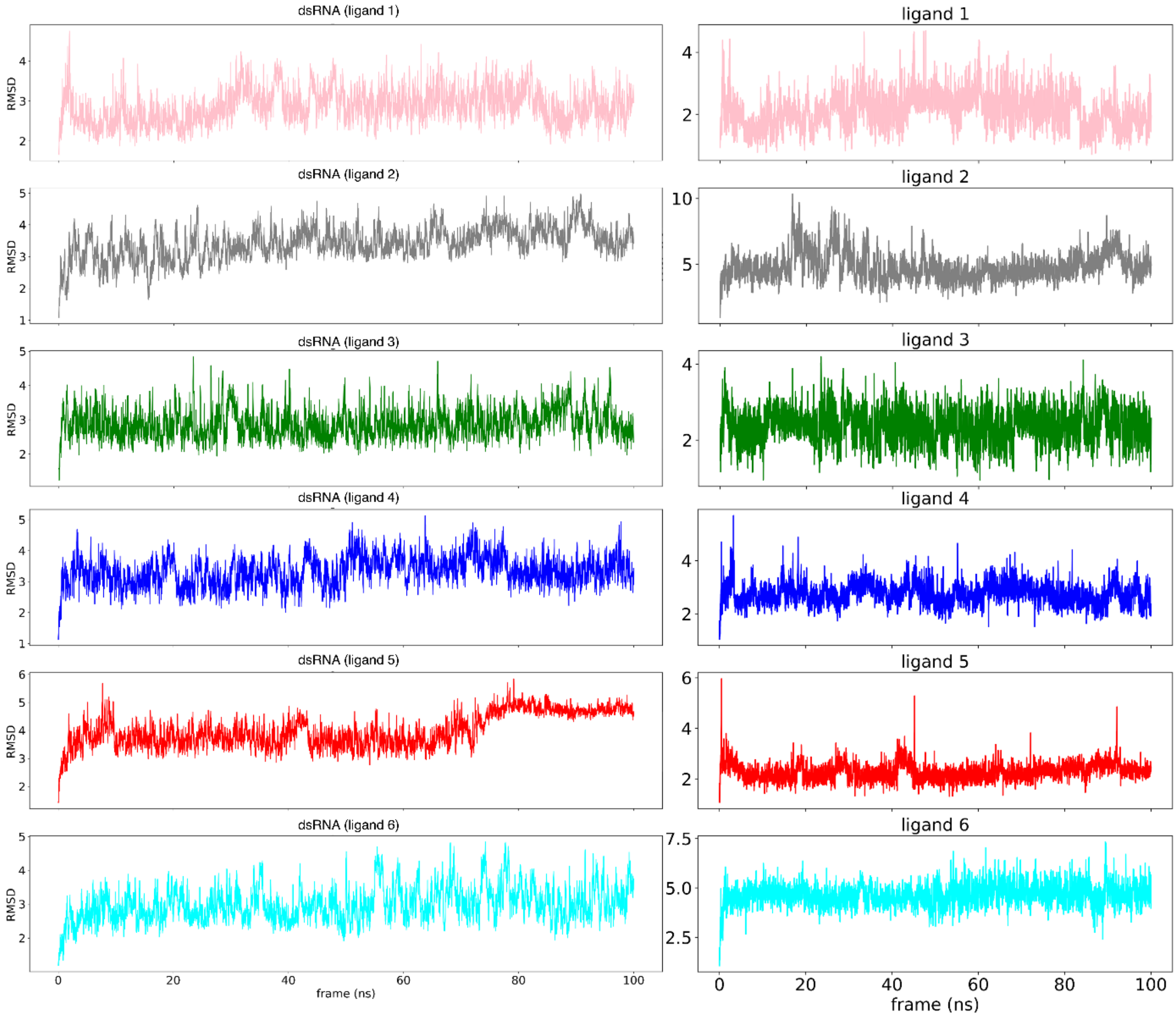
dsRNA (ligand 1)
ligand 1
dsRNA (ligand 2)
ligand 2
dsRNA (ligand 3)
ligand 3
dsRNA (ligand 4)
ligand 4
dsRNA (ligand 5)
ligand 5
dsRNA (ligand 6)
ligand 6
RMSD
frame (ns)

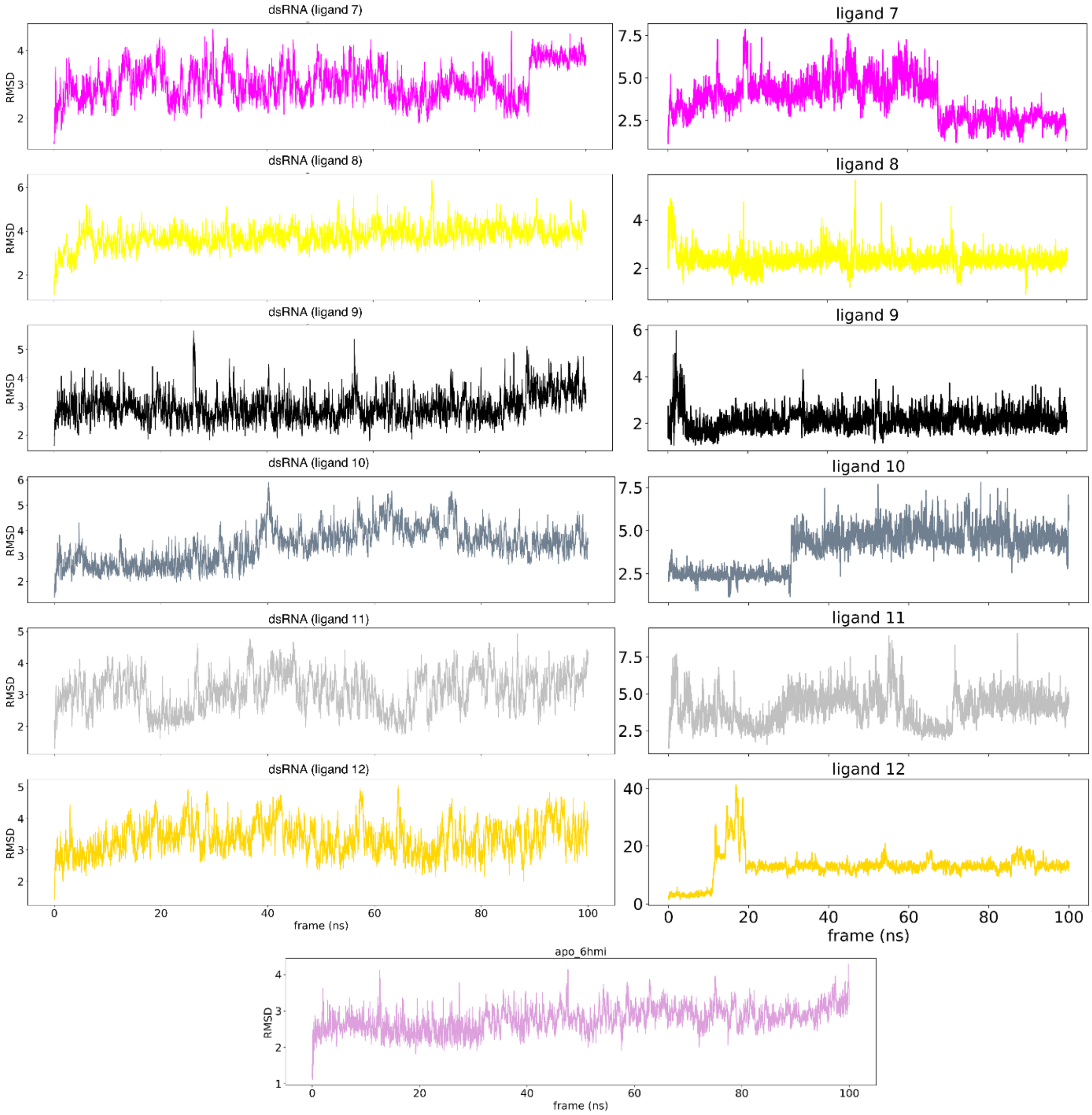


**Figure S98**. Root Mean Square Deviation (RMSD) of molecular dynamics trajectories calculated for dsRNA (left) and ligands (right). RMSD data indicated sampling from reasonably converged trajectories except ligand 12 which unbounded during the course of simulation (after several repeats – data not shown). Less potent ligands were less stable during the MD simulation.

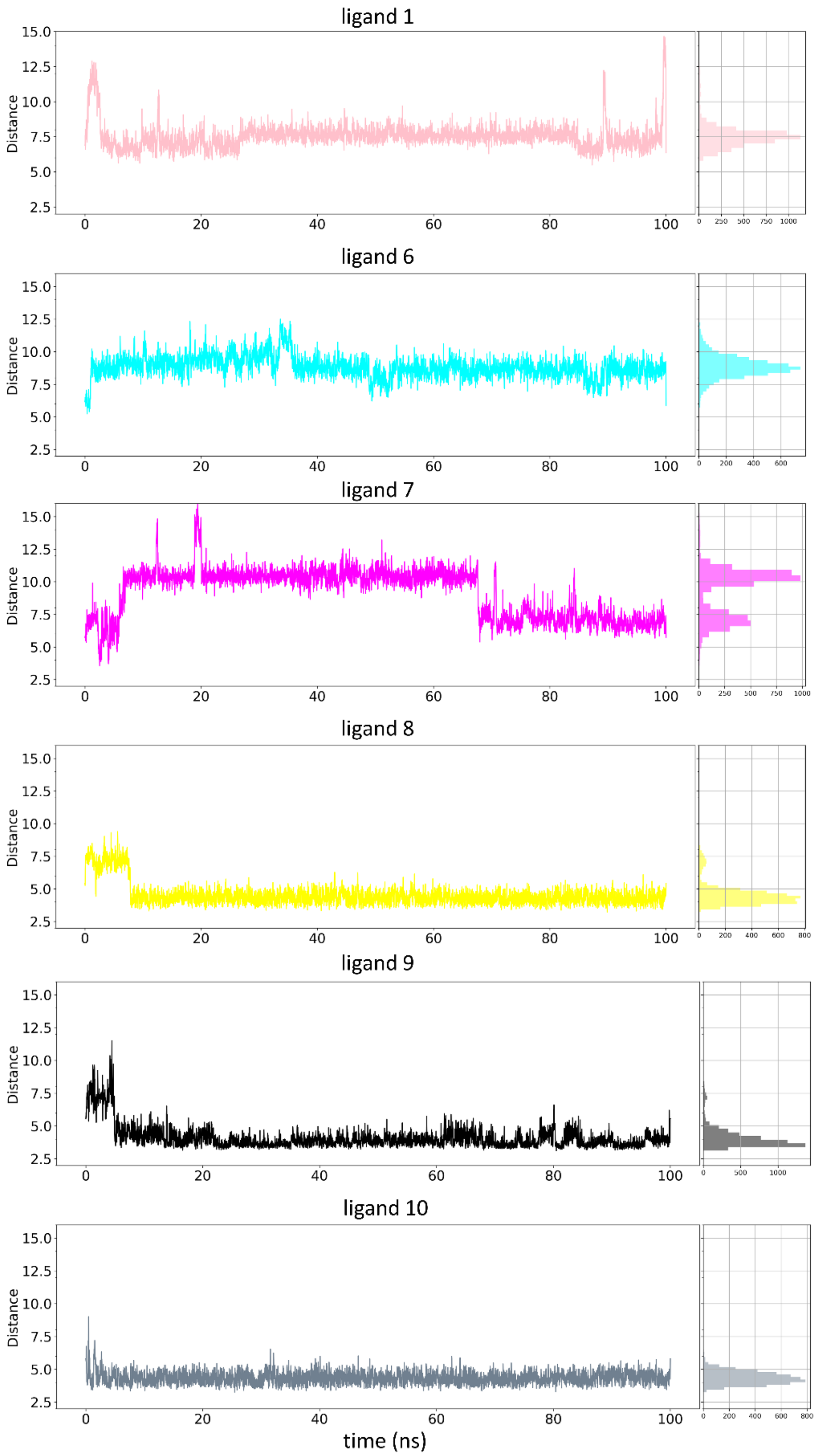


**Figure S99**. Comparison of distances (Å) between centers of masses for benzoxazole moiety and bulged adening $A_{-1}$ observed during molecular dynamics simulations for ligands containing tertiary amino group. Results indicated tight binding between $A_{-1}$ and ligands containing fluorinated benzoxazole.

**Table S9**: Predicted pKa values of secondary and tertiary amino groups for selected ligands. Calculations were performed with Marvin Suite v21.3.

| ID | **Predicted pKa value of amino moiety** |
|---|---|
| ligand 1 | 7.97 |
| ligand 2 | 9.57 |
| ligand 3 | 9.55 |
| ligand 4 | 9.55 |
| ligand 5 | 9.55 |
| ligand 6 | 8.34 |
| ligand 7 | 8.15 |
| ligand 8 | 8.15 |
| ligand 9 | 7.97 |
| ligand 10 | 8.78 |
| ligand 11 | 9.55 |
| ligand 12 | 9.55 |

## NMR-based Binding Site Mapping

The ligand binding site of compound **9** to E7_5'ss:U1 RNA was studied by NMR spectroscopy using 2D $^{1}$H,$^{1}$H-TOCSY and 2D $^{1}$H,$^{13}$C-HSQC experiments with ligand-induced RNA chemical shift perturbations as read-out. To account for isomer-specific binding effects of **9**, spectra were recorded in its thermal equilibrium, $PSS_{cis}$ and $PSS_{trans}$ via *in situ* photoirradiation in between measurements. Irradiation of the sample was performed in 200 ms intervals using a 355 nm (8 W) or 488 nm (5.5 W) laser connected to a cone-shaped stamp of a 5 mm Shigemi susceptibility-matched microtube via an optical fibre (optical coupling efficiency ~50%). The accumulation of *cis* or *trans* isomer was tracked by 1D $^{1}$H spectra recorded after each interval until reaching $PSS_{cis}$ or $PSS_{trans}$ with total irradiation times of 2,000 ms or 400 ms with the 355 nm or 488 nm laser. All NMR measurements were performed at 298 K and 800 MHz.
Samples were prepared in phosphate buffer (25 mM KPi, 50 mM KCl, pH 7.4) with 5% DMSO-$d_6$ at a final concentration of 500 µM RNA only or 200 µM RNA with 400 µM **9**.
The ligand-induced RNA chemical shift perturbations observed between the different spectra were quantified as euclidean distances (d) using the following equation: [18]

$$d = \sqrt{\frac{1}{2}(\delta^2{}_H + \alpha \times \delta^2{}_C)}$$

where δ = chemical shift difference of $^{1}$H … ratio of $^{13}$C and $^{1}$H (α = 0.25), and displayed as heatmap on the RNA secondary structure and as histogram.

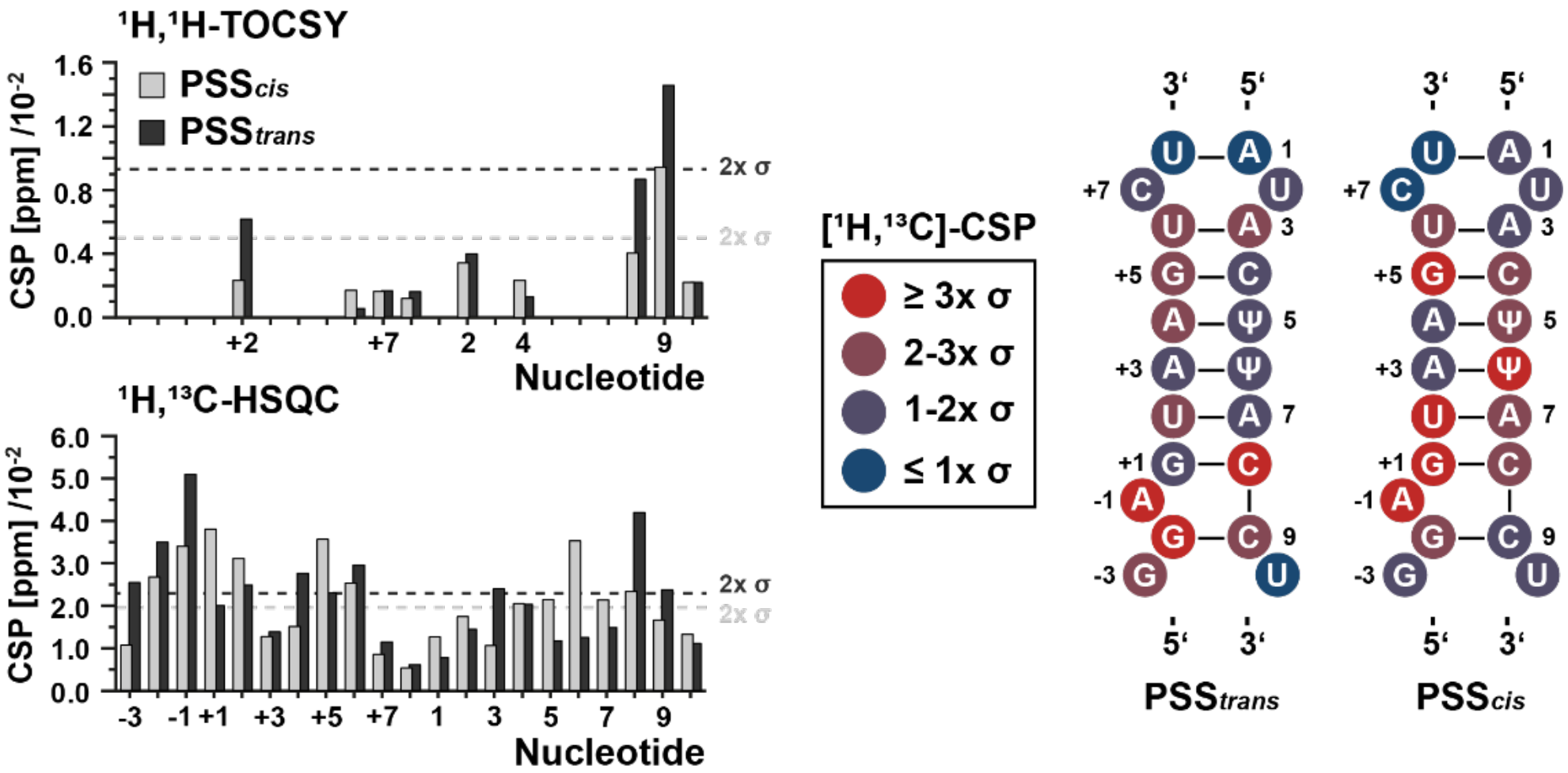


**Figure S100.** CSP-based histogram and heatmap of E7_5'ss:U1 RNA with compound **9**. CSPs were determined as euclidean distances between signals for RNA only and RNA with 2 eq **9** in its $PSS_{trans}$ (dark grey) and $PSS_{cis}$ (light grey). The histograms on the left show $^{1}$H,$^{1}$H-TOCSY- and $^{1}$H,$^{13}$C-HSQC-based CSPs for all detectable RNA signals. The $^{1}$H,$^{13}$C-HSQC-based CSPs are also shown as heatmap on the RNA secondary structure on the right. A threshold of twofold the standard deviation was used and is highlighted in the histograms. The heatmap was colored based on the CSP strength of individual signals compared to the total standard deviation.

## References


1 G. R. Fulmer, A. J. M. Miller, N. H. Sherden, H. E. Gottlieb, A. Nudelman, B. M. Stoltz, J. E. Bercaw and K. I. Goldberg, *Organometallics*, 2010, **29**, 2176–2179.

2 K. Stranius and K. Börjesson, *Sci Rep*, 2017, **7**, 41145.

3 L. Casimiro, L. Andreoni, J. Groppi, A. Credi, R. Métivier and S. Silvi, *Photochemical & Photobiological Sciences*, 2022, **21**, 825–833.

4 M. Zhang, C. Lorson, E. Androphy and J. Zhou, *Gene Ther*, 2001, **8**, 1532–1538.

5 J. O'Brien, I. Wilson, T. Orton and F. Pognan, *Eur J Biochem*, 2000, **267**, 5421–5426.

6 J. M. Baker and F. M. Boyce, *Journal of Visualized Experiments*, 2014, 1–10.

7 Marvin 21.3.0; ChemAxon. <https://Www.Chemaxon.Com>

8 *Gaussian, Inc., Wallingford CT*, 2016.

9 J. C. Cole, O. Korb, P. McCabe, M. G. Read and R. Taylor, *J Chem Inf Model*, 2018, **58**, 615–629.

10 L. Z. Sun, Y. Jiang, Y. Zhou and S. J. Chen, *J Chem Theory Comput*, 2020, **16**, 7173–7183.

11 D. Van Der Spoel, E. Lindahl, B. Hess, G. Groenhof, A. E. Mark and H. J. C. Berendsen, *J Comput Chem*, 2005, 26, 1701–1718.

12 D. Vassetti, M. Pagliai and P. Procacci, *J Chem Theory Comput*, 2019, **15**, 1983–1995.

13 P. Duchstein, C. Neiss, A. Görling and D. Zahn, *J Mol Model*, 2012, **18**, 2479–2482.

14 H. Bekker and H. J. C. Berendsen, *Article in Journal of Computational Chemistry*, , DOI:10.1002/(SICI)1096-987X(199709)18:123.0.CO;2-H.

15 B.-C. Yu, Y. Shirai and J. M. Tour, *Tetrahedron*, 2006, **62**, 10303–10310.

16 D. W. Engers, A. Y. Frist, C. W. Lindsley, C. C. Hong and C. R. Hopkins, *Bioorg Med Chem Lett*, 2013, **23**, 3248–3252.

17 H. Ratni, M. Ebeling, J. Baird, S. Bendels, J. Bylund, K. S. Chen, N. Denk, Z. Feng, L. Green, M. Guerard, P. Jablonski, B. Jacobsen, O. Khwaja, H. Kletzl, C.-P. Ko, S. Kustermann, A. Marquet, F. Metzger, B. Mueller, N. A. Naryshkin, S. V. Paushkin, E. Pinard, A. Poirier, M. Reutlinger, M. Weetall, A. Zeller, X. Zhao and L. Mueller, *J Med Chem*, 2018, **61**, 6501–6517.

18 M. M. Getz, A. J. Andrews, C. A. Fierke, H. M. Al-Hashimi, *RNA (New York, N.Y.)*, 2007, **13**, 251.
